# Resurrected Ancestral Cannabis Enzymes Unveil the Origin and Functional Evolution of Cannabinoid Synthases

**DOI:** 10.1111/pbi.70475

**Published:** 2025-12-26

**Authors:** Cloé Villard, Idil Baser, Arjen C. van de Peppel, Katarina Cankar, M. Eric Schranz, Robin van Velzen

**Affiliations:** ^1^ Biosystematics Group Wageningen University and Research Wageningen the Netherlands; ^2^ Horticulture and Product Physiology Group Wageningen University and Research Wageningen the Netherlands; ^3^ Business Unit Bioscience, Wageningen Plant Research Wageningen University & Research Wageningen the Netherlands

**Keywords:** ancestral sequence reconstruction, cannabinoid oxidocyclases, *Cannabis sativa* L, enzyme molecular mechanism, functional evolution, rational engineering

## Abstract

Cannabinoids, such as tetrahydrocannabinolic acid (THCA), cannabidiolic acid (CBDA) and cannabichromenic acid (CBCA), are bioactive and medicinally relevant compounds found in the cannabis plant ( *Cannabis sativa* L.). These three compounds are synthesised from a single precursor, cannabigerolic acid (CBGA), through regioselective reactions catalysed by different cannabinoid oxidocyclase enzymes. Despite the importance of cannabinoid oxidocyclases for determining cannabis chemotype and properties, the functional evolution and molecular mechanism of this enzyme family remain poorly understood. To address this gap, we combined ancestral sequence reconstruction and heterologous expression to resurrect and functionally characterise three ancestral cannabinoid oxidocyclases. Results showed that the ability to metabolise CBGA originated in a recent ancestor of cannabis and that early cannabinoid oxidocyclases were promiscuous enzymes producing all three THCA, CBDA and CBCA. Gene duplication and diversification later facilitated enzyme subfunctionalisation, leading to extant, highly‐specialised THCA and CBDA synthases. Through rational engineering of these ancestors, we designed hybrid enzymes which allowed identifying key amino acid mutations underlying the functional evolution of cannabinoid oxidocyclases. Ancestral and hybrid enzymes also displayed unique activities and proved to be easier to produce heterologously than their extant counterparts. Overall, this study contributes to understanding the origin, evolution and molecular mechanism of cannabinoid oxidocyclases, which opens new perspectives for breeding, biotechnological and medicinal applications.

## 1 Introduction

**FIGURE 1:**
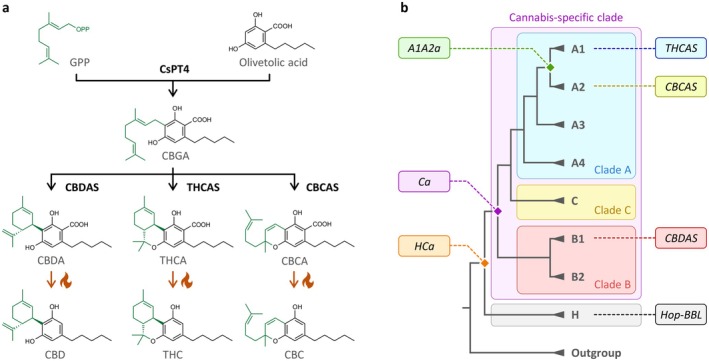
Cannabinoid biosynthesis in *Cannabis sativa* . (a) Cannabinoid biosynthesis pathway. Enzymatic reactions are symbolised with black arrows, nonenzymatic decarboxylations with orange arrows and delta symbols. (b) Simplified phylogeny of Berberine Bridge‐Like (BBL) enzymes. Clades A (blue), B (red) and C (yellow) form the *Cannabis*‐specific clade (purple), as defined previously (van Velzen and Schranz 2021). Clade H (grey) comprises uncharacterised *C. sativa* sequences as well as the *Humulus lupulus* sequence referred to as Hop‐BBL. Extant *THCAS*, *CBCAS*, *CBDAS* and *Hop‐BBL* genes are detailed on the right; internal nodes corresponding to ancestral *A1A2a*, *Ca* and *HCa* are on the left. Abbreviated molecules: CBC, cannabichromene; CBCA, cannabichromenic acid; CBD, cannabidiol; CBDA, cannabidiolic acid; CBGA, cannabigerolic acid; FAD, flavin adenine dinucleotide; GPP, geranyl pyrophosphate; THC, (−)‐trans‐Δ9‐tetrahydrocannabinol; THCA, (−)‐trans‐Δ9‐tetrahydrocannabinolic acid. Abbreviated enzymes: CBCAS, CBCA synthase; CBDAS, CBDA synthase; CsPT4, *C. sativa* prenyltransferase 4; THCAS, THCA synthase.

Cannabinoids are specialised metabolites produced by the plant *Cannabis sativa* L. (cannabis). The most abundant and well‐studied cannabinoids are (−)‐trans‐Δ9‐tetrahydrocannabinol (THC) and cannabidiol (CBD). THC is primarily responsible for cannabis psychotropic effects, but it can also alleviate chronic pain, inflammation and nausea (Costa 2007; Jeddi et al. 2024). Contrarily, CBD is non‐psychotropic and exhibits therapeutic potential in managing anxiety, depression and epilepsy (Aderinto et al. 2024; Han et al. 2024). Over 120 other cannabinoids have been identified in cannabis, including cannabichromene (CBC), which may contribute to neuroprotection and modulating inflammation (Stone et al. 2020; Cammà et al. 2025). Given their medicinal relevance, the biosynthetic pathway of THCA, CBDA and CBCA has been fully elucidated and associated biosynthetic genes were identified (Figure 1a). Briefly, cannabinoid biosynthesis begins with the formation of olivetolic acid (Taura et al. 2009; Stout et al. 2012; Gagne et al. 2012) and its prenylation into cannabigerolic acid (CBGA) (Fellermeier and Zenk 1998; Luo et al. 2019). CBGA is then converted into (−)‐trans‐Δ9‐tetrahydrocannabinolic acid (THCA), cannabidiolic acid (CBDA), or cannabichromenic acid (CBCA) by regioselective cannabinoid oxidocyclases named THCA synthase (THCA) (Taura et al. 1995; Sirikantaramas et al. 2004), CBDA synthase (CBDAS) (Taura et al. 1996, 2007) and CBCA synthase (CBCAS) (Morimoto et al. 1998; Laverty et al. 2019), based on their main product selectivity. Resulting cannabinoid acids can then undergo nonenzymatic decarboxylation (e.g., via heat exposure) to yield their neutral counterparts (Figure 1a). Due to the regioselectivity of cannabinoid oxidocyclase enzymes, the presence/absence and relative expression of associated genes control cannabis chemotype and therapeutic potential (Gülck and Møller 2020).

Cannabinoid oxidocyclases are members of the Berberine Bridge‐Like (BBL) enzyme family (Sirikantaramas et al. 2004; Taura et al. 2007), known for catalysing chemically challenging oxidoreductions via bi‐covalent attachment to their flavin adenine dinucleotide (FAD) cofactor (Daniel et al. 2017). Over the past 20 years, cannabinoid oxidocyclase enzymes have been investigated through targeted mutagenesis and crystallisation experiments. This led to the identification of residues involved in catalysis, substrate or FAD binding (Sirikantaramas et al. 2004; Taura et al. 2007; Shoyama et al. 2012; Zirpel et al. 2018; Villard et al. 2023; Dai et al. 2024), and to the proposal of catalytic mechanisms for CBGA conversion into THCA (Shoyama et al. 2012; Villard et al. 2023). Despite these valuable advances, it is still unclear how exactly cannabinoid oxidocyclases interact with CBGA and what controls their product selectivity, meaning that key residues are yet to be identified.

More recently, comparative genomics has revealed that the *THCAS*, *CBDAS* and *CBCAS* genes originated from recent gene duplications within the *Cannabis* lineage, thus forming a cannabis‐specific clade (Figure 1b) that is absent from *Humulus lupulus* L. (hop), a close cannabis relative (Vergara et al. 2019; van Velzen and Schranz 2021). This cannabis‐specific clade comprises three main clades (A–C) and seven subclades (A1–A4, B1–B2, C) (van Velzen and Schranz 2021). *THCAS* and *CBCAS* belong to subclades A1 and A2, respectively, and share 96% nucleotide identity. *CBDAS* belongs to subclade B1 and shares 89% identity with clade A. Other subclades contain uncharacterised genes and pseudogenes. Interestingly, the sister of the cannabis‐specific clade—hereafter referred to as clade H—comprises several cannabis sequences and a single uncharacterised hop gene (van Velzen and Schranz 2021), referred to as *Hop‐BBL* (Figure 1b). Given that all characterised enzymes from the cannabis‐specific clade can metabolise CBGA, and that hop does not produce cannabinoids, it was previously hypothesized that CBGA metabolisation originated within the *Cannabis* lineage (Vergara et al. 2019; van Velzen and Schranz 2021). However, this has never been experimentally verified, meaning that the origin and functional evolution of cannabinoid oxidocyclases remain unknown.

To address this gap, we combined ancestral sequence reconstruction and heterologous expression to resurrect and characterise three ancestral cannabinoid oxidocyclases. Ancestor *HCa* was defined as the most recent common ancestor (MRCA) of clade H and the cannabis‐specific clade; *Ca* as the MRCA of the cannabis‐specific clade, and *A1A2a* as the MRCA of *THCAS* and *CBCAS* (Figure 1b). Characterising these ancestors confirmed that CBGA metabolisation originated in a recent ancestor of the cannabis *Cannabis* lineage and demonstrated that early cannabinoid synthases could produce all three THCA, CBDA and CBCA. We also engineered hybrid enzymes by swapping residues of ancestral and extant enzymes, thus highlighting key mutations underlying the emergence of CBGA metabolisation and subsequent subfunctionalisation toward highly‐specialised THCAS and CBDAS. This work therefore contributes to the understanding of the functional evolution and molecular mechanism of cannabinoid oxidocyclases, which opens new perspectives for biotechnological uses.

## 2 Results

### 2.1 Reconstruction of Three Cannabinoid Oxidocyclase Ancestors

To reconstruct ancestral cannabinoid oxidocyclases, we selected 77 BBL sequences from cannabis, hop and *Trema orientale* (Data S1) and built a Bayesian gene tree (Figure S1), whose resulting topology was consistent with previous BBL classification (van Velzen and Schranz 2021). Internal nodes corresponding to ancestors *A1A2a*, *Ca* and *HCa* were identified and selected for ancestral sequence reconstruction (Figure S1). To ensure accuracy, each ancestral sequence was reconstructed four times, using Bayesian inference (nucleotide) and Maximum Likelihood (nucleotide, codon, amino acid models, Table S1, Data S2). The Bayesian‐inferred sequences, which shared over 99% identity with the Maximum Likelihood nucleotide ones, were selected as the most robust sequences. Average posterior probability was 0.96 for *A1A2a*, 0.95 for *Ca* and 0.94 for *HCa*, indicating overall high confidence. Nucleotides associated with the lowest posterior probabilities were carefully analysed and manually curated (Table S1, Data S3). The final *A1A2a* sequence shared ≈97% nucleotide identity with *THCAS* and *CBCAS*. *Ca* shared 93%–95% identity with *THCAS*, *CBCAS* and *CBDAS*. *HCa* shared 81%–82% identity with *THCAS*, *CBCAS* and *CBDAS*, and 88%–91% identity with clade H. Comparison of homologous genome sequences from cannabis and hop (Figure S2) revealed that most cannabinoid synthase genes and closely related BBLs are part of a large syntenic block that is conserved in both species, confirming that they result from local gene duplications.

### 2.2 Resurrection of Ancestral Enzymes Reveals the Origin and Specialisation of Cannabinoid Synthases

**FIGURE 2:**
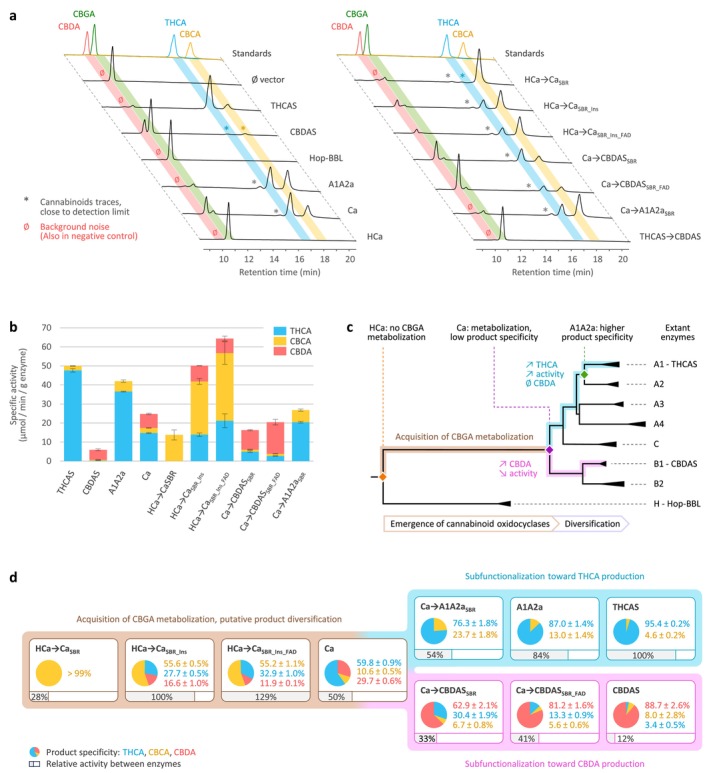
Activity and selectivity of cannabinoid oxidocyclases. (a) Functional screening: High‐performance liquid chromatography (HPLC) separation profiles of extant, ancestral (left), and hybrid enzymes (right), incubated in the presence of CBGA. Cannabinoids were identified by comparison to standard molecules. An empty vector (Ø) was used as a negative control. (b) Specific activity of the enzymes able to metabolise CBGA. All incubations were performed in triplicates; error bars represent the standard deviation. (c) Functional evolution of cannabinoid oxidocyclases. Results from panels (a, b) were placed in the context of the BBL phylogeny. The gene tree was drawn based on Figure S1; the branch lengths reflect the relative number of substitutions per site. (d) Emergence of CBGA metabolisation and partitioning of Ca activity. The relative activities (grey bars) and product specificities (pie charts) of each enzyme were determined based on the results displayed in panel (b). Note that the hybrids are artificial and do not necessarily reflect ‘real’ evolutionary steps. Abbreviated molecules: CBCA, cannabichromenic acid; CBDA, cannabidiolic acid; CBGA, cannabigerolic acid; THCA, (−)‐trans‐Δ9‐tetrahydrocannabinolic acid.

The coding sequences of ancestral *A1A2a*, *Ca*, *HCa* and extant *THCAS*, *CBDAS* and *Hop‐BBL* were domesticated (Data S4), synthesised, and expressed in yeast (*Komagataella phaffii*). Associated enzymes were purified and used for in vitro activity assays. To test whether these enzymes could metabolise CBGA, we first performed qualitative assays. Results showed that the THCAS and A1A2a enzymes could convert CBGA into THCA and CBCA, while the CBDAS and Ca enzymes produced THCA, CBCA and CBDA (Figure 2a). A1A2a and Ca also yielded traces of an unknown product, eluted at ≈16 min. This product was too close to the detection limit to be quantified and will not be further mentioned. To the contrary, Hop‐BBL and HCa did not convert CBGA into any detectable product (Figure 2a) despite being properly expressed in our yeast system (Figure S3). This demonstrates that the ability to metabolise CBGA emerged along the branch leading from HCa to Ca (summarised in Figure 2c).

Next, enzyme characterisation showed that Ca, the oldest active ancestor, could function at pH 4–8 (Figure S4a). Maximal activity was reached at pH 5.5, but pH 4–6 favoured THCA and CBDA production while pH 6–8 favoured CBCA production. Ca was also highly thermostable, with an optimum of 45°C–50°C (Figure S4b). These results are very similar to those of THCAS and CBDAS (Taura et al. 1995; Zirpel et al. 2018), meaning the overall pH and temperature activity profile of cannabinoid oxidocyclases did probably not undergo major evolutionary changes, and that ancestral and extant enzymes could be compared under the same reactional conditions.

We therefore proceeded with quantitative assays to compare active enzymes, using a pH of 5 (i.e., optimal for THCA and CBDA) and a temperature of 30°C (i.e., sub‐optimal but physiologically relevant in cannabis). Results showed that the most active enzyme was THCAS (specific activity: 50.0 ± 0.8 μmol min^−1^ g^−1^), which also exhibited the highest selectivity for THCA production, yielding 95% THCA and 5% CBCA (Figure 2b). A1A2a displayed similar performance, with slightly lower activity (84% relative to THCAS) and selectivity (87% THCA, 13% CBCA). Ca exhibited lower activity (50% relative to THCAS) and broader selectivity, yielding 60% THCA, 30% CBDA and 10% CBCA. Finally, CBDAS displayed the lowest activity (12% relative to THCAS) but the highest selectivity for CBDA (89% CBDA, 8% CBCA, 3% THCA, Figure 2b). These results highlight how gene duplications in ancestral cannabis plants led to the subfunctionalisation of the broad product selectivity of Ca into extant, highly‐specialised THCAS and CBDAS (Figure 2c).

### 2.3 Mutations Underlying the Origin of Cannabinoid Oxidocyclases

**FIGURE 3:**
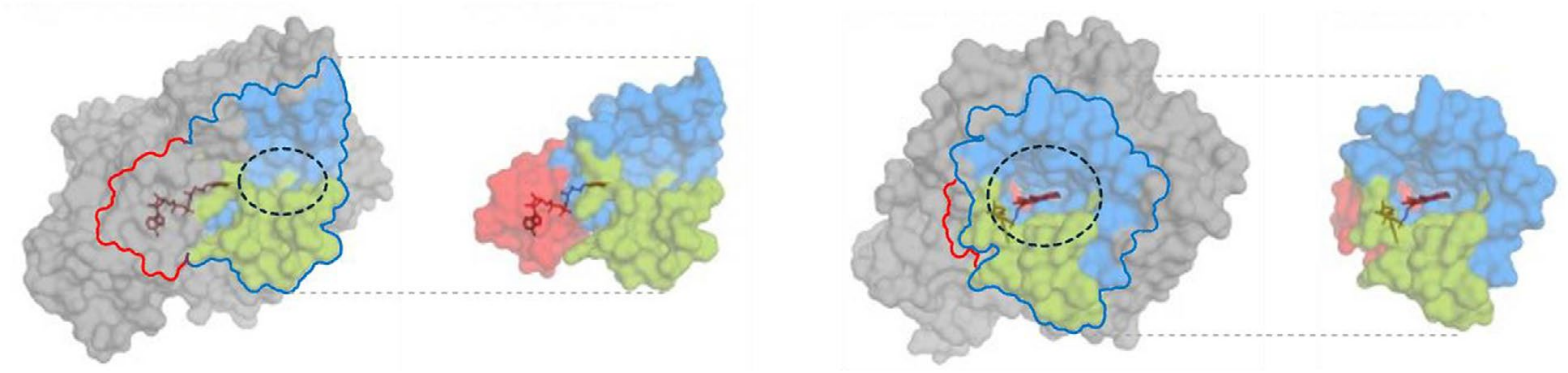
Three‐dimensional structure and main domains of the THCAS enzyme. The enzyme (3vte.1.A) is coloured in grey, with residues from the substrate binding region (SBR) in blue including the ASA‐loop in green, and from the FAD binding site (FBS) in red. The FAD cofactor is dark red. The substrate binding cavity is circled with a black dotted line.

To investigate key mutations underlying functional evolution, we designed hybrid enzymes corresponding to ancestral enzymes (as backbone sequences) in which amino acid residues of interest were substituted with their equivalents from evolutionary more recent oxidocyclases (as donor sequences). To keep consistency despite cannabinoid oxidocyclases sequence length variations, residues were numbered based on their homologous position in THCAS. Residues of interest were selected based on their structural context. For this, we defined two regions of cannabinoid oxidocyclases: the substrate binding region (SBR) and the FAD binding site (FBS, Figure 3). The SBR was defined as a large region comprising all residues that may contribute to shaping the substrate binding cavity and its surroundings. This includes the previously defined active site adjacent‐loop (ASA‐loop, residues 354–380) (Villard et al. 2023) and some residues surrounding the FAD isoalloxazine ring. The FBS was defined as the residues surrounding the FAD cofactor, which were not already part of the SBR (Figure 3).

**FIGURE 4:**
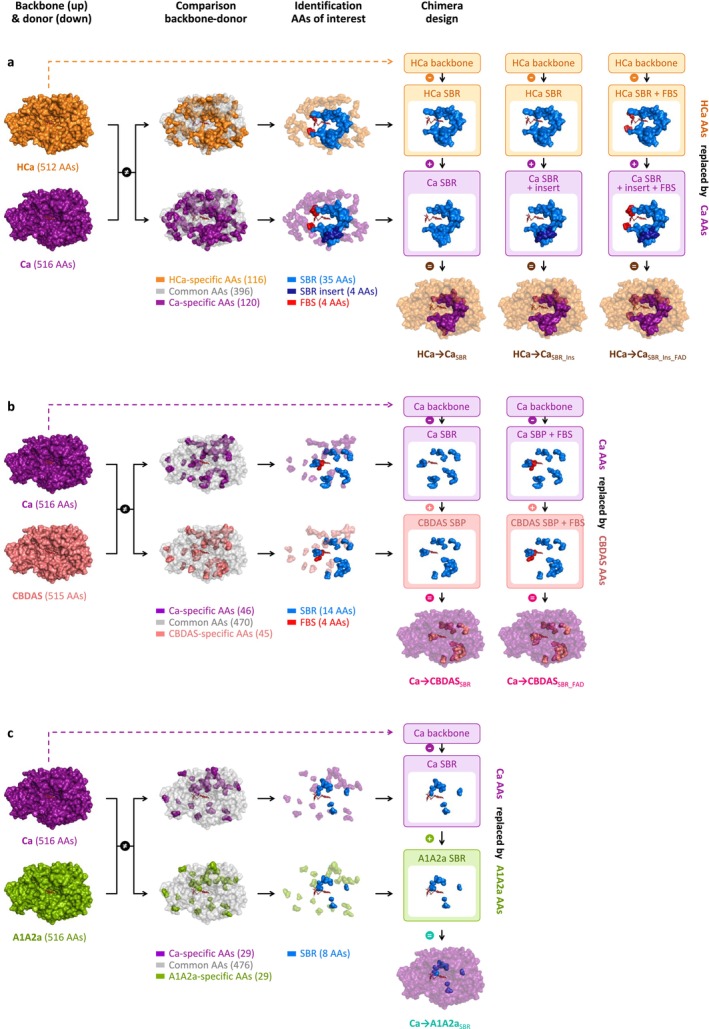
Design of the hybrid enzymes. (a) HCa → Ca, (b) Ca → CBDAS, and (c) Ca → A1A2a hybrid enzymes. The HCa backbone is orange, Ca is purple, CBDAS is salmon, A1A2a is green, and the FAD cofactor is dark red. Based on the backbone‐donor comparison, amino acids of interest were highlighted in blue (substrate binding region), dark blue (four‐residues insertion) and red (FAD binding site). AAs, amino acids; FBS, FAD binding site; SBR, substrate binding region.

To investigate the origin of CBGA metabolisation, we designed hybrids between HCa (backbone) and Ca (donor, Figure 4a). HCa and Ca share 77% amino acid identity, corresponding to 116 amino acid mutations and a four‐residue insertion (Table S2a). These evolutionary changes were highlighted in three‐dimensional enzyme homology models (Figure 4a), showing that 39 mutations and the four‐residue insertion occurred in the SBR, while four mutations occurred in the FBS. The 73 remaining mutations were not located in known regions of interest. We therefore designed three incremental hybrids: HCa → Ca_SBR_ corresponds to HCa with the 39 SBR mutations from Ca; HCa → Ca_SBR_Ins_ additionally includes the four‐residue insertion; and HCa → Ca_SBR_Ins_FAD_ additionally includes the four FBS mutations (Figure 4a, Table 1).

**TABLE 1:** Mutations introduced in the HCa backbone.

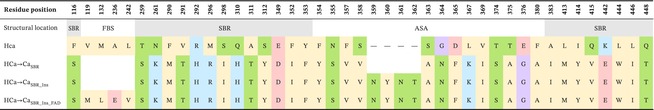

*Note:* Residues are numbered according to their THCAS equivalent. The raw “Structural location” distinguishes residues located in the FAD binding site (FBS, in red) and the substrate binding region (SBR, in blue), including the ASA‐loop (ASA, in green). Amino acids associated to each enzyme are colored according to the physicochemical properties of their side chain: positive amino acids are in blue, negative amino acids in red, polar uncharged amino acids in green, hydrophobic amino acids in yellow, others in purple. In the HCa backbone, “‐” means no amino acid (gap). In the hybrids, empty cells mean no mutation was introduced (*i.e*., same amino acid as in the backbone).

**FIGURE 5:**
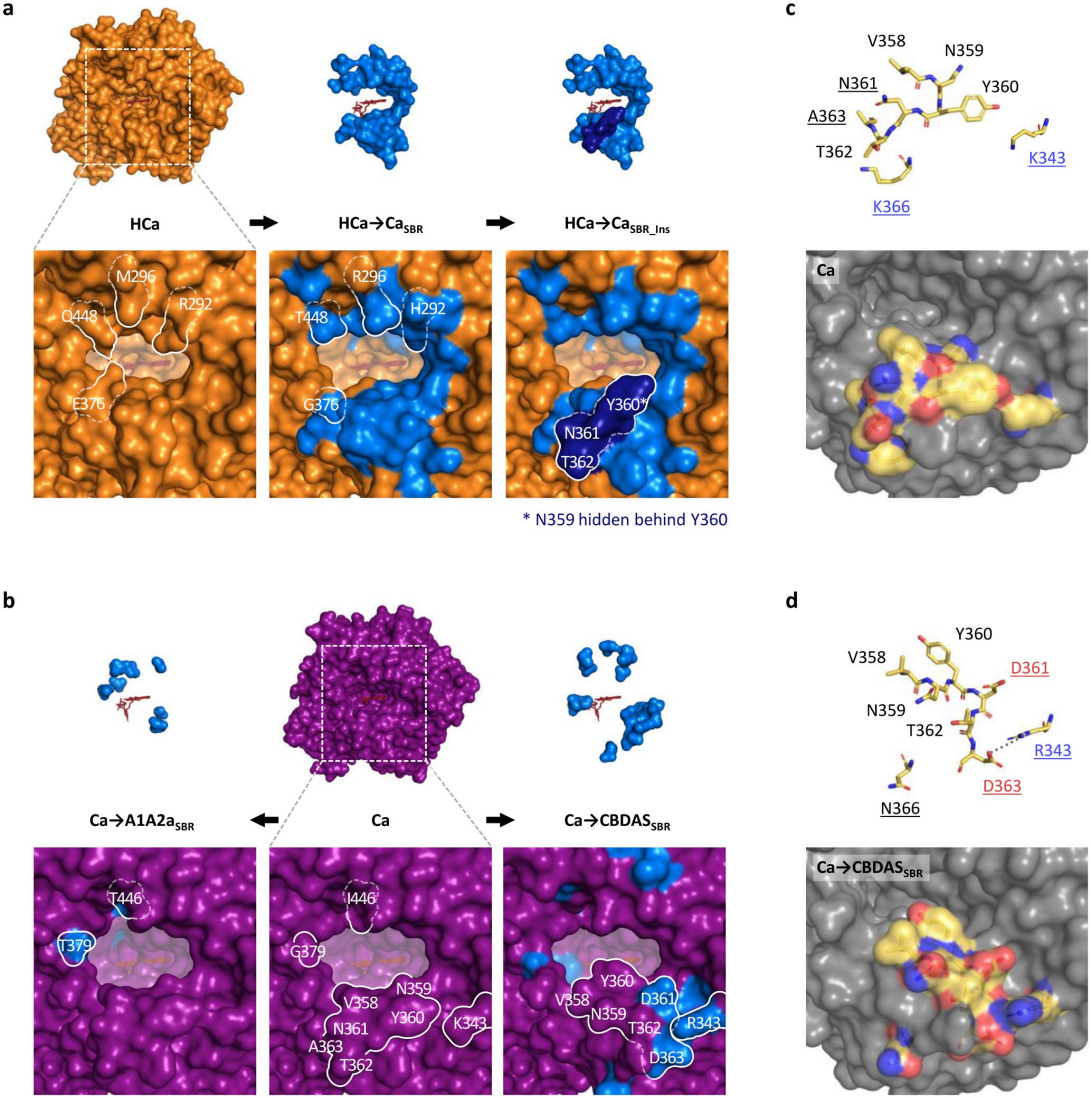
Major modifications in the substrate binding region of ancestral and hybrid cannabinoid oxidocyclases. (a, b) Comparison of the substrate binding region of HCa (a), Ca (b), and associated hybrids. The HCa backbone is orange, the Ca backbone is purple, mutated amino acids are light blue, the four‐amino acid insertion is dark blue, and the FAD cofactor is red. To improve three‐dimensional visualisation, the cavity opening toward the FAD was coloured in transparent white. Mutated residues that strongly impact the shape of the substrate binding region are outlined in white; the line is solid on the surface of the enzyme and dotted where the residues are buried. (c, d) Licorice (up) and surface views (down) of region 358–366 (ASA‐loop) in Ca (c) and Ca → CBDAS_SBR_ (d). Residues 343, 358–362 and 366 are shown in yellow, with red oxygen and blue nitrogen atoms. The surface views show these residues in their context (grey). In the licorice views, names of residues with electrically charged side chains are highlighted in red (negative) and blue (positive). Names of residues mutated in Ca → CBDAS_SBR_ are underlined. The dotted grey line between D363 and R343 in panel d is 4.8 Å long. To improve readability, residues 363–365 were not detailed in the licorice view, as they overlap with others and did not undergo major modifications. Residues are numbered according to their THCAS equivalent.

Subsequent enzyme characterisation showed that all three hybrids could metabolise CBGA (Figure 2a), producing CBCA as the main product (Figure 2b). First, HCa → Ca_SBR_ exhibited low activity (28% relative to THCAS) but an almost perfect selectivity for CBCA (> 99%) at every tested pH (Figure S5a). The 39 mutations introduced in HCa were therefore enough to allow CBGA metabolisation. From a structural perspective, mutations such as E376G and Q448T significantly widened the substrate binding cavity, creating a larger opening toward the FAD (Figure 5a). This suggests that HCa may have metabolised smaller substrate(s) and that cavity opening may have been key to initiating CBGA metabolisation.

Compared to HCa → Ca_SBR_, HCa → Ca_SBR_Ins_ exhibited a 3.6‐fold higher activity (100% relative to THCAS) and a broader selectivity, yielding 56% CBCA, 28% THCA and 16% CBDA (Figure 2d). This indicates that the four‐residues insertion at position 359–362, which is part of the ASA‐loop (Table 1), must have played a critical role in cannabinoid diversification (Figure 5a).

Compared to HCa → Ca_SBR_Ins_, HCa → Ca_SBR_Ins_FAD_ displayed a 1.3‐fold activity increase (129% relative to THCAS) and a 1.6‐fold higher THCA/CBDA ratio (55% CBCA, 33% THCA, 12% CBDA, Figure 2d), highlighting how subtle adjustments around the FAD ribityl tail can impact enzyme activity. Finally, even though HCa → Ca_SBR_Ins_FAD_ and Ca possess similar SBR and FBS, they displayed distinct specific activity (2.6‐fold difference) and main product (CBCA or THCA, Figure 2d). These differences must therefore result from the 77 mutations distant from the active site (Figure 4a).

### 2.4 Mutations Underlying the Specialisation of Cannabinoid Oxidocyclases

To investigate the subfunctionalisation of Ca, we designed hybrids combining Ca (backbone) with CBDAS or A1A2a (donors, Figure 4b,c). Compared with CBDAS, Ca shares 91% amino acid identity (45 mutations, one deletion) including 14 mutations in the SBR and four in the FBS (Figure 4b, Table S2b). We thus designed the hybrids Ca → CBDAS_SBR_, corresponding to Ca with the 14 SBR mutations from CBDAS, and Ca → CBDAS_SBR_FAD_, which additionally includes the four FBS mutations (Figure 4b, Table 2). Enzyme characterisation showed that both hybrids were active (Figure 2a). First, Ca → CBDAS_SBR_ exhibited low activity (33% relative to THCAS) but its main product was CBDA (63% CBDA, 30% THCA, 7% CBCA, Figure 2b). Compared to Ca, the 14 SBR mutations introduced in Ca → CBDAS_SBR_ therefore reversed the THCA/CBDA ratio, at the expense of a 1.5‐fold activity decrease (Figure 2d). Comparing Ca and Ca → CBDAS_SBR_ structures, we observed a rotation of residues 358–366, in the ASA‐loop (Figure 5b–d). This rotation might result from the charge‐altering mutations N361D, A363D, K366N and K343R, which caused residues D361 and D363 (negative) to move toward R343 (positive), carrying with them neighbouring residues (Figure 5c,d). This rotation might alter CBGA binding and favour CBDA production. Second, compared to Ca → CBDAS_SBR_, the Ca → CBDAS_SBR_FAD_ hybrid displayed a 1.2‐fold higher activity (41% relative to THCA) and a 1.3‐fold higher selectivity for CBDA (81% CBDA, 13% THCA, 6% CBCA, Figure 2d, Figure S5b). Again, this highlights the importance of residues around the FAD ribityl tail. Remarkably, Ca → CBDAS_SBR_FAD_ activity was 3.4‐fold higher than that of extant CBDAS enzyme, resulting in a 3.1‐fold higher CBDA production despite slightly lower selectivity (Figure 2b,d).

**TABLE 2:** Mutations introduced in the Ca backbone.

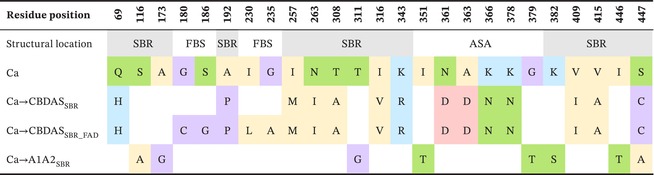

*Note:* Residues are numbered according to their THCAS equivalent. The raw “Structural location” distinguishes residues located in the FAD binding site (FBS, in red) and the substrate binding region (SBR, in blue), including the ASA‐loop (ASA, in green). Amino acids associated to each enzyme are colored according to the physicochemical properties of their side chain: positive amino acids are in blue, negative amino acids in red, polar uncharged amino acids in green, hydrophobic amino acids in yellow, others in purple. In the hybrids, empty cells mean no mutation was introduced (*i.e*., same amino acid as in the backbone).

Compared with A1A2a, Ca shares 94% identity (29 mutations) including eight mutations in the SBR and two in the FBS (Figure 4c, Table S2c). This led to the Ca → A1A2a_SBR_ hybrid, corresponding to Ca with the eight SBR mutations from A1A2a (Figure 4c, Table 2). Because of Ca and A1A2a's high identity, FBS mutations were ignored. Ca → A1A2a_SBR_ was about as active as Ca (54% relative to THCAS) but it did not produce CBDA, resulting in higher proportions of THCA (76%) and CBCA (24%, Figure 2b,d). The eight associated mutations were therefore enough to prevent CBDA production, without impacting overall metabolisation. Structurally, mutations G379T and I446T slightly alter the shape of the substrate binding cavity (Figure 5b) and are therefore the most likely mutations to prevent CBDA production.

Overall, the specialisation toward THCA production correlated with a significant activity increase, while specialisation toward CBDA correlated with lowered activity (Figure 2b–d). This suggests mutations favouring CBDA and/or preventing THCA production may negatively impact catalysis, and/or that selection favoured cannabis plants with less active CBDAS.

### 2.5 Ancestral Enzymes Provide Relevant Backbones for Engineering

Building on previous results, we tried to engineer extant THCAS to make it produce CBDA. Indeed, since we successfully prevented CBDA production in Ca → A1A2a_SBR_ and increased it in Ca → CBDAS_SBR_FAD_, we wondered if associated mutations could be leveraged in the highly active THCAS. We therefore designed the THCAS → CBDAS hybrid, corresponding to THCAS in which we reversed the eight Ca → A1A2a_SBR_ mutations preventing CBDA production and introduced the 18 Ca → CBDAS_SBR_FAD_ mutations favouring CBDA production (Figure S6). Unfortunately, enzyme characterisation showed that THCAS → CBDAS was not able to metabolise CBGA (Figure 2a). This suggests that THCAS overall structure is now so specialised toward THCA production that modifying its active site is not enough to restore CBDA production. Hence, ancestral enzymes like Ca could be used as more flexible entry points to engineer cannabinoid oxidocyclases than their highly‐specialised, extant counterparts.

Finally, even though our goal was not to improve enzyme heterologous expression, we noticed that ancestral enzymes were more highly expressed than extant ones. Indeed, compared to THCAS, expression levels were about 2 times lower for CBDAS, 3–4 times higher for A1A2a, 1.5–3 times higher for Ca, and 2–3 times higher for HCa. Expression levels of the hybrids were similar to that of their ancestral backbone, with moderate influence from their donor (Table S3). It may thus be possible to use A1A2a, Ca and HCa to engineer cannabinoid oxidocyclases that are easier to produce for biotechnological applications.

## 3 Discussion

Cannabinoid oxidocyclases are key enzymes that regioselectively convert CBGA into cannabinoids with different bioactivities; therefore modulating cannabis therapeutic potential. Through resurrecting and characterising three ancestral cannabinoid oxidocyclases, we experimentally tested the hypothesis (Vergara et al. 2019; van Velzen and Schranz 2021) that CBGA metabolisation emerged in a recent ancestor of cannabis. Our Bayesian gene tree (Figure S1) suggests that HCa was present prior to the divergence of cannabis and hop estimated at 25–27 million years ago (McPartland 2018; Jin et al. 2020), and that along the branch from HCa to Ca there was a loss in an ancestor of hop. In the absence of sequence data from an orthologous locus in the *Humulus* lineage, it is therefore currently impossible to determine whether cannabinoid biosynthesis originated within the *Cannabis* lineage or in a common ancestor of both *Cannabis* and *Humulus*. In any case, our results confirm that the acquisition of cannabinoid oxidocyclase activity arose independently in the Cannabaceae and other phylogenetically distant, cannabinoid‐producing taxa such as *Rhododendron* (Ericaceae) and *Helichrysum* (Asteraceae) (Taura et al. 2014; Pollastro et al. 2017; Berman et al. 2023).

Through characterising the ancestral Ca enzyme, we also demonstrated that early cannabinoid oxidocyclases were not CBDAS, as proposed earlier (Onofri et al. 2015), but rather promiscuous enzymes yielding THCA as the main product. However, the ‘first’ cannabinoid oxidocyclase was likely not Ca, but an ancestor along the HCa–Ca branch with a putatively different activity. Based on the characterisation of the HCa → Ca hybrids, we hypothesize that early cannabinoid oxidocyclases that did not yet possess the 359–362 insertion could convert CBGA into only CBCA. As data from extant plants are insufficient to determine the chronological order of mutations between HCa and Ca, it is not possible to test this hypothesis. Nevertheless, CBCA is produced at neutral pH and could therefore have been a physiologically relevant product before cannabinoid oxidocyclases were excreted into acidic trichomes (Sirikantaramas et al. 2005). Additionally, CBCA is produced by all characterised cannabinoid oxidocyclases (Sirikantaramas et al. 2004; Taura et al. 2007; Laverty et al. 2019). Finally, a BBL enzyme from *Rhododendron dauricum* produces daurichromenic acid (Taura et al. 2014) which is a CBCA farnesyl analog, and certain bacterial BBL enzymes produce CBC (Love et al. 2024), suggesting that BBL enzymes might be predisposed toward CBC(A) cyclisation. These various aspects suggest that CBCA might have been the ‘original’ product of cannabinoid oxidocyclases, with product diversification following the 359–362 insertion. Subsequent gene duplication and sequence evolution facilitated subfunctionalisation toward highly‐specialised THCAS and CBDAS.

To identify mutations underlying functional evolution, we designed hybrids by swapping residues between ancestral and extant enzymes. Across the various hybrid enzymes, 62 residues were investigated. Among them, 15 had previously been studied and showed slight to major impacts on THCAS and/or CBDAS (Shoyama et al. 2012; Zirpel et al. 2018; Villard et al. 2023; Dai et al. 2024) (summarised in Table S4). For instance, the residues mutated in HCa → Ca_SBR_ included three known critical residues: residue H292, which is the most likely counterion to CBGA carboxylate (Shoyama et al. 2012); A116, which helps position the catalytic base (Zirpel et al. 2018); and S355, which ancestral state in HCa (N) inactivates THCAS (Garfinkel et al. 2021; Villard et al. 2023). Therefore, even though the acquisition of CBGA metabolisation likely required concerted evolution of many more residues in HCa → Ca_SBR_, mutation of these three residues was probably essential. As far as we know, the remaining 47 residues investigated in the various hybrids were never tested before and could be valuable targets for site‐directed mutagenesis experiments, to further elucidate the cannabinoid oxidocyclase molecular mechanism.

Our results also showed that the ASA‐loop—a flexible looped region that has so far received very little attention (Villard et al. 2023)—has undergone major, recent evolutionary changes. Indeed, out of the 26 residues comprising the ASA‐loop (residues 354–380), 21 evolved from HCa to extant enzymes. These include critical modifications such as the abovementioned insertion of residues 359–362 from HCa to Ca, the loop rotation caused by mutations of residues 361 and 363 from Ca to CBDAS, and the mutation of residue 379 from Ca to THCAS, which likely contributed to CBDA production loss. These suggest that the recent evolution of the ASA‐loop was critical for the evolution of cannabinoid oxidocyclase substrate and product selectivity. Contrarily, residues known to be crucial for general BBL activity (e.g., catalytic base, covalent FAD‐binding) (Shoyama et al. 2012; Zirpel et al. 2018) were strictly conserved from HCa to extant enzymes, suggesting that HCa and Hop‐BBL are functional, even though their activities remain unknown.

Nowadays, medicinal cannabinoids are predominantly produced via cannabis cultivation (Adhikary et al. 2021). Biotechnological heterologous production represents a promising alternative, with potentially better scalability, but it is hampered by the low activity and expression levels of CBDAS and CBCAS (Carvalho et al. 2017; Luo et al. 2019; Schmidt et al. 2024). Interestingly, our reconstructed ancestors proved to be relatively easy to express and engineer, making them ideal backbones for designing suitable enzymes for biotechnological applications. For example, Ca → CBDAS_SBR_FAD_ showed 3.1‐fold higher CBDA production and 2–3 times higher expression than wildtype CBDAS. Similarly, HCa → Ca_SBR_ exhibited almost perfect CBCA selectivity and 3–4 times higher expression than wildtype THCAS. These enzymes could thus be used to help lift the classic bottleneck of cannabinoid production in microorganisms. As CBCA is typically present in low concentrations in most cannabis cultivars (Wishart et al. 2024), HCa → Ca_SBR_ could also be transformed into cannabis plants to generate novel CBCA‐dominant cultivars.

## 4 Experimental Procedures

### 4.1 Material

Standard solutions of cannabinoids 1.0 mg mL^−1^ in acetonitrile were purchased from Sigma Aldrich.

### 4.2 Phylogenetic Tree Inference and Gene Microsynteny Assessment

A BBL dataset was initially based on 248 nucleotide sequences from *C. sativa* , *H. lupulus* and *T. orientale* (Natsume et al. 2015; Van Velzen et al. 2018; Laverty et al. 2019; Vergara et al. 2019; McKernan et al. 2020; Gao et al. 2020; Grassa et al. 2021), collected from a previous compilation (van Velzen and Schranz 2021) (Data S5). This dataset was reduced to 77 sequences by excluding redundant sequences, sequences with ambiguous nucleotides (e.g., potential chimeras), and sequences bringing irrelevant diversity for ancestral reconstruction (e.g., sequences with an almost perfect identity with others except for one or a few unique, evolutionary recent mutations). The remaining 77 sequences (Data S1) were representative of every subclade from the cannabis‐specific clade and their closest relatives in *H. lupulus* and *T. orientale* , and the outgroup was selected based on the phylogeny of Eurosid BBL genes (van Velzen and Schranz 2021). To allow subsequent analyses, frameshift‐containing pseudogenes were altered by the addition of ‘N’ at the site of the indels (Data S1). BBL nucleotide sequences were then aligned with GENEIOUS (Geneious prime 2019), relying on a Translation Alignment based on MAFFT v.7.450. The alignment was manually curated (Data S1), partitioned (CodonPos1 CodonPos2, CodonPos3) in MESQUITE v.3.6, and used to build a Bayesian gene tree on MRBAYES (Ronquist and Huelsenbeck 2003; Ronquist et al. 2012) v.3.2.7 as described elsewhere (Villard et al. 2021). The tree was drawn with FIGTREE v.1.4.4 and used to identify clades and ancestors of interest.

To assess microsynteny of the selected BBL genes between cannabis and hop, we compared homologous sequences from hop chromosome 9 (accession NC_084801.1) and cannabis cultivar Jamaican Lion mother accessions JAATIP010000026.1 and JAATIP010000103.1 in Geneious prime v.2025.2.2. Jamaican Lion mother was selected because it comprises a large total number (26) of cannabinoid synthase genes (van Velzen and Schranz 2021).

### 4.3 Ancestral Sequence Reconstruction

Each ancestral sequence to reconstruct was inferred based on the curated alignment with both Bayesian statistics and Maximum Likelihood approaches. Bayesian inference was performed with MrBayes (Ronquist and Huelsenbeck 2003; Ronquist et al. 2012) v.3.2.7, using the following parameters: nst = mixed, rates = gamma, shape = (all), 10 million generations, temperature heating 0.05, partitioning: CodonPos1 CodonPos2, CodonPos3 (Data S6). Maximum Likelihood reconstruction was performed on PAML (Yang 1997) v.4.9, using an unrooted version of the BBL gene tree. Three models were tested, based on nucleotide, codon and amino acid analyses. Parameters of the best‐fit models were determined through likelihood ratio tests and were as follows: Nucleotide: baseml, model = 7, Mgene = 4, alpha = 1, ncatG = 5. Codon: codeml, model = 0, alpha = 0.5, ncatG = 20, CodonFreq = 1, aaDist = 1, NSsites = 0. Amino acids: codeml, model = 3, alpha = 0.5, ncatG = 10, aaRatefile = jones (Data S7). For each ancestor to reconstruct, the sequences obtained with the four Bayesian and maximum likelihood methods (Data S2) were manually processed. First, as ancestral inference places a nucleotide at each position of the alignment, even if it mostly consists of gaps, the position of ancestral gaps was manually predicted by carefully inspecting the multiple sequence alignment, and associated nucleotides or amino acids were deleted (Data S2). Specifically, most ancestral gaps originated from insertions in individual pseudogenes or outgroup sequences and could therefore be removed. There were two notable exceptions: (1) the four‐amino acid insertion, present in every sequence of the cannabis specific clade and absent elsewhere, and (2) a one‐amino acid deletion found exclusively in all sequences of clade B1. Next, the sequences obtained with the different models were compared, and the sequences inferred with Bayesian statistics were selected as the most accurate ones (Table S1). To further validate the quality of the Bayesian sequences, posterior probabilities were analysed: for each position of the alignment, nucleotides with a posterior probability above 0.6 were kept. When the nucleotide with the highest posterior probability was below 0.6, the position was identified as ambiguous and alternative nucleotides were considered. For this, the results obtained with the Bayesian and maximum likelihood models were compared, and the potential impact of alternative nucleotides on the enzyme was investigated (e.g., silent or missense mutation, amino acid properties, residue location, phylogenetic patterns, functional relevance), as described in Table S1. When ambiguity could not be disregarded (e.g., not a silent mutation), we selected the combination of ambiguous character states that most effectively allowed testing the ancestor's functional hypothesis, by prioritising those facilitating the alternative hypothesis (e.g., in HCa, we selected the character states most likely to facilitate CBGA metabolisation, Table S1). This led to the replacement of two, one and one ambiguous codon(s) of the Bayesian HCa, Ca and A1A2a sequences, respectively, by their second‐best alternative. Final ancestral sequences are available in Data S3.

### 4.4 Expression and Purification of BBL Enzymes

BBL enzymes were produced in yeast and purified following a previously described, THCAS‐optimised protocol (Villard et al. 2023). Briefly, His‐tagged coding sequences of the *THCAS* (GenBank accession AB057805), *CBDAS* (GenBank accession NM_001397936.1), *Hop‐BBL* (HopBase accession 000840F.g23.t1), and associated ancestors and mutants were domesticated (Data S4), synthesised by GenScript (Leiden, the Netherlands) and subcloned into the pPICZαA expression vector (Invitrogen, Thermo Fisher Scientific). Recombinant plasmids were transformed into the *K. phaffii* strain X‐33 (Invitrogen, Thermo Fisher Scientific). Heterologous expression was achieved by culturing the recombinant *K. phaffii* in methanol‐containing induction medium for 2 days. Resulting His‐tagged enzymes were harvested, purified by affinity and exchanged in assay buffer (sodium citrate, 100 mM, pH 5.0) (Villard et al. 2023). Purified enzymes were kept on ice and used immediately for activity assay. The presence of His‐tagged protein in the purified solution was verified by Western blot, using 6x‐His‐tag Monoclonal Antibody coupled with alkaline phosphatase (Thermo Fisher Scientific). Protein concentrations were quantified by Bradford tests, using bovine gamma globulin as protein standard.

### 4.5 Enzyme In Vitro Assays

To determine which enzymes could metabolise CBGA, qualitative assays were performed using reaction conditions (e.g., buffer, pH, temperature) previously described for extant cannabinoid oxidocyclases (Sirikantaramas et al. 2004; Taura et al. 2007; Zirpel et al. 2018). For these assays, 80–800 μg mL^−1^ of freshly produced enzymes (depending on expression levels) were incubated in 80 μL of sodium citrate buffer (100 mM, pH 5.0) containing 100 μM of CBGA. After 60 min at 45°C, 700 rpm, reactions were stopped by the addition of 0.25 volume of acetonitrile 100%. Enzymes that did not metabolise CBGA under these conditions were also tested with variable pH (3–8) and temperatures (25°C–60°C).

To determine the optimal reactional pH of Ca, HCa → Ca_SBR_ and Ca → CBDAS_SBR___FAD_, 83 μg mL^−1^ (Ca), 40 μg mL^−1^ (HCa → Ca_SBR_) or 74 μg mL^−1^ (Ca → CBDAS_SBR___FAD_) of freshly produced enzymes were incubated in 80 μL of sodium citrate (100 mM, pH 3, 4, 4.5, 5 and 6) or potassium phosphate (100 mM, pH 7 and 8) containing 75 μM of CBGA. After 55 min at 30°C, 700 rpm, reactions were stopped by the addition of 0.25 volume of 100% acetonitrile.

To determine the optimal reactional temperature of Ca, 40 μg mL^−1^ of freshly produced enzyme was incubated in 80 μL of sodium citrate (100 mM, pH 5) containing 75 μM of CBGA. After 30 min at 30°C–60°C, 700 rpm, reactions were stopped by the addition of 0.25 volume of 100% acetonitrile.

Due to the low solubility of cannabinoids in aqueous buffer (e.g., ≈150–200 μM for CBGA) (Zirpel et al. 2015), determining kinetic parameters of cannabinoid oxidocyclases is challenging. For instance, reported *K*_m_ values for THCAS range from *K*_m_ = 134 μM (Taura et al. 1995) to 540 μM (Sirikantaramas et al. 2004). Hence, performing standardised assays and determining specific activities are common alternatives to compare the performance of cannabinoid oxidocyclases (Shoyama et al. 2012; Zirpel et al. 2018; Villard et al. 2023). We therefore decided to perform standardised assays using 75 μM of CBGA, which ensures good solubility and remains well below the estimated *K*_m_ of the THCAS (i.e., below substrate inhibition). Standardised *assays were performed* by incubating 30 μg mL^−1^ of fresh enzymes in 80 μL of sodium citrate buffer (100 mM, pH 5.0) containing 75 μM of CBGA. After 30 min at 30°C, 700 rpm, reactions were stopped by the addition of 0.25 volume 100% acetonitrile. All reactions were performed in triplicates.

### 4.6 Identification and Quantification of Cannabinoids

Reaction mixtures were filtered at 0.2 μm and analysed by high‐performance liquid chromatography (HPLC) using previously described equipment, solvents and parameters (Villard et al. 2023). The mobile gradient phase was modified as follows (A/B; v/v): 60:40 between 0 and 1 min, 25:75 at 4 min, 10:90 at 19 min, 0:100 at 20 min and 60:40 between 25 and 30 min. Compounds were detected based on UV scans at 270, 254, 220 and 305 nm. Reaction products were identified by comparison of their retention time with those of standard molecules.

### 4.7 Homology Modelling and Design of Hybrid Enzymes

Three‐dimensional (3D) homology models of the wildtype, ancestral and hybrid BBLs were generated on the basis of the THCAS crystal structure (Shoyama et al. 2012) (PDB ID: 3VTE), using the SWISS‐MODEL protein homology modelling server (https://swissmodel.expasy.org/) (Guex et al. 2009; Bienert et al. 2017; Bertoni et al. 2017; Waterhouse et al. 2018; Studer et al. 2020). The BBL N‐terminal signal peptide was not included in the modelling. Model quality was assessed using the GMQE and QMEAN scoring functions (Table S5). Ramachandran plots and local quality estimates were also checked. The 3D homology models were visualised, analysed and compared using PyMOL v.2.5.4 (Schrödinger LLC) to investigate residue spatial location (i.e., close to the active site, to the cofactor, or on the surface of the enzyme) and design hybrids (Tables S1 and S2). Figures were prepared using PyMOL v.2.5.4.

## 5 Accession Numbers

All sequences used to build the phylogenetic gene tree and reconstruct ancestors are available in GenBank (https://www.ncbi.nlm.nih.gov/) and HopBase (https://hopbase.cgrb.oregonstate.edu/index.html) data libraries.

## Author Contributions

C.V. and R.V. designed the experiments. C.V. and I.B. conducted the in vitro experiments, with the support of A.C.P. and K.C. C.V. performed the in silico analyses, with the support of R.V. C.V. analysed the data. C.V., M.E.S. and R.V. wrote the article. M.E.S. and R.V. directed the project.

## Conflicts of Interest

C.V., R.V. and M.E.S. are listed as inventors on a pending patent application in the name of Wageningen University. The remaining authors declare no conflicts of interest.

## Supporting information


**Figure S1:** Phylogeny of Cannabaceae‐specific Berberine Bridge‐Like genes.
**Figure S2:** Syntenic blocks comprising cannabinoid synthase genes and closely‐related BBLs.
**Figure S3:** Evaluation of enzyme expression by immunodetection.
**Figure S4:** Determination of the optimal pH and reactional temperature for the activity of Ca.
**Figure S5:** Determination of the optimal pH for the activity of HCa → Ca_SBR_ and Ca → CBDAS_SBR_FAD_.
**Figure S6:** Design and structure of the THCAS → CBDAS hybrid.
**Table S1:** Analysis of the reconstructed ancestral sequences.
**Table S2:** Design of the HCa → Ca (a), Ca → CBDAS (b) and Ca → A1A2a (c) hybrids, based on sequence and structural comparison.
**Table S3:** Expression level of candidate enzymes (μg mL^−1^).
**Table S4:** Comparison of the mutations tested in previous studies with mutations included in our hybrids.
**Table S5:** Quality assessment of the three‐dimensional (3D) enzyme homology models.
**Data S1:** Sequence alignment used to generate the gene‐tree and reconstruct the ancestors.
**Data S2:** Ancestral sequences reconstructed with MrBayes and PAML.
**Data S3:** Sequences of A1A1a, Ca and HCa.
**Data S4:** Domesticated sequences used to express and characterise enzymes.
**Data S5:** Berberine Bridge‐Like dataset.
**Data S6:** Ancestral sequence reconstruction with MrBayes.
**Data S7:** Ancestral sequence reconstruction with PAML.

## Data Availability

The data that supports the findings of this study are available in the Supporting Information of this article.

## References

[pbi70475-bib-0001] Aderinto, N. , G. Olatunji , E. Kokori , et al. 2024. “The Efficacy and Safety of Cannabidiol (CBD) in Pediatric Patients With Dravet Syndrome: A Narrative Review of Clinical Trials.” European Journal of Medical Research 29: 182. 38500226 10.1186/s40001-024-01788-6 PMC10949818

[pbi70475-bib-0002] Adhikary, D. , M. Kulkarni , A. El‐Mezawy , et al. 2021. “Medical Cannabis and Industrial Hemp Tissue Culture: Present Status and Future Potential.” Frontiers in Plant Science 12: 627240. 33747008 10.3389/fpls.2021.627240 PMC7968383

[pbi70475-bib-0003] Berman, P. , L. A. De Haro , A. Jozwiak , et al. 2023. “Parallel Evolution of Cannabinoid Biosynthesis.” Nature Plants 9: 817–831. 37127748 10.1038/s41477-023-01402-3

[pbi70475-bib-0004] Bertoni, M. , F. Kiefer , M. Biasini , L. Bordoli , and T. Schwede . 2017. “Modeling Protein Quaternary Structure of Homo‐ and Hetero‐Oligomers Beyond Binary Interactions by Homology.” Scientific Reports 7: 10480. 28874689 10.1038/s41598-017-09654-8 PMC5585393

[pbi70475-bib-0005] Bienert, S. , A. Waterhouse , T. A. P. de Beer , et al. 2017. “The SWISS‐MODEL Repository—New Features and Functionality.” Nucleic Acids Research 45: D313–D319. 27899672 10.1093/nar/gkw1132 PMC5210589

[pbi70475-bib-0006] Cammà, G. , M. P. Verdouw , P. B. Van Der Meer , L. Groenink , and A. Batalla . 2025. “Therapeutic Potential of Minor Cannabinoids in Psychiatric Disorders: A Systematic Review.” European Neuropsychopharmacology 91: 9–24. 39541799 10.1016/j.euroneuro.2024.10.006

[pbi70475-bib-0007] Carvalho, Â. , E. H. Hansen , O. Kayser , S. Carlsen , and F. Stehle . 2017. “Designing Microorganisms for Heterologous Biosynthesis of Cannabinoids.” FEMS Yeast Research 17: fox037. 28582498 10.1093/femsyr/fox037 PMC5812543

[pbi70475-bib-0008] Costa, B. 2007. “On the Pharmacological Properties of Δ9‐Tetrahydrocannabinol (THC).” Chemistry & Biodiversity 4: 1664–1677. 17712813 10.1002/cbdv.200790146

[pbi70475-bib-0009] Dai, L. , T. Niu , R. Luo , et al. 2024. “Improvement of Cannabidiolic Acid Synthetase Activity Through Molecular Docking and Site‐Directed Mutagenesis.” Industrial Crops and Products 208: 117860.

[pbi70475-bib-0010] Daniel, B. , B. Konrad , M. Toplak , et al. 2017. “The Family of Berberine Bridge Enzyme‐Like Enzymes: A Treasure‐Trove of Oxidative Reactions.” Archives of Biochemistry and Biophysics 632: 88–103. 28676375 10.1016/j.abb.2017.06.023

[pbi70475-bib-0011] Fellermeier, M. , and M. H. Zenk . 1998. “Prenylation of Olivetolate by a Hemp Transferase Yields Cannabigerolic Acid, the Precursor of Tetrahydrocannabinol.” FEBS Letters 427: 283–285. 9607329 10.1016/s0014-5793(98)00450-5

[pbi70475-bib-0012] Gagne, S. J. , J. M. Stout , E. Liu , Z. Boubakir , S. M. Clark , and J. E. Page . 2012. “Identification of Olivetolic Acid Cyclase From *Cannabis sativa* Reveals a Unique Catalytic Route to Plant Polyketides.” Proceedings of the National Academy of Sciences 109: 12811–12816. 10.1073/pnas.1200330109 PMC3411943 22802619

[pbi70475-bib-0013] Gao, S. , B. Wang , S. Xie , et al. 2020. “A High‐Quality Reference Genome of Wild *Cannabis sativa* .” Horticulture Research 7: 73. 32377363 10.1038/s41438-020-0295-3 PMC7195422

[pbi70475-bib-0014] Garfinkel, A. R. , M. Otten , and S. Crawford . 2021. “SNP in Potentially Defunct Tetrahydrocannabinolic Acid Synthase Is a Marker for Cannabigerolic Acid Dominance in *Cannabis sativa* L.” Genes 12: 228. 33557333 10.3390/genes12020228 PMC7916091

[pbi70475-bib-0015] Grassa, C. J. , G. D. Weiblen , J. P. Wenger , et al. 2021. “A New *Cannabis* Genome Assembly Associates Elevated Cannabidiol (CBD) With Hemp Introgressed Into Marijuana.” New Phytologist 230: 1665–1679. 33521943 10.1111/nph.17243 PMC8248131

[pbi70475-bib-0016] Guex, N. , M. C. Peitsch , and T. Schwede . 2009. “Automated Comparative Protein Structure Modeling With SWISS‐MODEL and Swiss‐PdbViewer: A Historical Perspective.” Electrophoresis 30: S162–S173. 19517507 10.1002/elps.200900140

[pbi70475-bib-0017] Gülck, T. , and B. L. Møller . 2020. “Phytocannabinoids: Origins and Biosynthesis.” Trends in Plant Science 25: 985–1004. 32646718 10.1016/j.tplants.2020.05.005

[pbi70475-bib-0018] Han, K. , J.‐Y. Wang , P.‐Y. Wang , and Y.‐C.‐H. Peng . 2024. “Therapeutic Potential of Cannabidiol (CBD) in Anxiety Disorders: A Systematic Review and Meta‐Analysis.” Psychiatry Research 339: 116049. 38924898 10.1016/j.psychres.2024.116049

[pbi70475-bib-0019] Jeddi, H. M. , J. W. Busse , B. Sadeghirad , et al. 2024. “Cannabis for Medical Use Versus Opioids for Chronic Non‐Cancer Pain: A Systematic Review and Network Meta‐Analysis of Randomised Clinical Trials.” BMJ Open 14: e068182. 10.1136/bmjopen-2022-068182 PMC10773353 38171632

[pbi70475-bib-0020] Jin, J. , M. Yang , P. W. Fritsch , R. Van Velzen , D. Li , and T. Yi . 2020. “Born Migrators: Historical Biogeography of the Cosmopolitan Family Cannabaceae.” Journal of Systematics and Evolution 58: 461–473.

[pbi70475-bib-0021] Laverty, K. U. , J. M. Stout , M. J. Sullivan , et al. 2019. “A Physical and Genetic Map of *Cannabis sativa* Identifies Extensive Rearrangements at the *THC/CBD Acid Synthase* Loci.” Genome Research 29: 146–156. 30409771 10.1101/gr.242594.118 PMC6314170

[pbi70475-bib-0022] Love, A. C. , T. N. Purdy , F. M. Hubert , E. J. Kirwan , D. C. Holland , and B. S. Moore . 2024. “Discovery of Latent Cannabichromene Cyclase Activity in Marine Bacterial Flavoenzymes.” ACS Synthetic Biology 13: 1343–1354. 38459634 10.1021/acssynbio.4c00051 PMC11031283

[pbi70475-bib-0023] Luo, X. , M. A. Reiter , L. d'Espaux , et al. 2019. “Complete Biosynthesis of Cannabinoids and Their Unnatural Analogues in Yeast.” Nature 567: 123–126. 30814733 10.1038/s41586-019-0978-9

[pbi70475-bib-0024] McKernan, K. J. , Y. Helbert , L. T. Kane , et al. 2020. “Sequence and Annotation of 42 Cannabis Genomes Reveals Extensive Copy Number Variation in Cannabinoid Synthesis and Pathogen Resistance Genes.” *BioRxiv* 894428.

[pbi70475-bib-0025] McPartland, J. M. 2018. “ *Cannabis* Systematics at the Levels of Family, Genus, and Species.” Cannabis and Cannabinoid Research 3: 203–212. 30426073 10.1089/can.2018.0039 PMC6225593

[pbi70475-bib-0026] Morimoto, S. , K. Komatsu , F. Taura , and Y. Shoyama . 1998. “Purification and Characterization of Cannabichromenic Acid Synthase From *Cannabis sativa* .” Phytochemistry 49: 1525–1529. 9862135 10.1016/s0031-9422(98)00278-7

[pbi70475-bib-0027] Natsume, S. , H. Takagi , A. Shiraishi , et al. 2015. “The Draft Genome of Hop ( *Humulus lupulus* ), an Essence for Brewing.” Plant and Cell Physiology 56: 428–441. 25416290 10.1093/pcp/pcu169

[pbi70475-bib-0028] Onofri, C. , E. P. M. de Meijer , and G. Mandolino . 2015. “Sequence Heterogeneity of Cannabidiolic‐ and Tetrahydrocannabinolic Acid‐Synthase in *Cannabis sativa* L. and Its Relationship With Chemical Phenotype.” Phytochemistry 116: 57–68. 25865737 10.1016/j.phytochem.2015.03.006

[pbi70475-bib-0029] Pollastro, F. , L. De Petrocellis , A. Schiano‐Moriello , et al. 2017. “Amorfrutin‐Type Phytocannabinoids From *Helichrysum umbraculigerum* .” Fitoterapia 123: 13–17. 28941742 10.1016/j.fitote.2017.09.010

[pbi70475-bib-0030] Ronquist, F. , and J. P. Huelsenbeck . 2003. “MrBayes 3: Bayesian Phylogenetic Inference Under Mixed Models.” Bioinformatics 19: 1572–1574. 12912839 10.1093/bioinformatics/btg180

[pbi70475-bib-0031] Ronquist, F. , M. Teslenko , P. van der Mark , et al. 2012. “MrBayes 3.2: Efficient Bayesian Phylogenetic Inference and Model Choice Across a Large Model Space.” Systematic Biology 61: 539–542. 22357727 10.1093/sysbio/sys029 PMC3329765

[pbi70475-bib-0032] Schmidt, C. , M. Aras , and O. Kayser . 2024. “Engineering Cannabinoid Production in *Saccharomyces cerevisiae* .” Biotechnology Journal 19: 2300507. 10.1002/biot.202300507 38403455

[pbi70475-bib-0033] Shoyama, Y. , T. Tamada , K. Kurihara , et al. 2012. “Structure and Function of ∆1‐Tetrahydrocannabinolic Acid (THCA) Synthase, the Enzyme Controlling the Psychoactivity of *Cannabis sativa* .” Journal of Molecular Biology 423: 96–105. 22766313 10.1016/j.jmb.2012.06.030

[pbi70475-bib-0034] Sirikantaramas, S. , S. Morimoto , Y. Shoyama , et al. 2004. “The Gene Controlling Marijuana Psychoactivity.” Journal of Biological Chemistry 279: 39767–39774. 15190053 10.1074/jbc.M403693200

[pbi70475-bib-0035] Sirikantaramas, S. , F. Taura , Y. Tanaka , Y. Ishikawa , S. Morimoto , and Y. Shoyama . 2005. “Tetrahydrocannabinolic Acid Synthase, the Enzyme Controlling Marijuana Psychoactivity, Is Secreted Into the Storage Cavity of the Glandular Trichomes.” Plant and Cell Physiology 46: 1578–1582. 16024552 10.1093/pcp/pci166

[pbi70475-bib-0036] Stone, N. L. , A. J. Murphy , T. J. England , and S. E. O'Sullivan . 2020. “A Systematic Review of Minor Phytocannabinoids With Promising Neuroprotective Potential.” British Journal of Pharmacology 177: 4330–4352. 32608035 10.1111/bph.15185 PMC7484504

[pbi70475-bib-0037] Stout, J. M. , Z. Boubakir , S. J. Ambrose , R. W. Purves , and J. E. Page . 2012. “The Hexanoyl‐CoA Precursor for Cannabinoid Biosynthesis Is Formed by an Acyl‐Activating Enzyme in *Cannabis sativa* Trichomes: A Cytoplasmic Acyl‐Activating Enzyme Involved in Cannabinoid Biosynthesis.” Plant Journal 71: 353–365. 10.1111/j.1365-313X.2012.04949.x 22353623

[pbi70475-bib-0038] Studer, G. , C. Rempfer , A. M. Waterhouse , R. Gumienny , J. Haas , and T. Schwede . 2020. “QMEANDisCo—Distance Constraints Applied on Model Quality Estimation.” Bioinformatics 36: 1765–1771. 31697312 10.1093/bioinformatics/btz828 PMC7075525

[pbi70475-bib-0039] Taura, F. , M. Iijima , J.‐B. Lee , T. Hashimoto , Y. Asakawa , and F. Kurosaki . 2014. “Daurichromenic Acid‐Producing Oxidocyclase in the Young Leaves of *Rhododendron dauricum* .” Natural Product Communications 9: 1934578X1400900928. 25918805

[pbi70475-bib-0040] Taura, F. , S. Morimoto , and Y. Shoyama . 1996. “Purification and Characterization of Cannabidiolic‐Acid Synthase From *Cannabis sativa* L.” Journal of Biological Chemistry 271: 17411–17416. 8663284 10.1074/jbc.271.29.17411

[pbi70475-bib-0041] Taura, F. , S. Morimoto , Y. Shoyama , and R. Mechoulam . 1995. “First Direct Evidence for the Mechanism of D1‐Tetrahydrocannabinolic Acid Biosynthesis.” Journal of the American Chemical Society 117: 9766–9767.

[pbi70475-bib-0042] Taura, F. , S. Sirikantaramas , Y. Shoyama , K. Yoshikai , Y. Shoyama , and S. Morimoto . 2007. “Cannabidiolic‐Acid Synthase, the Chemotype‐Determining Enzyme in the Fiber‐Type *Cannabis sativa* .” FEBS Letters 581: 2929–2934. 17544411 10.1016/j.febslet.2007.05.043

[pbi70475-bib-0043] Taura, F. , S. Tanaka , C. Taguchi , et al. 2009. “Characterization of Olivetol Synthase, a Polyketide Synthase Putatively Involved in Cannabinoid Biosynthetic Pathway.” FEBS Letters 583: 2061–2066. 19454282 10.1016/j.febslet.2009.05.024

[pbi70475-bib-0044] Van Velzen, R. , R. Holmer , F. Bu , et al. 2018. “Comparative Genomics of the Nonlegume *Parasponia* Reveals Insights Into Evolution of Nitrogen‐Fixing Rhizobium Symbioses.” Proceedings of the National Academy of Sciences 115: E4700–E4709. 10.1073/pnas.1721395115 PMC5960304 29717040

[pbi70475-bib-0045] van Velzen, R. , and M. E. Schranz . 2021. “Origin and Evolution of the Cannabinoid Oxidocyclase Gene Family.” Genome Biology and Evolution 13: evab130. 34100927 10.1093/gbe/evab130 PMC8521752

[pbi70475-bib-0046] Vergara, D. , E. L. Huscher , K. G. Keepers , et al. 2019. “Gene Copy Number Is Associated With Phytochemistry in *Cannabis sativa* .” AoB Plants 11: plz074. 32010439 10.1093/aobpla/plz074 PMC6986684

[pbi70475-bib-0047] Villard, C. , C. Bayer , N. P. Medici , et al. 2023. “Natural Gene Variation in *Cannabis sativa* Unveils a Key Region of Cannabinoid Synthase Enzymes.”

[pbi70475-bib-0048] Villard, C. , R. Munakata , S. Kitajima , et al. 2021. “A New P450 Involved in the Furanocoumarin Pathway Underlies a Recent Case of Convergent Evolution.” New Phytologist 231: 1923–1939. 33978969 10.1111/nph.17458

[pbi70475-bib-0049] Waterhouse, A. , M. Bertoni , S. Bienert , et al. 2018. “SWISS‐MODEL: Homology Modelling of Protein Structures and Complexes.” Nucleic Acids Research 46: W296–W303. 29788355 10.1093/nar/gky427 PMC6030848

[pbi70475-bib-0050] Wishart, D. S. , M. Hiebert‐Giesbrecht , G. Inchehborouni , et al. 2024. “Chemical Composition of Commercial Cannabis.” Journal of Agricultural and Food Chemistry 72: 14099–14113. 38181219 10.1021/acs.jafc.3c06616 PMC11212042

[pbi70475-bib-0051] Yang, Z. 1997. “PAML: A Program Package for Phylogenetic Analysis by Maximum Likelihood.” Bioinformatics 13: 555–556. 10.1093/bioinformatics/13.5.555 9367129

[pbi70475-bib-0052] Zirpel, B. , O. Kayser , and F. Stehle . 2018. “Elucidation of Structure‐Function Relationship of THCA and CBDA Synthase From *Cannabis sativa* L.” Journal of Biotechnology 284: 17–26. 30053500 10.1016/j.jbiotec.2018.07.031

[pbi70475-bib-0053] Zirpel, B. , F. Stehle , and O. Kayser . 2015. “Production of Δ9‐Tetrahydrocannabinolic Acid From Cannabigerolic Acid by Whole Cells of Pichia (Komagataella) Pastoris Expressing Δ9‐Tetrahydrocannabinolic Acid Synthase From *Cannabis sativa* l.” Biotechnology Letters 37: 1869–1875. 25994576 10.1007/s10529-015-1853-x

