# Supplementary material for: Resurrected Ancestral Cannabis Enzymes Unveil the Origin and Functional Evolution of Cannabinoid Synthases
Source: Plant Biotechnol J. 2025 Dec 26;24(4):2685–97. doi: 10.1111/pbi.70475 (PMC13140220; doi:10.1111/pbi.70475)
Supplement: Supplementary file 1 — Figure S1: Phylogeny of Cannabaceae‐specific Berberine Bridge‐Like genes. Figure S2: Syntenic blocks comprising cannabinoid synthase genes and closely‐related BBLs. Figure S3: Evaluation of enzyme expression by immunodetection. Figure S4: Determination of the optimal pH and reactional temperature for the activity of Ca. Figure S5: Determination of the optimal pH for the activity of HCa → CaSBR and Ca → CBDASSBR_FAD. Figure S6: Design and structure of the THCAS → CBDAS hybrid. Table S1: Analysis of the reconstructed ancestral sequences. Table S2: Design of the HCa → Ca (a), Ca → CBDAS (b) and Ca → A1A2a (c) hybrids, based on sequence and structural comparison. Table S3: Expression level of candidate enzymes (μg mL−1). Table S4: Comparison of the mutations tested in previous studies with mutations included in our hybrids. Table S5: Quality assessment of the three‐dimensional (3D) enzyme homology models. Data S1: Sequence alignment used to generate the gene‐tree and reconstruct the ancestors. Data S2: Ancestral sequences reconstructed with MrBayes and PAML. Data S3: Sequences of A1A1a, Ca and HCa. Data S4: Domesticated sequences used to express and characterise enzymes. Data S5: Berberine Bridge‐Like dataset. Data S6: Ancestral sequence reconstruction with MrBayes. Data S7: Ancestral sequence reconstruction with PAML. [file PBI-24-2685-s001.zip › pbi70475-sup-0001-Supinfo.docx]

**Supporting Information**

**Supporting figures**


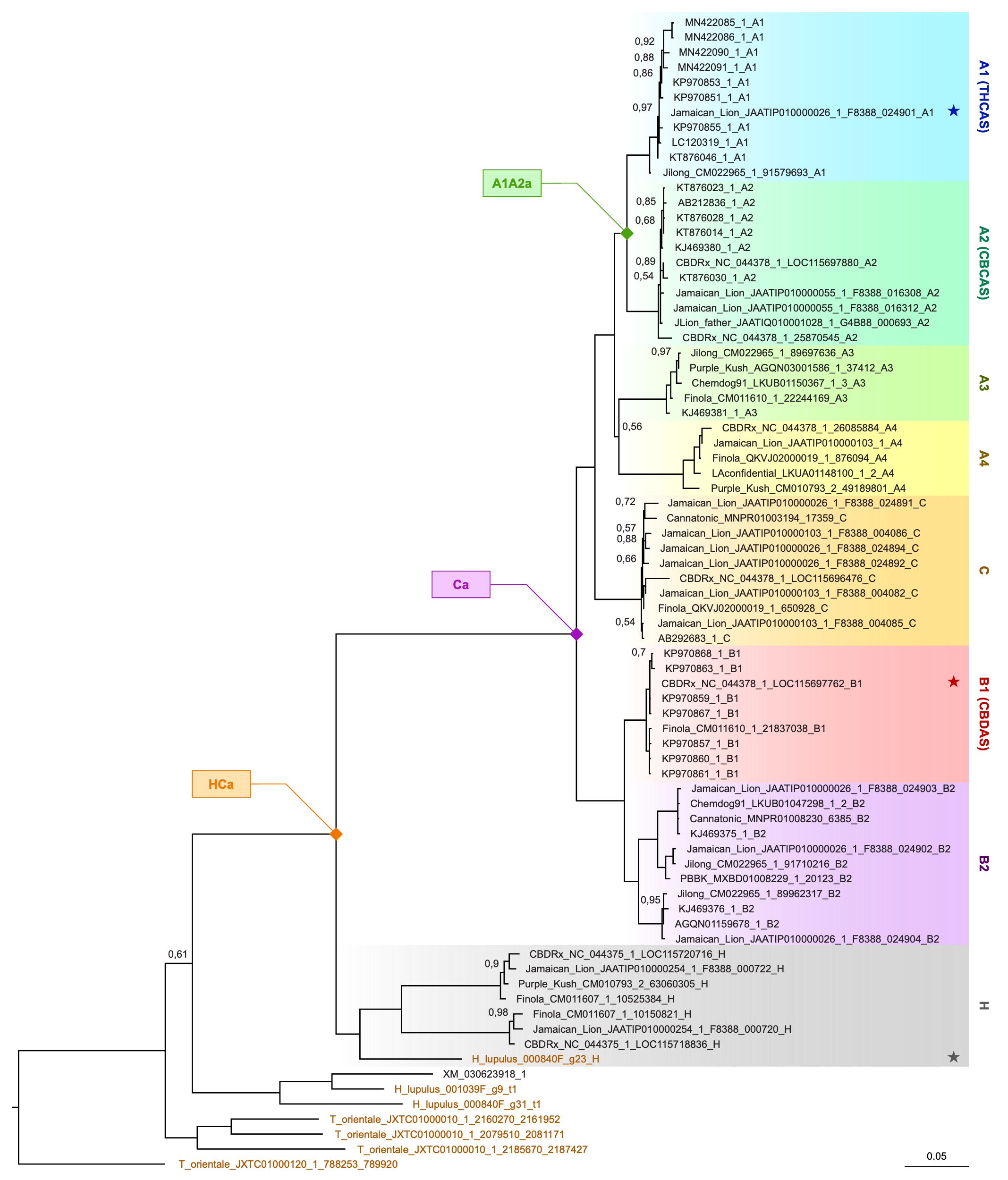


**Figure S1 | Phylogeny of Cannabaceae-specific Berberine Bridge-Like genes.** Bayesian inference gene-family tree based on BBLs from *Cannabis sativa*, *Humulus lupulus* and *Trema orientale*. Colored blocks highlight (sub)clades of interest. Subclades A1-A4, B1-B2 and C form the *Cannabis*-specific clade, as defined previously (van Velzen & Schranz, 2021). Clade H is defined as the sister of the Cannabis-specific clade and includes a non-*Cannabis* sequence: H_lupulus_000840F_g23, hereafter named Hop-BBL. Internal nodes corresponding to A1A2a, Ca and HCa are highlighted. Stars highlight extant enzymes that were functionally characterized in this study: reference THCAS (blue star), reference CBDAS (red star) and Hop-BBL (grey star). C*. sativa* sequences are in black. *H. lupulus* and *T. orientale* sequences are in brown; species names are detailed in labels. Branch labels indicate posterior probabilities below 0.99. Branch lengths indicate the number of substitutions per site.

**Figure S2 | Syntenic blocks comprising cannabinoid synthase genes and closely related BBLs.** Two alternate haplotypes from cannabis cultivar Jamaican Lion mother represent the common genomic context in either drug-type cultivars comprising genes from clades A1 (THCAS), B2 and A3 (haplotype 1) or in hemp-type cultivars comprising clade B1 (CBDAS; haplotype 2). The syntenic block in hop comprises both Hop-BBL and an outgroup (gene 000840F_g31). Triangles indicate genes (not to scale) and their orientation; nonfunctional (pseudo)genes are shown without black outlines. Genes are colored according to their homology and putative orthologs are connected with colored lines (or a block for the tandemly repeated array of clade C). Labels of cannabinoid synthase genes in cannabis and closely related BBLs in hop are in boldface and colored similarly as in Figure S1. The putative orthologs of Hop-BBL in cannabis are translocated to a different chromosome and therefore not shown. Ancestral genes HCa and Ca are highlighted in square blocks and connected to extant genes according to Figure S1.


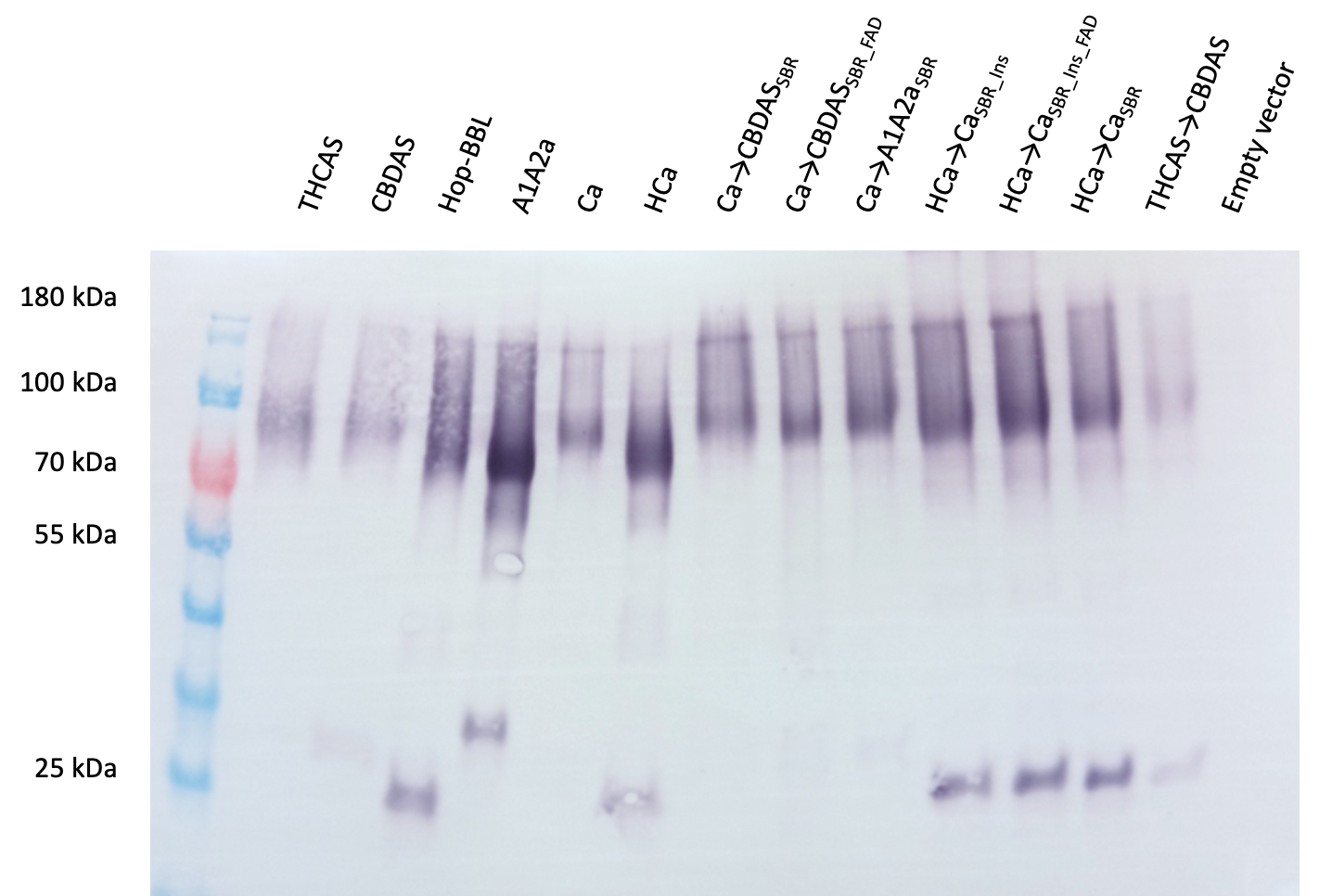
**Figure S3 | Evaluation of enzyme expression by immunodetection.** Western Blot performed on the purified candidate Berberine Bridge-Like (BBL) enzymes, directed against the 6xHis-tag added in the sequence of every candidate. Empty vector refers to the extract obtained from the yeasts transformed with the empty pPICZαA vector (negative control). A molecular weight marker was added in the first and last wells. The theoretical molecular weight of cannabinoid oxidocyclases, calculated based on amino acid composition, is usually of ≈ 60 kDa. However, multiple glycosylation sites are available on these enzymes, classically resulting in higher molecular weights and a smear aspect (Taura *et. al.*, 2007). The bands observed at ≈ 25 kDa are denaturation products appearing upon freezing and thawing enzymes.


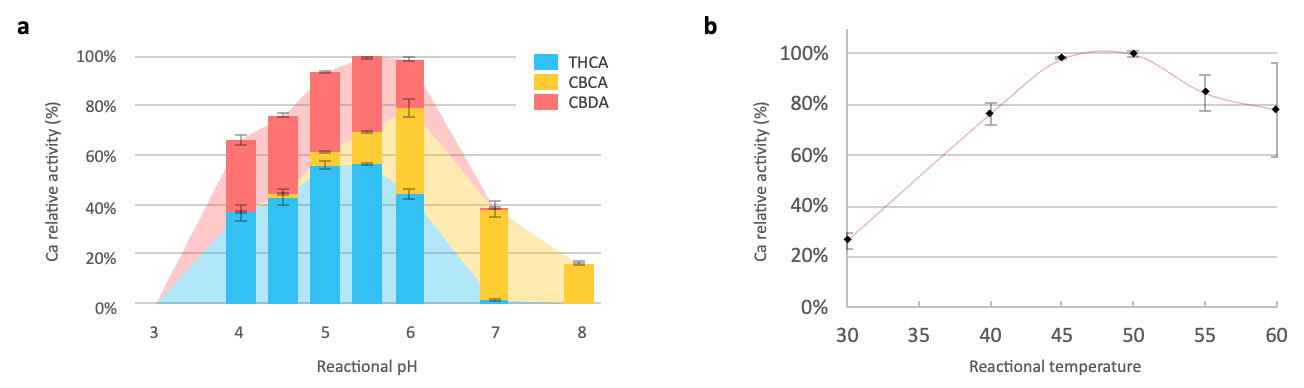
**Figure S4 | Determination of the optimal pH and reactional temperature for the activity of Ca.** **(a)** Optimal pH: Ca was incubated in the presence of CBGA in various reactional pH (30°C). **(b)** Optimal temperature: Ca was incubated in the presence of CBGA at various temperatures (pH 5). Reaction mixes were analyzed by high-performance liquid chromatography (HPLC). All incubations were performed in triplicates; error bars represent the standard deviation. Abbreviated molecules: CBCA, cannabichromenic acid; CBDA, cannabidiolic acid; CBGA, cannabigerolic acid; THCA, (−)-trans-Δ9-tetrahydrocannabinolic acid.


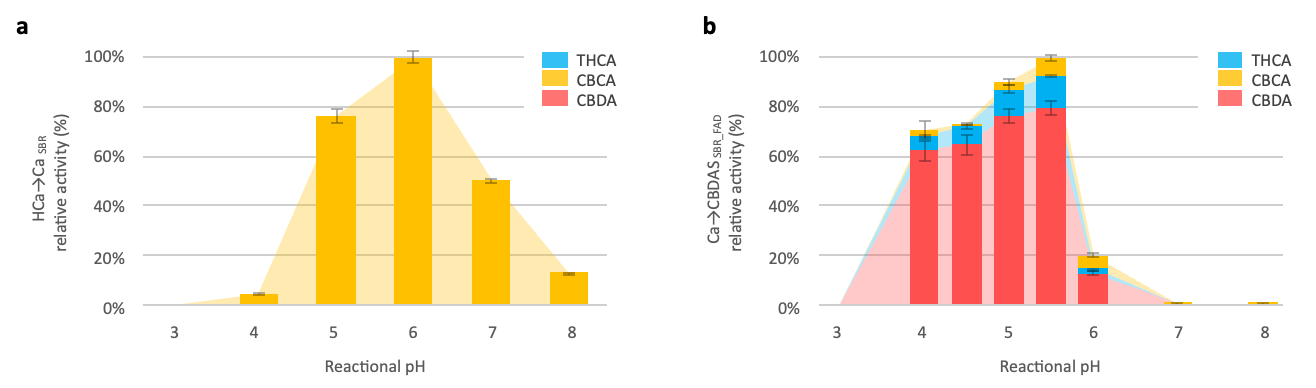
**Figure S5 | Determination of the optimal pH for the activity of HCa→Ca_SBR_ and Ca→CBDAS_SBR_FAD_.** The enzymes HCa→Ca_SBR_ **(a)** and Ca→CBDAS_SBR_FAD_ **(b)** were incubated in the presence of CBGA in various reactional pH (30°C). Reaction mixes were analyzed by high-performance liquid chromatography (HPLC). All incubations were performed in triplicates; error bars represent the standard deviation. Abbreviated molecules: CBCA, cannabichromenic acid; CBDA, cannabidiolic acid; CBGA, cannabigerolic acid; THCA, (−)-trans-Δ9-tetrahydrocannabinolic acid.


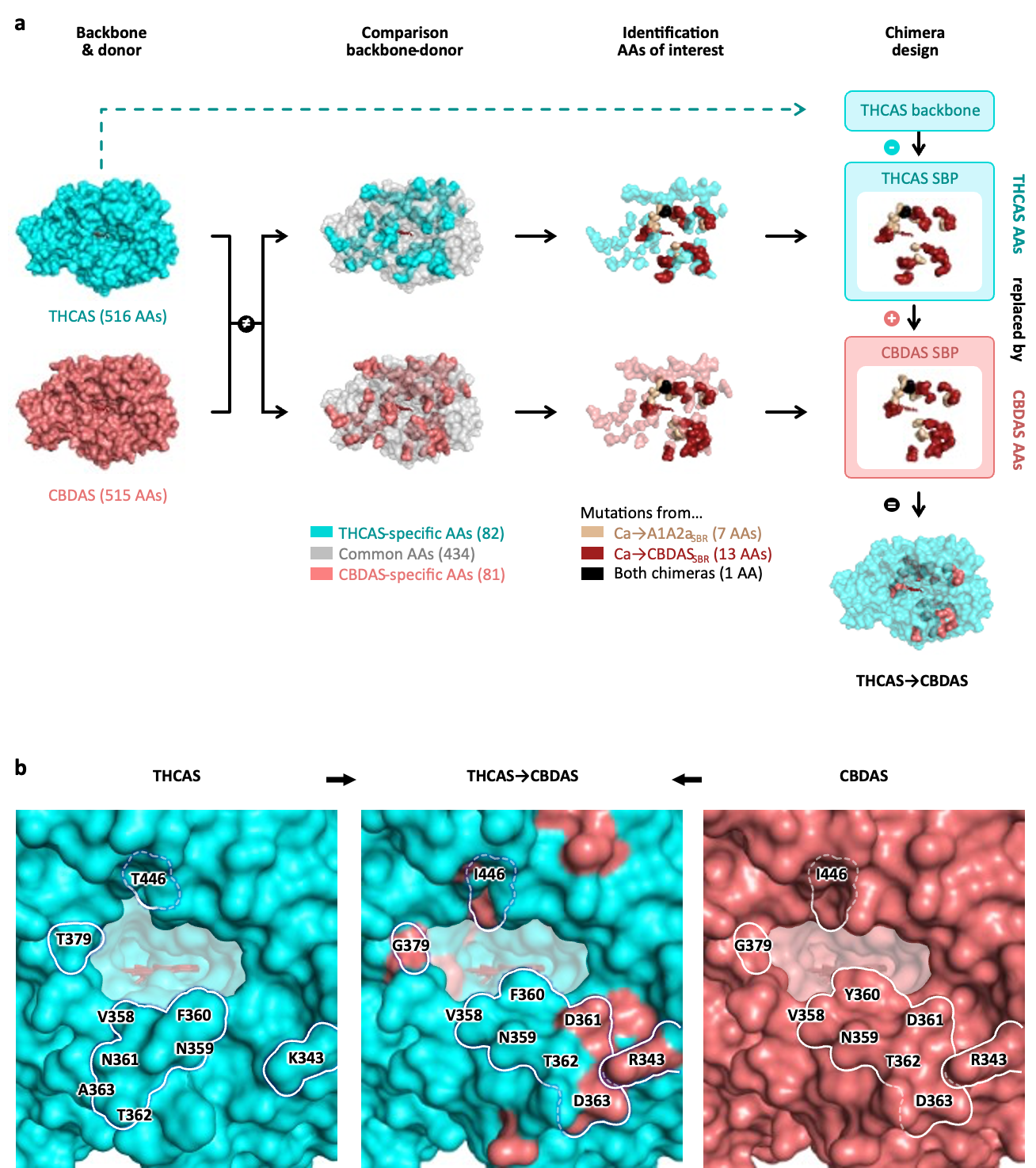
**Figure S6 | Design and structure of the THCAS→CBDAS hybrid.** The THCAS backbone is cyan, the CBDAS donor is salmon. The FAD cofactor is in red. **(a)** Design of THCAS→CBDAS. Mutations from Ca→A1A2a_SBR_ are in gold (reversed to their Ca state), mutations from Ca→CBDAS_SBR_ are in dark red (mutated to their CBDAS state), residues mutated in both Ca→A1A2a_SBR_ and Ca→CBDAS_SBR_ are in black (shown in their CBDAS state). Because of the high similarity in both sequence and activity between A1A2a and THCAS, mutations from A1A2a to THCAS were ignored. Abbreviations: AA, amino acids; SBR, substrate binding region; FBS, FAD binding site. **(b)** Evolution of the substrate binding region in THCAS, THCAS→CBDAS and CBDAS. To improve three-dimensional visualization, the cavity opening toward the FAD was colored in transparent white. Mutated residues that strongly impact the shape of the substrate binding region are outlined in white; the line is solid on the surface of the enzyme and dotted where the residues are buried. The THCAS→CBDAS hybrid successfully recovered the rotation of region 358-366 depicted in **Figure 5b-d**, despite mutation such as Y360F that occurred between A1A2a and THCAS. Residues are numbered according to their THCAS equivalent.

**Supporting tables**

*[Excel file attached]*

**Table S1 |Analysis of the reconstructed ancestral sequences.** **(a)** Comparison of the sequence obtained with the Bayesian and Maximum likelihood models. **(b)** Flowchart explaining how posterior probabilities were analyzed and summarizing which ambiguous characters were replaced. **(c)** Posterior probabilities associated to each nucleotide of the Bayesian inferred sequence. **(d-f)** Analysis of the ambiguous characters in HCa **(d)**, Ca **(e)** and A1A2a **(f)**.

*[Excel file attached]*

**Table S2 |Design of the HCa→Ca (a), Ca→CBDAS (b) and Ca→A1A2a (c) hybrids, based on sequence and structural comparison.** For each set of hybrids, the sequence of the backbone and donor were compared. Residues that were different in the backbone and donor (*i.e.*, evolutionary changes) were identified and detailed; identical residues were ignored. The structural location of the residues was investigated. Residues located in the FAD binding site (FBS, in red) and the substrate binding region (SBR, in blue), including the ASA-loop (ASA, in green) were identified, others were ignored. Physicochemical properties of the residues of interest in the backbone and donor were compared and classified into moderate to major differences (+), minor differences (-), and insertions (ins). These different elements were used to determine which residues to target while designing hybrids. Residues are numbered according to their THCAS equivalent. Residues are colored according to the physicochemical properties of their side chain: positive residues are in blue, negative residues in red, polar uncharged residues in green, hydrophobic residues in yellow, others in purple.

|  | **Batch A** | **Batch B** | **Batch C** | **Batch D** | **Batch E** | **Batch F** | **Batch G** | **Batch H** | **Batch I** | **Batch J** | **Batch K** |
| --- | --- | --- | --- | --- | --- | --- | --- | --- | --- | --- | --- |
| **THCAS** | 298 | 282 |  |  |  |  | 324 | 281 |  | 320 | 326 |
| **CBDAS** |  | 174 |  |  | 191 | 167 |  | 152 |  |  |  |
| **Hop-BBL** |  |  |  |  |  |  | 581 | 749 |  |  |  |
| **A1A2a** | 1315 | 861  789 |  |  | 659 |  |  | 976 |  |  |  |
| **Ca** | 703 | 444  498 |  |  | 382 |  |  | 747 | 460 |  | 520  446 |
| **Hca** | 685 | 564  681 |  |  | 769 |  |  | 985 |  |  |  |
| **HCa→Ca_SBR_** |  |  |  |  |  |  | 711 | 1060 |  | 1262 | 1055 |
| **HCa→Ca_SBR_Ins_** |  |  | 841 | 1051 | 371 | 907 |  | 1091 |  |  |  |
| **HCa→Ca_SBR_Ins_FAD_** |  |  | 756 | 1118 | 918 | 844 |  | 1245 |  |  |  |
| **Canna→CBDAS_SBR_** |  |  | 322 | 299 | 391 |  |  | 431 |  |  |  |
| **Canna→CBDAS_SBR_FAD_** |  |  |  |  |  |  | 382 | 430 | 422 | 450 |  |
| **Ca→A1A2a_SBR_** |  |  | 475 | 473 | 495 |  |  | 657 |  |  |  |
| **THCAS→CBDAS** |  |  |  |  |  |  | 263 | 135 | 167 |  |  |

**Table S3 | Expression level of candidate enzymes (µg.mL^-1^).** Candidate Berberine Bridge-Like (BBL) enzymes were expressed in yeast and purified. Their expression levels were determined after purification with a Bradford test across multiple, independent experimental batches. Displayed values have been standardized to compensate for the initial volumes of yeast culture and the final volumes of purified enzymes, which were sometimes adjusted from batch to batch depending on the experimental need for larger quantities or concentrations of enzymes. Cells containing two values correspond to enzymes that have been independently produced and quantified twice, within the same batch.

*[Excel file attached]*

**Table S4 |Comparison of the mutations tested in previous studies with mutations included in our hybrids.** All residues are numbered according to their THCAS equivalent. The first columns (B-D) present all cannabinoid oxidocyclase mutations that were investigated in previous studies, through site directed mutagenesis or bioinformatic analyses. Tested mutations are colored according to the role of the associated residues: residues involved in substrate binding and/or catalysis are blue, including residues from the ASA-loop in green, residues involved in FAD-binding are red, structurally essential residues are purple, glycosylation sites in yellow. Residues which role was not specified are white. The next columns (E-P) replace previously tested mutation in the context of the present study. Column E specifies the structural location of associated residues, according to our definitions: substrate binding region (SBR, blue), ASA-loop (ASA, green); FAD binding site (FBS, red). Columns F-J indicate associated amino acids in ancestral and extant enzymes. Residues that evolved between HCa and Ca are highlighted in purple, residues that evolved after Ca are highlighted in orange. Finally, columns K-P indicate which of the previously tested residues were also included in our hybrids.

| **Enzyme** | **Template** | **GMQE** | **QMEAN** |
| --- | --- | --- | --- |
| **THCAS** | 3vte.1.A | 0.96 | 0.92 |
| **CBDAS** | 3vte.1.A | 0.92 | 0.88 |
| **Hop-BBL** | 3vte.1.A | 0.88 | 0.85 |
| **Hca** | 3vte.1.A | 0.91 | 0.88 |
| **Ca** | 3vte.1.A | 0.94 | 0.91 |
| **A1A2a** | 3vte.1.A | 0.95 | 0.91 |
| **Hca→Ca_SBR_** | 3vte.1.A | 0.92 | 0.89 |
| **HCa→Ca_SBR_Ins_** | 3vte.1.A | 0.92 | 0.89 |
| **HCa→Ca_SBR_Ins_FAD_** | 3vte.1.A | 0.92 | 0.90 |
| **Ca→CBDAS_SBR_** | 3vte.1.A | 0.94 | 0.90 |
| **Ca→CBDAS_SBR_FAD_** | 3vte.1.A | 0.93 | 0.90 |
| **Ca→A1A2a_SBR_** | 3vte.1.A | 0.94 | 0.91 |
| **THCAS→CBDAS** | 3vte.1.A | 0.94 | 0.91 |

**Table S5 |Quality assessment of the three-dimensional (3D) enzyme homology models**. The Global Model Quality Estimate (GMQE) reflects the expected accuracy of the model. The Qualitative Model Energy ANalysis (QMEAN) is a composite estimator that reflects the global and local absolute quality estimates. Values vary between 0 (not reliable) and 1 (highly reliable).

**Supporting Data**

*[FASTA file attached]*

**Data S1 | Sequence alignment used to generate the gene-tree and reconstruct the ancestors.** The 77 Berberine Bridge-Like (BBL) sequences were retrieved from van Velzen and Schranz (2021) and use the same labels, which indicate GenBank accession of genomic contig and locus tag (when available) or start position. These sequences are representative of every subclade *from C. sativa* cannabinoid oxidocyclases to their closest *T. orientale* relatives. Frameshift-containing pseudogenes were altered by the addition of “N” at the site of the indels. Sequences were aligned using Geneious and manually curated.

*[FASTA file attached]*

**Data S2 | Ancestral sequences reconstructed with MrBayes and PAML.** Sites corresponding to ancestral gaps were automatically filled in during ancestral reconstruction. These sequences are labelled _Raw. The positions of ancestral gaps were subsequently predicted and associated replaced with gaps. These sequences are labelled _Gap.

*[FASTA file attached]*

**Data S3 | Sequences of A1A1a, Ca and HCa.** Final sequences of A1A2a, Ca and HCa, corresponding to the Bayesian-inferred ancestral sequences after correction of a few nucleotides associated with low posterior probabilities, as explained in **Table S1**.

*[FASTA file attached]*

**Data S4 | Domesticated sequences used to express and characterize enzymes.** Domesticated sequences include extant, ancestral and hybrid Berberine Bridge-Like (BBL) sequences. Extant enzymes include a reference *THCAS* (GenBank accession AB057805), a reference *CBDAS* (GenBank accession NM_001397936.1), and *Hop-BBL* (HopBase accession 000840F.g23.t1). Ancestral sequences are described in **Data S3**, associated hybrids in **Table S2**. Domestication involved the removal of the native plant signal peptide, the addition of a 6xHis-tag extension at the 3’ end, the removal of internal *Eco*RI and *Xba*I restriction sites using genetic code redundancy, and the addition of EcoRI and XBaI restriction sites at the 5’ and 3’ ends, respectively.

*[FASTA file attached]*

**Data S5 | Berberine Bridge-Like dataset.** The BBL sequences were retrieved from van Velzen and Schranz (2021) and use the same labels, which indicate GenBank accession of genomic contig and locus tag (when available) or start position.

*[Text file attached]*

**Data S6 | Ancestral sequence reconstruction with MrBayes.** Command bloc used to infer the three ancestors with MrBayes. Depending on the ancestor, the sequences included in "CladeOfInterest" were different; the command block displayed here applies to the reconstruction of A1A2a.

*[Text file attached]*

**Data S7 | Ancestral sequence reconstruction with PAML.** Control files used to reconstruct the three ancestors with PAML. **(a)** Nucleotide reconstruction with Baseml. **(b)** Codon reconstruction with Codeml. **(c)** Amino acid reconstruction with Codeml.
