# Supplementary material for: Resurrected Ancestral Cannabis Enzymes Unveil the Origin and Functional Evolution of Cannabinoid Synthases
Source: Plant Biotechnol J. 2025 Dec 26;24(4):2685–97. doi: 10.1111/pbi.70475 (PMC13140220; doi:10.1111/pbi.70475)
Supplement: Supplementary file 1 — Figure S1: Phylogeny of Cannabaceae‐specific Berberine Bridge‐Like genes. Figure S2: Syntenic blocks comprising cannabinoid synthase genes and closely‐related BBLs. Figure S3: Evaluation of enzyme expression by immunodetection. Figure S4: Determination of the optimal pH and reactional temperature for the activity of Ca. Figure S5: Determination of the optimal pH for the activity of HCa → CaSBR and Ca → CBDASSBR_FAD. Figure S6: Design and structure of the THCAS → CBDAS hybrid. Table S1: Analysis of the reconstructed ancestral sequences. Table S2: Design of the HCa → Ca (a), Ca → CBDAS (b) and Ca → A1A2a (c) hybrids, based on sequence and structural comparison. Table S3: Expression level of candidate enzymes (μg mL−1). Table S4: Comparison of the mutations tested in previous studies with mutations included in our hybrids. Table S5: Quality assessment of the three‐dimensional (3D) enzyme homology models. Data S1: Sequence alignment used to generate the gene‐tree and reconstruct the ancestors. Data S2: Ancestral sequences reconstructed with MrBayes and PAML. Data S3: Sequences of A1A1a, Ca and HCa. Data S4: Domesticated sequences used to express and characterise enzymes. Data S5: Berberine Bridge‐Like dataset. Data S6: Ancestral sequence reconstruction with MrBayes. Data S7: Ancestral sequence reconstruction with PAML. [file PBI-24-2685-s001.zip › pbi70475-sup-0006-DataS2.pdf]

>A1A2_ MrBayes_Raw
ATGAATTACTGCTCAACAGTATTCATACCTTCTTCCTTTTGGTTTGTTTGCAAATACTCTATAATATTATTTT
TCATTTCTTTTCTCTATTCATTCAATATCCAAATTTCAATAGCTAATTCTCCTCAACGTGATCATGAAAACTT
CCTTAAATGCTTCTCGCAATATATTCCCAACAATAACAATGCAGCAAATCCAGCCAAACTCGTATACACTCAA
CACGACCAATTGTATATGTCTGTCCTGAATTCGACAATACAAAATCTTAGATTCACCTCTGATACAACCCCAA
AACCACTCGTTATTGTCACTCCTTCAAATGTCTCCCATATCCAAGCCACTATTCTATGCTCCAAGAAAGTTGG
CTTGCAGATTCGAACTCGAAGCGGTGGCCATGATGCTGAGGGTTTGTCCTACATATCTCAATTAGTCCCATTT
GTTATAGTAGACTTGAGAAACATGCATTCGGTCAAAATAGATGTTCATAGCCAAACTGCGTGGGTTGAAGCCG
GAGCTACCCTTGGAGAAGTTTATTATTGGATCAATGAGAAGAATGAGAATCTTAGTTTTCCTGGTGGGTATTG
CCCTACTGTTGGCGCAGGTGGACACTTTAGTGGAGGAGGCTATGGAGCATTGATGCGAAATTATGGCCTCGCG
GCTGATAATATCATTGATGCACACTTAGTCAATGTTGATGGAAAAGTTCTAGATCGAAAATCCATGGGAGAAG
ATCTGTTTTGGGCTATACGTGGTGGTGGAGGAGAAAACTTTGGAATCATTGCAGCATGGAAAATCAAACTGGT
TGCTGTCCCATCAAAGTCTACTATATTCAGTGTTAAAAAGAACATGGAGATACATGGGCTTGTCAAGTTATTT
AACAAATGGCAAAATATTGCTTACAAGTATGACAAAGATTTAATACTCATGACTCACTTCATAACCAGGAATA
TTACAGATAATCATGGGAAGAATAGCAAGGAAAAGACTACAGTACATGGTTACTTCTCTTCCATTTTTCATGG
TGGAGTGGATAGTCTAGTCGACTTGATGAACAAGAGCTTTCCTGAGTTGGGTATTAAAAAAACTGATTGCAAA
GAATTGAGCTGGATTGATACAACCATCTTCTACAGTGGTGTTGTAAATTACAACACTGCTAATGCTTTTAAAA
AGGAAATTTTGCTTGATAGATCAGCTGGGTTACAGAAGACGGCTTTACCTTTCTCAATTAAGTTAGACTATGT
TAAGAAACCAATTCCAGAAACTGCAATGGTCAAAATTTTGGAAAAATTATATGAAGAAGATGTAGGAGTTGGG
ATGTATGTGTTGTACCCTTACGGTGGTATAATGGATGAGATTTCAGAATCAGCAATTCCATTCCCTCATCGAG
CTGGAATCATGTATGAACTTTGGTACACAGCTACCTGGGATGAGAAGCAAGAAGATATTAATGAAAAAGCAGT
TGAAATTATTAAGCATATAAACTGGGTTCGAAGTGTTTATAATTTCACGACTCCTTATGTGTCCCAAAATCCA
AGAATGGCGTATCTCAATTATAGGGACCTTGATTTAGGAAAAACTAATCCTGAGAGTCCTAATAATTACACAC
AAGCACGTATTTGGGGTGAAAAGTATTTTGGTAAAAACAATTTTAACAGGTTAGTTAAGGTGAAAACCAAAGT
TGATCCCAATAATTTTTTTAGAAACGAACAAAGTATCCCACCTCTTATGTCATTTCCATTGCGGCCGCGTCAT
CATTAATGAAATTAATATGGACCTTGATATGAGCTTTCTTATTATTATACTAGTTTCTGGACACGTAACTCCC
ATTTGGCCATATGTAGGGCCATATACAATAAGTTGAAGTGTTTAATGTAA

>A1A2a_MrBayes_Gap
ATGAAT---TGCTCAACA---TTC---------TCCTTTTGGTTTGTTTGCAAA------ATAATA---
TTTTTC------TTTCTC---TCATTCAATATCCAAATTTCAATAGCTAAT---CCTCAA---------
GAAAACTTCCTTAAATGCTTCTCGCAATATATTCCC------AACAATGCAGCAAATCCA---
AAACTCGTATACACTCAACACGACCAATTGTATATGTCTGTCCTGAATTCGACAATACAAAATCTTAGATTCA
CCTCTGATACAACCCCAAAACCACTCGTTATTGTCACTCCTTCAAATGTCTCCCATATCCAAGCCACTATTCT
ATGCTCCAAGAAAGTTGGCTTGCAGATTCGAACTCGAAGCGGTGGCCATGATGCTGAGGGTTTGTCCTACATA
TCTCAA---
GTCCCATTTGTTATAGTAGACTTGAGAAACATGCATTCGGTCAAAATAGATGTTCATAGCCAAACTGCGTGGG
TTGAAGCCGGAGCTACCCTTGGAGAAGTTTATTATTGGATCAATGAGAAGAATGAGAATCTTAGTTTTCCTGG
TGGGTATTGCCCTACTGTTGGCGCAGGTGGACACTTTAGTGGAGGAGGCTATGGAGCATTGATGCGAAATTAT
GGCCTCGCGGCTGATAATATCATTGATGCACACTTAGTCAATGTTGATGGAAAAGTTCTAGATCGAAAATCCA
TGGGAGAAGATCTGTTTTGGGCTATACGTGGTGGTGGAGGAGAAAACTTTGGAATCATTGCAGCATGGAAAAT
CAAACTGGTTGCTGTCCCATCAAAGTCTACTATATTCAGTGTTAAAAAGAACATGGAGATACATGGGCTTGTC
AAGTTATTTAACAAATGGCAAAATATTGCTTACAAGTATGACAAAGATTTAATACTCATGACTCACTTCATAA
CCAGGAATATTACAGATAATCATGGGAAGAAT---------
AAGACTACAGTACATGGTTACTTCTCTTCCATTTTTCATGGTGGAGTGGATAGTCTAGTCGACTTGATGAACA
AGAGCTTTCCTGAGTTGGGTATTAAAAAAACTGATTGCAAAGAATTGAGCTGGATTGATACAACCATCTTCTA
CAGTGGTGTTGTAAATTACAACACTGCTAAT---TTTAAAAAGGAAATTTTGCTTGATAGATCAGCTGGG---
CAGAAGACGGCT------
TTCTCAATTAAGTTAGACTATGTTAAGAAACCAATTCCAGAAACTGCAATGGTCAAAATTTTGGAAAAATTAT
ATGAAGAAGATGTAGGAGTTGGGATGTATGTGTTGTACCCTTACGGTGGTATAATGGATGAGATTTCAGAATC
AGCAATTCCATTCCCTCATCGAGCTGGAATCATGTATGAACTTTGGTACACAGCTACCTGG---
GAGAAGCAAGAAGAT---AATGAA------------------
AAGCATATAAACTGGGTTCGAAGTGTTTATAATTTCACGACTCCTTATGTGTCCCAAAATCCAAGAATGGCGT
ATCTCAATTATAGGGACCTTGATTTAGGAAAAACTAATCCTGAGAGTCCTAATAATTACACACAAGCACGTAT
TTGGGGTGAAAAGTATTTTGGTAAA---
AATTTTAACAGGTTAGTTAAGGTGAAAACCAAAGTTGATCCCAATAATTTTTTTAGAAACGAACAAAGTATCC
CACCTCTT---------CCA------CCGCGTCATCATTAA

>A1A2a_PAML_Nucleotide_Raw
ATGAATTACTGCTCAACAGTATTCATACCTTCTTCCTTTTGGTTTGTTTGCAAATACTCTATAATATTATTTT
TCATTTCTTTTCTCTATTCATTCAATATCCAAATTTCAATAGCTAATTCTCCTCAACGTGATCATGAAAACTT
CCTTAAATGCTTCTCGCAATATATTCCCAACAATAACAATGCAGCAAATCCAGCCAAACTCGTATACACTCAA
CACGACCAATTGTATATGTCTGTCCTGAATTCGACAATACAAAATCTTAGATTCACCTCTGATACAACCCCAA
AACCACTCGTTATTGTCACTCCTTCAAATGTCTCCCATATCCAAGCCACTATTCTATGCTCCAAGAAAGTTGG
CTTGCAGATTCGAACTCGAAGCGGTGGCCATGATGCTGAGGGTTTGTCCTACATATCTCAATTAGTCCCATTT
GTTATAGTAGACTTGAGAAACATGCATTCGATCAAAATAGATGTTCATAGCCAAACTGCGTGGGTTGAAGCCG
GAGCTACCCTTGGAGAAGTTTATTATTGGATCAATGAGAAGAATGAGAATCTTAGTTTTCCTGGTGGGTATTG
CCCTACTGTTGGCGCAGGTGGACACTTTAGTGGAGGAGGCTATGGAGCATTGATGCGAAATTATGGCCTCGCG
GCTGATAATATCATTGATGCACACTTAGTCAATGTTGATGGAAAAGTTCTAGATCGAAAATCCATGGGAGAAG
ATCTGTTTTGGGCTATACGTGGTGGTGGAGGAGAAAACTTTGGAATCATTGCAGCATGGAAAATCAAACTGGT
TGCTGTCCCATCAAAGTCTACTATATTCAGTGTTAAAAAGAACATGGAGATACATGGGCTTGTCAAGTTATTT
AACAAATGGCAAAATATTGCTTACAAGTATGACAAAGATTTATTACTCATGACTCACTTCATAACCAGGAATA
TTACAGATAATCATGGGAAGAATAGCAAGGAAAAGACTACAGTACATGGTTACTTCTCTTCCATTTTTCATGG
TGGAGTGGATAGTCTAGTCGACTTGATGAACAAGAGCTTTCCTGAGTTGGGTATTAAAAAAACTGATTGCAAA
GAATTGAGCTGGATTGATACAACCATCTTCTACAGTGGTGTTGTAAATTACAACACTGCTAATGCTTTTAAAA
AGGAAATTTTGCTTGATAGATCAGCTGGGTTACAGAAGACGGCTTTACCTTTCTCAATTAAGTTAGACTATGT
TAAGAAACCAATTCCAGAAACCGCAATGGTCAAAATTTTGGAAAAATTATATGAAGAAGATGTAGGAGTTGGG
ATGTATGTGTTGTACCCTTACGGTGGTATAATGGATGAGATTTCAGAATCAGCAATTCCATTCCCTCATCGAG
CTGGAATCATGTATGAACTTTGGTACACAGCTACCTGGGATGAGAAGCAAGAAGATATTAATGAAAAAGCAGC
TGAAATTATTAAGCATATAAACTGGGTTCGAAGTGTTTATAATTTCACGACTCCTTATGTGTCCCAAAATCCA
AGAATGGCGTATCTCAATTATAGGGACCTTGATTTAGGAAAAACTAATCCTGAGAGTCCTAATAATTACACAC
AAGCACGTATTTGGGGTGAAAAGTATTTTGGTAAAAACAATTTTAACAGGTTAGTTAAGGTGAAAACCAAAGT
TGATCCCAATAATTTTTTTAGAAACGAACAAAGTATCCCACCTCTTATGTCATTTCCATTGCGGCCGCGTCAT
CATTAATGAAATTAATATGGACCTTGATATGAGCTTTCTTATTATTATACTAGTTTCTGGACACGTAACTCCC
ATTTGGCCATATGTAGGGCCATATACAATAAGTTGAAGTGTTTAATGTAA

>A1A2a_PAML_Nucleotide_Gap
ATGAAT---TGCTCAACA---TTC---------TCCTTTTGGTTTGTTTGCAAA------ATAATA---
TTTTTC------TTTCTC---TCATTCAATATCCAAATTTCAATAGCTAAT---CCTCAA---------
GAAAACTTCCTTAAATGCTTCTCGCAATATATTCCC------AACAATGCAGCAAATCCA---
AAACTCGTATACACTCAACACGACCAATTGTATATGTCTGTCCTGAATTCGACAATACAAAATCTTAGATTCA
CCTCTGATACAACCCCAAAACCACTCGTTATTGTCACTCCTTCAAATGTCTCCCATATCCAAGCCACTATTCT
ATGCTCCAAGAAAGTTGGCTTGCAGATTCGAACTCGAAGCGGTGGCCATGATGCTGAGGGTTTGTCCTACATA
TCTCAA---
GTCCCATTTGTTATAGTAGACTTGAGAAACATGCATTCGATCAAAATAGATGTTCATAGCCAAACTGCGTGGG
TTGAAGCCGGAGCTACCCTTGGAGAAGTTTATTATTGGATCAATGAGAAGAATGAGAATCTTAGTTTTCCTGG
TGGGTATTGCCCTACTGTTGGCGCAGGTGGACACTTTAGTGGAGGAGGCTATGGAGCATTGATGCGAAATTAT
GGCCTCGCGGCTGATAATATCATTGATGCACACTTAGTCAATGTTGATGGAAAAGTTCTAGATCGAAAATCCA
TGGGAGAAGATCTGTTTTGGGCTATACGTGGTGGTGGAGGAGAAAACTTTGGAATCATTGCAGCATGGAAAAT
CAAACTGGTTGCTGTCCCATCAAAGTCTACTATATTCAGTGTTAAAAAGAACATGGAGATACATGGGCTTGTC
AAGTTATTTAACAAATGGCAAAATATTGCTTACAAGTATGACAAAGATTTATTACTCATGACTCACTTCATAA
CCAGGAATATTACAGATAATCATGGGAAGAAT---------
AAGACTACAGTACATGGTTACTTCTCTTCCATTTTTCATGGTGGAGTGGATAGTCTAGTCGACTTGATGAACA
AGAGCTTTCCTGAGTTGGGTATTAAAAAAACTGATTGCAAAGAATTGAGCTGGATTGATACAACCATCTTCTA
CAGTGGTGTTGTAAATTACAACACTGCTAAT---TTTAAAAAGGAAATTTTGCTTGATAGATCAGCTGGG---
CAGAAGACGGCT------
TTCTCAATTAAGTTAGACTATGTTAAGAAACCAATTCCAGAAACCGCAATGGTCAAAATTTTGGAAAAATTAT
ATGAAGAAGATGTAGGAGTTGGGATGTATGTGTTGTACCCTTACGGTGGTATAATGGATGAGATTTCAGAATC
AGCAATTCCATTCCCTCATCGAGCTGGAATCATGTATGAACTTTGGTACACAGCTACCTGG---
GAGAAGCAAGAAGAT---AATGAA------------------
AAGCATATAAACTGGGTTCGAAGTGTTTATAATTTCACGACTCCTTATGTGTCCCAAAATCCAAGAATGGCGT
ATCTCAATTATAGGGACCTTGATTTAGGAAAAACTAATCCTGAGAGTCCTAATAATTACACACAAGCACGTAT
TTGGGGTGAAAAGTATTTTGGTAAA---
AATTTTAACAGGTTAGTTAAGGTGAAAACCAAAGTTGATCCCAATAATTTTTTTAGAAACGAACAAAGTATCC
CACCTCTT---------CCA------CCGCGTCATCATTAA

>A1A2a_PAML_Codon_Raw
ATGAATTACTGCAAAACAGTATTCATACCTTCTTCCTTTTGGTTTGTTTGCAAATACTCTATAATATTATTTT
TCATTTCTTTTCTCTATTCATTCAATATCCAAATTTCAATAGCTAATTCTCCTCAACGTGATCATGAAAACTT
CCTTAAATGCTTCTCGCAATATATTCCCAACAATAACAATGCAGCAAATCCAGCCAAACTCGTATACACTCAA
CACGACCAATTGTATATGTCTGTCCTGAATTCGACAATACAAAATCTTAGATTCACCTCTGATACAACCCCAA
AACCACTCGTTATTGTCACTCCTTCAAATGTCTCCCATATCCAAGCCACTATTCTATGCTCCAAGAAAGTTGG
CTTGAAAATTCGAACTAAAAGCGGTGGCCATGATGCTGAGGGTTTGTCCTACATATCTCAATTAGTCCCATTT
GTTATAGTAGACTTGAGAAACATGCATAAAGTCAAAATAGATGTTCATAGCCAAACTGCGTGGGTTGAAGCCG
GAGCTACCCTTGGAGAAGTTTATTATTGGATCAATGAGAAGAATGAGAATCTTAGTTTTCCTGGTGGGTATTG
CCCTACTGTTGGCGCAGGTGGACACTTTAGTGGAGGAGGCTATGGAGCATTGATGAAAAATTATGGCCTCGCG
GCTGATAATATCATTGATGCACACTTAGTCAATGTTGATGGAAAAGTTCTAGATCGAAAATCCATGGGAGAAG
ATCTGTTTTGGGCTATACGTGGTGGTGGAGGAGAAAACTTTGGAATCATTGCAGCATGGAAAATCAAACTGGT
TGCTGTCCCATCAAAGTCTACTATATTCAGTGTTAAAAAGAACATGGAGATACATGGGCTTGTCAAGAAATTT
AACAAAAAACAAAATATTGCTTACAAGTATGACAAAGATAAATTACTCATGACTCACTTCATAACCAGGAATA
TTACAGATAATCATGGGAAGAATAGCAAGGAAAAGACTACAGTACATGGTTACTTCTCTTCCATTTTTCATGG
TGGAGTGGATAGTCTAGTCGACTTGATGAACAAGAGCTTTCCTGAGTTGGGTATTAAAAAAACTGATTGCAAA
GAATTGAGCAAAATTGATACAACCATCTTCTACAGTAAAGTTGTAAATTACAACACTGCTAATGCTTTTAAAA
AGGAAATTTTGCTTAAAAGATCAGCTGGGTTACAGAAGACGGCTTTACCTTTCTCAATTAAGTTAGACAAAGT
TAAGAAACCAATTCCAGAAACCGCAATGGTCAAAATTTTGGAAAAATTATATGAAGAAGATGTAGGAGTTGGG
ATGAAAGTGTTGTACCCTTACGGTGGTATAATGGATGAGATTTCAGAAAAAGCAATTCCATTCCCTCATAAAG
CTGGAATCATGTATGAACTTAAATACACAGCTACCAAAGATGAGAAGCAAGAAGATATTAATGAAAAAGCAGC
TGAAATTATTAAGCATATAAACTGGGTTAAAAGTGTTTATAATTTCACGACTCCTTATGTGTCCAAAAATCCA
AGAATGGCGTATCTCAATTATAGGGACCTTGATTTAGGAAAAACTAATCCTGAGAGTCCTAATAATTACACAC
AAGCACGTATTTGGGGTGAAAAGTATTTTGGTAAAAACAATTTTAACAGGTTAGTTAAGGTGAAAACCAAAGT
TGATCCCAATAATTTTTTTAGAAACGAACAAAGTATCCCACCTCTTATGTCATTTCCATTGCGGCCGCGTCAT
CATAAAAAAAATAAATATGGACCTAAATATGAGCTTTCTTATTATTATACTAGTTTCTGGACACGTAACTCCC
ATTTGGCCATATGTAGGGCCATATACAATAAGTTGAAGTGTTTAATGAAA

>A1A2a_PAML_Codon_Gap
ATGAAT---TGCAAAACA---TTC---------TCCTTTTGGTTTGTTTGCAAA------ATAATA---
TTTTTC------TTTCTC---TCATTCAATATCCAAATTTCAATAGCTAAT---CCTCAA---------
GAAAACTTCCTTAAATGCTTCTCGCAATATATTCCC------AACAATGCAGCAAATCCA---
AAACTCGTATACACTCAACACGACCAATTGTATATGTCTGTCCTGAATTCGACAATACAAAATCTTAGATTCA
CCTCTGATACAACCCCAAAACCACTCGTTATTGTCACTCCTTCAAATGTCTCCCATATCCAAGCCACTATTCT
ATGCTCCAAGAAAGTTGGCTTGAAAATTCGAACTAAAAGCGGTGGCCATGATGCTGAGGGTTTGTCCTACATA
TCTCAA---
GTCCCATTTGTTATAGTAGACTTGAGAAACATGCATAAAGTCAAAATAGATGTTCATAGCCAAACTGCGTGGG
TTGAAGCCGGAGCTACCCTTGGAGAAGTTTATTATTGGATCAATGAGAAGAATGAGAATCTTAGTTTTCCTGG
TGGGTATTGCCCTACTGTTGGCGCAGGTGGACACTTTAGTGGAGGAGGCTATGGAGCATTGATGAAAAATTAT
GGCCTCGCGGCTGATAATATCATTGATGCACACTTAGTCAATGTTGATGGAAAAGTTCTAGATCGAAAATCCA
TGGGAGAAGATCTGTTTTGGGCTATACGTGGTGGTGGAGGAGAAAACTTTGGAATCATTGCAGCATGGAAAAT
CAAACTGGTTGCTGTCCCATCAAAGTCTACTATATTCAGTGTTAAAAAGAACATGGAGATACATGGGCTTGTC
AAGAAATTTAACAAAAAACAAAATATTGCTTACAAGTATGACAAAGATAAATTACTCATGACTCACTTCATAA
CCAGGAATATTACAGATAATCATGGGAAGAAT---------
AAGACTACAGTACATGGTTACTTCTCTTCCATTTTTCATGGTGGAGTGGATAGTCTAGTCGACTTGATGAACA
AGAGCTTTCCTGAGTTGGGTATTAAAAAAACTGATTGCAAAGAATTGAGCAAAATTGATACAACCATCTTCTA
CAGTAAAGTTGTAAATTACAACACTGCTAAT---TTTAAAAAGGAAATTTTGCTTAAAAGATCAGCTGGG---
CAGAAGACGGCT------
TTCTCAATTAAGTTAGACAAAGTTAAGAAACCAATTCCAGAAACCGCAATGGTCAAAATTTTGGAAAAATTAT
ATGAAGAAGATGTAGGAGTTGGGATGAAAGTGTTGTACCCTTACGGTGGTATAATGGATGAGATTTCAGAAAA
AGCAATTCCATTCCCTCATAAAGCTGGAATCATGTATGAACTTAAATACACAGCTACCAAA---
GAGAAGCAAGAAGAT---AATGAA------------------
AAGCATATAAACTGGGTTAAAAGTGTTTATAATTTCACGACTCCTTATGTGTCCAAAAATCCAAGAATGGCGT
ATCTCAATTATAGGGACCTTGATTTAGGAAAAACTAATCCTGAGAGTCCTAATAATTACACACAAGCACGTAT
TTGGGGTGAAAAGTATTTTGGTAAA---
AATTTTAACAGGTTAGTTAAGGTGAAAACCAAAGTTGATCCCAATAATTTTTTTAGAAACGAACAAAGTATCC
CACCTCTT---------CCA------CCGCGTCATCATAAA

```
>A1A2a_PAML_AminoAcid_Raw
MNYCSTVFIPSSFWFVCKYSIILFFISFLYSFNIQISIANSPQREHENFLKCFSQYIPNNNNPANPAKLVYTQ
HDQLYMSVLNSTIQNLRFTSDTTPKPLVIVTPSNVSHIQATILCSKKVGLQIRTRSGGHDAEGMSYISQLVPF
VIVDLRNMHSVKIDVHSQTAWVEAGATLGEVYYWINEKNENLSFPGGYCPTVGAGGHFSGGGYGALMRNYGLA
ADNIIDAHLVNVDGKVLDRKSMGEDLFWAIRGGGGENFGIIAAWKIKLVAVPSKSTIFSVKKNMEIHGLVKLF
NKWQNIAYKYDKDLLLMTHFITRNITDNHGKNSKEKTTVHGYFSSIFHGGVDSLVDLMNKSFPELGIKKTDCK
ELSWIDTTIFYSGVVNYNTANAFKKEILLDRSAGSQKTALPFSIKLDYVKKPIPETAMVKILEKLYEEDVGVG
MYVLYPYGGIMDEISESAIPFPHRAGIMYELWYTATWEEKQEDINEKAAEIIKHINWVRSVYNFTTPYVSQNP
RMAYLNYRDLDLGKTNPESPNNYTQARIWGEKYFGKNNFNRLVKVKTKVDPNNFFRNEQSIPPLLSFPLRPRH
HKGNNYGPGYELSYYYTSFWTRNSHLAICRAIYNKLKCLMLL

>A1A2_PAML_AminoAcid_Gap
MN-CST-F---SFWFVCK--II-FF--FL-SFNIQISIAN-PQ---ENFLKCFSQYIP--NNPANP-
KLVYTQHDQLYMSVLNSTIQNLRFTSDTTPKPLVIVTPSNVSHIQATILCSKKVGLQIRTRSGGHDAEGMSYI
SQ-
VPFVIVDLRNMHSVKIDVHSQTAWVEAGATLGEVYYWINEKNENLSFPGGYCPTVGAGGHFSGGGYGALMRNY
GLAADNIIDAHLVNVDGKVLDRKSMGEDLFWAIRGGGGENFGIIAAWKIKLVAVPSKSTIFSVKKNMEIHGLV
KLFNKWQNIAYKYDKDLLLMTHFITRNITDNHGKN---
KTTVHGYFSSIFHGGVDSLVDLMNKSFPELGIKKTDCKELSWIDTTIFYSGVV----AN-FKKEILLDRSAG-
QKTA--
FSIKLDYVKKPIPETAMVKILEKLYEEDVGVGMYVLYPYGGIMDEISESAIPFPHRAGIMYELWYTATW-
EKQED-NE------KHINWVRSVYNFTTPYVSQNPRMAYLNYRDLDLGKTNPESPNNYTQARIWGEKYFGK-
NFNRLVKVKTKVDPNNFFRNEQSIPPL---P--PRHHK

>Ca_ MrBayes_Raw
ATGAAGTACTACTCAACAGTATTCATACCTTCTTCCTTTTGGTTTGTTTGCAAGTACTCTATAATATTATTTT
TCATTTCTTTTCTCTATTCATTCAATATCCAAACTTCAATAGCTAATTCTCCTCAACGTGATCATGAAAACTT
CCTTAAATGCTTCTCGCAATATATTCCCAACAATAACAATGCAACAAATCTAGCCAAACTCGTATACACTCAA
AACGACCAATTGTATATGTCTGTCCTAAATTCGACAATACAAAATCTTAGATTCACCTCTGACACAACCCCAA
AACCACTTGTTATCGTCACTCCTTCAAATGTCTCCCATATCCAAGGCACTATTCTATGCTCCAAGAAAGTTGG
CTTGCAGATTCGAACTCGAAGCGGTGGTCATGATTCTGAGGGCATGTCCTACATATCTCAATTAGTCCCATTT
GTTATAGTAGACTTGAGAAACATGCATTCGATCAAAATAGATGTTCATAGCCAAACTGCATGGGTTGAAGCCG
GAGCTACCCTTGGAGAAGTTTATTATTGGATTAATGAGAAAAATGAGAATCTTAGTTTTCCTGCTGGGTATTG
CCCTACTGTTGGCGCAGGTGGACACTTTAGTGGAGGAGGCTATGGAGCATTGATGCGAAATTATGGCCTCGCG
GCTGATAATATCATTGATGCACACTTAGTCAACGTTGATGGAAAAGTTCTAGATCGAAAATCCATGGGGGAAG
ATCTGTTTTGGGCTATACGTGGTGGTGGAGGAGAAAGCTTTGGAATCATTGTAGCGTGGAAAATTAGACTGGT
TGCTGTCCCATCAAAGTCTACTATATTCAGTGTTAAAAAGAACATGGAGATACATGAGCTTGTCAAGTTAGTT
AACAAATGGCAAAATATTGCTTACAAGTATGACAAAGATTTATTACTCATGACTCACTTCATAACTAGGAATA
TTACAGATAATCAAGGGAAGAATAGCAAGGAAAAGACAACAATACACACTTACTTCTCTTCCATTTTCCTTGG
TGGAGTGGATAGTCTAGTCGACTTGATGAACAAGAGCTTTCCTGAGTTGGGTATTAAAAAAACAGATTGCAAA
CAATTGAGCTGGATTGATACTATCATCTTCTACAGTGGTGTTGTAAATTACAACACTGCTAATGCTTTTAAAA
AGGAAATTTTGCTTGATAGATCAGCTGGGTTACAGAAGGGGGCTTTACCTTTCAAGATTAAGTTAGACTACGT
TAAGAAACCAATTCCAGAAACTGCAATGGTCAAAATTTTGGAAAAATTATATGAAGAAGATGTAGGAGTTGGG
ATGTATGTGTTGTACCCTTACGGTGGTATAATGGATGAGATTTCAGAATCAGCAATTCCATTCCCTCATCGAG
CTGGAATCATGTATGAAATTTGGTACATATCTACCTGGGATGAGAAGCAAGAAGATATTAATGAAAAAGCAGT
TGAAATTATTAAGCATATAAACTGGATTCGAAATGTTTATAATTTCATGACTCCTTATGTGTCCCAAAATCCA
AGAATGGCATATCTCAATTATAGGGACCTTGATTTAGGAAAAAATAATCCCAAGAGTCCTAATAATTACACAC
AAGCACGTATTTGGGGTGAAAAGTATTTTGGTAAAAACAATTTTAACAGGTTAGTAAAAGTGAAAACCAAGGT
TGATCCCAATAATTTTTTTAGAAACGAACAAAGCATCCCACCTCTTATGTCATTTCCATTGCGGCCGCGTCAT
CATTAATGAAATTAATATGGACCTTGATATGAGCTTTCTTATTATTATACTAGTTTCTGGACACGTAACTCCC
ATTTGGCCATATGTAGGGCCATATACAATAAGTTGAAGTGTTTAATGTAA

>Ca_MrBayes_Gap
ATGAAG---TACTCAACA---TTC---------TCCTTTTGGTTTGTTTGCAAG------ATAATA---
TTTTTC------TTTCTC---TCATTCAATATCCAAACTTCAATAGCTAAT---CCTCAA---------
GAAAACTTCCTTAAATGCTTCTCGCAATATATTCCC------AACAATGCAACAAATCTA---
AAACTCGTATACACTCAAAACGACCAATTGTATATGTCTGTCCTAAATTCGACAATACAAAATCTTAGATTCA
CCTCTGACACAACCCCAAAACCACTTGTTATCGTCACTCCTTCAAATGTCTCCCATATCCAAGGCACTATTCT
```

ATGCTCCAAGAAAGTTGGCTTGCAGATTCGAACTCGAAGCGGTGGTCATGATTCTGAGGGCATGTCCTACATA
TCTCAA---
GTCCCATTTGTTATAGTAGACTTGAGAAACATGCATTCGATCAAAATAGATGTTCATAGCCAAACTGCATGGG
TTGAAGCCGGAGCTACCCTTGGAGAAGTTTATTATTGGATTAATGAGAAAAATGAGAATCTTAGTTTTCCTGC
TGGGTATTGCCCTACTGTTGGCGCAGGTGGACACTTTAGTGGAGGAGGCTATGGAGCATTGATGCGAAATTAT
GGCCTCGCGGCTGATAATATCATTGATGCACACTTAGTCAACGTTGATGGAAAAGTTCTAGATCGAAAATCCA
TGGGGGAAGATCTGTTTTGGGCTATACGTGGTGGTGGAGGAGAAAGCTTTGGAATCATTGTAGCGTGGAAAAT
TAGACTGGTTGCTGTCCCATCAAAGTCTACTATATTCAGTGTTAAAAAGAACATGGAGATACATGAGCTTGTC
AAGTTAGTTAACAAATGGCAAAATATTGCTTACAAGTATGACAAAGATTTATTACTCATGACTCACTTCATAA
CTAGGAATATTACAGATAATCAAGGGAAGAAT---------
AAGACAACAATACACACTTACTTCTCTTCCATTTTCCTTGGTGGAGTGGATAGTCTAGTCGACTTGATGAACA
AGAGCTTTCCTGAGTTGGGTATTAAAAAAACAGATTGCAAACAATTGAGCTGGATTGATACTATCATCTTCTA
CAGTGGTGTTGTAAATTACAACACTGCTAAT---TTTAAAAAGGAAATTTTGCTTGATAGATCAGCTGGG---
CAGAAGGGGGCT------
TTCAAGATTAAGTTAGACTACGTTAAGAAACCAATTCCAGAAACTGCAATGGTCAAAATTTTGGAAAAATTAT
ATGAAGAAGATGTAGGAGTTGGGATGTATGTGTTGTACCCTTACGGTGGTATAATGGATGAGATTTCAGAATC
AGCAATTCCATTCCCTCATCGAGCTGGAATCATGTATGAAATTTGGTACATATCTACCTGG---
GAGAAGCAAGAAGAT---AATGAA------------------
AAGCATATAAACTGGATTCGAAATGTTTATAATTTCATGACTCCTTATGTGTCCCAAAATCCAAGAATGGCAT
ATCTCAATTATAGGGACCTTGATTTAGGAAAAAATAATCCCAAGAGTCCTAATAATTACACACAAGCACGTAT
TTGGGGTGAAAAGTATTTTGGTAAA---
AATTTTAACAGGTTAGTAAAAGTGAAAACCAAGGTTGATCCCAATAATTTTTTTAGAAACGAACAAAGCATCC
CACCTCTT---------CCA---CGGCCGCGT---CATTAA

>Ca_PAML_Nucleotide_Raw
ATGAAGTACTACTCAACAGTATTCATACCTTCTTCCTTTTGGTTTGTTTGCAAGTACTCTATAATATTATTTT
TCATTTCTTTTCTCTATTCATTCAATATCCAAACTTCAATAGCTAATTCTCCTCAACGTGATCATGAAAACTT
CCTTAAATGCTTCTCGCAATATATTCCCAACAATAACAATGCAACAAATCTAGCCAAACTCGTATACACTCAA
AACGACCAATTGTATATGTCTGTCCTGAATTCGACAATACAAAATCTTAGATTCACCTCTGACACAACCCCAA
AACCACTTGTTATCGTCACTCCTTCAAATGTCTCCCATATCCAAGGCACTATTCTATGCTCCAAGAAAGTTGG
CTTGCAGATTCGAACTCGAAGCGGTGGTCATGATTCTGAGGGCATGTCCTACATATCTCAATTAGTCCCATTT
GTTATAGTAGACTTGAGAAACATGCATTCGATCAAAATAGATGTTCATAGCCAAACTGCATGGGTTGAAGCCG
GAGCTACCCTTGGAGAAGTTTATTATTGGATTAATGAGAAAAATGAGAATCTTAGTTTTCCTGCTGGGTATTG
CCCTACTGTTGGCGCAGGTGGACACTTTAGTGGAGGAGGCTATGGAGCATTGATGCGAAATTATGGCCTCGCG
GCTGATAATATCATTGATGCACACTTAGTCAACGTTGATGGAAAAGTTCTAGATCGAAAATCCATGGGGGAAG
ATCTGTTTTGGGCTATACGTGGTGGTGGAGGAGAAAGCTTTGGAATCATTGTAGCGTGGAAAATTAGACTGGT
TGCTGTCCCATCAAAGTCTACTATATTCAGTGTTAAAAAGAACATGGAGATACATGAGCTTGTCAAGTTAGTT
AACAAATGGCAAAATATTGCTTACAAGTATGACAAAGATTTATTACTCATGACTCACTTCATAACTAGGAATA
TTACAGATAATCAAGGGAAGAATAGCAAGGAAAAGACAACAATACACACTTACTTCTCTTCCATTTTCCTTGG
TGGAGTGGATAGTCTAGTCGACTTGATGAACAAGAGCTTTCCTGAGTTGGGTATTAAAAAAACAGATTGCAAA
CAATTGAGCTGGATTGATACTATCATCTTCTACAGTGGTGTTGTAAATTACAACACTGCTAATGCTTTTAAAA
AGGAAATTTTGCTTGATAGATCAGCTGGGTTACAGAAGGGGGCTTTACCTTTCAAGATTAAGTTAGACTACGT
TAAGAAACCAATTCCAGAAACCGCAATGGTCAAAATTTTGGAAAAATTATATGAAGAAGATGTAGGAGTTGGG
ATGTATGTGTTGTACCCTTACGGTGGTATAATGGATGAGATTTCAGAATCAGCAATTCCATTCCCTCATCGAG
CTGGAATCATGTATGAAATTTGGTACATATCTACCTGGGATGAGAAGCAAGAAGATATTAATGAAAAAGCAGC
TGAAATTATTAAGCATATAAACTGGATTCGAAATGTTTATAATTTCATGACTCCTTATGTGTCCCAAAATCCA
AGAATGGCATATCTCAATTATAGGGACCTTGATTTAGGAAAAAATAATCCCAAGAGTCCTAATAATTACACAC
AAGCACGTATTTGGGGTGAAAAGTATTTTGGTAAAAACAATTTTAACAGGTTAGTAAAAGTGAAAACCAAGGT
TGATCCCAATAATTTTTTTAGAAACGAACAAAGCATCCCACCTCTTATGTCATTTCCATTGCGGCAGCGTCAT
CATTAATGAAATTAATATGGACCTTGATATGAGCTTTCTTATTATTATACTAGTTTCTGGACACGTAACTCCC
ATTTGGCCATATGTAGGGCCATATACAATAAGTTGAAGTGTTTAATGTAA

>Ca_PAML_Nucleotide_Gap
ATGAAG---TACTCAACA---TTC---------TCCTTTTGGTTTGTTTGCAAG------ATAATA---
TTTTTC------TTTCTC---TCATTCAATATCCAAACTTCAATAGCTAAT---CCTCAA---------
GAAAACTTCCTTAAATGCTTCTCGCAATATATTCCC------AACAATGCAACAAATCTA---
AAACTCGTATACACTCAAAACGACCAATTGTATATGTCTGTCCTGAATTCGACAATACAAAATCTTAGATTCA
CCTCTGACACAACCCCAAAACCACTTGTTATCGTCACTCCTTCAAATGTCTCCCATATCCAAGGCACTATTCT

ATGCTCCAAGAAAGTTGGCTTGCAGATTCGAACTCGAAGCGGTGGTCATGATTCTGAGGGCATGTCCTACATA
TCTCAA---
GTCCCATTTGTTATAGTAGACTTGAGAAACATGCATTCGATCAAAATAGATGTTCATAGCCAAACTGCATGGG
TTGAAGCCGGAGCTACCCTTGGAGAAGTTTATTATTGGATTAATGAGAAAAATGAGAATCTTAGTTTTCCTGC
TGGGTATTGCCCTACTGTTGGCGCAGGTGGACACTTTAGTGGAGGAGGCTATGGAGCATTGATGCGAAATTAT
GGCCTCGCGGCTGATAATATCATTGATGCACACTTAGTCAACGTTGATGGAAAAGTTCTAGATCGAAAATCCA
TGGGGGAAGATCTGTTTTGGGCTATACGTGGTGGTGGAGGAGAAAGCTTTGGAATCATTGTAGCGTGGAAAAT
TAGACTGGTTGCTGTCCCATCAAAGTCTACTATATTCAGTGTTAAAAAGAACATGGAGATACATGAGCTTGTC
AAGTTAGTTAACAAATGGCAAAATATTGCTTACAAGTATGACAAAGATTTATTACTCATGACTCACTTCATAA
CTAGGAATATTACAGATAATCAAGGGAAGAAT---------
AAGACAACAATACACACTTACTTCTCTTCCATTTTCCTTGGTGGAGTGGATAGTCTAGTCGACTTGATGAACA
AGAGCTTTCCTGAGTTGGGTATTAAAAAAACAGATTGCAAACAATTGAGCTGGATTGATACTATCATCTTCTA
CAGTGGTGTTGTAAATTACAACACTGCTAAT---TTTAAAAAGGAAATTTTGCTTGATAGATCAGCTGGG---
CAGAAGGGGGCT------
TTCAAGATTAAGTTAGACTACGTTAAGAAACCAATTCCAGAAACCGCAATGGTCAAAATTTTGGAAAAATTAT
ATGAAGAAGATGTAGGAGTTGGGATGTATGTGTTGTACCCTTACGGTGGTATAATGGATGAGATTTCAGAATC
AGCAATTCCATTCCCTCATCGAGCTGGAATCATGTATGAAATTTGGTACATATCTACCTGG---
GAGAAGCAAGAAGAT---AATGAA------------------
AAGCATATAAACTGGATTCGAAATGTTTATAATTTCATGACTCCTTATGTGTCCCAAAATCCAAGAATGGCAT
ATCTCAATTATAGGGACCTTGATTTAGGAAAAAATAATCCCAAGAGTCCTAATAATTACACACAAGCACGTAT
TTGGGGTGAAAAGTATTTTGGTAAA---
AATTTTAACAGGTTAGTAAAAGTGAAAACCAAGGTTGATCCCAATAATTTTTTTAGAAACGAACAAAGCATCC
CACCTCTT---------CCA---CGGCAGCGT---CATTAA

>Ca_PAML_Codon_Raw
ATGAAGTACTACAAAACAGTATTCATACCTTCTTCCTTTTGGTTTGTTTGCAAGTACTCTATAATATTATTTT
TCATTTCTTTTCTCTATTCATTCAATATCCAAACTTCAATAGCTAATTCTCCTCAACGTGATCATGAAAACTT
CCTTAAATGCTTCTCGCAATATATTCCCAACAATAACAATGCAACAAATCTAGCCAAACTCGTATACACTCAA
AACGACCAATTGTATATGTCTGTCCTGAATTCGACAATACAAAATCTTAGATTCACCTCTGACACAACCCCAA
AACCACTTGTTATCGTCACTCCTTCAAATGTCTCCCATATCCAAGGCACTATTCTATGCTCCAAGAAAGTTGG
CTTGAAAATTCGAACTAAAAGCGGTGGTCATGATTCTGAGGGCATGTCCTACATATCTCAATTAGTCCCATTT
GTTATAGTAGACTTGAGAAACATGCATAAAATCAAAATAGATGTTCATAGCCAAACTGCATGGGTTGAAGCCG
GAGCTACCCTTGGAGAAGTTTATTATTGGATTAATGAGAAAAATGAGAATCTTAGTTTTCCTGCTGGGTATTG
CCCTACTGTTGGCGCAGGTGGACACTTTAGTGGAGGAGGCTATGGAGCATTGATGAAAAATTATGGCCTCGCG
GCTGATAATATCATTGATGCACACTTAGTCAACGTTGATGGAAAAGTTCTAGATCGAAAATCCATGGGGGAAG
ATCTGTTTTGGGCTATACGTGGTGGTGGAGGAGAAAGCTTTGGAATCATTGTAGCGTGGAAAATTAGACTGGT
TGCTGTCCCATCAAAGTCTACTATATTCAGTGTTAAAAAGAACATGGAGATACATGAGCTTGTCAAGAAAGTT
AACAAAAAACAAAATATTGCTTACAAGTATGACAAAGATAAATTACTCATGACTCACTTCATAACTAGGAATA
TTACAGATAATCAAGGGAAGAATAGCAAGGAAAAGACAACAATACACACTTACTTCTCTTCCATTTTCCTTGG
TGGAGTGGATAGTCTAGTCGACTTGATGAACAAGAGCTTTCCTGAGTTGGGTATTAAAAAAACAGATTGCAAA
CAATTGAGCAAAATTGATACTATCATCTTCTACAGTAAAGTTGTAAATTACAACACTGATAATGCTTTTAAAA
AGGAAATTTTGCTTAAAAGATCAGCTGGGTTACAGAAGGGTGCTTTACCTTTCAAGATTAAGTTAGACAAAGT
TAAGAAACCAATTCCAGAAACTGCAATGGTCAAAATTTTGGAAAAATTATATGAAGAAGATGTAGGAGTTGGG
ATGAAAGTGTTGTACCCTTACGGTGGTATAATGGATGAGATTTCAGAAAAAGCAATTCCATTCCCTCATAAAG
CTGGAATCATGTATGAAATTAAATACATATCTACCAAAGATGAGAAGCAAGAAGATATTAATGAAAAAGCAGC
TGAAATTATTAAGCATATAAACTGGATTAAAAATGTTTATAATTTCATGACTCCTTATGTGTCCAAAAATCCA
AGAATGGCATATCTCAATTATAGGGACCTTGATTTAGGAAAAAATAATCCCAAGAGTCCTAATAATTACACAC
AAGCACGTATTTGGGGTGAAAAGTATTTTGGTAAAAACAATTTTAACAGGTTAGTAAAAGTGAAAACCAAGGT
TGATCCCAATAATTTTTTTAGAAACGAACAAAGCATCCCACCTCTTATGTCATTTCCATTGCGGCATCGTCAT
CATAAAAAAAATAAATATGGACCTAAATATGAGCTTTCTTATTATTATACTAGTTTCTGGACACGTAACTCCC
ATTTGGCCATATGTAGGGCCATATACAATAAGTTGAAGTGTTTAATGAAA

>Ca_PAML_Codon_Gap
ATGAAG---TACAAAACA---TTC---------TCCTTTTGGTTTGTTTGCAAG------ATAATA---
TTTTTC------TTTCTC---TCATTCAATATCCAAACTTCAATAGCTAAT---CCTCAA---------
GAAAACTTCCTTAAATGCTTCTCGCAATATATTCCC------AACAATGCAACAAATCTA---
AAACTCGTATACACTCAAAACGACCAATTGTATATGTCTGTCCTGAATTCGACAATACAAAATCTTAGATTCA
CCTCTGACACAACCCCAAAACCACTTGTTATCGTCACTCCTTCAAATGTCTCCCATATCCAAGGCACTATTCT

ATGCTCCAAGAAAGTTGGCTTGAAAATTCGAACTAAAAGCGGTGGTCATGATTCTGAGGGCATGTCCTACATA
TCTCAA---
GTCCCATTTGTTATAGTAGACTTGAGAAACATGCATAAAATCAAAATAGATGTTCATAGCCAAACTGCATGGG
TTGAAGCCGGAGCTACCCTTGGAGAAGTTTATTATTGGATTAATGAGAAAAATGAGAATCTTAGTTTTCCTGC
TGGGTATTGCCCTACTGTTGGCGCAGGTGGACACTTTAGTGGAGGAGGCTATGGAGCATTGATGAAAAATTAT
GGCCTCGCGGCTGATAATATCATTGATGCACACTTAGTCAACGTTGATGGAAAAGTTCTAGATCGAAAATCCA
TGGGGGAAGATCTGTTTTGGGCTATACGTGGTGGTGGAGGAGAAAGCTTTGGAATCATTGTAGCGTGGAAAAT
TAGACTGGTTGCTGTCCCATCAAAGTCTACTATATTCAGTGTTAAAAAGAACATGGAGATACATGAGCTTGTC
AAGAAAGTTAACAAAAAACAAAATATTGCTTACAAGTATGACAAAGATAAATTACTCATGACTCACTTCATAA
CTAGGAATATTACAGATAATCAAGGGAAGAAT---------
AAGACAACAATACACACTTACTTCTCTTCCATTTTCCTTGGTGGAGTGGATAGTCTAGTCGACTTGATGAACA
AGAGCTTTCCTGAGTTGGGTATTAAAAAAACAGATTGCAAACAATTGAGCAAAATTGATACTATCATCTTCTA
CAGTAAAGTTGTAAATTACAACACTGATAAT---TTTAAAAAGGAAATTTTGCTTAAAAGATCAGCTGGG---
CAGAAGGGTGCT------
TTCAAGATTAAGTTAGACAAAGTTAAGAAACCAATTCCAGAAACTGCAATGGTCAAAATTTTGGAAAAATTAT
ATGAAGAAGATGTAGGAGTTGGGATGAAAGTGTTGTACCCTTACGGTGGTATAATGGATGAGATTTCAGAAAA
AGCAATTCCATTCCCTCATAAAGCTGGAATCATGTATGAAATTAAATACATATCTACCAAA---
GAGAAGCAAGAAGAT---AATGAA------------------
AAGCATATAAACTGGATTAAAAATGTTTATAATTTCATGACTCCTTATGTGTCCAAAAATCCAAGAATGGCAT
ATCTCAATTATAGGGACCTTGATTTAGGAAAAAATAATCCCAAGAGTCCTAATAATTACACACAAGCACGTAT
TTGGGGTGAAAAGTATTTTGGTAAA---
AATTTTAACAGGTTAGTAAAAGTGAAAACCAAGGTTGATCCCAATAATTTTTTTAGAAACGAACAAAGCATCC
CACCTCTT---------CCA---CGGCATCGT---CATAAA

>Ca_PAML_AminoAcid_Raw
MKYYSTVFIPSSFWFVCKYSIILFFISFLYSFNIQTSIANSPQREHENFLKCFSQYIPNNNNVTNLAKLVYTQ
NDQLYMSVLNSTIQNLRFTSDTTPKPLVIVTPSNVSHIQGTILCSKKVGLQIRTRSGGHDSEGMSYISQLVPF
VIVDLRNMHSIKIDVHSQTAWVEAGATLGEVYYWINEKNENLSFPAGYCPTVGAGGHFSGGGYGALMRNYGLA
ADNIIDAHLVNVDGKVLDRKSMGEDLFWAIRGGGGESFGIIVAWKIRLVAVPSKSTIFSVKKNMEIHELVKLV
NKWQNIAYKYDKDLLLMTHFITRNITDNQGKNSKEKTTIHTYFSSIFLGGVDSLVDLMNKSFPELGIKKTDCK
QLSWIDTIIFYSGVVNYNTANAFKKEILLDRSAGSQKGALPFKIKLDYVKKPIPETAMVKILEKLYEEDVGVG
MYALYPYGGIMDEISESAIPFPHRAGIMYELWYIASWEEKQEDINEKAAEIIKHLNWIRNVYNFMTPYVSQNP
RMAYLNYRDLDLGKNNPKSPNNYTQARIWGEKYFGKNNFNRLVKVKTKVDPNNFFRNEQSIPPLLSFPLRHRH
HKGNNYGPGYELSYYYTSFWTRNSHLAICRAIYNKLKCLMLL

>Ca_PAML_AminoAcid_Gap
MK-YST-F---SFWFVCK--II-FF--FL-SFNIQTSIAN-PQ---ENFLKCFSQYIP--NNVTNL-
KLVYTQNDQLYMSVLNSTIQNLRFTSDTTPKPLVIVTPSNVSHIQGTILCSKKVGLQIRTRSGGHDSEGMSYI
SQ-
VPFVIVDLRNMHSIKIDVHSQTAWVEAGATLGEVYYWINEKNENLSFPAGYCPTVGAGGHFSGGGYGALMRNY
GLAADNIIDAHLVNVDGKVLDRKSMGEDLFWAIRGGGGESFGIIVAWKIRLVAVPSKSTIFSVKKNMEIHELV
KLVNKWQNIAYKYDKDLLLMTHFITRNITDNQGKN---
KTTIHTYFSSIFLGGVDSLVDLMNKSFPELGIKKTDCKQLSWIDTIIFYSGVV----AN-FKKEILLDRSAG-
QKGA--
FKIKLDYVKKPIPETAMVKILEKLYEEDVGVGMYALYPYGGIMDEISESAIPFPHRAGIMYELWYIASW-
EKQED-NE------KHLNWIRNVYNFMTPYVSQNPRMAYLNYRDLDLGKNNPKSPNNYTQARIWGEKYFGK-
NFNRLVKVKTKVDPNNFFRNEQSIPPL---P-RHR-HK

>HCa_MrBayes_Raw
ATGAAGTACTACTCAACAGTATTCATACCTTCTTCTTTTTGGTTTCTTTGCAAATACTCTATATTATTAGTTT
ATATTTCTCTTCTCTATTCATTCTCTATCCAAACTTCTCAAGCTAATTCTCCTCACCGTGATCATGAAGACTT
CCTTCAATGCTTCTCCCAACATATCTCCAACAATAACAATACAACAACCCTTGCCAAACTCATATACACTCCA
AACGACCCGTCGTATATCTCTGTCCTAAATTCGACCATACAAAACCTTAGATTCTCTTCTCCTTCAACCCCAA
AACCACTCGTTATCGTCACACCTTCAAATGCCTCCCATGTCCAAGCCTCTGTTTTATGCTCCAAGAAATATGG
CTTGCAGATTCGAACTCGAAGCGGCGGCCATGACTTTGAGGGTGTCTCCTACGTGTCTGAATTAGTCCCATTT
GTCATAGTAGACATGAGAAACCTACGTTCGATCACTGTAGACGTAGATAACAAAACTGCATGGGTTGAAGCTG
GAGCTACCCTTGGAGAAGTTTATTATAGGATTGCTGAGAAAAATGAGAATCTCAGTTTTCCTGCTGGCTATTG
CCATACTGTTGGCGTAGGTGGGCACTTCAGTGGAGGAGGCTATGGAGCATTGATGCGAAAATATGGCCTTGCA

GCTGATAATATCATTGATGCTCACTTAGTCAACGTTGATGGAAAAGTTCTTGACCGAAAATCTATGGGGGAAG
ATCTGTTTTGGGCCATACGTGGTGGTGGAGGAGCAAGCTTTGGAATCATTCTCGCTTGGAAAATTAGATTGGT
TCCTGTCCCATCAAAGGTTACTATATTCACTGTTAATAAGAACTTGGAGATAAATGAAACCGTGAAGCTTGTT
AACAAGTGGCAAAATATTGCTCACAAGTTTGACAAAGATTTGTTAATCTTTGTTAGGTTCATAACTATGAATT
CTACTGATGAACAAGGGAAGAATAGCAAGGAAAAGACAACAATACAAGCTTCATTCTCTTCTATTTTCCTTGG
TGGAGTGGATAGTCTACTTGCCTTGATGGAAAAGAGCTTTCCTGAGTTGGGTGTGAAAAGAAAAGATTGCATC
GAAATGAGCTGGATTGAAACTATCTTTTACTTCAACGGATTCTCAAATTACAACACTAGTGGGGCTGATAAAT
TGGAAGTTTTGCTTGATAGAACAACTGAATTACAAAAGGGTTTTTTACCTTTCAAGGCGAAACTAGACTACGT
TAAGAAGCCAATTCCAGAAACTGTAATGGTCAAAATTTTGGAGAAGTTATATGAAGAAGATGTAGGAGTGGGA
TTGATTCAATTGTACCCTTATGGTGGTAAAATGGACGAGATTCCTGAATCAGCAATTCCATTCCCTCACCGAG
CTGGAATCATGTACAAAATTTTGTACTTGTCTCAATGGGATGAGAAAGAAGAAGATATTGGTGAAAAAGCAGC
TGAAATTATTAGGCATATGAATTGGGTTCGAAGTGTTTACAATTACATGACTCCCTATGTGTCCAAAAATCCA
AGAGCTGCATATCTCAATTATAGGGACCTTGATTTGGGAACAAATAACGACAAGGGTCCTACTAGTTATGCAC
AAGCAAGTATTTGGGGAAAAAAGTATTTCGGTAAAAACAACTTTAAGAGGTTAGTTCATGTGAAAACCAAGGT
TGATCCCACTAATTTCTTCAGGAACGAACAAAGCATCCCACCTCTTCTGTCATTTCCGTTGCGACGTGTCAAG
CTCTAAAGTAATTAATATGGACCTTGATATGAGCTTTCTTATTATTATACTAGTTTCTGGACACGTAACTCCC
ATTTGGCCATATGTAGGGCCATATACAATAAGTTGAAGTGTTTAATGTAA

>HCa_MrBayes_Gap
ATGAAG---TACTCAACA---TTC---------TCTTTTTGGTTTCTTTGCAAA------ATATTA---
GTTTAT---TCTCTTCTC---TCATTCTCTATCCAAACTTCTCAAGCTAAT---CCTCAC---------
GAAGACTTCCTTCAATGCTTCTCCCAACATATCTCC------AACAAT---
ACAACCCTTGCCAAACTCATATACACTCCAAACGACCCGTCGTATATCTCTGTCCTAAATTCGACCATACAAA
ACCTTAGATTCTCTTCTCCTTCAACCCCAAAACCACTCGTTATCGTCACACCTTCAAATGCCTCCCATGTCCA
AGCCTCTGTTTTATGCTCCAAGAAATATGGCTTGCAGATTCGAACTCGAAGCGGCGGCCATGACTTTGAGGGT
GTCTCCTACGTGTCTGAA---
GTCCCATTTGTCATAGTAGACATGAGAAACCTACGTTCGATCACTGTAGACGTAGATAACAAAACTGCATGGG
TTGAAGCTGGAGCTACCCTTGGAGAAGTTTATTATAGGATTGCTGAGAAAAATGAGAATCTCAGTTTTCCTGC
TGGCTATTGCCATACTGTTGGCGTAGGTGGGCACTTCAGTGGAGGAGGCTATGGAGCATTGATGCGAAAATAT
GGCCTTGCAGCTGATAATATCATTGATGCTCACTTAGTCAACGTTGATGGAAAAGTTCTTGACCGAAAATCTA
TGGGGGAAGATCTGTTTTGGGCCATACGTGGTGGTGGAGGAGCAAGCTTTGGAATCATTCTCGCTTGGAAAAT
TAGATTGGTTCCTGTCCCATCAAAGGTTACTATATTCACTGTTAATAAGAACTTGGAGATAAATGAAACCGTG
AAGCTTGTTAACAAGTGGCAAAATATTGCTCACAAGTTTGACAAAGATTTGTTAATCTTTGTTAGGTTCATAA
CTATGAATTCTACTGATGAACAAGGGAAGAAT---------
AAGACAACAATACAAGCTTCATTCTCTTCTATTTTCCTTGGTGGAGTGGATAGTCTACTTGCCTTGATGGAAA
AGAGCTTTCCTGAGTTGGGTGTGAAAAGAAAAGATTGCATCGAAATGAGCTGGATTGAAACTATCTTTTACTT
CAACGGATTCTCA------------AGTGGG---GATAAATTGGAAGTTTTGCTTGATAGAACAACTGAA---
CAAAAGGGTTTT------
TTCAAGGCGAAACTAGACTACGTTAAGAAGCCAATTCCAGAAACTGTAATGGTCAAAATTTTGGAGAAGTTAT
ATGAAGAAGATGTAGGAGTGGGATTGATTCAATTGTACCCTTATGGTGGTAAAATGGACGAGATTCCTGAATC
AGCAATTCCATTCCCTCACCGAGCTGGAATCATGTACAAAATTTTGTACTTGTCTCAATGG---
GAGAAAGAAGAAGAT---GGTGAA------------------
AGGCATATGAATTGGGTTCGAAGTGTTTACAATTACATGACTCCCTATGTGTCCAAAAATCCAAGAGCTGCAT
ATCTCAATTATAGGGACCTTGATTTGGGAACAAATAACGACAAGGGTCCTACTAGTTATGCACAAGCAAGTAT
TTGGGGAAAAAAGTATTTCGGTAAA---
AACTTTAAGAGGTTAGTTCATGTGAAAACCAAGGTTGATCCCACTAATTTCTTCAGGAACGAACAAAGCATCC
CACCTCTT---------CCG---CGACGT------CTCTAA

>HCa_PAML_Nucleotide_Raw
ATGAAGTACTACTCAACAGTATTCATACCTTCTTCTTTTTGGTTTCTTTGCAAATACTCTATATTATTAGTTT
ATATTTCTCTTCTCTATTCATTCTCTATCCAAACTTCTCAAGCTAATTCTCCTCACCGTGATCATGAAAACTT
CCTTCAATGCTTCTCCCAACATATCTCCAACAATAACAATACAACAACCCTTGCCAAACTCATATACACTCCA
AACGACCCGTCGTATATCTCTGTCCTAAATTCGACCATACAAAACCTTAGATTCTCTTCTCCTTCAACCCCAA
AACCACTAGTTATCATCACACCTTCAAATGCCTCCCATGTCCAAGCCTCTGTTTTATGCTCCAAGAAATATGG
CTTGCAGATTCGAACTCGAAGCGGCGGCCATGACTTTGAGGGTGTCTCCTACGTGTCTGAATTAGTCCCATTT
GTCATAGTAGACATGAGAAACCTACGTTCAATCACTGTAGACGTAGATAACAAAACTGCATGGGTTGAAGCTG
GAGCTACCCTTGGAGAAGTTTATTATAGGATTGCTGAGAAAAATGAGAATCTCAGTTTTCCTGCTGGCTATTG
CCATACTGTTGGCGTAGGTGGGCACTTCAGTGGAGGAGGCTATGGAGCATTGATGCGAAAATATGGCCTTGCA

GCTGATAATATCATTGATGCTCACTTAGTCAACGTTGATGGAAAAGTTCTTGACCGAAAATCTATGGGGGAAG
ATCTGTTTTGGGCCATACGTGGTGGTGGAGGAGCAAGCTTTGGAATCATTCTTGCTTGGAAAATTAGATTGGT
TCCTGTCCCATCAAAGGTTACTATATTCACTGTTAATAAGAACTTGGAGATAAATGAAACCGTGAAGCTTGTT
AACAAGTGGCAAAATATTGCTCACAAGTTTGACAAAGATTTGTTAATCTTTGTTAGGTTCATAACTATGAATT
CTACTGATGAACAAGGGAAGAATAGCAAGGAAAAGACAACAATACAAGCTTCATTCTCTTCTATTTTCCTTGG
TGGAGTGGATAGTCTACTTGCCTTGATGGAAAAGAGCTTTCCTGAGTTGGGTGTGAAAAGAAAAGATTGCATC
GAAATGAGCTGGATTGAAACTATCTTTTACTTCAACGGATTCTCAAATTACAACACTAGTGGGGCTGATAAAT
TGGAAGTTTTGCTTGATAGAACAACTGAATTACAAAAGGGTTTTTTACCTTTCAAGGCGAAACTAGACTACGT
TAAGAAGCCAATTCCAGAAACTGTAATGGTCAAAATTTTGGAGAAGTTATATGAAGAAGATGTAGGAGTGGGA
TTGATTCAATTGTACCCTTATGGTGGTAAAATGGACGAGATTCCTGAATCAGCAATTCCATTCCCTCACCGAG
CTGGAATCATGTACAAAATTTTGTACTTGTCTCAATGGGATGAGAAAGAAGAAGAAATTAGTGAAAAAGCAGC
TGAAATTATTAGGCATATGAATTGGGTTCGAAGTGTTTACAATTACATGACTCCCTATGTGTCCAAAAAATCCA
AGAGCTGCATATCTCAATTATAGGGACCTTGATTTGGGAACAAATAACGAAAAGGGTCCTACTAGTTATGCAC
AAGCAAGTATTTGGGGAAAAAAGTATTTCGGTAAAAACAACTTTAAGAGGTTAGTTCATGTGAAAACCAAGGT
TGATCCCACTAATTTCTTCAGGAACGAACAAAGCATCCCACCTCTTCTGTCATTTCCGTTGCGACGTGTCAAG
CTCTAAGGTAATTAATATGGACCTTGATATGAGCTTTCTTATTATTATACTAGTTTCTGGACACGTAACTCCC
ATTTGGCCATATGTAGGGCCATATACAATAAGTTGAAGTGTTTAATGTAA

>HCa_PAML_Nucleotide_Gap
ATGAAG---TACTCAACA---TTC---------TCTTTTTGGTTTCTTTGCAAA------ATATTA---
GTTTAT---TCTCTTCTC---TCATTCTCTATCCAAACTTCTCAAGCTAAT---CCTCAC---------
GAAAACTTCCTTCAATGCTTCTCCCAACATATCTCC------AACAAT---
ACAACCCTTGCCAAACTCATATACACTCCAAACGACCCGTCGTATATCTCTGTCCTAAATTCGACCATACAAA
ACCTTAGATTCTCTTCTCCTTCAACCCCAAAACCACTAGTTATCATCACACCTTCAAATGCCTCCCATGTCCA
AGCCTCTGTTTTATGCTCCAAGAAATATGGCTTGCAGATTCGAACTCGAAGCGGCGGCCATGACTTTGAGGGT
GTCTCCTACGTGTCTGAA---
GTCCCATTTGTCATAGTAGACATGAGAAACCTACGTTCAATCACTGTAGACGTAGATAACAAAACTGCATGGG
TTGAAGCTGGAGCTACCCTTGGAGAAGTTTATTATAGGATTGCTGAGAAAAATGAGAATCTCAGTTTTCCTGC
TGGCTATTGCCATACTGTTGGCGTAGGTGGGCACTTCAGTGGAGGAGGCTATGGAGCATTGATGCGAAAATAT
GGCCTTGCAGCTGATAATATCATTGATGCTCACTTAGTCAACGTTGATGGAAAAGTTCTTGACCGAAAATCTA
TGGGGGAAGATCTGTTTTGGGCCATACGTGGTGGTGGAGGAGCAAGCTTTGGAATCATTCTTGCTTGGAAAAT
TAGATTGGTTCCTGTCCCATCAAAGGTTACTATATTCACTGTTAATAAGAACTTGGAGATAAATGAAACCGTG
AAGCTTGTTAACAAGTGGCAAAATATTGCTCACAAGTTTGACAAAGATTTGTTAATCTTTGTTAGGTTCATAA
CTATGAATTCTACTGATGAACAAGGGAAGAAT---------
AAGACAACAATACAAGCTTCATTCTCTTCTATTTTCCTTGGTGGAGTGGATAGTCTACTTGCCTTGATGGAAA
AGAGCTTTCCTGAGTTGGGTGTGAAAAGAAAAGATTGCATCGAAATGAGCTGGATTGAAACTATCTTTTACTT
CAACGGATTCTCA------------AGTGGG---GATAAATTGGAAGTTTTGCTTGATAGAACAACTGAA---
CAAAAGGGTTTT------
TTCAAGGCGAAACTAGACTACGTTAAGAAGCCAATTCCAGAAACTGTAATGGTCAAAATTTTGGAGAAGTTAT
ATGAAGAAGATGTAGGAGTGGGATTGATTCAATTGTACCCTTATGGTGGTAAAATGGACGAGATTCCTGAATC
AGCAATTCCATTCCCTCACCGAGCTGGAATCATGTACAAAATTTTGTACTTGTCTCAATGG---
GAGAAAGAAGAAGAA---AGTGAA------------------
AGGCATATGAATTGGGTTCGAAGTGTTTACAATTACATGACTCCCTATGTGTCCAAAAAATCCAAGAGCTGCAT
ATCTCAATTATAGGGACCTTGATTTGGGAACAAATAACGAAAAGGGTCCTACTAGTTATGCACAAGCAAGTAT
TTGGGGAAAAAAGTATTTCGGTAAA---
AACTTTAAGAGGTTAGTTCATGTGAAAACCAAGGTTGATCCCACTAATTTCTTCAGGAACGAACAAAGCATCC
CACCTCTT---------CCG---CGACGT------CTCTAA

>HCa_PAML_Codon_Raw
ATGAAGTACTACAAAACAGTATTCATACCTTCTTCTTTTTGGTTTCTTTGCAAATACTCTATATTATTAGTTT
ATATTTCTCTTCTCTATTCATTCTCTATCCAAACTTCTCAAGCTAATTCTCCTCACCGTGATCATGAGAACTT
CCTTCAATGCTTCTCCCAACATATCTCCAACAATAACAATACAACAACCCTTGCCAAACTCATATACACTCCA
AACGACCCGTCGTATATCTCTGTCCTAAATTCGACCATACAAAACCTTAGATTCTCTTCTCCTTCAACCCCAA
AACCACTCGTTATCGTCACACCTTCAAATGCCTCCCATGTCCAAGCCTCTGTTTTATGCTCCAAGAAATATGG
CTTGAAAATTCGAACTAAAAGCGGTGGCCATGACTTTGAGGGTGTCTCCTACGTGTCTGAATTAGTCCCATTT
GTCATAGTAGACATGAGAAACCTACGTAAAATCACTGTAGACGTAGATAACAAAACTGCATGGGTTGAAGCCG
GAGCTACCCTTGGAGAAGTTTATTATAGGATTGCTGAGAAAAATGAGAATCTCAGTTTTCCTGCTGGCTATTG
CCCTACTGTTGGCGTAGGTGGGCACTTCAGTGGAGGAGGCTATGGAGCATTGATGAAAAAATATGGCCTTGCA

GCTGATAATATCATTGATGCTCACTTAGTCAACGTTGATGGAAAAGTTCTTGACCGAAAATCTATGGGGGAAG
ATCTGTTTTGGGCCATACGTGGTGGTGGAGGAGCAAGCTTTGGAATCATTCTCGCTTGGAAAATTAGATTGGT
TCCTGTCCCATCAAAGGTTACTATATTCACTGTTAATAAGAACTTGGAGATAAATGAAACCGTGAAGAAAGTT
AACAAGAAACAAAATATTGCTCACAAGTTTGACAAAGATAAATTAATCTTTGTTAGGTTCATAACTATGAATT
CTACTGATGAACAAGGGAAGAATAGCAAGGAAAAGACAACAATACAAGCTTCATTCTTTTCTATTTTCCTTGG
TGGAGTGGATAGTCTACTTGCTTTGATGGAAAAGAGCTTTCCTGAGTTGGGTGTGAAAAGAAAAGATTGCATC
GAAATGAGCAAAATTGAAACTATCTTTTACTTCAATAAATTCTCAAATTACAACATTAGTGGGGCTGATAAAT
TGGAAGTTTTGCTTAAAAGAACAACTGAATTACAAAAGGGTTTTTTACCTTTCAAGGCGAAACTAGACAAAGT
TAAGAAGCCAATTCCAGAAACTGTAATGGTCAAAATTTTGGAGAAGTTATATGAAGAAGATGTAGGAGTGGGA
TTGAAACAATTGTACCCTTATGGTGGTAAAATGGATGAGATTCCTGAAAAAGCAATTCCATTCCCTCACAAAG
CTGGAATCATGTACAAAATTAAATACTTGTCTCAAAAAGATGAGAAAGAAGAAGATATTAGTGAAAAAGCAGC
TGAAATTATTAGGCATATGAATTGGGTTAAAAGTGTTTACAATTACATGACTCCTTATGTGTCCAAAAATCCA
AGAGCTGCATATCTCAATTATAGGGACCTTGATTTGGGAACAAATAACGAAAAGGGTCCTACTAGTTATGCAC
AAGCAAGCATTTGGGGAAAAAAGTATTTCGGTAAAAACAACTTTAAGAGGTTAGTTCATGTGAAAACCAAGGT
TGATCCCACTAATTTCTTCAGGAACGAACAAAGCATCCCACCTCTTATGTCATTTCCATTGCGACGTCTTAAG
CTCAAAAAAAATAAATATGGACCTAAATATGAGCTTTCTTATTATTATACTAGTTTCTGGACACGTAACTCCC
ATTTGGCCATATGTAGGGCCATATACAATAAGTTGAAGTGTTTAATGAAA

>HCA_PAML_Codon_Gap
ATGAAG---TACAAAACA---TTC---------TCTTTTTGGTTTCTTTGCAAA------ATATTA---
GTTTAT---TCTCTTCTC---TCATTCTCTATCCAAACTTCTCAAGCTAAT---CCTCAC---------
GAGAACTTCCTTCAATGCTTCTCCCAACATATCTCC------AACAAT---
ACAACCCTTGCCAAACTCATATACACTCCAAACGACCCGTCGTATATCTCTGTCCTAAATTCGACCATACAAA
ACCTTAGATTCTCTTCTCCTTCAACCCCAAAACCACTCGTTATCGTCACACCTTCAAATGCCTCCCATGTCCA
AGCCTCTGTTTTATGCTCCAAGAAATATGGCTTGAAAATTCGAACTAAAAGCGGTGGCCATGACTTTGAGGGT
GTCTCCTACGTGTCTGAA---
GTCCCATTTGTCATAGTAGACATGAGAAACCTACGTAAAATCACTGTAGACGTAGATAACAAAACTGCATGGG
TTGAAGCCGGAGCTACCCTTGGAGAAGTTTATTATAGGATTGCTGAGAAAAATGAGAATCTCAGTTTTCCTGC
TGGCTATTGCCCTACTGTTGGCGTAGGTGGGCACTTCAGTGGAGGAGGCTATGGAGCATTGATGAAAAAATAT
GGCCTTGCAGCTGATAATATCATTGATGCTCACTTAGTCAACGTTGATGGAAAAGTTCTTGACCGAAAATCTA
TGGGGGAAGATCTGTTTTGGGCCATACGTGGTGGTGGAGGAGCAAGCTTTGGAATCATTCTCGCTTGGAAAAT
TAGATTGGTTCCTGTCCCATCAAAGGTTACTATATTCACTGTTAATAAGAACTTGGAGATAAATGAAACCGTG
AAGAAAGTTAACAAGAAACAAAATATTGCTCACAAGTTTGACAAAGATAAATTAATCTTTGTTAGGTTCATAA
CTATGAATTCTACTGATGAACAAGGGAAGAAT---------
AAGACAACAATACAAGCTTCATTCTTTTCTATTTTCCTTGGTGGAGTGGATAGTCTACTTGCTTTGATGGAAA
AGAGCTTTCCTGAGTTGGGTGTGAAAAGAAAAGATTGCATCGAAATGAGCAAAATTGAAACTATCTTTTACTT
CAATAAATTCTCA------------AGTGGG---GATAAATTGGAAGTTTTGCTTAAAAGAACAACTGAA---
CAAAAGGGTTTT------
TTCAAGGCGAAACTAGACAAAGTTAAGAAGCCAATTCCAGAAACTGTAATGGTCAAAATTTTGGAGAAGTTAT
ATGAAGAAGATGTAGGAGTGGGATTGAAACAATTGTACCCTTATGGTGGTAAAATGGATGAGATTCCTGAAAA
AGCAATTCCATTCCCTCACAAAGCTGGAATCATGTACAAAATTAAATACTTGTCTCAAAAA---
GAGAAAGAAGAAGAT---AGTGAA------------------
AGGCATATGAATTGGGTTAAAAGTGTTTACAATTACATGACTCCTTATGTGTCCAAAAATCCAAGAGCTGCAT
ATCTCAATTATAGGGACCTTGATTTGGGAACAAATAACGAAAAGGGTCCTACTAGTTATGCACAAGCAAGCAT
TTGGGGAAAAAAGTATTTCGGTAAA---
AACTTTAAGAGGTTAGTTCATGTGAAAACCAAGGTTGATCCCACTAATTTCTTCAGGAACGAACAAAGCATCC
CACCTCTT---------CCA---CGACGT------CTCAAA

>HCa_PAML_AminoAcid_Raw
MKYYSTVFIPSSFWFLCKYSILLVYISLLYSFSIQTSQANSPHREHEDFLQCFSQHISNNNNTTTLAKLIYTP
NDPSYISVLNSTIQNLRFSSPSTPKPLVIITPSNTSHVQASVLCSKKYGLQIRTRSGGHDFEGVSYVSELVPF
VIVDLRNLRSITVDVDNKTAWVEAGATLGEVYYRIAEKNENLSFPAGYCPTVGVGGHFSGGGYGALMRKYGLA
ADNIIDAHLVNVDGKVLDRKSMGEDLFWAIRGGGGASFGIILAWKIRLVPVPSKVTIFSVNKNLEINETVKLV
NKWQNIAHKFDKDLLIFVRFMTVNSTDGQGKNSKEKTTIQASFFSIFLGGVDSLLSLMEKSFPELGVKRKDCF
EMSWIETIFYFNGFSNYNLSGADKLEVLLDRTSQSQKGFLPFKAKLDYVKKPIPETVMVKLLEKLYEEDVGLG
LIQMYPYGGKMDEIPESAIPFPHRAGFMYKILYLSQWEEKEEDISEKAAEIIRHLNWVRSVYNYMTPYVSKNP
RAAYLNYRDLDLGKNNEKGPTSYAQASIWGKKYFGKNNFKRLVHVKTKVDPTNFFRNEQSIPPLLSFPLRRVK
LKGNNYGPGYELSYYYTSFWTRNSHLAICRAIYNKLKCLMLL

```
>HCa_PAML_AminoAcid_Gap
MK-YST-F---SFWFLCK--IL-VY-SLL-SFSIQTSQAN-PH---EDFLQCFSQHIS--NN-
TTLAKLIYTPNDPSYISVLNSTIQNLRFSSPSTPKPLVIITPSNTSHVQASVLCSKKYGLQIRTRSGGHDFEG
VSYVSE-
VPFVIVDLRNLRSITVDVDNKTAWVEAGATLGEVYYRIAEKNENLSFPAGYCPTVGVGGHFSGGGYGALMRKY
GLAADNIIDAHLVNVDGKVLDRKSMGEDLFWAIRGGGGASFGIILAWKIRLVPVPSKVTIFSVNKNLEINETV
KLVNKWQNIAHKFDKDLLIFVRFMTVNSTDGQGKN---
KTTIQASFFSIFLGGVDSLLSLMEKSFPELGVKRKDCFEMSWIETIFYFNGFS----SG-DKLEVLLDRTSQ-
QKGF--
FKAKLDYVKKPIPETVMVKLLEKLYEEDVGLGLIQMYPYGGKMDEIPESAIPFPHRAGFMYKILYLSQW-
EKEED-SE------RHLNWVRSVYNYMTPYVSKNPRAAYLNYRDLDLGKNNEKGPTSYAQASIWGKKYFGK-
NFKRLVHVKTKVDPTNFFRNEQSIPPL---P-RR--
```
