# Supplementary material for: Resurrected Ancestral Cannabis Enzymes Unveil the Origin and Functional Evolution of Cannabinoid Synthases
Source: Plant Biotechnol J. 2025 Dec 26;24(4):2685–97. doi: 10.1111/pbi.70475 (PMC13140220; doi:10.1111/pbi.70475)
Supplement: Supplementary file 1 — Figure S1: Phylogeny of Cannabaceae‐specific Berberine Bridge‐Like genes. Figure S2: Syntenic blocks comprising cannabinoid synthase genes and closely‐related BBLs. Figure S3: Evaluation of enzyme expression by immunodetection. Figure S4: Determination of the optimal pH and reactional temperature for the activity of Ca. Figure S5: Determination of the optimal pH for the activity of HCa → CaSBR and Ca → CBDASSBR_FAD. Figure S6: Design and structure of the THCAS → CBDAS hybrid. Table S1: Analysis of the reconstructed ancestral sequences. Table S2: Design of the HCa → Ca (a), Ca → CBDAS (b) and Ca → A1A2a (c) hybrids, based on sequence and structural comparison. Table S3: Expression level of candidate enzymes (μg mL−1). Table S4: Comparison of the mutations tested in previous studies with mutations included in our hybrids. Table S5: Quality assessment of the three‐dimensional (3D) enzyme homology models. Data S1: Sequence alignment used to generate the gene‐tree and reconstruct the ancestors. Data S2: Ancestral sequences reconstructed with MrBayes and PAML. Data S3: Sequences of A1A1a, Ca and HCa. Data S4: Domesticated sequences used to express and characterise enzymes. Data S5: Berberine Bridge‐Like dataset. Data S6: Ancestral sequence reconstruction with MrBayes. Data S7: Ancestral sequence reconstruction with PAML. [file PBI-24-2685-s001.zip › pbi70475-sup-0007-DataS3.pdf]

>HCa_corrected
ATGAAGTACTCAACATTCTCTTTTTGGTTTCTTTGCAAAATATTAGTTTATTCTCTTCTCTCATTCTCTATCC
AAACTTCTCAAGCTAATCCTCACGAAAACTTCCTTCAATGCTTCTCCCAACATATCTCCAACAATACAACCCT
TGCCAAACTCATATACACTCCAAACGACCCGTCGTATATCTCTGTCCTAAATTCGACCATACAAAACCTTAGA
TTCTCTTCTCCTTCAACCCCAAAACCACTCGTTATCGTCACACCTTCAAATGCCTCCCATGTCCAAGCCTCTG
TTTTATGCTCCAAGAAATATGGCTTGCAGATTCGAACTCGAAGCGGCGGCCATGACTTTGAGGGTGTCTCCTA
CGTGTCTGAAGTCCCATTTGTCATAGTAGACATGAGAAACCTACGTTCGATCACTGTAGACGTAGATAACAAA
ACTGCATGGGTTGAAGCTGGAGCTACCCTTGGAGAAGTTTATTATAGGATTGCTGAGAAAAATGAGAATCTCA
GTTTTCCTGCTGGCTATTGCCCTACTGTTGGCGTAGGTGGGCACTTCAGTGGAGGAGGCTATGGAGCATTGAT
GCGAAAATATGGCCTTGCAGCTGATAATATCATTGATGCTCACTTAGTCAACGTTGATGGAAAAGTTCTTGAC
CGAAAATCTATGGGGGAAGATCTGTTTTGGGCCATACGTGGTGGTGGAGGAGCAAGCTTTGGAATCATTCTCG
CTTGGAAAATTAGATTGGTTCCTGTCCCATCAAAGGTTACTATATTCACTGTTAATAAGAACTTGGAGATAAA
TGAAACCGTGAAGCTTGTTAACAAGTGGCAAAATATTGCTCACAAGTTTGACAAAGATTTGTTAATCTTTGTT
AGGTTCATAACTATGAATTCTACTGATGAACAAGGGAAGAATAAGACAACAATACAAGCTTCATTCTCTTCTA
TTTTCCTTGGTGGAGTGGATAGTCTACTTGCCTTGATGGAAAAGAGCTTTCCTGAGTTGGGTGTGAAAAGAAA
AGATTGCATCGAAATGAGCTGGATTGAAACTATCTTTTACTTCAACGGATTCTCAAGTGGGGATAAATTGGAA
GTTTTGCTTGATAGAACAACTGAACAAAAGGGTTTTTTCAAGGCGAAACTAGACTACGTTAAGAAGCCAATTC
CAGAAACTGTAATGGTCAAAATTTTGGAGAAGTTATATGAAGAAGATGTAGGAGTGGGATTGATTCAATTGTA
CCCTTATGGTGGTAAAATGGACGAGATTCCTGAATCAGCAATTCCATTCCCTCACCGAGCTGGAATCATGTAC
AAAATTTTGTACTTGTCTCAATGGGAGAAAGAAGAAGATGGTGAAAGGCATATGAATTGGGTTCGAAGTGTTT
ACAATTACATGACTCCCTATGTGTCCAAAAATCCAAGAGCTGCATATCTCAATTATAGGGACCTTGATTTGGG
AACAAATAACGACAAGGGTCCTACTAGTTATGCACAAGCAAGTATTTGGGGAAAAAAGTATTTCGGTAAAAAC
TTTAAGAGGTTAGTTCATGTGAAAACCAAGGTTGATCCCACTAATTTCTTCAGGAACGAACAAAGCATCCCAC
CTCTTCCGCGACGTCTCTAA

>Ca_corrected
ATGAAGTACTCAACATTCTCCTTTTGGTTTGTTTGCAAGATAATATTTTTCTTTCTCTCATTCAATATCCAAA
CTTCAATAGCTAATCCTCAAGAAAACTTCCTTAAATGCTTCTCGCAATATATTCCCAACAATGCAACAAATCT
AAAACTCGTATACACTCAAAACGACCAATTGTATATGTCTGTCCTAAATTCGACAATACAAAATCTTAGATTC
ACCTCTGACACAACCCCAAAACCACTTGTTATCGTCACTCCTTCAAATGTCTCCCATATCCAAGGCACTATTC
TATGCTCCAAGAAAGTTGGCTTGCAGATTCGAACTCGAAGCGGTGGTCATGATTCTGAGGGCATGTCCTACAT
ATCTCAAGTCCCATTTGTTATAGTAGACTTGAGAAACATGCATTCGATCAAAATAGATGTTCATAGCCAAACT
GCATGGGTTGAAGCCGGAGCTACCCTTGGAGAAGTTTATTATTGGATTAATGAGAAAAATGAGAATCTTAGTT
TTCCTGCTGGGTATTGCCCTACTGTTGGCGCAGGTGGACACTTTAGTGGAGGAGGCTATGGAGCATTGATGCG
AAATTATGGCCTCGCGGCTGATAATATCATTGATGCACACTTAGTCAACGTTGATGGAAAAGTTCTAGATCGA
AAATCCATGGGGGAAGATCTGTTTTGGGCTATACGTGGTGGTGGAGGAGAAAGCTTTGGAATCATTGTAGCGT
GGAAAATTAGACTGGTTGCTGTCCCATCAAAGTCTACTATATTCAGTGTTAAAAAGAACATGGAGATACATGA
GCTTGTCAAGTTAGTTAACAAATGGCAAAATATTGCTTACAAGTATGACAAAGATTTATTACTCATGACTCAC
TTCATAACTAGGAATATTACAGATAATCAAGGGAAGAATAAGACAACAATACACACTTACTTCTCTTCCATTT
TCCTTGGTGGAGTGGATAGTCTAGTCGACTTGATGAACAAGAGCTTTCCTGAGTTGGGTATTAAAAAAACAGA
TTGCAAACAATTGAGCTGGATTGATACTATCATCTTCTACAGTGGTGTTGTAAATTACAACACTGCTAATTTT
AAAAAGGAAATTTTGCTTGATAGATCAGCTGGGCAGAAGGGGGCTTTCAAGATTAAGTTAGACTACGTTAAGA
AACCAATTCCAGAAACTGCAATGGTCAAAATTTTGGAAAAATTATATGAAGAAGATGTAGGAGTTGGGATGTA
TGTGTTGTACCCTTACGGTGGTATAATGGATGAGATTTCAGAATCAGCAATTCCATTCCCTCATCGAGCTGGA
ATCATGTATGAAATTTGGTACATATCTACCTGGGAGAAGCAAGAAGATAATGAAAAGCATATAAACTGGATTC
GAAATGTTTATAATTTCATGACTCCTTATGTGTCCCAAAATCCAAGAATGGCATATCTCAATTATAGGGACCT
TGATTTAGGAAAAAATAATCCCAAGAGTCCTAATAATTACACACAAGCACGTATTTGGGGTGAAAAGTATTTT
GGTAAAAATTTTAACAGGTTAGTAAAAGTGAAAACCAAGGTTGATCCCAATAATTTTTTAGAAACGAACAAA
GCATCCCACCTCTTCCACGGCATCGTCATTAA

>A1A2a_corrected
ATGAATTGCTCAACATTCTCCTTTTGGTTTGTTTGCAAAATAATATTTTTCTTTCTCTCATTCAATATCCAAA
TTTCAATAGCTAATCCTCAAGAAAACTTCCTTAAATGCTTCTCGCAATATATTCCCAACAATGCAGCAAATCC
AAAACTCGTATACACTCAACACGACCAATTGTATATGTCTGTCCTGAATTCGACAATACAAAATCTTAGATTC
ACCTCTGATACAACCCCAAAACCACTCGTTATTGTCACTCCTTCAAATGTCTCCCATATCCAAGCCACTATTC
TATGCTCCAAGAAAGTTGGCTTGCAGATTCGAACTCGAAGCGGTGGCCATGATGCTGAGGGTTTGTCCTACAT
ATCTCAAGTCCCATTTGTTATAGTAGACTTGAGAAACATGCATTCGGTCAAAATAGATGTTCATAGCCAAACT
GCGTGGGTTGAAGCCGGAGCTACCCTTGGAGAAGTTTATTATTGGATCAATGAGAAGAATGAGAATCTTAGTT

TTCCTGGTGGGTATTGCCCTACTGTTGGCGCAGGTGGACACTTTAGTGGAGGAGGCTATGGAGCATTGATGCG
AAATTATGGCCTCGCGGCTGATAATATCATTGATGCACACTTAGTCAATGTTGATGGAAAAGTTCTAGATCGA
AAATCCATGGGAGAAGATCTGTTTTGGGCTATACGTGGTGGTGGAGGAGAAAACTTTGGAATCATTGCAGCAT
GGAAAATCAAACTGGTTGCTGTCCCATCAAAGTCTACTATATTCAGTGTTAAAAAGAACATGGAGATACATGG
GCTTGTCAAGTTATTTAACAAATGGCAAAATATTGCTTACAAGTATGACAAAGATTTATTACTCATGACTCAC
TTCATAACCAGGAATATTACAGATAATCATGGGAAGAATAAGACTACAGTACATGGTTACTTCTCTTCCATTT
TTCATGGTGGAGTGGATAGTCTAGTCGACTTGATGAACAAGAGCTTTCCTGAGTTGGGTATTAAAAAAACTGA
TTGCAAAGAATTGAGCTGGATTGATACAACCATCTTCTACAGTGGTGTTGTAAATTACAACACTGCTAATTTT
AAAAAGGAAATTTTGCTTGATAGATCAGCTGGGCAGAAGACGGCTTTCTCAATTAAGTTAGACTATGTTAAGA
AACCAATTCCAGAAACTGCAATGGTCAAAATTTTGGAAAAATTATATGAAGAAGATGTAGGAGTTGGGATGTA
TGTGTTGTACCCTTACGGTGGTATAATGGATGAGATTTCAGAATCAGCAATTCCATTCCCTCATCGAGCTGGA
ATCATGTATGAACTTTGGTACACAGCTACCTGGGAGAAGCAAGAAGATAATGAAAAGCATATAAACTGGGTTC
GAAGTGTTTATAATTTCACGACTCCTTATGTGTCCCAAAATCCAAGAATGGCGTATCTCAATTATAGGGACCT
TGATTTAGGAAAAACTAATCCTGAGAGTCCTAATAATTACACACAAGCACGTATTTGGGGTGAAAAGTATTTT
GGTAAAAATTTTAACAGGTTAGTTAAGGTGAAAACCAAAGTTGATCCCAATAATTTTTTAGAAACGAACAAA
GTATCCCACCTCTTCCACCGCGTCATCATTAA
