# Supplementary material for: Resurrected Ancestral Cannabis Enzymes Unveil the Origin and Functional Evolution of Cannabinoid Synthases
Source: Plant Biotechnol J. 2025 Dec 26;24(4):2685–97. doi: 10.1111/pbi.70475 (PMC13140220; doi:10.1111/pbi.70475)
Supplement: Supplementary file 1 — Figure S1: Phylogeny of Cannabaceae‐specific Berberine Bridge‐Like genes. Figure S2: Syntenic blocks comprising cannabinoid synthase genes and closely‐related BBLs. Figure S3: Evaluation of enzyme expression by immunodetection. Figure S4: Determination of the optimal pH and reactional temperature for the activity of Ca. Figure S5: Determination of the optimal pH for the activity of HCa → CaSBR and Ca → CBDASSBR_FAD. Figure S6: Design and structure of the THCAS → CBDAS hybrid. Table S1: Analysis of the reconstructed ancestral sequences. Table S2: Design of the HCa → Ca (a), Ca → CBDAS (b) and Ca → A1A2a (c) hybrids, based on sequence and structural comparison. Table S3: Expression level of candidate enzymes (μg mL−1). Table S4: Comparison of the mutations tested in previous studies with mutations included in our hybrids. Table S5: Quality assessment of the three‐dimensional (3D) enzyme homology models. Data S1: Sequence alignment used to generate the gene‐tree and reconstruct the ancestors. Data S2: Ancestral sequences reconstructed with MrBayes and PAML. Data S3: Sequences of A1A1a, Ca and HCa. Data S4: Domesticated sequences used to express and characterise enzymes. Data S5: Berberine Bridge‐Like dataset. Data S6: Ancestral sequence reconstruction with MrBayes. Data S7: Ancestral sequence reconstruction with PAML. [file PBI-24-2685-s001.zip › pbi70475-sup-0008-DataS4.pdf]

```
>THCAS_domesticated
GAATTCAATCCTCGAGAAAACTTCCTTAAATGCTTCTCAAAACATATTCCCAACAATGTAGCAAATCCAAAAC
TCGTATACACTCAACACGACCAATTGTATATGTCTATCCTTAATTCGACAATACAAAATCTTAGATTCATCTC
TGATACAACCCCAAAACCACTCGTTATTGTCACTCCTTCAAATAACTCCCATATCCAAGCAACTATTTTATGC
TCTAAGAAAGTTGGCTTGCAGATTCGAACTCGAAGCGGTGGCCATGATGCTGAGGGTATGTCCTACATATCTC
AAGTCCCATTTGTTGTAGTAGACTTGAGAAACATGCATTCGATCAAAATAGATGTTCATAGCCAAACTGCGTG
GGTTGAAGCCGGAGCTACCCTTGGAGAAGTTTATTATTGGATCAATGAGAAGAATGAGAATCTTAGTTTTCCT
GGTGGGTATTGCCCTACTGTTGGCGTAGGTGGACACTTTAGTGGAGGAGGCTATGGAGCATTGATGCGAAATT
ATGGCCTTGCGGCTGATAATATTATTGATGCACACTTAGTCAATGTTGATGGAAAAGTTCTGGATCGAAAATC
CATGGGAGAAGATCTGTTTTGGGCTATACGTGGTGGTGGAGGAGAAAACTTTGGAATCATTGCAGCATGGAAA
ATCAAACTGGTTGCTGTCCCATCAAAGTCTACTATATTCAGTGTTAAAAAGAACATGGAGATACATGGGCTTG
TCAAGTTATTTAACAAATGGCAAAATATTGCTTACAAGTATGACAAAGATTTAGTACTCATGACTCACTTCAT
AACAAAGAATATTACAGATAATCATGGGAAGAATAAGACTACAGTACATGGTTACTTCTCTTCAATTTTTCAT
GGTGGAGTGGATAGTCTAGTCGACTTGATGAACAAGAGCTTTCCTGAGTTGGGTATTAAAAAAACTGATTGCA
AAGAATTTAGCTGGATTGATACAACCATCTTCTACAGTGGTGTTGTAAATTTTAACACTGCTAATTTTAAAAA
GGAAATTTTGCTTGATAGATCAGCTGGGAAGAAGACGGCTTTCTCAATTAAGTTAGACTATGTTAAGAAACCA
ATTCCAGAAACTGCAATGGTCAAAATTTTGGAAAAATTATATGAAGAAGATGTAGGAGCTGGGATGTATGTGT
TGTACCCTTACGGTGGTATAATGGAGGAGATTTCAGAATCAGCAATTCCATTCCCTCATCGAGCTGGAATAAT
GTATGAACTTTGGTACACTGCTTCCTGGGAGAAGCAAGAAGATAATGAAAAGCATATAAACTGGGTTCGAAGT
GTTTATAATTTTACGACTCCTTATGTGTCCCAAAATCCAAGATTGGCGTATCTCAATTATAGGGACCTTGATT
TAGGAAAAACTAATCATGCGAGTCCTAATAATTACACACAAGCACGTATTTGGGGTGAAAAGTATTTTGGTAA
AAATTTTAACAGGTTAGTTAAGGTGAAAACTAAAGTTGATCCCAATAATTTTTTTAGAAACGAACAAAGTATC
CCACCTCTTCCACCGCATCATCATCATCATCATtaaTCTAGA

>CBDAS_domesticated
GAATTCAATCCTCGAGAAAACTTCCTTAAATGCTTCTCGCAATATATTCCCAATAATGCAACAAATCTAAAAC
TCGTATACACTCAAAACAACCCATTGTATATGTCTGTCCTAAATTCGACAATACACAATCTTAGATTCACCTC
TGACACAACCCCAAAACCACTTGTTATCGTCACTCCTTCACATGTCTCTCATATCCAAGGCACTATTCTATGC
TCCAAGAAAGTTGGCTTGCAGATTCGAACTCGAAGTGGTGGTCATGATTCTGAGGGCATGTCCTACATATCTC
AAGTCCCATTTGTTATAGTAGACTTGAGAAACATGCGTTCAATCAAAATAGATGTTCATAGCCAAACTGCATG
GGTTGAAGCCGGAGCTACCCTTGGAGAAGTTTATTATTGGGTTAATGAGAAAAATGAGAATCTTAGTTTGGCG
GCTGGGTATTGCCCTACTGTTTGCGCAGGTGGACACTTTGGTGGAGGAGGCTATGGACCATTGATGAGAAACT
ATGGCCTCGCGGCTGATAATATCATTGATGCACACTTAGTCAACGTTCATGGAAAAGTGCTAGATCGAAAATC
TATGGGGGAAGATCTCTTTTGGGCTTTACGTGGTGGTGGAGCAGAAAGCTTCGGAATCATTGTAGCATGGAAA
ATTAGACTGGTTGCTGTCCCAAAGTCTACTATGTTTAGTGTTAAAAAGATCATGGAGATACATGAGCTTGTCA
AGTTAGTTAACAAATGGCAAAATATTGCTTACAAGTATGACAAAGATTTATTACTCATGACTCACTTCATAAC
TAGGAACATTACAGATAATCAAGGGAAGAATAAGACAGCAATACACACTTACTTCTCTTCAGTTTTCCTTGGT
GGAGTGGATAGTCTAGTCGACTTGATGAACAAGAGTTTTCCTGAGTTGGGTATTAAAAAAACGGATTGCAGAC
AATTGAGCTGGATTGATACTATCATCTTCTATAGTGGTGTTGTAAATTACGACACTGATAATTTTAACAAGGA
AATTTTGCTTGATAGATCCGCTGGGCAGAACGGTGCTTTCAAGATTAAGTTAGACTACGTTAAGAAACCAATT
CCAGAATCTGTATTTGTCCAAATTTTGGAAAAATTATATGAAGAAGATATAGGAGCTGGGATGTATGCGTTGT
ACCCTTACGGTGGTATAATGGATGAGATTTCAGAATCAGCAATTCCATTCCCTCATCGAGCTGGAATCTTGTA
TGAGTTATGGTACATATGTAGTTGGGAGAAGCAAGAAGATAACGAAAAGCATCTAAACTGGATTAGAAATATT
TATAACTTCATGACTCCTTATGTGTCCAAAAATCCAAGATTGGCATATCTCAATTATAGAGACCTTGATATAG
GAATAAATGATCCCAAGAATCCAAATAATTACACACAAGCACGTATTTGGGGTGAGAAGTATTTTGGTAAAAA
TTTTGACAGGCTAGTAAAAGTGAAAACCCTGGTTGATCCCAATAACTTTTTTAGAAACGAACAAAGCATCCCA
CCTCTTCCACGGCATCGTCATCATCATCATCATCATtaaTCTAGA

>Hop-BBL_domesticated
GAATTCGATCCTCACGCTGATTTCCTTCAATGCTTCTCCCAATATATCTCAAACAGTACAACCATTGCCAAAC
TCATCTACACTCCAAATGATCCGTTGTATATCTCCATCCTAAACTCGACTATACAAAACAACAGATTTTCTTC
TCCTTCAACCCCTAAACCACTGATTATCATTACACCATTAAATTCCTTCCATGTACAAGCCTCCATTCTATGC
TCCAGGAAATATGGCTTGCAAATTCGAACCCGAAGCGGCGGCCATGACTTTGAGGGTGTCTCCTATGTGTCTG
AAGTCCCATTTGTCATAGTAGACATGAGAAACCTACGTTCCATCACCATTGACGTAGATAACAAAACTGCATG
GGTTGATGTTGGAGCTACCCTTGGAGAACTGTATTATAGGATTGCTGAGAAAAATGAGAATCTCAGTTTTCCT
GCTGGCTATTGCCATACCGTTGGCGTAGGTGGGCACTTCAGTGGAGGAGGCTATGGAGCATTGATGCGAAAAT
ATGGGCTCGCAGCTGATAATGTCATTGATGCTCACTTAGTCAATGTTGATGGAGAAGTTCTTGACCGACAATC
CATGGGGGAAGATCTGTTTTGGGCCATACGTGGTGGTGGAGGAGCAAGCTTTGGAATCATTCTTGCTTGGAAA
```

ATTAGATTGGTTCCTGTCCCATCAAAGGTTACTATAGTCTCCATTAATAAGAACTTGGAGATAAATGAAACCG
TGAAGCTTTACAACAAGTGGCAAAATATTGCTCACAAGTTTGACAAAGATTTATTAATCTTTGTAAGGTTCAC
AACTATGAACTCTACTGATGGACAAGGGAAGAACAAGACGGCAATACTAACTTCATTCTACTCTATTTTCTTT
GGTGGAATGGATGGTTTACTTGCTTTGATGGAAAAGAGCTTTCCTGAGTTGGATGTGAAAAGAAAAGATTGTT
TCGAAGCAAGCTGGATCGAAATGATCTTTTACTTCAACGGATTCTCAAGTGGGGATAAATTGGAAGTCTTGCT
TGGTAGAACAAATGAAGAAAAGGGTTTTTTCAAGGCAAAACTAGACTACGTTAGGAAGCCGATACCAGAAACA
GTAATAGTCAAACTTTTGGAGAAGTTATATAATGAAGATGTAGGATTGGGATTGATTCAAATGTACCCGTATG
GTGGTAAAATGGACGAGATTCCTGAATCTGCAATTCCATTCCCTCACCGAGTTGGATTCATTTACAAAATTTT
GTACTTGTCTCAATGGGAGAAAGAAGAAGAAGGTGAAAGACATCTGAACTGGGTTCGGAGTGTTTACAATTAC
ATGACTCCCTTTGTGTCTAAAAGTCCAAGAGCTTCATATCTCAATTATAGGGACTTTGATTTGGGAACAAATA
ACAAAAATGGTCCTACTAGTTATGGACAAGCAAGCATTTGGGGAAAAAAGTATTTCGATAAAAACTTTAAGAG
GTTAGTTCATGTGAAAACGAAGGTTGATCCCACTAATTTCTTCAGGAACGAACAAAGCATCCCACCTCTTTCA
GTGCGAGGTCTCCATCATCATCATCATCATtaaTCTAGA

>HCa_domesticated
GAATTCAATCCTCACGAAAACTTCCTTCAATGCTTCTCCCAACATATCTCCAACAATACAACCCTTGCCAAAC
TCATATACACTCCAAACGACCCGTCGTATATCTCTGTCCTAAATTCGACCATACAAAACCTTAGATTCTCTTC
TCCTTCAACCCCAAAACCACTCGTTATCGTCACACCTTCAAATGCCTCCCATGTCCAAGCCTCTGTTTTATGC
TCCAAGAAATATGGCTTGCAGATTCGAACTCGAAGCGGCGGCCATGACTTTGAGGGTGTCTCCTACGTGTCTG
AAGTCCCATTTGTCATAGTAGACATGAGAAACCTACGTTCGATCACTGTAGACGTAGATAACAAAACTGCATG
GGTTGAAGCTGGAGCTACCCTTGGAGAAGTTTATTATAGGATTGCTGAGAAAAATGAGAATCTCAGTTTTCCT
GCTGGCTATTGCCCTACTGTTGGCGTAGGTGGGCACTTCAGTGGAGGAGGCTATGGAGCATTGATGCGAAAAT
ATGGCCTTGCAGCTGATAATATCATTGATGCTCACTTAGTCAACGTTGATGGAAAAGTTCTTGACCGAAAATC
TATGGGGGAAGATCTGTTTTGGGCCATACGTGGTGGTGGAGGAGCAAGCTTTGGAATCATTCTCGCTTGGAAA
ATTAGATTGGTTCCTGTCCCATCAAAGGTTACTATATTCACTGTTAATAAGAACTTGGAGATAAATGAAACCG
TGAAGCTTGTTAACAAGTGGCAAAATATTGCTCACAAGTTTGACAAAGATTTGTTAATCTTTGTTAGGTTCAT
AACTATGAACTCTACTGATGAACAAGGGAAGAATAAGACAACAATACAAGCTTCATTCTCTTCTATTTTCCTT
GGTGGAGTGGATAGTCTACTTGCCTTGATGGAAAAGAGCTTTCCTGAGTTGGGTGTGAAAAGAAAAGATTGCA
TCGAAATGAGCTGGATTGAAACTATCTTTTACTTCAACGGATTCTCAAGTGGGGATAAATTGGAAGTTTTGCT
TGATAGAACAACTGAACAAAAGGGTTTTTTCAAGGCGAAACTAGACTACGTTAAGAAGCCAATTCCAGAAACT
GTAATGGTCAAAATTTTGGAGAAGTTATATGAAGAAGATGTAGGAGTGGGATTGATTCAATTGTACCCTTATG
GTGGTAAAATGGACGAGATTCCTGAATCAGCAATTCCATTCCCTCACCGAGCTGGAATCATGTACAAAATTTT
GTACTTGTCTCAATGGGAGAAAGAAGAAGATGGTGAAAGGCATATGAATTGGGTTCGAAGTGTTTACAATTAC
ATGACTCCCTATGTGTCCAAAAATCCAAGAGCTGCATATCTCAATTATAGGGACCTTGATTTGGGAACAAATA
ACGACAAGGGTCCTACTAGTTATGCACAAGCAAGTATTTGGGGAAAAAAGTATTTCGGTAAAAACTTTAAGAG
GTTAGTTCATGTGAAAACCAAGGTTGATCCCACTAATTTCTTCAGGAACGAACAAAGCATCCCACCTCTTCCG
CGACGTCTCCATCATCATCATCATCATtaaTCTAGA

>Ca_domesticated
GAATTCAATCCTCAAGAAAACTTCCTTAAATGCTTCTCGCAATATATTCCCAACAATGCAACAAATCTAAAAC
TCGTATACACTCAAAACGACCAATTGTATATGTCTGTCCTAAATTCGACAATACAAAATCTTAGATTCACCTC
TGACACAACCCCAAAACCACTTGTTATCGTCACTCCTTCAAATGTCTCCCATATCCAAGGCACTATTCTATGC
TCCAAGAAAGTTGGCTTGCAGATTCGAACTCGAAGCGGTGGTCATGATTCTGAGGGCATGTCCTACATATCTC
AAGTCCCATTTGTTATAGTAGACTTGAGAAACATGCATTCGATCAAAATAGATGTTCATAGCCAAACTGCATG
GGTTGAAGCCGGAGCTACCCTTGGAGAAGTTTATTATTGGATTAATGAGAAAAATGAGAATCTTAGTTTTCCT
GCTGGGTATTGCCCTACTGTTGGCGCAGGTGGACACTTTAGTGGAGGAGGCTATGGAGCATTGATGCGAAATT
ATGGCCTCGCGGCTGATAATATCATTGATGCACACTTAGTCAACGTTGATGGAAAAGTTCTGGATCGAAAATC
CATGGGGGAAGATCTGTTTTGGGCTATACGTGGTGGTGGAGGAGAAAGCTTTGGAATCATTGTAGCGTGGAAA
ATTAGACTGGTTGCTGTCCCATCAAAGTCTACTATATTCAGTGTTAAAAAGAACATGGAGATACATGAGCTTG
TCAAGTTAGTTAACAAATGGCAAAATATTGCTTACAAGTATGACAAAGATTTATTACTCATGACTCACTTCAT
AACTAGGAATATTACAGATAATCAAGGGAAGAATAAGACAACAATACACACTTACTTCTCTTCCATTTTCCTT
GGTGGAGTGGATAGTCTAGTCGACTTGATGAACAAGAGCTTTCCTGAGTTGGGTATTAAAAAAACAGATTGCA
AACAATTGAGCTGGATTGATACTATCATCTTCTACAGTGGTGTTGTAAATTACAACACTGCTAATTTTAAAAA
GGAAATTTTGCTTGATAGATCAGCTGGGCAGAAGGGGGCTTTCAAGATTAAGTTAGACTACGTTAAGAAACCA
ATTCCAGAAACTGCAATGGTCAAAATTTTGGAAAAATTATATGAAGAAGATGTAGGAGTTGGGATGTATGTGT
TGTACCCTTACGGTGGTATAATGGATGAGATTTCAGAATCAGCAATTCCATTCCCTCATCGAGCTGGAATCAT
GTATGAAATTTGGTACATATCTACCTGGGAGAAGCAAGAAGATAATGAAAAGCATATAAACTGGATTCGAAAT
GTTTATAATTTCATGACTCCTTATGTGTCCCAAAATCCAAGAATGGCATATCTCAATTATAGGACCTTGATT

TAGGAAAAAATAATCCCAAGAGTCCTAATAATTACACACAAGCACGTATTTGGGGTGAAAAGTATTTTGGTAA
AAATTTTAACAGGTTAGTAAAAGTGAAAACCAAGGTTGATCCCAATAATTTTTTTAGAAACGAACAAAGCATC
CCACCTCTTCCACGGCATCGTCATCATCATCATCATCATtaaTCTAGA

>A1A2a_domesticated
GAATTCAATCCTCAAGAAAACTTCCTTAAATGCTTCTCGCAATATATTCCCAACAATGCAGCAAATCCAAAAC
TCGTATACACTCAACACGACCAATTGTATATGTCTGTCCTGAACTCGACAATACAAAATCTTAGATTCACCTC
TGATACAACCCCAAAACCACTCGTTATTGTCACTCCTTCAAATGTCTCCCATATCCAAGCCACTATTCTATGC
TCCAAGAAAGTTGGCTTGCAGATTCGAACTCGAAGCGGTGGCCATGATGCTGAGGGTTTGTCCTACATATCTC
AAGTCCCATTTGTTATAGTAGACTTGAGAAACATGCATTCGGTCAAAATAGATGTTCATAGCCAAACTGCGTG
GGTTGAAGCCGGAGCTACCCTTGGAGAAGTTTATTATTGGATCAATGAGAAGAATGAGAATCTTAGTTTTCCT
GGTGGGTATTGCCCTACTGTTGGCGCAGGTGGACACTTTAGTGGAGGAGGCTATGGAGCATTGATGCGAAATT
ATGGCCTCGCGGCTGATAATATCATTGATGCACACTTAGTCAATGTTGATGGAAAAGTTCTGGATCGAAAATC
CATGGGAGAAGATCTGTTTTGGGCTATACGTGGTGGTGGAGGAGAAAACTTTGGAATCATTGCAGCATGGAAA
ATCAAACTGGTTGCTGTCCCATCAAAGTCTACTATATTCAGTGTTAAAAAGAACATGGAGATACATGGGCTTG
TCAAGTTATTTAACAAATGGCAAAATATTGCTTACAAGTATGACAAAGATTTATTACTCATGACTCACTTCAT
AACCAGGAATATTACAGATAATCATGGGAAGAATAAGACTACAGTACATGGTTACTTCTCTTCCATTTTTCAT
GGTGGAGTGGATAGTCTAGTCGACTTGATGAACAAGAGCTTTCCTGAGTTGGGTATTAAAAAAACTGATTGCA
AAGAATTGAGCTGGATTGATACAACCATCTTCTACAGTGGTGTTGTAAATTACAACACTGCTAATTTTAAAAA
GGAAATTTTGCTTGATAGATCAGCTGGGCAGAAGACGGCTTTCTCAATTAAGTTAGACTATGTTAAGAAACCA
ATTCCAGAAACTGCAATGGTCAAAATTTTGGAAAAATTATATGAAGAAGATGTAGGAGTTGGGATGTATGTGT
TGTACCCTTACGGTGGTATAATGGATGAGATTTCAGAATCAGCAATTCCATTCCCTCATCGAGCTGGAATCAT
GTATGAACTTTGGTACACAGCTACCTGGGAGAAGCAAGAAGATAATGAAAAGCATATAAACTGGGTTCGAAGT
GTTTATAATTTCACGACTCCTTATGTGTCCCAAAATCCAAGAATGGCGTATCTCAATTATAGGGACCTTGATT
TAGGAAAAACTAATCCTGAGAGTCCTAATAATTACACACAAGCACGTATTTGGGGTGAAAAGTATTTTGGTAA
AAATTTTAACAGGTTAGTTAAGGTGAAAACCAAAGTTGATCCCAATAATTTTTTTAGAAACGAACAAAGTATC
CCACCTCTTCCACCGCGTCATCATCATCATCATCATtaaTCTAGA

>HCa-Ca_SBR_domesticated
GAATTCAATCCTCACGAAAACTTCCTTCAATGCTTCTCCCAACATATCTCCAACAATACAACCCTTGCCAAAC
TCATATACACTCCAAACGACCCGTCGTATATCTCTGTCCTAAATTCGACCATACAAAACCTTAGATTCTCTTC
TCCTTCAACCCCAAAACCACTCGTTATCGTCACACCTTCAAATGCCTCCCATGTCCAAGCCTCTGTTTTATGC
TCCAAGAAATATGGCTTGCAGATTCGAACTCGAAGCGGCGGCCATGACTCTGAGGGTGTCTCCTACGTGTCTG
AAGTCCCATTTGTCATAGTAGACATGAGAAACCTACGTTCGATCACTGTAGACGTAGATAACAAAACTGCATG
GGTTGAAGCTGGAGCTACCCTTGGAGAAGTTTATTATAGGATTGCTGAGAAAAATGAGAATCTCAGTTTTCCT
GCTGGCTATTGCCCTACTGTTGGCGTAGGTGGGCACTTCAGTGGAGGAGGCTATGGAGCATTGATGCGAAAAT
ATGGCCTTGCAGCTGATAATATCATTGATGCTCACTTAGTCAACGTTGATGGAAAAGTTCTTGACCGAAAATC
TATGGGGGAAGATCTGTTTTGGGCCATACGTGGTGGTGGAGGAGCAAGCTTTGGAATCATTCTCGCTTGGAAA
ATTAGATTGGTTCCTGTCCCATCAAAGGTTACTATATTCAGTGTTAAAAAGAACTTGGAGATAAATGAAACCG
TGAAGCTTGTTAACAAGTGGCAAAATATTGCTCACAAGTTTGACAAAGATTTGTTAATCATGACTCACTTCAT
AACTAGGAATATTACTGATGAACAAGGGAAGAATAAGACAACAATACACACTTACTTCTCTTCTATTTTCCTT
GGTGGAGTGGATAGTCTACTTGCCTTGATGGAAAAGAGCTTTCCTGAGTTGGGTGTGAAAAGAAAAGATTGCA
TCGAAATGAGCTGGATTGATACTATCATCTTCTACAGTGGTGTTGTAGCTAATTTTAAAAAGGAAATTTTGCT
TGATAGATCAGCTGGGCAAAAGGGTGCTTTCAAGATTAAACTAGACTACGTTAAGAAGCCAATTCCAGAAACT
GTAATGGTCAAAATTTTGGAGAAGTTATATGAAGAAGATGTAGGAGTGGGAATGTATGTGTTGTACCCTTATG
GTGGTAAAATGGACGAGATTCCTGAATCAGCAATTCCATTCCCTCACCGAGCTGGAATCATGTACGAAATTTG
GTACATATCTACCTGGGAGAAAGAAGAAGATGGTGAAAGGCATATGAATTGGGTTCGAAGTGTTTACAATTAC
ATGACTCCCTATGTGTCCAAAAATCCAAGAGCTGCATATCTCAATTATAGGGACCTTGATTTGGGAACAAATA
ACGACAAGGGTCCTACTAGTTATGCACAAGCAAGTATTTGGGGAAAAAAGTATTTCGGTAAAAACTTTAAGAG
GTTAGTTCATGTGAAAACCAAGGTTGATCCCACTAATTTCTTCAGGAACGAACAAAGCATCCCACCTCTTCCG
CGACGTCTCCATCATCATCATCATCATtaaTCTAGA

>HCa-Ca_SBR_Ins_domesticated
GAATTCAATCCTCACGAAAACTTCCTTCAATGCTTCTCCCAACATATCTCCAACAATACAACCCTTGCCAAAC
TCATATACACTCCAAACGACCCGTCGTATATCTCTGTCCTAAATTCGACCATACAAAACCTTAGATTCTCTTC
TCCTTCAACCCCAAAACCACTCGTTATCGTCACACCTTCAAATGCCTCCCATGTCCAAGCCTCTGTTTTATGC
TCCAAGAAATATGGCTTGCAGATTCGAACTCGAAGCGGCGGCCATGACTCTGAGGGTGTCTCCTACGTGTCTG
AAGTCCCATTTGTCATAGTAGACATGAGAAACCTACGTTCGATCACTGTAGACGTAGATAACAAAACTGCATG

GGTTGAAGCTGGAGCTACCCTTGGAGAAGTTTATTATAGGATTGCTGAGAAAAATGAGAATCTCAGTTTTCCT
GCTGGCTATTGCCCTACTGTTGGCGTAGGTGGGCACTTCAGTGGAGGAGGCTATGGAGCATTGATGCGAAAAT
ATGGCCTTGCAGCTGATAATATCATTGATGCTCACTTAGTCAACGTTGATGGAAAAGTTCTTGACCGAAAATC
TATGGGGGAAGATCTGTTTTGGGCCATACGTGGTGGTGGAGGAGCAAGCTTTGGAATCATTCTCGCTTGGAAA
ATTAGATTGGTTCCTGTCCCATCAAAGGTTACTATATTCAGTGTTAAAAAGAACTTGGAGATAAATGAAACCG
TGAAGCTTGTTAACAAGTGGCAAAATATTGCTCACAAGTTTGACAAAGATTTGTTAATCATGACTCACTTCAT
AACTAGGAATATTACTGATGAACAAGGGAAGAATAAGACAACAATACACACTTACTTCTCTTCTATTTTCCTT
GGTGGAGTGGATAGTCTACTTGCCTTGATGGAAAAGAGCTTTCCTGAGTTGGGTGTGAAAAGAAAAGATTGCA
TCGAAATGAGCTGGATTGATACTATCATCTTCTACAGTGGTGTTGTAAATTACAACACTGCTAATTTTAAAAA
GGAAATTTTGCTTGATAGATCAGCTGGGCAAAAGGGTGCTTTCAAGATTAAACTAGACTACGTTAAGAAGCCA
ATTCCAGAAACTGTAATGGTCAAAATTTTGGAGAAGTTATATGAAGAAGATGTAGGAGTGGGAATGTATGTGT
TGTACCCTTATGGTGGTAAAATGGACGAGATTCCTGAATCAGCAATTCCATTCCCTCACCGAGCTGGAATCAT
GTACGAAATTTGGTACATATCTACCTGGGAGAAAGAAGAAGATGGTGAAAGGCATATGAATTGGGTTCGAAGT
GTTTACAATTACATGACTCCCTATGTGTCCAAAAATCCAAGAGCTGCATATCTCAATTATAGGGACCTTGATT
TGGGAACAAATAACGACAAGGGTCCTACTAGTTATGCACAAGCAAGTATTTGGGGAAAAAAGTATTTCGGTAA
AAACTTTAAGAGGTTAGTTCATGTGAAAACCAAGGTTGATCCCACTAATTTCTTCAGGAACGAACAAAGCATC
CCACCTCTTCCGCGACGTCTCCATCATCATCATCATCATtaaTCTAGA

>HCa-Ca_SBR_Ins_FAD_domesticated
GAATTCAATCCTCACGAAAACTTCCTTCAATGCTTCTCCCAACATATCTCCAACAATACAACCCTTGCCAAAC
TCATATACACTCCAAACGACCCGTCGTATATCTCTGTCCTAAATTCGACCATACAAAACCTTAGATTCTCTTC
TCCTTCAACCCCAAAACCACTCGTTATCGTCACACCTTCAAATGCCTCCCATGTCCAAGCCTCTGTTTTATGC
TCCAAGAAATATGGCTTGCAGATTCGAACTCGAAGCGGCGGCCATGACTCTGAGGGTATGTCCTACGTGTCTG
AAGTCCCATTTGTCATAGTAGACTTGAGAAACCTACGTTCGATCACTGTAGACGTAGATAACAAAACTGCATG
GGTTGAAGCTGGAGCTACCCTTGGAGAAGTTTATTATAGGATTGCTGAGAAAAATGAGAATCTCAGTTTTCCT
GCTGGCTATTGCCCTACTGTTGGCGTAGGTGGGCACTTCAGTGGAGGAGGCTATGGAGCATTGATGCGAAAAT
ATGGCCTTGCAGCTGATAATATCATTGATGCTCACTTAGTCAACGTTGATGGAAAAGTTCTTGACCGAAAATC
TATGGGGGAAGATCTGTTTTGGGCCATACGTGGTGGTGGAGGAGAAAGCTTTGGAATCATTGTAGCTTGGAAA
ATTAGATTGGTTCCTGTCCCATCAAAGGTTACTATATTCAGTGTTAAAAAGAACTTGGAGATAAATGAAACCG
TGAAGCTTGTTAACAAGTGGCAAAATATTGCTCACAAGTTTGACAAAGATTTGTTAATCATGACTCACTTCAT
AACTAGGAATATTACTGATGAACAAGGGAAGAATAAGACAACAATACACACTTACTTCTCTTCTATTTTCCTT
GGTGGAGTGGATAGTCTACTTGCCTTGATGGAAAAGAGCTTTCCTGAGTTGGGTGTGAAAAGAAAAGATTGCA
TCGAAATGAGCTGGATTGATACTATCATCTTCTACAGTGGTGTTGTAAATTACAACACTGCTAATTTTAAAAA
GGAAATTTTGCTTGATAGATCAGCTGGGCAAAAGGGTGCTTTCAAGATTAAACTAGACTACGTTAAGAAGCCA
ATTCCAGAAACTGTAATGGTCAAAATTTTGGAGAAGTTATATGAAGAAGATGTAGGAGTGGGAATGTATGTGT
TGTACCCTTATGGTGGTAAAATGGACGAGATTCCTGAATCAGCAATTCCATTCCCTCACCGAGCTGGAATCAT
GTACGAAATTTGGTACATATCTACCTGGGAGAAAGAAGAAGATGGTGAAAGGCATATGAATTGGGTTCGAAGT
GTTTACAATTACATGACTCCCTATGTGTCCAAAAATCCAAGAGCTGCATATCTCAATTATAGGGACCTTGATT
TGGGAACAAATAACGACAAGGGTCCTACTAGTTATGCACAAGCAAGTATTTGGGGAAAAAAGTATTTCGGTAA
AAACTTTAAGAGGTTAGTTCATGTGAAAACCAAGGTTGATCCCACTAATTTCTTCAGGAACGAACAAAGCATC
CCACCTCTTCCGCGACGTCTCCATCATCATCATCATCATtaaTCTAGA

>Ca-CBDAS_SBR_domesticated
GAATTCAATCCTCAAGAAAACTTCCTTAAATGCTTCTCGCAATATATTCCCAACAATGCAACAAATCTAAAAC
TCGTATACACTCAAAACGACCAATTGTATATGTCTGTCCTAAATTCGACAATACACAATCTTAGATTCACCTC
TGACACAACCCCAAAACCACTTGTTATCGTCACTCCTTCAAATGTCTCCCATATCCAAGGCACTATTCTATGC
TCCAAGAAAGTTGGCTTGCAGATTCGAACTCGAAGCGGTGGTCATGATTCTGAGGGCATGTCCTACATATCTC
AAGTCCCATTTGTTATAGTAGACTTGAGAAACATGCATTCGATCAAAATAGATGTTCATAGCCAAACTGCATG
GGTTGAAGCCGGAGCTACCCTTGGAGAAGTTTATTATTGGATTAATGAGAAAAATGAGAATCTTAGTTTTCCT
GCTGGGTATTGCCCTACTGTTGGCGCAGGTGGACACTTTAGTGGAGGAGGCTATGGACCATTGATGCGAAATT
ATGGCCTCGCGGCTGATAATATCATTGATGCACACTTAGTCAACGTTGATGGAAAAGTTCTGGATCGAAAATC
CATGGGGGAAGATCTGTTTTGGGCTATACGTGGTGGTGGAGGAGAAAGCTTTGGAATCATTGTAGCGTGGAAA
ATTAGACTGGTTGCTGTCCCATCAAAGTCTACTATGTTCAGTGTTAAAAAGATCATGGAGATACATGAGCTTG
TCAAGTTAGTTAACAAATGGCAAAATATTGCTTACAAGTATGACAAAGATTTATTACTCATGACTCACTTCAT
AACTAGGAATATTACAGATAATCAAGGGAAGAATAAGACAGCAATACACACTTACTTCTCTTCCGTTTTCCTT
GGTGGAGTGGATAGTCTAGTCGACTTGATGAACAAGAGCTTTCCTGAGTTGGGTATTAAAAAAACAGATTGCA
GACAATTGAGCTGGATTGATACTATCATCTTCTACAGTGGTGTTGTAAATTACGACACTGATAATTTTAACAA
GGAAATTTTGCTTGATAGATCAGCTGGGCAGAACGGGGCTTTCAAGATTAAGTTAGACTACGTTAAGAAACCA

ATTCCAGAAACTGCAATGGTCAAAATTTTGGAAAAATTATATGAAGAAGATATAGGAGTTGGGATGTATGCGT
TGTACCCTTACGGTGGTATAATGGATGAGATTTCAGAATCAGCAATTCCATTCCCTCATCGAGCTGGAATCAT
GTATGAAATTTGGTACATATGTACCTGGGAGAAGCAAGAAGATAATGAAAAGCATATAAACTGGATTCGAAAT
GTTTATAATTTCATGACTCCTTATGTGTCCCAAAATCCAAGAATGGCATATCTCAATTATAGGGACCTTGATT
TAGGAAAAAATAATCCCAAGAGTCCTAATAATTACACACAAGCACGTATTTGGGGTGAAAAGTATTTTGGTAA
AAATTTTAACAGGTTAGTAAAAGTGAAAACCAAGGTTGATCCCAATAATTTTTTTAGAAACGAACAAAGCATC
CCACCTCTTCCACGGCATCGTCATCATCATCATCATCATtaaTCTAGA

>Ca-CBDAS_SBR_FAD_domesticated
GAATTCAATCCTCAAGAAAACTTCCTTAAATGCTTCTCGCAATATATTCCCAACAATGCAACAAATCTAAAAC
TCGTATACACTCAAAACGACCAATTGTATATGTCTGTCCTAAATTCGACAATACACAATCTTAGATTCACCTC
TGACACAACCCCAAAACCACTTGTTATCGTCACTCCTTCAAATGTCTCCCATATCCAAGGCACTATTCTATGC
TCCAAGAAAGTTGGCTTGCAGATTCGAACTCGAAGCGGTGGTCATGATTCTGAGGGCATGTCCTACATATCTC
AAGTCCCATTTGTTATAGTAGACTTGAGAAACATGCATTCGATCAAAATAGATGTTCATAGCCAAACTGCATG
GGTTGAAGCCGGAGCTACCCTTGGAGAAGTTTATTATTGGATTAATGAGAAAAATGAGAATCTTAGTTTTCCT
GCTGGGTATTGCCCTACTGTTTGCGCAGGTGGACACTTTGGTGGAGGAGGCTATGGACCATTGATGCGAAATT
ATGGCCTCGCGGCTGATAATATCATTGATGCACACTTAGTCAACGTTGATGGAAAAGTTCTGGATCGAAAATC
CATGGGGGAAGATCTGTTTTGGGCTTTACGTGGTGGTGGAGCAGAAAGCTTTGGAATCATTGTAGCGTGGAAA
ATTAGACTGGTTGCTGTCCCATCAAAGTCTACTATGTTCAGTGTTAAAAAGATCATGGAGATACATGAGCTTG
TCAAGTTAGTTAACAAATGGCAAAATATTGCTTACAAGTATGACAAAGATTTATTACTCATGACTCACTTCAT
AACTAGGAATATTACAGATAATCAAGGGAAGAATAAGACAGCAATACACACTTACTTCTCTTCCGTTTTCCTT
GGTGGAGTGGATAGTCTAGTCGACTTGATGAACAAGAGCTTTCCTGAGTTGGGTATTAAAAAAACAGATTGCA
GACAATTGAGCTGGATTGATACTATCATCTTCTACAGTGGTGTTGTAAATTACGACACTGATAATTTTAACAA
GGAAATTTTGCTTGATAGATCAGCTGGGCAGAACGGGGCTTTCAAGATTAAGTTAGACTACGTTAAGAAACCA
ATTCCAGAAACTGCAATGGTCAAAATTTTGGAAAAATTATATGAAGAAGATATAGGAGTTGGGATGTATGCGT
TGTACCCTTACGGTGGTATAATGGATGAGATTTCAGAATCAGCAATTCCATTCCCTCATCGAGCTGGAATCAT
GTATGAAATTTGGTACATATGTACCTGGGAGAAGCAAGAAGATAATGAAAAGCATATAAACTGGATTCGAAAT
GTTTATAATTTCATGACTCCTTATGTGTCCCAAAATCCAAGAATGGCATATCTCAATTATAGGGACCTTGATT
TAGGAAAAAATAATCCCAAGAGTCCTAATAATTACACACAAGCACGTATTTGGGGTGAAAAGTATTTTGGTAA
AAATTTTAACAGGTTAGTAAAAGTGAAAACCAAGGTTGATCCCAATAATTTTTTTAGAAACGAACAAAGCATC
CCACCTCTTCCACGGCATCGTCATCATCATCATCATCATtaaTCTAGA

>Ca-A1A2a_SBR_domesticated
GAATTCAATCCTCAAGAAAACTTCCTTAAATGCTTCTCGCAATATATTCCCAACAATGCAACAAATCTAAAAC
TCGTATACACTCAAAACGACCAATTGTATATGTCTGTCCTAAATTCGACAATACAAAATCTTAGATTCACCTC
TGACACAACCCCAAAACCACTTGTTATCGTCACTCCTTCAAATGTCTCCCATATCCAAGGCACTATTCTATGC
TCCAAGAAAGTTGGCTTGCAGATTCGAACTCGAAGCGGTGGTCATGATGCTGAGGGCATGTCCTACATATCTC
AAGTCCCATTTGTTATAGTAGACTTGAGAAACATGCATTCGATCAAAATAGATGTTCATAGCCAAACTGCATG
GGTTGAAGCCGGAGCTACCCTTGGAGAAGTTTATTATTGGATTAATGAGAAAAATGAGAATCTTAGTTTTCCT
GGTGGGTATTGCCCTACTGTTGGCGCAGGTGGACACTTTAGTGGAGGAGGCTATGGAGCATTGATGCGAAATT
ATGGCCTCGCGGCTGATAATATCATTGATGCACACTTAGTCAACGTTGATGGAAAAGTTCTGGATCGAAAATC
CATGGGGGAAGATCTGTTTTGGGCTATACGTGGTGGTGGAGGAGAAAGCTTTGGAATCATTGTAGCGTGGAAA
ATTAGACTGGTTGCTGTCCCATCAAAGTCTACTATATTCAGTGTTAAAAAGAACATGGAGATACATGAGCTTG
TCAAGTTAGTTAACAAATGGCAAAATATTGCTTACAAGTATGACAAAGATTTATTACTCATGACTCACTTCAT
AACTAGGAATATTACAGATAATCAAGGGAAGAATAAGACAACAATACACGGTTACTTCTCTTCCATTTTCCTT
GGTGGAGTGGATAGTCTAGTCGACTTGATGAACAAGAGCTTTCCTGAGTTGGGTATTAAAAAAACAGATTGCA
AACAATTGAGCTGGATTGATACTACCATCTTCTACAGTGGTGTTGTAAATTACAACACTGCTAATTTTAAAAA
GGAAATTTTGCTTGATAGATCAGCTGGGCAGAAGACGGCTTTCTCAATTAAGTTAGACTACGTTAAGAAACCA
ATTCCAGAAACTGCAATGGTCAAAATTTTGGAAAAATTATATGAAGAAGATGTAGGAGTTGGGATGTATGTGT
TGTACCCTTACGGTGGTATAATGGATGAGATTTCAGAATCAGCAATTCCATTCCCTCATCGAGCTGGAATCAT
GTATGAAATTTGGTACACTGCTACCTGGGAGAAGCAAGAAGATAATGAAAAGCATATAAACTGGATTCGAAAT
GTTTATAATTTCATGACTCCTTATGTGTCCCAAAATCCAAGAATGGCATATCTCAATTATAGGGACCTTGATT
TAGGAAAAAATAATCCCAAGAGTCCTAATAATTACACACAAGCACGTATTTGGGGTGAAAAGTATTTTGGTAA
AAATTTTAACAGGTTAGTAAAAGTGAAAACCAAGGTTGATCCCAATAATTTTTTTAGAAACGAACAAAGCATC
CCACCTCTTCCACGGCATCGTCATCATCATCATCATCATtaaTCTAGA

>THCAS-CBDAS_domesticated

GAATTCAATCCTCGAGAAAACTTCCTTAAATGCTTCTCAAAACATATTCCCAACAATGTAGCAAATCCAAAAC
TCGTATACACTCAACACGACCAATTGTATATGTCTATCCTTAATTCGACAATACACAATCTTAGATTCATCTC
TGATACAACCCCAAAACCACTCGTTATTGTCACTCCTTCAAATAACTCCCATATCCAAGCAACTATTTTATGC
TCTAAGAAAGTTGGCTTGCAGATTCGAACTCGAAGCGGTGGCCATGATTCTGAGGGTATGTCCTACATATCTC
AAGTCCCATTTGTTGTAGTAGACTTGAGAAACATGCATTCGATCAAAATAGATGTTCATAGCCAAACTGCGTG
GGTTGAAGCCGGAGCTACCCTTGGAGAAGTTTATTATTGGATCAATGAGAAGAATGAGAATCTTAGTTTTCCT
GCTGGGTATTGCCCTACTGTTGGCGTAGGTGGACACTTTAGTGGAGGAGGCTATGGACCATTGATGCGAAATT
ATGGCCTTGCGGCTGATAATATTATTGATGCACACTTAGTCAATGTTGATGGAAAAGTTCTGGATCGAAAATC
CATGGGAGAAGATCTGTTTTGGGCTATACGTGGTGGTGGAGGAGAAAACTTTGGAATCATTGCAGCATGGAAA
ATCAAACTGGTTGCTGTCCCATCAAAGTCTACTATGTTCAGTGTTAAAAAGATCATGGAGATACATGGGCTTG
TCAAGTTATTTAACAAATGGCAAAATATTGCTTACAAGTATGACAAAGATTTAGTACTCATGACTCACTTCAT
AACAAAGAATATTACAGATAATCATGGGAAGAATAAGACTGCAGTACATACTTACTTCTCTTCAGTTTTTCAT
GGTGGAGTGGATAGTCTAGTCGACTTGATGAACAAGAGCTTTCCTGAGTTGGGTATTAAAAAAACTGATTGCA
GAGAATTTAGCTGGATTGATACAATCATCTTCTACAGTGGTGTTGTAAATTTTGACACTGATAATTTTAACAA
GGAAATTTTGCTTGATAGATCAGCTGGGAAGAACGGGGCTTTCAAGATTAAGTTAGACTATGTTAAGAAACCA
ATTCCAGAAACTGCAATGGTCAAAATTTTGGAAAAATTATATGAAGAAGATATAGGAGCTGGGATGTATGCGT
TGTACCCTTACGGTGGTATAATGGAGGAGATTTCAGAATCAGCAATTCCATTCCCTCATCGAGCTGGAATAAT
GTATGAACTTTGGTACATATGTTCCTGGGAGAAGCAAGAAGATAATGAAAAGCATATAAACTGGGTTCGAAGT
GTTTATAATTTTACGACTCCTTATGTGTCCCAAAATCCAAGATTGGCGTATCTCAATTATAGGGACCTTGATT
TAGGAAAAACTAATCATGCGAGTCCTAATAATTACACACAAGCACGTATTTGGGGTGAAAAGTATTTTGGTAA
AAATTTTAACAGGTTAGTTAAGGTGAAAACTAAAGTTGATCCCAATAATTTTTTTAGAAACGAACAAAGTATC
CCACCTCTTCCACCGCATCATCATCATCATCATtaaTCTAGA
