# Supplementary material for: Resurrected Ancestral Cannabis Enzymes Unveil the Origin and Functional Evolution of Cannabinoid Synthases
Source: Plant Biotechnol J. 2025 Dec 26;24(4):2685–97. doi: 10.1111/pbi.70475 (PMC13140220; doi:10.1111/pbi.70475)
Supplement: Supplementary file 1 — Figure S1: Phylogeny of Cannabaceae‐specific Berberine Bridge‐Like genes. Figure S2: Syntenic blocks comprising cannabinoid synthase genes and closely‐related BBLs. Figure S3: Evaluation of enzyme expression by immunodetection. Figure S4: Determination of the optimal pH and reactional temperature for the activity of Ca. Figure S5: Determination of the optimal pH for the activity of HCa → CaSBR and Ca → CBDASSBR_FAD. Figure S6: Design and structure of the THCAS → CBDAS hybrid. Table S1: Analysis of the reconstructed ancestral sequences. Table S2: Design of the HCa → Ca (a), Ca → CBDAS (b) and Ca → A1A2a (c) hybrids, based on sequence and structural comparison. Table S3: Expression level of candidate enzymes (μg mL−1). Table S4: Comparison of the mutations tested in previous studies with mutations included in our hybrids. Table S5: Quality assessment of the three‐dimensional (3D) enzyme homology models. Data S1: Sequence alignment used to generate the gene‐tree and reconstruct the ancestors. Data S2: Ancestral sequences reconstructed with MrBayes and PAML. Data S3: Sequences of A1A1a, Ca and HCa. Data S4: Domesticated sequences used to express and characterise enzymes. Data S5: Berberine Bridge‐Like dataset. Data S6: Ancestral sequence reconstruction with MrBayes. Data S7: Ancestral sequence reconstruction with PAML. [file PBI-24-2685-s001.zip › pbi70475-sup-0009-DataS5.pdf]

>A1_LA_confidential_LKUA01068262.1_650
ATGAATTGCTCAGCATTTTCCTTTTGGTTTGTTTGCAAAATAATATTTTTCTTTCTCTCATTCCATATCCAAA
TTTCAATAGCTAATCCTCGAGAAAACTTCCTTAAATGCTTCTCAAAACATATTCCCAACAATGTAGCAAATCC
AAAACTCGTATACACTCAACACGACCAATTGTATATGTCTATCCTGAATTCGACAATACAAAATCTTAGATTC
ATCTCTGATACAACCCCAAAACCACTCGTTATTGTCACTCCTTCAAATAACTCCCATATCCAAGCAACTATTT
TATGCTCTAAGAAAGTTGGCTTGCAGATTCGAACTCGAAGCGGTGGCCATGATGCTGAGGGTATGTCCTACAT
ATCTCAAGTCCCATTTGTTGTAGTAGACTTGAGAAACATGCATTCGATCAAAATAGATGTTCATAGCCAAACT
GCGTGGGTTGAAGCCGGAGCTACCCTTGGAGAAGTTTATTATTGGATCAATGAGAAGAATGAGAATCTTAGTT
TTCCTGGTGGGTATTGCCCTACTGTTGGCGTAGGTGGACACTTTAGTGGAGGAGGCTATGGAGCATTGATGCG
AAATTATGGCCTTGCGGCTGATAATATTATTGATGCACACTTAGTCAATGTTGATGGAAAAGTTCTAGATCGA
AAATCCATGGGAGAAGATCTGTTTTGGGCTATACGTGGTGGTGGAGGAGAAAACTTTGGAATCATTGCAGCAT
GGAAAATCAAACTGGTTGCTGTCCCATCAAAGTCTACTATATTCAGTGTTAAAAAGAACATGGAGATACATGG
GCTTGTCAAGTTATTTAACAAATGGCAAAATATTGCTTACAAGTATGACAAAGATTTAGTACTCATGACTCAC
TTCATAACAAAGAATATTACAGATAATCATGGGAAGAATAAGACTACAGTACATGGTTACTTCTCTTCAATTT
TTCATGGTGGAGTGGATAGTCTAGTCGACTTGATGAACAAGAGCTTTCGTGAGTTGGGTATTAAAAAAACTGA
TTGCAAAGAATTTAGCTGGATTGATACAACCATCTTCTACAGTGGTGTTGTAAATTTTAACACTGCTAATTTT
AAAAAGGAAATTTTGCTTGATAGATCAGCTGGGAAGAAGACGGCTTTCTCAATTAAGTTAGACTATGTTAAGA
AACCAATTCCAGAAACTGCAATGGTCAAAATTTTGGAAAAATTATATGAAGAAGATGTAGGAGCTGGGATGTA
TGTGTTGTACCCTTACGGTGGTATAATGGAGGAGATTTCAGAATCAGCAATTCCATTCCCTCATCGAGCTGGA
ATAATGTATGAACTTTGGTACACTGCTTCCTGGGAGAAGCAAGAAGATAATGAAAAGCATATAAACTGGGTTC
GAAGTGTTTATAATTTTACGACTCCTTATGTGTCCCAAAATCCAAGATTGGCGTATCTCAATTATAGGGACCT
TGATTTAGGAAAAACTAATCATGCGAGTCCTAATAATTACACACAAGCACGTATTTGGGGTGAAAAGTATTTT
GGTAAAAATTTTAACAGGTTAGTTAAGGTGAAAACTAAAGTTGATCCCAATAATTTTTTTAGAAACGAACAAA
GTATCCCACCTCTTCCACCGCATCATCATTAA
>A1_PBBK_MXBD01001769.1_40421
ATGAATTGCTCAGCATTTTCCTTTTGGTTTGTTTGCAAAATAATATTTTTCTTTCTCTCATTCCATATCCAAA
TTTCAATAGCTAATCCTCGAGAAAACTTCCTTAAATGCTTCTCAAAACATATTCCCAACAATGTAGCAAATCC
AAAACTCGTATACACTCAACACGACCAATTGTATATGTCTATCCTGAATTCGACAATACAAAATCTTAGATTC
ATCTCTGATACAACCCCAAAACCACTCGTTATTGTCACTCCTTCAAATAACTCCCATATCCAAGCAACTATTT
TATGCTCTAAGAAAGTTGGCTTGCAGATTCGAACTCGAAGCGGTGGCCATGATGCTGAGGGTATGTCCTACAT
ATCTCAAGTCCCATTTGTTGTAGTAGACTTGAGAAACATGCATTCGATCAAAATAGATGTTCATAGCCAAACT
GCGTGGGTTGAAGCCGGAGCTACCCTTGGAGAAGTTTATTATTGGATCAATGAGAAGAATGAGAATCTTAGTT
TTCCTGGTGGGTATTGCCCTACTGTTGGCGTAGGTGGACACTTTAGTGGAGGAGGCTATGGAGCATTGATGCG
AAATTATGGCCTTGCGGCTGATAATATTATTGATGCACACTTAGTCAATGTTGATGGAAAAGTTCTAGATCGA
AAATCCATGGGAGAAGATCTGTTTTGGGCTATACGTGGTGGTGGAGGAGAAAACTTTGGAATCATTGCAGCAT
GGAAAATCAAACTGGTTGCTGTCCCATCAAAGTCTACTATATTCAGTGTTAAAAAGAACATGGAGATACATGG
GCTTGTCAAGTTATTTAACAAATGGCAAAATATTGCTTACAAGTATGACAAAGATTTAGTACTCATGACTCAC
TTCATAACAAAGAATATTACAGATAATCATGGGAAGAATAAGACTACAGTACATGGTTACTTCTCTTCAATTT
TTCATGGTGGAGTGGATAGTCTAGTCGACTTGATGAACAAGAGCTTTCCTGAGTTGGGTATTAAAAAAACTGA
TTGCAAAGAATTTAGCTGGATTGATACAACCATCTTCTACAGTGGTGTTGTAAATTTTAACACTGCTAATTTT
AAAAAGGAAATTTTGCTTGATAGATCAGCTGGGAAGAAGACGGCTTTCTCAATTAAGTTAGACTATGTTAAGA
AACCAATTCCAGAAACTGCAATGGTCAAAATTTTGGAAAAATTATATGAAGAAGATGTAGGAGCTGGGATGTA
TGTGTTGTACCCTTACGGTGGTATAATGGAGGAGATTTCAGAATCAGCAATTCCATTCCCTCATCGAGCTGGA
ATAATGTATGAACTTTGGTACACTGCTTCCTGGGAGAAGCAAGAAGATAATGAAAAGCATATAAACTGGGTTC
GAAGTGTTTATAATTTTACGACTCCTTATGTGTCCCAAAATCCAAGATTGGCGTATCTCAATTATAGGGACCT
TGATTTAGGAAAAACTAATCATGCGAGTCCTAATAATTACACACAAGCACGTATTTGGGGTGAAAAGTATTTT
GGTAAAAATTTTAACAGGTTAGTTAAGGTGAAAACTAAAGTTGATCCCAATAATTTTTTTAGAAACGAACAAA
GTATCCCACCTCTTCCACCGCATCATCATTAA
>A1_Cannatonic_MNPR01002992_10888
ATGAATTGCTCAGCATTTTCCTTTTGGTTTGTTTGCAAAATAATATTTTTCTTTCTCTCATTCCATATCCAAA
TTTCAATAGCTAATCCTCGAGAAAACTTCCTTAAATGCTTCTCAAAACATATTCCCAACAATGTAGCAAATCC
AAAACTCGTATACACTCAACACGACCAATTGTATATGTCTATCCTGAATTCGACAATACAAAATCTTAGATTC
ATCTCTGATACAACCAAACCACTCGTTATTGTCACTCCTTCAAATAACTCCCATATCCAAGCAACTATTTTAT
GCTCTAAGAAAGTTGGCTTGCAGATTCGAACTCGAAGCGGTGGCCATGATGCTGAGGGTATGTCCTACATATC
TCAAGTCCCATTTGTTGTAGTAGACTTGAGAAACATGCATTCGATCAAAATAGATGTTCATAGCCAAACTGCG
TGGGTTGAAGCCGGAGCTACCCTTGGAGAAGTTTATTATTGGATCAATGAGAAGAATGAGAATCTTAGTTTTC
CTGGTGGGTATTGCCCTACTGTTGGCGTAGGTGGACACTTTAGTGGAGGAGGCTATGGAGCATTGATGCGAAA
TTATGGCCTTGCGGCTGATAATATTATTGATGCACACTTAGTCAATGTTGATGGAAAAGTTCTAGATCGAAAA

TCCATGGGAGAAGATCTGTTTTGGGCTATACGTGGTGGTGGAGGAGAAAACTTTGGAATCATTGCAGCATGGA
AAATCAAACTGGTTGCTGTCCCATCAAAGTCTACTATATTCAGTGTTAAAAAGAACATGGAGATACATGGGCT
TGTCAAGTTATTTAACAAATGGCAAAATATTGCTTACAAGTATGACAAAGATTTAGTACTCATGACTCACTTC
ATAACAAAGAATATTACAGATAATCATGGGAAGAATAAGACTACAGTACATGGTTACTTCTCTTCAATTTTTC
ATGGTGGAGTGGATAGTCTAGTCGACTTGATGAACAAGAGCTTTCCTGAGTTGGGTATTAAAAAAACTGATTG
CAAAGAATTTAGCTGGATTGATACAACCATCTTCTACAGTGGTGTTGTAAATTTTAACACTGCTAATTTTAAA
AAGGAAATTTTGCTTGATAGATCAGCTGGGAAGAAGACGGCTTTCTCAATTAAGTTAGACTATGTTAAGAAAC
CAATTCCAGAAACTGCAATGGTCAAAATTTTGGAAAAATTATATGAAGAAGATGTAGGAGCTGGGATGTATGT
GTTGTACCCTTACGGTGGTATAATGGAGGAGATTTCAGAATCAGCAATTCCATTCCCTCATCGAGCTGGAATA
ATGTATGAACTTTGGTACACTGCTTCCTGGGAGAAGCAAGAAGATAATGAAAAGCATATAAACTGGGTTCGAA
GTGTTTATAATTTTACGACTCCTTATGTGTCCCAAAATCCAAGATTGGCGTATCTCAATTATAGGGACCTTGA
TTTAGGAAAAACTAATCATGCGAGTCCTAATAATTACACACAAGCACGTATTTGGGGTGAAAAGTATTTTGGT
AAAAATTTTAACAGGTTAGTTAAGGTGAAAACTAAAGTTGATCCCAATAATTTTTTTAGAAACGAACAAAGTA
TCCCACCTCTTCCACCGCATCATCATTAA
>A1_LN998183.1
AATAACCCCCATATCCAAGCAACTATTTTATGCTCTAAGAAAGTTGGCTTGCAGATTCAACTCGAAGCGGTGG
CCATGATGCTGAGGGTATGTCCTACATATCTCAAGTCCCATTTGTGTAGTAGACTTGAGAACATGCATTCGAA
CCATTCGATCAAAATAGATGTTCATAGCCAAACTGCGTGGGTTGAAGCCGGAGCTACCCTTGGAGAAGTTTAT
TATTGGATCAATGAGAAGAATGAGAATCTTAGTTTTCCTGGTGGGTATTGCCCTACTGTTGGCGTAGGTGGAC
ACTTTAGTGGAGGAGGCTATGGAGCATTGATGCGAAATTATGGCCTTGCGGCTGATAATATTATTGATGCACA
CTTAGTCAATGTTGATGGAAAAGTTCTAGATCGAAAATCCATGGGAGAAGATCTGTTTTGGGCTATACGTGGT
GGTGGAGGAGAAAACTTTGGAATCATTGCAGCATGGAAAATCAAACTGGTTGCTGTCCCATCAAAGTCTACTA
TATTCAGTGTTAAAAAGAACATGGAGATACATGGGCTTGTCAAGTTATTTAACAAATGGCAAAATATTGCTTA
CAAGTATGTCAAAGATTTAGTACTCATGACTCACTTCATACCAAAGAATATTACAGATAATCATGGGAAGAAT
AAGACTACAGTACATGGTTACTTCTCTTCAATTTTTCATGGTGGAGTGGATAGTCTAGTCGACTTGATGAACA
AGAGCTTTCCTGAGTTGGGTATTAAAAAAACTGATTGCAAAGAATTTAGCTGGATTGATACAACCATCTTCTA
CAGTGGTGTTGTAAATTTTAACACTGCTAATTTTAAAAAGGAAATTTTGCTTGATAGATCAGCTGGGAAGAAG
ACGGCTTTCTCAATTAAGTTAGACTATGTTAAGAAACCAATTCCAGAAACTGCAATGGTCAAAATTTTGGAAA
AATTATATGAAGAAGATGTAGGAGCTGGGATGTATGTGTTGTACCCTTACGGTGGTATAATGGAGGAGATTTC
AGAATCAGCAATTCCATTCCCTCATCGAGCTGGAATAATGTATGAACTTTGGTACACTGCTTCCTGGGAGAAG
CAAGAAGATAATGAAAAGCATATAAACTGGGTTCGAAGTGTTTATAATTTTACGACTCCTTATGTGTCCCAAA
ATCCAAGATTGGCGTATCTCAATTATAGGGACCTTGATTTAGGAAAAACTAATCATGCGAGTCCT
>A1_MN422087.1
TTTTGGTTTGTTTGCAAAATAATATTTTTCTTTCTCTCATTCCATATCCAAATTTCAATAGCTAATCCTCGAG
AAAACTTCCTTAAATGCTTCTCAAAACATATTCCCAACAATGTAGCAAATCCAAAATTCGTATACACTCAACA
CGACCAATTGTATATGTCTATCCTGAATTCGACAATACAAAATCTTAGATTCATCTCTGATACAACCCCAAAA
CCACTCGTTATTGTCACTCCTTCAAATATCTCCCATATCCAAGCAACTATTTTATGCTCTAAGAAAGTTGGCT
TGCAGATTCGAACTCGAAGCGGTGGGCATGATGCTGAGGGTATGTCCTACATTTCTCAACTCCCATTTGTTAT
AGTAGACTTGAGAAACATGCATTCGGTCAAAATAGATGTTCATAGCCAAACTGCGTGGGTTGAAGCCGGAGCT
ACCCTTGGAGAAGTTTATTATTGGATCAATGAGAAGAATGAGAATCTTAGTTTTCCTGGTGGGTATTGCCCTA
CTGTTGGCGTAGGTGGACACTTTAGTGGAGGAGGCTATGGAGCATTGATGCGAAATTATGGCCTTGCGGCTGA
TAATATTATTGATTCACACTTAGTCAATGTTGATGGAAAAGTTCTAGATCGAAAATCCATGGGAGAAGATCTG
TTTTGGGCTATACGTGGTGGTGGAGGAGAAAACTTTGGAATCATTGCAGCATGGAAAATCAAACTGGTTGTTG
TCCCATCAAAGTCTACTATATTCAGTGTTAAAAAGAACATGGAGATACATGGGCTTGTCAAGTTATTTAACAA
ATGGCAAAATATTGCTTACAAGTATGACAAAGATTTAGTACTCATGACTCACTTCAGAACAAAGAATATTACA
GATAATCATGGGAAGAATAAGACTACAGTACATGGTTACTTCTCTTCAATTTTTCATGGTGGAGTGGATAGTC
TAGTCGACTTGATGAACAAGAGCTTTCCTGAGTTGGGTATTAAAAAAACTGATTGCAAAGAATTTAGCTGGTT
TGATACAACCATCTTCTACAGTGGTGTTGTAAATTTTAACACTGCTAATTTTAAAAAGGAAATTTTGCTTGAT
AGATCAGCTGGGAAGAAGACGGCTTTCTCAATTAAGTTAGACTATGTTAAGAAACCAATTCCAGAAACTGCAA
TGGTCAAAATTTTGGAAAAATTATATGAAGAAGATGTAGGAGCTGGGATGTATGTGTTGTACCCTTACGGTGG
TATAATGGAGGAGATTTCAGAATCAGCAATTCCATTCCCTCATCGAGCTGGAATAATGTATGAACTTTGGTAC
ACTGCTTCCTGGGAGAAGCAAGAAGATAATGAAAAGCATATAAACTGGGTTCGAAGTGTATATAATTTTACGA
CTCCTTATGTGTCCCAAAATCCAAGATTGGCGTATCTCAATTATAGGGACCTTGATTTAGGAAAAACTAATCC
TGCGAGTCCTAATAATTACACACAAGCACGTATTTGGGGTGAAAAGTATTTTGGTAAAAATTTTAACAGGTTA
GTTAAGGTGAAAACTAAAGTTGATCCCAATAATTTTTTTAGAAACGAACAAAGTATCCCACCTCTT
>A1_MN422085.1
TTTTGGTTTGTTTGCAAAATAATATTTTTCTTTCTCTCATTCCATATCCAAATTTCAATAGCTAATCCTCGAG
AAAACTTCCTTAAATGCTTCTCAAAACATATTCCCAACAATGTAGCAAATCCAAAATTCGTATACACTCAACA

CGACCAATTGTATATGTCTATCCTGAATTCGACAATACAAAATCTTAGATTCATCTCTGATACAACCCCAAAA
CCACTCGTTATTGTCACTCCTTCAAATATCTCCCATATCCAAGCAACTATTTTATGCTCTAAGAAAGTTGGCT
TGCAGATTCGAACTCGAAGCGGTGGGCATGATGCTGAGGGTATGTCCTACATTTCTCAACTCCCATTTGTTAT
AGTAGACTTGAGAAACATGCATTCGGTCAAAATAGATGTTCATAGCCAAACTGCGTGGGTTGAAGCCGGAGCT
ACCCTTGGAGAAGTTTATTATTGGATCAATGAGAAGAATGAGAATCTTAGTTTTCCTGGTGGGTATTGCCCTA
CTGTTGGCGTAGGTGGACACTTTAGTGGAGGAGGCTATGGAGCATTGATGCGAAATTATGGCCTTGCGGCTGA
TAATATTATTGATTCACACTTAGTCAATGTTGATGGAAAAGTTCTAGATCGAAAATCCATGGGAGAAGATCTG
TTTTGGGCTATACGTGGTGGTGGAGGAGAAAACTTTGGAATCATTGCAGCATGGAAAATCAAACTGGTTGTTG
TCCCATCAAAGTCTACTATATTCAGTGTTAAAAAGAACATGGAGATACATGGGCTTGTCAAGTTATTTAACAA
ATGGCAAAATATTGCTTACAAGTATGACAAAGATTTAGTACTCATGACTCACTTCAGAACAAAGAATATTACA
GATAATCATGGGAAGAATAAGACTACAGTACATGGTTACTTCTCTTCAATTTTTCATGGTGGAGTGGATAGTC
TAGTCGACTTGATGAACAAGAGCTTTCCTGAGTTGGGTATTAAAAAAACTGATTGCAAAGAATTTAGCTGGTT
TGATACAACCATCTTCTACAGTGGTGTTGTAAATTTTAACACTGCTAATTTTAAAAAGGAAATTTTGCTTGAT
AGATCAGCTGGGAAGAAGACGGCTTTCTCAATTAAGTTAGACTATGTTAAGAAACCAATTCCAGAAACTGCAA
TGGTCAAAATTTTGGAAAAATTATATGAAGAAGATGTAGGAGCTGGGATGTATGTGTTGTACCCTTACGGTGG
TATAATGGAGGAGATTTCAGAATCAGCAATTCCATTCCCTCATCGAGCTGGAATAATGTATGAACTTTGGTAC
ACTGCTTCCTGGGAGAAGCAAGAAGATAATGAAAAGCATATAAACTGGGTTCGAAGTGTATATAATTTTACGA
CTCCTTATGTGTCCCAAAATCCAAGATTGGCGTATCTCAATTATAGGGACCTTGATTTAGGAAAAACTAATCC
TGCGAGTCCTAATAATTACACACAAGCACGTATTTGGGGTGAAAAGTATTTTGGTAAAAATTTTAACAGGTTA
GTTAAGGTGAAAACCAAAGTTGATCCCAATAATTTTTTTAGAAACGAACAAAGTATCCCACCTCTT
>A1_MN422086.1
TTTTGGTTTGTTTGCAAAATAATATTTTTCTTTCTCTCATTCCATATCCAAATTTCAATAGCTAATCCTCGAG
AAAACTTCCTTAAATGCTTCTCAAAACATATTCCCAACAATGTAGCAAATCCAAAATTCGTATACACTCAACA
CGACCAATTGTATATGTCTATCCTGAATTCGACAATACAAAATCTTAGATTCATCTCTGATACAACCCCAAAA
CCACTCGTTATTGTCACTCCTTCAAATATCTCCCATATCCAAGCAACTATTTTATGCTCTAAGAAAGTTGGCT
TGCAGATTCGAACTCGAAGCGGTGGGCATGATGCTGAGGGTATGTCCTACATTTCTCAACTCCCATTTGTTAT
AGTAGACTTGAGAAACATGCATTCGGTCAAAATAGATGTTCATAGCCAAACTGCGTGGGTTGAAGCCGGAGCT
ACCCTTGGAGAAGTTTATTATTGGATCAATGAGAAGAATGAGAATCTTAGTTTTCCTGGTGGGTATTGCCCTA
CTGTTGGCGTAGGTGGACACTTTAGTGGAGGAGGCTATGGAGCATTGATGCGAAATTATGGCCTTGCGGCTGA
TAATATTATTGATTCACACTTAGTCAATGTTGATGGAAAAGTTCTAGATCGAAAATCCATGGGAGAAGATCTG
TTTTGGGCTATACGTGGTGGTGGAGGAGAAAACTTTGGAATCATTGCAGCATGGAAAATCAAACTGGTTGTTG
TCCCATCAAAGTCTACTATATTCAGTGTTAAAAAGAACATGGAGATACATGGGCTTGTCAAGTTATTTAACAA
ATGGCAAAATATTGCTTACAAGTATGACAAAGATTTAGTACTCATGACTCACTTCAGAACAAAGAATATTACA
GATAATCATGGGAAGAATAAGACTACAGTACATGGTTACTTCTCTTCAATTTTTCATGGTGGAGTGGATAGTC
TAGTCGACTTGATGAACAAGAGCTTTCCTGAGTTGGGTATTAAAAAAACTGATTGCAAAGAATTTAGCTGGTT
TGATACAACCATCTTCTACAGTGGTGTTGTAAATTTTAACACTGCTAATTTTAAAAAGGAAATTTTGCTTGAT
AGATCAGCTGGGAAGAAGACGGCTTTCTCAATTAAGTTAGACTATGTTAAGAAACCAATTCCAGAAACTGCAA
TGGTCAAAATTTTGGAAAAATTATATGAAGAAGATGTAGGAGCTGGGATGTATGTGTTGTACCCTTACGGTGG
TATAATGGAGGAGATTTCAGAATCAGCAATTCCATTCCCTCATCGAGCTGGAATAATGTATGAACTTTGGTAC
ACTGCTTCCTGGGAGAAGCAAGAAGATAATGAAAAGCATATAAACTGGGTTCGAAGTGTATATAATTTTACGA
CTCCTTATGTGTCCCAAAATCCAAGATTGGCGTATCTCAATTATAGGGACCTTGATTTTGGAAAAACTAATCC
TGCGAGTCCTAATAATTACACACAAGCACGTATTTGGGGTGAAAAGTATTTTGGTAAAAATTTTAACAGGTTA
GTTAAAGTGAAAACTAAAGTTGATCCCAATAATTTTTTTAGAAACGAACAAAGTATCCCACCTCTT
>A1_MN422084.1
TTTTGGTTTGTTTGCAAAATAATATTTTTCTTTCTCTCATTCCATATCCAAATTTCAATAGCTAATCCTCGAG
AAAACTTCCTTAAATGCTTCTCAAAACATATTCCCAACAATGTAGCAAATCCAAAATTCGTATACACTCAACA
CGACCAATTGTATATGTCTATCCTGAATTCGACAATACAAAATCTTAGATTCATCTCTGATACAACCCCAAAA
CCACTCGTTATTGTCACTCCTTCAAATATCTCCCATATCCAAGCAACTATTTTATGCTCTAAGAAAGTTGGCT
TGCAGATTCGAACTCGAAGCGGTGGGCATGATGCTGAGGGTATGTCCTACATTTCTCAACTCCCATTTGTTAT
AGTAGACTTGAGAAACATGCATTCGGTCAAAATAGATGTTCATAGCCAAACTGCGTGGGTTGAAGCCGGAGCT
ACCCTTGGAGAAGTTTATTATTGGATCAATGAGAAGAATGAGAATCTTAGTTTTCCTGGTGGGTATTGCCCTA
CTGTTGGCGTAGGTGGACACTTTAGTGGAGGAGGCTATGGAGCATTGATGCGAAATTATGGCCTTGCGGCTGA
TAATATTATTGATTCACACTTAGTCAATGTTGATGGAAAAGTTCTAGATCGAAAATCCATGGGAGAAGATCTG
TTTTGGGCTATACGTGGTGGTGGAGGAGAAAACTTTGGAATCATTGCAGCATGGAAAATCAAACTGGTTGTTG
TCCCATCAAAGTCTACTATATTCAGTGTTAAAAAGAACATGGAGATACATGGGCTTGTCAAGTTATTTAACAA
ATGGCAAAATATTGCTTACAAGTATGACAAAGATTTAGTACTCATGACTCACTTCAGAACAAAGAATATTACA
GATAATCATGGGAAGAATAAGACTACAGTACATGGTTACTTCTCTTCAATTTTTCATGGTGGAGTGGATAGTC
TAGTCGACTTGATGAACAAGAGCTTTCCTGAGTTGGGTATTAAAAAAACTGATTGCAAAGAATTTAGCTGGTT

TGATACAACCATCTTCTACAGTGGTGTTGTAAATTTTAACACTGCTAATTTTAAAAAGGAAATTTTGCTTGAT
AGATCAGCTGGGAAGAAGACGGCTTTCTCAATTAAGTTAGACTATGTTAAGAAACCAATTCCAGAAACTGCAA
TGGTCAAAATTTTGGAAAAATTATATGAAGAAGATGTAGGAGCTGGGATGTATGTGTTGTACCCTTACGGTGG
TATAATGGAGGAGATTTCAGAATCAGCAATTCCATTCCCTCATCGAGCTGGAATAATGTATGAACTTTGGTAC
ACTGCTTCCTGGGAGAAGCAAGAAGATAATGAAAAGCATATAAACTGGGTTCGAAGTGTATATAATTTTACGA
CTCCTTATGTGTCCCAAAATCCAAGATTGGCGTATCTCAATTATAGGGACCTTGATTTAGGAAAAACTAATCC
TGCGAGTCCTAATAATTACACACAAGCACGTATTTGGGGTGAAAAGTATTTTGGTAAAAATTTTAACAGGTTA
GTTAAAGTGAAAACTAAAGTTGATCCCAATAATTTTTTTAGAAACGAACAAAGTATCCCACCTCTT
>A1_MN422090.1
TTTTGGTTTGTTTGCAAAATAATATTTTTCTTTCTCTCATTCCATATCCAAATTTCATTAGCTAATCCTCGAG
AAAACTTCCTTAAATGCTTCTCAAAACATATTCCCAACAATGTAGCAAATCCAAAACTCGTATACACTCAACA
CGACCAATTGTATATGTCTATCCTGAATTCGACAATACAAAATCTTAGATTCATCTCTGATGCAACCCCAAAA
CCACTCGTTATTGTCACTCCTTCAAATAACTCCCATATCCAAGCAACTATTTTATGCTCTAAGAAAGTTGGTT
TGCAGATTCGAACTCGAAGCGGTGGCCATGATGCTGAGGGTATGTCCTACATTTCTCAACTCCCATTTGTTGT
AGTAGACTTGAGAAACATGCATTCGATCAAAATAGATGTTCATAGCCAAACTGCGTGGGTTGAAGCCGGAGCT
ACCCTTGGAGAAGTTTATTATTGGATCAATGAGAAGAATGAGAATCTTAGTTTTCCTGGTGGGTATTGCCCTA
CTGTTGGCGTAGGTGGACACTTTAGTGGAGGAGGCTATGGAGCATTGATGCGAAATTATGGCCTTGCGGCTGA
TAATATTATTGATGCACACTTAGTCAATGTTGATGGAAAAGTTCTAGATCGAAAATCCATGGGAGAAGATCTG
TTTTGGGCTATACGTGGTGGTGGAGGAGAAAACTTTGGAATCATTGCAGCATGGAAAATCAAACTGGTTGCTG
TCCCATCAAAGTCTACTATATTCAGTGTTAAAAAGAACATGGAGATACATGGGCTTGTCAAGTTATTTAACAA
ATGGCAAAATATTGCTTACAAGTATGACAAAGATTTAGTACTCATGACTCACTTCATAATAAAGAATATTACA
GATAATCATGGGAAGAATAAGACTACAGTACATGGTTACTTCTCTTCAATTTTTCATGGTGGAGTGGATAGTC
TAGTCGACTTGATGAACAAGAGCTTTCCTGAGTTGGGTATTAAAAAAACTGATTGCAAAGAATTTAGCTGGAT
TGATACAACCATCTTCTACAGTGGTGTTGTAAATTTTAACACTGCTAATTTTAAAAAGGAAATTTTGCTTGAT
AGATCAGCTGGGAAGAAGACGGCTTTCTCAATTAAGTTAGACTATGTTAAGAAACCAATTCCAGAAACTGCAA
TGGTCAAAATTTTGGAAAAATTATATGAAGAAGATGTAGGAGCTGGGATGTATGTGTTGTACCCTTACGGTGG
TATAATGGAGGAGATTTCGGAATCAGCAATTCCATTCCCTCATCGAGCTGGAATAATGTATGAACTTTGGTAC
ACTGCTTCCTGGGAGAAGCAAGAAGATAATGAAAAGCATATAAACTGGGTTCGAAGTGTATATAATTTTACGA
CTCCTTATGTGTCCCAAAATCCAAGATTGGCGTATCTCAATTATAGGGACCTTGATTTAGGAAAAACTAATCA
TGCGAGTCCTAATAATTACACACAAGCACGTATTTGGGGTGAAAAGTATTTTGGTAAAAATTTTATTAGGTTA
GTTAAAGTGAAAACTAAAGTTGATCCCAATAATTTTTTTAGAAACGAACAAAGTATCCCACCTCTT
>A1_Jamaican_Lion_JAATIP010000026.1_F8388_024901
ATGAATTGCTCAGCATTTTCCTTTTGGTTTGTTTGCAAAATAATATTTTTCTTTCTCTCATTCCATATCCAAA
TTTCAATAGCTAATCCTCGAGAAAACTTCCTTAAATGCTTCTCAAAACATATTCCCAACAATGTAGCAAATCC
AAAACTCGTATACACTCAACACGACCAATTGTATATGTCTATCCTGAATTCGACAATACAAAATCTTAGATTC
ATCTCTGATACAACCCCAAAACCACTCGTTATTGTCACTCCTTCAAATAACTCCCATATCCAAGCAACTATTT
TATGCTCTAAGAAAGTTGGCTTGCAGATTCGAACTCGAAGCGGTGGCCATGATGCTGAGGGTATGTCCTACAT
ATCTCAAGTCCCATTTGTTGTAGTAGACTTGAGAAACATGCATTCGATCAAAATAGATGTTCATAGCCAAACT
GCGTGGGTTGAAGCCGGAGCTACCCTTGGAGAAGTTTATTATTGGATCAATGAGAAGAATGAGAATCTTAGTT
TTCCTGGTGGGTATTGCCCTACTGTTGGCGTAGGTGGACACTTTAGTGGAGGAGGCTATGGAGCATTGATGCG
AAATTATGGCCTTGCGGCTGATAATATTATTGATGCACACTTAGTCAATGTTGATGGAAAAGTTCTAGATCGA
AAATCCATGGGAGAAGATCTGTTTTGGGCTATACGTGGTGGTGGAGGAGAAAACTTTGGAATCATTGCAGCAT
GGAAAATCAAACTGGTTGCTGTCCCATCAAAGTCTACTATATTCAGTGTTAAAAAGAACATGGAGATACATGG
GCTTGTCAAGTTATTTAACAAATGGCAAAATATTGCTTACAAGTATGACAAAGATTTAGTACTCATGACTCAC
TTCATAACAAAGAATATTACAGATAATCATGGGAAGAATAAGACTACAGTACATGGTTACTTCTCTTCAATTT
TTCATGGTGGAGTGGATAGTCTAGTCGACTTGATGAACAAGAGCTTTCCTGAGTTGGGTATTAAAAAAACTGA
TTGCAAAGAATTTAGCTGGATTGATACAACCATCTTCTACAGTGGTGTTGTAAATTTTAACACTGCTAATTTT
AAAAAGGAAATTTTGCTTGATAGATCAGCTGGGAAGAAGACGGCTTTCTCAATTAAGTTAGACTATGTTAAGA
AACCAATTCCAGAAACTGCAATGGTCAAAATTTTGGAAAAATTATATGAAGAAGATGTAGGAGCTGGGATGTA
TGTGTTGTACCCTTACGGTGGTATAATGGAGGAGATTTCAGAATCAGCAATTCCATTCCCTCATCGAGCTGGA
ATAATGTATGAACTTTGGTACACTGCTTCCTGGGAGAAGCAAGAAGATAATGAAAAGCATATAAACTGGGTTC
GAAGTGTTTATAATTTTACGACTCCTTATGTGTCCCAAAATCCAAGATTGGCGTATCTCAATTATAGGGACCT
TGATTTAGGAAAAACTAATCATGCGAGTCCTAATAATTACACACAAGCACGTATTTGGGGTGAAAAGTATTTT
GGTAAAAATTTTAACAGGTTAGTTAAGGTGAAAACTAAAGTTGATCCCAATAATTTTTTTAGAAACGAACAAA
GTATCCCACCTCTTCCACCGCATCATCATTAA
>A1_Purple_Kush_CM010797.2_28651687
ATGAATTGCTCAGCATTTTCCTTTTGGTTTGTTTGCAAAATAATATTTTTCTTTCTCTCATTCCATATCCAAA
TTTCAATAGCTAATCCTCGAGAAAACTTCCTTAAATGCTTCTCAAAACATATTCCCAACAATGTAGCAAATCC

AAAACTCGTATACACTCAACACGACCAATTGTATATGTCTATCCTGAATTCGACAATACAAAATCTTAGATTC
ATCTCTGATACAACCCCAAAACCACTCGTTATTGTCACTCCTTCAAATAACTCCCATATCCAAGCAACTATTT
TATGCTCTAAGAAAGTTGGCTTGCAGATTCGAACTCGAAGCGGTGGCCATGATGCTGAGGGTATGTCCTACAT
ATCTCAAGTCCCATTTGTTGTAGTAGACTTGAGAAACATGCATTCGATCAAAATAGATGTTCATAGCCAAACT
GCGTGGGTTGAAGCCGGAGCTACCCTTGGAGAAGTTTATTATTGGATCAATGAGAAGAATGAGAATCTTAGTT
TTCCTGGTGGGTATTGCCCTACTGTTGGCGTAGGTGGACACTTTAGTGGAGGAGGCTATGGAGCATTGATGCG
AAATTATGGCCTTGCGGCTGATAATATTATTGATGCACACTTAGTCAATGTTGATGGAAAAGTTCTAGATCGA
AAATCCATGGGAGAAGATCTGTTTTGGGCTATACGTGGTGGTGGAGGAGAAAACTTTGGAATCATTGCAGCAT
GGAAAATCAAACTGGTTGCTGTCCCATCAAAGTCTACTATATTCAGTGTTAAAAAGAACATGGAGATACATGG
GCTTGTCAAGTTATTTAACAAATGGCAAAATATTGCTTACAAGTATGACAAAGATTTAGTACTCATGACTCAC
TTCATAACAAAGAATATTACAGATAATCATGGGAAGAATAAGACTACAGTACATGGTTACTTCTCTTCAATTT
TTCATGGTGGAGTGGATAGTCTAGTCGACTTGATGAACAAGAGCTTTCGTGAGTTGGGTATTAAAAAAACTGA
TTGCAAAGAATTTAGCTGGATTGATACAACCATCTTCTACAGTGGTGTTGTAAATTTTAACACTGCTAATTTT
AAAAAGGAAATTTTGCTTGATAGATCAGCTGGGAAGAAGACGGCTTTCTCAATTAAGTTAGACTATGTTAAGA
AACCAATTCCAGAAACTGCAATGGTCAAAATTTTGGAAAAATTATATGAAGAAGATGTAGGAGCTGGGATGTA
TGTGTTGTACCCTTACGGTGGTATAATGGAGGAGATTTCAGAATCAGCAATTCCATTCCCTCATCGAGCTGGA
ATAATGTATGAACTTTGGTACACTGCTTCCTGGGAGAAGCAAGAAGATAATGAAAAGCATATAAACTGGGTTC
GAAGTGTTTATAATTTTACGACTCCTTATGTGTCCCAAAATCCAAGATTGGCGTATCTCAATTATAGGGACCT
TGATTTAGGAAAAACTAATCATGCGAGTCCTAATAATTACACACAAGCACGTATTTGGGGTGAAAAGTATTTT
GGTAAAAATTTTAACAGGTTAGTTAAGGTGAAAACTAAAGTTGATCCCAATAATTTTTTTAGAAACGAACAAA
GTATCCCACCTCTTCCACCGCATCATCATTAA
>A1_Jilong_CM022965.1_91579693
ATGAATTGCTCAGCATTTTCCTTTTGGTTTGTTTGCAAAATAATATTTTTCTTTCTCTCATTCCATATCCAAA
TTTCAATAGCTAATCCTCGAGAAAACTTCCTTAAATGCTTCTCAAAACATATTCCCAACAATGTAGCAAATCC
AAAACTCGTATACACTCAACACGACCAATTGTATATGTCTATCCTGAATTCGACAATACAAAATCTTAGATTC
ATCTCTGATACAACCCCAAAACCACTCGTTATTGTCACTCCTTCAAATAACTCCCATATCCAAGCAACTATTT
TATGCTCTAAGAAAGTTGGCTTGCAGATTCGAACTCGAAGCGGTGGCCATGATGCTGAGGGTATGTCCTACAT
ATCTCAAGTCCCATTTGTTGTAGTAGACTTGAGAAACATGCATTCGATCAAAATAGATGTTCATAGCCAAACT
GCGTGGGTTGAAGCCGGAGCTACCCTTGGAGAAGTTTATTATTGGATCAATGAGAAGAATGAGAATCTTAGTT
TTCCTGGTGGGTATTGCCCTACTGTTGGCGCAGGTGGACACTTTAGTGGAGGAGGCTATGGAGCATTGATGCG
AAATTATGGCCTCGCGGCTGATAATATTATTGATGCACACTTAGTCAATGTTGATGGAAAAGTTCTAGATCGA
AAATCCATGGGAGAAGATCTGTTTTGGGCTATACGTGGTGGTGGAGGAGAAAACTTTGGAATCATTGCAGCAT
GGAAAATCAAACTGGTTGCTGTCCCATCAAAGTCTACTATATTCAGTGTTAAAAAGAACATGGAGATACATGG
GCTTGTCAAGTTATTTAACAAATGGCAAAATATTGCTTACAAGTATGACAAAGATTTAGTACTCATGACTCAC
TTCATAACAAAGAATATTACAGATAATCATGGGAAGAATAAGACTACAGTACATGGTTACTTCTCTTCAATTT
TTCATGGTGGAGTGGATAGTCTAGTCGACTTGATGAACAAGAGCTTTCCTGAGTTGGGTATTAAAAAAACTGA
TTGCAAAGAATTTAGCTGGATTGATACAACCATCTTCTACAGTGGTGTTGTAAATTTTAACACTGCTAATTTT
AAAAAGGAAATTTTGCTTGATAGATCAGCTGGGCAGAAGACGGCTTTCTCAATTAAGTTAGACTATGTTAAGA
AACCAATTCCAGAAACCGCAATGGTCAAAATTTTGGAAAAATTATATGAAGAAGATGTAGGAGTTGGGATGTA
TGTGTTGTACCCTTACGGTGGTATAATGGATGAGATTTCAGAATCAGCAATTCCATTCCCTCATCGAGCTGGA
ATCATGTATGAACTTTGGTACACAGCTTCCTGGGAGAAGCAAGAAGATAATGAAAAGCATATAAACTGGGTTC
GAAGTGTTTATAATTTCACGACTCCTTATGTGTCCCAAAATCCAAGAATGGCGTATCTCAATTATAGGGACCT
TGATTTAGGAAAAACTAATCATGAGAGTCCTAATAATTACACACAAGCACGTATTTGGGGTGAAAAGTATTTT
GGTAAAAATTTTAACAGGTTAGTTAAGGTGAAAACCAAAGTTGATCCCAATAATTTTTTTAGAAACGAACAAA
GTATCCCACCTCTTCCACCGCATCATCATTAA
>A1_AB057805.1
ATGAATTGCTCAGCATTTTCCTTTTGGTTTGTTTGCAAAATAAT
ATTTTTCTTTCTCTCATTCCATATCCAAATTTCAATAGCTAATCCTCGAGAAAACTTCCTTAAATGCTTC
TCAAAACATATTCCCAACAATGTAGCAAATCCAAAACTCGTATACACTCAACACGACCAATTGTATATGT
CTATCCTGAATTCGACAATACAAAATCTTAGATTCATCTCTGATACAACCCCAAAACCACTCGTTATTGT
CACTCCTTCAAATAACTCCCATATCCAAGCAACTATTTTATGCTCTAAGAAAGTTGGCTTGCAGATTCGA
ACTCGAAGCGGTGGCCATGATGCTGAGGGTATGTCCTACATATCTCAAGTCCCATTTGTTGTAGTAGACT
TGAGAAACATGCATTCGATCAAAATAGATGTTCATAGCCAAACTGCGTGGGTTGAAGCCGGAGCTACCCT
TGGAGAAGTTTATTATTGGATCAATGAGAAGAATGAGAATCTTAGTTTTCCTGGTGGGTATTGCCCTACT
GTTGGCGTAGGTGGACACTTTAGTGGAGGAGGCTATGGAGCATTGATGCGAAATTATGGCCTTGCGGCTG
ATAATATTATTGATGCACACTTAGTCAATGTTGATGGAAAAGTTCTAGATCGAAAATCCATGGGAGAAGA
TCTGTTTTGGGCTATACGTGGTGGTGGAGGAGAAAACTTTGGAATCATTGCAGCATGGAAAATCAAACTG
GTTGCTGTCCCATCAAAGTCTACTATATTCAGTGTTAAAAAGAACATGGAGATACATGGGCTTGTCAAGT

TATTTAACAAATGGCAAAATATTGCTTACAAGTATGACAAAGATTTAGTACTCATGACTCACTTCATAAC
AAAGAATATTACAGATAATCATGGGAAGAATAAGACTACAGTACATGGTTACTTCTCTTCAATTTTTCAT
GGTGGAGTGGATAGTCTAGTCGACTTGATGAACAAGAGCTTTCCTGAGTTGGGTATTAAAAAAACTGATT
GCAAAGAATTTAGCTGGATTGATACAACCATCTTCTACAGTGGTGTTGTAAATTTTAACACTGCTAATTT
TAAAAAGGAAATTTTGCTTGATAGATCAGCTGGGAAGAAGACGGCTTTCTCAATTAAGTTAGACTATGTT
AAGAAACCAATTCCAGAAACTGCAATGGTCAAAATTTTGGAAAAATTATATGAAGAAGATGTAGGAGCTG
GGATGTATGTGTTGTACCCTTACGGTGGTATAATGGAGGAGATTTCAGAATCAGCAATTCCATTCCCTCA
TCGAGCTGGAATAATGTATGAACTTTGGTACACTGCTTCCTGGGAGAAGCAAGAAGATAATGAAAAGCAT
ATAAACTGGGTTCGAAGTGTTTATAATTTTACGACTCCTTATGTGTCCCAAAATCCAAGATTGGCGTATC
TCAATTATAGGGACCTTGATTTAGGAAAAACTAATCATGCGAGTCCTAATAATTACACACAAGCACGTAT
TTGGGGTGAAAAGTATTTTGGTAAAAATTTTAACAGGTTAGTTAAGGTGAAAACTAAAGTTGATCCCAAT
AATTTTTTTAGAAACGAACAAAGTATCCCACCTCTTCCACCGCATCATCATTAA
>A1_AB212832.1
ATGAATTGCTCAGCATTTTCCTTTTGGTTTGTTTGCAAAATAATATTTTTCTTTCTCTCATTCCATATCCAAA
TTTCAATAGCTAATCCTCGAGAAAACTTCCTTAAATGCTTCTCAAAACATATTCCCAACAATGTAGCAAATCC
AAAACTCGTATACACTCAACACGACCAATTGTATATGTCTATCCTGAATTCGACAATACAAAATCTTAGATTC
ATCTCTGATACAACCCCAAAACCACTCGTTATTGTCACTCCTTCAAATAACTCCCATATCCAAGCAACTATTT
TATGCTCTAAGAAAGTTGGCTTGCAGATTCGAACTCGAAGCGGTGGCCATGATGCTGAGGGTATGTCCTACAT
ATCTCAAGTCCCATTTGTTGTAGTAGACTTGAGAAACATGCATTCGATCAAAATAGATGTTCATAGCCAAACT
GCGTGGGTTGAAGCCGGAGCTACCCTTGGAGAAGTTTATTATTGGATCAATGAGAAGAATGAGAATCTTAGTT
TTCCTGGTGGGTATTGCCCTACTGTTGGCGTAGGTGGACACTTTAGTGGAGGAGGCTATGGAGCATTGATGCG
AAATTATGGCCTTGCGGCTGATAATATTATTGATGCACACTTAGTCAATGTTGATGGAAAAGTTCTAGATCGA
AAATCCATGGGAGAAGATCTGTTTTGGGCTATACGTGGTGGTGGAGGAGAAAACTTTGGAATCATTGCAGCAT
GGAAAATCAAACTGGTTGCTGTCCCATCAAAGTCTACTATATTCAGTGTTAAAAAGAACATGGAGATACATGG
GCTTGTCAAGTTATTTAACAAATGGCAAAATATTGCTTACAAGTATGACAAAGATTTAGTACTCATGACTCAC
TTCATAACAAAGAATATTACAGATAATCATGGGAAGAATAAGACTACAGTACATGGTTACTTCTCTTCAATTT
TTCATGGTGGAGTGGATAGTCTAGTCGACTTGATGAACAAGAGCTTTCCTGAGTTGGGTATTAAAAAAACTGA
TTGCAAAGAATTTAGCTGGATTGATACAACCATCTTCTACAGTGGTGTTGTAAATTTTAACACTGCTAATTTT
AAAAAGGAAATTTTGCTTGATAGATCAGCTGGGAAGAAGACGGCTTTCTCAATTAAGTTAGACTATGTTAAGA
AACCAATTCCAGAAACTGCAATGGTCAAAATTTTGGAAAAATTATATGAAGAAGATGTAGGAGCTGGGATGTA
TGTGTTGTACCCTTACGGTGGTATAATGGAGGAGATTTCAGAATCAGCAATTCCATTCCCTCATCGAGCTGGA
ATAATGTATGAACTTTGGTACACTGCTTCCTGGGAGAAGCAAGAAGATAATGAAAAGCATATAAACTGGGTTC
GAAGTGTTTATAATTTTACGACTCCTTATGTGTCCCAAAATCCAAGATTGGCGTATCTCAATTATAGGGACCT
TGATTTAGGAAAAACTAATCATGCGAGTCCTAATAATTACACACAAGCACGTATTTGGGGTGAAAAGTATTTT
GGTAAAAATTTTAACAGGTTAGTTAAGGTGAAAACTAAAGTTGATCCCAATAATTTTTTTAGAAACGAACAAA
GTATCCCACCTCTTCCACCGCATCATCAT
>A1_AB212834.1
ATGAATTGCTCAGCATTTTCCTTTTGGTTTGTTTGCAAAATAATATTTTTCTTTCTCTCATTCCATATCCAAA
TTTCAATAGCTAATCCTCGAGAAAACTTCCTTAAATGCTTCTCAAAACATATTCCCAACAATGTAGCAAATCC
AAAACTCGTATACACTCAACACGACCAATTGTATATGTCTATCCTGAATTCGACAATACAAAATCTTAGATTC
ATCTCTGATACAACCCCAAAACCACTCGTTATTGTCACTCCTTCAAATAACTCCCATATCCAAGCAACTATTT
TATGCTCTAAGAAAGTTGGCTTGCAGATTCGAACTCGAAGCGGTGGCCATGATGCTGAGGGTATGTCCTACAT
ATCTCAAGTCCCATTTGTTGTAGTAGACTTGAGAAACATGCATTCGATCAAAATAGATGTTCATAGCCAAACT
GCGTGGGTTGAAGCCGGAGCTACCCTTGGAGAAGTTTATTATTGGATCAATGAGAAGAATGAGAATCTTAGTT
TTCCTGGTGGGTATTGCCCTACTGTTGGCGTAGGTGGACACTTTAGTGGAGGAGGCTATGGAGCATTGATGCG
AAATTATGGCCTTGCGGCTGATAATATTATTGATGCACACTTAGTCAATGTTGATGGAAAAGTTCTAGATCGA
AAATCCATGGGAGAAGATCTGTTTTGGGCTATACGTGGTGGTGGAGGAGAAAACTTTGGAATCATTGCAGCAT
GGAAAATCAAACTGGTTGCTGTCCCATCAAAGTCTACTATATTCAGTGTTAAAAAGAACATGGAGATACATGG
GCTTGTCAAGTTATTTAACAAATGGCAAAATATTGCTTACAAGTATGACAAAGATTTAGTACTCATGACTCAC
TTCATAACAAAGAATATTACAGATAATCATGGGAAGAATAAGACTACAGTACATGGTTACTTCTCTTCAATTT
TTCATGGTGGAGTGGATAGTCTAGTCGACTTGATGAACAAGAGCTTTCCTGAGTTGGGTATTAAAAAAACTGA
TTGCAAAGAATTTAGCTGGATTGATACAACCATCTTCTACAGTGGTGTTGTAAATTTTAACACTGCTAATTTT
AAAAAGGAAATTTTGCTTGATAGATCAGCTGGGAAGAAGACGGCTTTCTCAATTAAGTTAGACTATGTTAAGA
AACCAATTCCAGAAACTGCAATGGTCAAAATTTTGGAAAAATTATATGAAGAAGATGTAGGAGCTGGGATGTA
TGTGTTGTACCCTTACGGTGGTATAATGGAGGAGATTTCAGAATCAGCAATTCCATTCCCTCATCGAGCTGGA
ATAATGTATGAACTTTGGTACACTGCTTCCTGGGAGAAGCAAGAAGATAATGAAAAGCATATAAACTGGGTTC
GAAGTGTTTATAATTTTACGACTCCTTATGTGTCCCAAAATCCAAGATTGGCGTATCTCAATTATAGGGACCT
TGATTTAGGAAAAACTAATCATGCGAGTCCTAATAATTACACACAAGCACGTATTTGGGGTGAAAAGTATTTT

GGTAAAAATTTTAACAGGTTAGTTAAGGTGAAAACTAAAGTTGATCCCAATAATTTTTTTAGAAACGAACAAA
GTATCCCACCTCTTCCACCGCATCATCAT
>A1_AB212835.1
ATGAATTGCTCAGCATTTTCCTTTTGGTTTGTTTGCAAAATAATATTTTTCTTTCTCTCATTCCATATCCAAA
TTTCAATAGCTAATCCTCGAGAAAACTTCCTTAAATGCTTCTCAAAACATATTCCCAACAATGTAGCAAATCC
AAAACTCGTATACACTCAACACGACCAATTGTATATGTCTATCCTGAATTCGACAATACAAAATCTTAGATTC
ATCTCTGATACAACCCCAAAACCACTCGTTATTGTCACTCCTTCAAATAACTCCCATATCCAAGCAACTATTT
TATGCTCTAAGAAAGTTGGCTTGCAGATTCGAACTCGAAGCGGTGGCCATGATGCTGAGGGTATGTCCTACAT
ATCTCAAGTCCCATTTGTTGTAGTAGACTTGAGAAACATGCATTCGATCAAAATAGATGTTCATAGCCAAACT
GCGTGGGTTGAAGCCGGAGCTACCCTTGGAGAAGTTTATTATTGGATCAATGAGAAGAATGAGAATCTTAGTT
TTCCTGGTGGGTATTGCCCTACTGTTGGCGTAGGTGGACACTTTAGTGGAGGAGGCTATGGAGCATTGATGCG
AAATTATGGCCTTGCGGCTGATAATATTATTGATGCACACTTAGTCAATGTTGATGGAAAAGTTCTAGATCGA
AAATCCATGGGAGAAGATCTGTTTTGGGCTATACGTGGTGGTGGAGGAGAAAACTTTGGAATCATTGCAGCAT
GGAAAATCAAACTGGTTGCTGTCCCATCAAAGTCTACTATATTCAGTGTTAAAAAGAACATGGAGATACATGG
GCTTGTCAAGTTATTTAACAAATGGCAAAATATTGCTTACAAGTATGACAAAGATTTAGTACTCATGACTCAC
TTCATAACAAAGAATATTACAGATAATCATGGGAAGAATAAGACTACAGTACATGGTTACTTCTCTTCAATTT
TTCATGGTGGAGTGGATAGTCTAGTCGACTTGATGAACAAGAGCTTTCCTGAGTTGGGTATTAAAAAAACTGA
TTGCAAAGAATTTAGCTGGATTGATACAACCATCTTCTACAGTGGTGTTGTAAATTTTAACACTGCTAATTTT
AAAAAGGAAATTTTGCTTGATAGATCAGCTGGGAAGAAGACGGCTTTCTCAATTAAGTTAGACTATGTTAAGA
AACCAATTCCAGAAACTGCAATGGTCAAAATTTTGGAAAAATTATATGAAGAAGATGTAGGAGCTGGGATGTA
TGTGTTGTACCCTTACGGTGGTATAATGGAGGAGATTTCAGAATCAGCAATTCCATTCCCTCATCGAGCTGGA
ATAATGTATGAACTTTGGTACACTGCTTCCTGGGAGAAGCAAGAAGATAATGAAAAGCATATAAACTGGGTTC
GAAGTGTTTATAATTTTACGACTCCTTATGTGTCCCAAAATCCAAGATTGGCGTATCTCAATTATAGGGACCT
TGATTTAGGAAAAACTAATCATGCGAGTCCTAATAATTACACACAAGCACGTATTTGGGGTGAAAAGTATTTT
GGTAAAAATTTTAACAGGTTAGTTAAGGTGAAAACTAAAGTTGATCCCAATAATTTTTTTAGAAACGAACAAA
GTATCCCACCTCTTCCACCGCATCATCAT
>A1_AB212837.1
ATGAATTGCTCAGCATTTTCCTTTTGGTTTGTTTGCAAAATAATATTTTTCTTTCTCTCATTCCATATCCAAA
TTTCAATAGCTAATCCTCGAGAAAACTTCCTTAAATGCTTCTCAAAACATATTCCCAACAATGTAGCAAATCC
AAAACTCGTATACACTCAACACGACCAATTGTATATGTCTATCCTGAATTCGACAATACAAAATCTTAGATTC
ATCTCTGATACAACCCCAAAACCACTCGTTATTGTCACTCCTTCAAATAACTCCCATATCCAAGCAACTATTT
TATGCTCTAAGAAAGTTGGCTTGCAGATTCGAACTCGAAGCGGTGGCCATGATGCTGAGGGTATGTCCTACAT
ATCTCAAGTCCCATTTGTTGTAGTAGACTTGAGAAACATGCATTCGATCAAAATAGATGTTCATAGCCAAACT
GCGTGGGTTGAAGCCGGAGCTACCCTTGGAGAAGTTTATTATTGGATCAATGAGAAGAATGAGAATCTTAGTT
TTCCTGGTGGGTATTGCCCTACTGTTGGCGTAGGTGGACACTTTAGTGGAGGAGGCTATGGAGCATTGATGCG
AAATTATGGCCTTGCGGCTGATAATATTATTGATGCACACTTAGTCAATGTTGATGGAAAAGTTCTAGATCGA
AAATCCATGGGAGAAGATCTGTTTTGGGCTATACGTGGTGGTGGAGGAGAAAACTTTGGAATCATTGCAGCAT
GGAAAATCAAACTGGTTGCTGTCCCATCAAAGTCTACTATATTCAGTGTTAAAAAGAACATGGAGATACATGG
GCTTGTCAAGTTATTTAACAAATGGCAAAATATTGCTTACAAGTATGACAAAGATTTAGTACTCATGACTCAC
TTCATAACAAAGAATATTACAGATAATCATGGGAAGAATAAGACTACAGTACATGGTTACTTCTCTTCAATTT
TTCATGGTGGAGTGGATAGTCTAGTCGACTTGATGAACAAGAGCTTTCCTGAGTTGGGTATTAAAAAAACTGA
TTGCAAAGAATTTAGCTGGATTGATACAACCATCTTCTACAGTGGTGTTGTAAATTTTAACACTGCTAATTTT
AAAAAGGAAATTTTGCTTGATAGATCAGCTGGGAAGAAGACGGCTTTCTCAATTAAGTTAGACTATGTTAAGA
AACCAATTCCAGAAACTGCAATGGTCAAAATTTTGGAAAAATTATATGAAGAAGATGTAGGAGCTGGGATGTA
TGTGTTGTACCCTTACGGTGGTATAATGGAGGAGATTTCAGAATCAGCAATTCCATTCCCTCATCGAGCTGGA
ATAATGTATGAACTTTGGTACACTGCTTCCTGGGAGAAGCAAGAAGATAATGAAAAGCATATAAACTGGGTTC
GAAGTGTTTATAATTTTACGACTCCTTATGTGTCCCAAAATCCAAGATTGGCGTATCTCAATTATAGGGACCT
TGATTTAGGAAAAACTAATCATGCGAGTCCTAATAATTACACACAAGCACGTATTTGGGGTGAAAAGTATTTT
GGTAAAAATTTTAACAGGTTAGTTAAGGTGAAAACTAAAGTTGATCCCAATAATTTTTTTAGAAACGAACAAA
GTATCCCACCTCTTCCACCGCATCATCAT
>A1_AB212838.1
ATGAATTGCTCAGCATTTTCCTTTTGGTTTGTTTGCAAAATAATATTTTTCTTTCTCTCATTCCATATCCAAA
TTTCAATAGCTAATCCTCGAGAAAACTTCCTTAAATGCTTCTCAAAACATATTCCCAACAATGTAGCAAATCC
AAAACTCGTATACACTCAACACGACCAATTGTATATGTCTATCCTGAATTCGACAATACAAAATCTTAGATTC
ATCTCTGATACAACCCCAAAACCACTCGTTATTGTCACTCCTTCAAATAACTCCCATATCCAAGCAACTATTT
TATGCTCTAAGAAAGTTGGCTTGCAGATTCGAACTCGAAGCGGTGGCCATGATGCTGAGGGTATGTCCTACAT
ATCTCAAGTCCCATTTGTTGTAGTAGACTTGAGAAACATGCATTCGATCAAAATAGATGTTCATAGCCAAACT
GCGTGGGTTGAAGCCGGAGCTACCCTTGGAGAAGTTTATTATTGGATCAATGAGAAGAATGAGAATCTTAGTT

TTCCTGGTGGGTATTGCCCTACTGTTGGCGTAGGTGGACACTTTAGTGGAGGAGGCTATGGAGCATTGATGCG
AAATTATGGCCTTGCGGCTGATAATATTATTGATGCACACTTAGTCAATGTTGATGGAAAAGTTCTAGATCGA
AAATCCATGGGAGAAGATCTGTTTTGGGCTATACGTGGTGGTGGAGGAGAAAACTTTGGAATCATTGCAGCAT
GGAAAATCAAACTGGTTGCTGTCCCATCAAAGTCTACTATATTCAGTGTTAAAAAGAACATGGAGATACATGG
GCTTGTCAAGTTATTTAACAAATGGCAAAATATTGCTTACAAGTATGACAAAGATTTAGTACTCATGACTCAC
TTCATAACAAAGAATATTACAGATAATCATGGGAAGAATAAGACTACAGTACATGGTTACTTCTCTTCAATTT
TTCATGGTGGAGTGGATAGTCTAGTCGACTTGATGAACAAGAGCTTTCCTGAGTTGGGTATTAAAAAAACTGA
TTGCAAAGAATTTAGCTGGATTGATACAACCATCTTCTACAGTGGTGTTGTAAATTTTAACACTGCTAATTTT
AAAAAGGAAATTTTGCTTGATAGATCAGCTGGGAAGAAGACGGCTTTCTCAATTAAGTTAGACTATGTTAAGA
AACCAATTCCAGAAACTGCAATGGTCAAAATTTTGGAAAAATTATATGAAGAAGATGTAGGAGCTGGGATGTA
TGTGTTGTACCCTTACGGTGGTATAATGGAGGAGATTTCAGAATCAGCAATTCCATTCCCTCATCGAGCTGGA
ATAATGTATGAACTTTGGTACACTGCTTCCTGGGAGAAGCAAGAAGATAATGAAAAGCATATAAACTGGGTTC
GAAGTGTTTATAATTTTACGACTCCTTATGTGTCCCAAAATCCAAGATTGGCGTATCTCAATTATAGGGACCT
TGATTTAGGAAAAACTAATCATGCGAGTCCTAATAATTACACACAAGCACGTATTTGGGGTGAAAAGTATTTT
GGTAAAAATTTTAACAGGTTAGTTAAGGTGAAAACTAAAGTTGATCCCAATAATTTTTTTAGAAACGAACAAA
GTATCCCACCTCTTCCACCGCATCATCAT
>A1_KT876012.1
ATGAATTGCTCAGCATTTTCCTTTTGGTTTGTTTGCAAAATAATATTTTTCTTTCTCTCATTCCATATCCAAA
TTTCAATAGCTAATCCTCGAGAAAACTTCCTTAAATGCTTCTCAAAACATATTCCCAACAATGTAGCAAATCC
AAAACTCGTATACACTCAACACGACCAATTGTATATGTCTATCCTGAATTCGACAATACAAAATCTTAGATTC
ATCTCTGATACAACCCCAAAACCACTCGTTATTGTCACTCCTTCAAATAACTCCCATATCCAAGCAACTATTT
TATGCTCTAAGAAAGTTGGCTTGCAGATTCGAACTCGAAGCGGTGGCCATGATGCTGAGGGTATGTCCTACAT
ATCTCAAGTCCCATTTGTTGTAGTAGACTTGAGAAACATGCATTCGATCAAAATAGATGTTCATAGCCAAACT
GCGTGGGTTGAAGCCGGAGCTACCCTTGGAGAAGTTTATTATTGGATCAATGAGAAGAATGAGAATCTTAGTT
TTCCTGGTGGGTATTGCCCTACTGTTGGCGTAGGTGGACACTTTAGTGGAGGAGGCTATGGAGCATTGATGCG
AAATTATGGCCTTGCGGCTGATAATATTATTGATGCACACTTAGTCAATGTTGATGGAAAAGTTCTAGATCGA
AAATCCATGGGAGAAGATCTGTTTTGGGCTATACGTGGTGGTGGAGGAGAAAACTTTGGAATCATTGCAGCAT
GGAAAATCAAACTGGTTGCTGTCCCATCAAAGTCTACTATATTCAGTGTTAAAAAGAACATGGAGATACATGG
GCTTGTCAAGTTATTTAACAAATGGCAAAATATTGCTTACAAGTATGACAAAGATTTAGTACTCATGACTCAC
TTCATAACAAAGAATATTACAGATAATCATGGGAAGAATAAGACTACAGTACATGGTTACTTCTCTTCAATTT
TTCATGGTGGAGTGGATAGTCTAGTCGACTTGATGAACAAGAGCTTTCCTGAGTTGGGTATTAAAAAAACTGA
TTGCAAAGAATTTAGCTGGATTGATACAACCATCTTCTACAGTGGTGTTGTAAATTTTAACACTGCTAATTTT
AAAAAGGAAATTTTGCTTGATAGATCAGCTGGGAAGAAGACGGCTTTCTCAATTAAGTTAGACTATGTTAAGA
AACCAATTCCAGAAACTGCAATGGTCAAAATTTTGGAAAAATTATATGAAGAAGATGTAGGAGCTGGGATGTA
TGTGTTGTACCCTTACGGTGGTATAATGGAGGAGATTTCAGAATCAGCAATTCCATTCCCTCATCGAGCTGGA
ATAATGTATGAACTTTGGTACACTGCTTCCTGGGAGAAGCAAGAAGATAATGAAAAGCATATAAACTGGGTTC
GAAGTGTTTATAATTTTACGACTCCTTATGTGTCCCAAAATCCAAGATTGGCGTATCTCAATTATAGGGACCT
TGATTTAGGAAAAACTAATCATGCGAGTCCTAATAATTACACACAAGCACGTATTTGGGGTGAAAAGTATTTT
GGTAAAAATTTTAACAGGTTAGTTAAGGTGAAAACTAAAGTTGATCCCAATAATTTTTTTAGAAACGAACAAA
GTATCCCACCTCTTCCACCGCATCATCAT
>A1_KT876016.1
ATGAATTGCTCAGCATTTTCCTTTTGGTTTGTTTGCAAAATAATATTTTTCTTTCTCTCATTCCATATCCAAA
TTTCAATAGCTAATCCTCGAGAAAACTTCCTTAAATGCTTCTCAAAACATATTCCCAACAATGTAGCAAATCC
AAAACTCGTATACACTCAACACGACCAATTGTATATGTCTATCCTGAATTCGACAATACAAAATCTTAGATTC
ATCTCTGATACAACCCCAAAACCACTCGTTATTGTCACTCCTTCAAATAACTCCCATATCCAAGCAACTATTT
TATGCTCTAAGAAAGTTGGCTTGCAGATTCGAACTCGAAGCGGTGGCCATGATGCTGAGGGTATGTCCTACAT
ATCTCAAGTCCCATTTGTTGTAGTAGACTTGAGAAACATGCATTCGATCAAAATAGATGTTCATAGCCAAACT
GCGTGGGTTGAAGCCGGAGCTACCCTTGGAGAAGTTTATTATTGGATCAATGAGAAGAATGAGAATCTTAGTT
TTCCTGGTGGGTATTGCCCTACTGTTGGCGTAGGTGGACACTTTAGTGGAGGAGGCTATGGAGCATTGATGCG
AAATTATGGCCTTGCGGCTGATAATATTATTGATGCACACTTAGTCAATGTTGATGGAAAAGTTCTAGATCGA
AAATCCATGGGAGAAGATCTGTTTTGGGCTATACGTGGTGGTGGAGGAGAAAACTTTGGAATCATTGCAGCAT
GGAAAATCAAACTGGTTGCTGTCCCATCAAAGTCTACTATATTCAGTGTTAAAAAGAACATGGAGATACATGG
GCTTGTCAAGTTATTTAACAAATGGCAAAATATTGCTTACAAGTATGACAAAGATTTAGTACTCATGACTCAC
TTCATAACAAAGAATATTACAGATAATCATGGGAAGAATAAGACTACAGTACATGGTTACTTCTCTTCAATTT
TTCATGGTGGAGTGGATAGTCTAGTCGACTTGATGAACAAGAGCTTTCCTGAGTTGGGTATTAAAAAAACTGA
TTGCAAAGAATTTAGCTGGATTGATACAACCATCTTCTACAGTGGTGTTGTAAATTTTAACACTGCTAATTTT
AAAAAGGAAATTTTGCTTGATAGATCAGCTGGGAAGAAGACGGCTTTCTCAATTAAGTTAGACTATGTTAAGA
AACCAATTCCAGAAACTGCAATGGTCAAAATTTTGGAAAAATTATATGAAGAAGATGTAGGAGCTGGGATGTA

TGTGTTGTACCCTTACGGTGGTATAATGGAGGAGATTTCAGAATCAGCAATTCCATTCCCTCATCGAGCTGGA
ATAATGTATGAACTTTGGTACACTGCTTCCTGGGAGAAGCAAGAAGATAATGAAAAGCATATAAACTGGGTTC
GAAGTGTTTATAATTTTACGACTCCTTATGTGTCCCAAAATCCAAGATTGGCGTATCTCAATTATAGGGACCT
TGATTTAGGAAAAACTAATCATGCGAGTCCTAATAATTACACACAAGCACGTATTTGGGGTGAAAAGTATTTT
GGTAAAAATTTTAACAGGTTAGTTAAGGTGAAAACTAAAGTTGATCCCAATAATTTTTTTAGAAACGAACAAA
GTATCCCACCTCTTCCACCGCATCATCAT
>A1_KT876018.1
ATGAATTGCTCAGCATTTTCCTTTTGGTTTGTTTGCAAAATAATATTTTTCTTTCTCTCATTCCATATCCAAA
TTTCAATAGCTAATCCTCGAGAAAACTTCCTTAAATGCTTCTCAAAACATATTCCCAACAATGTAGCAAATCC
AAAACTCGTATACACTCAACACGACCAATTGTATATGTCTATCCTGAATTCGACAATACAAAATCTTAGATTC
ATCTCTGATACAACCCCAAAACCACTCGTTATTGTCACTCCTTCAAATAACTCCCATATCCAAGCAACTATTT
TATGCTCTAAGAAAGTTGGCTTGCAGATTCGAACTCGAAGCGGTGGCCATGATGCTGAGGGTATGTCCTACAT
ATCTCAAGTCCCATTTGTTGTAGTAGACTTGAGAAACATGCATTCGATCAAAATAGATGTTCATAGCCAAACT
GCGTGGGTTGAAGCCGGAGCTACCCTTGGAGAAGTTTATTATTGGATCAATGAGAAGAATGAGAATCTTAGTT
TTCCTGGTGGGTATTGCCCTACTGTTGGCGTAGGTGGACACTTTAGTGGAGGAGGCTATGGAGCATTGATGCG
AAATTATGGCCTTGCGGCTGATAATATTATTGATGCACACTTAGTCAATGTTGATGGAAAAGTTCTAGATCGA
AAATCCATGGGAGAAGATCTGTTTTGGGCTATACGTGGTGGTGGAGGAGAAAACTTTGGAATCATTGCAGCAT
GGAAAATCAAACTGGTTGCTGTCCCATCAAAGTCTACTATATTCAGTGTTAAAAAGAACATGGAGATACATGG
GCTTGTCAAGTTATTTAACAAATGGCAAAATATTGCTTACAAGTATGACAAAGATTTAGTACTCATGACTCAC
TTCATAACAAAGAATATTACAGATAATCATGGGAAGAATAAGACTACAGTACATGGTTACTTCTCTTCAATTT
TTCATGGTGGAGTGGATAGTCTAGTCGACTTGATGAACAAGAGCTTTCCTGAGTTGGGTATTAAAAAAACTGA
TTGCAAAGAATTTAGCTGGATTGATACAACCATCTTCTACAGTGGTGTTGTAAATTTTAACACTGCTAATTTT
AAAAAGGAAATTTTGCTTGATAGATCAGCTGGGAAGAAGACGGCTTTCTCAATTAAGTTAGACTATGTTAAGA
AACCAATTCCAGAAACTGCAATGGTCAAAATTTTGGAAAAATTATATGAAGAAGATGTAGGAGCTGGGATGTA
TGTGTTGTACCCTTACGGTGGTATAATGGAGGAGATTTCAGAATCAGCAATTCCATTCCCTCATCGAGCTGGA
ATAATGTATGAACTTTGGTACACTGCTTCCTGGGAGAAGCAAGAAGATAATGAAAAGCATATAAACTGGGTTC
GAAGTGTTTATAATTTTACGACTCCTTATGTGTCCCAAAATCCAAGATTGGCGTATCTCAATTATAGGGACCT
TGATTTAGGAAAAACTAATCATGCGAGTCCTAATAATTACACACAAGCACGTATTTGGGGTGAAAAGTATTTT
GGTAAAAATTTTAACAGGTTAGTTAAGGTGAAAACTAAAGTTGATCCCAATAATTTTTTTAGAAACGAACAAA
GTATCCCACCTCTTCCACCGCATCATCAT
>A1_KT876027.1
ATGAATTGCTCAGCATTTTCCTTTTGGTTTGTTTGCAAAATAATATTTTTCTTTCTCTCATTCCATATCCAAA
TTTCAATAGCTAATCCTCGAGAAAACTTCCTTAAATGCTTCTCAAAACATATTCCCAACAATGTAGCAAATCC
AAAACTCGTATACACTCAACACGACCAATTGTATATGTCTATCCTGAATTCGACAATACAAAATCTTAGATTC
ATCTCTGATACAACCCCAAAACCACTCGTTATTGTCACTCCTTCAAATAACTCCCATATCCAAGCAACTATTT
TATGCTCTAAGAAAGTTGGCTTGCAGATTCGAACTCGAAGCGGTGGCCATGATGCTGAGGGTATGTCCTACAT
ATCTCAAGTCCCATTTGTTGTAGTAGACTTGAGAAACATGCATTCGATCAAAATAGATGTTCATAGCCAAACT
GCGTGGGTTGAAGCCGGAGCTACCCTTGGAGAAGTTTATTATTGGATCAATGAGAAGAATGAGAATCTTAGTT
TTCCTGGTGGGTATTGCCCTACTGTTGGCGTAGGTGGACACTTTAGTGGAGGAGGCTATGGAGCATTGATGCG
AAATTATGGCCTTGCGGCTGATAATATTATTGATGCACACTTAGTCAATGTTGATGGAAAAGTTCTAGATCGA
AAATCCATGGGAGAAGATCTGTTTTGGGCTATACGTGGTGGTGGAGGAGAAAACTTTGGAATCATTGCAGCAT
GGAAAATCAAACTGGTTGCTGTCCCATCAAAGTCTACTATATTCAGTGTTAAAAAGAACATGGAGATACATGG
GCTTGTCAAGTTATTTAACAAATGGCAAAATATTGCTTACAAGTATGACAAAGATTTAGTACTCATGACTCAC
TTCATAACAAAGAATATTACAGATAATCATGGGAAGAATAAGACTACAGTACATGGTTACTTCTCTTCAATTT
TTCATGGTGGAGTGGATAGTCTAGTCGACTTGATGAACAAGAGCTTTCCTGAGTTGGGTATTAAAAAAACTGA
TTGCAAAGAATTTAGCTGGATTGATACAACCATCTTCTACAGTGGTGTTGTAAATTTTAACACTGCTAATTTT
AAAAAGGAAATTTTGCTTGATAGATCAGCTGGGAAGAAGACGGCTTTCTCAATTAAGTTAGACTATGTTAAGA
AACCAATTCCAGAAACTGCAATGGTCAAAATTTTGGAAAAATTATATGAAGAAGATGTAGGAGCTGGGATGTA
TGTGTTGTACCCTTACGGTGGTATAATGGAGGAGATTTCAGAATCAGCAATTCCATTCCCTCATCGAGCTGGA
ATAATGTATGAACTTTGGTACACTGCTTCCTGGGAGAAGCAAGAAGATAATGAAAAGCATATAAACTGGGTTC
GAAGTGTTTATAATTTTACGACTCCTTATGTGTCCCAAAATCCAAGATTGGCGTATCTCAATTATAGGGACCT
TGATTTAGGAAAAACTAATCATGCGAGTCCTAATAATTACACACAAGCACGTATTTGGGGTGAAAAGTATTTT
GGTAAAAATTTTAACAGGTTAGTTAAGGTGAAAACTAAAGTTGATCCCAATAATTTTTTTAGAAACGAACAAA
GTATCCCACCTCTTCCACCGCATCATCAT
>A1_KT876047.1
ATGAATTGCTCAGCATTTTCCTTTTGGTTTGTTTGCAAAATAATATTTTTCTTTCTCTCATTCCATATCCAAA
TTTCAATAGCTAATCCTCGAGAAAACTTCCTTAAATGCTTCTCAAAACATATTCCCAACAATGTAGCAAATCC
AAAACTCGTATACACTCAACACGACCAATTGTATATGTCTATCCTGAATTCGACAATACAAAATCTTAGATTC

ATCTCTGATACAACCCCAAAACCACTCGTTATTGTCACTCCTTCAAATAACTCCCATATCCAAGCAACTATTT
TATGCTCTAAGAAAGTTGGCTTGCAGATTCGAACTCGAAGCGGTGGCCATGATGCTGAGGGTATGTCCTACAT
ATCTCAAGTCCCATTTGTTGTAGTAGACTTGAGAAACATGCATTCGATCAAAATAGATGTTCATAGCCAAACT
GCGTGGGTTGAAGCCGGAGCTACCCTTGGAGAAGTTTATTATTGGATCAATGAGAAGAATGAGAATCTTAGTT
TTCCTGGTGGGTATTGCCCTACTGTTGGCGTAGGTGGACACTTTAGTGGAGGAGGCTATGGAGCATTGATGCG
AAATTATGGCCTTGCGGCTGATAATATTATTGATGCACACTTAGTCAATGTTGATGGAAAAGTTCTAGATCGA
AAATCCATGGGAGAAGATCTGTTTTGGGCTATACGTGGTGGTGGAGGAGAAAACTTTGGAATCATTGCAGCAT
GGAAAATCAAACTGGTTGCTGTCCCATCAAAGTCTACTATATTCAGTGTTAAAAAGAACATGGAGATACATGG
GCTTGTCAAGTTATTTAACAAATGGCAAAATATTGCTTACAAGTATGACAAAGATTTAGTACTCATGACTCAC
TTCATAACAAAGAATATTACAGATAATCATGGGAAGAATAAGACTACAGTACATGGTTACTTCTCTTCAATTT
TTCATGGTGGAGTGGATAGTCTAGTCGACTTGATGAACAAGAGCTTTCCTGAGTTGGGTATTAAAAAAACTGA
TTGCAAAGAATTTAGCTGGATTGATACAACCATCTTCTACAGTGGTGTTGTAAATTTTAACACTGCTAATTTT
AAAAAGGAAATTTTGCTTGATAGATCAGCTGGGAAGAAGACGGCTTTCTCAATTAAGTTAGACTATGTTAAGA
AACCAATTCCAGAAACTGCAATGGTCAAAATTTTGGAAAAATTATATGAAGAAGATGTAGGAGCTGGGATGTA
TGTGTTGTACCCTTACGGTGGTATAATGGAGGAGATTTCAGAATCAGCAATTCCATTCCCTCATCGAGCTGGA
ATAATGTATGAACTTTGGTACACTGCTTCCTGGGAGAAGCAAGAAGATAATGAAAAGCATATAAACTGGGTTC
GAAGTGTTTATAATTTTACGACTCCTTATGTGTCCCAAAATCCAAGATTGGCGTATCTCAATTATAGGGACCT
TGATTTAGGAAAAACTAATCATGCGAGTCCTAATAATTACACACAAGCACGTATTTGGGGTGAAAAGTATTTT
GGTAAAAATTTTAACAGGTTAGTTAAGGTGAAAACTAAAGTTGATCCCAATAATTTTTTTAGAAACGAACAAA
GTATCCCACCTCTTCCACCGCATCATCAT
>A1_KT876005.1
ATGAATTGCTCAGCATTTTCCTTTTGGTTTGTTTGCAAAATAATATTTTTCTTTCTCTCATTCCATATCCAAA
TTTCAATAGCTAATCCTCGAGAAAACTTCCTTAAATGCTTCTCAAAACATATTCCCAACAATGTAGCAAATCC
AAAACTCGTATACACTCAACACGACCAATTGTATATGTCTATCCTGAATTCGACAATACAAAATCTTAGATTC
ATCTCTGATACAACCCCAAAACCACTCGTTATTGTCACTCCTTCAAATAACTCCCATATCCAAGCAACTATTT
TATGCTCTAAGAAAGTTGGCTTGCAGATTCGAACTCGAAGCGGTGGCCATGATGCTGAGGGTATGTCCTACAT
ATCTCAAGTCCCATTTGTTGTAGTAGACTTGAGAAACATGCATTCGATCAAAATAGATGTTCATAGCCAAACT
GCGTGGGTTGAAGCCGGAGCTACCCTTGGAGAAGTTTATTATTGGATCAATGAGAAGAATGAGAATCTTAGTT
TTCCTGGTGGGTATTGCCCTACTGTTGGCGTAGGTGGACACTTTAGTGGAGGAGGCTATGGAGCATTGATGCG
AAATTATGGCCTTGCGGCTGATAATATTATTGATGCACACTTAGTCAATGTTGATGGAAAAGTTCTAGATCGA
AAATCCATGGGAGAAGATCTGTTTTGGGCTATACGTGGTGGTGGAGGAGAAAACTTTGGAATCATTGCAGCAT
GGAAAATCAAACTGGTTGCTGTCCCATCAAAGTCTACTATATTCAGTGTTAAAAAGAACATGGAGATACATGG
GCTTGTCAAGTTATTTAACAAATGGCAAAATATTGCTTACAAGTATGACAAAGATTTAGTACTCATGACTCAC
TTCATAACAAAGAATATTACAGATAATCATGGGAAGAATAAGACTACAGTACATGGTTACTTCTCTTCAATTT
TTCATGGTGGAGTGGATAGTCTAGTCGACTTGATGAACAAGAGCTTTCCTGAGTTGGGTATTAAAAAAACTGA
TTGCAAAGAATTTAGCTGGATTGATACAACCATCTTCTACAGTGGTGTTGTAAATTTTAACACTGCTAATTTT
AAAAAGGAAATTTTGCTTGATAGATCAGCTGGGAAGAAGACGGCTTTCTCAATTAAGTTAGACTATGTTAAGA
AACCAATTCCAGAAACTGCAATGGTCAAAATTTTGGAAAAATTATATGAAGAAGATGTAGGAGCTGGGATGTA
TGTGTTGTACCCTTACGGTGGTATAATGGAGGAGATTTCAGAATCAGCAATTCCATTCCCTCATCGAGCTGGA
ATAATGTATGAACTTTGGTACACTGCTTCCTGGGAGAAGCAAGAAGATAATGAAAAGCATATAAACTGGGTTC
GAAGTGTTTATAATTTTACGACTCCTTATGTGTCCCAAAATCCAAGATTGGCGTATCTCAATTATAGGGACCT
TGATTTAGGAAAAACTAATCATGCGAGTCCTAATAATTACACACAAGCACGTATTTGGGGTGAAAAGTATTTT
GGTAAAAATTTTAACAGGTTAGTTAAGGTGAAAACTAAAGTTGATCCCAATAATTTTTTTAGAAACGAACAAA
GTATCCCACCTCTTCCACCGCATCAT
>A1_MG996418.1
TTTTGGTTTGTTTGCAAAATAATATTTTTCTTTCTCTCATTCCATATCCAAATTTCAATAGCTAATCCTCGAG
AAAACTTCCTTAAATGCTTCTCAAAACATATTCCCAACAATGTAGCAAATCCAAAACTCGTATACACTCAACA
CGACCAATTGTATATGTCTATCCTGAATTCGACAATACAAAATCTTAGATTCATCTCTGATACAACCCCAAAA
CCACTCGTTATTGTCACTCCTTCAAATAACTCCCATATCCAAGCAACTATTTTATGCTCTAAGAAAGTTGGCT
TGCAGATTCGAACTCGAAGCGGTGGCCATGATGCTGAGGGTATGTCCTACATATCTCAAGTCCCATTTGTTGT
AGTAGACTTGAGAAACATGCATTCGATCAAAATAGATGTTCATAGCCAAACTGCGTGGGTTGAAGCCGGAGCT
ACCCTTGGAGAAGTTTATTATTGGATCAATGAGAAGAATGAGAATCTTAGTTTTCCTGGTGGGTATTGCCCTA
CTGTTGGCGTAGGTGGACACTTTAGTGGAGGAGGCTATGGAGCATTGATGCGAAATTATGGCCTTGCGGCTGA
TAATATTATTGATGCACACTTAGTCAATGTTGATGGAAAAGTTCTAGATCGAAAATCCATGGGAGAAGATCTG
TTTTGGGCTATACGTGGTGGTGGAGGAGAAAACTTTGGAATCATTGCAGCATGGAAAATCAAACTGGTTGCTG
TCCCATCAAAGTCTACTATATTCAGTGTTAAAAAGAACATGGAGATACATGGGCTTGTCAAGTTATTTAACAA
ATGGCAAAATATTGCTTACAAGTATGACAAAGATTTAGTACTCATGACTCACTTCATAACAAAGAATATTACA
GATAATCATGGGAAGAATAAGACTACAGTACATGGTTACTTCTCTTCAATTTTTCATGGTGGAGTGGATAGTC

TAGTCGACTTGATGAACAAGAGCTTTCCTGAGTTGGGTATTAAAAAAACTGATTGCAAAGAATTTAGCTGGAT
TGATACAACCATCTTCTACAGTGGTGTTGTAAATTTTAACACTGCTAATTTTAAAAAGGAAATTTTGCTTGAT
AGATCAGCTGGGAAGAAGACGGCTTTCTCAATTAAGTTAGACTATGTTAAGAAACCAATTCCAGAAACTGCAA
TGGTCAAAATTTTGGAAAAATTATATGAAGAAGATGTAGGAGCTGGGATGTATGTGTTGTACCCTTACGGTGG
TATAATGGAGGAGATTTCAGAATCAGCAATTCCATTCCCTCATCGAGCTGGAATAATGTATGAACTTTGGTAC
ACTGCTTCCTGGGAGAAGCAAGAAGATAATGAAAAGCATATAAACTGGGTTCGAAGTGTTTATAATTTTACGA
CTCCTTATGTGTCCCAAAATCCAAGATTGGCGTATCTCAATTATAGGGACCTTGATTTAGGAAAAACTAATCA
TGCGAGTCCTAATAATTACACACAAGCACGTATTTGGGGTGAAAAGTATTTTGGTAAAAATTTTAACAGGTTA
GTTAAGGTGAAAACTAAAGTTGATCCCAATAATTTTTTTAGAAACGAACAAA
>A1_KP970849.1
ATGAATTGCTCAGCATTTTCCTTTTGGTTTGTTTGCAAAATAATATTTTTCTTTCTCTCATTCCATATCC
AAATTTCAATAGCTAACCCTCGAGAAAACTTCCTTAAATGCTTCTCAAAACATATTCCCAACAATGTAGC
AAATCCAAAACTCGTATACACTCAACACGACCAATTGTATATGTCTATCCTGAATTCGACAATACAAAAT
CTTAGATTCATCTCTGATACAACCCCAAAACCACTCGTTATTGTCACTCCTTCAAATAACTCCCATATCC
AAGCAACTATTTTATGCTCTAAGAAAGTTGGCTTGCAGATTCGAACTCGAAGCGGTGGCCATGATGCTGA
GGGTATGTCCTACATATCTCAAGTCCCATTTGTTGTAGTAGACTTGAGAAACATGCATTCGATCAAAATA
GATGTTCATAGCCAAACTGCGTGGGTTGAAGCCGGAGCTACCCTTGGAGAAGTTTATTATTGGATCAATG
AGAAGAATGAGAATCTTAGTTTTCCTGGTGGGTATTGCCCTACTGTTGGCGTAGGTGGACACTTTAGTGG
AGGAGGCTATGGAGCATTGATGCGAAATTATGGCCTTGCGGCTGATAATATTATTGATGCACACTTAGTC
AATGTTGATGGAAAAGTTCTAGATCGAAAATCCATGGGAGAAGATCTGTTTTGGGCTATACGTGGTGGTG
GAGGAGAAAACTTTGGAATCATTGCAGCATGGAAAATCAAACTGGTTGCTGTCCCATCAAAGTCTACTAT
ATTCAGTGTTAAAAAGAACATGGAGATACATGGGCTTGTCAAGTTATTTAACAAATGGCAAAATATTGCT
TACAAGTATGACAAAGATTTAGTACTCATGACTCACTTCATAACAAAGAATATTACAGATAATCATGGGA
AGAATAAGACTACAGTACATGGTTACTTCTCTTCAATTTTTCATGGTGGAGTGGATAGTCTAGTCGACTT
GATGAACAAGAGCTTTCCTGAGTTGGGTATTAAAAAAACTGATTGCAAAGAATTTAGCTGGATTGATACA
ACCATCTTCTACAGTGGTGTTGTAAATTTTAACACTGCTAATTTTAAAAAGGAAATTTTGCTTGATAGAT
CAGCTGGGAAGAAGACGGCTTTCTCAATTAAGTTAGACTATGTTAAGAAACCAATTCCAGAAACTGCAAT
GGTCAAAATTTTGGAAAAATTATATGAAGAAGATGTAGGAGCTGGGATGTATGTGTTGTACCCTTACGGT
GGTATAATGGAGGAGATTTCAGAATCAGCAATTCCATTCCCTCATCGAGCTGGAATAATGTATGAACTTT
GGTACACTGCTTCCTGGGAGAAGCAAGAAGATAATGAAAAGCATATAAACTGGGTTCGAAGTGTTTATAA
TTTTACGACTCCTTATGTGTCCCAAAATCCAAGATTGGCGTATCTCAATTATAGGGACCTTGATTTAGGA
AAAACTAATCATGCGAGTCCTAATAATTACACACAAGCACGTATTTGGGGTGAAAAGTATTTTGGTAAAA
ATTTTAACAGGTTAGTTAAGGTGAAAACTAAAGTTGATCCCAATAATTTTTTTAGAAACGAACAAAGTAT
CCCACCTCTTCCACCGCATCATCAT
>A1_KP970850.1
ATGAATTGCTCAGCATTTTCCTTTTGGTTTGTTTGCAAAATAATATTTTTCTTTCTCTCATTCCATATCC
AAATTTCAATAGCTAATCCTCGAGAAAACTTCCTTAAATGCTTCTCAAAACATATTCCCAACAATGTAGC
AAATCCAAAACTCGTATACACTCAACACGACCAATTGTATATGTCTATCCTGAATTCGACAATACAAAAT
CTTAGATTCATCTCTGATACAACCCCAAAACCACTCGTTATTGTCACTCCTTCAAATAACTCCCATATCC
AAGCAACTATTTTATGCTCTAAGAAAGTTGGCTTGCAGATTCGAACTCGAAGCGGTGGCCATGATGCTGA
GGGTATGTCCTACATATCTCAAGTCCCATTTGTTGTAGTAGACTTGAGAAACATGCATTCGATCAAAATA
GATGTTCATAGCCAAACTGCGTGGGTTGAAGCCGGAGCTACCCTTGGAGAAGTTTATTATTGGATCAATG
AGAAGAATGAGAATCTTAGTTTTCCTGGTGGGTATTGCCCTACTGTTGGCGTAGGTGGACACTTTAGTGG
AGGAGGCTATGGAGCATTGATGCGAAATTATGGCCTTGCGGCTGATAATATTATTGATGCACACTTAGTC
AATGTTGATGGAAAAGTTCTAGATCGAAAATCCATGGGAGAAGATCTGTTTTGGGCTATACGTGGTGGTG
GAGGACAAAACTTTGGAATCATTGCAGCATGGAAAATCAAACTGGTTGCTGTCCCATCAAAGTCTACTAT
ATTCAGTGTTAAAAAGAACATGGAGATACATGGGCTTGTCAAGTTATTTAACAAATGGCAAAATATTGCT
TACAAGTATGACAAAGATTTAGTACTCATGACTCACTTCATAACAAAGAATATTACAGATAATCATGGGA
AGAATAAGACTACAGTACATGGTTACTTCTCTTCAATTTTTCATGGTGGAGTGGATAGTCTAGTCGACTT
GATGAACAAGAGCTTTCCTGAGTTGGGTATTAAAAAAACTGATTGCAAAGAATTTAGCTGGATTGATACA
ACCATCTTCTACAGTGGTGTTGTAAATTTTAACACTGCTAATTTTAAAAAGGAAATTTTGCTTGATAGAT
CAGCTGGGAAGAAGACGGCTTTCTCAATTAAGTTAGACTATGTTAAGAAACCAATTCCAGAAACTGCAAT
GGTCAAAATTTTGGAAAAATTATATGAAGAAGATGTAGGAGCTGGGATGTATGTGTTGTACCCTTACGGT
GGTATAATGGAGGAGATTTCAGAATCAGCAATTCCATTCCCTCATCGAGCTGGAATAATGTATGAACTTT
GGTACACTGCTTCCTGGGAGAAGCAAGAAGATAATGAAAAGCATATAAACTGGGTTCGAAGTGTTTATAA
TTTTACGACTCCTTATGTGTCCCAAAATCCAAGATTGGCGTATCTCAATTATAGGGACCTTGATTTAGGA
AAAACTAATCATGCGAGTCCTAATAATTACACACAAGCACGTATTTGGGGTGAAAAGTATTTTGGTAAAA
ATTTTAACAGGTTAGTTAAGGTGAAAACTAAAGTTGATCCCAATAATTTTTTTAGAAACGAACAAAGTAT

CCCACCTCTTCCACCGCATCATCAT
>A1_KT876046.1
ATGAATTGCTCAGCATTTTCCTTTTGGTTTGTTTGCAAAATAATATTTTTCTTTCTCTCATTCCATATCCAAA
TTTCAATAGCTAATCCTCGAGAAAACTTCCTTAAATGCTTCTCAAAACATATTCCCAACAATGTAGCAAATCC
AAAACTCGTATACACTCAACACGACCAATTGTATATGTCTATCCTGAATTCGACAATACAAAATCTTAGATTC
ATCTCTGATACAACCCCAAAACCACTCGTTATTGTCACTCCTTCAAATAACTCCCATATCCAAGCAACTATTT
TATGCTCTAAGAAAGTTGGCTTGCAGATTCGAACTCGAAGCGGTGGCCATGATGCTGAGGGTATGTCCTACAT
ATCTCAAGTCCCATTTGTTGTAGTAGACTTGAGAAACATGCATTCGATCAAAATAGATGTTCATAGCCAAACT
GCGTGGGTTGAAGCCGGAGCTACCCTTGGAGAAGTTTATTATTGGATCAATGAGAAGAATGAGAATCTTAGTT
TTCCTGGTGGGTATTGCCCTACTGTTGGCGTAGGTGGACACTTTAGTGGAGGAGGCTATGGAGCATTGATGCG
AAATTATGGCCTTGCGGCTGATAATATTATTGATGCACACTTAGTCAATGTTGATGGAAAAGTTCTAGATCGA
AAATCCATGGGAGAAGATCTGTTTTGGGCTATACGTGGTGGTGGAGGAGAAAACTTTGGAATCATTGCAGCAT
GGAAAATCAAACTGGTTGCTGTCCCATCAAAGTCTACTATATTCAGTGTTAAAAAGAACATGGAGATACATGG
GCTTGTCAAGTTATTTAACAAATGGCAAAATATTGCTTACAAGTATGACAAAGATTTAGTACTCATGACTCAC
TTCATAACAAAGAATATTACAGATAATCATGGGAAGAATAAGACTACAGTACATGGTTACTTCTCTTCAATTT
TTCATGGTGGAGTGGATAGTCTAGTCGACTTGATGAACAAGAGCTTTCCTGAGTTGGGTATTAAAAAAACTGA
TTGCAAAGAATTTAGCTGGATTGATACAACCATCTTCTACAGTGGTGTTGTAAATTTTAACACTGCTAATTTT
AAAAAGGAAATTTTGCTTGATAGATCAGCTGGGAAGAAGACGGCTTTCTCAATTAAGTTAGACTATGTTAAGA
AACCAATTCCAGAAACTGCAATGGTCAAAATTTTGGAAAAATTATATGAAGAAGATGTAGGAGTTGGGATGTA
TGTGTTGTACCCTTACGGTGGTATAATGGAGGAGATTTCAGAATCAGCAATTCCATTCCCTCATCGAGCTGGA
ATAATGTATGAACTTTGGTACACTGCTTCCTGGGAGAAGCAAGAAGATAATGAAAAGCATATAAACTGGGTTC
GAAGTGTTTATAATTTTACGACTCCTTATGTGTCCCAAAATCCAAGATTGGCGTATCTCAATTATAGGGACCT
TGATTTAGGAAAAACTAATCATGCGAGTCCTAATAATTACACACAAGCACGTATTTGGGGTGAAAAGTATTTT
GGTAAAAATTTTAACAGGTTAGTTAAGGTGAAAACTAAAGTTGATCCCAATAATTTTTTTAGAAACGAACAAA
GTATCCCACCTCTTCCACCGCATCATCAT
>A1_KJ469378.1
ATGAATTGCTCAGCATTTTCCTTTTGGTTTGTTTGCAAAATAATATTTTTCTTTCTCTCATTCCATATCCAAA
TTTCAATAGCTAATCCTCGAGAAAACTTCCTTAAATGCTTCTCAAAACATATTCCCAACAATGTAGCAAATCC
AAAACTCGTATACACTCAACACGACCAATTGTATATGTCTATCCTGAATTCGACAATACAAAATCTTAGATTC
ATCTCTGATACAACCCCAAAACCACTCGTTATTGTCACTCCTTCAAATAACTCCCATATCCAAGCAACTATTT
TATGCTCTAAGAAAGTTGGCTTGCAGATTCGAACTCGAAGCGGTGGCCATGATGCTGAGGGTATGTCCTACAT
ATCTCAAGTCCCATTTGTTGTAGTAGACTTGAGAAACATGCATTCGATCAAAATAGATGTTCATAGCCAAACT
GCGTGGGTTGAAGCCGGAGCTACCCTTGGAGAAGTTTATTATTGGATCAATGAGAAGAATGAGAATCTTAGTT
TTCCTGGTGGGTATTGCCCTACTGTTGGCGTAGGTGGACACTTTAGTGGAGGAGGCTATGGAGCATTGATGCG
AAATTATGGCCTTGCGGCTGATAATATTATTGATGCACACTTAGTCAATGTTGATGGAAAAGTTCTAGATCGA
AAATCCATGGGAGAAGATCTGTTTTGGGCTATACGTGGTGGTGGAGGAGAAAACTTTGGAATCATTGCAGCAT
GGAAAATCAAACTGGTTGATGTCCCATCAAAGTCTACTATATTCAGTGTTAAAAAGAACATGGAGATACATGG
GCTTGTCAAGTTATTTAACAAATGGCAAAATATTGCTTACAAGTATGACAAAGATTTAGTACTCATGACTCAC
TTCATAACAAAGAATATTACAGATAATCATGGGAAGAATAAGACTACAGTACATGGTTACTTCTCTTCAATTT
TTCATGGTGGAGTGGATAGTCTAGTCGACTTGATGAACAAGAGCTTTCCTGAGTTGGGTATTAAAAAAACTGA
TTGCAAAGAATTTAGCTGGATTGATACAACCATCTTCTACAGTGGTGTTGTAAATTTTAACACTGCTAATTTT
AAAAAGGAAATTTTGCTTGATAGATCAGCTGGGAAGAAGACGGCTTTCTCAATTAAGTTAGACTATGTTAAGA
AACCAATTCCAGAAACTGCAATGGTCAAAATTTTGGAAAAATTATATGAAGAAGATGTAGGAGCTGGGATGTA
TGTGTTGTACCCTTACGGTGGTATAATGGAGGAGATTTCAGAATCAGCAATTCCATTCCCTCATCGAGCTGGA
ATAATGTATGAACTTTGGTACACTGCTTCCTGGGAGAAGCAAGAAGATAATGAAAAGCATATAAACTGGGTTC
GAAGTGTTTATAATTTTACGACTCCTTATGTGTCCCAAAATCCAAGATTGGCGTATCTCAATTATAGGGACCT
TGATTTAGGAAAAACTAATCATGCGAGTCCTAATAATTACACACAAGCACGTATTTGGGGTGAAAAGTATTTT
GGTAAAAATTTTAACAGGTTAGTTAAGGTGAAAACTAAAGTTGATCCCAATAATTTTTTTAGAAACGAACAAA
GTATCCCACCTCTTCCACCGCATCATCATTAA
>A1_KP970856.1
ATGAATTGCTCAGCATTTTCCTTTTGGTTTGTTTGCAAAATAATATTTTTCTTTCTCTCATTCCATATCC
AAATTTCAATAGCTAATCCTCGAGAAAACTTCCTTAAATGCTTCTCAAAACATATTCCCAACAATGTAGC
AAATCCAAAACTCGTATACACTCAACACGACCAATTGTATATGTCTATCCTGAATTCGACAATACAAAAT
CTTAGATTCATCTCTGATACAACCCCAAAACCACTCGTTATTGTCACTCCTTCAAATAACTCCCATATCC
AAGCAACTATTTTATGCTCTAAGAAAGTTGGCTTGCAGATTCGAACTCGAAGCGGTGGCCATGATGCTGA
GGGTATGTCCTACATATCTCAAGTCCCATTTGTTGTAGTAGACTTGAGAAACATGCATTCGATCAAAATA
GATGTTCATAGCCAAACTGCGTGGGTTGAAGCCGGAGCTACCCTTGGAGAAGTTTATTATTGGATCAATG
AGAAGAATGAGAATCTTAGTTTTCCTGGTGGGTATTGCCCTACTGTTGGCGTAGGTGGACACTTTAGTGG

AGGAGGCTATGGAGCATTGATGCGAAATTATGGCCTTGCGGCTGATAATATTATTGATGCACACTTAGTC
AATGTTGATGGAAAAGTTCTAGATCGAAAATCCATGGGAGAAGATCTGTTTTGGGCTATACGTGGTGGTG
GAGGAGAAAACTTTGGAATCATTGCAGCATGGAAAATCAAACTGGTTGATGTCCCATCAAAGTCTACTAT
ATTCAGTGTTAAAAAGAACATGGAGATACATGGGCTTGTCAAGTTATTTAACAAATGGCAAAATATTGCT
TACAAGTATGACAAAGATTTAGTACTCATGACTCACTTCATAACAAAGAATATTACAGATAATCATGGGA
AGAATAAGACTACAGTACATGGTTACTTCTCTTCAATTTTTCATGGTGGAGTGGATAGTCTAGTCGACTT
GATGAACAAGAGCTTTCCTGAGTTGGGTATTAAAAAAACTGATTGCAAAGAATTTAGCTGGATTGATACA
ACCATCTTCTACAGTGGTGTTGTAAATTTTAACACTGCTAATTTTAAAAAGGAAATTTTGCTTGATAGAT
CAGCTGGGAAGAAGACGGCTTTCTCAATTAAGTTAGACTATGTTAAGAAACCAATTCCAGAAACTGCAAT
GGTCAAAATTTTGGAAAAATTATATGAAGAAGATGTAGGAGCTGGGATGTATGTGTTGTACCCTTACGGT
GGTATAATGGAGGAGATTTCAGAATCAGCAATTCCATTCCCTCATCGAGCTGGAATAATGTATGAACTTT
GGTACACTGCTTCCTGGGAGAAGCAAGAAGATAATGAAAAGCATATAAACTGGGTTCGAAGTGTTTATAA
TTTTACGACTCCTTATGTGTCCCAAAATCCAAGATTGGCGTATCTCAATTATAGGGACCTTGATTTAGGA
AAAACTAATCATGCGAGTCCTAATAATTACACACAAGCACGTATTTGGGGTGAAAAGTATTTTGGTAAAA
ATTTTAACAGGTTAGTTAAGGTGAAAACTAAAGTTGATCCCAATAATTTTTTTAGAAACGAACAAAGTAT
CCCACCTCTTCCACCGCATCATCAT
>A1_KT876009.1
ATGAATTGCTCAGCATTTTCCTTTTGGTTTGTTTGCAAAATAATATTTTTCTTTCTCTCATTCCATATCCAAA
TTTCAATAGCTAATCCTCGAGAAAACTTCCTTAAATGCTTCTCAAAACATATTCCCAACAATGTAGCAAATCC
AAAACTCGTATACACTCAACACGACCAATTGTATATGTCTATCCTGAATTCGACAATACAAAATCTTAGATTC
ATCTCTGATACAACCCCAAAACCACTCGTTATTGTCACTCCTTCAAATAACTCCCATATCCAAGCAACTATTT
TATGCTCTAAGAAAGTTGGCTTGCAGATTCGAACTCGAAGCGGTGGCCATGATGCTGAGGGTATGTCCTACAT
ATCTCAAGTCCCATTTGTTGTAGTAGACTTGAGAAACATGCATTCGATCAAAATAGATGTTCATAGCCAAACT
GCGTGGGTTGAAGCCGGAGCTACCCTTGGAGAAGTTTATTATTGGATCAATGAGAAGAATGAGAATCTTAGTT
TTCCTGGTGGGTATTGCCCTACTGTTGGCGTAGGTGGACACTTTAGTGGAGGAGGCTATGGAGCATTGATGCG
AAATTATGGCCTTGCGGCTGATAATATTATTGATGCACACTTAGTCAATGTTGATGGAAAAGTTCTAGATCGA
AAATCCATGGGAGAAGATCTGTTTTGGGCTATACGTGGTGGTGGAGGAGAAAACTTTGGAATCATTGCAGCAT
GGAAAATCAAACTGGTTGATGTCCCATCAAAGTCTACTATATTCAGTGTTAAAAAGAACATGGAGATACATGG
GCTTGTCAAGTTATTTAACAAATGGCAAAATATTGCTTACAAGTATGACAAAGATTTAGTACTCATGACTCAC
TTCATAACAAAGAATATTACAGATAATCATGGGAAGAATAAGACTACAGTACATGGTTACTTCTCTTCAATTT
TTCATGGTGGAGTGGATAGTCTAGTCGACTTGATGAACAAGAGCTTTCCTGAGTTGGGTATTAAAAAAACTGA
TTGCAAAGAATTTAGCTGGATTGATACAACCATCTTCTACAGTGGTGTTGTAAATTTTAACACTGCTAATTTT
AAAAAGGAAATTTTGCTTGATAGATCAGCTGGGAAGAAGACGGCTTTCTCAATTAAGTTAGACTATGTTAAGA
AACCAATTCCAGAAACTGCAATGGTCAAAATTTTGGAAAAATTATATGAAGAAGATGTAGGAGCTGGGATGTA
TGTGTTGTACCCTTACGGTGGTATAATGGAGGAGATTTCAGAATCAGCAATTCCATTCCCTCATCGAGCTGGA
ATAATGTATGAACTTTGGTACACTGCTTCCTGGGAGAAGCAAGAAGATAATGAAAAGCATATAAACTGGGTTC
GAAGTGTTTATAATTTTACGACTCCTTATGTGTCCCAAAATCCAAGATTGGCGTATCTCAATTATAGGGACCT
TGATTTAGGAAAAACTAATCATGCGAGTCCTAATAATTACACACAAGCACGTATTTGGGGTGAAAAGTATTTT
GGTAAAAATTTTAACAGGTTAGTTAAGGTGAAAACTAAAGTTGATCCCAATAATTTTTTTAGAAACGAACAAA
GTATCCCACCTCTTCCACCGCATCATCAT
>A1_KT876022.1
ATGAATTGCTCAGCATTTTCCTTTTGGTTTGTTTGCAAAATAATATTTTTCTTTCTCTCATTCCATATCCAAA
TTTCAATAGCTAATCCTCGAGAAAACTTCCTTAAATGCTTCTCAAAACATATTCCCAACAATGTAGCAAATCC
AAAACTCGTATACACTCAACACGACCAATTGTATATGTCTATCCTGAATTCGACAATACAAAATCTTAGATTC
ATCTCTGATACAACCCCAAAACCACTCGTTATTGTCACTCCTTCAAATAACTCCCATATCCAAGCAACTATTT
TATGCTCTAAGAAAGTTGGCTTGCAGATTCGAACTCGAAGCGGTGGCCATGATGCTGAGGGTATGTCCTACAT
ATCTCAAGTCCCATTTGTTGTAGTAGACTTGAGAAACATGCATTCGATCAAAATAGATGTTCATAGCCAAACT
GCGTGGGTTGAAGCCGGAGCTACCCTTGGAGAAGTTTATTATTGGATCAATGAGAAGAATGAGAATCTTAGTT
TTCCTGGTGGGTATTGCCCTACTGTTGGCGTAGGTGGACACTTTAGTGGAGGAGGCTATGGAGCATTGATGCG
AAATTATGGCCTTGCGGCTGATAATATTATTGATGCACACTTAGTCAATGTTGATGGAAAAGTTCTAGATCGA
AAATCCATGGGAGAAGATCTGTTTTGGGCTATACGTGGTGGTGGAGGAGAAAACTTTGGAATCATTGCAGCAT
GGAAAATCAAACTGGTTGATGTCCCATCAAAGTCTACTATATTCAGTGTTAAAAAGAACATGGAGATACATGG
GCTTGTCAAGTTATTTAACAAATGGCAAAATATTGCTTACAAGTATGACAAAGATTTAGTACTCATGACTCAC
TTCATAACAAAGAATATTACAGATAATCATGGGAAGAATAAGACTACAGTACATGGTTACTTCTCTTCAATTT
TTCATGGTGGAGTGGATAGTCTAGTCGACTTGATGAACAAGAGCTTTCCTGAGTTGGGTATTAAAAAAACTGA
TTGCAAAGAATTTAGCTGGATTGATACAACCATCTTCTACAGTGGTGTTGTAAATTTTAACACTGCTAATTTT
AAAAAGGAAATTTTGCTTGATAGATCAGCTGGGAAGAAGACGGCTTTCTCAATTAAGTTAGACTATGTTAAGA
AACCAATTCCAGAAACTGCAATGGTCAAAATTTTGGAAAAATTATATGAAGAAGATGTAGGAGCTGGGATGTA

TGTGTTGTACCCTTACGGTGGTATAATGGAGGAGATTTCAGAATCAGCAATTCCATTCCCTCATCGAGCTGGA
ATAATGTATGAACTTTGGTACACTGCTTCCTGGGAGAAGCAAGAAGATAATGAAAAGCATATAAACTGGGTTC
GAAGTGTTTATAATTTTACGACTCCTTATGTGTCCCAAAATCCAAGATTGGCGTATCTCAATTATAGGGACCT
TGATTTAGGAAAAACTAATCATGCGAGTCCTAATAATTACACACAAGCACGTATTTGGGGTGAAAAGTATTTT
GGTAAAAATTTTAACAGGTTAGTTAAGGTGAAAACTAAAGTTGATCCCAATAATTTTTTTAGAAACGAACAAA
GTATCCCACCTCTTCCACCGCATCATCAT
>A1_KT876026.1
ATGAATTGCTCAGCATTTTCCTTTTGGTTTGTTTGCAAAATAATATTTTTCTTTCTCTCATTCCATATCCAAA
TTTCAATAGCTAATCCTCGAGAAAACTTCCTTAAATGCTTCTCAAAACATATTCCCAACAATGTAGCAAATCC
AAAACTCGTATACACTCAACACGACCAATTGTATATGTCTATCCTGAATTCGACAATACAAAATCTTAGATTC
ATCTCTGATACAACCCCAAAACCACTCGTTATTGTCACTCCTTCAAATAACTCCCATATCCAAGCAACTATTT
TATGCTCTAAGAAAGTTGGCTTGCAGATTCGAACTCGAAGCGGTGGCCATGATGCTGAGGGTATGTCCTACAT
ATCTCAAGTCCCATTTGTTGTAGTAGACTTGAGAAACATGCATTCGATCAAAATAGATGTTCATAGCCAAACT
GCGTGGGTTGAAGCCGGAGCTACCCTTGGAGAAGTTTATTATTGGATCAATGAGAAGAATGAGAATCTTAGTT
TTCCTGGTGGGTATTGCCCTACTGTTGGCGTAGGTGGACACTTTAGTGGAGGAGGCTATGGAGCATTGATGCG
AAATTATGGCCTTGCGGCTGATAATATTATTGATGCACACTTAGTCAATGTTGATGGAAAAGTTCTAGATCGA
AAATCCATGGGAGAAGATCTGTTTTGGGCTATACGTGGTGGTGGAGGAGAAAACTTTGGAATCATTGCAGCAT
GGAAAATCAAACTGGTTGATGTCCCATCAAAGTCTACTATATTCAGTGTTAAAAAGAACATGGAGATACATGG
GCTTGTCAAGTTATTTAACAAATGGCAAAATATTGCTTACAAGTATGACAAAGATTTAGTACTCATGACTCAC
TTCATAACAAAGAATATTACAGATAATCATGGGAAGAATAAGACTACAGTACATGGTTACTTCTCTTCAATTT
TTCATGGTGGAGTGGATAGTCTAGTCGACTTGATGAACAAGAGCTTTCCTGAGTTGGGTATTAAAAAAACTGA
TTGCAAAGAATTTAGCTGGATTGATACAACCATCTTCTACAGTGGTGTTGTAAATTTTAACACTGCTAATTTT
AAAAAGGAAATTTTGCTTGATAGATCAGCTGGGAAGAAGACGGCTTTCTCAATTAAGTTAGACTATGTTAAGA
AACCAATTCCAGAAACTGCAATGGTCAAAATTTTGGAAAAATTATATGAAGAAGATGTAGGAGCTGGGATGTA
TGTGTTGTACCCTTACGGTGGTATAATGGAGGAGATTTCAGAATCAGCAATTCCATTCCCTCATCGAGCTGGA
ATAATGTATGAACTTTGGTACACTGCTTCCTGGGAGAAGCAAGAAGATAATGAAAAGCATATAAACTGGGTTC
GAAGTGTTTATAATTTTACGACTCCTTATGTGTCCCAAAATCCAAGATTGGCGTATCTCAATTATAGGGACCT
TGATTTAGGAAAAACTAATCATGCGAGTCCTAATAATTACACACAAGCACGTATTTGGGGTGAAAAGTATTTT
GGTAAAAATTTTAACAGGTTAGTTAAGGTGAAAACTAAAGTTGATCCCAATAATTTTTTTAGAAACGAACAAA
GTATCCCACCTCTTCCACCGCATCATCAT
>A1_KT876037.1
ATGAATTGCTCAGCATTTTCCTTTTGGTTTGTTTGCAAAATAATATTTTTCTTTCTCTCATTCCATATCCAAA
TTTCAATAGCTAATCCTCGAGAAAACTTCCTTAAATGCTTCTCAAAACATATTCCCAACAATGTAGCAAATCC
AAAACTCGTATACACTCAACACGACCAATTGTATATGTCTATCCTGAATTCGACAATACAAAATCTTAGATTC
ATCTCTGATACAACCCCAAAACCACTCGTTATTGTCACTCCTTCAAATAACTCCCATATCCAAGCAACTATTT
TATGCTCTAAGAAAGTTGGCTTGCAGATTCGAACTCGAAGCGGTGGCCATGATGCTGAGGGTATGTCCTACAT
ATCTCAAGTCCCATTTGTTGTAGTAGACTTGAGAAACATGCATTCGATCAAAATAGATGTTCATAGCCAAACT
GCGTGGGTTGAAGCCGGAGCTACCCTTGGAGAAGTTTATTATTGGATCAATGAGAAGAATGAGAATCTTAGTT
TTCCTGGTGGGTATTGCCCTACTGTTGGCGTAGGTGGACACTTTAGTGGAGGAGGCTATGGAGCATTGATGCG
AAATTATGGCCTTGCGGCTGATAATATTATTGATGCACACTTAGTCAATGTTGATGGAAAAGTTCTAGATCGA
AAATCCATGGGAGAAGATCTGTTTTGGGCTATACGTGGTGGTGGAGGAGAAAACTTTGGAATCATTGCAGCAT
GGAAAATCAAACTGGTTGATGTCCCATCAAAGTCTACTATATTCAGTGTTAAAAAGAACATGGAGATACATGG
GCTTGTCAAGTTATTTAACAAATGGCAAAATATTGCTTACAAGTATGACAAAGATTTAGTACTCATGACTCAC
TTCATAACAAAGAATATTACAGATAATCATGGGAAGAATAAGACTACAGTACATGGTTACTTCTCTTCAATTT
TTCATGGTGGAGTGGATAGTCTAGTCGACTTGATGAACAAGAGCTTTCCTGAGTTGGGTATTAAAAAAACTGA
TTGCAAAGAATTTAGCTGGATTGATACAACCATCTTCTACAGTGGTGTTGTAAATTTTAACACTGCTAATTTT
AAAAAGGAAATTTTGCTTGATAGATCAGCTGGGAAGAAGACGGCTTTCTCAATTAAGTTAGACTATGTTAAGA
AACCAATTCCAGAAACTGCAATGGTCAAAATTTTGGAAAAATTATATGAAGAAGATGTAGGAGCTGGGATGTA
TGTGTTGTACCCTTACGGTGGTATAATGGAGGAGATTTCAGAATCAGCAATTCCATTCCCTCATCGAGCTGGA
ATAATGTATGAACTTTGGTACACTGCTTCCTGGGAGAAGCAAGAAGATAATGAAAAGCATATAAACTGGGTTC
GAAGTGTTTATAATTTTACGACTCCTTATGTGTCCCAAAATCCAAGATTGGCGTATCTCAATTATAGGGACCT
TGATTTAGGAAAAACTAATCATGCGAGTCCTAATAATTACACACAAGCACGTATTTGGGGTGAAAAGTATTTT
GGTAAAAATTTTAACAGGTTAGTTAAGGTGAAAACTAAAGTTGATCCCAATAATTTTTTTAGAAACGAACAAA
GTATCCCACCTCTTCCACCGCATCATCAT
>A1_KT876044.1
ATGAATTGCTCAGCATTTTCCTTTTGGTTTGTTTGCAAAATAATATTTTTCTTTCTCTCATTCCATATCCAAA
TTTCAATAGCTAATCCTCGAGAAAACTTCCTTAAATGCTTCTCAAAACATATTCCCAACAATGTAGCAAATCC
AAAACTCGTATACACTCAACACGACCAATTGTATATGTCTATCCTGAATTCGACAATACAAAATCTTAGATTC

ATCTCTGATACAACCCCAAAACCACTCGTTATTGTCACTCCTTCAAATAACTCCCATATCCAAGCAACTATTT
TATGCTCTAAGAAAGTTGGCTTGCAGATTCGAACTCGAAGCGGTGGCCATGATGCTGAGGGTATGTCCTACAT
ATCTCAAGTCCCATTTGTTGTAGTAGACTTGAGAAACATGCATTCGATCAAAATAGATGTTCATAGCCAAACT
GCGTGGGTTGAAGCCGGAGCTACCCTTGGAGAAGTTTATTATTGGATCAATGAGAAGAATGAGAATCTTAGTT
TTCCTGGTGGGTATTGCCCTACTGTTGGCGTAGGTGGACACTTTAGTGGAGGAGGCTATGGAGCATTGATGCG
AAATTATGGCCTTGCGGCTGATAATATTATTGATGCACACTTAGTCAATGTTGATGGAAAAGTTCTAGATCGA
AAATCCATGGGAGAAGATCTGTTTTGGGCTATACGTGGTGGTGGAGGAGAAAACTTTGGAATCATTGCAGCAT
GGAAAATCAAACTGGTTGATGTCCCATCAAAGTCTACTATATTCAGTGTTAAAAAGAACATGGAGATACATGG
GCTTGTCAAGTTATTTAACAAATGGCAAAATATTGCTTACAAGTATGACAAAGATTTAGTACTCATGACTCAC
TTCATAACAAAGAATATTACAGATAATCATGGGAAGAATAAGACTACAGTACATGGTTACTTCTCTTCAATTT
TTCATGGTGGAGTGGATAGTCTAGTCGACTTGATGAACAAGAGCTTTCCTGAGTTGGGTATTAAAAAAACTGA
TTGCAAAGAATTTAGCTGGATTGATACAACCATCTTCTACAGTGGTGTTGTAAATTTTAACACTGCTAATTTT
AAAAAGGAAATTTTGCTTGATAGATCAGCTGGGAAGAAGACGGCTTTCTCAATTAAGTTAGACTATGTTAAGA
AACCAATTCCAGAAACTGCAATGGTCAAAATTTTGGAAAAATTATATGAAGAAGATGTAGGAGCTGGGATGTA
TGTGTTGTACCCTTACGGTGGTATAATGGAGGAGATTTCAGAATCAGCAATTCCATTCCCTCATCGAGCTGGA
ATAATGTATGAACTTTGGTACACTGCTTCCTGGGAGAAGCAAGAAGATAATGAAAAGCATATAAACTGGGTTC
GAAGTGTTTATAATTTTACGACTCCTTATGTGTCCCAAAATCCAAGATTGGCGTATCTCAATTATAGGGACCT
TGATTTAGGAAAAACTAATCATGCGAGTCCTAATAATTACACACAAGCACGTATTTGGGGTGAAAAGTATTTT
GGTAAAAATTTTAACAGGTTAGTTAAGGTGAAAACTAAAGTTGATCCCAATAATTTTTTTAGAAACGAACAAA
GTATCCCACCTCTTCCACCGCATCATCAT
>A1_LC120319.1
ATGAATTGCTCAGCATTTTCCTTTTGGTTTGTTTGCAAAATAATATTTTTCTTTCTCTCATTCCATATCCAAA
TTTCAATAGCTAATCCTCGAGAAAACTTCCTTAAATGCTTCTCAAAACATATTCCCAACAATGTAGCAAATCC
AAAACTCGTATACACTCAACACGACCAATTGTATATGTCTATCCTGAATTCGACAATACAAAATCTTAGATTC
ATCTCTGATACAACCCCAAAACCACTCGTTATTGTCACTCCTTCAAATAACTCCCATATCCAAGCAACTATTT
TATGCTCTAAGAAAGTTGGCTTGCAGATTCGAACTCGAAGCGGTGGCCATGATGCTGAGGGTATGTCCTACAT
ATCTCAAGTCCCATTTGTTGTAGTAGACTTGAGAAACATGCATTCGATCAAAATAGATGTTCATAGCCAAACT
GCGTGGGTTGAAGCCGGAGCTACCCTTGGAGAAGTTTATTATTGGATCAATGAGAAGAATGAGAATCTTAGTT
TTCCTGGTGGGTATTGCCCTACTGTTGGCGTAGGTGGACACTTTAGTGGAGGAGGCTATGGAGCATTGATGCG
AAATTATGGCCTTGCGGCTGATAATATTATTGATGCACACTTAGTCAATGTTGATGGAAAAGTTCTAGATCGA
AAATCCATGGGAGAAGATCTGTTTTGGGCTATACGTGGTGGTGGAGGAGAAAACTTTGGAATCATTGCAGCAT
GGAAAATCAAACTGGTTGATGTCCCATCAAAGTCTACTATATTCAGTGTTAAAAAGAACATGGAGATACATGG
GCTTGTCAAGTTATTTAACAAATGGCAAAATATTGCTTACAAGTATGACAAAGATTTAGTACTCATGACTCAC
TTCATAACAAAGAATATTACAGATAATCATGGGAAGAATAAGACTACAGTACATGGTTACTTCTCTTCAATTT
TTCATGGTGGAGTGGATAGTCTAGTCGACTTGATGAACAAGAGCTTTCCTGAGTTGGGTATTAAAAAAGCTGA
TTGCAAAGAATTTAGCTGGATTGATACAACCATCTTCTACAGTGGTGTTGTAAATTTTAACACTGCTAATTTT
AAAAAGGAAATTTTGCTTGATAGATCAGCTGGGAAGAAGACGGCTTTCTCAATTAAGTTAGACTATGTTAAGA
AACCAATTCCAGAAACTGCAATGGTCAAAATTTTGGAAAAATTATATGAAGAAGATGTAGGAGCTGGGATGTA
TGTGTTGTACCCTTACGGTGGTATAATGGAGGAGATTTCAGAATCAGCAATTCCATTCCCTCATCGAGCTGGA
ATAATGTATGAACTTTGGTACACTGCTTCCTGGGAGAAGCAAGAAGATAATGAAAAGCATATAAACTGGGTTC
GAAGTGTTTATAATTTTACGACTCCTTATGTGTCCCAAAATCCAAGATTGGCGTATCTCAATTATAGGGACCT
TGATTTAGGAAAAACTAATCATGCGAGTCCTAATAATTACACACAAGCACGTATTTGGGGTGAAAAGTATTTT
GGTAAAAATTTTAACAGGTTAGTTAAGGTGAAAACTAAAGTTGATCCCAATAATTTTTTTAGAAACGAACAAA
GTATCCCACCTCTTCCACCGCATCATCAT
>A1_KT876015.1
ATGAATTGCTCAGCATTTTCCTTTTGGTTTGTTTGCAAAATAATATTTTTCTTTCTCTCATTCCATATCCAAA
TTTCAATAGCTAATCCTCGAGAAAACTTCCTTAAATGCTTCTCAAAACATATTCCCAACAATGTAGCAAATCC
AAAACTCGTATACACTCAACACGACCAATTGTATATGTCTATCCTGAATTCGACAATACAAAATCTTAGATTC
ATCTCTGATACAACCCCAAAACCACTCGTTATTGTCACTCCTTCAAATAACTCCCATATCCAAGCAACTATTT
TATGCTCTAAGAAAGTTGGCTTGCAGATTCGAACTCGAAGCGGTGGCCATGATGCTGAGGGTATGTCCTACAT
ATCTCAAGTCCCATTTGTTGTAGTAGACTTGAGAAACATGCATTCGATCAAAATAGATGTTCATAGCCAAACT
GCGTGGGTTGAAGCCGGAGCTACCCTTGGAGAAGTTTATTATTGGATCAATGAGAAGAATGAGAATCTTAGTT
TTCCTGGTGGGTATTGCCCTACTGTTGGCGTAGGTGGACACTTTAGTGGAGGAGGCTATGGAGCATTGATGCG
AAATTATGGCCTTGCGGCTGATAATATTATTGATGCACACTTAGTCAATGTTGATGGAAAAGTTCTAGATCGA
AAATCCATGGGAGAAGATCTGTTTTGGGCTATACGTGGTGGTGGAGGAGAAAACTTTGGAATCATTGCAGCAT
GGAAAATCAAACTGGTTGCTGTCCCATCAAAGTCTACTATATTCAGTGTTAAAAAGAACATGGAGATACATGG
GCTTGTCAAGTTATTTAACAAATGGCAAAATATTGCTTACAAGTATGACAAAGATTTAGTACTCATGACTCAC
TTCATAACAAAGAATATTACAGATAATCATGGGAAGAATAAGACTACAGTACATGGTTACTTCTCTTCAATTT

TTCATGGTGGAGTGGATAGTCTAGTCGACTTGATGAACAAGAGCTTTCGTGAGTTGGGTATTAAAAAAACTGA
TTGCAAAGAATTTAGCTGGATTGATACAACCATCTTCTACAGTGGTGTTGTAAATTTTAACACTGCTAATTTT
AAAAAGGAAATTTTGCTTGATAGATCAGCTGGGAAGAAGACGGCTTTCTCAATTAAGTTAGACTATGTTAAGA
AACCAATTCCAGAAACTGCAATGGTCAAAATTTTGGAAAAATTATATGAAGAAGATGTAGGAGCTGGGATGTA
TGTGTTGTACCCTTACGGTGGTATAATGGAGGAGATTTCAGAATCAGCAATTCCATTCCCTCATCGAGCTGGA
ATAATGTATGAACTTTGGTACACTGCTTCCTGGGAGAAGCAAGAAGATAATGAAAAGCATATAAACTGGGTTC
GAAGTGTTTATAATTTTACGACTCCTTATGTGTCCCAAAATCCAAGATTGGCGTATCTCAATTATAGGGACCT
TGATTTAGGAAAAACTAATCATGCGAGTCCTAATAATTACACACAAGCACGTATTTGGGGTGAAAAGTATTTT
GGTAAAAATTTTAACAGGTTAGTTAAGGTGAAAACTAAAGTTGATCCCAATAATTTTTTTAGAAACGAACAAA
GTATCCCACCTCTTCCACCGCATCATCAT
>A1_KT876040.1
ATGAATTGCTCAGCATTTTCCTTTTGGTTTGTTTGCAAAATAATATTTTTCTTTCTCTCATTCCATATCCAAA
TTTCAATAGCTAATCCTCGAGAAAACTTCCTTAAATGCTTCTCAAAACATATTCCCAACAATGTAGCAAATCC
AAAACTCGTATACACTCAACACGACCAATTGTATATGTCTATCCTGAATTCGACAATACAAAATCTTAGATTC
ATCTCTGATACAACCCCAAAACCACTCGTTATTGTCACTCCTTCAAATAACTCCCATATCCAAGCAACTATTT
TATGCTCTAAGAAAGTTGGCTTGCAGATTCGAACTCGAAGCGGTGGCCATGATGCTGAGGGTATGTCCTACAT
ATCTCAAGTCCCATTTGTTGTAGTAGACTTGAGAAACATGCATTCGATCAAAATAGATGTTCATAGCCAAACT
GCGTGGGTTGAAGCCGGAGCTACCCTTGGAGAAGTTTATTATTGGATCAATGAGAAGAATGAGAATCTTAGTT
TTCCTGGTGGGTATTGCCCTACTGTTGGCGTAGGTGGACACTTTAGTGGAGGAGGCTATGGAGCATTGATGCG
AAATTATGGCCTTGCGGCTGATAATATTATTGATGCACACTTAGTCAATGTTGATGGAAAAGTTCTAGATCGA
AAATCCATGGGAGAAGATCTGTTTTGGGCTATACGTGGTGGTGGAGGAGAAAACTTTGGAATCATTGCAGCAT
GGAAAATCAAACTGGTTGCTGTCCCATCAAAGTCTACTATATTCAGTGTTAAAAAGAACATGGAGATACATGG
GCTTGTCAAGTTATTTAACAAATGGCAAAATATTGCTTACAAGTATGACAAAGATTTAGTACTCATGACTCAC
TTCATAACAAAGAATATTACAGATAATCATGGGAAGAATAAGACTACAGTACATGGTTACTTCTCTTCAATTT
TTCATGGTGGAGTGGATAGTCTAGTCGACTTGATGAACAAGAGCTTTCGTGAGTTGGGTATTAAAAAAACTGA
TTGCAAAGAATTTAGCTGGATTGATACAACCATCTTCTACAGTGGTGTTGTAAATTTTAACACTGCTAATTTT
AAAAAGGAAATTTTGCTTGATAGATCAGCTGGGAAGAAGACGGCTTTCTCAATTAAGTTAGACTATGTTAAGA
AACCAATTCCAGAAACTGCAATGGTCAAAATTTTGGAAAAATTATATGAAGAAGATGTAGGAGCTGGGATGTA
TGTGTTGTACCCTTACGGTGGTATAATGGAGGAGATTTCAGAATCAGCAATTCCATTCCCTCATCGAGCTGGA
ATAATGTATGAACTTTGGTACACTGCTTCCTGGGAGAAGCAAGAAGATAATGAAAAGCATATAAACTGGGTTC
GAAGTGTTTATAATTTTACGACTCCTTATGTGTCCCAAAATCCAAGATTGGCGTATCTCAATTATAGGGACCT
TGATTTAGGAAAAACTAATCATGCGAGTCCTAATAATTACACACAAGCACGTATTTGGGGTGAAAAGTATTTT
GGTAAAAATTTTAACAGGTTAGTTAAGGTGAAAACTAAAGTTGATCCCAATAATTTTTTTAGAAACGAACAAA
GTATCCCACCTCTTCCACCGCATCATCAT
>A1_MG996415.1
TTTTGGTTTGTTTGCAAAATAATATTTTTCTTTCTCTCATTCCATATCCAAATTTCAATAGCTAATCCTCGAG
AAAACTTCCTTAAATGCTTCTCAAAACATATTCCCAACAATGTAGCAAATCCAAAACTCGTATACACTCAACA
CGACCAATTGTATATGTCTATCCTGAATTCGACAATACAAAATCTTAGATTCATCTCTGATACAACCCCAAAA
CCACTCGTTATTGTCACTCCTTCAAATAACTCCCATATCCAAGCAACTATTTTATGCTCTAAGAAAGTTGGCT
TGCAGATTCGAACTCGAAGCGGTGGCCATGATGCTGAGGGTATGTCCTACATATCTCAAGTCCCATTTGTTGT
AGTAGACTTGAGAAACATGCATTCGATCAAAATAGATGTTCATAGCCAAACTGCGTGGGTTGAAGCCGGAGCT
ACCCTTGGAGAAGTTTATTATTGGATCAATGAGAAGAATGAGAATCTTAGTTTTCCTGGTGGGTATTGCCCTA
CTGTTGGCGTAGGTGGACACTTTAGTGGAGGAGGCTATGGAGCATTGATGCGAAATTATGGCCTTGCGGCTGA
TAATATTATTGATGCACACTTAGTCAATGTTGATGGAAAAGTTCTAGATCGAAAATCCATGGGAGAAGATCTG
TTTTGGGCTATACGTGGTGGTGGAGGAGAAAACTTTGGAATCATTGCAGCATGGAAAATCAAACTGGTTGCTG
TCCCATCAAAGTCTACTATATTCAGTGTTAAAAAGAACATGGAGATACATGGGCTTGTCAAGTTATTTAACAA
ATGGCAAAATATTGCTTACAAGTATGACAAAGATTTAGTACTCATGACTCACTTCATAACAAAGAATATTACA
GATAATCATGGGAAGAATAAGACTACAGTACATGGTTACTTCTCTTCAATTTTTCATGGTGGAGTGGATAGTC
TAGTCGACTTGATGAACAAGAGCTTTCGTGAGTTGGGTATTAAAAAAACTGATTGCAAAGAATTTAGCTGGAT
TGATACAACCATCTTCTACAGTGGTGTTGTAAATTTTAACACTGCTAATTTTAAAAAGGAAATTTTGCTTGAT
AGATCAGCTGGGAAGAAGACGGCTTTCTCAATTAAGTTAGACTATGTTAAGAAACCAATTCCAGAAACTGCAA
TGGTCAAAATTTTGGAAAAATTATATGAAGAAGATGTAGGAGCTGGGATGTATGTGTTGTACCCTTACGGTGG
TATAATGGAGGAGATTTCAGAATCAGCAATTCCATTCCCTCATCGAGCTGGAATAATGTATGAACTTTGGTAC
ACTGCTTCCTGGGAGAAGCAAGAAGATAATGAAAAGCATATAAACTGGGTTCGAAGTGTTTATAATTTTACGA
CTCCTTATGTGTCCCAAAATCCAAGATTGGCGTATCTCAATTATAGGGACCTTGATTTAGGAAAAACTAATCA
TGCGAGTCCTAATAATTACACACAAGCACGTATTTGGGGTGAAAAGTATTTTGGTAAAAATTTTAACAGGTTA
GTTAAGGTGAAAACTAAAGTTGATCCCAATAATTTTTTTAGAAACGAACAAAGTATCCCACCTCTT
>A1_MG996417.1

TTTTGGTTTGTTTGCAAAATAATATTTTTCTTTCTCTCATTCCATATCCAAATTTCAATAGCTAATCCTCGAG
AAAACTTCCTTAAATGCTTCTCAAAACATATTCCCAACAATGTAGCAAATCCAAAACTCGTATACACTCAACA
CGACCAATTGTATATGTCTATCCTGAATTCGACAATACAAAATCTTAGATTCATCTCTGATACAACCCCAAAA
CCACTCGTTATTGTCACTCCTTCAAATAACTCCCATATCCAAGCAACTATTTTATGCTCTAAGAAAGTTGGCT
TGCAGATTCGAACTCGAAGCGGTGGCCATGATGCTGAGGGTATGTCCTACATATCTCAAGTCCCATTTGTTGT
AGTAGACTTGAGAAACATGCATTCGATCAAAATAGATGTTCATAGCCAAACTGCGTGGGTTGAAGCCGGAGCT
ACCCTTGGAGAAGTTTATTATTGGATCAATGAGAAGAATGAGAATCTTAGTTTTCCTGGTGGGTATTGCCCTA
CTGTTGGCGTAGGTGGACACTTTAGTGGAGGAGGCTATGGAGCATTGATGCGAAATTATGGCCTTGCGGCTGA
TAATATTATTGATGCACACTTAGTCAATGTTGATGGAAAAGTTCTAGATCGAAAATCCATGGGAGAAGATCTG
TTTTGGGCTATACGTGGTGGTGGAGGAGAAAACTTTGGAATCATTGCAGCATGGAAAATCAAACTGGTTGCTG
TCCCATCAAAGTCTACTATATTCAGTGTTAAAAAGAACATGGAGATACATGGGCTTGTCAAGTTATTTAACAA
ATGGCAAAATATTGCTTACAAGTATGACAAAGATTTAGTACTCATGACTCACTTCATAACAAAGAATATTACA
GATAATCATGGGAAGAATAAGACTACAGTACATGGTTACTTCTCTTCAATTTTTCATGGTGGAGTGGATAGTC
TAGTCGACTTGATGAACAAGAGCTTTCGTGAGTTGGGTATTAAAAAAACTGATTGCAAAGAATTTAGCTGGAT
TGATACAACCATCTTCTACAGTGGTGTTGTAAATTTTAACACTGCTAATTTTAAAAAGGAAATTTTGCTTGAT
AGATCAGCTGGGAAGAAGACGGCTTTCTCAATTAAGTTAGACTATGTTAAGAAACCAATTCCAGAAACTGCAA
TGGTCAAAATTTTGGAAAAATTATATGAAGAAGATGTAGGAGCTGGGATGTATGTGTTGTACCCTTACGGTGG
TATAATGGAGGAGATTTCAGAATCAGCAATTCCATTCCCTCATCGAGCTGGAATAATGTATGAACTTTGGTAC
ACTGCTTCCTGGGAGAAGCAAGAAGATAATGAAAAGCATATAAACTGGGTTCGAAGTGTTTATAATTTTACGA
CTCCTTATGTGTCCCAAAATCCAAGATTGGCGTATCTCAATTATAGGGACCTTGATTTAGGAAAAACTAATCA
TGCGAGTCCTAATAATTACACACAAGCACGTATTTGGGGTGAAAAGTATTTTGGTAAAAATTTTAACAGGTTA
GTTAAGGTGAAAACTAAAGTTGATCCCAATAATTTTTTTAGAAACGAACAAA
>A1_MT338560.1
ATGAATTGCTCAGCATTTTCCTTTTGGTTTGTTTGCAAAATAATATTTTTCTTTCTCTCATTCCATATCCAAA
TTTCAATAGCTAATCCTCGAGAAAACTTCCTTAAATGCTTCTCAAAACATATTCCCAACAATGTAGCAAATCC
AAAACTCGTATACACTCAACACGACCAATTGTATATGTCTATCCTGAATTCGACAATACAAAATCTTAGATTC
ATCTCTGATACAACCCCAAAACCACTCGTTATTGTCACTCCTTCAAATAACTCCCATATCCAAGCAACTATTT
TATGCTCTAAGAAAGTTGGCTTGCAGATTCGAACTCGAAGCGGTGGCCATGATGCTGAGGGTATGTCCTACAT
ATCTCAAGTCCCATTTGTTGTAGTAGACTTGAGAAACATGCATTCGATCAAAATAGATGTTCATAGCCAAACT
GCGTGGGTTGAAGCCGGAGCTACCCTTGGAGAAGTTTATTATTGGATCAATGAGAAGAATGAGAATCTTAGTT
TTCCTGGTGGGTATTGCCCTACTGTTGGCGTAGGTGGACACTTTAGTGGAGGAGGCTATGGAGCATTGATGCG
AAATTATGGCCTTGCGGCTGATAATATTATTGATGCACACTTAGTCAATGTTGATGGAAAAGTTCTAGATCGA
AAATCCATGGGAGAAGATCTGTTTTGGGCTATACGTGGTGGTGGAGGAGAAAACTTTGGAATCATTGCAGCAT
GGAAAATCAAACTGGTTGCTGTCCCATCAAAGTCTACTATATTCAGTGTTAAAAAGAACATGGAGATACATGG
GCTTGTCAAGTTATTTAACAAATGGCAAAATATTGCTTACAAGTATGACAAAGATTTAGTACTCATGACTCAC
TTCATAACAAAGAATATTACAGATAATCATGGGAAGAATAAGACTACAGTACATGGTTACTTCTCTTCAATTT
TTCATGGTGGAGTGGATAGTCTAGTCGACTTGATGAACAAGAGCTTTCGTGAGTTGGGTATTAAAAAAACTGA
TTGCAAAGAATTTAGCTGGATTGATACAACCATCTTCTACAATGGTGTTGTAAATTTTAACACTGCTAATTTT
AAAAAGGAAATTTTGCTTGATAGATCAGCTGGGAAGAAGACGGCTTTCTCAATTAAGTTAGACTATGTTAAGA
AACCAATTCCAGAAACTGCAATGGTCAAAATTTTGGAAAAATTATATGAAGAAGATGTAGGAGCTGGGATGTA
TGTGTTGTACCCTTACGGTGGTATAATGGAGGAGATTTCAGAATCAGCAATTCCATTCCCTCATCGAGCTGGA
ATAATGTATGAACTTTGGTACACTGCTTCCTGGGAGAAGCAAGAAGATAATGAAAAGCATATAAACTGGGTTC
GAAGTGTTTATAATTTTACGACTCCTTATGTGTCCCAAAATCCAAGATTGGCGTATCTCAATTATAGGGACCT
TGATTTAGGAAAAACTAATCATGCGAGTCCTAATAATTACACACAAGCACGTATTTGGGGTGAAAAGTATTTT
GGTAAAAATTTTAACAGGTTAGTTAAGGTGAAAACTAAAGTTGATCCCAATAATTTTTTTAGAAACGAACAAA
GTATCCCACCTCTTCCACCGCATCATCATTAA
>A1_KP970852.1
ATGAATTGCTCAGCATTTTCCTTTTGGTTTGTTTGCAAAATAATATTTTTCTTTCTCTCATTCCATATCC
AAATTTCAATAGCTAATCCTCGAGAAAACTTCCTTAAATGCTTCTCAAAACATATTCCCAACAATGTAGC
AAATCCAAAACTCGTATACACTCAACACGACCAATTGTATATGTCTATCCTGAATTCGACAATACAAAAT
CTTAGATTCATCTCTGATACAACCCCAAAACCACTCGTTATTGTCACTCCTTCAAATAACTCCCATATCC
AAGCAACTATTTTATGCTCTAAGAAAGTTGGCTTGCAGATTCGAACTCGAAGCGGTGGCCATGATGCTGA
GGGTATGTCCTACATTTCTCAACTCCCATTTGTTGTAGTAGACTTGAGAAACATGCATTCGATCAAAATA
GATGTTCATAGCCAAACTGCGTGGGTTGAAGCCGGAGCTACCCTTGGAGAAGTTTATTATTGGATCAATG
AGAAGAATGAGAATCTTAGTTTTCCTGGTGGGTATTGCCCTACTGTTGGCGTAGGTGGACACTTTAGTGG
AGGAGGCTATGGAGCATTGATGCGAAATTATGGCCTTGCGGCTGATAATATTATTGATGCACACTTAGTC
AATGTTGATGGAAAAGTTCTAGATCGAAAATCCATGGGAGAAGATCTGTTTTGGGCTATACGTGGTGGTG
GAGGAGAAAACTTTGGAATCATTGCAGCATGGAAAATCAAACTGGTTGCTGTCCCATCAAAGTCTACTAT

ATTCAGTGTTAAAAAGAACATGGAGATACATGGGCTTGTCAAGTTATTTAACAAATGGCAAAATATTGCT
TACAAGTATGACAAAGATTTAGTACTCATGACTCACTTCATAACAAAGAATATTACAGATAATCATGGGA
AGAATAAGACTACAGTACATGGTTACTTCTCTTCAATTTTTCATGGTGGAGTGGATAGTCTAGTCGACTT
GATGAACAAGAGCTTTCCTGAGTTGGGTATTAAAAAAACTGATTGCAAAGAATTTAGCTGGATTGATACA
ACCATCTTCTACAGTGGTGTTGTAAATTTTAACACTGCTAATTTTAAAAAGGAAATTTTGCTTGATAGAT
CAGCTGGGAAGAAGACGGCTTTCTCAATTAAGTTAGACTATGTTAAGAAACCAATTCCAGAAACTGCAAT
GGTCAAAATTTTGGAAAAATTATATGAAGAAGATGTAGAAGCTGGGATGTATGTGTTGTACCCTTACGGT
GGTATAATGGAGGAGATTTCAGAATCAGCAATTCCATTCCCTCATCGAGCTGGAATAATGTATGAACTTT
GGTACACTGCTTCCTGGGAGAAGCAAGAAGATAATGAAAAGCATATAAACTGGGTTCGAAGTGTATATAA
TTTTACGACTCCTTATGTGTCCCAAAATCCAAGATTGGCGTATCTCAATTATAGGGACCTTGATTTAGGA
AAAACTAATCATGCGAGTCCTAATAATTACACACAAGCACGTATTTGGGGTGAAAAGTATTTTGGTAAAA
ATTTTAACAGGTTAGTTAAAGTGAAAACTAAAGTTGATCCCAATAATTTTTTTAGAAACGAACAAAGTAT
CCCACCTCTTCCACCGCATCATCAT
>A1_KT876013.1
ATGAATTGCTCAGCATTTTCCTTTTGGTTTGTTTGCAAAATAATATTTTTCTTTCTCTCATTCCATATCCAAA
TTTCAATAGCTAATCCTCGAGAAAACTTCCTTAAATGCTTCTCAAAACATATTCCCAACAATGTAGCAAATCC
AAAACTCGTATACACTCAACACGACCAATTGTATATGTCTATCCTGAATTCGACAATACAAAATCTTAGATTC
ATCTCTGATACAACCCCAAAACCACTCGTTATTGTCACTCCTTCAAATAACTCCCATATCCAAGCAACTATTT
TATGCTCTAAGAAAGTTGGCTTGCAGATTCGAACTCGAAGCGGTGGCCATGATGCTGAGGGTATGTCCTACAT
TTCTCAACTCCCATTTGTTGTAGTAGACTTGAGAAACATGCATTCGATCAAAATAGATGTTCATAGCCAAACT
GCGTGGGTTGAAGCCGGAGCTACCCTTGGAGAAGTTTATTATTGGATCAATGAGAAGAATGAGAATCTTAGTT
TTCCTGGTGGGTATTGCCCTACTGTTGGCGTAGGTGGACACTTTAGTGGAGGAGGCTATGGAGCATTGATGCG
AAATTATGGCCTTGCGGCTGATAATATTATTGATGCACACTTAGTCAATGTTGATGGAAAAGTTCTAGATCGA
AAATCCATGGGAGAAGATCTGTTTTGGGCTATACGTGGTGGTGGAGGAGAAAACTTTGGAATCATTGCAGCAT
GGAAAATCAAACTGGTTGCTGTCCCATCAAAGTCTACTATATTCAGTGTTAAAAAGAACATGGAGATACATGG
GCTTGTCAAGTTATTTAACAAATGGCAAAATATTGCTTACAAGTATGACAAAGATTTAGTACTCATGACTCAC
TTCATAACAAAGAATATTACAGATAATCATGGGAAGAATAAGACTACAGTACATGGTTACTTCTCTTCAATTT
TTCATGGTGGAGTGGATAGTCTAGTCGACTTGATGAACAAGAGCTTTCCTGAGTTGGGTATTAAAAAAACTGA
TTGCAAAGAATTTAGCTGGATTGATACAACCATCTTCTACAGTGGTGTTGTAAATTTTAACACTGCTAATTTT
AAAAAGGAAATTTTGCTTGATAGATCAGCTGGGAAGAAGACGGCTTTCTCAATTAAGTTAGACTATGTTAAGA
AACCAATTCCAGAAACTGCAATGGTCAAAATTTTGGAAAAATTATATGAAGAAGATGTAGAAGCTGGGATGTA
TGTGTTGTACCCTTACGGTGGTATAATGGAGGAGATTTCAGAATCAGCAATTCCATTCCCTCATCGAGCTGGA
ATAATGTATGAACTTTGGTACACTGCTTCCTGGGAGAAGCAAGAAGATAATGAAAAGCATATAAACTGGGTTC
GAAGTGTATATAATTTTACGACTCCTTATGTGTCCCAAAATCCAAGATTGGCGTATCTCAATTATAGGGACCT
TGATTTAGGAAAAACTAATCATGCGAGTCCTAATAATTACACACAAGCACGTATTTGGGGTGAAAAGTATTTT
GGTAAAAATTTTAACAGGTTAGTTAAAGTGAAAACTAAAGTTGATCCCAATAATTTTTTTAGAAACGAACAAA
GTATCCCACCTCTTCCACCGCATCATCAT
>A1_KT876034.1
ATGAATTGCTCAGCATTTTCCTTTTGGTTTGTTTGCAAAATAATATTTTTCTTTCTCTCATTCCATATCCAAA
TTTCAATAGCTAATCCTCGAGAAAACTTCCTTAAATGCTTCTCAAAACATATTCCCAACAATGTAGCAAATCC
AAAACTCGTATACACTCAACACGACCAATTGTATATGTCTATCCTGAATTCGACAATACAAAATCTTAGATTC
ATCTCTGATACAACCCCAAAACCACTCGTTATTGTCACTCCTTCAAATAACTCCCATATCCAAGCAACTATTT
TATGCTCTAAGAAAGTTGGCTTGCAGATTCGAACTCGAAGCGGTGGCCATGATGCTGAGGGTATGTCCTACAT
TTCTCAACTCCCATTTGTTGTAGTAGACTTGAGAAACATGCATTCGATCAAAATAGATGTTCATAGCCAAACT
GCGTGGGTTGAAGCCGGAGCTACCCTTGGAGAAGTTTATTATTGGATCAATGAGAAGAATGAGAATCTTAGTT
TTCCTGGTGGGTATTGCCCTACTGTTGGCGTAGGTGGACACTTTAGTGGAGGAGGCTATGGAGCATTGATGCG
AAATTATGGCCTTGCGGCTGATAATATTATTGATGCACACTTAGTCAATGTTGATGGAAAAGTTCTAGATCGA
AAATCCATGGGAGAAGATCTGTTTTGGGCTATACGTGGTGGTGGAGGAGAAAACTTTGGAATCATTGCAGCAT
GGAAAATCAAACTGGTTGCTGTCCCATCAAAGTCTACTATATTCAGTGTTAAAAAGAACATGGAGATACATGG
GCTTGTCAAGTTATTTAACAAATGGCAAAATATTGCTTACAAGTATGACAAAGATTTAGTACTCATGACTCAC
TTCATAACAAAGAATATTACAGATAATCATGGGAAGAATAAGACTACAGTACATGGTTACTTCTCTTCAATTT
TTCATGGTGGAGTGGATAGTCTAGTCGACTTGATGAACAAGAGCTTTCCTGAGTTGGGTATTAAAAAAACTGA
TTGCAAAGAATTTAGCTGGATTGATACAACCATCTTCTACAGTGGTGTTGTAAATTTTAACACTGCTAATTTT
AAAAAGGAAATTTTGCTTGATAGATCAGCTGGGAAGAAGACGGCTTTCTCAATTAAGTTAGACTATGTTAAGA
AACCAATTCCAGAAACTGCAATGGTCAAAATTTTGGAAAAATTATATGAAGAAGATGTAGAAGCTGGGATGTA
TGTGTTGTACCCTTACGGTGGTATAATGGAGGAGATTTCAGAATCAGCAATTCCATTCCCTCATCGAGCTGGA
ATAATGTATGAACTTTGGTACACTGCTTCCTGGGAGAAGCAAGAAGATAATGAAAAGCATATAAACTGGGTTC
GAAGTGTATATAATTTTACGACTCCTTATGTGTCCCAAAATCCAAGATTGGCGTATCTCAATTATAGGGACCT

TGATTTAGGAAAAACTAATCATGCGAGTCCTAATAATTACACACAAGCACGTATTTGGGGTGAAAAGTATTTT
GGTAAAAATTTTAACAGGTTAGTTAAAGTGAAAACTAAAGTTGATCCCAATAATTTTTTTAGAAACGAACAAA
GTATCCCACCTCTTCCACCGCATCATCAT
>A1_KP970853.1
ATGAATTGCTCAGCATTTTCCTTTTGGTTTGTTTGCAAAATAATATTTTTCTTTCTCTCATTCCATATCC
AAATTTCAATAGCTAATCCTCGAGAAAACTTCCTTAAATGCTTCTCAAAACATATTCCCAACAATGTAGC
AAATCCAAAACTCGTATACACTCAACACGACCAATTGTATATGTCTATCCTGAATTCGACAATACAAAAT
CTTAGATTCATCTCTGATACAACCCCAAAACCACTCGTTATTGTCACTCCTTCAAATAACTCCCATATCC
AAGCAACTATTTTATGCTCTAAGAAAGTTGGCTTGCAGATTCGAACTCGAAGCGGTGGCCATGATGCTGA
GGGTATGTCCTACATTTCTCAACTCCCATTTGTTGTAGTAGACTTGAGAAACATGCATTCGATCAAAATA
GATGTTCATAGCCAAACTGCGTGGGTTGAAGCCGGAGCTACCCTTGGAGAAGTTTATTATTGGATCAATG
AGAAGAATGAGAATCTTAGTTTTCCTGGTGGGTATTGCCCTACTGTTGGCGTAGGTGGACACTTTAGTGG
AGGAGGCTATGGAGCATTGATGCGAAATTATGGCCTTGCGGCTGATAATATTATTGATGCACACTTAGTC
AATGTTGATGGAAAAGTTCTAGATCGAAAATCCATGGGAGAAGATCTGTTTTGGGCTATACGTGGTGGTG
GAGGAGAAAACTTTGGAATCATTGCAGCATGGAAAATCAAACTGGTTGCTGTCCCATCAAAGTCTACTAT
ATTCAGTGTTAAAAAGAACATGGAGATACATGGGCTTGTCAAGTTATTTAACAAATGGCAAAATATTGCT
TACAAGTATGACAAAGATTTAGTACTCATGACTCACTTCATAACAAAGAATATTACAGATAATCATGGGA
AGAATAAGACTACAGTACATGGTTACTTCTCTTCAATTTTTCATGGTGGAGTGGATAGTCTAGTCGACTT
GATGAACAAGAGCTTTCCTGAGTTGGGTATTAAAAAAACTGATTGCAAAGAATTTAGCTGGATTGATACA
ACCATCTTCTACAGTGGTGTTGTAAATTTTAACACTGCTAATTTTAAAAAGGAAATTTTGCTTGATAGAT
CAGCTGGGAAGAAGACGGCTTTCTCAATTAAGTTAGACTATGTTAAGAAACCAATTCCAGAAACTGCAAT
GGTCAAAATTTTGGAAAAATTATATGAAGAAGATGTAGGAGCTGGGATGTATGTGTTGTACCCTTACGGT
GGTATAATGGAGGAGATTTCAGAATCAGCAATTCCATTCCCTCATCGAGCTGGAATAATGTATGAACTTT
GGTACACTGCTTCCTGGGAGAAGCAAGAAGATAATGAAAAGCATATAAACTGGGTTCGAAGTGTTTATAA
TTTTACGACTCCTTATGTGTCCCAAAATCCAAGATTGGCGTATCTCAATTATAGGGACCTTGATTTAGGA
AAAACTAATCATGCGAGTCCTAATAATTACACACAAGCACGTATTTGGGGTGAAAAGTATTTTGGTAAAA
ATTTTAACAGGTTAGTTAAGGTGAAAACTAAAGTTGATCCCAATAATTTTTTTAGAAACGAACAAAGTAT
CCCACCTCTTCCACCGCATCATCAT
>A1_KP970854.1
ATGAATTGCTCAGCATTTTCCTTTTGGTTTGTTTGCAAAATAATATTTTTCTTTCTCTCATTCCATATCC
AAATTTCAATAGCTAATCCTCGAGAAAACTTCCTTAAATGCTTCTCAAAACATATTCCCAACAATGTAGC
AAATCCAAAACTCGTATACACTCAACACGACCAATTGTATATGTCTATCCTGAATTCGACAATACAAAAT
CTTAGATTCATCTCTGATACAACCCCAAAACCACTCGTTATTGTCACTCCTTCAAATAACTCCCATATCC
AAGCAACTATTTTATGCTCTAAGAAAGTTGGCTTGCAGATTCGAACTCGAAGCGGTGGCCATGATGCTGA
GGGTATGTCCTACATTTCTCAACTCCCATTTGTTGTAGTAGACTTGAGAAACATGCATTCGATCAAAATA
GATGTTCATAGCCAAACTGCGTGGGTTGAAGCCGGAGCTACCCTTGGAGAAGTTTATTATTGGATCAATG
AGAAGAATGAGAATCTTAGTTTTCCTGGTGGGTATTGCCCTACTGTTGGCGTAGGTGGACACTTTAGTGG
AGGAGGCTATGGAGCATTGATGCGAAATTATGGCCTTGCGGCTGATAATATTATTGATGCACACTTAGTC
AATGTTGATGGAAAAGTTCTAGATCGAAAATCCATGGGAGAAGATCTGTTTTGGGCTATACGTGGTGGTG
GAGGAGAAAACTTTGGAATCATTGCAGCATGGAAAATCAAACTGGTTGCTGTCCCATCAAAGTCTACTAT
ATTCAGTGTTAAAAAGAACATGGAGATACATGGGCTTGTCAAGTTATTTAACAAATGGCAAAATATTGCT
TACAAGTATGACAAAGATTTAGTACTCATGACTCACTTCATAACAAAGAATATTACAGATAATCATGGGA
AGAATAAGACTACAGTACATGGTTACTTCTCTTCAATTTTTCATGGTGGAGTGGATAGTCTAGTCGACTT
GATGAACAAGAGCTTTCCTGAGTTGGGTATTAAAAAAACTGATTGCAAAGAATTTAGCTGGATTGATACA
ACCATCTTCTACAGTGGTGTTGTAAATTTTAACACTGCTAATTTTAAAAAGGAAATTTTGCTTGATAGAT
CAGCTGGGAAGAAGACGGCTTTCTCAATTAAGTTAGACTATGTTAAGAAACCAATTCCAGAAACTGCAAT
GGTCAAAATTTTGGAAAAATTATATGAAGAAGATGTAGAAGCTGGGATGTATGTGTTGTACCCTTACGGT
GGTATAATGGAGGAGATTTCAGAATCAGCAATTCCATTCCCTCATCGAGCTGGAATAATGTATGAACTTT
GGTACACTGCTTCCTGGGAGAAGCAAGAAGATAATGAAAAGCATATAAACTGGGTTCGAAGTGTTTATAA
TTTTACGACTCCTTATGTGTCCCAAAATCCAAGATTGGCGTATCTCAATTATAGGGACCTTGATTTAGGA
AAAACTAATCATGCGAGTCCTAATAATTACACACAAGCACGTATTTGGGGTGAAAAGTATTTTGGTAAAA
ATTTTAACAGGTTAGTTAAGGTGAAAACTAAAGTTGATCCCAATAATTTTTTTAGAAACGAACAAAGTAT
CCCACCTCTTCCACCGCATCATCAT

>A1_KP970855.1
ATGAATTGCTCAGCATTTTCCTTTTGGTTTGTTTGCAAAATAATATTTTTCTTTCTCTCATTCCATATCC
AAATTTCAATAGCTAATCCTCGAGAAAACTTCCTTAAATGCTTCTCAAAACATATTCCCAACAATGTAGC
AAATCCAAAACTCGTATACACTCAACACGACCAATTGTATATGTCTATCCTGAATTCGACAATACAAAAT

CTTAGATTCATCTCTGATACAACCCCAAAACCACTCGTTATTGTCACTCCTTCAAATAACTCCCATATCC
AAGCAACTATTTTATGCTCTAAGAAAGTTGGCTTGCAGATTCGAACTCGAAGCGGTGGCCATGATGCTGA
GGGTATGTCCTACATATCTCAAGTCCCATTTGTTGTAGTAGACTTGAGAAACATGCATTCGATCAAAATA
GATGTTCATAGCCAAACTGCGTGGGTTGAAGCCGGAGCTACCCTTGGAGAAGTTTATTATTGGATCAATG
AGAAGAATGAGAATCTTAGTTTTCCTGGTGGGTATTGCCCTACTGTTGGCGTAGGTGGACACTTTAGTGG
AGGAGGCTATGGAGCATTGATGCGAAATTATGGCCTTGCGGCTGATAATATTATTGATGCACACTTAGTC
AATGTTGATGGAAAAGTTCTAGATCGAAAATCCATGGGAGAAGATCTGTTTTGGGCTATACGTGGTGGTG
GAGGAGAAAACTTTGGAATCATTGCAGCATGGAAAATCAAACTGGTTGCTGTCCCATCAAAGTCTACTAT
ATTCAGTGTTAAAAAGAACATGGGGATACATGGGCTTGTCAAGTTATTTAACAAATGGCAAAATATTGCT
TACAAGTATGACAAAGATTTAGTACTCATGACTCACTTCATAACAAAGAATATTACAGATAATCATGGGA
AGAATAAGACTACAGTACATGGTTACTTCTCTTCAATTTTTCATGGTGGAGTGGATAGTCTAGTCGACTT
GATGAACAAGAGCTTTCCTGAGTTGGGTATTAAAAAAACTGATTGCAAAGAATTTAGCTGGATTGATACA
ACCATCTTCTACAGTGGTGTTGTAAATTTTAACACTGCTAATTTTAAAAAGGAAATTTTGCTTGATAGAT
CAGCTGGGAAGAAGACGGCTTTCTCAATTAAGTTAGACTATGTTAAGAAACCAATTCCAGAAACTGCAAT
GGTCAAAATTTTGGAAAAATTATATGAAGAAGATGTAGAAGCTGGGATGTATGTGTTGTACCCTTACGGT
GGTATAATGGAGGAGATTTCAGAATCAGCAATTCCATTCCCTCATCGAGCTGGAATAATGTATGAACTTT
GGTACACTGCTTCCTGGGAGAAGCAAGAAGATAATGAAAAGCATATAAACTGGGTTCGAAGTGTATATAA
TTTTACGACTCCTTATGTGTCCCAAAATCCAAGATTGGCGTATCTCAATTATAGGGACCTTGATTTAGGA
AAAACTAATCATGCGAGTCCTAATAATTACACACAAGCACGTATTTGGGGTGAAAAGTATTTTGGTAAAA
ATTTTAACAGGTTAGTTAAAGTGAAAACTAAAGTTGATCCCAATAATTTTTTTAGAAACGAACAAAGTAT
CCCACCTCTTCCACCGCATCATCAT
>A1_LC378397.1
AATAACTCCCATATCCAAGCAACTATTTTATGCTCTAAGAAAGTTGGCTTGCAGATTCGAACTCGAAGCGGTG
GCCATGATGCTGAGGGTATGTCCTACATATCTCAAGTCCCATTTGTTGTAGTAGACTTGAGAAACATGCATTC
GATCAAAATAGATGTTCATAGCCAAACTGCGTGGGTTGAAGCCGGAGCTACCCTTGGAGAAGTTTATTATTGG
ATCAATGAGAAGAATGAGAATCTTAGTTTTCCTGGTGGGTATTGCCCTACTGTTGGCGTAGGTGGACACTTTA
GTGGAGGAGGCTATGGAGCATTGATGCGAAATTATGGCCTTGCGGCTGATAATATTATTGATGCACACTTAGT
CAATGTTGATGGAAAAGTTCTAGATCGAAAATCCATGGGAGAAGATCTGTTTTGGGCTATACGTGGTGGTGGA
GGAGAAAACTTTGGAATCATTGCAGCATGGAAAATCAAACTGGTTGCTGTCCCATCAAAGTCTACTATATTCA
GTGTTAAAAAGAACATGGAGATACATGGGCTTGTCAAGTTATTTAACAAATGGCAAAATATTGCTTACAAGTA
TGTCAAAGATTTAGTACTCATGACTCACTTCATACCAAAGAATATTACAGATAATCATGGGAAGAATAAGACT
ACAGTACATGGTTACTTCTCTTCAATTTTTCATGGTGGAGTGGATAGTCTAGTCGACTTGATGAACAAGAGCT
TTCCTGAGTTGGGTATTAAAAAAACTGATTGCAAAGAATTTAGCTGGATTGATACAACCATCTTCTACAGTGG
TGTTGTAAATTTTAACACTGCTAATTTTAAAAAGGAAATTTTGCTTGATAGATCAGCTGGGAAGAAGACGGCT
TTCTCAATTAAGTTAGACTATGTTAAGAAACCAATTCCAGAAACTGCAATGGTCAAAATTTTGGAAAAATTAT
ATGAAGAAGATGTAGGAGCTGGGATGTATGTGTTGTACCCTTACGGTGGTATAATGGAGGAGATTTCAGAATC
AGCAATTCCATTCCCTCATCGAGCTGGAATAATGTATGAACTTTGGTACACTGCTTCCTGGGAGAAGCAAGAA
GATAATGAAAAGCATATAAACTGGGTTCGAAGTGTTTATAATTTTACGACTCCTTATGTGTCCCAAAATCCAA
GATTGGCGTATCTCAATTATAGGGACCTTGATTTAGGAAAAACTAATCATGCGAGTCCT
>A1_LC378398.1
AATAACTCCCATATCCAAGCAACTATTTTATGCTCTAAGAAAGTTGGCTTGCAGATTCGAACTCGAAGCGGTG
GCCATGATGCTGAGGGTATGTCCTACATATCTCAAGTCCCATTTGTTGTAGTAGACTTGAGAAACATGCATTC
GATCAAAATAGATGTTCATAGCCAAACTGCGTGGGTTGAAGCCGGAGCTACCCTTGGAGAAGTTTATTATTGG
ATCAATGAGAAGAATGAGAATCTTAGTTTTCCTGGTGGGTATTGCCCTACTGTTGGCGTAGGTGGACACTTTA
GTGGAGGAGGCTATGGAGCATTGATGCGAAATTATGGCCTTGCGGCTGATAATATTATTGATGCACACTTAGT
CAATGTTGATGGAAAAGTTCTAGATCGAAAATCCATGGGAGAAGATCTGTTTTGGGCTATACGTGGTGGTGGA
GGAGAAAACTTTGGAATCATTGCAGCATGGAAAATCAAACTGGTTGCTGTCCCATCAAAGTCTACTATATTCA
GTGTTAAAAAGAACATGGAGATACATGGGCTTGTCAAGTTATTTAACAAATGGCAAAATATTGCTTACAAGTA
TGTCAAAGATTTAGTACTCATGACTCACTTCATACCAAAGAATATTACAGATAATCATGGGAAGAATAAGACT
ACAGTACATGGTTACTTCTCTTCAATTTTTCATGGTGGAGTGGATAGTCTAGTCGACTTGATGAACAAGAGCT
TTCCTGAGTTGGGTATTAAAAAAACTGATTGCAAAGAATTTAGCTGGATTGATACAACCATCTTCTACAGTGG
TGTTGTAAATTTTAACACTGCTAATTTTAAAAAGGAAATTTTGCTTGATAGATCAGCTGGGAAGAAGACGGCT
TTCTCAATTAAGTTAGACTATGTTAAGAAACCAATTCCAGAAACTGCAATGGTCAAAATTTTGGAAAAATTAT
ATGAAGAAGATGTAGGAGCTGGGATGTATGTGTTGTACCCTTACGGTGGTATAATGGAGGAGATTTCAGAATC
AGCAATTCCATTCCCTCATCGAGCTGGAATAATGTATGAACTTTGGTACACTGCTTCCTGGGAGAAGCAAGAA
GATAATGAAAAGCATATAAACTGGGTTCGAAGTGTTTATAATTTTACGACTCCTTATGTGTCCCAAAATCCAA
GATTGGCGTATCTCAATTATAGGGACCTTGATTTAGGAAAAACTAATCATGCGAGTCCT
>A1_LC378399.1

AATAACTCCCATATCCAAGCAACTATTTTATGCTCTAAGAAAGTTGGCTTGCAGATTCGAACTCGAAGCGGTG
GCCATGATGCTGAGGGTATGTCCTACATATCTCAAGTCCCATTTGTTGTAGTAGACTTGAGAAACATGCATTC
GATCAAAATAGATGTTCATAGCCAAACTGCGTGGGTTGAAGCCGGAGCTACCCTTGGAGAAGTTTATTATTGG
ATCAATGAGAAGAATGAGAATCTTAGTTTTCCTGGTGGGTATTGCCCTACTGTTGGCGTAGGTGGACACTTTA
GTGGAGGAGGCTATGGAGCATTGATGCGAAATTATGGCCTTGCGGCTGATAATATTATTGATGCACACTTAGT
CAATGTTGATGGAAAAGTTCTAGATCGAAAATCCATGGGAGAAGATCTGTTTTGGGCTATACGTGGTGGTGGA
GGAGAAAACTTTGGAATCATTGCAGCATGGAAAATCAAACTGGTTGCTGTCCCATCAAAGTCTACTATATTCA
GTGTTAAAAAGAACATGGAGATACATGGGCTTGTCAAGTTATTTAACAAATGGCAAAATATTGCTTACAAGTA
TGTCAAAGATTTAGTACTCATGACTCACTTCATACCAAAGAATATTACAGATAATCATGGGAAGAATAAGACT
ACAGTACATGGTTACTTCTCTTCAATTTTTCATGGTGGAGTGGATAGTCTAGTCGACTTGATGAACAAGAGCT
TTCCTGAGTTGGGTATTAAAAAAACTGATTGCAAAGAATTTAGCTGGATTGATACAACCATCTTCTACAGTGG
TGTTGTAAATTTTAACACTGCTAATTTTAAAAAGGAAATTTTGCTTGATAGATCAGCTGGGAAGAAGACGGCT
TTCTCAATTAAGTTAGACTATGTTAAGAAACCAATTCCAGAAACTGCAATGGTCAAAATTTTGGAAAAATTAT
ATGAAGAAGATGTAGGAGCTGGGATGTATGTGTTGTACCCTTACGGTGGTATAATGGAGGAGATTTCAGAATC
AGCAATTCCATTCCCTCATCGAGCTGGAATAATGTATGAACTTTGGTACACTGCTTCCTGGGAGAAGCAAGAA
GATAATGAAAAGCATATAAACTGGGTTCGAAGTGTTTATAATTTTACGACTCCTTATGTGTCCCAAAATCCAA
GATTGGCGTATCTCAATTATAGGGACCTTGATTTAGGAAAAACTAATCATGCGAGTCCT
>A1_LC378400.1
AATAACTCCCATATCCAAGCAACTATTTTATGCTCTAAGAAAGTTGGCTTGCAGATTCGAACTCGAAGCGGTG
GCCATGATGCTGAGGGTATGTCCTACATATCTCAAGTCCCATTTGTTGTAGTAGACTTGAGAAACATGCATTC
GATCAAAATAGATGTTCATAGCCAAACTGCGTGGGTTGAAGCCGGAGCTACCCTTGGAGAAGTTTATTATTGG
ATCAATGAGAAGAATGAGAATCTTAGTTTTCCTGGTGGGTATTGCCCTACTGTTGGCGTAGGTGGACACTTTA
GTGGAGGAGGCTATGGAGCATTGATGCGAAATTATGGCCTTGCGGCTGATAATATTATTGATGCACACTTAGT
CAATGTTGATGGAAAAGTTCTAGATCGAAAATCCATGGGAGAAGATCTGTTTTGGGCTATACGTGGTGGTGGA
GGAGAAAACTTTGGAATCATTGCAGCATGGAAAATCAAACTGGTTGCTGTCCCATCAAAGTCTACTATATTCA
GTGTTAAAAAGAACATGGAGATACATGGGCTTGTCAAGTTATTTAACAAATGGCAAAATATTGCTTACAAGTA
TGTCAAAGATTTAGTACTCATGACTCACTTCATACCAAAGAATATTACAGATAATCATGGGAAGAATAAGACT
ACAGTACATGGTTACTTCTCTTCAATTTTTCATGGTGGAGTGGATAGTCTAGTCGACTTGATGAACAAGAGCT
TTCCTGAGTTGGGTATTAAAAAAACTGATTGCAAAGAATTTAGCTGGATTGATACAACCATCTTCTACAGTGG
TGTTGTAAATTTTAACACTGCTAATTTTAAAAAGGAAATTTTGCTTGATAGATCAGCTGGGAAGAAGACGGCT
TTCTCAATTAAGTTAGACTATGTTAAGAAACCAATTCCAGAAACTGCAATGGTCAAAATTTTGGAAAAATTAT
ATGAAGAAGATGTAGGAGCTGGGATGTATGTGTTGTACCCTTACGGTGGTATAATGGAGGAGATTTCAGAATC
AGCAATTCCATTCCCTCATCGAGCTGGAATAATGTATGAACTTTGGTACACTGCTTCCTGGGAGAAGCAAGAA
GATAATGAAAAGCATATAAACTGGGTTCGAAGTGTTTATAATTTTACGACTCCTTATGTGTCCCAAAATCCAA
GATTGGCGTATCTCAATTATAGGGACCTTGATTTAGGAAAAACTAATCATGCGAGTCCT
>A1_LN998182.1
AATAACTCCCATATCCAAGCAACTATTTTATGCTCTAAGAAAGTTGGCTTGCAGATTCGAACTCGAAGCGGTG
GCCATGATGCTGAGGGTATGTCCTACATATCTCAAGTCCCATTTGTTGTAGTAGACTTGAGAAACATGCATTC
GATCAAAATAGATGTTCATAGCCAAACTGCGTGGGTTGAAGCCGGAGCTACCCTTGGAGAAGTTTATTATTGG
ATCAATGAGAAGAATGAGAATCTTAGTTTTCCTGGTGGGTATTGCCCTACTGTTGGCGTAGGTGGACACTTTA
GTGGAGGAGGCTATGGAGCATTGATGCGAAATTATGGCCTTGCGGCTGATAATATTATTGATGCACACTTAGT
CAATGTTGATGGAAAAGTTCTAGATCGAAAATCCATGGGAGAAGATCTGTTTTGGGCTATACGTGGTGGTGGA
GGAGAAAACTTTGGAATCATTGCAGCATGGAAAATCAAACTGGTTGCTGTCCCATCAAAGTCTACTATATTCA
GTGTTAAAAAGAACATGGAGATACATGGGCTTGTCAAGTTATTTAACAAATGGCAAAATATTGCTTACAAGTA
TGTCAAAGATTTAGTACTCATGACTCACTTCATACCAAAGAATATTACAGATAATCATGGGAAGAATAAGACT
ACAGTACATGGTTACTTCTCTTCAATTTTTCATGGTGGAGTGGATAGTCTAGTCGACTTGATGAACAAGAGCT
TTCCTGAGTTGGGTATTAAAAAAACTGATTGCAAAGAATTTAGCTGGATTGATACAACCATCTTCTACAGTGG
TGTTGTAAATTTTAACACTGCTAATTTTAAAAAGGAAATTTTGCTTGATAGATCAGCTGGGAAGAAGACGGCT
TTCTCAATTAAGTTAGACTATGTTAAGAAACCAATTCCAGAAACTGCAATGGTCAAAATTTTGGAAAAATTAT
ATGAAGAAGATGTAGGAGCTGGGATGTATGTGTTGTACCCTTACGGTGGTATAATGGAGGAGATTTCAGAATC
AGCAATTCCATTCCCTCATCGAGCTGGAATAATGTATGAACTTTGGTACACTGCTTCCTGGGAGAAGCAAGAA
GATAATGAAAAGCATATAAACTGGGTTCGAAGTGTTTATAATTTTACGACTCCTTATGTGTCCCAAAATCCAA
GATTGGCGTATCTCAATTATAGGGACCTTGATTTAGGAAAAACTAATCATGCGAGTCCT
>A1_AB212829.1
ATGAATTGCTCAGCATTTTCCTTTTGGTTTGTTTGCAAAATAATATTTTTCTTTCTCTCATTCCATATCCAAA
TTTCAATAGCTAATCCTCGAGAAAACTTCCTTAAATGCTTCTCAAAACATATTCCCAACAATGTAGCAAATCC
AAAACTCGTATACACTCAACACGACCAATTGTATATGTCTCTCCTGAATTCGACAATACAAAATCTTAGATTC
ATCTCTGATACAACCCCAAAACCACTCGTTATTGTCACTCCTTCAAATAACTCCCATATCCAAGCAACTATTT

TATGCTCTAAGAAAGTTGGCTTGCAGATTCGAACTCGAAGCGGTGGCCATGATGCTGAGGGTATGTCCTACAT
TTCTCAAGTCCCATTTGTTGTAGTAGACTTGAGGAACATGCATTCGATCAAAATAGATGTTCATAGCCAAACT
GCGTGGGTTGAAGCCGGAGCTACCCTTGGAGAAGTTTATTATTGGATCAATGAGAAGAATGAGAATCTTAGTT
TTCCTGGTGGGTATTGCCCTACTGTTGGCGTAGGTGGACACTTTAGTGGAGGAGGCTATGGAGCATTGATGCG
AAATTATGGCCTTGCGGCTGATAATATTATTGATGCACACTTAGTCAATGTTGATGGAAAAGTTCTAGATCGA
AAATCCATGGGAGAAGATCTGTTTTGGGCTATACGTGGTGGTGGAGGAGAAAACTTTGGAATCATTGCAGCAT
GGAAAATCAAACTGGTTGCTGTCCCATCAAAGTCTACTATATTCAGTGTTAAAAAGAACATGGAGATACATGG
GCTTGTCAAGTTATTTAACAAATGGCAAAATATTGCTTACAAGTATGACAAAGATTTAGTACTCATGACTCAC
TTCATAACAAAGAATATTACAGATAATCATGGGAAGAATAAGACTACAGTACATGGTTACTTCTCTTCAATTT
TTCATGGTGGAGTGGATAGTCTAGTCGACTTGATGAACAAGAGCTTTCCTGAGTTGGGTATTAAAAAAACTGA
TTGCAAAGAATTTAGCTGGATTGATACAACCATCTTCTACAGTGGTGTTGTAAATTTTAACACTGCTAATTTT
AAAAAGGAAATTTTGCTTGATAGATCAGCTGGGAAGAAGACGGCTTTCTCAATTAAGTTAGACTATGTTAAGA
AACCAATTCCTGAAACTGCAATGGTCAAAATTTTGGAAAAATTATATGAAGAAGATGTAGGAGCTGGGATGTA
TGTGTTGTACCCTTACGGTGGTATAATGGAGGAGATTTCAGAATCAGCAATTCCATTCCCTCATCGAGCTGGA
ATAATGTATGAACTTTGGTACACTGCTTCCTGGGAGAAGCAAGAAGATAATGAAAAGCATATAAACTGGGTTC
GAAGTGTTTATAATTTTACGACTCCTTATGTGTCCCAAAATCCAAGATTGGCGTATCTCAATTATAGGGACCT
TGATTTAGGAAAAACTAATCATGCGAGTCCTAATAATTACACACAAGCACGTATTTGGGGTGAAAAGTATTTT
GGTAAAAATTTTAACAGGTTAGTTAAGGTGAAAACTAAAGTTGATCCCAATAATTTTTTTAGAAACGAACAAA
GTATCCCACCTCTTCCACCGCATCATCAT
>A1_KP970851.1
ATGAATTGCTCAGCATTTTCCTTTTGGTTTGTTTGCAAAATAATATTTTTCTTTCTCTCATTCCATATCC
AAATTTCAATAGCTAATCCTCGAGAAAACTTCCTTAAATGCTTCTCAAAACATATTCCCAACAATGTAGC
AAATCCAAAACTCGTATACACTCAACACGACCAATTGTATATGTCTCTCCTGAATTCGACAATACAAAAT
CTTAGATTCATCTCTGATACAACCCCAAAACCACTCGTTATTGTCACTCCTTCAAATAACTCCCATATCC
AAGCAACTATTTTATGCTCTAAGAAAGTTGGCTTGCAGATTCGAACTCGAAGCGGTGGCCATGATGCTGA
GGGTATGTCCTACATTTCTCAAGTCCCATTTGTTGTAGTAGACTTGAGGAACATGCATTCGATCAAAATA
GATGTTCATAGCCAAACTGCGTGGGTTGAAGCCGGAGCTACCCTTGGAGAAGTTTATTATTGGATCAATG
AGAAGAATGAGAATCTTAGTTTTCCTGGTGGGTATTGCCCTACTGTTGGCGTAGGTGGACACTTTAGTGG
AGGAGGCTATGGAGCATTGATGCGAAATTATGGCCTTGCGGCTGATAATATTATTGATGCACACTTAGTC
AATGTTGATGGAAAAGTTCTAGATCGAAAATCCATGGGAGAAGATCTGTTTTGGGCTATACGTGGTGGTG
GAGGAGAAAACTTTGGAATCATTGCAGCATGGAAAATCAAACTGGTTGCTGTCCCATCAAAGTCTACTAT
ATTCAGTGTTAAAAAGAACATGGAGATACATGGGCTTGTCAAGTTATTTAACAAATGGCAAAATATTGCT
TACAAGTATGACAAAGATTTAGTACTCATGACTCACTTCATAACAAAGAATATTACAGATAATCATGGGA
AGAATAAGACTACAGTACATGGTTACTTCTCTTCAATTTTTCATGGTGGAGTGGATAGTCTAGTCGACTT
GATGAACAAGAGCTTTCCTGAGTTGGGTATTAAAAAAACTGATTGCAAAGAATTTAGCTGGATTGATACA
ACCATCTTCTACAGTGGTGTTGTAAATTTTAACACTGCTAATTTTAAAAAGGAAATTTTGCTTGATAGAT
CAGCTGGGAAGAAGACGGCTTTCTCAATTAAGTTAGACTATGTTAAGAAACCAATTCCTGAAACTGCAAT
GGTCAAAATTTTGGAAAAATTATATGAAGAAGATGTAGGAGCTGGGATGTATGTGTTGTACCCTTACGGT
GGTATAATGGAGGAGATTTCAGAATCAGCAATTCCATTCCCTCATCGAGCTGGAATAATGTATGAACTTT
GGTACACTGCTTCCTGGGAGAAGCAAGAAGATAATGAAAAGCATATAAACTGGGTTCGAAGTGTTTATAA
TTTTACGACTCCTTATGTGTCCCAAAATCCAAGATTGGCGTATCTCAATTATAGGGACCTTGATTTAGGA
AAAACTAATCATGCGAGTCCTAATAATTACACACAAGCACGTATTTGGGGTGAAAAGTATTTTGGTAAAA
ATTTTAACAGGTTAGTTAAGGTGAAAACTAAAGTTGATCCCAATAATTTTTTTAGAAACGAACAAAGTAT
CCCACCTCTTCCACCGCATCATCAT
>A1_MN422092.1
TTTTGGTTTGTTTGCAAAATAATATTTTTCTTTCTCTCATTCCATATCCAAATTTCAATAGCTAATCCTCGAG
AAAACTTCCTTAAATGCTTCTCAAAACATATTCCCAACAATGTAGCAAATCCAAAACTCGTATACACTCAACA
CGACCAATTGTATATGTCTATCCTGAATTCGACAATACAAAATCTTAGATTCATCTCTGATACAACCCCATAA
CCACTCGTTATTGTCACTCCTTCAAATAACTCCCATATCCAAGCAACTATTTTATGCTCTAAGAAAGTTGGCT
TGCAGATTCGAACTCGAAGCGGTTGCCATGATGCTGAGGGTATGTCCTACATTTCTCAACTCCCATTTGTTGT
AGTAGACTTGAGAAACATGCATTCGATCAAAATAGATGTTCATAGCCAAACTGCGTGGGTTGAAGCCGGAGCT
TCCCTTGGAGAAGTTTATTATTGGATCAATGAGAAGAATGAGAATCTTAGTTTTCCTGGTGGGTATTGCCCTA
CTGTTGGCGTAGGTGGACACTTTAGTGGAGGAGGCTATGGAGCATTGATGCGAAATTATGGCCTTGCGGCTGA
TAATATTATTGATGCACACTTAGTCAATGTTGATGGAAAAGTTCTAGATCGAAAATCCATGGGAGAAGATCTG
TTTTGGGCTATACGTGGTGGTGGAGGAGAAAACTTTGGAATCATTGCAGCATGGAAAATCAAACTGGTTGCTG
TCCCATCAAAGTCTACTATATTCAGTGTTAAAAAGAACATGGAGATACATGGGCTTGTCAAGTTATTTAACAA
ATGGCAAAATATTGCTTACAAGTATGACAAAGATTTAGTACTCATGACTCACTTCATAACAAAGAATATTACA
GATAATCATGGGAAGAATAAGACTACAGTACATGGTTACTTCTCTTCAATTTTTCATGGTGGAGTGGATAGTC

TAGTCGACTTGATGAACAAGAGCTTTCCTGAGTTGGGTATTAAAAAAACTGATTGCAAAGAATTTAGCTGGAT
TGATACAACCATCTTCTACAGTGGTGTTGTAAATTTTAACACTGCTAATTTTAAAAAGGAAATTTTGCTTGAT
AGATCAGCTGGGAAGAAGACGGCTTTCTCAATTAAGTTAGACTATGTTAAGAAACCAATTCCAGAAACTGCAA
TGGTCAAAATTTTGGAAAAATTATATGAAGAAGATGTAGGAGCTGGGATGTATGTGTTGTACCCTTACGGTGG
TATAATGGAGGAGATTTCAGAATCAGCAATTCCATTCCCTCATCGAGCTGGAATAATGTATGAACTTTGGTAC
ACTGCTTCCTGGGAGAAGCAAGAAGATAATGAAAAGCATATAAACTGGGTTCGAAGTGTATATAATTTTACGA
CTCCTTATGTGTCCCAAAATCCAAGATTGGCGTATCTCAATTATAGGGACCTTGATTTAGGAAAAACTAATCA
TGCGAGTCCTAATAATTACACACAAGCACGTATTTGGGGTGAAAAGTATTTTGGTAAAAATTTTAACAGGTTA
GTTAAAGTGAAAACTAAAGTTGATCCCAATAATTTTTTTAGAAACGAACAAAGTATCCCACCTCTT
>A1_MN422091.1
TTTTGGTTTGTTTGCAAAATAATATTTTTCTTTCTCCCATTCCATATCCAAATTTCAATAGCTAATCCTCGAG
AAAACTTCCTTAAATGCTTCTCAAAATATATTCCCAACAATGTAGCAAATCCAAAACTCATATACACTCAACA
CGACCAATTGTATATGTCTATCCTGAATTCGACAATACAAAATCTTAGATTCATCTCTGATACAACCCCAAAA
CCACTCGTTATTGTCACTCCTTCAAATAACTCCCATATCCAAGCAACTATTTTATGCTCTAACAAAGTTGGCT
TGCAGATTCGAACTCGAAGCGGTGGCCATGATGCTGAGGGTATGTCCTACATTTCTCAACTCCCATTTGTTGT
AGTAGACTTGAGAAACATGCATTCGATCAAAATAGATGTTCATAGCCAAACTGCGTGGGTTGAAGCCGGAGCT
ACCCTTGGAGAAGTTTATTATTGGATCAATGAGAAGAATGAGAATCTTAGTTTTCCTGGTGGGTATTGCCCTA
CTGTTGGCGTAGGTGGACACTTTCGTGGAGGAGGCTATGGAGCATTGATGCGAAATTATGGCCTTGCGGCTGA
TAATATTATTGATGCACACTTAGTCAATGTTGATGGAAAAGTTCTAGATCGAAAATCCATGGGAGAAGATCTG
TTTTGGGCTATACGTGGTGGTGGAGGAGAAAACTTTGGAATCATTGCAGCATGGAAAATCAAACTGGTTGCTG
TCCCATCAAAGTCTACTATATTCAGTGTTAAAAAGAACATGGAGATACATGGGCTTGTCAAGTTATTTAACAA
ATGGCAAAATATTGCTTACAAGTATGACAAAGATTTAGTACTCATGACTCACTTCATAACAAAGAATATTACA
GATAATCATGGGAAGAATAAGACTACAGTACATGGTTACTTCTCTTCAATTTTTCATGGTGGAGTGGATAGTC
TAGTCGACTTGATGAACAAGAGCTTTCCTGAGTTGGGTATTAAAAAAACTGATTGCAAAGAATTTAGCTGGAT
TGATACAACCATCTTCTACAGTGGTGTTGTAAATTTTAACACTGCTAATTTTAAAAAGGAAATTTTGCTTGAT
AGATCAGCTGGGAAGAAGACGGCTTTCTCAATTAAGTTAGACTATGTTAAGAAACCAATTCCAGAAACTGCAA
TGGTCAAAATTTTGGAAAAATTATATGAAGAAGATGTAGGAGCTGGGATGTATGTGTTGTACCCTTACGGTGG
TATAATGGAGGAGATTTCAGAATCAGCAATTCCATTCCCTCATCGAGCTGGAATAATGTATGAACTTTGGTAC
ACTGCTTCCTGGGAGAAGCAAGAAGATAATGAAAAGCATATAAACTGGGTTCGAAGTGTATATAATTTTACGA
CTCCTTATGTGTCCCAAAATCCAAGATTGGCGTATCTCAATTATAGGGACCTTGATTTAGGAAAAACTAATCA
TGCGAGTCCTAATAATTACACACAAGCACGTATTTGGGGTGAAAAGTATTTTGGTAAAAATTTTAACAGGTTA
GTTAAAGTGAAAACTAAAGTTGATCCCAATAATTTTTTTAGAAACGAACAAAGTATCCCACCTCTT
>A1_MN422088.1
TTTTGGTTTGTTTGCAAAATAATATTTTTCTTTCTCTCATTCCATATCCAAATTTCATTAGCTAATCCTCGAG
AAAACTTCCTTAAATGCTTCTCAAAACATATTCCCAACAATGTAGCAAATCCAAAACTCGTATACACTCAACA
CGACCAATTGTATATGTCTATCCTGAATTCGACAATACAAAATCTTAGATTCATCTCTGATGCAACCCCAAAA
CCACTCGTTATTGTCACTCCTTCAAATAACTCCCATATCCAAGCAACTATTTTATGCTCTAAGAAAGTTGGCT
TGCAGATTCGAACTCGAAGCGGTGGCCATGATGCTGAGGGTATGTCCTACATTTCTCAACTCCCATTTGTTGT
AGTAGACTTGAGAAACATGCATTCGATCAAAATAGATGTTCATAGCCAAACTGCGTGGGTTGAAGCCGGAGCT
ACCCTTGGAGAAGTTTATTATTGGATCAATGAGAAGAATGAGAATCTTAGTTTTCCTGGTGGGTATTGCCCTA
CTGTTGGCGTAGGTGGACACTTTAGTGGAGGAGGCTATGGAGCATTGATGCGAAATTATGGCCTTGCGGCTGA
TAATATTATTGATGCACACTTAGTCAATGTTGATGGAAAAGTTCTAGATCGAAAATCCATGGGAGAAGATCTG
TTTTGGGCTATACGTGGTGGTGGAGGAGAAAACTTTGGAATCATTGCAGCATGGAAAATCAAACTGGTTGCTG
TCCCATCAAAGTCTACTATATTCAGTGTTAAAAAGAACATGGAGATACATGGGCTTGTCAAGTTATTTAACAA
ATGGCAAAATATTGCTTACAAGTATGACAAAGATTTAGTACTCATGACTCACTTCATAATAAAGAATATTACA
GATAATCATGGGAAGAATAAGACTACAGTACATGGTTACTTCTCTTCAATTTTTCATGGTGGAGTGGATAGTC
TAGTCGACTTGATGAACAAGAGCTTTCCTGAGTTGGGTATTAAAAAAACTGATTGCAAAGAATTTAGCTGGAT
TGATACAACCATCTTCTACAGTGGTGTTGTAAATTTTAACACTGCTAATTTTAAAAAGGAAATTTTGCTTGAT
AGATCAGCTGGGAAGAAGACGGCTTTCTCAATTAAGTTAGACTATGTTAAGAAACCAATTCCAGAAACTGCAA
TGGTCAAAATTTTGGAAAAATTATATGAAGAAGATGTAGGAGCTGGGATGTATGTGTTGTACCCTTACGGTGG
TATAATGGAGGAGATTTCGGAATCAGCAATTCCATTCCCTCATCGAGCTGGAATAATGTATGAACTTTGGTAC
ACTGCTTCCTGGGAGAAGCAAGAAGATAATGAAAAGCATATAAACTGGGTTCGAAGTGTATATAATTTTACGA
CTCCTTATGTGTCCCAAAATCCAAGATTGGCGTATCTCAATTATAGGGACCTTGATTTAGGAAAAACTAATCA
TGCGAGTCCTAATAATTACACACAAGCACGTATTTGGGGTGAAAAGTATTTTGGTAAAAATTTTATTAGGTTA
GTTAAAGTGAAAACTAAAGTTGATCCCAATAATTTTTTTAGAAACGAACAAAGTATCCCACCTCTT
>A1_MN422089.1
TTTTGGTTTGTTTGCAAAATAATATTTTTCTTTCTCTCATTCCATATCCAAATTTCATTAGCTAATCCTCGAG
AAAACTTCCTTAAATGCTTCTCAAAACATATTCCCAACAATGTAGCAAATCCAAAACTCGTATACACTCAACA

CGACCAATTGTATATGTCTATCCTGAATTCGACAATACAAAATCTTAGATTCATCTCTGATGCAACCCCAAAA
CCACTCGTTATTGTCACTCCTTCAAATAACTCCCATATCCAAGCAACTATTTTATGCTCTAAGAAAGTTGGTT
TGCAGATTCGAACTCGAAGCGGTGGCCATGATGCTGAGGGTATGTCCTACATTTCTCAACTCCCATTTGTTGT
AGTAGACTTGAGAAACATGCATTCGATCAAAATAGATGTTCATAGCCAAACTGCGTGGGTTGAAGCCGGAGCT
ACCCTTGGAGAAGTTTATTATTGGATCAATGAGAAGAATGAGAATCTTAGTTTTCCTGGTGGGTATTGCCCTA
CTGTTGGCGTAGGTGGACACTTTAGTGGAGGAGGCTATGGAGCATTGATGCGAAATTATGGCCTTGCGGCTGA
TAATATTATTGATGCACACTTAGTCAATGTTGATGGAAAAGTTCTAGATCGAAAATCCATGGGAGAAGATCTG
TTTTGGGCTATACGTGGTGGTGGAGGAGAAAACTTTGGAATCATTGCAGCATGGAAAATCAAACTGGTTGCTG
TCCCATCAAAGTCTACTATATTCAGTGTTAAAAAGAACATGGAGATACATGGGCTTGTCAAGTTATTTAACAA
ATGGCAAAATATTGCTTACAAGTATGACAAAGATTTAGTACTCATGACTCACTTCATAATAAAGAATATTACA
GATAATCATGGGAAGAATAAGACTACAGTACATGGTTACTTCTCTTCAATTTTTCATGGTGGAGTGGATAGTC
TAGTCGACTTGATGAACAAGAGCTTTCCTGAGTTGGGTATTAAAAAAACTGATTGCAAAGAATTTAGCTGGAT
TGATACAACCATCTTCTACAGTGGTGTTGTAAATTTTAACACTGCTAATTTTAAAAAGGAAATTTTGCTTGAT
AGATCAGCTGGGAAGAAGACGGCTTTCTCAATTAAGTTAGACTATGTTAAGAAACCAATTCCAGAAACTGCAA
TGGTCAAAATTTTGGAAAAATTATATGAAGAAGATGTAGGAGCTGGGATGTATGTGTTGTACCCTTACGGTGG
TATAATGGAGGAGATTTCGGAATCAGCAATTCCATTCCCTCATCGAGCTGGAATAATGTATGAACTTTGGTAC
ACTGCTTCCTGGGAGAAGCAAGAAGATAATGAAAAGCATATAAACTGGGTTCGAAGTGTATATAATTTTACGA
CTCCTTATGTGTCCCAAAATCCAAGATTGGCGTATCTCAATTATAGGGACCTTGATTTAGGAAAAACTAATCA
TGCGAGTCCTAATAATTACACACAAGCACGTATTTGGGGTGAAAAGTATTTTGGTAAAAATTTTATTAGGTTA
GTTAAAGTGAAAACTAAAGTTGATCCCAATAATTTTTTTAGAAACGAACAAAGTATCCCACCTCTT

>A2_AB212830.1
ATGAATTGCTCAGCATTTTCCTTTTGGTTTGTTTGCAAAATAATATTTTTCTTTCTCTCATTCAATATCCAAA
TTTCAATAGCTAATCCTCAAGAAAACTTCCTTAAATGCTTCTCGGAATATATTCCTAACAATCCAGCAAATCC
AAAATTCATATACACTCAACACGACCAATTGTATATGTCTGTCCTGAATTCGACAATACAAAATCTTAGATTC
ACCTCTGATACAACCCCAAAACCACTCGTTATTGTCACTCCTTCAAATGTCTCCCATATCCAGGCCAGTATTC
TCTGCTCCAAGAAAGTTGGTTTGCAGATTCGAACTCGAAGCGGTGGCCATGATGCTGAGGGTTTGTCCTACAT
ATCTCAAGTCCCATTTGCTATAGTAGACTTGAGAAACATGCATACGGTCAAAGTAGATATTCATAGCCAAACT
GCGTGGGTTGAAGCCGGAGCTACCCTTGGAGAAGTTTATTATTGGATCAATGAGATGAATGAGAATTTTAGTT
TTCCTGGTGGGTATTGCCCTACTGTTGGCGTAGGTGGACACTTTAGTGGAGGAGGCTATGGAGCATTGATGCG
AAATTATGGCCTTGCGGCTGATAATATCATTGATGCACACTTAGTCAATGTTGATGGAAAAGTTCTAGATCGA
AAATCCATGGGAGAAGATCTATTTTGGGCTATACGTGGTGGAGGAGGAGAAAACTTTGGAATCATTGCAGCAT
GGAAAATCAAACTTGTTGTTGTCCCATCAAAGGCTACTATATTCAGTGTTAAAAAGAACATGGAGATACATGG
GCTTGTCAAGTTATTTAACAAATGGCAAAATATTGCTTACAAGTATGACAAAGATTTAATGCTCACGACTCAC
TTCAGAACTAGGAATATTACAGATAATCATGGGAAGAATAAGACTACAGTACATGGTTACTTCTCTTCCATTT
TTCTTGGTGGAGTGGATAGTCTAGTTGACTTGATGAACAAGAGCTTTCCTGAGTTGGGTATTAAAAAAACTGA
TTGCAAAGAATTGAGCTGGATTGATACAACCATCTTCTACAGTGGTGTTGTAAATTACAACACTGCTAATTTT
AAAAAGGAAATTTTGCTTGATAGATCAGCTGGGAAGAAGACGGCTTTCTCAATTAAGTTAGACTATGTTAAGA
AACTAATACCTGAAACTGCAATGGTCAAAATTTTGGAAAAATTATATGAAGAAGAGGTAGGAGTTGGGATGTA
TGTGTTGTACCCTTACGGTGGTATAATGGATGAGATTTCAGAATCAGCAATTCCATTCCCTCATCGAGCTGGA
ATAATGTATGAACTTTGGTACACTGCTACCTGGGAGAAGCAAGAAGATAACGAAAAGCATATAAACTGGGTTC
GAAGTGTTTATAATTTCACAACTCCTTATGTGTCCCAAAATCCAAGATTGGCGTATCTCAATTATAGGGACCT
TGATTTAGGAAAAACTAATCCTGAGAGTCCTAATAATTACACACAAGCACGTATTTGGGGTGAAAAGTATTTT
GGTAAAAATTTTAACAGGTTAGTTAAGGTGAAAACCAAAGCTGATCCCAATAATTTTTTTAGAAACGAACAAA
GTATCCCACCTCTTCCACCGCATCATCAT
>A2_KT876010.1
ATGAATTGCTCAGCATTTTCCTTTTGGTTTGTTTGCAAAATAATATTTTTCTTTCTCTCATTCAATATCCAAA
TTTCAATAGCTAATCCTCAAGAAAACTTCCTTAAATGCTTCTCGGAATATATTCCTAACAATCCAGCAAATCC
AAAATTCATATACACTCAACACGACCAATTGTATATGTCTGTCCTGAATTCGACAATACAAAATCTTAGATTC
ACCTCTGATACAACCCCAAAACCACTCGTTATTGTCACTCCTTCAAATGTCTCCCATATCCAGGCCAGTATTC
TCTGCTCCAAGAAAGTTGGTTTGCAGATTCGAACTCGAAGCGGTGGCCATGATGCTGAGGGTTTGTCCTACAT
ATCTCAAGTCCCATTTGCTATAGTAGACTTGAGAAACATGCATACGGTCAAAGTAGATATTCATAGCCAAACT
GCGTGGGTTGAAGCCGGAGCTACCCTTGGAGAAGTTTATTATTGGATCAATGAGATGAATGAGAATTTTAGTT
TTCCTGGTGGGTATTGCCCTACTGTTGGCGTAGGTGGACACTTTAGTGGAGGAGGCTATGGAGCATTGATGCG
AAATTATGGCCTTGCGGCTGATAATATCATTGATGCACACTTAGTCAATGTTGATGGAAAAGTTCTAGATCGA
AAATCCATGGGAGAAGATCTATTTTGGGCTATACGTGGTGGAGGAGGAGAAAACTTTGGAATCATTGCAGCAT
GGAAAATCAAACTTGTTGTTGTCCCATCAAAGGCTACTATATTCAGTGTTAAAAAGAACATGGAGATACATGG
GCTTGTCAAGTTATTTAACAAATGGCAAAATATTGCTTACAAGTATGACAAAGATTTAATGCTCACGACTCAC

TTCAGAACTAGGAATATTACAGATAATCATGGGAAGAATAAGACTACAGTACATGGTTACTTCTCTTCCATTT
TTCTTGGTGGAGTGGATAGTCTAGTTGACTTGATGAACAAGAGCTTTCCTGAGTTGGGTATTAAAAAAACTGA
TTGCAAAGAATTGAGCTGGATTGATACAACCATCTTCTACAGTGGTGTTGTAAATTACAACACTGCTAATTTT
AAAAAGGAAATTTTGCTTGATAGATCAGCTGGGAAGAAGACGGCTTTCTCAATTAAGTTAGACTATGTTAAGA
AACTAATACCTGAAACTGCAATGGTCAAAATTTTGGAAAAATTATATGAAGAAGAGGTAGGAGTTGGGATGTA
TGTGTTGTACCCTTACGGTGGTATAATGGATGAGATTTCAGAATCAGCAATTCCATTCCCTCATCGAGCTGGA
ATAATGTATGAACTTTGGTACACTGCTACCTGGGAGAAGCAAGAAGATAACGAAAAGCATATAAACTGGGTTC
GAAGTGTTTATAATTTCACAACTCCTTATGTGTCCCAAAATCCAAGATTGGCGTATCTCAATTATAGGGACCT
TGATTTAGGAAAAACTAATCCTGAGAGTCCTAATAATTACACACAAGCACGTATTTGGGGTGAAAAGTATTTT
GGTAAAAATTTTAACAGGTTAGTTAAGGTGAAAACCAAAGCTGATCCCAATAATTTTTTTAGAAACGAACAAA
GTATCCCACCTCTTCCACCGCATCATCAT
>A2_KT876014.1
ATGAATTGCTCAGCATTTTCCTTTTGGTTTGTTTGCAAAATAATATTTTTCTTTCTCTCATTCAATATCCAAA
TTTCAATAGCTAATCCTCAAGAAAACTTCCTTAAATGCTTCTCGGAATATATTCCTAACAATCCAGCAAATCC
AAAATTCATATACACTCAACACGACCAATTGTATATGTCTGTCCTGAATTCGACAATACAAAATCTTAGATTC
ACCTCTGATACAACCCCAAAACCACTCGTTATTGTCACTCCTTCAAATGTCTCCCATATCCAGGCCAGTATTC
TCTGCTCCAAGAAAGTTGGTTTGCAGATTCGAACTCGAAGCGGTGGCCATGATGCTGAGGGTTTGTCCTACAT
ATCTCAAGTCCCATTTGCTATAGTAGACTTGAGAAACATGCATACGGTCAAAGTAGATATTCATAGCCAAACT
GCGTGGGTTGAAGCCGGAGCTACCCTTGGAGAAGTTTATTATTGGATCAATGAGATGAATGAGAATTTTAGTT
TTCCTGGTGGGTATTGCCCTACTGTTGGCGTAGGTGGACACTTTAGTGGAGGAGGCTATGGAGCATTGATGCG
AAATTATGGCCTTGCGGCTGATAATATCATTGATGCACACTTAGTCAATGTTGATGGAAAAGTTCTAGATCGA
AAATCCATGGGAGAAGATCTATTTTGGGCTATACGTGGTGGAGGAGGAGAAAACTTTGGAATCATTGCAGCAT
GGAAAATCAAACTTGTTGTTGTCCCATCAAAGGCTACTATATTCAGTGTTAAAAAGAACATGGAGATACATGG
GCTTGTCAAGTTATTTAACAAATGGCAAAATATTGCTTACAAGTATGACAAAGATTTAATGCTCACGACTCAC
TTCAGAACTAGGAATATTACAGATAATCATGGGAAGAATAAGACTACAGTACATGGTTACTTCTCTTCCATTT
TTCTTGGTGGAGTGGATAGTCTAGTTGACTTGATGAACAAGAGCTTTCCTGAGTTGGGTATTAAAAAAACTGA
TTGCAAAGAATTGAGCTGGATTGATACAACCATCTTCTACAGTGGTGTTGTAAATTACAACACTGCTAATTTT
AAAAAGGAAATTTTGCTTGATAGATCAGCTGGGAAGAAGACGGCTTTCTCAATTAAGTTAGACTATGTTAAGA
AACTAATACCTGAAACTGCAATGGTCAAAATTTTGGAAAAATTATATGAAGAAGAGGTAGGAGTTGGGATGTA
TGTGTTGTACCCTTACGGTGGTATAATGGATGAGATTTCAGAATCAGCAATTCCATTCCCTCATCGAGCTGGA
ATAATGTATGAACTTTGGTACACTGCTACCTGGGAGAAGCAAGAAGATAACGAAAAGCATATAAACTGGGTTC
GAAGTGTTTATAATTTCACAACTCCTTATGTGTCCCAAAATCCAAGATTGGCGTATCTCAATTATAGGGACCT
TGATTTAGGAAAAACTAATCCTGAGAGTCCTAATAATTACACACAAGCACGTATTTGGGGTGAAAAGTATTTT
GGTAAAAATTTTAACAGGTTAGTTAAGGTGAAAACCAAAGCTGATCCCAATAATTTTTTTAGAAACGAACAAA
GTATCCCACCTCTTCCACCGCATCATCAT
>A2_KT876017.1
ATGAATTGCTCAGCATTTTCCTTTTGGTTTGTTTGCAAAATAATATTTTTCTTTCTCTCATTCAATATCCAAA
TTTCAATAGCTAATCCTCAAGAAAACTTCCTTAAATGCTTCTCGGAATATATTCCTAACAATCCAGCAAATCC
AAAATTCATATACACTCAACACGACCAATTGTATATGTCTGTCCTGAATTCGACAATACAAAATCTTAGATTC
ACCTCTGATACAACCCCAAAACCACTCGTTATTGTCACTCCTTCAAATGTCTCCCATATCCAGGCCAGTATTC
TCTGCTCCAAGAAAGTTGGTTTGCAGATTCGAACTCGAAGCGGTGGCCATGATGCTGAGGGTTTGTCCTACAT
ATCTCAAGTCCCATTTGCTATAGTAGACTTGAGAAACATGCATACGGTCAAAGTAGATATTCATAGCCAAACT
GCGTGGGTTGAAGCCGGAGCTACCCTTGGAGAAGTTTATTATTGGATCAATGAGATGAATGAGAATTTTAGTT
TTCCTGGTGGGTATTGCCCTACTGTTGGCGTAGGTGGACACTTTAGTGGAGGAGGCTATGGAGCATTGATGCG
AAATTATGGCCTTGCGGCTGATAATATCATTGATGCACACTTAGTCAATGTTGATGGAAAAGTTCTAGATCGA
AAATCCATGGGAGAAGATCTATTTTGGGCTATACGTGGTGGAGGAGGAGAAAACTTTGGAATCATTGCAGCAT
GGAAAATCAAACTTGTTGTTGTCCCATCAAAGGCTACTATATTCAGTGTTAAAAAGAACATGGAGATACATGG
GCTTGTCAAGTTATTTAACAAATGGCAAAATATTGCTTACAAGTATGACAAAGATTTAATGCTCACGACTCAC
TTCAGAACTAGGAATATTACAGATAATCATGGGAAGAATAAGACTACAGTACATGGTTACTTCTCTTCCATTT
TTCTTGGTGGAGTGGATAGTCTAGTTGACTTGATGAACAAGAGCTTTCCTGAGTTGGGTATTAAAAAAACTGA
TTGCAAAGAATTGAGCTGGATTGATACAACCATCTTCTACAGTGGTGTTGTAAATTACAACACTGCTAATTTT
AAAAAGGAAATTTTGCTTGATAGATCAGCTGGGAAGAAGACGGCTTTCTCAATTAAGTTAGACTATGTTAAGA
AACTAATACCTGAAACTGCAATGGTCAAAATTTTGGAAAAATTATATGAAGAAGAGGTAGGAGTTGGGATGTA
TGTGTTGTACCCTTACGGTGGTATAATGGATGAGATTTCAGAATCAGCAATTCCATTCCCTCATCGAGCTGGA
ATAATGTATGAACTTTGGTACACTGCTACCTGGGAGAAGCAAGAAGATAACGAAAAGCATATAAACTGGGTTC
GAAGTGTTTATAATTTCACAACTCCTTATGTGTCCCAAAATCCAAGATTGGCGTATCTCAATTATAGGGACCT
TGATTTAGGAAAAACTAATCCTGAGAGTCCTAATAATTACACACAAGCACGTATTTGGGGTGAAAAGTATTTT

GGTAAAAATTTTAACAGGTTAGTTAAGGTGAAAACCAAAGCTGATCCCAATAATTTTTTTAGAAACGAACAAA
GTATCCCACCTCTTCCACCGCATCATCAT
>A2_KT876020.1
ATGAATTGCTCAGCATTTTCCTTTTGGTTTGTTTGCAAAATAATATTTTTCTTTCTCTCATTCAATATCCAAA
TTTCAATAGCTAATCCTCAAGAAAACTTCCTTAAATGCTTCTCGGAATATATTCCTAACAATCCAGCAAATCC
AAAATTCATATACACTCAACACGACCAATTGTATATGTCTGTCCTGAATTCGACAATACAAAATCTTAGATTC
ACCTCTGATACAACCCCAAAACCACTCGTTATTGTCACTCCTTCAAATGTCTCCCATATCCAGGCCAGTATTC
TCTGCTCCAAGAAAGTTGGTTTGCAGATTCGAACTCGAAGCGGTGGCCATGATGCTGAGGGTTTGTCCTACAT
ATCTCAAGTCCCATTTGCTATAGTAGACTTGAGAAACATGCATACGGTCAAAGTAGATATTCATAGCCAAACT
GCGTGGGTTGAAGCCGGAGCTACCCTTGGAGAAGTTTATTATTGGATCAATGAGATGAATGAGAATTTTAGTT
TTCCTGGTGGGTATTGCCCTACTGTTGGCGTAGGTGGACACTTTAGTGGAGGAGGCTATGGAGCATTGATGCG
AAATTATGGCCTTGCGGCTGATAATATCATTGATGCACACTTAGTCAATGTTGATGGAAAAGTTCTAGATCGA
AAATCCATGGGAGAAGATCTATTTTGGGCTATACGTGGTGGAGGAGGAGAAAACTTTGGAATCATTGCAGCAT
GGAAAATCAAACTTGTTGTTGTCCCATCAAAGGCTACTATATTCAGTGTTAAAAAGAACATGGAGATACATGG
GCTTGTCAAGTTATTTAACAAATGGCAAAATATTGCTTACAAGTATGACAAAGATTTAATGCTCACGACTCAC
TTCAGAACTAGGAATATTACAGATAATCATGGGAAGAATAAGACTACAGTACATGGTTACTTCTCTTCCATTT
TTCTTGGTGGAGTGGATAGTCTAGTTGACTTGATGAACAAGAGCTTTCCTGAGTTGGGTATTAAAAAAACTGA
TTGCAAAGAATTGAGCTGGATTGATACAACCATCTTCTACAGTGGTGTTGTAAATTACAACACTGCTAATTTT
AAAAAGGAAATTTTGCTTGATAGATCAGCTGGGAAGAAGACGGCTTTCTCAATTAAGTTAGACTATGTTAAGA
AACTAATACCTGAAACTGCAATGGTCAAAATTTTGGAAAAATTATATGAAGAAGAGGTAGGAGTTGGGATGTA
TGTGTTGTACCCTTACGGTGGTATAATGGATGAGATTTCAGAATCAGCAATTCCATTCCCTCATCGAGCTGGA
ATAATGTATGAACTTTGGTACACTGCTACCTGGGAGAAGCAAGAAGATAACGAAAAGCATATAAACTGGGTTC
GAAGTGTTTATAATTTCACAACTCCTTATGTGTCCCAAAATCCAAGATTGGCGTATCTCAATTATAGGGACCT
TGATTTAGGAAAAACTAATCCTGAGAGTCCTAATAATTACACACAAGCACGTATTTGGGGTGAAAAGTATTTT
GGTAAAAATTTTAACAGGTTAGTTAAGGTGAAAACCAAAGCTGATCCCAATAATTTTTTTAGAAACGAACAAA
GTATCCCACCTCTTCCACCGCATCATCAT
>A2_KT876032.1
ATGAATTGCTCAGCATTTTCCTTTTGGTTTGTTTGCAAAATAATATTTTTCTTTCTCTCATTCAATATCCAAA
TTTCAATAGCTAATCCTCAAGAAAACTTCCTTAAATGCTTCTCGGAATATATTCCTAACAATCCAGCAAATCC
AAAATTCATATACACTCAACACGACCAATTGTATATGTCTGTCCTGAATTCGACAATACAAAATCTTAGATTC
ACCTCTGATACAACCCCAAAACCACTCGTTATTGTCACTCCTTCAAATGTCTCCCATATCCAGGCCAGTATTC
TCTGCTCCAAGAAAGTTGGTTTGCAGATTCGAACTCGAAGCGGTGGCCATGATGCTGAGGGTTTGTCCTACAT
ATCTCAAGTCCCATTTGCTATAGTAGACTTGAGAAACATGCATACGGTCAAAGTAGATATTCATAGCCAAACT
GCGTGGGTTGAAGCCGGAGCTACCCTTGGAGAAGTTTATTATTGGATCAATGAGATGAATGAGAATTTTAGTT
TTCCTGGTGGGTATTGCCCTACTGTTGGCGTAGGTGGACACTTTAGTGGAGGAGGCTATGGAGCATTGATGCG
AAATTATGGCCTTGCGGCTGATAATATCATTGATGCACACTTAGTCAATGTTGATGGAAAAGTTCTAGATCGA
AAATCCATGGGAGAAGATCTATTTTGGGCTATACGTGGTGGAGGAGGAGAAAACTTTGGAATCATTGCAGCAT
GGAAAATCAAACTTGTTGTTGTCCCATCAAAGGCTACTATATTCAGTGTTAAAAAGAACATGGAGATACATGG
GCTTGTCAAGTTATTTAACAAATGGCAAAATATTGCTTACAAGTATGACAAAGATTTAATGCTCACGACTCAC
TTCAGAACTAGGAATATTACAGATAATCATGGGAAGAATAAGACTACAGTACATGGTTACTTCTCTTCCATTT
TTCTTGGTGGAGTGGATAGTCTAGTTGACTTGATGAACAAGAGCTTTCCTGAGTTGGGTATTAAAAAAACTGA
TTGCAAAGAATTGAGCTGGATTGATACAACCATCTTCTACAGTGGTGTTGTAAATTACAACACTGCTAATTTT
AAAAAGGAAATTTTGCTTGATAGATCAGCTGGGAAGAAGACGGCTTTCTCAATTAAGTTAGACTATGTTAAGA
AACTAATACCTGAAACTGCAATGGTCAAAATTTTGGAAAAATTATATGAAGAAGAGGTAGGAGTTGGGATGTA
TGTGTTGTACCCTTACGGTGGTATAATGGATGAGATTTCAGAATCAGCAATTCCATTCCCTCATCGAGCTGGA
ATAATGTATGAACTTTGGTACACTGCTACCTGGGAGAAGCAAGAAGATAACGAAAAGCATATAAACTGGGTTC
GAAGTGTTTATAATTTCACAACTCCTTATGTGTCCCAAAATCCAAGATTGGCGTATCTCAATTATAGGGACCT
TGATTTAGGAAAAACTAATCCTGAGAGTCCTAATAATTACACACAAGCACGTATTTGGGGTGAAAAGTATTTT
GGTAAAAATTTTAACAGGTTAGTTAAGGTGAAAACCAAAGCTGATCCCAATAATTTTTTTAGAAACGAACAAA
GTATCCCACCTCTTCCACCGCATCATCAT
>A2_MG996402.1
TTTTGGTTTGTTTGCAAAATAATATTTTTCTTTCTCTCATTCAATATCCAAATTTCAATAGCTAATCCTCAAG
AAAACTTCCTTAAATGCTTCTCGGAATATATTCCTAACAATCCAGCAAATCCAAAATTCATATACACTCAACA
CGACCAATTGTATATGTCTGTCCTGAATTCGACAATACAAAATCTTAGATTCACCTCTGATACAACCCCAAAA
CCACTCGTTATTGTCACTCCTTCAAATGTCTCCCATATCCAGGCCAGTATTCTCTGCTCCAAGAAAGTTGGTT
TGCAGATTCGAACTCGAAGCGGTGGCCATGATGCTGAGGGTTTGTCCTACATATCTCAAGTCCCATTTGCTAT
AGTAGACTTGAGAAACATGCATACGGTCAAAGTAGATATTCATAGCCAAACTGCGTGGGTTGAAGCCGGAGCT
ACCCTTGGAGAAGTTTATTATTGGATCAATGAGATGAATGAGAATTTTAGTTTTCCTGGTGGGTATTGCCCTA

CTGTTGGCGTAGGTGGACACTTTAGTGGAGGAGGCTATGGAGCATTGATGCGAAATTATGGCCTTGCGGCTGA
TAATATCATTGATGCACACTTAGTCAATGTTGATGGAAAAGTTCTAGATCGAAAATCCATGGGAGAAGATCTA
TTTTGGGCTATACGTGGTGGAGGAGGAGAAAACTTTGGAATCATTGCAGCATGGAAAATCAAACTTGTTGTTG
TCCCATCAAAGGCTACTATATTCAGTGTTAAAAAGAACATGGAGATACATGGGCTTGTCAAGTTATTTAACAA
ATGGCAAAATATTGCTTACAAGTATGACAAAGATTTAATGCTCACGACTCACTTCAGAACTAGGAATATTACA
GATAATCATGGGAAGAATAAGACTACAGTACATGGTTACTTCTCTTCCATTTTTCTTGGTGGAGTGGATAGTC
TAGTTGACTTGATGAACAAGAGCTTTCCTGAGTTGGGTATTAAAAAAACTGATTGCAAAGAATTGAGCTGGAT
TGATACAACCATCTTCTACAGTGGTGTTGTAAATTACAACACTGCTAATTTTAAAAAGGAAATTTTGCTTGAT
AGATCAGCTGGGAAGAAGACGGCTTTCTCAATTAAGTTAGACTATGTTAAGAAACTAATACCTGAAACTGCAA
TGGTCAAAATTTTGGAAAAATTATATGAAGAAGAGGTAGGAGTTGGGATGTATGTGTTGTACCCTTACGGTGG
TATAATGGATGAGATTTCAGAATCAGCAATTCCATTCCCTCATCGAGCTGGAATAATGTATGAACTTTGGTAC
ACTGCTACCTGGGAGAAGCAAGAAGATAACGAAAAGCATATAAACTGGGTTCGAAGTGTTTATAATTTCACAA
CTCCTTATGTGTCCCAAAATCCAAGATTGGCGTATCTCAATTATAGGGACCTTGATTTAGGAAAAACTAATCC
TGAGAGTCCTAATAATTACACACAAGCACGTATTTGGGGTGAAAAGTATTTTGGTAAAAATTTTAACAGGTTA
GTTAAGGTGAAAACCAAAGCTGATCCCAATAATTTTTTTAGAAACGAACAAAGTATCCCACCTCTT
>A2_MG996405.1
TTTTGGTTTGTTTGCAAAATAATATTTTTCTTTCTCTCATTCAATATCCAAATTTCAATAGCTAATCCTCAAG
AAAACTTCCTTAAATGCTTCTCGGAATATATTCCTAACAATCCAGCAAATCCAAAATTCATATACACTCAACA
CGACCAATTGTATATGTCTGTCCTGAATTCGACAATACAAAATCTTAGATTCACCTCTGATACAACCCCAAAA
CCACTCGTTATTGTCACTCCTTCAAATGTCTCCCATATCCAGGCCAGTATTCTCTGCTCCAAGAAAGTTGGTT
TGCAGATTCGAACTCGAAGCGGTGGCCATGATGCTGAGGGTTTGTCCTACATATCTCAAGTCCCATTTGCTAT
AGTAGACTTGAGAAACATGCATACGGTCAAAGTAGATATTCATAGCCAAACTGCGTGGGTTGAAGCCGGAGCT
ACCCTTGGAGAAGTTTATTATTGGATCAATGAGATGAATGAGAATTTTAGTTTTCCTGGTGGGTATTGCCCTA
CTGTTGGCGTAGGTGGACACTTTAGTGGAGGAGGCTATGGAGCATTGATGCGAAATTATGGCCTTGCGGCTGA
TAATATCATTGATGCACACTTAGTCAATGTTGATGGAAAAGTTCTAGATCGAAAATCCATGGGAGAAGATCTA
TTTTGGGCTATACGTGGTGGAGGAGGAGAAAACTTTGGAATCATTGCAGCATGGAAAATCAAACTTGTTGTTG
TCCCATCAAAGGCTACTATATTCAGTGTTAAAAAGAACATGGAGATACATGGGCTTGTCAAGTTATTTAACAA
ATGGCAAAATATTGCTTACAAGTATGACAAAGATTTAATGCTCACGACTCACTTCAGAACTAGGAATATTACA
GATAATCATGGGAAGAATAAGACTACAGTACATGGTTACTTCTCTTCCATTTTTCTTGGTGGAGTGGATAGTC
TAGTTGACTTGATGAACAAGAGCTTTCCTGAGTTGGGTATTAAAAAAACTGATTGCAAAGAATTGAGCTGGAT
TGATACAACCATCTTCTACAGTGGTGTTGTAAATTACAACACTGCTAATTTTAAAAAGGAAATTTTGCTTGAT
AGATCAGCTGGGAAGAAGACGGCTTTCTCAATTAAGTTAGACTATGTTAAGAAACTAATACCTGAAACTGCAA
TGGTCAAAATTTTGGAAAAATTATATGAAGAAGAGGTAGGAGTTGGGATGTATGTGTTGTACCCTTACGGTGG
TATAATGGATGAGATTTCAGAATCAGCAATTCCATTCCCTCATCGAGCTGGAATAATGTATGAACTTTGGTAC
ACTGCTACCTGGGAGAAGCAAGAAGATAACGAAAAGCATATAAACTGGGTTCGAAGTGTTTATAATTTCACAA
CTCCTTATGTGTCCCAAAATCCAAGATTGGCGTATCTCAATTATAGGGACCTTGATTTAGGAAAAACTAATCC
TGAGAGTCCTAATAATTACACACAAGCACGTATTTGGGGTGAAAAGTATTTTGGTAAAAATTTTAACAGGTTA
GTTAAGGTGAAAACCAAAGCTGATCCCAATAATTTTTTTAGAAACGAACAAAGTATCCCACCTCTT
>A2_KJ469379.1
ATGAATTGCTCAGCATTCTCCTTTTGGTTTGTTTGCAAAATAATATTTTTCTTTCTCTCATTCAATATCCAAA
TTTCAATAGCTAATCCTCAAGAAAACTTCCTTAAATGCTTCTCGGAATATATTCCTAACAATCCAGCAAATCC
AAAATTCATATACACTCAACACGACCAATTGTATATGTCTGTCCTGAATTCGACAATACAAAATCTTAGATTC
ACCTCTGATACAACCCCAAAACCACTCGTTATTGTCACTCCTTCAAATGTCTCCCATATCCAGGCCAGTATTC
TCTGCTCCAAGAAAGTTGGTTTGCAGATTCGAACTCGAAGCGGTGGCCATGATGCTGAGGGTTTGTCCTACAT
ATCTCAAGTCCCATTTGCTATAGTAGACTTGAGAAACATGCATACGGTCAAAGTAGATATTCATAGCCAAACT
GCGTGGGTTGAAGCCGGAGCTACCCTTGGAGAAGTTTATTATTGGATCAATGAGATGAATGAGAATTTTAGTT
TTCCTGGTGGGTATTGCCCTACTGTTGGCGTAGGTGGACACTTTAGTGGAGGAGGCTATGGAGCATTGATGCG
AAATTATGGCCTTGCGGCTGATAATATCATTGATGCACACTTAGTCAATGTTGATGGAAAAGTTCTAGATCGA
AAATCCATGGGAGAAGATCTATTTTGGGCTATACGTGGTGGAGGAGGAGAAAACTTTGGAATCATTGCAGCAT
GGAAAATCAAACTTGTTGTTGTCCCATCAAAGGCTACTATATTCAGTGTTAAAAAGAACATGGAGATACATGG
GCTTGTCAAGTTATTTAACAAATGGCAAAATATTGCTTACAAGTATGACAAAGATTTAATGCTCACGACTCAC
TTCAGAACTAGGAATATTACAGATAATCATGGGAAGAATAAGACTACAGTACATGGTTACTTCTCTTCCATTT
TTCTTGGTGGAGTGGATAGTCTAGTTGACTTGATGAACAAGAGCTTTCCTGAGTTGGGTATTAAAAAAACTGA
TTGCAAAGAATTGAGCTGGATTGATACAACCATCTTCTACAGTGGTGTTGTAAATTACAACACTGCTAATTTT
AAAAAGGAAATTTTGCTTGATAGATCAGCTGGGAAGAAGACGGCTTTCTCAATTAAGTTAGACTATGTTAAGA
AACTAATACCTGAAACTGCAATGGTCAAAATTTTGGAAAAATTATATGAAGAAGAGGTAGGAGTTGGGATGTA
TGTGTTGTACCCTTACGGTGGTATAATGGATGAGATTTCAGAATCAGCAATTCCATTCCCTCATCGAGCTGGA
ATAATGTATGAACTTTGGTACACTGCTACCTGGGAGAAGCAAGAAGATAACGAAAAGCATATAAACTGGGTTC

GAAGTGTTTATAATTTCACAACTCCTTATGTGTCCCAAAATCCAAGATTGGCGTATCTCAATTATAGGGACCT
TGATTTAGGAAAAACTAATCCTGAGAGTCCTAATAATTACACACAAGCACGTATTTGGGGTGAAAAGTATTTT
GGTAAAAATTTTAACAGGTTAGTTAAGGTGAAAACCAAAGCTGATCCCAATAATTTTTTTAGAAACGAACAAA
GTATCCCACCTCTTCCACCGCGTCATCATTAA
>A2_MG996404.1
TTTTGGTTTGTTTGCAAAATAATATTTTTCTTTCTCTCATTCAATATCCAAATTTCAATAGCTAATCCTCAAG
AAAWCTTCCTTAAATGCTTCTCGGAATATATTCCTAACAATCCAGCAAATCCAAAATTCATATACACTCAACA
CGACCAATTGTATATGTCTGTCCTGAATTCGACAATACAAAATCTTAGATTCACCTCTGATACAACCCCAAAA
CCACTCGTTATTGTCACTCCTTCAAATGTCTCCCATATCCAGGCCAGTATTCTCTGCTCCAAGAAAGTTGGTT
TGCAGATTCGAACTCGAAGCGGTGGCCATGATGCTGAGGGTTTGTCCTACATATCTCAAGTCCCATTTGCTAT
AGTAGACTTGAGAAACATGCATACGGTCAAAGTAGATATTCATAGCCAAACTGCGTGGGTTGAAGCCGGAGCT
ACCCTTGGAGAAGTTTATTATTGGATCAATGAGATGAATGAGAATTTTAGTTTTCCTGGTGGGTATTGCCCTA
CTGTTGGCGTAGGTGGACACTTTAGTGGAGGAGGCTATGGAGCATTGATGCGAAATTATGGCCTTGCGGCTGA
TAATATCATTGATGCACACTTAGTCAATGTTGATGGAAAAGTTCTAGATCGAAAATCCATGGGAGAAGATCTA
TTTTGGGCTATACGTGGTGGAGGAGGAGAAAACTTTGGAATCATTGCAGCATGGAAAATCAAACTTGTTGTTG
TCCCATCAAAGGCTACTATATTCAGTGTTAAAAAGAACATGGAGATACATGGGCTTGTCAAGTTATTTAACAA
ATGGCAAAATATTGCTTACAAGTATGACAAAGATTTAATGCTCACGACTCACTTCAGAACTAGGAATATTACA
GATAATCATGGGAAGAATAAGACTACAGTACATGGTTACTTCTCTTCCATTTTTCTTGGTGGAGTGGATAGTC
TAGTTGACTTGATGAACAAGAGCTTTCCTGAGTTGGGTATTAAAAAAACTGATTGCAAAGAATTGAGCTGGAT
TGATACAACCATCTTCTACAGTGGTGTTGTAAATTACAACACTGCTAATTTTAAAAAGGAAATTTTGCTTGAT
AGATCAGCTGGGAAGAAGACGGCTTTCTCAATTAAGTTAGACTATGTTAAGAAACTAATACCTGAAACTGCAA
TGGTCAAAATTTTGGAAAAATTATATGAAGAAGAGGTAGGAGTTGGGATGTATGTGTTGTACCCTTACGGTGG
TATAATGGATGAGATTTCAGAATCAGCAATTCCATTCCCTCATCGAGCTGGAATAATGTATGAACTTTGGTAC
ACTGCTACCTGGGAGAAGCAAGAAGATAACGAAAAGCATATAAACTGGGTTCGAAGTGTTTATAATTTCACAA
CTCCTTATGTGTCCCAAAATCCAAGATTGGCGTATCTCAATTATAGGGACCTTGATTTAGGAAAAACTAATCC
TGAGAGTCCTAATAATTACACACAAGCACGTATTTGGGGTGAAAAGTATTTTGGTAAAAATTTTAACAGGTTA
GTTAAGGTGAAAACCAAAGCTGATCCCAATAATTTTTTTAGAAACGAACAAAGTATCCCACCTCTT
>A2_MG996408.1
TTTTGGTTTGTTTGCAAAATAATATTTTTCTTTCTCTCATTCAATATCCAAATTTCAWTAGCTAATCCTCAAG
AAAACTTCCTTAAATGCTTCTCGGAATATATTCCTAACAATCCAGCAAATCCAAAATTCATATACACTCAACA
CGACCAATTGTATATGTCTGTCCTGAATTCGACAATACAAAATCTTAGATTCACCTCTGATACAACCCCAAAA
CCACTCGTTATTGTCACTCCTTCAAATGTCTCCCATATCCAGGCCAGTATTCTCTGCTCCAAGAAAGTTGGTT
TGCAGATTCGAACTCGAAGCGGTGGCCATGATGCTGAGGGTTTGTCCTACATATCTCAAGTCCCATTTGCTAT
AGTAGACTTGAGAAACATGCATACGGTCAAAGTAGATATTCATAGCCAAACTGCGTGGGTTGAAGCCGGAGCT
ACCCTTGGAGAAGTTTATTATTGGATCAATGAGATGAATGAGAATTTTAGTTTTCCTGGTGGGTATTGCCCTA
CTGTTGGCGTAGGTGGACACTTTAGTGGAGGAGGCTATGGAGCATTGATGCGAAATTATGGCCTTGCGGCTGA
TAATATCATTGATGCACACTTAGTCAATGTTGATGGAAAAGTTCTAGATCGAAAATCCATGGGAGAAGATCTA
TTTTGGGCTATACGTGGTGGAGGAGGAGAAAACTTTGGAATCATTGCAGCATGGAAAATCAAACTTGTTGTTG
TCCCATCAAAGGCTACTATATTCAGTGTTAAAAAGAACATGGAGATACATGGGCTTGTCAAGTTATTTAACAA
ATGGCAAAATATTGCTTACAAGTATGACAAAGATTTAATGCTCACGACTCACTTCAGAACTAGGAATATTACA
GATAATCATGGGAAGAATAAGASTACAGTACATGGTTACTTCTCTTCCATTTTTCTTGGTGGAGTGGATAGTC
TAGTTGACTTGATGAACAAGAGCTTTCCTGAGTTGGGTATTAAAAAAACTGATTGCAAAGAATTGAGCTGGAT
TGATACAACCATCTTCTACAGTGGTGTTGTAAATTACAACACTGCTAATTTTAAAAAGGAAATTTTGCTTGAT
AGATCAGCTGGGAAGAAGACGGCTTTCTCAATTAAGTTAGACTATGTTAAGAAACTAATACCTGAAACTGCAA
TGGTCAAAATTTTGGAAAAATTATATGAAGAAGAGGTAGGAGTTGGGATGTATGTGTTGTACCCTTACGGTGG
TATAATGGATGAGATTTCAGAATCAGCAATTCCATTCCCTCATCGAGCTGGAATAATGTATGAACTTTGGTAC
ACTGCTACCTGGGAGAAGCAAGAAGATAACGAAAAGCATATAAACTGGGTTCGAAGTGTTTATAATTTCACAA
CTCCTTATGTGTCCCAAAATCCAAGATTGGCGTATCTCAATTATAGGGACCTTGATTTAGGAAAAACTAATCC
TGAGAGTCCTAATAATTACACACAAGCACGTATTTGGGGTGAAAAGTATTTTGGTAAAAATTTTAACAGGTTA
GTTAAGGTGAAAACCAAAGCTGATCCCAATAATTTTTTTAGAAACGAACAAAGTATCCCACCTCTT
>A2_MG996410.1
TTTTGGTTTGTTTGCAAAATAATATTTTTCTTTCTCTCATTCAATATCCAAATTTCAWTAGCTAATCCTCAAG
AAAACTTCCTTAAATGCTTCTCGGAATATATTCCTAACAATCCAGCAAATCCAAAATTCATATACACTCAACA
CGACCAATTGTATATGTCTGTCCTGAATTCGACAATACAAAATCTTAGATTCACCTCTGATACAACCCCAAAA
CCACTCGTTATTGTCACTCCTTCAAATGTCTCCCATATCCAGGCCAGTATTCTCTGCTCCAAGAAAGTTGGTT
TGCAGATTCGAACTCGAAGCGGTGGCCATGATGCTGAGGGTTTGTCCTACATATCTCAAGTCCCATTTGCTAT
AGTAGACTTGAGAAACATGCATACGGTCAAAGTAGATATTCATAGCCAAACTGCGTGGGTTGAAGCCGGAGCT
ACCCTTGGAGAAGTTTATTATTGGATCAATGAGATGAATGAGAATTTTAGTTTTCCTGGTGGGTATTGCCCTA

CTGTTGGCGTAGGTGGACACTTTAGTGGAGGAGGCTATGGAGCATTGATGCGAAATTATGGCCTTGCGGCTGA
TAATATCATTGATGCACACTTAGTCAATGTTGATGGAAAAGTTCTAGATCGAAAATCCATGGGAGAAGATCTA
TTTTGGGCTATACGTGGTGGAGGAGGAGAAAACTTTGGAATCATTGCAGCATGGAAAATCAAACTTGTTGTTG
TCCCATCAAAGGCTACTATATTCAGTGTTAAAAAGAACATGGAGATACATGGGCTTGTCAAGTTATTTAACAA
ATGGCAAAATATTGCTTACAAGTATGACAAAGATTTAATGCTCACGACTCACTTCAGAACTAGGAATATTACA
GATAATCATGGGAAGAATAAGACTACAGTACATGGTTACTTCTCTTCCATTTTTCTTGGTGGAGTGGATAGTC
TAGTTGACTTGATGAACAAGAGCTTTCCTGAGTTGGGTATTAAAAAAACTGATTGCAAAGAATTGAGCTGGAT
TGATACAACCATCTTCTACAGTGGTGTTGTAAATTACAACACTGCTAATTTTAAAAAGGAAATTTTGCTTGAT
AGATCAGCTGGGAAGAAGACGGCTTTCTCAATTAAGTTAGACTATGTTAAGAAACTAATACCTGAAACTGCAA
TGGTCAAAATTTTGGAAAAATTATATGAAGAAGAGGTAGGAGTTGGGATGTATGTGTTGTACCCTTACGGTGG
TATAATGGATGAGATTTCAGAATCAGCAATTCCATTCCCTCATCGAGCTGGAATAATGTATGAACTTTGGTAC
ACTGCTACCTGGGAGAAGCAAGAAGATAACGAAAAGCATATAAACTGGGTTCGAAGTGTTTATAATTTCACAA
CTCCTTATGTGTCCCAAAATCCAAGATTGGCGTATCTCAATTATAGGGACCTTGATTTAGGAAAAACTAATCC
TGAGAGTCCTAATAATTACACACAAGCACGTATTTGGGGTGAAAAGTATTTTGGTAAAAATTTTAACAGGTTA
GTTAAGGTGAAAACCAAAGCTGATCCCAATAATTTTTTTAGAAACGAACAA
>A2_MG996403.1
TTTTGGTTTGTTTGCAAAATAATATTTTTCTTTCTCTCATTCAATATCCAAATTTCAATAGCTAATCCTCAAG
AAAACTTCCTTAAATGCTTCTCGGAATATATTCCTAACAATCCAGCAAATCCAAAATTCATATACACTCAACA
CGACCAATTGTATATGTCTGTCCTGAATTCGACAATACAAAATCTTAGATTCACCTCTGATACAACCCCAAAA
CCACTCGTTATTGTCACTCCTTCAAATGTCTCCCATATCCAGGCCAGTATTCTCTGCTCCAAGAAAGTTGGTT
TGCAGATTCGAACTCGAAGCGGTGGCCATGATGCTGAGGGTTTGTCCTACATATCTCAAGTCCCATTTGCTAT
AGTAGACTTGAGAAACATGCATACGGTCAAAGTAGATATTCATAGCCAAACTGCGTGGGTTGAAGCCGGAGCT
ACCCTTGGAGAAGTTTATTATTGGATCAATGAGATGAATGAGAATTTTAGTTTTCCTGGTGGGTATTGCCCTA
CTGTTGGCGTAGGTGGACACTTTAGTGGAGGAGGCTATGGAGCATTGATGCGAAATTATGGCCTTGCGGCTGA
TAATATCATTGATGCACACTTAGTCAATGTTGATGGAAAAGTTCTAGATCGAAAATCCATGGGAGAAGATCTA
TTTTGGGCTATACGTGGTGGAGGAGGAGAAAACTTTGGAATCATTGCAGCATGGAAAATCAAACTTGTTGTTG
TCCCATCAAAGGCTACTATATTCAGTGTTAAAAAGAACATGGAGATACATGGGCTTTTCAAGTTATTTAACAA
ATGGCAAAATATTGCTTACAAGTATGACAAAGATTTAATGCTCACGACTCACTTCAGAACTAGGAATATTACA
GATAATCATGGGAAGAATAAGACTACAGTACATGGTTACTTCTCTTCCATTTTTCTTGGTGGAGTGGATAGTC
TAGTTGACTTGATGAACAAGAGCTTTCCTGAGTTGGGTATTAAAAAAACTGATTGCAAAGAATTGAGCTGGAT
TGATACAACCATCTTCTACAGTGGTGTTGTAAATTACAACACTGCTAATTTTAAAAAGGAAATTTTGCTTGAT
AGATCAGCTGGGAAGAAGACGGCTTTCTCAATTAAGTTAGACTATGTTAAGAAACTAATACCTGAAACTGCAA
TGGTCAAAATTTTGGAAAAATTATATGAAGAAGAGGTAGGAGTTGGGATGTATGTGTTGTACCCTTACGGTGG
TATAATGGATGAGATTTCAGAATCAGCAATTCCATTCCCTCATCGAGCTGGAATAATGTATGAACTTTGGTAC
ACTGCTACCTGGGAGAAGCAAGAAGATAACGAAAAGCATATAAACTGGGTTCGAAGTGTTTATAATTTCACAA
CTCCTTATGTGTCCCAAAATCCAAGATTGGCGTATCTCAATTATAGGGACCTTGATTTAGGAAAAACTAATCC
TGAGAGTCCTAATAATTACACACAAGCACGTATTTGGGGTGAAAAGTATTTTGGTAAAAATTTTAACAGGTTA
GTTAAGGTGAAAACCAAAGCTGATCCCAATAATTTTTTTAGAAACGAACAAAGTATCCCACCTCTT
>A2_KJ469380.1
ATGAATTGCTCAGCATTCTCCTTTTGGTTTGTTTGCAAAATAATATTTTTCTTTCTCTCATTCAATATCCAAA
TTTCAATAGCTAATCCTCAAGAAAACTTCCTTAAATGCTTCTCGGAATATATTCCTAACAATCCAGCAAATCC
AAAATTCATATACACTCAACACGACCAATTGTATATGTCTGTCCTGAATTCGACAATACAAAATCTTAGATTC
ACCTCTGATACAACCCCAAAACCACTCGTTATTGTCACTCCTTCAAATGTCTCCCATATCCAGGCCAGTATTC
TCTGCTCCAAGAAAGTTGGTTTGCAGATTCGAACTCGAAGCGGTGGCCATGATGCTGAGGGTTTGTCCTACAT
ATCTCAAGTCCCATTTGCTATAGTAGACTTGAGAAACATGCATACGGTCAAAGTAGATATTCATAGCCAAACT
GCGTGGGTTGAAGCCGGAGCTACCCTTGGAGAAGTTTATTATTGGATCAATGAGATGAATGAGAATTTTAGTT
TTCCTGGTGGGTATTGCCCTACTGTTGGCGTAGGTGGACACTTTAGTGGAGGAGGCTATGGAGCATTGATGCG
AAATTATGGCCTTGCGGCTGATAATATCATTGATGCACACTTAGTCAATGTTGATGGAAAAGTTCTAGATCGA
AAATCCATGGGAGAAGATCTATTTTGGGCTATACGTGGTGGAGGAGGAGAAAACTTTGGAATCATTGCAGCAT
GGAAAATCAAACTTGTTGTTGTCCCATCAAAGGCTACTATATTCAGTGTTAAAAAGAACATGGAGATACATGG
GCTTGTCAAGTTATTTAACAAATGGCAAAATATTGCTTACAAGTATGACAAAGATTTAATGCTCACGACTCAC
TTCAGAACTAGGAATATTACAGATAATCATGGGAAGAATAAGACTACAGTACATGGTTACTTCTCTTCCATTT
TTCTTGGTGGAGTGGATAGTCTAGTTGACTTGATGAACAAGAGCTTTCCTGAGTTGGGTATTAAAAAAACTGA
TTGCAAAGAATTGAGCTGGATTGATACAACCATCTTCTACAGTGGTGTTGTAAATTACAACACTGCTAATTTT
AAAAAGGAAATTTTGCTTGATAGATCAGCTGGGAAGAAGACGGCTTTCTCAATTAAGTTAGACTATGTTAAGA
AACTAATACCTGAAACTGCAATGGTCAAAATTTTGGAAAAATTATATGAAGAAGAGGTAGGAGTTGGGATGTA
TGTGTTGTACCCTTACGGTGGTATAATGGATGAGATTTCAGAATCAGCAATTCCATTCCCTCATCGAGCTGGA
ATAATGTATGAACTTTGGTACGCTGCTACCTGGGAGAAGCAAGAAGATAACGAAAAGCATATAAACTGGGTTC

GAAGTGTTTATAATTTCACAACTCCTTATGTGTCCCAAAATCCAAGATTGGCGTATCTCAATTATAGGGACCT
TGATTTAGGAAAAACTAATCCTGAGAGTCCTAATAATTACACACAAGCACGTATTTGGGGTGAAAAGTATTTT
GGTAAAAATTTTAACAGGTTAGTTAAGGTGAAAACCAAAGCTGATCCCAATAATTTTTTTAGAAACGAACAAA
GTATCCCACCTCTTCCACCGCGTCATCATTAA
>A2_AB212831.1
ATGAATTGCTCAGCATTTTCCTTTTGGTTTGTTTGCAAAATAATATTTTTCTTTCTCTCATTCAATATCCAAA
TTTCAATAGCTAATCCTCAAGAAAACTTCCTTAAATGCTTCTCGGAATATATTCCTAACAATCCAGCAAATCC
AAAATTCATATACACTCAACACGACCAATTGTATATGTCTGTCCTGAATTCAACAATACAAAATCTTAGATTC
ACCTCTGATACAACCCCAAAACCACTCGTTATTGTCACTCCTTCAAATGTCTCCCATATCCAGGCCAGTATTC
TCTGCTCCAAGAAAGTTGGTTTGCAGATTCGAACTCGAAGCGGTGGCCATGATGCTGAGGGTTTGTCCTACAT
ATCTCAAGTCCCATTTGCTATAGTAGACTTGAGAAACATGCATACGGTCAAAGTAGATATTCATAGCCAAACT
GCGTGGGTTGAAGCCGGAGCTACCCTTGGAGAAGTTTATTATTGGATCAATGAGATGAATGAGAATTTTAGTT
TTCCTGGTGGGTATTGCCCTACTGTTGGCGTAGGTGGACACTTTAGTGGAGGAGGCTATGGAGCATTGATGCG
AAATTATGGCCTTGCGGCTGATAATATCATTGATGCACACTTAGTCAATGTTGATGGAAAAGTTCTAGATCGA
AAATCCATGGGAGAAGATCTATTTTGGGCTATACGTGGTGGAGGAGGAGAAAACTTTGGAATCATTGCAGCAT
GGAAAATCAAACTTGTTGTTGTCCCATCAAAGGCTACTATATTCAGTGTTAAAAAGAACATGGAGATACATGG
GCTTGTCAAGTTATTTAACAAATGGCAAAATATTGCTTACAAGTATGACAAAGATTTAATGCTCACGACTCAC
TTCAGAACTAGGAATATTACAGATAATCATGGGAAGAATAAGACTACAGTACATGGTTACTTCTCTTCCATTT
TTCTTGGTGGAGTGGATAGTCTAGTTGACTTGATGAACAAGAGCTTTCCTGAGTTGGGTATTAAAAAAACTGA
TTGCAAAGAATTGAGCTGGATTGATACAACCATCTTCTACAGTGGTGTTGTAAATTACAACACTGCTAATTTT
AAAAAGGAAATTTTGCTTGATAGATCAGCTGGGAAGAAGACGGCTTTCTCAATTAAGTTAGACTATGTTAAGA
AACTAATACCTGAAACTGCAATGGTCAAAATTTTGGAAAAATTATATGAAGAAGAGGTAGGAGTTGGGATGTA
TGTGTTGTACCCTTACGGTGGTATAATGGATGAGATTTCAGAATCAGCAATTCCATTCCCTCATCGAGCTGGA
ATAATGTATGAACTTTGGTACACTGCTACCTGGGAGAAGCAAGAAGATAACGAAAAGCATATAAACTGGGTTC
GAAGTGTTTATAATTTCACAACTCCTTATGTGTCCCAAAATCCAAGATTGGCGTATCTCAATTATAGGGACCT
TGATTTAGGAAAAACTAATCCTGAGAGTCCTAATAATTACACACAAGCACGTATTTGGGGTGAAAAGTATTTT
GGTAAAAATTTTAACAGGTTAGTTAAGGTGAAAACCAAAGCTGATCCCAATAATTTTTTTAGAAACGAACAAA
GTATCCCACCTCTTCCACCGCATCATCAT
>A2_AB212840.1
ATGAATTGCTCAGCATTTTCCTTTTGGTTTGTTTGCAAAATAATATTTTTCTTTCTCTCATTCAATATCCAAA
TTTCAATAGCTAATCCTCAAGAAAACTTCCTTAAATGCTTCTCGGAATATATTCCTAACAATCCAGCAAATCC
AAAATTCATATACACTCAACACGACCAATTGTATATGTCTGTCCTGAATTCGACAATACAAAATCTTAGATTC
ACCTCTGATGCAACCCCAAAACCACTCGTTATTGTCACTCCTTCAAATGTCTCCCATATCCAGGCCAGTATTC
TCTGCTCCAAGAAAGTTGGTTTGCAGATTCGAACTCGAAGCGGTGGCCATGATGCTGAGGGTTTGTCCTACAT
ATCTCAAGTCCCATTTGCTATAGTAGACTTGAGAAACATGCATACGGTCAAAGTAGATATTCATAGCCAAACT
GCGTGGGTTGAAGCCGGAGCTACCCTTGGAGAAGTTTATTATTGGATCAATGAGATGAATGAGAATTTTAGTT
TTCCTGGTGGGTATTGCCCTACTGTTGGCGTAGGTGGACACTTTAGTGGAGGAGGCTATGGAGCATTGATGCG
AAATTATGGCCTTGCGGCTGATAATATCATTGATGCACACTTAGTCAATGTTGATGGAAAAGTTCTAGATCGA
AAATCCATGGGAGAAGATCTATTTTGGGCTATACGTGGTGGAGGAGGAGAAAACTTTGGAATCATTGCAGCAT
GGAAAATCAAACTTGTTGTTGTCCCATCAAAGGCTACTATATTCAGTGTTAAAAAGAACATGGAGATACATGG
GCTTGTCAAGTTATTTAACAAATGGCAAAATATTGCTTACAAGTATGACAAAGATTTAATGCTCACGACTCAC
TTCAGAACTAGGAATATTACAGATAATCATGGGAAGAATAAGACTACAGTACATGGTTACTTCTCTTCCATTT
TTCTTGGTGGAGTGGATAGTCTAGTTGACTTGATGAACAAGAGCTTTCCTGAGTTGGGTATTAAAAAAACTGA
TTGCAAAGAATTGAGCTGGATTGATACAACCATCTTCTACAGTGGTGTTGTAAATTACAACACTGCTAATTTT
AAAAAGGAAATTTTGCTTGATAGATCAGCTGGGAAGAAGACGGCTTTCTCAATTAAGTTAGACTATGTTAAGA
AACTAATACCTGAAACTGCAATGGTCAAAATTTTGGAAAAATTATATGAAGAAGAGGTAGGAGTTGGGATGTA
TGTGTTGTACCCTTACGGTGGTATAATGGATGAGATTTCAGAATCAGCAATTCCATTCCCTCATCGAGCTGGA
ATAATGTATGAACTTTGGTACACTGCTACCTGGGAGAAGCAAGAAGATAACGAAAAGCATATAAACTGGGTTC
GAAGTGTTTATAATTTCACAACTCCTTATGTGTCCCAAAATCCAAGATTGGCGTATCTCAATTATAGGGACCT
TGATTTAGGAAAAACTAATCCTGAGAGTCCTAATAATTACACACAAGCACGTATTTGGGGTGAAAAGTATTTT
GGTAAAAATTTTAACAGGTTAGTTAAGGTGAAAACCAAAGCTGATCCCAATAATTTTTTTAGAAACGAACAAA
GTATCCCACCTCTTCCACCGCATCATCATCAT
>A2_KT876021.1
ATGAATTGCTCAGCATTTTCCTTTTGGTTTGTTTGCAAAATAATATTTTTCTTTCTCTCATTCAATATCCAAA
TTTCAATAGCTAATCCTCAAGAAAACTTCCTTAAATGCTTCTCGGAATATATTCCTAACAATCCAGCAAATCC
AAAATTCATATACACTCAACACGACCAATTGTATATGTCTGTCCTGAATTCGACAATACAAAATCTTAGATTC
ACCTCTGATACAACCCCAAAACCACTCGTTATTGTCACTCCTTCAAATGTCTCCCATATCCAGGCCAGTATTC
TCTGCTCCAAGAAAGTTGGTTTGCAGATTCGAACTCGAAGCGGTGGCCATGATGCTGAGGGTTTGTCCTACAT

ATCTCAAGTCCCATTTGCTATAGTAGACTTGAGAAACATGCATACGGTCAAAGTAGATATTCATAGCCAAACT
GCGTGGGTTGAAGCCGGAGCTACCCTTGGAGAAGTTTATTATTGGATCAATGAGATGAATGAGAATTTTAGTT
TTCCTGGTGGGTATTGCCCTACTGTTGGCGTAGGTGGACACTTTAGTGGAGGAGGCTATGGAGCATTGATGCG
AAATTATGGCCTTGCGGCTGATAATATCATTGATGCACACTTAGTCAATGTTGATGGAAAAGTTCTAGATCGA
AAATCCATGGGAGAAGATCTATTTTGGGCTATACGTGGTGGAGGAGGAGAAAACTTTGGAATCATTGCAGCAT
GGAAAATCAAACTTGTTGTTGTCCCATCAAAGGCTACTATATTCAGTGTTAAAAAGAACATGGAGATACATGG
GCTTGTCAAGTTATTTAACAAATGGCAAAATATTGCTTACAAGTATGACAAAGATTTAATGCTCACGACTCAC
TTCAGAACTAGGAATATTACAGATAATCATGGGAAGAATAAGACTACAGTACATGGTTACTTCTCTTCCATTT
TTCTTGGTGGAGTGGATAGTCTAGTTGACTTGATGAACAAGAGCTTTCCTGAGTTGGGTATTAAAAAAACTGA
TTGCAAAGAATTGAGCTGGATTGATACAACCATCTTCTACAGTGGTGTTGTAAATTACAACACTGCTAATTTT
AAAAAGGAAATTTTGCTTGATAGATCAGTTGGGAAGAAGACGGCTTTCTCAATTAAGTTAGACTATGTTAAGA
AACTAATACCTGAAACTGCAATGGTCAAAATTTTGGAAAAATTATATGAAGAAGAGGTAGGAGTTGGGATGTA
TGTGTTGTACCCTTACGGTGGTATAATGGATGAGATTTCAGAATCAGCAATTCCATTCCCTCATCGAGCTGGA
ATAATGTATGAACTTTGGTACACTGCTACCTGGGAGAAGCAAGAAGATAACGAAAAGCATATAAACTGGGTTC
GAAGTGTTTATAATTTCACAACTCCTTATGTGTCCCAAAATCCAAGATTGGCGTATCTCAATTATAGGGACCT
TGATTTAGGAAAAACTAATCCTGAGAGTCCTAATAATTACACACAAGCACGTATTTGGGGTGAAAAGTATTTT
GGTAAAAATTTTAACAGGTTAGTTAAGGTGAAAACCAAAGCTGATCCCAATAATTTTTTTAGAAACGAACAAA
GTATCCCACCTCTTCCACCGCATCATCAT
>A2_KT876023.1
ATGAATTGCTCAGCATTTTCCTTTTGGTTTGTTTGCAAAATAATATTTTTCTTTCTCTCATTCAATATCCAAA
TTTCAATAGCTAATCCTCAAGAAAACTTCCTTAAATGCTTCTCGGAATATATTCCTAACAATCCAGCAAATCC
AAAATTCATATACACTCAACACGACCAATTGTATATGTCTGTCCTGAATTCGACAATACAAAATCTTAGATTC
ACCTCTGATACAACCCCAAAACCACTCGTTATTGTCACTCCTTCAAATGTCTCCCATATCCAGGCCAGTATTC
TCTGCTCCAAGAAAGTTGGTTTGCAGATTCGAACTCGAAGCGGTGGCCATGATGCTGAGGGTTTGTCCTACAT
ATCTCAAGTCCCATTTGCTATAGTAGACTTGAGAAACATGCATACGGTCAAAGTAGATATTCATAGCCAAACT
GCGTGGGTTGAAGCCGGAGCTACCCTTGGAGAAGTTTATTATTGGATCAATGAGATGAATGAGAATTTTAGTT
TTCCTGGTGGGTATTGCCCTACTGTTGGCGTAGGTGGACACTTTAGTGGAGGAGGCTATGGAGCATTGATGCG
AAATTATGGCCTTGCGGCTGATAATATCATTGATGCACACTTAGTCAATGTTGATGGAAAAGTTCTAGATCGA
AAATCCATGGGAGAAGATCTATTTTGGGCTATACGTGGTGGAGGAGGAGAAAACTTTGGAATCATTGCAGCAT
GGAAAATCAAACTTGTTGTTGTCCCATCAAAGGCTACTATATTCAGTGTTAAAAAGAACATGGAGATACATGG
GCTTGTCAAGTTATTTAACAAATGGCAAAATATTGCTTACAAGTATGACAAAGATTTAATGCTCACGACTCAC
TTCAGAACTAGGAATATTACAGATAATCATGGGAAGAATAAGACTACAGTACATGGTTACTTCTCTTCCATTT
TTCTTGGTGGAGTGGATAGTCTAGTTGACTTGATGAACAAGAGCTTTCCTGAGTTGGGTATTAAAAAAACTGA
TTGCAAAGAATTGAGCTGGATTGATACAACCATCTTCTACAGTGGTGTTGTAAATTACAACACTGCTAATTTT
AAAAAGGAAATTTTGCTTGATAGATCAGCTGGGAAGAAGACGGCTTTCTCAATTAAGTTAGACTATGTTAAGA
AACTAATACCTGAAACTGCAATGGTCAAAATTTTGGAAAAATTATATGAAGAAGAGGTAGGAGTTGGGATGTA
TGTGTTGTACCCTTACGGTGGTATAATGGATGAGATTTCAGAATCAACAATTCCATTCCCTCATCGAGCTGGA
ATAATGTATGAACTTTGGTACACTGCTACCTGGGAGAAGCAAGAAGATAACGAAAAGCATATAAACTGGGTTC
GAAGTGTTTATAATTTCACAACTCCTTATGTGTCCCAAAATCCAAGATTGGCGTATCTCAATTATAGGGACCT
TGATTTAGGAAAAACTAATCCTGAGAGTCCTAATAATTACACACAAGCACGTATTTGGGGTGAAAAGTATTTT
GGTAAAAATTTTAACAGGTTAGTTAAGGTGAAAACCAAAGCTGATCCCAATAATTTTTTTAGAAACGAACAAA
GTATCCCACCTCTTCCACCGCATCATCAT
>A2_KT876024.1
ATGAATTGCTCAGCATTTTCCTTTTGGTTTGTTTGCAAAATAATATTTTTCTTTCTCTCATTCAATATCCAAA
TTTCAATAGCTAATCCTCAAGAAAACTTCCTTAAATGCTTCTCGGAATATATTCCTAACAATCCAGCAAATCC
AAAATTCATATACACTCAACACGACCAATTGTATATGTCTGTCCTGAATTCGACAATACAAAATCTTAGATTC
ACCTCTGATACAACCCCAAAACCACTCGTTATTGTCACTCCTTCAAATGTCTCCCATATCCAGGCCAGTATTC
TCTGCTCCAAGAAAGTTGGTTTGCAGATTCGAACTCGAAGCGGTGGCCATGATGCTGAGGGTTTGTCCTACAT
ATCTCAAGTCCCATTTGCTATAGTAGACTTGAGAAACATGCATACGGTCAAAGTAGATATTCATAGCCAAACT
GCGTGGGTTGAAGCCGGAGCTACCCTTGGAGAAGTTTATTATTGGATCAATGAGATGAATGAGAATTTTAGTT
TTCCTGGTGGGTATTGCCCTACTGTTGGCGTAGGTGGACACTTTAGTGGAGGAGGCTATGGAGCATTGATGCG
AAATTATGGCCTTGCGGCTGATAATATCATTGATGCACACTTAGTCAATGTTGATGGAAAAGTTCTAGATCGA
AAATTCATGGGAGAAGATCTATTTTGGGCTATACGTGGTGGAGGAGGAGAAAACTTTGGAATCATTGCAGCAT
GGAAAATCAAACTTGTTGTTGTCCCATCAAAGGCTACTATATTCAGTGTTAAAAAGAACATGGAGATACATGG
GCTTGTCAAGTTATTTAACAAATGGCAAAATATTGCTTACAAGTATGACAAAGATTTAATGCTCACGACTCAC
TTCAGAACTAGGAATATTACAGATAATCATGGGAAGAATAAGACTACAGTACATGGTTACTTCTCTTCCATTT
TTCTTGGTGGAGTGGATAGTCTAGTTGACTTGATGAACAAGAGCTTTCCTGAGTTGGGTATTAAAAAAACTGA
TTGCAAAGAATTGAGCTGGATTGATACAACCATCTTCTACAGTGGTGTTGTAAATTACAACACTGCTAATTTT

AAAAAGGAAATTTTGCTTGATAGATCAGCTGGGAAGAAGACGGCTTTCTCAATTAAGTTAGACTATGTTAAGA
AACTAATACCTGAAACTGCAATGGTCAAAATTTTGGAAAAATTATATGAAGAAGAGGTAGGAGTTGGGATGTA
TGTGTTGTACCCTTACGGTGGTATAATGGATGAGATTTCAGAATCAGCAATTCCATTCCCTCATCGAGCTGGA
ATAATGTATGAACTTTGGTACACTGCTACCTGGGAGAAGCAAGAAGATAACGAAAAGCATATAAACTGGGTTC
GAAGTGTTTATAATTTCACAACTCCTTATGTGTCCCAAAATCCAAGATTGGCGTATCTCAATTATAGGGACCT
TGATTTAGGAAAAACTAATCCTGAGAGTCCTAATAATTACACACAAGCACGTATTTGGGGTGAAAAGTATTTT
GGTAAAAATTTTAACAGGTTAGTTAAGGTGAAAACCAAAGCTGATCCCAATAATTTTTTTAGAAACGAACAAA
GTATCCCACCTCTTCCACCGCATCATCAT
>A2_KT876028.1
ATGAATTGCTCAGCATTTTCCTTTTGGTTTGTTTGCAAAATAATATTTTTCTTTCTCTCATTCAATATCCAAA
TTTCAATAGCTAATCCTCAAGAAAACTTCCTTAAATGCTTCTCGGAATATATTCCTAACAATCCAGCAAATCC
AAAATTCATATACACTCAACACGACCAATTGTATATGTCTGTCCTGAATTCGACAATACAAAATCTTAGATTC
ACCTCTGATACAACCCCAAAACCACTCGTTATTGTCACTCCTTCAAATGTCTCCCATATCCAGGCCAGTATTC
TCTGCTCCAAGAAAGTTGGTTTGCAGATTCGAACTCGAAGCGGTGGCCATGATGCTGAGGGTTTGTCCTACAT
ATCTCAAGTCCCATTTGCTATAGTAGACTTGAGAAACATGCATACGGTCAAAGTAGATATTCATAGCCAAACT
GCGTGGGTTGAAGCCGGAGCTACCCTTGGAGAAGTTTATTATTGGATCAATGAGATGAATGAGAATTTTAGTT
TTCCTGGTGGGTATTGCCCTACTGTTGGCGTAGGTGGACACTTTAGTGGAGGAGGCTATGGAGCATTGATGCG
AAATTATGGCCTTGCGGCTGATAATATCATTGATGCACACTTAGTCAATGTTGATGGAAAAGTTCTAGATCGA
AAATTCATGGGAGAAGATCTATTTTGGGCTATACGTGGTGGAGGAGGAGAAAACTTTGGAATCATTGCAGCAT
GGAAAATCAAACTTGTTGTTGTCCCATCAAAGGCTACTATATTCAGTGTTAAAAAGAACATGGAGATACATGG
GCTTGTCAAGTTATTTAACAAATGGCAAAATATTGCTTACAAGTATGACAAAGATTTAATGCTCACGACTCAC
TTCAGAACTAGGAATATTACAGATAATCATGGGAAGAATAAGACTACAGTACATGGTTACTTCTCTTCCATTT
TTCTTGGTGGAGTGGATAGTCTAGTTGACTTGATGAACAAGAGCTTTCCTGAGTTGGGTATTAAAAAAACTGA
TTGCAAAGAATTGAGCTGGATTGATACAACCATCTTCTACAGTGGTGTTGTAAATTACAACACTGCTAATTTT
AAAAAGGAAATTTTGCTTGATAGATCAGCTGGGAAGAAGACGGCTTTCTCAATTAAGTTAGACTATGTTAAGA
AACTAATACCTGAAACTGCAATGGTCAAAATTTTGGAAAAATTATATGAAGAAGAGGTAGGAGTTGGGATGTA
TGTGTTGTACCCTTACGGTGGTATAATGGATGAGATTTCAGAATCAGCAATTCCATTCCCTCATCGAGCTGGA
ATAATGTATGAACTTTGGTACACTGCTACCTGGGAGAAGCAAGAAGATAACGAAAAGCATATAAACTGGGTTC
GAAGTGTTTATAATTTCACAACTCCTTATGTGTCCCAAAATCCAAGATTGGCGTATCTCAATTATAGGGACCT
TGATTTAGGAAAAACTAATCCTGAGAGTCCTAATAATTACACACAAGCACGTATTTGGGGTGAAAAGTATTTT
GGTAAAAATTTTAACAGGTTAGTTAAGGTGAAAACCAAAGCTGATCCCAATAATTTTTTTAGAAACGAACAAA
GTATCCCACCTCTTCCACCGCATCATCAT
>A2_AB212839.1
ATGAATTGCTCAGCATTTTCCTTTTGGTTTGTTTGCAAAATAATAATTTTCTTTCTCTCATTCAATATCCAAA
TTTCAATAGCTAATCCTCAAGAAAACTTCCTTAAATGCTTCTCGGAATATATTCCTAACAATCCAGCAAATCC
AAAATTCATATACACTCAACACGACCAATTGTATATGTCTGTCCTGAATTCGACAATACAAAATCTTAGATTC
ACCTCTGATACAACCCCAAAACCACTCGTTATTGTCACTCCTTCAAATGTCTCCCATATCCAGGCCAGTATTC
TCTGCTCCAAGAAAGTTGGTTTGCAGATTCGAACTCGAAGCGGTGGCCATGATGCTGAGGGTTTGTCCTACAT
ATCTCAAGTCCCATTTGCTATAGTAGACTTGAGAAACATGCATACGGTCAAAGTAGATATTCATAGCCAAACT
GCGTGGGTTGAAGCCGGAGCTACCCTTGGAGAAGTTTATTATTGGATCAATGAGATGAATGAGAATTTTAGTT
TTCCTGGTGGGTATTGCCCTACTGTTGGCGTAGGTGGACACTTTAGTGGAGGAGGCTATGGAGCATTGATGCG
AAATTATGGCCTTGCGGCTGATAATATCATTGATGCACACTTAGTCAATGTTGATGGAAAAGTTCTAGATCGA
AAATCCATGGGAGAAGATCTATTTTGGGCTATACGTGGTGGAGGAGGAGAAAACTTTGGAATCATTGCAGCAT
GGAAAATCAAACTTGTTGTTGTCCCATCAAAGGCTACTATATTCAGTGTTAAAAAGAACATGGAGATACATGG
GCTTGTCAAGTTATTTAACAAATGGCAAAATATTGCTTACAAGTATGACAAAGATTTAATGCTCACGACTCAC
TTCAGAACTAGGAATATTACAGATAATCATGGGAAGAATAAGACTACAGTACATGGTTACTTCTCTTCCATTT
TTCTTGGTGGAGTGGATAGTCTAGTTGACTTGATGAACAAGAGCTTTCCTGAGTTGGGTATTAAAAAAACTGA
TTGCAAAGAATTGAGCTGGATTGATACAACCATCTTCTACAGTGGTGTTGTAAATTACAACACTGCTAATTTT
AAAAAGGAAATTTTGCTTGATAGATCAGCTGGGAAGAAGACGGCTTTCTCAATTAAGTTAGACTATGTTAAGA
AACTAATACCTGAAACTGCAATGGTCAAAATTTTGGAAAAATTATATGAAGAAGAGGTAGGAGTTGGGATGTA
TGTGTTGTACCCTTACGGTGGTATAATGGATGAGATTTCAGAATCAGCAATTCCATTCCCTCATCGAGCTGGA
ATAATGTATGAACTTTGGTACACTGCTACCTGGGAGAAGCAAGAAGATAACGAAAAGCATATAAACTGGGTTC
GAAGTGTTTATAATTTCACAACTCCTTATGTGTCCCAAAATCCAAGATTGGCGTATCTCAATTATAGGGACCT
TGATTTAGGAAAAACTAATCCTGAGAGTCCTAATAATTACACACAAGCACGTATTTGGGGTGAAAAGTATTTT
GGTAAAAATTTTAACAGGTTAGTTAAGGTGAAAACCAAAGCTGATCCCAATAATTTTTTTAGAAACGAACAAA
GTATCCCACCTCTTCCACCGCATCATCAT
>A2_AB212841.1

ATGAATTGCTCAGCATTTTCCTTTTGGTTTGTTTGCAAAATAATATTTTTCTTTCTCTCATTCAATATCCAAA
TTTCATTAGCTAATCCTCAAGAAAACTTCCTTAAATGCTTCTCGGAATATATTCCTAACAATCCAGCAAATCC
AAAATTCATATACACTCAACACGACCAATTGTATATGTCTGTCCTGAATTCGACAATACAAAATCTTAGATTC
ACCTCTGATACAACCCCAAAACCACTCGTTATTGTCACTCCTTCAAATGTCTCCCATATCCAGGCCAGTATTC
TCTGCTCCAAGAAAGTTGGTTTGCAGATTCGAACTCGAAGCGGTGGCCATGATGCTGAGGGTTTGTCCTACAT
ATCTCAAGTCCCATTTGCTATAGTAGACTTGAGAAACATGCATACGGTCAAAGTAGATATTCATAGCCAAACT
GCGTGGGTTGAAGCCGGAGCTACCCTTGGAGAAGTTTATTATTGGATCAATGAGATGAATGAGAATTTTAGTT
TTCCTGGTGGGTATTGCCCTACTGTTGGCGTAGGTGGACACTTTAGTGGAGGAGGCTATGGAGCATTGATGCG
AAATTATGGCCTTGCGGCTGATAATATCATTGATGCACACTTAGTCAATGTTGATGGAAAAGTTCTAGATCGA
AAATCCATGGGAGAAGATCTATTTTGGGCTATACGTGGTGGAGGAGGAGAAAACTTTGGAATCATTGCAGCAT
GGAAAATCAAACTTGTTGTTGTCCCATCAAAGGCTACTATATTCAGTGTTAAAAAGAACATGGAGATACATGG
GCTTGTCAAGTTATTTAACAAATGGCAAAATATTGCTTACAAGTATGACAAAGATTTAATGCTCACGACTCAC
TTCAGAACTAGGAATATTACAGATAATCATGGGAAGAATAAGACTACAGTACATGGTTACTTCTCTTCCATTT
TTCTTGGTGGAGTGGATAGTCTAGTTGACTTGATGAACAAGAGCTTTCCTGAGTTGGGTATTAAAAAAACTGA
TTGCAAAGAATTGAGCTGGATTGATACAACCATCTTCTACAGTGGTGTTGTAAATTACAACACTGCTAATTTT
AAAAAGGAAATTTTGCTTGATAGATCAGCTGGGAAGAAGACGGCTTTCTCAATTAAGTTAGACTATGTTAAGA
AACTAATACCTGAAACTGCAATGGTCAAAATTTTGGAAAAATTATATGAAGAAGAGGTAGGAGTTGGGATGTA
TGTGTTGTACCCTTACGGTGGTATAATGGATGAGATTTCAGAATCAGCAATTCCATTCCCTCATCGAGCTGGA
ATAATGTATGAACTTTGGTACACTGCTACCTGGGAGAAGCAAGAAGATAACGAAAAGCATATAAACTGGGTTC
GAAGTGTTTATAATTTCACAACGCCTTATGTGTCCCAAAATCCAAGATTGGCGTATCTCAATTATAGGGACCT
TGATTTAGGAAAAACTAATCCTGAGAGTCCTAATAATTACACACAAGCACGTATTTGGGGTGAAAAGTATTTT
GGTAAAAATTTTAACAGGTTAGTTAAGGTGAAAACCAAAGCTGATCCCAATAATTTTTTTAGAAACGAACAAA
GTATCCCACCTCTTCCACCGCATCATCAT
>A2_KT876019.1
ATGAATTGCTCAGCATTTTCCTTTTGGTTTGTTTGCAAAATAATATTTTTCTTTCTCTCATTCAGTATCCAAA
TTTCAATAGCTAATCCTCAAGAAAACTTCCTTAAATGCTTCTCGGAATATATTCCTAACAATCCAGCAAATCC
AAAATTCATATACACTCAACACGACCAATTGTATATGTCTGTCCTGAATTCGACAATACAAAATCTTAGATTC
ACCTCTGATACAACCCCAAAATCACTCGTTATTGTCACTCCTTCAAATGTCTCCCATATCCAGGCCAGTATTC
TCTGCTCCAAGAAAGTTGGTTTGCAGATTCGAACTCGAAGCGGTGGCCATGATGCTGAGGGTTTGTCCTACAT
ATCTCAAGTCCCATTTGCTATAGTAGACTTGAGAAACATGCATACGGTCAAAGTAGATATTCATAGCCAAACT
GCGTGGGTTGAAGCCGGAGCTACCCTTGGAGAAGTTTATTATTGGATCAATGAGATGAATGAGAATTTTAGTT
TTCCTGGTGGGTATTGCCCTACTGTTGGCGTAGGTGGACACTTTAGTGGAGGAGGCTATGGAGCATTGATGCG
AAATTATGGCCTTGCGGCTGATAATATCATTGATGCACACTTAGTCAATGTTGATGGAAAAGTTCTAGATCGA
AAATCCATGGGAGAAGATCTATTTTGGGCTATACGTGGTGGAGGAGGAGAAAACTTTGGAATCATTGCAGCAT
GGAAAATCAAACTTGTTGTTGTCCCATCAAAGGCTACTATATTCAGTGTTAAAAAGAACATGGAGATACATGG
GCTTGTCAAGTTATTTAACAAATGGCAAAATATTGCTTACAAGTATGACAAAGATTTAATGCTCACGACTCAC
TTCAGAACTAGGAATATTACAGATAATCATGGGAAGAATAAGACTACAGTACATGGTTACTTCTCTTCCATTT
TTCTTGGTGGAGTGGATAGTCTAGTTGACTTGATGAACAAGAGCTTTCCTGAGTTGGGTATTAAAAAAACTGA
TTGCAAAGAATTGAGCTGGATTGATACAACCATCTTCTACAGTGGTGTTGTAAATTACAACACTGCTAATTTT
AAAAAGGAAATTTTGCTTGATAGATCAGCTGGGAAGAAGACGGCTTTCTCAATTAAGTTAGACTATGTTAAGA
AACTAATACCTGAAACTGCAATGGTCAAAATTTTGGAAAAATTATATGAAGAAGAGGTAGGAGTTGGGATGTA
TGTGTTGTACCCTTACGGTGGTATAATGGATGAGATTTCAGAATCAGCAATTCCATTCCCTCATCGAGCTGGA
ATAATGTATGAACTTTGGTACACTGCTACCTGGGAGAAGCAAGAAGATAACGAAAAGCATATAAACTGGGTTC
GAAGTGTTTATAATTTCACAACTCCTTATGTGTCCCAAAATCCAAGATTGGCGTATCTCAATTATAGGGACCT
TGATTTAGGAAAAACTAATCCTGAGAGTCCTAATAATTACACACAAGCACGTATTTGGGGTGAAAAGTATTTT
GGTAAAAATTTTAACAGGTTAGTTAAGGTGAAAACCAAAGCTGATCCCAATAATTTTTTTAGAAACGAACAAA
GTATCCCACCTCTTCCACCGCATCATCAT
>A2_AB212836.1
ATGAATTGCTCAGCATTTTCCTTTTGGTTTGTTTGCAAAATAATATTTTTCTTTCTCTCATTCAATATCCAAA
TTTCATTAGCTAATCCTCAAGAAAACTTCCTTAAATGCTTCTCGGAATATATTCCTAACAATCCAGCAAATCC
AAAATTCATATACACTCAACACGACCAATTGTATATGTCTGTCCTGAATTCGACAATACAAAATCTTAGATTC
ACCTCTGATACAACCCCAAAACCACTCGTTATTGTCACTCCTTCAAATGTCTCCCATATCCAGGCCAGTATTC
TCTGCTCCAAGAAAGTTGGTTTGCAGATTCGAACTCGAAGCGGTGGCCATGATGCTGAGGGTTTGTCCTACAT
ATCTCAAGTCCCATTTGCTATAGTAGACTTGAGAAACATGCATACGGTCAAAGTAGATATTCATAGCCAAACT
GCGTGGGTTGAAGCCGGAGCTACCCTTGGAGAAGTTTATTATTGGATCAATGAGATGAATGAGAATTTTAGTT
TTCCTGGTGGGTATTGCCCTACTGTTGGCGTAGGTGGACACTTTAGTGGAGGAGGCTATGGAGCATTGATGCG
AAATTATGGCCTTGCGGCTGATAATATCATTGATGCACACTTAGTCAATGTTGATGGAAAAGTTCTAGATCGA
AAATCCATGGGAGAAGATCTATTTTGGGCTATACGTGGTGGAGGAGGAGAAAACTTTGGAATCATTGCAGCAT

GGAAAATCAAACTTGTTGTTGTCCCATCAAAGGCTACTATATTCAGTGTTAAAAAGAACATGGAGATACGTGG
GCTTGTCAAGTTATTTAACAAATGGCAAAATATTGCTTACAAGTATGACAAAGATTTAATGCTCACGACTCAC
TTCAGAACTAGGAATATTACAGATAATCATGGGAAGAATAAGACTACAGTACATGGTTACTTCTCTTCCATTT
TTCTTGGTGGAGTGGATAGTCTAGTTGACTTGATGAACAAGAGCTTTCCTGAGTTGGGTATTAAAAAAACTGA
TTGCAAAGAATTGAGCTGGATTGATACAACCATCTTCTACAGTGGTGTTGTAAATTACAACACTGCTAATTTT
AAAAAGGAAATTTTGCTTGATAGATCAGCTGGGAAGAAGACGGCTTTCTCAATTAAGTTAGACTATGTTAAGA
AACTAATACCTGAAACTGCAATGGTCAAAATTTTGGAAAAATTATATGAAGAAGAGGTAGGAGTTGGGATGTA
TGTGTTGTACCCTTACGGTGGTATAATGGATGAGATTTCAGAATCAGCAATTCCATTCCCTCATCGAGCTGGA
ATAATGTATGAACTTTGGTACACTGCTACCTGGGAGAAGCAAGAAGATAACGAAAAGCATATAAACTGGGTTC
GAAGTGTTTACAATTTCACAACTCCTTATGTGTCCCAAAATCCAAGATTGGCGTATCTCAATTATAGGGACCT
TGATTTAGGAAAAACTAATCCTGAGAGTCCTAATAATTACACACAAGCACGTATTTGGGGTGAAAAGTATTTT
GGTAAAAATTTTAACAGGTTAGTTAAGGTGAAAACCAAAGCTGATCCCAATAATTTTTTTAGAAACGAACAAA
GTATCCCACCTCTTCCACCGCATCATCAT
>A2_KT876035.1
ATGAATTGCTCAGCATTTTCCTTTTGGTTTGTTTGCAAAATAATATTTTTCTTTCTCTCATTCAATATCCAAA
TTTCAATAGCTAATCCTCAAGAAAACTTCCTTAAATGCTTCTCGGAATATATTCCTAACAATCCAGCAAATCC
AAAATTCATATACACTCAACACGACCAATTGTATATGTCTGTCATGAATTCGACAATACAAAATCTTAGATTC
ACCTCTGATACAACCCCAAAACCACTCGTTATTGTCACTCCTTCAAATGTCTCCCATATCCAGGCCAGTATTC
TCTGCTCCAAGAAAGTTGGTTTGCAGATTCGAACTCGAAGCGGTGGCCATGATGCTGAGGGTTTGTCCTACAT
ATCTCAAGTCCCATTTGCTATAGTAGACTTGAGAAACATGCATACGATCAAAGTAGATATTCATAGCCAAACT
GCGTGGGTTGAAGCCGGAGCTACCCTTGGAGAAGTTTATTATTGGATCAATGAGATGAATGAGAATTTTAGTT
TTCCTGGTGGGTATTGCCCTACTGTTGGCGTAGGTGGACACTTTAGTGGAGGAGGCTATGGAGCATTGATGCG
AAATTATGGCCTTGCGGCTGATAATATCATTGATGCACACTTAGTCAATGTTGATGGAAAAGTTCTAGATCGA
AAATCCATGGGAGAAGATCTATTTTGGGCTATACGTGGTGGAGGAGGAGAAAACTTTGGAATCATTGCAGCAT
GGAAAATCAAACTTGTTGTTGTCCCATCAAAGGCTACCATATTCAGTGTTAAAAAGAACATGGAGATACATGG
GCTTGTCAAGTTATTTAACAAATGGCAAAATATTGCTTACAAGTATGACAAAGATTTAATGCTCACGACTCAC
TTCAGAACTAGGAATATTACAGATAATCATGGGAAGAATAAGACTACAGTACATGGTTACTTCTCTTCCATTT
TTCTTGGTGGAGTGGATAGTCTAGTTGACTTGATGAACAAGAGCTTTCCTGAGTTGGGTATTAAAAAAACTGA
TTGCAAAGAATTGAGCTGGATTGATACAACCATCTTCTACAGTGGTGTTGTAAATTACAACACTGCTAATTTT
AAAAAGGAAATTTTGCTTGATAGATCAGCTGGGAAGAAGACGGCTTTCTCAATTAAGTTAGACTATGTTAAGA
AACTAATACCTGAAACTGCAATGGTCAAAATTTTGGAAAAATTATATGAAGAAGAGGTAGGAGTTGGGATGTA
TGTGTTGTACCCTTACGGTGGTATAATGGATGAGATTTCAGAATCAGCAATTCCATTCCCTCATCGAGCTGGA
ATAATGTATGAACTTTGGTACACTGCTACCTGGGAGAAGCAAGAAGATAACGAAAAGCATATAAACTGGGTTC
GAAGTGTTTATAATTTCACAACTCCTTATGTGTCCCAAAATCCAAGATTGGCGTATCTCAATTATAGGGACCT
TGATTTAGGAAAAACTAATCCTGAGAGTCCTAATAATTACACACAAGCACGTATTTGGGGTGAAAAGTATTTT
GGTAAAAATTTTAACAGGTTAGTTAAGGTGAAAACCAAAGCTGATCCCAATAATTTTTTTAGAAACGAACAAA
GTATCCCACCTCTTCCACCGCATCATCAT
>A2_AB212833.1
ATGAATTGCTCAGCATTTTCCTTTTGGTTTGTTTGCAAAATAATATTTTTCTTTCTCTCATTCAATATCCAAA
TTTCAATAGCTAATCCTCAAGAAAACTTCCTTAAATGCTTCTCGGAATATATTCCTAACAATCCAGCAAATCC
AAAATTCATATACACTCAACACGACCAATTGTATATGTCTGTCCTGAATTCGACAATACAAAATCTTAGATTC
ACCTCTGATACAACCCCAAAACCACTCGTTATTGTCACTCCTTCAAATGTCTCCCATATCCAGGCCAGTATTC
TCTGCTCCAAGAAAGTTGGTTTGCAGATTCGAACTCGAAGCGGTGGCCATGATGCTGAGGGTTTGTCCTACAT
ATCTCAAGTCCCATTTGCTATAGTAGACTTGAGAAACATGCATACGGTCAAAGTAGATATTCATAGCCAAACT
GCGTGGGTTGAAGCCGGAGCTACCCTTGGAGAAGTTTATTATTGGATCAATGAGATGAATGAGAATTTTAGTT
TTCCTGGTGGGTATTGCCCTACTGTTGGCGTAGGTGGACACTTTAGTGGAGGAGGCTATGGAGCATTGATGCG
AAATTATGGCCTTGCGGCTGATAATATCATTGATTCACACTTAGTCAATGTTGATGGAAAAGTTCTAGATCGA
AAATCCATGGGAGAAGATCTATTTTGGGCTATACGTGGTGGAGGAGGAGAAAACTTTGGAATCATTGCAGCAT
GGAAAATCAAACTTGTTGTTGTCCCATCAAAGGCTACTATATTCAGTGTTAAAAAGAACATGGAGATACATGG
GCTTGTCAAGTTATTTAACAAATGGCAAAATATTGCTTACAAGTATGACAAAGATTTAATGCTCACGACTCAC
TTCAGAACTAGGAATATTACAGATAATCATGGGAAGAATAAGACTACAGTACATGGTTACTTCTCTTCCATTT
TTCTTGGTGGAGTGGATAGTCTAGTTGACTTGATGAACAAGAGCTTTCCTGAGTTGGGTATTAAAAAAACTGA
TTGCAAAGAATTGAGCTGGATTGATACAACCATCTTCTACAGTGGTGTTGTCAATTACAACACTGCTAATTTT
AAAAAGGAAATTTTGCTTGATAGATCAGCTGGGAAGAAGACGGCTTTCTCAATTAAGTTAGACTATGTTAAGA
AACTAATACCTGAAACTGCAATGGTCAAAATTTTGGAAAAATTATATGAAGAAGAGGTAGGAGTTGGGATGTA
TGTGTTGTACCCTTACGGTGGTATAATGGATGAGATTTCAGAATCAGCAATTCCATTCCCTCATCGAGCTGGA
ATAATGTATGAACTTTGGTACACTGCTACCTGGGAGAAGCAAGAAGATAACGAAAAGCATATAAACTGGGTTC
GAAGTGTTTATAATTTCACAACTCCTTATGTGTCCCAAAATCCAAGATTGGCGTATCTCAATTATAGGGACCT

TGATTTAGGAAAAACTAATCCTGAGAGTCCTAATAATTACACACAAGCACGTATTTGGGGTGAAAAGTATTTT
GGTAAAAATTTTAACAGGTTAGTTAAAGTGAAAACCAAAGCTGATCCCAATAATTTTTTTAGAAACGAACAAA
GTATCCCACCTCTTCCACCGCATCATCAT
>A2_KT876036.1
ATGAATTGCTCAGCATTTTCCTTTTGGTTTGTTTGCAAAATAATATTTTTCTTTCTCTCATTCAATATCCAAA
TTTCAATAGCTAATCCTCAAGAAAACTTCCTTAAATGCTTCTCGGAATATATTCCTAACAATCCAGCAAATCC
AAAATTCATATACACTCAACACGACCAATTGTATATGTCTGTCCTGAATTCGACAATACAAAATCTTAGATTC
ACCTCTGATACAACCCCAAAACCACTCGTTATTGTCACTCCTTCAAATGTCTCCCATATCCAGGCCAGTATTC
TCTGCTCCAAGAAAGTTGGTTTGCAGATTCGAACTCGAAGCGGTGGCCATGATGCTGAGGGTTTGTCCTACAT
ATCTCAAGTCCCATTTGCTATAGTAGACTTGAGAAACATGCATACGGTCAAAGTAGATATTCACAGCCAAACT
GCGTGGGTTGAAGCCGGAGCTACCCTTGGAGAAGTTTATTATTGGATCAATGAGATGAATGAGAATTTTAGTT
TTCCTGGTGGGTATTGCCCTACTGTTGGCGTAGGTGGACACTTTAGTGGAGGAGGCTATGGAGCATTGATGCG
AAATTATGGCCTTGCGGCTGATAATATCATTGATGCACACTTAGTCAATGTTGATGGAAAAGTTCTAGATCGA
AAATCCATGGGAGAAGATCTATTTTGGGCTATACGTGGTGGAGGAGGAGAAAACTTTGGAATCATTGCAGCAT
GGAAAATCAAACTTGTTGTTCTCCCATCAAAGGCTACTATATTCAGTGTTAAAAAGAACATGGAGATACATGG
GCTTGTAAAGTTATTTAACAAATGGCAAAATATTGCTTACAAGTATGACAAAGATTTAATGCTCACGACTCAC
TTCAGAACTAGGAATATTACAGATAATCATGGGAAGAATAAGACTACAGTACATGGTTACTTCTCTTCCATTT
TTCTTGGTGGAGTGGATAGTCTAGTTGACTTGATGAACAAGAGCTTTCCTGAGTTGGGTATTAAAAAAACTGA
TTGCAAAGAATTGAGCTGGATTGATACAACCATCTTCTACAGTGGTGTTGTAAATTACAACACTGCTAATTTT
AAAAAGGAAATTTTGCTTGATAGATCAGCTGGGAAGAAGACGGCTTTCTCAATTAAGTTAGACTATGTTAAGA
AACTAATACCTGAAACTGCAATGGTCAAAATTTTGGAAAAATTATATGAAGAAGAGGTAGGAGTTGGGATGTA
TGTGTTGTACCCTTACGGTGGTATAATGGATGAGATTTCAGAATCAGCAATTCCATTCCCTCATCGAGCTGGA
ATAATGTATGAACTTTGGTACACTGCTACCTGGGAGAAGCAAGAAGATAACGAAAAGCATATAAACTGGGTTC
GAAGTGTTTATAATTTCACAACTCCTTATGTGTCCCAAAATCCAAGATTGGCGTATCTCAATTATAGGGACCT
TGATTTAGGAAAAACTAATCCTGAGAGTCCTAATAATTACACACAAGCACGTATTTGGGGTGAAAAGTATTTT
GGTAAAAATTTTAACAGGTTAGTTAAGGTGAAAACCAAAGCTGATCCCAATAATTTTTTTAGAAACGAACAAA
GTATCCCACCTCTTCCACCGCATCATCAT
>A2_MG996406.1
TTTTGGTTTGTTTGCAAAATAATATTTTTCTTTCTCTCATTCAATATCCAAATTTCAATAGCTAATCCTCAAG
AAAACTTCCTTAAATGCTTCTCGGAATATATTCCTAACAATCCAGCAAATCCAAAATTCATATACACTCAACA
CGACCAATTGTATATGTCTGTCCTGAATTCGACAATACAAAATCTTAGATTCACCTCTGATACAACCCCAAAA
CCACTCGTTATTGTCACTCCTTCRAATGTCTCCCATATCCAGGCCAGTATTCTCTGCTCSAAGAAAGTTGGTT
TGCRGATTCGAACTCGAAGCGGTGGCCATGATGCTGAGGGTTTGTCCTACATATCTCAAGTCCCATTTGCTAT
RGTAGACTTGAGAAACATGCATACGGTCAAAGTAGATATTCATAGCCAAACTGCGTGGGTTGAAGCCGGAGCT
ACCCTTGGAGAAGTTTATTATTGGATCAATGAGATGAATGAGAATTTTAGTTTTCCTGGTGGGTATTGCCCTA
CTGTTGGCGTAGGTGGACACTTTAGTGGAGGAGGCTATGGAGCATTGATGCGAAATTRTGGCCTTGCGGCTGA
TAATATCATTGATGCACACTTAGTCAATGTTGATGGAAAAGTTCTAGATCGAAAATCCATGGGAGAAGATCTA
TTTTGGGCTATACGTGGTGGAGGAGGAGAAAACTTTGGAATCATTGCAGCATGTAAAATCAAACTTGTTGTTG
TCCCATCAAAGGCTACTATATTCAGTGTTAAAAAGAACATGGAGATACATGGGCTTGTCAAGTTATTTAACAA
ATGGCAAAATATTGCTTACAAGTATGACAAAGATTTAATGCTCACGACTCACTTCAGAACTAGGAATATTACA
GATAATCATGGGAAGAATAAGACTACAGTACATGGTTACTTCTCTTCCATTTTTCTTGGTGGAGTGGATAGTC
TAGTTGACTTGATGAACAAGAGCTTTCCTGAGTTGGGTATTAAAAAAACTGATTGCAAAGAATTGAGCTGGAT
TGATACAACCATCTTCTACAGTGGTGTTGTAAATTACAACACTGCTAATTTTAAAAAGGAAATTTTGCTTGAT
AGATCAGCTGGGAAGAAGACGGCTTTCTCAATTAAGTTAGACTATGTTAAGAAACTAATACCTGAAACTGCAA
TGGTCAAAATTTTGGAAAAATTATATGAAGAAGAGGTAGGAGTTGGGATGTATGTGTTGTACCCTTACGGTGG
TATAATGGATGAGATTTCAGAATCAGCAATTCCATTCCCTCATCGAGCTGGAATAATGTATGAACTTTGGTAC
ACTGCTACCTGGGAGAAGCAAGAAGATAACGAAAAGCATATAAACTGGGTTCGAAGTGTTTATAATTTCACAA
CTCCTTATGTGTCCCAAAATCCAAGATTGGCGTATCTCAATTATAGGGACCTTGATTTAGGAAAAACTAATCC
TGAGAGTCCTAATAATTACACACAAGCACGTATTTGGGGTGAAAAGTATTTTGGTAAAAATTTTAACAGGTTA
GTTAAGGTGAAAACCAAAGCTGATCCCAATAATTTTTTTAGAAACGAACAAAGTATCCCACCTCTT
>A2_LC120320.1
ATGAATTGCTCAGCATTTTCCTTTTGGTTTGTTTGCAAAATAATATTTTTCTTTCTCTCATTCAATATCCAAA
TTTCAATAGCTAATCCTCAAGAAAACTTCCTTAAATGCTTCTCGGAATATATTCCTAACAATCCAGCAAATCC
AAAATTCATATACACTCAACACGACCAGTTGTATATGTCTGTCCTGAATTCGACAATACAAAATCTTAGATTC
ACCTCTGATACAACCCCAAAACCACTCGTTATTGTCACTCCTTCAAATGTCTCCCATATCCAGGCCAGTATTC
TCTGCTCCAAGAAAGTTGGTTTGCAGATTCGAACTCGAAGCGGTGGCCATGATGCTGAGGGTTTGTCCTACAT
ATCTCAAGTCCCATTTGCTATAGTAGACTTGAGAAACATGCATACGGTCAAAGTAGATATTCATAGCCAAACT
GCGTGGGTTGAAGCCGGAGCTACCCTTGGAGAAGTTTATTATTGGATCAATGAGATGAATGAGAATTTTAGTT

TTCCTGGTGGGTATTGCCCTACTGTTGGCGTAGGTGGACACTTTAGTGGAGGAGGCTATGGAGCATTGATGCG
AAATTATGGCCTTGCGGCTGATAATATCATTGATGCACACTTAGTCAATGTTGATGGAAAAGTTCTAGATCGA
AAATCCATGGGAGAAGATCTATTTTGGGCTATACGTGGTGGAGGAGGAGAAAACTTTGGAATCATTGCAGCAT
GGAAAATCAAACTTGTTGTTGTCCCATCAAAGGCTACTATATTCAGTGTTAAAAAGAACATGGAGATACATGG
GCTTGTCAAGTTATTTAACAAATGGCAAAATATTGCTTACAAGTATGACAAAGATTTAATGCTCACGACTCAC
TTCAGAACTAGGAATATTACAGATAATCATGGGAAGAATAAGACTACAGTACATGGTTACTTCTCTTCCATTT
TTCTTGGTGGAGTGGATAGTCTAGTTGACTTGATGAACAAGAGCTTTCCTGAGTTGGGTATTAAAAAAACTGA
TAGCAAAGAATTGAGCTGGATTGATACAACCATCTTCTACAGTGGTGTTGTAAATTACAACACTGCTAATTTT
AAAAAGGAAATTTTGCTTGATGGATCAGCTGGGAAGAAGACGGCTTTCTCAATTAAGTTAGACTATGTTAAGA
AACTAATACCTGAAACTGCAATGGTCAAAATTTTGGAAAAATTATATGAAGAAGAGGTAGGAGTTGGGATGTA
TGTGTTGTACCCTTACGGTGGTATAATGGATGAGATTTCAGAATCAGCAATTCCATTCCCTCATCGAGTTGGA
ATAATGTATGAACTTTGGTACACTGCTACCTGGGAGAAGCAAGAAGATAACGAAAAGCATATAAACTGGGTTC
GAAGTGTTTATAATTTCACAACTCCTTATGTGTCCCAAAATCCAAGATTGGCGTATCTCAATTATAGGGACCT
TGATTTAGGAAAAACTAATCCTGAGAGTCCTAATAATTACACACAAGCACGTATTTGGGGTGAAAAGTATTTT
GGTAAAAATTTTAACAGGTTAGTTAAGGTGAAAACCAAAGCTGATCCCAATAATTTTTTTAGAAACGAACAAA
GTATCCCACCTCTTCCACCGCATCATCAT
>A2_KT876011.1
ATGAATTGCTCAGCATTTTCCTTTTGGTTTGTTTGCAAAATAATATTTTTCTTTCTCTCATTCAATATCCAAA
TTTCAATAGCTAATCCTCAAGAAAACTTCCTTAAATGCTTCTCGGAATATATTCCTAACAATCCAGCAAATCC
AAAATTCATATACACTCAACACGACCAATTGTATATGTCTGTCCTGAATTCGACAATACAAAATCTTAGATTC
ACCTCTGATACAACCCCAAAACCACTCGTTATTGTCACTCCTTCAAATGTCTCCCATATCTAGGCCAGTATTC
TCTGCTCCAAGAAAGTTGGTTTGCAGATTCGAACTCGAAGCGGTGGCCATGATGCTGAGGGTTTGTCCTACAT
ATCTCAAGTCCCATTTGCTATAGTAGACTTGAGAAACATGCATACGGTCAAAGTAGATATTCATAGCCAAACT
GCGTGGGTTGAAGCCGGAGCTACCCTTGGAGAAGTTTATTATTGGATCAATGAGATGAATGAGAATTTTAGTT
TTCCTGGTGGGTATTGCCCTACTGTTGGCGTAGGTGGACACTTTAGTGGAGGAGGCTATGGAGCATTGATGCG
AAATTATGGCCTTGCGGCTGATAATATCATTGATGCACACTTAGTCAATGTTGATGGAAAAGTTCTAGATCGA
AAATCCATGGGAGAAGATCTATTTTGGGCTATACGTGGTGGAGGAGGAGAAAACTTTGGAATCATTGCAGCAT
GGAAAATCAAACTTGTTGTTGTCCCATCAAAGGCTACTATATTCAGTGTTAAAAAGAACATGGAGATACATGG
GCTTGTCAAGTTATTTAACAAATGGCAAAATATTGCTTACAAGTATGACAAAGATTTAATGCTCACGACTCAC
TTCAGAACTAGGAATATTACAGATAATCATGGGAAGAATAAGACTACAGTACATGGTTACTTCTCTTCCATTT
TTCTTGGTGGAGTGGATAGTCTAGTTGACTTGATGAACAAGAGCTTTCCTGAGTTGGGTATTAAAAAAACTGA
TTGCAAAGAATTGAGCTGGATTGATACAACCATCTTCTACAGTGGTGTTGTAAATTACAACACTGCTAATTTT
AAAAAGGAAATTTTGCTTGATAGATCAGCTGGGAAGAAGACGGCTTTCTCAATTAAGTTAGACTATGTTAAGA
AACTAATACCTGAAACTGCAATGGTCAAAATTTTGGAAAAATTATATGAAGAAGAGGTAGGAGTTGGGATGTA
TGTGTTGTACCCTTACGGTGGTATAATGGATGAGATTTCAGAATCAACAATTCCATTCCTTCATCGAGCTTGA
ATAATGTATGAACTTTGGTACACTGCTACCTGGGAGAAGCAAGAAGATAACGAAAAGCATATAAACTGGGTTC
GAAGTGTTTATAATTTCACAACTCCTTATGTGTCCCAAAATCCAAGATTGGCGTATCTCAATTATAGGGACCT
TGATTTAGGAAAAACTAATCCTGAGAGTCCTAATAATTACACACAAGCACGTATTTGGGGTGAAAAGTATTTT
GGTAAAAATTTTAACAGGTTAGTTAAGGTGAAAACCAAAGCTGATCCCAATAATTTTTTTAGAAATGAACAAA
GTATCCCACCTCTTCCACCGCATCATCAT
>A2_KT876031.1
ATGAATTGCTCAGCATTTTCCTTTTGGTTTGTTTGCAAAATAATATTTTTCTTTCTCTCATTCAATATCCAAA
TTTCAATAGCTAATCCTCAAGAAAACTTCCTTAAATGCTTCTCGGAATATATTCCTAACAATCCAGCAAATCC
AAAATTCATATACACTCAACACGACCAATTGTATATGTCTGTCCTGAATTCGACAATACAAAATCTTAGATTC
ACCTCTGATACAACCCCAAAACCACTCGTTATTGTCACTCCTTCAAATGTCTCCCATATCCAGGCCAGTATTC
TCTGCTCCAAGAAAGTTGGTTTGCAGATTCGAACTCGAAGCGGTGGCCATGATGCTGAGGGTTTGTCCTACAT
ATCTCAAGTCCCATTTGCTATAGTAGACTTGAGAAACATCCATACGGTCAAAGTAGATATTCATAGCCAAACT
GCGTGGGTTGAAGCCGGAGCTACCCTTGGAGAAGTTTATTATTGGATCAATGAGATGAATGAGAATTTTAGTT
TTCCTGGTGGGTATTGCCCTACTGTTGGCGTAGGTGGACACTTTAGTGGAGGAGGCTATGGAGCATTGATGCG
AAATTATGGCCTTGCGGCTGATAATATCATTGATGCACACTTAGTCAATGTTGATGGAAAAGTTCTAGATCGA
AAATCCATGGGAGAAGATCTATTTTGGGCTATACGTGGTGGAGGAGGAGAAAACTTTGGAATCATTGCAGCAT
GGAAAATCAAACTTGTTGTTGTCCCATCAAAGGCTACTATATTCAGTGTTAAAAAGAACATGGAGATACATGG
GCTTGTCAAGTTATTTAACAAATGGCAAAATATTGCTTACAAGTATGACAAAGATTTAATGCTCACGACTCAC
TTCAGAACTAGGAATATTACAGATAATCATGGGAAGAATAAGACTACAGTACATGGTTACTTCTCTTCCATTT
TTCTTGGTGGAGTGGATAGTCTAGTTGACTTGATGAACAAGAGCTTTCCTGAGTTGGGTATTAAAAAAACTGA
TTGCAAAGAATTGAGCTGGATTGATACAACCATCTTCTACAGTGGTGTTGTAAATTACAACACTGCTAATTTT
AAAAAGGAAATTTTGCTTGATAGATCAGCTGGGAAGAAGACGGCTTTCTCAATTAAGTTAGACTATGTTAAGA
AACTAATACCTGAAACTGCAATGGTCAAAATTTTGGAAAAATTATATGAAGAAGAGGTAGGAGTTGGGATGTA

TGTGTTGTACCCTTACGGTGGTATAATGGATGAGATTTCAGAATCAACAATTCCATTCCTTCATCGAGCTTGA
ATAATGTATGAACTTTGGTACACTGCTACCTGGGAGAAGCAAGAAGATAACGAAAAGCATATAAACTGGGTTC
GAAGTGTTTATAATTTCACAACTCCTTATGTGTCCCAAAATCCAAGATTGGCGTATCTCAATTATAGGGACCT
TGATTTAGGAAAAACTAATCCTGAGAGTCCTAATAATTACACACAAGCACGTATTTGGGGTGAAAAGTATTTT
GGTAAAAATTTTAACAGGTTAGTTAAGGTGAAAACCAAAGCTGATCCCAATAATTTTTTTAGAAATGAACAAA
GTATCCCACCTCTTCCACCGCATCATCAT
>A2_KT876030.1
ATGAATTGCTCAGCATTTTCCTTTTGGTTTGTTTGCAAAATAATATTTTTCTTTCTCTCATTCAATATCCAAA
TTTCAATAGCTAATCCTCAAGAAAACTTCCTTAAATGCTTCTCGGAATATATTCCTAACAATCCAGCAAATCC
AAAATTCATATACACTCAACACGACCAATTGTATATGTCTGTCCTGAATTCGACAATACAAAATCTTAGATTC
ACCTCTGATACAACCCCAAAACCACTCGTTATTGTCACTCCTTCAAATTTCTCCCATATCCAGGCCAGTATTC
TCTGCTCCAAGAAAGTTGGTTTGCAGATTCGAACTCGAAGCGGTGGCCATGATGCTGAGGGTTTGTCCTACAT
ATCTCAAGTCCCATTTGCTATAGTAGACTTGAGAAACATGCATACGGTCAAAGTAGATATTCATAGCCAAACT
GCGTGGGTTGAAGCCGGAGCTACCCTTGGAGAAGTTTATTATTGGATCAATGAGATGAATGAGAATTTTAGTT
TTCCTGGTGGGTATTGCCCTACTGTTGGCGTAGGTGGACACTTTAGTGGAGGAGGCTATGGAGCATTGATGCG
AAATTATGGCCTTGCGGCTGATAATATCATTGATGCACACTTAGTCAATGTTGATGGAAAAGTTCTAGATCGA
AAATCCATGGGAGAAGATCTATTTTGGGCTATACGTGGTGGAGGAGGAGAAAACTTTGGAATCATTGCAGCAT
GGAAAATCAAACTTGTTGTTGTCCCATCAAAGGCTACTATATTCAGTGTTAAAAAGAACATGGAGACACATGG
GCTTGTCAAGTTATTTAACAAATGGCAAAATATTGCTTACAAGTATGACAAAGATTTAATGCTCACGACTCAC
TTCAGAACTAGGAATATTACAGATAATCATGGGAAGAATAAGACTACAGTACATGGTTACTTCTCTTCCATTT
TTCTTGGTGGAGTGGATAGTCTAGTTGACTTGATGAACAAGAGCTTTCCTGAGTTGGGTATTAAAAAAACTGA
TTGCAAAGAATTGAGCTGGATTGATACAACCATCTTCTACAGTGGTGTTGTAAATTACAACACTGCTAATTTT
AAAAAGGAAATTTTTCTTGATAGATCAGCTGGGAAGAAGACGGCTTTCTCAATTAAGTTAGACTATGTTAAGA
AACTAATACCTGAAACTGCAATGGTCAAAATTTTGGAAAAATTATATGAAGAAGAGGTAGGAGTTGGGATGTA
TGTGTTGTACCCTTACGATGGTATAATGGATGAGATTTCAGAATCAGCAATTCCATTCCCTCATCGAGCTGGA
ATAATGTATGAACTTTGGTACACTGCTACCTGGGAGAAGCAAGAAGATAACGAAAAGCATATAAACTGGGTTC
GAAGTGTTTATAATTTCACAACTCCTTGTGTGTCCCAAAATCCAAGATTGGCGTATCTCAATTATAGGGACCT
TGATTTAGGAAAATCTAATCCTGAGAGTCCTAATAATTACACACAAGCACGTATTTGGGGTGAAAAGTATTTT
GGTAAAAATTTTAACAGGTTAGTTAAGGTGAAAACCAAAGCTGATCCCAATAATTTTTTTAGAAACGAACAAA
GTATCCCACCTCTTCCACCGCATCATCAT
>A2_KT876025.1
ATGAATTGCTCAGCATTTTCCTTTTGGTTTGTTTGCAAAATAATATTTTTCTTTCTCTCATTCAATATCCAAA
TTTCAATAGCTAATCCTCAAGAAAACTTCCTTAAATGCTTCTCGGAATATATTCCTAACAATCCAGCAAATCC
AAAATTCATATACACTCAACACGACCAATTGTATATGTCTGTCCTGAATTCGACAATACAAAATCTTAGATTC
ACCTTTGATACAACCCCAAAACCACTCGTTATTGTCACTCCTTCAAATGTCTCCCATATCCAGGCCAGTATTC
TCTGCTCCAAGAAAGTTGGTTTGCAGATTCGAACTCGAAGCGGTGGCCATGATGCTGAGGGTTTGTCCTACAT
ATCTCAAGTCCCATTTGCTATAGTAGACTTGAGAAACATGCATACGGTCAAAGTAGATATTCATAGCCAAACT
GCGTGGGTTGAAGCCGGAGCTACCCTTGGAGAAGTTTATTATTGGATCAATGAGATGAATGAGAATTTTAGTT
TTCCTGGTGGGTATTGCCCTACTGTTGGCGTAGGTGGACACTTTAGTGGAGGAGGCTATGGAGCATTGATGCG
AAATTATGGCCTTGCGGCTGATAATATCATTGATGCACACTTAGTCAATGTTGATGGAAAAGTTCTAGATCGA
AAATCCATGGGAGAAGATCTATTTTGGGCTATACGTGGTGGAGGAGGAGAAAACTTTGGAATCATTGCAGCAT
GGAAAATCAAACTTGTTGTTGTCCCATCAAAGGCTACTATATTCAGTGTTAAAAAGAACATGGAGATACATGG
GCTTGTCAAGTTATTTAACAAATGGCAAAATATTGCTTACAAGTATGACAAAGATTTAATGCTCACGACTCAC
TTCAGAAACTAGGAATATTACAGATAATCATGGGAAGAATAAGACTACAGTACATGGTTACTTCTCTTCCATT
TTTCTTGGTGGAGTGGATAGTCTAGTTGACTTGATGAACAAGAGCTTTCCTGAGTTGGGTATTAAAAAAACTG
ATTGCAAAGAATTGAGCTGGATTGATACAACCATCTTCTACAGTGGTGTTGTAAATTACAACACTGCTAATTT
TAAAAAGGAAATTTTGCTTGATAGATCAGCTGGGAAGAAGACGGCTTTCTCAATTAAGTTAGACTATGTTAAG
AAACTAATACCTGAAACTGTAATGGTCAAAATTTTGGAAAAATTATATGAAGAAGAGGTAGGAGTTGGGATGT
ATGTGTTGTACCCTTACGGTGGTATAATGGATGAGATTTCAGAATCAGCAATTCCATTCCCTCATCGAGCTGG
AATAATGTATGAACTTTGGTACACTGCTACCTGGGAGAAGCAAGAAGATAACGAAAAGCATATAAACTGGGTT
CGAAGTGTTTATAATTTCACAACTCCTTATGTGTCCCAAAATCCAAGATTGGCGTATCTCAATTATAGGGACC
TTGATTTAGGAAAAACTAATCCTGAGAGTCCTAATAATTACACACAAGCACGTATTTGGGGTGAAAAGTATTT
TGGTAAAAATTTTAACAGGTTAGTTAAGGTGAAAACCAAAGCTGATCCCAATAATTTTTTTAGAAACGAACAA
AGTATCCCACCTCTTCCACCGCATCATCAT
>A2_KT876029.1
ATGAATTGCTCAGCATTTTCCTTTTGGTTTGTTTGCAAAATAATATTTTTCTTTCTCTCATTCAATATCCAAA
TTTCAATAGCTAATCCTCAAGAAAACTTCCTTAAATGCTTCTCGGAATATATTCCTAACAATCCAGCAAATCC
AAAATTCATATACACTCAACACGACCAATTGTATATGTCTGTCCTGAATTCGACAATACAAAATCTTAGATTC

ACCTTTGATACAACCCCAAAACCACTCGTTATTGTCACTCCTTCAAATGTCTCCCATATCCAGGCCAGTATTC
TCTGCTCCAAGAAAGTTGGTTTGCAGATTCGAACTCGAAGCGGTGGCCATGATGCTGAGGGTTTGTCCTACAT
ATCTCAAGTCCCATTTGCTATAGTAGACTTGAGAAACATGCATACGGTCAAAGTAGATATTCATAGCCAAACT
GCGTGGGTTGAAGCCGGAGCTACCCTTGGAGAAGTTTATTATTGGATCAATGAGATGAATGAGAATTTTAGTT
TTCCTGGTGGGTATTGCCCTACTGTTGGCGTAGGTGGACACTTTAGTGGAGGAGGCTATGGAGCATTGATGCG
AAATTATGGCCTTGCGGCTGATAATATCATTGATGCACACTTAGTCAATGTTGATGGAAAAGTTCTAGATCGA
AAATCCATGGGAGAAGATCTATTTTGGGCTATACGTGGTGGAGGAGGAGAAAACTTTGGAATCATTGCAGCAT
GGAAAATCAAACTTGTTGTTGTCCCATCAAAGGCTACTATATTCAGTGTTAAAAAGAACATGGAGATACATGG
GCTTGTCAAGTTATTTAACAAATGGCAAAATATTGCTTACAAGTATGACAAAGATTTAATGCTCACGACTCAC
TTCAGAAACTAGGAATATTACAGATAATCATGGGAAGAATAAGACTACAGTACATGGTTACTTCTCTTCCATT
TTTCTTGGTGGAGTGGATAGTCTAGTTGACTTGATGAACAAGAGCTTTCCTGAGTTGGGTATTAAAAAAACTG
ATTGCAAAGAATTGAGCTGGATTGATACAACCATCTTCTACAGTGGTGTTGTAAATTACAACACTGCTAATTT
TAAAAAGGAAATTTTGCTTGATAGATCAGCTGGGAAGAAGACGGCTTTCTCAATTAAGTTAGACTATGTTAAG
AAACTAATACCTGAAACTGTAATGGTCAAAATTTTGGAAAAATTATATGAAGAAGAGGTAGGAGTTGGGATGT
ATGTGTTGTACCCTTACGGTGGTATAATGGATGAGATTTCAGAATCAGCAATTCCATTCCCTCATCGAGCTGG
AATAATGTATGAACTTTGGTACACTGCTACCTGGGAGAAGCAAGAAGATAACGAAAAGCATATAAACTGGGTT
CGAAGTGTTTATAATTTCACAACTCCTTATGTGTCCCAAAATCCAAGATTGGCGTATCTCAATTATAGGGACC
TTGATTTAGGAAAAACTAATCCTGAGAGTCCTAATAATTACACACAAGCACGTATTTGGGGTGAAAAGTATTT
TGGTAAAAATTTTAACAGGTTAGTTAAGGTGAAAACCAAAGCTGATCCCAATAATTTTTTTAGAAACGAACAA
AGTATCCCACCTCTTCCACCGCATCATCAT
>A2_Cannatonic_MNPR01002882_12890
ATGAATTGCTCAACATTCTCCTTTTGGTTTGTTTGCAAAATAATATTTTTCTTTCTCTCATTCAATATCCAAA
TTTCAATAGCTAATCCTCAAGAAAACTTCCTTAAATGCTTCTCGGAATATATTCCTAACAATCCAGCAAATCC
AAAATTCATATACACTCAACACGACCAATTGTATATGTCTGTCCTGAATTCGACAATACAAAATCTTAGATTC
ACCTCTGATACAACCCCAAAACCACTCGTTATTGTCACTCCTTCAAATGTCTCCCATATCCAGGCCAGTATTC
TCTGCTCCAAGAAAGTTGGTTTGCAGATTCGAACTCGAAGCGGTGGCCATGATGCTGAGGGTTTGTCCTACAT
ATCTCAAGTCCCATTTGCTATAGTAGACTTGAGAAACATGCATACGGTCAAAGTAGATATTCATAGCCAAACT
GCGTGGGTTGAAGCCGGAGCTACCCTTGGAGAAGTTTATTATTGGATCAATGAGATGAATGAGAATTTTAGTT
TTCCTGGTGGGTATTGCCCTACTGTTGGCGTAGGTGGACACTTTAGTGGAGGAGGCTATGGAGCATTGATGCG
AAATTATGGCCTTGCGGCTGATAATATCATTGATGCACACTTAGTCAATGTTGATGGAAAAGTTCTAGATCGA
AAATCCATGGGAGAAGATCTATTTTGGGCTATACGTGGTGGAGGAGGAGAAAACTTTGGAATCATTGCAGCAT
GGAAAATCAAACTTGTTGTTGTCCCATCAAAGGCTACTATATTCAGTGTTAAAAAGAACATGGAGATACATGG
GCTTGTCAAGTTATTTAACAAATGGCAAAATATTGCTTACAAGTATGACAAAGATTTAATGCTCACGACTCAC
TTCAGAACTAGGAATATTACAGATAATCATGGGAAGAATAAGACTACAGTACATGGTTACTTCTCTTCCATTT
TTCTTGGTGGAGTGGATAGTCTAGTTGACTTGATGAACAAGAGCTTTCCTGAGTTGGGTATTAAAAAAACTGA
TTGCAAAGAATTGAGCTGGATTGATACAACCATCTTCTACAGTGGTGTTGTAAATTACAACACTGCTAATTTT
AAAAAGGAAATTTTGCTTGATAGATCAGCTGGGAAGAAGACGGCTTTCTCAATTAAGTTAGACTATGTTAAGA
AACTAATACCTGAAACTGCAATGGTCAAAATTTTGGAAAAATTATATGAAGAAGAGGTAGGAGTTGGGATGTA
TGTGTTGTACCCTTACGGTGGTATAATGGATGAGATTTCAGAATCAGCAATTCCATTCCCTCATCGAGCTGGA
ATAATGTATGAACTTTGGTACACTGCTACCTGGGAGAAGCAAGAAGATAACGAAAAGCATATAAACTGGGTTC
GAAGTGTTTATAATTTCACAACTCCTTATGTGTCCCAAAATCCAAGATTGGCGTATCTCAATTATAGGGACCT
TGATTTAGGAAAAACTAATCCTGAGAGTCCTAATAATTACACACAAGCACGTATTTGGGGTGAAAAGTATTTT
GGTAAAAATTTTAACAGGTTAGTTAAGGTGAAAACCAAAGCTGATCCCAATAATTTTTTTAGAAACGAACAAA
GTATCCCACCTCTTCCACCGCGTCATCATTAA
>A2_JLion_father_JAATIQ010000121.1_G4B88_001350
ATGAATTGCTCAACATTCTCCTTTTGGTTTGTTTGCAAAATAATATTTTTCTTTCTCTCATTCAATATCCAAA
TTTCAATAGCTAATCCTCAAGAAAACTTCCTTAAATGCTTCTCGGAATATATTCCTAACAATCCAGCAAATCC
AAAATTCATATACACTCAACACGACCAATTGTATATGTCTGTCCTGAATTCGACAATACAAAATCTTAGATTC
ACCTCTGATACAACCCCAAAACCACTCGTTATTGTCACTCCTTCAAATGTCTCCCATATCCAGGCCAGTATTC
TCTGCTCCAAGAAAGTTGGTTTGCAGATTCGAACTCGAAGCGGTGGCCATGATGCTGAGGGTTTGTCCTACAT
ATCTCAAGTCCCATTTGCTATAGTAGACTTGAGAAACATGCATACGGTCAAAGTAGATATTCATAGCCAAACT
GCGTGGGTTGAAGCCGGAGCTACCCTTGGAGAAGTTTATTATTGGATCAATGAGATGAATGAGAATTTTAGTT
TTCCTGGTGGGTATTGCCCTACTGTTGGCGTAGGTGGACACTTTAGTGGAGGAGGCTATGGAGCATTGATGCG
AAATTATGGCCTTGCGGCTGATAATATCATTGATGCACACTTAGTCAATGTTGATGGAAAAGTTCTAGATCGA
AAATCCATGGGAGAAGATCTATTTTGGGCTATACGTGGTGGAGGAGGAGAAAACTTTGGAATCATTGCAGCAT
GGAAAATCAAACTTGTTGTTGTCCCATCAAAGGCTACTATATTCAGTGTTAAAAAGAACATGGAGATACATGG
GCTTGTCAAGTTATTTAACAAATGGCAAAATATTGCTTACAAGTATGACAAAGATTTAATGCTCACGACTCAC
TTCAGAACTAGGAATATTACAGATAATCATGGGAAGAATAAGACTACAGTACATGGTTACTTCTCTTCCATTT

TTCTTGGTGGAGTGGATAGTCTAGTTGACTTGATGAACAAGAGCTTTCCTGAGTTGGGTATTAAAAAAACTGA
TTGCAAAGAATTGAGCTGGATTGATACAACCATCTTCTACAGTGGTGTTGTAAATTACAACACTGCTAATTTT
AAAAAGGAAATTTTGCTTGATAGATCAGCTGGGAAGAAGACGGCTTTCTCAATTAAGTTAGACTATGTTAAGA
AACTAATACCTGAAACTGCAATGGTCAAAATTTTGGAAAAATTATATGAAGAAGAGGTAGGAGTTGGGATGTA
TGTGTTGTACCCTTACGGTGGTATAATGGATGAGATTTCAGAATCAGCAATTCCATTCCCTCATCGAGCTGGA
ATAATGTATGAACTTTGGTACACTGCTACCTGGGAGAAGCAAGAAGATAACGAAAAGCATATAAACTGGGTTC
GAAGTGTTTATAATTTCACAACTCCTTATGTGTCCCAAAATCCAAGATTGGCGTATCTCAATTATAGGGACCT
TGATTTAGGAAAAACTAATCCTGAGAGTCCTAATAATTACACACAAGCACGTATTTGGGGTGAAAAGTATTTT
GGTAAAAATTTTAACAGGTTAGTTAAGGTGAAAACCAAAGCTGATCCCAATAATTTTTTTAGAAACGAACAAA
GTATCCCACCTCTTCCACCGCGTCATCATTAA
>A2_JLion_father_JAATIQ010000121.1_G4B88_001351
ATGAATTGCTCAACATTCTCCTTTTGGTTTGTTTGCAAAATAATATTTTTCTTTCTCTCATTCAATATCCAAA
TTTCAATAGCTAATCCTCAAGAAAACTTCCTTAAATGCTTCTCGGAATATATTCCTAACAATCCAGCAAATCC
AAAATTCATATACACTCAACACGACCAATTGTATATGTCTGTCCTGAATTCGACAATACAAAATCTTAGATTC
ACCTCTGATACAACCCCAAAACCACTCGTTATTGTCACTCCTTCAAATGTCTCCCATATCCAGGCCAGTATTC
TCTGCTCCAAGAAAGTTGGTTTGCAGATTCGAACTCGAAGCGGTGGCCATGATGCTGAGGGTTTGTCCTACAT
ATCTCAAGTCCCATTTGCTATAGTAGACTTGAGAAACATGCATACGGTCAAAGTAGATATTCATAGCCAAACT
GCGTGGGTTGAAGCCGGAGCTACCCTTGGAGAAGTTTATTATTGGATCAATGAGATGAATGAGAATTTTAGTT
TTCCTGGTGGGTATTGCCCTACTGTTGGCGTAGGTGGACACTTTAGTGGAGGAGGCTATGGAGCATTGATGCG
AAATTATGGCCTTGCGGCTGATAATATCATTGATGCACACTTAGTCAATGTTGATGGAAAAGTTCTAGATCGA
AAATCCATGGGAGAAGATCTATTTTGGGCTATACGTGGTGGAGGAGGAGAAAACTTTGGAATCATTGCAGCAT
GGAAAATCAAACTTGTTGTTGTCCCATCAAAGGCTACTATATTCAGTGTTAAAAAGAACATGGAGATACATGG
GCTTGTCAAGTTATTTAACAAATGGCAAAATATTGCTTACAAGTATGACAAAGATTTAATGCTCACGACTCAC
TTCAGAACTAGGAATATTACAGATAATCATGGGAAGAATAAGACTACAGTACATGGTTACTTCTCTTCCATTT
TTCTTGGTGGAGTGGATAGTCTAGTTGACTTGATGAACAAGAGCTTTCCTGAGTTGGGTATTAAAAAAACTGA
TTGCAAAGAATTGAGCTGGATTGATACAACCATCTTCTACAGTGGTGTTGTAAATTACAACACTGCTAATTTT
AAAAAGGAAATTTTGCTTGATAGATCAGCTGGGAAGAAGACGGCTTTCTCAATTAAGTTAGACTATGTTAAGA
AACTAATACCTGAAACTGCAATGGTCAAAATTTTGGAAAAATTATATGAAGAAGAGGTAGGAGTTGGGATGTA
TGTGTTGTACCCTTACGGTGGTATAATGGATGAGATTTCAGAATCAGCAATTCCATTCCCTCATCGAGCTGGA
ATAATGTATGAACTTTGGTACACTGCTACCTGGGAGAAGCAAGAAGATAACGAAAAGCATATAAACTGGGTTC
GAAGTGTTTATAATTTCACAACTCCTTATGTGTCCCAAAATCCAAGATTGGCGTATCTCAATTATAGGGACCT
TGATTTAGGAAAAACTAATCCTGAGAGTCCTAATAATTACACACAAGCACGTATTTGGGGTGAAAAGTATTTT
GGTAAAAATTTTAACAGGTTAGTTAAGGTGAAAACCAAAGCTGATCCCAATAATTTTTTTAGAAACGAACAAA
GTATCCCACCTCTTCCACCGCGTCATCATTAA
>A2_LAconfidential_LKUA01009384.1_3595
ATGAATTGCTCAACATTCTCCTTTTGGTTTGTTTGCAAAATAATATTTTTCTTTCTCTCATTCAATATCCAAA
TTTCAATAGCTAATCCTCAAGAAAACTTCCTTAAATGCTTCTCGGAATATATTCCTAACAATCCAGCAAATCC
AAAATTCATATACACTCAACACGACCAATTGTATATGTCTGTCCTGAATTCGACAATACAAAATCTTAGATTC
ACCTCTGATACAACCCCAAAACCACTCGTTATTGTCACTCCTTCAAATGTCTCCCATATCCAGGCCAGTATTC
TCTGCTCCAAGAAAGTTGGTTTGCAGATTCGAACTCGAAGCGGTGGCCATGATGCTGAGGGTTTGTCCTACAT
ATCTCAAGTCCCATTTGCTATAGTAGACTTGAGAAACATGCATACGGTCAAAGTAGATATTCATAGCCAAACT
GCGTGGGTTGAAGCCGGAGCTACCCTTGGAGAAGTTTATTATTGGATCAATGAGATGAATGAGAATTTTAGTT
TTCCTGGTGGGTATTGCCCTACTGTTGGCGTAGGTGGACACTTTAGTGGAGGAGGCTATGGAGCATTGATGCG
AAATTATGGCCTTGCGGCTGATAATATCATTGATGCACACTTAGTCAATGTTGATGGAAAAGTTCTAGATCGA
AAATCCATGGGAGAAGATCTATTTTGGGCTATACGTGGTGGAGGAGGAGAAAACTTTGGAATCATTGCAGCAT
GGAAAATCAAACTTGTTGTTGTCCCATCAAAGGCTACTATATTCAGTGTTAAAAAGAACATGGAGATACATGG
GCTTGTCAAGTTATTTAACAAATGGCAAAATATTGCTTACAAGTATGACAAAGATTTAATGCTCACGACTCAC
TTCAGAACTAGGAATATTACAGATAATCATGGGAAGAATAAGACTACAGTACATGGTTACTTCTCTTCCATTT
TTCTTGGTGGAGTGGATAGTCTAGTTGACTTGATGAACAAGAGCTTTCCTGAGTTGGGTATTAAAAAAACTGA
TTGCAAAGAATTGAGCTGGATTGATACAACCATCTTCTACAGTGGTGTTGTAAATTACAACACTGCTAATTTT
AAAAAGGAAATTTTGCTTGATAGATCAGCTGGGAAGAAGACGGCTTTCTCAATTAAGTTAG
>A2_Cannatonic_MNPR01009629_3702
ATGAATTGCTCAACATTCTCCTTTTGGTTTGTTTGCAAAATAATATTTTTCTTTCTCTCATTCAATATCCAAA
TTTCAATAGCTAATCCTCAAGAAAACTTCCTTAAATGCTTCTCGGAATATATTCCTAACAATCCAGCAAATCC
AAAATTCATATACACTCAACACGACCAATTGTATATGTCTGTCCTGAATTCGACAATACAAAATCTTAGATTC
ACCTCTGATACAACCCCAAAACCACTCGTTATTGTCACTCCTTCAAATGTCTCCCATATCCAGGCCAGTATTC
TCTGCTCCAAGAAAGTTGGTTTGCAGATTCGAACTCGAAGCGGTGGCCATGATGCTGAGGGTTTGTCCTACAT
ATCTCAAGTCCCATTTGCTATAGTAGACTTGAGAAACATGCATACGGTCAAAGTAGATATTCATAGCCAAACT

GCGTGGGTTGAAGCCGGAGCTACCCTTGGAGAAGTTTATTATTGGATCAATGAGATGAATGAGAATTTTAGTT
TTCCTGGTGGGTATTGCCCTACTGTTGGCGTAGGTGGACACTTTAGTGGAGGAGGCTATGGAGCATTGATGCG
AAATTATGGCCTTGCGGCTGATAATATCATTGATGCACACTTAGTCAATGTTGATGGAAAAGTTCTAGATCGA
AAATCCATGGGAGAAGATCTATTTTGGGCTATACGTGGTGGAGGAGGAGAAAACTTTGGAATCATTGCAGCAT
GGAAAATCAAACTTGTTGTTGTCCCATCAAAGGCTACTATATTCAGTGTTAAAAAGAACATGGAGATACATGG
GCTTGTCAAGTTATTTAACAAATGGCAAAATATTGCTTACAAGTATGACAAAGATTTAATGCTCACGACTCAC
TTCAGAACTAGGAATATTACAGATAATCATGGGAAGAATAAGACTACAGTACATGGTTACTTCTCTTCCATTT
TTCTTGGTGGAGTGGATAGTCTAGTTGACTTGATGAACAAGAGCTTTCCTGAGTTGGGTATTAAAAAAACTGA
TTGCAAAGAATTGAGCTGGATTGATACAACCATCTTCTACAGTGGTGTTGTAAATTACAACACTGCTAATTTT
AAAAAGGAAATTTTGCTTGATAGATCAGCTGGGAAGAAGACGGCTTTCTCAATTAAGTTAGAT
>A2_PBBK_MXBD01006695.1_30203
TGCTCAACATTCTCCTTTTGGTTTGTTTGCAAAATAATATTTTTCTTTCTCTCATTCAATATCCAAATTTCAA
TAGCTAATCCTCAAGAAAACTTCCTTAAATGCTTCTCGGAATATATTCCTAACAATCCAGCAAATCCAAAATT
CATATACACTCAACACGACCAATTGTATATGTCTGTCCTGAATTCGACAATACAAAATCTTAGATTCACCTCT
GATACAACCCCAAAACCACTCGTTATTGTCACTCCTTCAAATGTCTCCCATATCCAGGCCAGTATTCTCTGCT
CCAAGAAAGTTGGTTTGCAGATTCGAACTCGAAGCGGTGGCCATGATGCTGAGGGTTTGTCCTACATATCTCA
AGTCCCATTTGCTATAGTAGACTTGAGAAACATGCATACGGTCAAAGTAGATATTCATAGCCAAACTGCGTGG
GTTGAAGCCGGAGCTACCCTTGGAGAAGTTTATTATTGGATCAATGAGATGAATGAGAATTTTAGTTTTCCTG
GTGGGTATTGCCCTACTGTTGGCGTAGGTGGACACTTTAGTGGAGGAGGCTATGGAGCATTGATGCGAAATTA
TGGCCTTGCGGCTGATAATATCATTGATGCACACTTAGTCAATGTTGATGGAAAAGTTCTAGATCGAAAATCC
ATGGGAGAAGATCTATTTTGGGCTATACGTGGTGGAGGAGGAGAAAACTTTGGAATCATTGCAGCATGGAAAA
TCAAACTTGTTGTTGTCCCATCAAAGGCTACTATATTCAGTGTTAAAAAGAACATGGAGATACATGGGCTTGT
CAAGTTATTTAACAAATGGCAAAATATTGCTTACAAGTATGACAAAGATTTAATGCTCACGACTCACTTCAGA
ACTAGGAATATTACAGATAATCATGGGAAGAATAAGACTACAGTACATGGTTACTTCTCTTCCATTTTTCTTG
GTGGAGTGGATAGTCTAGTTGACTTGATGAACAAGAGCTTTCCTGAGTTGGGTATTAAAAAAACTGATTGCAA
AGAATTGAGCTGGATTGATACAACCATCTTCTACAGTGGTGTTGTAAATTACAACACTGCTAATTTTAAAAAG
GAAATTTTGCTTGATAGATCAGCTGGGAAGAAGACGGCTTTCTCAATTAAGTTAGACTATGTTAAGAAACTAA
TACCTGAAACTGCAATGGTCAAAATTTTGGAAAAATTATATGAAGAAGAGGTAGGAGTTGGGATGTATGTGTT
GTACCCTTACGGTGGTATAATGGATGAGATTTCAGAATCAGCAATTCCATTCCCTCATCGAGCTGGAATAATG
TATGAACTTTGGTACACTGCTACCTGGGAGAAGCAAGAAGATAACGAAAAGCATATAAACTGGGTTCGAAGTG
TTTATAATTTCACAACTCCTTATGTGTCCCAAAATCCAAGATTGGCGTATCTCAATTATAGGGACCTTGATTT
AGGAAAAACTAATCCTGAGAGTCCTAATAATTACACACAAGCACGTATTTGGGGTGAAAAGTATTTTGGTAAA
AATTTTAACAGGTTAGTTAAGGTGAAAACCAAAGCTGATCCCAATAATTTTTTTAGAAACGAACAAAGTATCC
CACCTCTTCCACCGCGTCATCATTAA
>A2_JLion_father_JAATIQ010001028.1_G4B88_000693
ATGAATTGCTCAACATTCTCCTTTTGGTTTGTTTGCAAAATAATATTTTTCTTTCTCTCATTCAATATCCAAA
TTTCAATAGCTAATCCTCAAGAAAACTTCCTTAAATGCTTCTCGGAATATATTCCTAACAATCCAGCAAATCC
AAAATTCATATACACTCAACACGACCAATTGTATATGTCTGTCCTGAATTCGACAATACAAAATCTTAGATTC
ACCTCTGATACAACCCCAAAACCACTCGTTATTGTCACTCCTTCAAATGTCTCCCATATCCAGGCCAGTATTC
TCTGCTCCAAGAAAGTTGGTTTGCAGATTCGAACTCGAAGCGGTGGCCATGATGCTGAGGGTTTGTCCTACAT
ATCTCAAGTCCCATTTGCTATAGTAGACTTGAGAAACATGCATACGGTCAAAGTAGATATTCATAGCCAAACT
GCGTGGGTTGAAGCCGGAGCTACCCTTGGAGAAGTTTATTATTGGATCAATGAGATGAATGAGAATTTTAGTT
TTCCTGGTGGGTATTGCCCTACTGTTGGCGTAGGTGGACACTTTAGTGGAGGAGGCTATGGAGCATTGATGCA
AAATTATGGCCTTGCGGCTGATAATATCATTGATGCACACTTAGTCAATGTTGATGGAAAAGTTCTAGATCGA
AAATCCATGGGAGAAGATCTATTTTGGGCTATACGTGGTGGAGGAGGAGAAAACTTTGGAATCATTGCAGCAT
GGAAAATCAAACTTGTTGTTGTCCCATCAAAGGCTACTATATTCAGTGTTAAAAAGAACATGGAGATACATGG
GCTTGTCAAGTTATTTAACAAATGGCAAAATATTGCTTACAAGTATGACAAAGATTTAATGCTCACGACTCAC
TTCAGAACTAGGAATATTACAGATAATCATGGGAAGAATAAGACTACAGTACATGGTTACTTCTCTTCCATTT
TTCTTGGTGGAGTGGATAGTCTAGTTGACTTGATGAACAAGAGCTTTCCTGAGTTGGGTATTAAAAAAACTGA
TTGCAAAGAATTGAGCTGGATTGATACAACCATCTTCTACAGTGGTGTTGTAAATTACAACACTGCTAATTTT
AAAAAGGAAATTTTGCTTGATAGATCAGCTGGGAAGAAGACGGCTTTCTCAATTAAGTTAGACTATGTTAAGA
AACTAATACCTGAAACTGCAATGGTCAAAATTTTGGAAAAATTATATGAAGAAGAGGTAGGAGTTGGGATGTA
TGTGTTGTACCCTTACGGTGGTATAATGGATGAGATTTCAGAATAAGCAATTCCATTCCCTCATCGAGCTGGA
ATAATGTATGAACTTTGGTACACTGCTACCTGGGAGAAGCAAGAAGATAACGAAAAGCATATAAACTGGGTTC
GAAGTGTTTATAATTTCACAACTCCTTATGTGTCCCAAAATCCAAGATTGGCGTATCTCAATTATAGGGACCT
TGATTTAGGAAAAACTAATCCTGAGAGTCCTAATAATTACACACAAGCACGTATTTGGGGTGAAAAGTATTTT
GGTAAAAATTTTAACAGGTTAGTTAAGGTGAAAACCAAAGCTGATCCCAATAATTTTTTTAGAAACGAACAAA
GTATCCCACCTCTTCCACCGCGTCATCATTAA

>A2_PBBK_MXBD01007385.1_10498
GTTTGCAAAATAATATTTTTCTTTCTCTCATTCAATATCCAAATTTCAATAGCTAATCCTCAAGAAAACTTCC
TTAAATGCTTCTCGGAATATATTCCTAACAATCCAGCAAATCCAAAATTCATATACACTCAACACGACCAATT
GTATATGTCTGTCCTGAATTCGACAATACAAAATCTTAGATTCACCTCTGATACAACCCCAAAACCACTCGTT
ATTGTCACTCCTTCAAATGTCTCCCATATCCAGGCCAGTATTCTCTGCTCCAAGAAAGTTGGTTTGCAGATTC
GAACTCGAAGCGGTGGCCATGATGCTGAGGGTTTGTCCTACATATCTCAAGTCCCATTTGCTATAGTAGACTT
GAGAAACATGCATACGGTCAAAGTAGATATTCATAGCCAAACTGCGTGGGTTGAAGCCGGAGCTACCCTTGGA
GAAGTTTATTATTGGATCAATGAGATGAATGAGAATTTTAGTTTTCCTGGTGGGTATTGCCCTACTGTTGGCG
TAGGTGGACACTTTAGTGGAGGAGGCTATGGAGCATTGATGCGAAATTATGGCCTTGCGGCTGATAATATCAT
TGATGCACACTTAGTCAATGTTGATGGAAAAGTTCTAGATCGAAAATCCATGGGAGAAGATCTATTTTGGGCT
ATACGTGGTGGAGGAGGAGAAAACTTTGTAATCATTGCAGCATGGAAAATCAAACTTGTTGTTGTCCCATCAA
AGGCTACTATATTCAGTGTTAAAAAGAACATGGAGATACATGGGCTTGTCAAGTTATTTAACAAATGGCAAAA
TATTGCTTACAAGTATGACAAAGATTTAATGCTCACGACTCACTTCAGAACTAGGAATATTACAGATAATCAT
GGGAAGAATAAGACTACAGTACATGGTTACTTCTCTTCCATTTTTCTTGGTGGAGTGGATAGTCTAGTTGACT
TGATGAACAAGAGCTTTCCTGAGTTGGGTATTAAAAAAACTGATTGCAAAGAATTGAGCTGGATTGATACAAC
CATCTTCTACAGTGGTGTTGTAAATTACAACACTGCTAATTTTAAAAAGGAAATTTTGCTTGATAGATCAGCT
GGGAAGAAGACGGCTTTCTCAATTAAGTTAGACTATGTTAAGAAACTAATACCTGAAACTGCAATGGTCAAAA
TTTTGGAAAAATTATATGAAGAAGAGGTAGGAGTTGGGATGTATGTGTTGTACCCTTACGGTGGTATAATGGA
TGAGATTTCAGAATCAGCAATTCCATTCCCTCATCGAGCTGGAATAATGTATGAACTTTGGTACACTGCTACC
TGGGAGAAGCAAGAAGATAACGAAAAGCATATAAACTGGGTTCGAAGTGTTTATAATTTCACAACTCCTTATG
TGTCCCAAAATCCAAGATTGGCGTATCTCAATTATAGGGACCTTGATTTAGGAAAAACTAATCCTGAGAGTCC
TAATAATTACACACAAGCACGTATTTGGGGTGAAAAGTATTTTGGTAAAAATTTTAACAGGTTAGTTAAGGTG
AAAACCAAAGCTGATCCCAATAATTTTTTTAGAAACGAACAAAGTATCCCACCTCTTCCACCGCGTCATCATT
AA
>A2_Chemdog91_LKUB01086152.1_655
ATGAATTGCTCAACATTCTCCTTTTGGTTTGTTTGCAAAATAATATTTTTCTTTCTCTCATTCAATATCCAAA
TTTCAATAGCTAATCCTCAAGAAAACTTCCTTAAATGCTTCTCGGAATATATTCCTAACAATCCAGCAAATCC
AAAATTCATATACACTCAACACGACCAATTGTATATGTCTGTCCTGAATTCGACAATACAAAATCTTAGATTC
ACCTCTGATACAACCCCAAAACCACTCGTTATTGTCACTCCTTCAAATGTCTCCCATATCCAGGCCAGTATTC
TCTGCTCCAAGAAAGTTGGTTTGCAGATTCGAACTCGAAGCGGTGGCCATGATGCTGAGGGTTTGTCCTACAT
ATCTCAAGTCCCATTTGCTATAGTAGACTTGAGAAACATCCATACGGTCAAAGTAGATATTCATAGCCAAACT
GCGTGGGTTGAAGCCGGAGCTACCCTTGGAGAAGTTTATTATTGGATCAATGAGATGAATGAGAATTTTAGTT
TTCCTGGTGGGTATTGCCCTACTGTTGGCGTAGGTGGACACTTTAGTGGAGGAGGCTATGGAGCATTGATGCG
AAATTATGGCCTTGCGGCTGATAATATCATTGATGCACACTTAGTCAATGTTGATGGAAAAGTTCTAGATCGA
AAATCCATGGGAGAAGATCTATTTTGGGCTATACGTGGTGGAGGAGGAGAAAACTTTGGAATCATTGCAGCAT
GGAAAATCAAACTTGTTGTTGTCCCATCAAAGGCTACTATATTCAGTGTTAAAAAGAACATGGAGATACATGG
GCTTGTCAAGTTATTTAACAAATGGCAAAATATTGCTTACAAGTATGACAAAGATTTAATGCTCACGACTCAC
TTCAGAACTAGGAATATTACAGATAATCATGGGAAGAATAAGACTACAGTACATGGTTACTTCTCTTCCATTT
TTCTTGGTGGAGTGGATAGTCTAGTTGACTTGATGAACAAGAGCTTTCCTGAGTTGGGTATTAAAAAAACTGA
TTGCAAAGAATTGAGCTGGATTGATACAACCATCTTCTACAGTGGTGTTGTAAATTACAACACTGCTAATTTT
AAAAAGGAAATTTTGCTTGATAGATCAGCTGGGAAGAAGACGGCTTTCTCAATTAAGTTAGACTATGTTAAGA
AACTAATACCTGAAACTGCAATGGTCAAAATTTTGGAAAAATTATATGAAGAAGAGGTAGGAGTTGGGATGTA
TGTGTTGTACCCTTACGGTGGTATAATGGATGAGATTTCAGAAT
>A2_JLion_father_JAATIQ010000121.1_G4B88_001352
ATGAATTGCTCAACATTCTCCTTTTGGTTTGTTTGCAAAATAATATTTTTCTTTCTCTCATTCAATATCCAAA
TTTCAATAGCTAATCCTCAAGAAAACTTCCTTAAATGCTTCTCGGAATATATTCCTAACAATCCAGCAAATCC
AAAATTCATATACACTCAACACGACCAATTGTATATGTCTGTCCTGAATTCGACAATACAAAATCTTAGATTC
ACCTCTGATACAACCCCAAAACCACTCGTTATTGTCACTCCTTCAAATGTCTCCCATATCCAGGCCAGTATTC
TCTGCTCCAAGAAAGTTGGTTTGCAGATTCAAACTCGAAGCGGTGGCCATGATGCTGAGGGTTTGTCCTACAT
ATCTCAAGTCCCATTTGCTATAGCAGACTTGAGAAACATGCATACGGTCAAAGTAGATATTCATAGCCAAACT
GCGTGGGTTGAAGCCGGAGCTACCCTTGGAGAAGTTTATTATTGGATCAATGAGATGAATGAGAATTTTAGTT
TTCCTGGTGGGTATTGCCCTACTGTTGGCGTAGGTGGACACTTTAGTGGAGGAGGCTATGGAGCATTGATGCA
AAATTATGGCCTTGCGGCTGATAATATCATTGATGCACACTTAGTCAATGTTGATGGAAAAGTTCTAGATCGA
AAATCCATGGGAGAAGATCTATTTTGGGCTATACGTGGTGGAGGAGGAGAAAACTTTGGAATCATTGCAGCAT
GGAAAATCAAACTTGTTGTTGTCCCATCAAAGGCTACTATATTCAGTGTTAAAAAGAACATGGAGATACATGG
GCTTGTCAAGTTATTTAACAAATGGCAAAATATTGCTTACAAGTATGACAAAGATTTAATGCTCACGACTCAC
TTCAGAACTAGGAATATTACAGATAATCATGGGAAGAATAAGACTACAGTACATGGTTACTTCTCTTCCATTT
TTCTTGGTGGAGTGGATAGTCTAGTTGACTTGATGAACAAGAGCTTTCCTGAGTTGGGTATTAAAAAAACTGA

TTGCAAAGAATTGAGCTGGATTGATACAACCATCTTCTACAGTGGTGTTGTAAATTACAACACTGCTAATTTT
AAAAAGGAAATTTTGCTTGATAGATCAGCTGGGAAGAAGACGGCTTTCTCAATTAAGTTAGACTATGTTAAGA
AACTAATACCTGAAACTGCAATGGTCAAAATTTTGGAAAAATTATATGAAGAAGAGGTAGGAGTTGGGATGTA
TGTGTTGTACCCTTACGGTGGTATAATGGATGAGATTTCAGAATCAGCAATTCCATTCCCTCATCGAGCTGGA
ATAATGTATGAACTTTGGTACACTGCTACCTGGGAGAAGCAAGAAGATAACGAAAAGCATATAAACTGGGTTC
GAAGTGTTTATAATTTCACAACTCCTTATGTGTCCCAAAATCCAAGATTGGCGTACCTCAATTATAGGGACCT
TGATTTAGGAAAAACTAATCCTGAGAGTCCTAATAATTACACACAAGCACGTATTTGGGGTGAAAAGTATTTT
GGTAAAAATTTTAACAGGTTAGTTAAGGTGAAAACCAAAGCTGATCCCAATAATTTTTTTAGAAACGAACAAA
GTATCCCACCTCTTCCACCGCGTCATCATTAA
>A2_PBBK_MXBD01004643.1_52291
GCTCAACATTCTCCTTTTGGTTTGTTTGCAAAATAATATTTTTCTTTCTCTCATTCAATATCCAAATTTCAAT
AGCTAATCCTCAAGAAAACTTCCTTAAATGCTTCTCGGAATATATTCCTAACAATCCAGCAAATCCAAAATTC
ATATACACTCAACACGACCAATTGTATATGTCTGTCCTGAATTCGACAATACAAAATCTTAGATTCACCTCTG
ATACAACCCCAAAACCACTCGTTATTGTCACTCCTTCAAATGTCTCCCATATCCAGGCCAGTATTCTCTGCTC
CAAGAAAGTTGGTTTGCAGATTCGAACTCGAAGCGGTGGCCATGATGCTGAGGGTTTGTCCTACATATCTCAA
GTCCCATTTGCTATAGTAGACTTGAGAAACATGCATACGGTCAAAGTAGATATTCATAGCCAAACTGCGTGGG
TTGAAGCCGGAGCTACCCTTGGAGAAGTTTATTATTGGATCAATGAGATGAATGAGAATTTTAGTTTTCCTGG
TGGGTATTGCCCTACTGTTGGCGTAGGTGGACACTTTAGTGGAGGAGGCTATGGAGCATTGATGCGAAATTAT
GGCCTTGCGGCTGATAATATCATTGATGCACACTTAGTCAATGTTGATGGAAAAGTTCTAGATCGAAAATCCA
TGGGAGAAGATCTATTTTGGGCTATACGTGGTGGAGGAGGAGAAAACTTTGGAATCATTGCAGCATGGAAAAT
CAAACTTGTTGTTGTCCCATCAAAGGCTACTATATTCAGTGTTAAAAAGAACATGGAGATACATGGGCTTGTC
AAGTTATTTAACAAATGGCAAAATATTGCTTACAAGTATGACAAAGATTTAATGCTCACGACTCACTTCAGAA
CTAGGAATATTACAGATAATCATGGGAAGAATAAGACTACAGTACATGGTTACTTCTCTTCCATTTTTCTTGG
TGGAGTGGATAGTCTAGTTGACTTGATGAACAAGAGCTTTCCTGAGTTGGGTATTAAAAAAACTGATTGCAAA
GAATTGAGCTGGATTGATACAACCATCTTCTACAGTGGTGTTGTAAATTACAACACTGCTAATTTTAAAAAGG
AAATTTTGCTTGATAGATCAGCTGGGAAGAAGACGGCTTTCTCAATTAAGTTAGACTATGTTAAGAAACTAAT
ACCTGAAACTGCAATGGTCAAAATTTTGGAAATTATATGAAGAAGAGGTAGGAGTTGGGATGTATGTGTTGTA
CTACGGTGGTATAATGGATGAGATTTCAGAATCAGCAATTCCATTCCCTCATCGAGCTGGAATAATGTATGAA
CTTTGGTACACTGCTACCTGGGAGAAGCAAGAAGATAACGAAAAGCATATAAACTGGGTTCGAAGTGTTTATA
ATTTCACAACTCCTTATGTGTCCCAAAATCCAAGATTGGCGTATCTCAATTATAGGGACCTTGATTTAGGAAA
AACTAATCCTGAGAGTCCTAATAATTACACACAAGCACGTATTTGGGGTGAAAAGTATTTTGGTAAAAATTTT
AACAGGTTAGTTAAGGTGAAAACCAAAGCTGATCCCAATAATTTTTTTAGAAACGAACAAAGTATCCCACCTC
TTCCACCGCGTCATCATTAA
>A2_CBDRx_NC_044378.1_LOC115696909
ATGAATTGCTCAACATTCTCCTTTTGGTTTGTTTGCAAAATAATATTTTTCTTTCTCTCATTCAATATCCAAA
TTTCAATAGCTAATCCTCAAGAAAACTTCCTTAAATGCTTCTCTTATATTCCTAACAATCCAAGAAATCCAAA
ATTCATATACACTCAACACGACCAATTGTATATGTCTCATTCCGAATTGACAATACAAAATCTTAGATTCACC
TCTGATACAACCCCAAAACCACTGATTGTCACTCCTTCAAATGTCTCCCATATCCAGGCCAGTATTCTCTGCT
CCAAGAAAGTTGGTTTGCAGATTCGAACTCGAAGCGGTGGCCATGATGCTGAGGGTTTGTCCTACATATCTCA
AGTCCCATTTGCTATAGTAGACTTGAGAAACATCCATACGGTCAAAGTAGATATTCATAGCCAAACTGCGTGG
GTTGAAGCCTGAGCTACCCTTGGAGAAGTTTATTATTGGATCAATGAGATGAATGAGAATTTTAGTTTTCCTG
GTGGGTATTGCCCTGGTGGACACTTTAGTGGAGGAGGCTATGGAGCATTGATGCGAAATTATGGCCTTGCGAC
TGATAATATCATTGATGCACACTTAGTCAATGTTGATGGAAAAGTTCTAGATCGAAAATCCATGGGAGAAGAT
CTATTTTGGGCTATACGTGGTGGAGGAGGAGAAAACTTTGGAATCATTGCAGCATGGAAAATCAAACTTGTTG
TTGTCCCATCAAAGGCTACTATATTCAGTGTTAAAAAGAACATGGAGATACATGGGCTTGTCAAGTTATTTAA
CAAATGGCAAAATATTGCTTACAAGTATGACAAAGATTTAATGCTCACGACTCACTTCAGAACTAGGAATATT
ACAGATAATCATGGGAAGAATAAGACTACAGTACATGGTTACTTCTCTTCCATTTTTCTTGGTGGAGTGGATA
GTCTAGTTGACTTGATGAACAAGAGCTTTCCTGAGTTGGGTATTAAAAAAACTGATTGCAAAGAATTGAGCTG
GATTGATACAACCATCTTCTACAGTGGTGTTGTAAATTACAACACTGCTAATTTTAAAAAGGAAATTTTGCTT
GATAGATCAGCTGGGAAGAAGACGGCTTTCTCAATTAAGTTAGATTAG
>A2_Purple_Kush__AGQN03006292.1_8880
ATGAATTGCTCAACATTCTCCTTTTGGTTTGTTTGCAAAATAATATTTTTCTTTCTCTCATTCAATATCCAAA
TTTCAATAGCTAATCCTCAAGAAAACTTCCTTAAATGCTTCTCGGAATATATTCCTAACAATCCAGCAAATCC
AAAATTCATATACACTCAACACGACCAATTGTATATGTCTGTCCTGAATTCGACAATACAAAATCTTAGATTC
ACCTCTGATACAACCCCAAAACCACTCGTTATTGTCACTCCTTCAAATGTCTCCCATATCCAGGCCAGTATTC
TCTGCTCCAAGAAAGTTGGTTTGCAGATTCGAACTCGAAGCGGTGGCCATGATGCTGAGGGTTTGTCCTACAT
ATCTCAAGTCCCATTTGCTATAGTAGACTTGAGAAACATGCATACGGTCAAAGTAGATATTCATAGCCAAACT
GCGTGGGTTGAAGCCGGAGCTACCCTTGGAGAAGTTTATTATTGGATCAATGAGATGAATGAGAATTTTAGTT

TTCCTGGTGGGTATTGCCCTACTGTTGGCGTAGGTGGACACTTTAGTGGAGGAGGCTATGGAGCATTGATGCG
AAATTATGGCCTTGCGGCTGATAATATCATTGATGCACACTTAGTCAATGTTGATGGAAAAGTTCTAGATCGA
AAATCCATGGGAGAAGATCTATTTTGGGCTATACGTGGTGGAGGAGGAGAAAACTTTGGAATCATTGCAGCAT
GGAAAATCAAACTTGTTGTTGTCCCATCAAAGGCTACTATATTCAGTGTTAAAAAGAACATGGAGATACATGG
GCTTGTCAAGTTATTTAACAAATGGCAAAATATTGCTTACAAGTATGACAAAGATTTAATGCTCACGACTCAC
TTCAGAACTAGGAATATTACAGATAATCATGGGAAGAATAAGACTACAGTACATGGTTACTTCTCTTCCATTT
TTCTTGGTGGAGTGGATAGTCTAGTTGACTTGATGAACAAGAGCTTTCCTGAGTTGGGTATTAAAAAAACTGA
TTGCAAAGAATTGAGCTGGATTGATACAACCATCTTCTACAGTGGTGTTGTAAATTACAACACTGCTAATTTT
AAAAAGGAAATTTTGCTTGATAGATCAGCTGGGAAGAAGACGGCTTTCTCAATTAAGTTAGATTAG
>A2_Purple_Kush__AGQN03010731.1_1830
ATGAATTGCTCAACATTCTCCTTTTGGTTTGTTTGCAAAATAATATTTTTCTTTCTCTCATTCAATATCCAAA
TTTCAATAGCTAATCCTCAAGAAAACTTCCTTAAATGCTTCTCGGAATATATTCCTAACAATCCAGCAAATCC
AAAATTCATATACACTCAACACGACCAATTGTATATGTCTGTCCTGAATTCGACAATACAAAATCTTAGATTC
ACCTCTGATACAACCCCAAAACCACTCGTTATTGTCACTCCTTCAAATGTCTCCCATATCCAGGCCAGTATTC
TCTGCTCCAAGAAAGTTGGTTTGCAGATTCGAACTCGAAGCGGTGGCCATGATGCTGAGGGTTTGTCCTACAT
ATCTCAAGTCCCATTTGCTATAGTAGACTTGAGAAACATGCATACGGTCAAAGTAGATATTCATAGCCAAACT
GCGTGGGTTGAAGCCGGAGCTACCCTTGGAGAAGTTTATTATTGGATCAATGAGATGAATGAGAATTTTAGTT
TTCCTGGTGGGTATTGCCCTACTGTTGGCGTAGGTGGACACTTTAGTGGAGGAGGCTATGGAGCATTGATGCG
AAATTATGGCCTTGCGGCTGATAATATCATTGATGCACACTTAGTCAATGTTGATGGAAAAGTTCTAGATCGA
AAATCCATGGGAGAAGATCTATTTTGGGCTATACGTGGTGGAGGAGGAGAAAACTTTGGAATCATTGCAGCAT
GGAAAATCAAACTTGTTGTTGTCCCATCAAAGGCTACTATATTCAGTGTTAAAAAGAACATGGAGATACATGG
GCTTGTCAAGTTATTTAACAAATGGCAAAATATTGCTTACAAGTATGACAAAGATTTAATGCTCACGACTCAC
TTCAGAACTAGGAATATTACAGATAATCATGGGAAGAATAAGACTACAGTACATGGTTACTTCTCTTCCATTT
TTCTTGGTGGAGTGGATAGTCTAGTTGACTTGATGAACAAGAGCTTTCCTGAGTTGGGTATTAAAAAAACTGA
TTGCAAAGAATTGAGCTGGATTGATACAACCATCTTCTACAGTGGTGTTGTAAATTACAACACTGCTAATTTT
AAAAAGGAAATTTTGCTTGATAGATCAGCTGGGAAGAAGACGGCTTTCTCAATTAAGTTAGATTAG
>A2_Purple_Kush__AGQN03006963.1_15918
ATGAATTGCTCAACATTCTCCTTTTGGTTTGTTTGCAAAATAATATTTTTCTTTCTCTCATTCAATATCCAAA
TTTCAATAGCTAATCCTCAAGAAAACTTCCTTAAATGCTTCTCGGAATATATTCCTAACAATCCAGCAAATCC
AAAATTCATATACACTCAACACGACCAATTGTATATGTCTGTCCTGAATTCGACAATACAAAATCTTAGATTC
ACCTCTGATACAACCCCAAAACCACTCGTTATTGTCACTCCTTCAAATGTCTCCCATATCCAGGCCAGTATTC
TCTGCTCCAAGAAAGTTGGTTTGCAGATTCGAACTCGAAGCGGTGGCCATGATGCTGAGGGTTTGTCCTACAT
ATCTCAAGTCCCATTTGCTATAGTAGACTTGAGAAACATGCATACGGTCAAAGTAGATATTCATAGCCAAACT
GCGTGGGTTGAAGCCGGAGCTACCCTTGGAGAAGTTTATTATTGGATCAATGAGATGAATGAGAATTTTAGTT
TTCCTGGTGGGTATTGCCCTACTGTTGGCGTAGGTGGACACTTTAGTGGAGGAGGCTATGGAGCATTGATGCG
AAATTATGGCCTTGCGGCTGATAATATCATTGATGCACACTTAGTCAATGTTGATGGAAAAGTTCTAGATCGA
AAATCCATGGGAGAAGATCTATTTTGGGCTATACGTGGTGGAGGAGGAGAAAACTTTGGAATCATTGCAGCAT
GGAAAATCAAACTTGTTGTTGTCCCATCAAAGGCTACTATATTCAGTGTTAAAAAGAACATGGAGATACATGG
GCTTGTCAAGTTATTTAACAAATGGCAAAATATTGCTTACAAGTATGACAAAGATTTAATGCTCACGACTCAC
TTCAGAACTAGGAATATTACAGATAATCATGGGAAGAATAAGACTACAGTACATGGTTACTTCTCTTCCATTT
TTCTTGGTGGAGTGGATAGTCTAGTTGACTTGATGAACAAGAGCTTTCCTGAGTTGGGTATTAAAAAAACTGA
TTGCAAAGAATTGAGCTGGATTGATACAACCATCTTCTACAGTGGTGTTGTAAATTACAACACTGCTAATTTT
AAAAAGGAAATTTTGCTTGATAGATCAGCTGGGAAGAAGACGGCTTTCTCAATTAAGTTAGACTATGTTAAGA
AACTAATACCTGAAACTGCAATGGTCAAAATTTTGGAAAAAATTATATGAAGAAGAGGTAGGAGTTGGGATGTA
TGTGTTGTACCCTTACGGTGGTATAATGGATGAGATTTCAGAATCAGCAATTCCATTCCCTCATCGAGCTGGA
ATAATGTATGAACTTTGGTACACTGCTACCTGGGAGCAAGAAGATAACGAAAAGCATATAAACTGGCGAAGTG
TTTATAATTTCACAACTCCTTATGTGTCCCAAAATCCAAGATTGGCGTATCTCAATTATAGGGACCTTGATTT
AGGAAAAACTAATCCTGAGAGTCCTAATAATTACACACAAGCACGTATTTGGGGTGAAAAGTATTTTGGTAAA
AATTTTAACAGGTTAGTTAGGGAAACCAAAGCTGATAATAATTTTTTTAGAAACGAACAAAGTATCCCACCTC
TTCCACCGCGTCATCATTAA
>A2_CBDRx_NC_044378.1_LOC115697880
ATGAATTGCTCAACATTCTCCTTTTGGTTTGTTTGCAAAATAATATTTTTCTTTCTCTCATTCAATATCCAAA
TTTCAATAGCTAATCCTCAAGAAAACTTCCTTAAATGCTTCTCGGAATATATTCCTAACAATCCAGCAAATCC
AAAATTCATATACACTCAACACGACCAATTGTATATGTCTGTCCTGAATTCGACAATACAAAATCTTAGATTC
ACCTCTGATACAACCCCAAAACCACTCGTTATTGTCACTCCTTCAAATGTCTCCCATATCCAGGCCAGTATTC
TCTGCTCCAAGAAAGTTGGTTTGCAGATTCGAACTCGAAGCGGTGGCCATGATGCTGAGGGTTTGTCCTACAT
ATCTCAAGTCCCATTTGCTATAGTAGACTTGAGAAACATGCATACGGTCAAAGTAGATATTCATAGCCAAACT
GCGTGGGTTGAAGCCGGAGCTACCCTTGGAGAAGTTTATTATTGGATCAATGAGATGAATGAGAATTTTAGTT

TTCCTGGTGGGTATTGCCCTACTGTTGGCGTAGGTGGACACTTTAGTGGAGGAGGCTATGGAGCATTGATGCG
AAATTATGGCCTTGCGGCTGATAATATCATTGATGCACACTTAGTCAATGTTGATGGAAAAGTTCTAGATCGA
AAATCCATGGGAGAAGATCTATTTTGGGCTATACGTGGTGGAGGAGGAGAAAACTTTGGAATCATTGCAGCAT
GGAAAATCAAACTTGTTGTTGTCCCATCAAAGGCTACTATATTCAGTGTTAAAAAGAACATGGAGATACATGG
GCTTGTCAAGTTATTTAACAAATGGCAAAATATTGCTTACAAGTATGACAAAGATTTAATGCTCACGACTCAC
TTCAGAACTAGGAATATTACAGATAATCATGGGAAGAATAAGACTACAGTACATGGTTACTTCTCTTCCATTT
TTCTTGGTGGAGTGGATAGTCTAGTTGACTTGATGAACAAGAGCTTTCCTGAGTTGGGTATTAAAAAAACTGA
TTGCAAAGAATTGAGCTGGATTGATACAACCATCTTCTACAGTGGTGTTGTAAATTACAACACTGCTAATTTT
AAAAAGGAAATTTTGCTTGATAGATCAGCTGGGAAGAAGACGGCTTTCTCAATTAAGTTAGACTATGTTAAGA
AACTAATACCTGAAACTGCAATGGTCAAAATTTTGGAAAAAATTATATGAAGAAGAGGTAGGAGTTGGGATGTA
TGTGTTGTACCCTTACGATGGTATAATGGATGAGATTTCAGAATCAGCAATTCCATTCCCTCATCGAGCTGGA
ATAATGTATGAACTTTGGTACACTGCTACCTGGGAGAAGCAAGAAGATAACGAAAAGCATATAAACTGGGTTC
GAAGTGTTTATAATTTCACAACTCCTTGTGTGTCCCAAAATCCAAGATTGGCGTATCTCAATTATAGGGACCT
TGATTTAGGAAAATCTAATCCTGAGAGTCCTAATAATTACACACAAGCACGTATTTGGGGTGAAAAGTATTTT
GGTAAAAATTTTAACAGGTTAGTTAAGGTGAAAACCAAAGCTGATCCCAATAATTTTTTAGAAACGAACAAA
GTATCCCACCTCTTCCACCGCGTCATCATTAA
>A2_Finola_QKVJ02001794.1_70796
ATGAATTGCTCAACATTCTCCTTTTGGTTTGTTTGCAAAATAATATTTTTCTTTCTCTCATTCAATATCCAAA
TTTCAATAGCTAATCCTCAAGAAAACTTCCTTAAATGCTTCTCGGAATATATTCCTAACAATCCAGCAAATCC
AAAATTCATATACACTCAACACGACCAATTGTATATGTCTGTCCTGAATTCGACAATACAAAATCTTAGATTC
ACCTCTGATACAACCCCAAAACCACTCGTTATTGTCACTCCTTCAAATGTCTCCCATATCCAGGCCAGTATTC
TCTGCTCCAAGAAAGTTGGTTTGCAGATTCGAACTCGAAGCGGTGGCCATGATGCTGAGGGTTTGTCCTACAT
ATCTCAAGTCCCATTTGCTATAGTAGACTTGAGAAACATGCATACGGTCAAAGTAGATATTCATAGCCAAACT
GCGTGGGTTGAAGCCGGAGCTACCCTTGGAGAAGTTTATTATTGGATCAATGAGATGAATGAGAATTTTAGTT
TTCCTGGTGGGTATTGCCCTACTGTTGGCGTAGGTGGACACTTTAGTGGAGGAGGCTATGGAGCATTGATGCG
AAATTATGGCCTTGCGGCTGATAATATCATTGATGCACACTTAGTCAATGTTGATGGAAAAGTTCTAGATCGA
AAATCCATGGGAGAAGATCTATTTTGGGCTATACGTGGTGGAGGAGGAGAAAACTTTGGAATCATTGCAGCAT
GGAAAATCAAACTTGTTGTTGTCCCATCAAAGGCTACTATATTCAGTGTTAAAAAGAACATGGAGATACATGG
GCTTGTCAAGTTATTTAACAAATGGCAAAATATTGCTTACAAGTATGACAAAGATTTAATGCTCACGACTCAC
TTCAGAACTAGGAATATTACAGATAATCATGGGAAGAATAAGACTACAGTACATGGTTACTTCTCTTCCATTT
TTCTTGGTGGAGTGGATAGTCTAGTTGACTTGATGAACAAGAGCTTTCCTGAGTTGGGTATTAAAAAAACTGA
TTGCAAAGAATTGAGCTGGATTGATACAACCATCTTCTACAGTGGTGTTGTAAATTACAACACTGCTAATTTT
AAAAAGGAAATTTTGCTTGATAGATCAGCTGGGAAGAAGACGGCTTTCTCAATTAAGTTAGACTATGTTAAGA
AACTAATACCTGAAACTGCAATGGTCAAAATTTTGGAAAAAATTATATGAAGAAGAGGTAGGAGTTGGGATGTA
TGTGTTGTACCCTTACGGTGGTATAATGGATGAGATTTCAGAATCAGCAATTCCATTCCCTCATCGAGCTGGA
ATAATGTATGAACTTTGGTACACTGCTACCTGGGAGAAGCAAGAAGATAACGAAAAGCATATAAACTGGGTTC
GAAGTGTTTATAATTTCACAACTCCTTATGTGTCCCAAAATCCAAGATTGGCGTATCTCAATTATAGGGACCT
TGATTTAGGAAAAACTAATCCTGAGAGTCCTAATAATTACACACAAGCACGTATTTGGGGTGAAAAGTATTTT
GGTAAAAATTTTAACAGGTTAGTTAAGGTGAAAACCAAAGCTGATCCCAATAATTTTTTAGAAACGAACAAA
GTATCCCACCTCTTCCACCGCGTCATCATTAA
>A2_Finola_QKVJ02004887.1_15577
ATGAATTGCTCAACATTCTCCTTTTGGTTTGTTTGCAAAATAATATTTTTCTTTCTCTCATTCAATATCCAAA
TTTCAATAGCTAATCCTCAAGAAAACTTCCTTAAATGCTTCTCGGAATATATTCCTAACAATCCAGCAAATCC
AAAATTCATATACACTCAACACGACCAATTGTATATGTCTGTCCTGAATTCGACAATACAAAATCTTAGATTC
ACCTCTGATACAACCCCAAAACCACTCGTTATTGTCACTCCTTCAAATGTCTCCCATATCCAGGCCAGTATTC
TCTGCTCCAAGAAAGTTGGTTTGCAGATTCGAACTCGAAGCGGTGGCCATGATGCTGAGGGTTTGTCCTACAT
ATCTCAAGTCCCATTTGCTATAGTAGACTTGAGAAACATGCATACGGTCAAAGTAGATATTCATAGCCAAACT
GCGTGGGTTGAAGCCGGAGCTACCCTTGGAGAAGTTTATTATTGGATCAATGAGATGAATGAGAATTTTAGTT
TTCCTGGTGGGTATTGCCCTACTGTTGGCGTAGGTGGACACTTTAGTGGAGGAGGCTATGGAGCATTGATGCG
AAATTATGGCCTTGCGGCTGATAATATCATTGATGCACACTTAGTCAATGTTGATGGAAAAGTTCTAGATCGA
AAATCCATGGGAGAAGATCTATTTTGGGCTATACGTGGTGGAGGAGGAGAAAACTTTGGAATCATTGCAGCAT
GGAAAATCAAACTTGTTGTTGTCCCATCAAAGGCTACTATATTCAGTGTTAAAAAGAACATGGAGATACATGG
GCTTGTCAAGTTATTTAACAAATGGCAAAATATTGCTTACAAGTATGACAAAGATTTAATGCTCACGACTCAC
TTCAGAACTAGGAATATTACAGATAATCATGGGAAGAATAAGACTACAGTACATGGTTACTTCTCTTCCATTT
TTCTTGGTGGAGTGGATAGTCTAGTTGACTTGATGAACAAGAGCTTTCCTGAGTTGGGTATTAAAAAAACTGA
TTGCAAAGAATTGAGCTGGATTGATACAACCATCTTCTACAGTGGTGTTGTAAATTACAACACTGCTAATTTT
AAAAAGGAAATTTTGCTTGATAGATCAGCTGGGAAGAAGACGGCTTTCTCAATTAAGTTAGACTATGTTAAGA
AACTAATACCTGAAACTGCAATGGTCAAAATTTTGGAAAAAATTATATGAAGAAGAGGTAGGAGTTGGGATGTA

TGTGTTGTACCCTTACGGTGGTATAATGGATGAGATTTCAGAATCAGCAATTCCATTCCCTCATCGAGCTGGA
ATAATGTATGAACTTTGGTACACTGCTACCTGGGAGAAGCAAGAAGATAACGAAAAGCATATAAACTGGGTTC
GAAGTGTTTATAATTTCACAACTCCTTATGTGTCCCAAAATCCAAGATTGGCGTATCTCAATTATAGGGACCT
TGATTTAGGAAAAACTAATCCTGAGAGTCCTAATAATTACACACAAGCACGTATTTGGGGTGAAAAGTATTTT
GGTAAAAATTTTAACAGGTTAGTTAAGGTGAAAACCAAAGCTGATCCCAATAATTTTTTTAGAAACGAACAAA
GTATCCCACCTCTTCCACCGCGTCATCATTAA
>A2_Jamaican_Lion_JAATIP010000055.1_F8388_016312
ATGAATTGCTCAACATTCTCCTTTTGGTTTGTTTGCAAAATAATATTTTTCTTTCTCTCATTCAATATCCAAA
TTTCAATAGCTAATCCTCAAGAAAACTTCCTTAAATGCTTCTCGGAATATATTCCTAACAATCCAGCAAATCC
AAAATTCATATACACTCAACACGACCAATTGTATATGTCTGTCCTGAATTCGACAATACAAAATCTTAGATTC
ACCTCTGATACAACCCCAAAACCACTCGTTATTGTCACTCCTTCAAATGTCTCCCATATCCAGGCCAGTATTC
TCTGCTCCAAGAAAGTTGGTTTGCAGATTCGAACTCGAAGCGGTGGCCATGATGCTGAGGGTTTGTCCTACAT
ATCTCAAGTCCCATTTGCTATAGTAGACTTGAGAAACATGCATACGGTCAAAGTAGATATTCATAGCCAAACT
GCGTGGGTTGAAGCCGGAGCTACCCTTGGAGAAGTTTATTATTGGATCAATGAGATGAATGAGAATTTTAGTT
TTCCTGGTGGGTATTGCCCTACTGTTGGCGTAGGTGGACACTTTAGTGGAGGAGGCTATGGAGCATTGATGCG
AAATTATGGCCTTGCGGCTGATAATATCATTGATGCACACTTAGTCAATGTTGATGGAAAAGTTCTAGATCGA
AAATCCATGGGAGAAGATCTATTTTGGGCTATACGTGGTGGAGGAGGAGAAAACTTTGGAATCATTGCAGCAT
GGAAAATCAAACTTGTTGTTGTCCCATCAAAGGCTACTATATTCAGTGTTAAAAAGAACATGGAGATACATGG
GCTTGTCAAGTTATTTAACAAATGGCAAAATATTGCTTACAAGTATGACAAAGATTTAATGCTCACGACTCAC
TTCAGAACTAGGAATATTACAGATAATCATGGGAAGAATAAGACTACAGTACATGGTTACTTCTCTTCCATTT
TTCTTGGTGGAGTGGATAGTCTAGTTGACTTGATGAACAAGAGCTTTCCTGAGTTGGGTATTAAAAAAACTGA
TTGCAAAGAATTGAGCTGGATTGATACAACCATCTTCTACAGTGGTGTTGTAAATTACAACACTGCTAATTTT
AAAAAGGAAATTTTGCTTGATAGATCAGCTGGGAAGAAGACGGCTTTCTCAATTAAGTTAGACTATGTTAAGA
AACTAATACCTGAAACTGCAATGGTCAAAATTTTGGAAAAATTATATGAAGAAGAGGTAGGAGTTGGGATGTA
TGTGTTGTACCCTTACGGTGGTATAATGGATGAGATTTCAGAATCAGCAATTCCATTCCCTCATCGAGCTGGA
ATAATGTATGAACTTTGGTACACTGCTACCTGGGAGAAGCAAGAAGATAACGAAAAGCATATAAACTGGGTTC
GAAGTGTTTATAATTTCACAACTCCTTATGTGTCCCAAAATCCAAGATTGGCGTATCTCAATTATAGGGACCT
TGATTTAGGAAAAACTAATCCTGAGAGTCCTAATAATTACACACAAGCACGTATTTGGGGTGAAAAGTATTTT
GGTAAAAATTTTAACAGGTTAGTTAAGGTGAAAACCAAAGCTGATCCCAATAATTTTTTTAGAAACGAACAAA
GTATCCCACCTCTTCCACCGCGTCATCATTAA
>A2_Purple_Kush__AGQN03005496.1_4620
ATGAATTGCTCAACATTCTCCTTTTGGTTTGTTTGCAAAATAATATTTTTCTTTCTCTCATTCAATATCCAAA
TTTCAATAGCTAATCCTCAAGAAAACTTCCTTAAATGCTTCTCGGAATATATTCCTAACAATCCAGCAAATCC
AAAATTCATATACACTCAACACGACCAATTGTATATGTCTGTCCTGAATTCGACAATACAAAATCTTAGATTC
ACCTCTGATACAACCCCAAAACCACTCGTTATTGTCACTCCTTCAAATGTCTCCCATATCCAGGCCAGTATTC
TCTGCTCCAAGAAAGTTGGTTTGCAGATTCGAACTCGAAGCGGTGGCCATGATGCTGAGGGTTTGTCCTACAT
ATCTCAAGTCCCATTTGCTATAGTAGACTTGAGAAACATGCATACGGTCAAAGTAGATATTCATAGCCAAACT
GCGTGGGTTGAAGCCGGAGCTACCCTTGGAGAAGTTTATTATTGGATCAATGAGATGAATGAGAATTTTAGTT
TTCCTGGTGGGTATTGCCCTACTGTTGGCGTAGGTGGACACTTTAGTGGAGGAGGCTATGGAGCATTGATGCG
AAATTATGGCCTTGCGGCTGATAATATCATTGATGCACACTTAGTCAATGTTGATGGAAAAGTTCTAGATCGA
AAATCCATGGGAGAAGATCTATTTTGGGCTATACGTGGTGGAGGAGGAGAAAACTTTGGAATCATTGCAGCAT
GGAAAATCAAACTTGTTGTTGTCCCATCAAAGGCTACTATATTCAGTGTTAAAAAGAACATGGAGATACATGG
GCTTGTCAAGTTATTTAACAAATGGCAAAATATTGCTTACAAGTATGACAAAGATTTAATGCTCACGACTCAC
TTCAGAACTAGGAATATTACAGATAATCATGGGAAGAATAAGACTACAGTACATGGTTACTTCTCTTCCATTT
TTCTTGGTGGAGTGGATAGTCTAGTTGACTTGATGAACAAGAGCTTTCCTGAGTTGGGTATTAAAAAAACTGA
TTGCAAAGAATTGAGCTGGATTGATACAACCATCTTCTACAGTGGTGTTGTAAATTACAACACTGCTAATTTT
AAAAAGGAAATTTTGCTTGATAGATCAGCTGGGAAGAAGACGGCTTTCTCAATTAAGTTAGACTATGTTAAGA
AACTAATACCTGAAACTGCAATGGTCAAAATTTTGGAAAAATTATATGAAGAAGAGGTAGGAGTTGGGATGTA
TGTGTTGTACCCTTACGGTGGTATAATGGATGAGATTTCAGAATCAGCAATTCCATTCCCTCATCGAGCTGGA
ATAATGTATGAACTTTGGTACACTGCTACCTGGGAGAAGCAAGAAGATAACGAAAAGCATATAAACTGGGTTC
GAAGTGTTTATAATTTCACAACTCCTTATGTGTCCCAAAATCCAAGATTGGCGTATCTCAATTATAGGGACCT
TGATTTAGGAAAAACTAATCCTGAGAGTCCTAATAATTACACACAAGCACGTATTTGGGGTGAAAAGTATTTT
GGTAAAAATTTTAACAGGTTAGTTAAGGTGAAAACCAAAGCTGATCCCAATAATTTTTTTAGAAACGAACAAA
GTATCCCACCTCTTCCACCGCGTCATCATTAA
>A2_Jamaican_Lion_JAATIP010000055.1_F8388_016310
ATGAATTGCTCAACATTCTCCTTTTGGTTTGTTTGCAAAATAATATTTTTCTTTCTCTCATTCAATATCCAAA
TTTCAATAGCTAATCCTCAAGAAAACTTCCTTAAATGCTTCTCGGAATATATTCCTAACAATCCAGCAAATCC
AAAATTCATATACACTCAACACGACCAATTGTATATGTCTGTCCTGAATTCGACAATACAAAATCTTAGATTC

ACCTCTGATACAACCCCAAAACCACTCGTTATTGTCACTCCTTCAAATGTCTCCCATATCCAGGCCAGTATTC
TCTGCTCCAAGAAAGTTGGTTTGCAGATTCGAACTCGAAGCGGTGGCCATGATGCTGAGGGTTTGTCCTACAT
ATCTCAAGTCCCATTTGCTATAGTAGACTTGAGAAACATGCATACGGTCAAAGTAGATATTCATAGCCAAACT
GCGTGGGTTGAAGCCGGAGCTACCCTTGGAGAAGTTTATTATTGGATCAATGAGATGAATGAGAATTTTAGTT
TTCCTGGTGGGTATTGCCCTACTGTTGGCGTAGGTGGACACTTTAGTGGAGGAGGCTATGGAGCATTGATGCG
AAATTATGGCCTTGCGGCTGATAATATCATTGATGCACACTTAGTCAATGTTGATGGAAAAGTTCTAGATCGA
AAATCCATGGGAGAAGATCTATTTTGGGCTATACGTGGTGGAGGAGGAGAAAACTTTGGAATCATTGCAGCAT
GGAAAATCAAACTTGTTGTTGTCCCATCAAAGGCTACTATATTCAGTGTTAAAAAGAACATGGAGATACATGG
GCTTGTCAAGTTATTTAACAAATGGCAAAATATTGCTTACAAGTATGACAAAGATTTAATGCTCACGACTCAC
TTCAGAACTAGGAATATTACAGATAATCATGGGAAGAATAAGACTACAGTACATGGTTACTTCTCTTCCATTT
TTCTTGGTGGAGTGGATAGTCTAGTTGACTTGATGAACAAGAGCTTTCCTGAGTTGGGTATTAAAAAAACTGA
TTGCAAAGAATTGAGCTGGATTGATACAACCATCTTCTACAGTGGTGTTGTCAATTACAACACTGCTAATTTT
AAAAAGGAAATTTTGCTTGATAGATCAGCTGGGAAGAAGACGGCTTTCTCAATTAAGTTAGACTATGTTAAGA
AACTAATACCTGAAACTGCAATGGTCAAAATTTTGGAAAAATTATATGAAGAAGAGGTAGGAGTTGGGATGTA
TGTGTTGTACCCTTACGGTGGTATAATGGATGAGATTTCAGAATCAGCAATTCCATTCCCTCATCGAGCTGGA
ATAATGTATGAACTTTGGTACACTGCTACCTGGGAGAAGCAAGAAGATAACGAAAAGCATATAAACTGGGTTC
GAAGTGTTTATAATTTCACAACTCCTTATGTGTCCCAAAATCCAAGATTGGCGTATCTCAATTATAGGGACCT
TGATTTAGGAAAAACTAATCCTGAGAGTCCTAATAATTACACACAAGCACGTATTTGGGGTGAAAAGTATTTT
GGTAAAAATTTTAACAGGTTAGTTAAAGTGAAAACCAAAGCTGATCCCAATAATTTTTTTAGAAACGAACAAA
GTATCCCACCTCTTCCACCGCGTCATCATTAA
>A2_Jamaican_Lion_JAATIP010000055.1_F8388_016311
ATGAATTGCTCAACATTCTCCTTTTGGTTTGTTTGCAAAATAATATTTTTCTTTCTCTCATTCAATATCCAAA
TTTCAATAGCTAATCCTCAAGAAAACTTCCTTAAATGCTTCTCGGAATATATTCCTAACAATCCAGCAAATCC
AAAATTCATATACACTCAACACGACCAATTGTATATGTCTGTCCTGAATTCGACAATACAAAATCTTAGATTC
ACCTCTGATACAACCCCAAAACCACTCGTTATTGTCACTCCTTCAAATGTCTCCCATATCCAGGTCAGTATTC
TCTGCTCCAAGAAAGTTGGTTTGCAGATTCGAACTCGAAGCGGTGGCCATGATGCTGAGGGTTTGTCCTACAT
ATCTCAAGTCCCATTTGCTATAGTAGACTTGAGAAACATGCATACGGTCAAAGTAGATATTCATAGCCAAACT
GCGTGGGTTGAAGCCGGAGCTACCCTTGGAGAAGTTTATTATTGGATCAATGAGATGAATGAGAATTTTAGTT
TTCCTGGTGGGTATTGCCCTACTGTTGGCGTAGGTGGACACTTTAGTGGAGGAGGCTATGGAGCATTGATGCG
AAATTATGGCCTTGCGGCTGATAATATCATTGATGCACACTTAGTCAATGTTGATGGAAAAGTTCTAGATCGA
AAATCCATGGGAGAAGATCTATTTTGGGCTATACGTGGTGGAGGAGGAGAAAACTTTGGAATCATTGCAGCAT
GGAAAATCAAACTTGTTGTTGTCCCATCAAAGGCTACTATATTCAGTGTTAAAAAGAACATGGAGATACATGG
GCTTGTCAAGTTATTTAACAAATGGCAAAATATTGCTTACAAGTATGACAAAGATTTAATGCTCACGACTCAC
TTCAGAACTAGGAATATTACAGATAATCATGGGAAGAATAAGACTACAGTACATGGTTACTTCTCTTCCATTT
TTCTTGGTGGAGTGGATAGTCTAGTTCACTTGATGAACAAGAGCTTTCCTGAGTTGGGTATTAAAAAAACTGA
TTGCAAAGAATTGAGCTGGATTGATACAACCATCTTCTACAGTGGTGTTGTAAATTACAACACTGCTAATTTT
AAAAAGGAAATTTTGCTTGATAGATCAGCTGGGAAGAAGACGGCTTTCTCAATTAAGTTAGACTATGTTAAGA
AACTAATACCTGAAACTGCAATGGTCAAAATTTTGGAAAAATTATATGAAGAAGAGGTAGGAGTTGGGATGTA
TGTGTTGTACCCTTACGGTGGTATAATGGATGAGATTTCAGAATCAGCAATTCCATTCCCTCATCGAGCTGGA
ATAATGTATGAACTTTGGTACACTGCTACCTGGGAGAAGCAAGAAGATAACGAAAAGCATATAAACTGGGTTC
GAAGTGTTTATAATTTCACAACTCCTTATGTGTCCCAAAATCCAAGATTGGCGTATCTCAATTATAGGGACCT
TGATTTAGGAAAAACTAATCCTGAGAGTCCTAATAATTACACACAAGCACGTATTTGGGGTGAAAAGTATTTT
GGTAAAAATTTTAACAGGTTAGTTAAGGTGAAAACCAAAGCTGATCCCAATAATTTTTTTAGAAACGAACAAA
GTATCCCACCTCTTCCACCGCGTCATCATTAA
>A2_Jamaican_Lion_JAATIP010000055.1_F8388_016304
ATGAATTGCTCAACATTCTCCTTTTGGTTTGTTTGCAAAATAATATTTTTCTTTCTCTCATTCAATATCCAAA
TTTCAATAGCTAATCCTCAAGAAAACTTCCTTAAATGCTTCTCGGAATATATTCCTAACAATCCAGCAAATCC
AAAATTCATATACACTCAACACGACCAATTGTATATGTCTGTCCTGAATTCGACAATACAAAATCTTAGATTC
ACCTCTGATACAACCCCAAAACCACTCGTTATTGTCACTCCTTCAAATGTCTCCCATATCCAGGCCAGTATTC
TCTGCTCCAAGAAAGTTGGTTTGCAGATTCGAACTCGAAGCGGTGGCCATGATGCTGAGGGTTTGTCCTACAT
ATCTCAAGTCCCATTTGCTATAGTAGACTTGAGAAACATGCATACGGTCAAAGTAGATATTCATAGCCAAACT
GCGTGGGTTGAAGCCGGAGCTACCCTTGGAGAAGTTTATTATTGGATCAATGAGATGAATGAGAATTTTAGTT
TTCCTGGTGGGTATTGCCCTACTGTTGGCGTAGGTGGACACTTTAGTGGAGGAGGCTATGGAGCATTGATGCA
AAATTATGGCCTTGCGGCTGATAATATCATTGATGCACACTTAGTCAATGTTGATGGAAAAGTTCTAGATCGA
AAATCCATGGGAGAAGATCTATTTTGGGCTATACGTGGTGGAGGAGGAGAAAACTTTGGAATCATTGCAGCAT
GGAAAATCAAACTTGTTGTTGTCCCATCAAAGGCTACTATATTCAGTGTTAAAAAGAACATGGAGATACATGG
GCTTGTCAAGTTATTTAACAAATGGCAAAATATTGCTTACAAGTATGACAAAGATTTAATGCTCACGACTCAC
TTCAGAACTAGGAATATTACAGATAATCATGGGAAGAATAAGACTACAGTACATGGTTACTTCTCTTCCATTT

TTCTTGGTGGAGTGGATAGTCTAGTTGACTTGATGAACAAGAGCTTTCCTGAGTTGGGTATTAAAAAAACTGA
TTGCAAAGAATTGAGCTGGATTGATACAACCATCTTCTACAGTGGTGTTGTAAATTACAACACTGCTAATTTT
AAAAAGGAAATTTTGCTTGATAGATCAGCTGGGAAGAAGACGGCTTTCTCAATTAAGTTAGACTATGTTAAGA
AACTAATACCTGAAACTGCAATGGTCAAAATTTTGGAAAAATTATATGAAGAAGAGGTAGGAGTTGGGATGTA
TGTGTTGTACCCTTACGGTGGTATAATGGATGAGATTTCAGAATAAGCAATTCCATTCCCTCATCGAGCTGGA
ATAATGTATGAACTTTGGTACACTGCTACCTGGGAGAAGCAAGAAGATAACGAAAAGCATATAAACTGGGTTC
GAAGTGTTTATAATTTCACAACTCCTTATGTGTCCCAAAATCCAAGATTGGCGTATCTCAATTATAGGGACCT
TGATTTAGGAAAAACTAATCCTGAGAGTCCTAATAATTACACACAAGCACGTATTTGGGGTGAAAAGTATTTT
GGTAAAAATTTTAACAGGTTAGTTAAGGTGAAAACCAAAGCTGATCCCAATAATTTTTTTAGAAACGAACAAA
GTATCCCACCTCTTCCACCGCGTCATCATTAA
>A2_Finola_QKVJ02004358.1_21738
ATGAATTGCTCAACATTCTCCTTTTGGTTTGTTTGCAAAATAATATTTTTCTTTCTCTCTTTCAATATCCAAA
TTTCAATAGCTAATCCTCAAGAAAACTTCCTTAAATGCTTCTCGGAATATATTCCTAACAATCCAGCAAATCC
AAAATTCATATACACTCAACACGACCAATTGTATATGTCTGTCCTGAATTCGACAATACAAAATCTTAGATTC
ACCTCTGATACAACCCCAAAACCACTCGTTATTGTCACTCCTTCAAATGTCTCCCATATCCAGGCCAGTATTC
TCTGCTCCAAGAAAGTTGGTTTGCAGATTCGAACTCGAAGCGGTGGCCATGATGCTGAGGGTTTGTCCTACAT
ATCTCAAGTCCCATTTGCTATAGTAGACTTGAGAAACATGCATACGGTCAAAGTAGATATTCATAGCCAAACT
GCGTGGGTTGAAGCCGGAGCTACCCTTGGAGAAGTTTATTATTGGATCAATGAGATGAATGAGAATTTTAGTT
TTCCTGGTGGGTATTGCCCTACTGTTGGCGTAGGTGGACACTTTAGTGGAGGAGGCTATGGAGCATTGATGCG
AAATTATGGCCTTGCGGCTGATAATATCATTGATGCACACTTAGTCAATGTTGATGGAAAAGTTCTAGATCGA
AAATCCATGGGAGAAGATCTATTTTGGGCTATACGTGGTGGAGGAGGAGAAAACTTTGGAATCATTGCAGCAT
GGAAAATCAAACTTGTTGTTGTCCCATCAAAGGCTACTATATTCAGTGTTAAAAAGAACATGGAGATACATGG
GCTTGTCAAGTTATTTAACAAATGGCAAAATATTGCTTACAAGTATGACAAAGATTTAATGCTCACGACTCAC
TTCAGAACTAGGAATATTACAGATAATCATGGGAAGAATAAGACTACAGTACATGGTTACTTCTCTTCCATTT
TTCTTGGTGGAGTGGATAGTCTAGTTGACTTGATGAACAAGAGCTTTCCTGAGTTGGGTATTAAAAAAACTGA
TTGCAAAGAATTGAGCTGGATTGATACAACCATCTTCTACAGTGGTGTTGTAAATTACAACACTGCTAATTTT
AAAAAGGAAATTTTGCTTGATAGATCAGCTGGGAAGAAGACGGCTTTCTCAATTAAGTTAGACTATGTTAAGA
AACTAATACCTGAAACTGCAATGGTCAAAATTTTGGAAAAATTATATGAAGAAGAGGTAGGAGTTGGGATGTA
TGTGTTGTACCCTTACGGTGGTATAATGGATGAGATTTCAGAATCAGCAATTCCATTCCCTCATCGAGCTGGA
ATAATGTATGAACTTTGGTACACTGCTACCTGGGAGAAGCAAGAAGATAACGAAAAGCATATAAACTGGGTTC
GAAGTGTTTATAATTTCACAACTCCTTATGTGTCCCAAAATCCAAGATTGGCGTATCTCAATTATAGGGACCT
TGATTTAGGAAAAAAATAATCCTGAGAGTCCTAATAATTACACACAAGCACGTATTTGGGGTGAAAAGTATTTT
GGTAAAAATTTTAACAGGTTAGTTAAGGTGAAAACCAAAGCTGATCCCAATAATTTTTTTAGAAACGAACAAA
GTATCCCACCTCTTCCACCGCGTCATCATTAA
>A2_Jamaican_Lion_JAATIP010000055.1_F8388_016307
ATGAATTGCTCAACATTCTCCTTTTGGTTTGTTTGCAAAATAATATTTTTCTTTCTCTCATTCAATATCCAAA
TTTCAATAGCTAATCCTCAAGAAAACTTCCTTAAATGCTTCTCGGAATATATTCCTAACAATCCAGCAAATCC
AAAATTCATATACACTCAACACGACCAATTGTATATGTCTGTCTTGAATTCGACAATACAAAATCTTAGATTC
ACCTCTGATACAACCCCAAAACCACTCGTTATTGTCACTCCTTCAAATGTCTCCCATATCCAGGCCAGTATTC
TCTGCTCCAAGAAAGTTGGTTTGCAGATTCGAACTCGAAGCGGTGGCCATGATGCTGAGGGTTTGTCCTACAT
ATCTCAAGTCCCATTTGCTATAGTAGACTTGAGAAACATGCATACGGTCAAAGTAGATATTCATAGCCAAACT
GCGTGGGTTGAAGCCGGAGCTACCCTTGGAGAAGTTTATTATTGGATCAATGAGATGAATGAGAATTTTAGTT
TTCCTGGTGGGTATTGCCCTACTGTTGGCGTAGGTGGACACTTTAGTGGAGGAGGCTATGGAGCATTGATGCG
AAATTATGGCCTTGCGGCTGATAATATCATTGATGCACACTTAGTCAATGTTGATGGAAAAGTTCTAGATCGA
AAATCCATGGGAGAAGATCTATTTTGGGCTATACGTGGTGGAGGAGGAGAAAACTTTGGAATCATTGCAGCAT
GGAAAATCAAACTTGTTGTTGTCCCATCAAAGGCTACTATATTCAGTGTTAAAAAGAACATGGAGATACATGG
GCTTGTCAAGTTATTTAACAAATGGCAAAATATTGCTTACAAGTATGACAAAGATTTAATGCTCACGACTCAC
TTCAGAACTAGGAATATTACAGATAATCATGGGAAGAATAAGACTACAGTACATGGTTACTTCTCTTCCATTT
TTCTTGGTGGAGTGGATAGTCTAGTTGACTTGATGAACAAGAGCTTTCCTGAGTTGGGTATTAAAAAAACTGA
TTGCAAAGAATTGAGCTGGATTGATACAACCATCTTCTACAGTGGTGTTGTAAATTACAACACTGCTAATTTT
AAAAAGGAAATTTTGCTTGATAGATCAGCTGGGAAGAAGACGGCTTTCTCAATTAAGTTAGACTATGTTAAGA
AACTAATACCTGAAACTGCAATGGTCAAAATTTTGGAAAAATTATATGAAGAAGAGGTAGGAGTTGGGATGTA
TGTGTTGTACCCTTACGGTGGTATAATGGATGAGATTTCAGAATCAGCAATTCCATTCCCTCATCGAGCTGGA
ATAATGTATGAACTTTGGTACACTGCTACCTGGGAGAAGCAAGAAGATAACGAAAAGAATATAAACTGGGTTC
GAAGTGTTTATAATTTCACAACTCCTTATGTGTCCCAAAATCCAAGATTGGCGTATCTCAATTATAGGGACCT
TGATTTAGGAAAAACTAATCCTGAGAGTCCTAATAATTACACACAAGCACGTATTTGGGGTGAAAAGTATTTT
GGTAAAAATTTTAACAAGTTAGTTAAGGTGAAAACCAAAGCTGATCCCAATAATTTTTTTAGAAACGAACAAA
GTATCCCACCTCTTCCACCGCGTCATCATTAA

>A2_Jamaican_Lion_JAATIP010000055.1_F8388_016309
ATGAATTGCTCAACATTCTCCTTTTGGTTTGTTTGCAAAATAATATTTTTCTTTCTCTCATTCAATATCCAAA
TTTCAATAGCTAATCCTCAAGAAAACTTCCTTAAATGCTTCTCGGAATATATTCCTAACAATCCAGCAAATCC
AAAATTCATATACACTCAACACGACCAATTGTATATGTCTGTCCTGAATTCGACAATACAAAATCTTAGATTC
ACCTCTGATACAACCCCAAAACCACTCGTTATTGTCACTCCTTCAAATGTCTCCCATATCCAGGCCAGTATTC
TCTGCTCCAAGAAAGTTGGTTTGCAGATTCAAACTCGAAGCGGTGGCCATGATGCTGAGGGTTTGTCCTACAT
ATCTCAAGTCCCATTTGCTATAGCAGACTTGAGAAACATGCATACGGTCAAAGTAGATATTCATAGCCAAACT
GCGTGGGTTGAAGCCGGAGCTACCCTTGGAGAAGTTTATTATTGGATCAATGAGATGAATGAGAATTTTAGTT
TTCCTGGTGGGTATTGCCCTACTGTTGGCGTAGGTGGACACTTTAGTGGAGGAGGCTATGGAGCATTGATGCA
AAATTATGGCCTTGCGGCTGATAATATCATTGATGCACACTTAGTCAATGTTGATGGAAAAGTTCTAGATCGA
AAATCCATGGGAGAAGATCTATTTTGGGCTATACGTGGTGGAGGAGGAGAAAACTTTGGAATCATTGCAGCAT
GGAAAATCAAACTTGTTGTTGTCCCATCAAAGGCTACTATATTCAGTGTTAAAAAGAACATGGAGATACATGG
GCTTGTCAAGTTATTTAACAAATGGCAAAATATTGCTTACAAGTATGACAAAGATTTAATGCTCACGACTCAC
TTCAGAACTAGGAATATTACAGATAATCATGGGAAGAATAAGACTACAGTACATGGTTACTTCTCTTCCATTT
TTCTTGGTGGAGTGGATAGTCTAGTTGACTTGATGAACAAGAGCTTTCCTGAGTTGGGTATTAAAAAAACTGA
TTGCAAAGAATTGAGCTGGATTGATACAACCATCTTCTACAGTGGTGTTGTAAATTACAACACTGCTAATTTT
AAAAAGGAAATTTTGCTTGATAGATCAGCTGGGAAGAAGACGGCTTTCTCAATTAAGTTAGACTATGTTAAGA
AACTAATACCTGAAACTGCAATGGTCAAAATTTTGGAAAAATTATATGAAGAAGAGGTAGGAGTTGGGATGTA
TGTGTTGTACCCTTACGGTGGTATAATGGATGAGATTTCAGAATCAGCAATTCCATTCCCTCATCGAGCTGGA
ATAATGTATGAACTTTGGTACACTGCTACCTGGGAGAAGCAAGAAGATAACGAAAAGCATATAAACTGGGTTC
GAAGTGTTTATAATTTCACAACTCCTTATGTGTCCCAAAATCCAAGATTGGCGTACCTCAATTATAGGGACCT
TGATTTAGGAAAAACTAATCCTGAGAGTCCTAATAATTACACACAAGCACGTATTTGGGGTGAAAAGTATTTT
GGTAAAAATTTTAACAGGTTAGTTAAGGTGAAAACCAAAGCTGATCCCAATAATTTTTTTAGAAACGAACAAA
GTATCCCACCTCTTCCACCGCGTCATCATTAA
>A2_Jamaican_Lion_JAATIP010000055.1_F8388_016306
ATGAATTGCTCAACATTCTCCTTTTGGTTTGTTTGCAAAATAATATTTTTCTTTCTCTCATTCAATATCCAAA
TTTCAATAGCTAATCCTCAAGAAAACTTCCTTAAATGCTTCTCGGAATATATTCCTAACAATCCAGCAAATCC
AAAATTCATATACACTCAACACGACCAATTGTATATGTCTGTCCTGAATTCGACAATACAAAATCTTAGATTC
ACCTCTGATACAACCCCAAAACCACTCGTTATTGTCACTCCTTCAAATGTCTCCCATATCCAGGCCAGTATTC
TCTGCTCCAAGAAAGTTGGTTTGCAGATTCGAACTCGAAGCGGTGGCCATGATGCTGAGGGTTTGTCCTACAT
ATCTCAAGTCCCATTTGCTATAGTAGACTTGAGAAACATGCATACGGTCAAAGTAGATATTCATAGCCAAACT
GCGTGGGTTCAAGCCGGAGCTACCATTGGAGAAGTTTATTATTGGATCAATGAGATGAATGAGAATTTTAGTT
TTCCTGGTGGGTATTGCCCTACTGTTGGCGTAGGTGGACACTTTAGTGGAGGAGGCTATGGAGCATTGATGCG
AAATTATGGCCTTGCTGCTGATAATATCATTGATGCACACTTAGTCAATGTTGATGGAAAAGTTCTAGATCGA
AAATCCATGGGAGAAGATCTATTTTGGGCTATACGTGGTGGAGGAGGAGAAAACTTTGGAATCATTGCAGCAT
GGAAAATCAAACTTGTTGTTGTCCCATCAAAGGCTACTATATTCAGTGTTAAAAAGAACATGGAGATACATGG
GCTTGTCAAGTTATTTAACAAATGGCAAAATATTGCTTACAAGTATGACAAAGATTTAATGCTCACGACTCAC
TTCAGAACTAGGAATATTACAGATAATCATGGGAAGAATAAGACTACAGTACATGGTTACTTCTCTTCCATTT
TTCTTGGTGGAGTGGATAGTCTAGTTGACTTGATGAACAAGAGCTTTCCTGAGTTGGGTATTAAAAAAACTGA
TTGCAAAGAATTGAGCTGGATTGATACAACCATCTTCTACAGTGGTGTTGTAAATTACAACACTGCTAATTTT
AAAAAGGAAATTTTGCTTGATAGATCAGCTGGGAAGAAGACGGCTTTCTCAATTAAGTTAGACTATGTTAAGA
AACTAATACCTGAAACTGCAATGGTCAAAATTTTGGAAAAATTATATGAAGAAGAGGTAGGAGTTGGGATGTA
TGTGTTGTACCCTTACGGTGGTATAATGGATGAGATTTCAGAATCAGCAATTCCATTCCCTCATCGAGCTGGA
ATAATGTATGAACTTTGGTACACTGCTACCTGGGAGAAGCAAGAAGATAACGAAAAGCATATAAACTGGGTTC
GAAGTGTTTATAATTTCACAACTCCTTATGTGTCCCAAAATCCAAGATTGGCGTATCTCAATTATAGGGACCT
TGATTTAGGAAAAACTAATCCTGAGAGTCCTAATAATTACACACAAGCACGTATTTGGGGTGAAAAGTATTTT
GGTAAAAATTTTAACAGGTTAGTTAAGGTGAAAACCAAAGCTGATCCCAATAATTTTTTTAGAAACGAACAAA
GTATCCCACCTCTTCCACCGCGTCATCATTAA
>A2_Jamaican_Lion_JAATIP010000055.1_F8388_016308
ATGAATTGCTCAACATTCTCCTTTTGGTTTGTTTGCAAAATAATATTTTTCTTTCTCTCATTCAATATCCAAT
TTTCAATAGCTAATCCTCAAGAAAACTTCCTTAAATGCTTCTCGGAATATATTCCTAACAATCCAGCAAATCC
AAAATTCATATACACTCAACACGACCAATTGTATATGTCATTCCTGAATTCGACAATACAAAATCTTAGATTC
ACCTCTGATACAACCCCAAAACCACTCGTTATTGTCACTCCTTCAAATGTCTCCCATATCCAGGCCAGTATTC
TCTGCTCCAAGAAAGTTGGTTTGCAGATTCGAACTCGAAGCGGTGGCCATGATGCTGAGGGTTTGTCCTACAT
ATCTCAAGTCCCATTTGCTATAGTAGACTTGAGAAACATGCATACGGTCAAAGTAGATATTCATAGCCAAACT
GCGTGGGTTGAAGCCGGAGCTACCCTTGGAGAAGTTTATTATTGGATCAATGAGATGAATGAGAATTTTAGTT
TTCCTGGTGGGTATTGCCCTACTGTTGGCGTAGGTGGACACTTTAGTGGAGGAGGCTATGGAGCATTGATGCG
AAATTATGGCCTTGCGGCTGATAATATCATTGATGCACACTTAGTCAATGTTGATGGAAAAGTTCTAGATCGA

AAATCCATGGGAGAAGATCTATTTTGGGCTATACGTGGTGGAGGAGGAGAAAACTTTGGAATCATTGCAGCAT
GGAAAATCAAACTTGTTGTTGTCCCATCAAAGGCTACTATATTCAGTGTTAAAAAGAACATGGAGATACATGG
GCTTGTCAAGTTATTTAACAAATGGCAAAATATTGCTTACAAGTATGACAAAGATTTAATGCTCACGACTCAC
TTCAGAACTAGGAATATTACAGATAATCATGGGAAGAATAAGACTACAGTACATGGTTACTTCTCTTCCATTT
TTCTTGGTGGAGTGGATAGTCTAGTTGACTTGATGAACAAGAGCTTTCCTGAGTTGGGTATTAAAAAAACTGA
TTGCAAAGAATTGAGCTGGATTGATACAACCATCTTCTACAGTGGTGTTGTAAATTACAACACTGCTAATTTT
AAAAAGGAAATTTTGCTTGATAGATCAGCTGGGAAGAAGACGGCTTTCTCAATTAAGTTAGACTATGTTAAGA
AACTAATACCTGAAACTGCAATGGTCAAAATTTTGGAAAAATTATATGAAGAAGAGGTAGGAGTTGGGATGTA
TGTGTTGTACCCTTACGGTGGTATAATGGATGAGATTTCAGAATCAGCAATTCCATTCCCTCATCGAGCTGGA
ATAATGTATGAACTTTGGTACACTGCTACCTGGGAGAAGCAAGAAGATAACGAAAAGCATATAAACTGGGTTC
GAAGTGTTTATAATTTCACAACTCCTTATGTGTCCCAAAATCCAAGATTGGCGTATCTCAATTATAGGGACCT
TGATTTAGGAAAAAAATAATCCTGAGAGTCCTAATAATTACACACAAGCACGTATTTGGGGTGAAAAGTATTTT
GGTAAAAATTTTAACAGGTTAGTTAAGGTGAAAACCAAAGCTGATCCCAATAATTTTTTTAGAAACGAACAAA
GTATCCCACCTCTTCCACCGCGTCATCATTAA
>A2_Finola_QKVJ02001794.1_11056
ATGAATTGCTCAACATTCTCCTTTTGGTTTTGCAAAATAATATTTTTCTTTCTCTCATTCAATATCCAAATTT
CAATAGCTAATCCTCAAGAAAACTTCCTTAAATGCTTCTCGGAATATATTCCTAACAATCCAGCAAATCCAAA
ATTCATATACACTCAACACGACCAATTGTATATGTCTGTCCTGAATTCGACAATACAAAATCTTAGATTCACC
TCTGATACAACCCCAAAACCACTCGTTATTGTCACTCCTTCAAATGTCTCCCATATCCAGGCCAGTATTCTCT
GCTCCAAGAAAGTTGGTTTGCAGATTCGAACTCGAAGCGGTGGCCATGATGCTGAGGGTTTGTCCTACATATC
TCAAGTCCCATTTGCTATAGTAGACTTGAGAAACATGCATACGGTCAAAGTAGATATTCATAGCCAAACTGCG
TGGGTTGAAGCCGGAGCTACCCTTGGAGAAGTTTATTATTGGATCAATGAGATGAATGAGAATTTTAGTTTTC
CTGGTGGGTATTGCCCTACTGTTGGCGTAGGTGGACACTTTAGTGGAGGAGGCTATGGAGCATTGATGCGAAA
TTATGGCCTTGCGGCTGATAATATCATTGATGCACACTTAGTCAATGTTGATGGAAAAGTTCTAGATCGAAAA
TCCATGGGAGAAGATCTATTTTGGGCTATACGTGGTGGAGGAGGAGAAAACTTTGGAATCATTGCAGCATGGA
AAATCAAACTTGTTGTTGTCCCATCAAAGGCTACTATATTCAGTGTTAAAAAGAACATGGAGATACATGGGCT
TGTCAAGTTATTTAACAAATGGCAAAATATTGCTTACAAGTATGACAAAGATTTAATGCTCACGACTCACTTC
AGAACTAGGAATATTACAGATAATCATGGGAAGAATAAGACTACAGTACATGGTTACTTCTCTTCCATTTTTC
TTGGTGGAGTGGATAGTCTAGTTGACTTGATGAACAAGAGCTTTCCTGAGTTGGGTATTAAAAAAACTGATTG
CAAAGAATTGAGCTGGATTGATACAACCATCTTCTACAGTGGTGTTGTAAATTACAACACTGCTAATTTTAAA
AAGGAAATTTTGCTTGATAGATCAGCTGGGAAGAAGACGGCTTTCTCAATTAAGTTAGACTATGTTAAGAAAC
TAATACCTGAAACTGCAATGGTCAAAATTTTGGAAAAATTATATGAAGAAGAGGTAGGAGTTGGGATGTATGT
GTTGTACCCTTACGGTGGTATAATGGATGAGATTTCAGAATCAGCAATTCCATTCCCTCATCGAGCTGGAATA
ATGTATGAACTTTGGTACACTGCTACCTGGGAGAAGCAAGAAGATAACGAAAAGCATATAAACTGGGTTCGAA
GTGTTTATAATTTCACAACTCCTTATGTGTCCCAAAATCCAAGATTGGCGTATCTCAATTATAGGGACCTTGA
TTTAGGAAAAACTAATCCTGAGAGTCCTAATAATTACACACAAGCACGTATTTGGGGTGAAAAGTATTTTGGT
AAAAATTTTAACAGGTTAGTTAAGGTGAAAACCAAAGCTGATCCCAATAATTTTTTTAGAAACGAACAAAGTA
TCCCACCTCTTCCACCGCGTCATCATTAA
>A2_Finola_QKVJ02004488.1_6165
ATGAATTGCTCAACATTCTCCTTTTGGTTTTGCAAAATAATATTTTTCTTTCTCTCATTCAATATCCAAATTT
CAATAGCTAATCCTCAAGAAAACTTCCTTAAATGCTTCTCGGAATATATTCCTAACAATCCAGCAAATCCAAA
ATTCATATACACTCAACACGACCAATTGTATATGTCTGTCCTGAATTCGACAATACAAAATCTTAGATTCACC
TCTGATACAACCCCAAAACCACTCGTTATTGTCACTCCTTCAAATGTCTCCCATATCCAGGCCAGTATTCTCT
GCTCCAAGAAAGTTGGTTTGCAGATTCGAACTCGAAGCGGTGGCCATGATGCTGAGGGTTTGTCCTACATATC
TCAAGTCCCATTTGCTATAGTAGACTTGAGAAACATGCATACGGTCAAAGTAGATATTCATAGCCAAACTGCG
TGGGTTGAAGCCGGAGCTACCCTTGGAGAAGTTTATTATTGGATCAATGAGATGAATGAGAATTTTAGTTTTC
CTGGTGGGTATTGCCCTACTGTTGGCGTAGGTGGACACTTTAGTGGAGGAGGCTATGGAGCATTGATGCGAAA
TTATGGCCTTGCGGCTGATAATATCATTGATGCACACTTAGTCAATGTTGATGGAAAAGTTCTAGATCGAAAA
TCCATGGGAGAAGATCTATTTTGGGCTATACGTGGTGGAGGAGGAGAAAACTTTGGAATCATTGCAGCATGGA
AAATCAAACTTGTTGTTGTCCCATCAAAGGCTACTATATTCAGTGTTAAAAAGAACATGGAGATACATGGGCT
TGTCAAGTTATTTAACAAATGGCAAAATATTGCTTACAAGTATGACAAAGATTTAATGCTCACGACTCACTTC
AGAACTAGGAATATTACAGATAATCATGGGAAGAATAAGACTACAGTACATGGTTACTTCTCTTCCATTTTTC
TTGGTGGAGTGGATAGTCTAGTTGACTTGATGAACAAGAGCTTTCCTGAGTTGGGTATTAAAAAAACTGATTG
CAAAGAATTGAGCTGGATTGATACAACCATCTTCTACAGTGGTGTTGTAAATTACAACACTGCTAATTTTAAA
AAGGAAATTTTGCTTGATAGATCAGCTGGGAAGAAGACGGCTTTCTCAATTAAGTTAGACTATGTTAAGAAAC
TAATACCTGAAACTGCAATGGTCAAAATTTTGGAAAAATTATATGAAGAAGAGGTAGGAGTTGGGATGTATGT
GTTGTACCCTTACGGTGGTATAATGGATGAGATTTCAGAATCAGCAATTCCATTCCCTCATCGAGCTGGAATA
ATGTATGAACTTTGGTACACTGCTACCTGGGAGAAGCAAGAAGATAACGAAAAGCATATAAACTGGGTTCGAA

GTGTTTATAATTTCACAACTCCTTATGTGTCCCAAAATCCAAGATTGGCGTATCTCAATTATAGGGACCTTGA
TTTAGGAAAAACTAATCCTGAGAGTCCTAATAATTACACACAAGCACGTATTTGGGGTGAAAAGTATTTTGGT
AAAAATTTTAACAGGTTAGTTAAGGTGAAAACCAAAGCTGATCCCAATAATTTTTTTAGAAACGAACAAAGTA
TCCCACCTCTTCCACCGCGTCATCATTAA
>A2_Purple_Kush__CM010797.2_46551515
ATGAATTGCTCAACATTCTCCTTTTGGTTTGTTTGCAAAATAATATTTTTCTTTCTCTCATTCAATATCCAAA
TTTCAATAGCTAATCCTCAAGAAAACTTCCTTAAATGCTTCTCGGAATATATTCCTAACAATCCAGCAAATCC
AAAATTCATATACACTCAACACGACCAATTGTATATGTCTGTCCTGAATTCGACAATACAAAATCTTAGATTC
ACCTCTGATACAACCCCAAAACCACTCGTTATTGTCACTCCTTCAAATGTCTCCCATATCCAGGCCAGTATTC
TCTGCTCCAAGAAAGTTGGTTTGCAGATTCGAACTCGAAGCGGTGGCCATGATGCTGAGGGTTTGTCCTACAT
ATCTCAAGTCCCATTTGCTATAGTAGACTTGAACATGCATACGGTCAAAGTAGATATTCATAGCCAAACTGCG
TGGGTTGAAGCCGGAGCTACCCTTGGAGAAGTTTATTATTGGATCAATGAGATGAATGAGAATTTTAGTTTTC
CTGGTGGGTATTGCCCTACTGTTGGCGTAGGTGGACACTTTAGTGGAGGAGGCTATGGAGCATTGATGCGAAA
TTATGGCCTTGCGGCTGATAATATCATTGATGCACACTTAGTCAATGTTGATGGAAAAGTTCTAGATCGAAAA
TCCGGAGAAGATCTATTTTGGGCTATACGTGGTGGAGGAGGAGAAAACTTTGGAATCATTGCAGCATGGAAAA
TCAAACTTGTTGTTGTCCCATCAAAGGCTACTATATTCAGTGTTAAAAAGAACATGGAGATACATGGGCTTGT
CAAGTTATTTAACAAATGGCAAAATATTGCTTACAAGTATGACAAAGATTTAATGCTCACGACTCACTTCACT
AGGAATATTACAGATAATCATGGGAAGAATAAGACTACAGTACATGGTTACTTCTCTTCCATTTTTCTTGGTG
GAGTGGATAGTCTAGTTGACTTGATGAACAAGAGCTTTCCTGAGTTGGGTATTAAAAAAACTGATTGCAAAGA
ATTGAGCTGGATTGATACAACCATCTTCTACAGTGGTGTTGTAAATTACAACACTGCTAATTTTAAAAAGGAA
ATTTTGCTTGATAGATCAGCTGGGAAGAAGACGGCTTTCTCAATTAAGTTAGACTATGTTAAGAAACTAATAC
CTGAAACTGTAATGGTCAAAATTTTGGAAAAATTATATGAAGAAGAGGTAGGAGTTGGGATGTATGTGTTGTA
CCCTTACGGTGGTATAATGGATGAGATTTCAGAATCAGCAATTCCATTCCCTCATCGAGCTGGAATAATGTAT
GAACTTTGGTACACTGCTACCTGGGAGAAGCAAGAAGATAACGAAAAGCATATAAACTGGGTTCGAAGTGTTT
ATAATTTCACAACTCCTTATGTGTCCCAAAATCCAAGATTGGCGTATCTCAATTATAGGGACCTTGATTTAGG
AAAAACTAATCCTGAGAGTCCTAATAATTACACACAAGCACGTATTTGGGGTGAAAAGTATTTTGGTAAAAAT
TTTAACAGGTTAGTTAAGGTGAAAACCAAAGCTGATCCCAATAATTTTTTTAGAAACGAACAAAGTATCCCAC
CTCTTCCACCGCGTCATCATTAA
>A2_Finola_QKVJ02001794.1_138341
ATGAATTGCTCAACATTCTCCTTTTGGTTTGTTTGCAAAATAATATTTTTCTTTCTCTCATTCAATATCCAAA
TTTCAATAGCTAATCCTCAAGAAAACTTCCTTAAATGCTTCTCGGAATATATTCCTAACAATCCAGCAAATCC
AAAATTCATATACACTCAACACGACCAATTGTATATGTCTGTCCTGAATTCGACAATACAAAATCTTAGATTC
ACCTCTGATACAACCCCAAAACCACTCGTTATTGTCACTCCTTCAAATGTCTCCCATATCCAGGCCAGTATTC
TCTGCTCCAAGAAAGTTGGTTTGCAGATTCGAACTCGAAGCGGTGGCCATGATGCTGAGGGTTTGTCCTACAT
ATCTCAAGTCCCATTTGCTATAGTAGACTTGAGAAACATGCATACGGTCAAAGTAGATATTCATAGCCAAACT
GCGTGGGTTGAAGCCGGAGCTACCCTTGGAGAAGTTTATTATTGGATCAATGAGATGAATGAGAATTTTAGTT
TTCCTGGTGGGTATTGCCCTACTGTTGGCGTAGGTGGACACTTTAGTGGAGGAGGCTATGGAGCATTGATGCA
AAATGGCCTTGCGGCTGATAATATCATTGATGCACACTTAGTCAATGTTGATGGAAAAGTTCTAGATCGAAAA
TCCATGGGAGAAGATCTATTTTGGGCTATACGTGGTGGAGGGGAAAACTTTGGAATCATTGCAGCATGGAAAA
TCAAACTTGTTGTTGTCCCATCAAAGGCTACTATATTCAGTGTTAAAAAGAACATGGAGATACATGGGCTTGT
CAAGTTATTTAACAAATGGCAAAATATTGCTTACAAGTATGACAAAGATTTAATGCTCACGACTCACTTCAGA
ACTAGGAATATTACAGATAATCATGGGAAGAATAAGACTACAGTACATGGTTACTTCTCTTCCATTTTTCTTG
GTGGAGTGGATAGTCTAGTTGACTTGATGAACAAGAGCTTTCCTGAGTTGGGTATTAAAAAAACTGATTGCAA
AGAATTGAGCTGGATTGATACAACCATCTTCTACAGTGGTGTTGTAAATTACAACACTGCTAATTTTAAAAAG
GAAATTTTGCTTGATAGATCAGCTGGGAAGAAGACGGCTTTCTCAATTAAGTTAGACTATGTTAAGAAACTAA
TACCTACTGCAATGGTCAAAATTTTGGAAAAATTATATGAAGAAGAGGTAGGAGTTGGGATGTATGTGTTGTA
CCCTTACGGTGGTATAATGGATGAGATTTCAGAATCAGCAATTCCATTCCCTCATCGAGCTGGAATAATGTAT
GAACTTTGGTACACTGCTACCTGGGAGAAGCAAGAAGATAACGAAAAGCATATAAACTGGGTTCGAAGTGTTT
ATAATTTCACAACTCCTTATGTGTCCCAAAATCCAAGATTGGCGTATCTCAATTATAGGGACCTTGATTTAGG
AAAAACTAATCCTGAGAGTCCTAATAATTACACACAAGCACGTATTTGGGGTGAAAAGTATTTTGGTAAAAAT
TTTAACAGGTTAGTTAAGGTGAAAACCAAAGCTGATCCCAATAATTTTTTTAGAAACGAACAAAGTATCCCAC
CTCTTCCACCGCGTCATCATTAA
>A2_CBDRx_NC_044378.1_26045537
ATGAATTGCTCAACATTCTCCTTTTGGTTTGTTTGCAAAATAATATTTTTCTTTCTCTCATTCAATATCCAAA
TTTCAATAGCTAATCCTCAAGAAAACTTCCTTAAATGCTTCTCGGAATATATTCCTAACAATCCAGCAAATCC
AAAATTCATATACACTCAACACGACCAATTGTATATGTCTGTCCTGAATTCGACAATACAAAATCTTAGATTC
ACCTCTGATACAACCCCAAAACCACTCGTTATTGTCACTCCTTCAAATGTCTCCCATATCCAGGCCAGTATTC
TCTGCTCCAAGAAAGTTGGTTTGCAGATTCGAACTCGAAGCGGTGGCCATGATGCTGAGGGTTTGTCCTACAT

ATCTCAAGTCCCATTTGCTATAGTAGACTTGAGAAACATGCATACGGTCAAAGTAGATATTCATAGCCAAACT
GCGTGGGTTGAAGCCGGAGCTACCCTTGGAGAAGTTTATTATTGGATCAATGAGATGAATGAGAATTTTAGTT
TTCCTGGTGGGTATTGCCCTACTGTTGGCGTAGGTGGACACTTTAGTGGAGGAGGCTATGGAGCATTGATGCG
AAATTATGGCCTTGCGGCTGATAATATCATTGATGCACACTTAGTCAATGTTGATGGAAAAGTTCTAGATCGA
AAATCCATGGGAGAAGATCTATTTTGGGCTATACGTGGTGGAGGAGGAGAAAACTTTGGAATCATTGCAGCAT
GGAAAATCAAACTTGTTGTTGTCCCATCAAAGGCTACTATATTCAGTGTTAAAAAGAACATGGAGATACATGG
GCTTGTCAAGTTATTTAACAAATGGCAAAATATTGCTTACAAGTATGACAAAGATTTAATGCTCACGACTCAC
TTCAGAACTAGGAATATTACAGATAATCATGGGAAGAATAAGACTACAGTACATGGTTACTTCTCTTCCATTT
TTCTTGGTGGAGTGGATAGTCTAGTTGACTTGATGAACAAGAGCTTTCCTGAGTTGGGTATTAAAGCTGATTG
CAAAGAATTGAGTATTGATACAACCATCTTCTACAGTGGTGTTGTAAATTACAACACCGCTAATTTTAAGGAA
ATTTTGCTTGATAGATCATTTGGGAAGAAGACGGCTTTCTCAATTAAGTTAGACTATGTTAAGAAACTAATAC
CTGAAACTGTAATGGTCAAAATTTTGGAAAAATTATATGAAGAAGAGGTAGGAGTTGGGATGTATGTGTTGTA
CCCTTACGGTGGTATAATGGATGAGATTTCAGAATCAGCAATTCCATTCCCTCATCGAGCTGGAATAATGTAT
GAACTTTGGTACACTGCTACCTGGGAGAAGCAAGAAGATAACGAAAAGCATATAAACTGGGTTCGAAGTGTTT
ATAATTTCACAACTCCTTATGTGTCCCAAAATCCAAGATTGGCGTATCTCAATTATAGGGACCTTGATTTAGG
AAAAACTAATCCTGAGAGTCCTAATAATTACACACAAGCACGTATTTGGGGTGAAAAGTATTTTGGTAAAAAT
TTTAACAGGTTAGTTAAGGTGAAAACCAAAGCTGATCCCAATAATTTTTTTAGAAACGAACAAAGTATCCCAC
CTCTTCCACCGCGTCATCATTAA

>A2_Purple_Kush__AGQN03010271.1_4605

ATGAATTGCTCAACATTCTCCTTTTGGTTTGTTTGCAAAATAATATTTTTCTTTCTCTCATTCAATATCCAAA
TTTCAATAGCTAATCCTCAAGAAAACTTCCTTAAATGCTTCTCGGAATATATTCCTAACAATCCAGCAAATCC
AAAATTCATATACACTCAACACGACCAATTGTATATGTCTGTCCTGAATTCGACAATACAAAATCTTAGATTC
ACCTCTGATACAACCCCAAAACCACTCGTTATTGTCACTCCTTCAAATGTCTCCCATATCCAGGCCAGTATTC
TCTGCTCCAAGAAAGTTGGTTTGCAGATTCGAACTCGAAGCGGTGGCCATGATGCTGAGGGTTTGTCCTACAT
ATCTCAAGTCCCATTTGCTATAGTAGACTTGAGAAACATGCATACGGTCAAAGTAGATATTCATAGCCAAACT
GCGTGGGTTGAAGCCGGAGCTACCCTTGGAGAAGTTTATTATTGGATCAAGATGAATGAGAATTTTAGTTTTC
CTGGTGGGTATTGCCCTACTGTTGGCGTAGGTGGACACTTTAGTGGAGGAGGCTATGGAGCATTGATGCGAAA
TTATGGCCTTGCGGCTGATAATATCATTGATGCACACTTAGTCAATGTTGATGGAAAAGTTCTAGATCGAAAA
TCCATGGGAGAAGATCTATTTTGGGCTATACGTGGTGGAGGAGGAGAAAACTTTGGAATCATTGCAGCATGGA
AAATCAAACTTGTTGTTGTCCCATCAAAGGCTACTATATTCAGTGTTAAAAAGAACATGGAGATACATGGGCT
TGTCAAGTTATTTAACAAATGGCAAAATATTGCTTACAAGTATGACAAAGATTTAATGCTCACGACTCACTTC
AGAACTAGGAATATTACAGATAATCATGGGAAGAATAAGACTACAGTACATGGTTACTTCTCTTCCATTTTTC
TTGGTGGAGTGGATAGTCTAGTTGACTTGATGAACAAGAGCTTTCCTGAGTTGGGTATTAAAAAAACTGATTG
CAAAGAATTGAGCTGGATTGATACAACCATCTTCTACAGTGGTGTTGTAAATTACAACACTGCTAATTTTAAA
AAGGAAATTTTCGATAGATCAGCTGGGAAGAAGACGGCTTTCTCAATTAAGTTAGACTATGTTAAGAAACTAA
TACCTGAAACTGCAATGGTCAAAATTTTGGAAAAATTATATGAAGAAGAGGTAGGAGTTGGGATGTATGTGTT
GTACCCTTACGGTATAATGGATGAGATTTCAGAATCAGCAATTCCATTCCCTCATCGAGCTGGAATAATGTAT
GAACTTTGGTACACTGCTACCTGGGAGAAGCAAGAAGATAACGAAAAGCATATAAACTGGGTTCGAAGTGTTT
ATAATTTCACAACTCCTTATGTGTCCCAAAATCCAAGATTGGCGTATCTCAATTATAGGGACCTTGATTTAGG
AAAAAATCCTGAGAGTCCTAATAATTACACACAAGCACGTATTTGGGGTGAAAAGTATTTTGGTAAAAATTTT
AACAGGTTAGTTAAGGTGAAAACCAAAGCTGATCCCAATAATTTTTTTAGAAACGAACAAAGTATCCCACCTC
TTCCACCGCGTCATCATTAA

>A2_CBDRx_NC_044378.1_25982099

ATGAATTGCTCAACATTCTCCTTTTGGTTTGTTTGCAAAATAATATTTTTCTTTCTCTCATTCAATATCCAAA
TTTCAATAGCTAATCCTCAAGAAAACTTCCTTAAATGCTTCTCGGAATATATTCCTAACAATCCAGCAAATCC
AAAATTCATATACACTCAACACGACCAATTGTATATGTCTGTCCTGAATTCGACAATACAAAATCTTAGATTC
ACCTCTGATACAACCCCAAAACCACTCGTTATTGTCACTCCTTCAAATGTCTCCCATATCCAGGCCAGTATTC
TCTGCTCCAAGAAAGTTGGTTTGCAGATTCGAACTCGAAGCGGTGGCCATGATGCTGAGGGTTTGTCCTACAT
ATCTCAAGTCCCATTTGCTATAGTAGACTTGAGAAACATGCATACTGTCAAAGTAGATATTCATAGCCAAGCT
CAGTGGGTTGAAGCCTGAGCTACCCTTGGAGAAGTTTATTATTGGATCAATGAGATGAATGAGAATTTTAGTT
TTCCTGGTGGGTATTGCCCCGTTGGCGGTGGACACTTTAGTGGAGGAGGCTATGGAGCATTGATGCGAAATTA
TGGCCTCACTGATAATATCATTGATGCACACTTAGTCAATGTTGATGGAAAAGTTCTAGATCGAAAATCCATG
GGAGAAGATCTATTTTGGGCTATACGTGGTGGAGGAGGAGAAAACTTTGGAATCATTGCAGCATGGAAAATCA
AACTTGTTGTTGTCCCATCAAAGGCTACTATATTCAGTGTTAAAAAGAACATGGAGATACATGGGCTTGTCAA
GTTATTTAACAAATGGCAAAATATTGCTTACAAGTATGACAAAGATTTAATGCTCACGACTCACTTCAGAACT
AGGAATATTACAGATAATCATGGGAAGAATAAGACTACAGTACATGGTTACTTCTCTTCCATTTTTCTTGGTG
GAGTGGATAGTCTAGTTGACTTGATGAACAAGAGCTTTCCTGAGTTGGGTATTAAAAAAACTGATTGCAAAGA
ATTGAGCTGGATTGATACAACCATCTTCTACAGTGGTGTTGTAAATTACAACACTGCTAATTTTAAAAAGGAA

ATTTTGCTTGATAGATCAGCTGGGAAGAAGACGGCTTTCTCAATTAAGTTAGACTATGTTAAGAAACTAATAC
CTGAAACTGCAATGGTCAAAATTTTGGAAAAATTATATGAAGAAGAGGTAGGAGTTGGGATGTATGTGTTGTA
CCCTTACGGTGGTATAATGGATGAGATTTCAGAATCAACAATTCCATTCCCTCATCGAGCTGGAATAATGTAT
GAACTTTGGTACACTGCTACCTGGGAGAAGCAAGAAGATAACGAAAAGCATATAAACTGGGTTCGAAGTGTTT
ATAATTTCACAACTCCTTATGTGTCCCAAAATCCAAGATTGGCGTATCTCAATTATAGGGACCTTGATTTAGG
AAAAACTAATCCTGAGAGTCCTAATAATTACACACAAGCACGTATTTGGGGTGAAAAGTATTTTGGTAAAAAT
TTTAACAGGTTAGTTAAGGTGAAAACCAAAGCTGATCCCAATAATTTTTTTAGAAACGAACAAAGTATCCCAC
CTCTTCCACCGCGTCATCATTAA
>A2_CBDRx_NC_044378.1_26085884
ATGAATTGCTCAACATTCTCCTTTTGGTTTGTTTGCAAAATAATATTTTTCTTTCTCTCATTCAATATCCAAA
TTTCAATAGCTAATCCTCAAGAAAACTTCCTTAAATGCTTCTCGGAATATATTCCTAACAATCCAGCAAATCC
AAAATTCATATACACTCAACACGACCAATTGTATATGTCTGTCCTGAATTCGACAATACAAAATCTTAGATTC
ACCTCTGATACAACCCCAAAACCACTCGTTATTGTCACTCCTTCAAATGTCTCCCATATCCGGGCCAAGATTC
TCTGCTCCAAGAAAGTTGGTTTGCAGATTCGAACTCGAAGCGGTGGCCATGATGCTGAGGGTTTGTCCTACAT
ATCTCAAGTCCCATTTGCTATAGTAGACTTGAGAAACATGCATACGGTCAAAGTAGATATTCATAGCCAAACT
GCGTGGGTTGAAGCCGGAGCTACCCTTGGAGAAGTTTATTATTGGATCAATGAGATGAATGAGAATTTTAGTT
TTCCTGGTGGGTATTGCCCTCTTGTTGGTGGACACTTTAGTGGAGGAGGCTATGGAGCATTGATGCGAAATTA
TGGCCTAGGTGATAATATCATTGATGCACACTTAGTCAATGTTGATGGAAAAGTTCTAGATCGAAAATCCATG
GGAGAAGATCTATTTTGGGCTATACGTGGTGGAGGAGGAGAAAACTTTGGAATCATTGCAGCATGGAAAATCA
AACTTGTTGTTGTCCCATCAAAGGCTACTATATTCAGTGTTAAAAAGAACATGGAGATACATGGGCTTGTCAA
GTTATTTAACAAATGGCAAAATATTGCTTACAAGTATGACAAAGATTTAATGCTCACGACTCACTTCAGAACT
AGGAATATTACAGATAATCATGGGAAGAATAAGACTACAGTACATGGTTACTTCTCTTCCATTTTTCTTGGTG
GAGTGGATAGTCTAGTTGACTTGATGAACAAGAGCTTTCCTGAGTTGGGTATTAAAAAAACTGATTGCAAAGA
ATTGAGCTGGATTGATACAACCATCTTCTACAGTGGTGTTGTAAATTACAACACTGCTAATTTTAAAAAGGAA
ATTTTGCTTGATAGATCAGCTGGGAAGAAGACGGCTTTCTCAATTAAGTTAGACTATGTTAAGAAACTAATAC
CTGAAACTGCAATGGTCAAAATTTTGGAAAAATTATATGAAGAAGAGGTAGGAGTTGGGATGTATGTGTTGTA
CCCTTACGGTGGTATAATGGATGAGATTTCAGAATCAGCAATTCCATTCCCTCATCGAGCTGGAATAATGTAT
GAACTTTGGTACACTACACTGGAGAAGCAAGAAGATAACGAAAAGCATATAAACTGGGTTCGAAGTGTTTATA
ATTTCACAACTCCTTATGTGTCCCAAAATCCAAGATTGGCGTATCTCAATTATAGGGACCTTGATTTAGGAAA
AACTAATCCCAGGAGTCCTAATAATTACACACAAGCATGTATTTGGGGTGAAAAGTATTTTAGTAAAAATTTT
AACAGGTTTGTTAAGGTGAAAACCAAGGTTGATCCCAATAATTTTTTTAGAAACGAACAAAGCATCCCACCTC
TTCCACCGCGTCATCATTAA
>A2_CBDRx_NC_044378.1_26171983
ATGAATTGCTCAACATTCTCCTTTTGGTTTGTTTGCAAAATAATATTCTTTCTCTCATTCAATATCCAAATTT
CAATAGCTAATCCTCAAGAAAACTTCCTTAAATGCTTCTCTGAATATATTCCTAACAATCCAAGAAATCCAAA
ATTCATATACACTCAACACGACCAATTGTATATGTCGACAGATTCGACAATACAAAATCTTAGATTCACCTCG
ACAACCCCAAAACCACTCGTTATTGTCACTCCTTCAAATGTCTCCCATATCCAGGCCAGATTCTGCTCCAAGA
AAGTTGGTTTGCAGATTAGCGGTGGCCATGATGAGGGTTTGTACATATCTCAAGTCCCATTTGCTATAGTAGA
CTTGAGAAACATGCATCTGGTCAAAGTAGATATTCATAGCCAAGCGTGGGTTGAAGCTTGAGCTACCCTTGGA
GAAGTTTATTATTGGATCAATGAGATGAATGAGAATTTTAGTTTTCCGGGGTATTGCCCTCTTGTTGGCGTGA
GTGGACACTTTAGTGGAGGAGGCTATGGAGCATTGATGCGAAATTATGGCCTTGGTGATAATATCATTGATGC
ACACTTAGTCAATGTTGATGGAAAAGTTCTAGATCGAAAATCCATGGGAGAAGATCTATTTTGGGCTATCGGT
GGAGGAGGAGAAAACTTTGGAATCATTGCAGCATGGAAAATCAAACTTGTTGTTGTCCCATCAAAGGCTACTA
TATTCAGTGTTAAGAACATGGAGATACATGGGCTTGTCAAGTTATTTAACAAATGGCAAAATATTGCTTACAA
GTATGACAAAGATTTAATGCTCACGACTCACTTGACTAGGAATATTGATAATCATGGGAAGAATAAGACTACA
GCACATGGTTACTTCTCTTCCATTTTTCTTGGTGGAGTGGATAGTCTAGTTGACTTGATGAACAAGAGCTTTG
AGTTGGGTATTAAAACTGATTGCAAAGAATTGAGTATTGATACAACCATCTACGATGGTGTTGTAAATTACAA
CACCAATTTTAAGGAAATTTTGCTTGATAGATCATTTGGGAAGAAGACGGCTTTCTCAATTAAGTTAGACTAT
GTTAAGAAACTAATACACACTGCAATGGTCAAAATTTTGGAAAAATTATATGAAGAAGAGGTAGGAGTTGGGA
TGTATGTGTTGTACCCTTACGGTGGTATAATGGATGAGATTTCGTCAACAATTCCATTCCCTCATCGAGTTGG
AATAATGTATGAACTTTGGTACATCTGCCTGGAGAAGCAAGAAGATAACAAGCATATAAACTGGGTTAGTGTT
TATAATTTCACAACTCCTTATGTGTCCCAAAATCCAAGATTGTATCTCAATTATAGGGACCTTGATTTAGGAA
AAACTAATCCTGAGAGTCCTAATAATTACACACAAGCACGTATTTGGGGTGAAAAGTATTTTGGTAAAAATTT
TAACAGGTTAGTTAAGGTGAAAACCAAAGCTGATCCCAATAATTTTAGAAACGAACAAAGTATCCCACCTCTT
CCACATCGTCATCATTAA
>A3_Chemdog91_LKUB01150367.1_3
AAAAAGAACATGGAGATACATGGGCTTGTCAAGTTATTTAACAAATGGCAAAATATTGCTTACAAGTATGACA
AAGATTTATTACTCATGACTCACTTCATAACCAGGAATATTATAGATAATCAAGGAAAGAATAAGACTACAGT

ACACGGTTACTTCTCTTGCATTTTCCATGGTGGAGTAGATAGTCTAGTCAACTTGATGAACAAGAGCTTTCCT
GAGTTGGGTATTAAAAAAACTGATTGCAAAGAATTGAGCTGGATTGATACAACCATCTTCTACAGTGGTGTTG
TAAATTATAACACTACTAATTTTCAAAAGGAAATTTTGCTTGATAGATCAGCTGGGCAGAAAGTAGCTTTCTC
AGTTAAGTTAGACTACGTTAAGAAACCAATTCCAGAAACTGCAATTGTCAAAATTTTGGAGAAATTGTATGAA
GAAGATGTAGGAGTTGGGGTGTATGTATTGTACCCTTACGGTGGTATAATGGACAAGATCTCAGAATCAACAA
TTCCTTTCCCTCATCGAGCTGGAATCATGTACGAAGTTTGATACGCAGCTACCTGGGAGAAGCAAGAAGATAA
TGAAAAGCATATAAACTGGGTTTGAAGTGTTTATAATTTCATGACGCCTTATGTGTCCCAAAATCCAAGAATG
GCGTATCTCAATTATAGGGACCTTGATTTAGGAAAAACTGATCCCAAGAGTCCTAATAATTACACCCAAGCAC
GTATCTGGGGTGAAAAGTACTTTGGTAAAAACTTTGACAAGTTAGTTAAGGTGAAAACCAAAGTTGATCCCAA
TAATTTTTTTAGAAACGAGCAAAGCATCCCACCTCTTCCGCCACGACGTCATTAA
>A3_LAconfidential_LKUA01025043.1_3322
ATGAAGTACTACTCAACATTCTCCTTTAGGTTTGTTTACAAAATTATATTTTTCTTTCTCTCATTCAATATCA
AAATTTCAATAGCTAATCCTCAAGAAAATTTCCTAAAATGCTTCTCCCAATATATTCATAACAATCCAGCAAA
TCTAAAACTCGTATACACTCAACACGACCAATTGTATATGTCTGTCCTGAATTTGACAATACAAAATCTTAGA
TTTACCTCTGATACAACCCCAAAACCACTCGTTATTGTCACTCCTTCAAATGTCTCCCATATCCAAGCCACTA
TTCTATGCTCCAAGAAAGTGGGCTTGCAAATTCGAACTCGAAGCGGTGGCCATGATGCTGAGGGTTTGTCCTA
CACATCTCAAGTCCCATTTGTTATAGTAGACTTGAGAAACATGCATTCGGTGAAAATAGATATTCGTAGCCAA
ATTGCGTGGGTTGAAGCCGGAGCTACCCTTGGAGAAGTTTATTATTGGATTAATGAGAATCTTAGTTTTCCTG
GTGGGTATTGCCCTACTGTTGGCGTAGGTGGACACTTTAGTGGAGGAGGCTATAGAGCATTAATGCGAAATTA
TGGCCTCGCAGCTGATAATATCATTGATGCACACTTAGTCAATGTTGATGGAAAAGTTCTAGATCGAAAATCC
ATGGGGGAAGATCTATTTTGGGCTATACGTGGTGGTGGAGGTGAAAACTTTGGAATCATTGCAGCGTGGAAAA
TTAGACTGGTTGCTGTCCCATCAAGGGCTACTATATTCAGTGTTAAAAGGAATATGGAGATACATGGGCTTGT
CAAGTTATTTAATAAATGGCAAAATATTGCTTACAAGTATGACAAAGATTTATTACTCATGACTCACTTCATA
ACCAGGAATATTATAGATAATCAAGGAAAGAATAAGACTACAGTACACGGTTACTTCTCTTGCATTTTCCATG
GTGGAGTAGATAGTCTAGTCAACTTGATGAACAAGAGCTTTCCTGAGTTGGGTATTAAAAAAACTGATTGCAA
AGAATTGAGCTGGATTGATACTACCATCTTCTACAGTGGTGTTGTAAATTATAACACTACTAATTTTCAAAAG
GAAATTTTGCTTGATAGATCAGCTGGGCAGAAAGTAGCTTTCTCAGTTAAGTTAGACTACGTTAAGAAACCAA
TTCCAGAAACTGCAATTGTCAAAATTTTGGAGAAATTGTATGAAGAAGATGTAGGAGTTGGGGTGTATGTATT
GTACCCTTACGGTGGTATAATGGACAAGATCTCAGAATCAACAATTCCTTTCCCTCATCGAGCTGGAATCATG
TACGAAGTTTGATACGCAGCTACCTGGGAGAAGCAAGAAGATAATGAAAAGCATATAAACTGGGTTTGAAGTG
TTTATAATTTCATGACGCCTTATGTGTCCCAAAATCCAAGAATGGCGTATCTCAATTATAGGGACCTTGATTT
AGGAAAAACTGATCCCAAGAGTCCTAATAATTACACCCAAGCACGTATCTGGGGTGAAAAGTACTTTGGTAAA
AACTTTGACAAGTTAGTTAAGGTGAAAACCAAAGTTGATCCCAATAATTTTTTTAGAAACGAGCAAAGCATCC
CACCTCTTCCGCCACGACGTCATTAA
>A3_Jilong_CM022965.1_92151528
AAGTACTACTCAACATTCTCCTTTAGGTTTGTTTACAAAATTATATTTTTCTTTCTCTCATTCAATATCAAAA
TTTCAATAGCTAATCCTCAAGAAAATTTCCTAAAATGCTTCTCCCAATATATTCATAACAATCCAGCAAATCT
AAAGCTCGTATACACTCAACACGACCAATTGTATATGTCTGTCCTGAATTTGACAATACAAAATCTTAGATTT
ACCTCTGATACAACCCCAAAACCACTCGTTATTGTCACTCCTTCAAATGTCTCCCATATCCAAGCCACTATTC
TATGCTCCAAGAAAGTGGGCTTGCAAATTCGAACTCGAAGCGGTGGCCATGATGCTGAGGGTTTGTCCTACAC
ATCTCAAGTCCCATTTGTTATAGTAGACTTGAGAAACATGCATTCGGTGAAAATAGATATTCGTAGCCAAATT
GCGTGGGTTGAAGCCGGAGCTACCCTTGGAGAAGTTTATTATTGGATTAATGAGAATCTTAGTTTTCCTGGTG
GGTATTGCCCTACTGTTGGCGTAGGTGGACACTTTAGTGGAGGAGGCTATAGAGCATTAATGCGAAATTATGG
CCTCGCAGCTGATAATATCATTGATGCACACTTAGTCAATGTTGATGGAAAAGTTCTAGATCGAAAATCCATG
GGGGAAGATCTATTTTGGGCTATACGTGGTGGTGGAGGTGAAAACTTTGGAATCATTGCAGCGTGGAAAATTA
GACTGGTTGCTGTCCCATCAAGGGCTACTATATTCAGTGTTAAAAGGAATATGGAGATACATGGGCTTGTCAA
GTTATTTAATAAATGGCAAAATATTGCTTACAAGTATGACAAAGATTTATTACTCATGACTCACTTCATAACC
AGGAATATTATAGATAATCAAGGAAAGAATAAGACTACAGTACACGGTTACTTCTCTTGCATTTTCCATGGTG
GAGTAGATAGTCTAGTCAACTTGATGAACAAGAGCTTTCCTGAGTTGGGTATTAAAAAAACTGATTGCAAAGA
ATTGAGCTGGTTTGATACTACCATCTTCTACAGTGGTGTTGTAAATTATAACACTACTAATTTTCAAAAGGAA
ATTTTGCTTGATAGATCAGCTGGGCAGAAAGTAGCTTTCTCAGTTAAGTTAGACTACGTTAAGAAACCAATTC
CAGAAACTGCAATTGTCAAAATTTTGGAGAAATTGTATGAAGAAGATGTAGGAGTTGGGGTGTATGTATTGTA
CCCTTACGGTGGTATAATGGACAAGATCTCAGAATCAACAATTCCTTTCCCTCATCGAGCTGGAATCATGTAC
GAAGTTTGATACGCAGCTACCTGGGAGAAGCAAGAAGATAATGAAAAGCATATAAACTGGGTTTGAAGTGTTT
ATAATTTCATGACGCCTTATGTGTCCCAAAATCCAAGAATGGCGTATCTCAATTATAGGGACCTTGATTTAGG
AAAAACTGATCCCAAGAGTCCTAATAATTACACCCAAGCACGTATCTGGGGTGAAAAGTACTTTGGTAAAAAC
TTTGACAAGTTAGTTAAGGTGAAAACCAAAGTTGATCCCAATAATTTTTTTAGAAACGAGCAAAGCATCCCAC
CTCTTCCGCCACGACGTCATTAA

>A3_Jilong_CM022965.1_89697636
AAGTACTACTCAACATTCTCCTTTAGGTTTGTTTACAAAATTATATTTTTCTTTCTCTCATTCAATATCAAAA
TTTCAATAGCTAATCCTCAAGAAAATTTCCTAAAATGCTTCTCCCAATATATTCATAACAATCCAGCAAATCT
AAAGCTCGTATACACTCAACACGACCAATTGTATATGTCTGTCCTGAATTTGACAATACAAAATCTTAGATTT
ACCTCTGATACAACCCCAAAACCACTCGTTATTGTCACTCCTTCAAATGTCTCCCATATCCAAGCCACTATTC
TATGCTCCAAGAAAGTGGGCTTGCAAATTCGAACTCGAAGCGGTGGCCATGATGCTGAGGGTTTGTCCTACAC
ATCTCAAGTCCCATTTGTTATAGTAGACTTGAGAAACATGCATTCGGTGAAAATAGATATTCGTAGCCAAATT
GCGTGGGTTGAAGCCGGAGCTACCCTTGGAGAAGTTTATTATTGGATTAATGAGAATCTTAGTTTTCCTGGTG
GGTATTGCCCTACTGTTGGCGTAGGTGGACACTTTAGTGGAGGAGGCTATAGAGCATTAATGCGAAATTATGG
CCTCGCAGCTGATAATATCATTGATGCACACTTAGTCAATGTTGATGGAAAAGTTCTAGATCGAAAATCCATG
GGGGAAGATCTATTTTGGGCTATACGTGGTGGTGGAGGTGAAAACTTTGGAATCATTGCAGCGTGGAAAATTA
GACTGGTTGCTGTCCCATCAAGGGCTACTATATTCAGTGTTAAAAGGAATATGGAGATACATGGGCTTGTCAA
GTTATTTAATAAATGGCAAAATATTGCTTACAAGTATGACAAAGATTTATTACTCATGACTCACTTCATAACC
AGGAATATTATAGATAATCAAGGAAAGAATAAGACTACAGTACACGGTTACTTCTCTTGCATTTTCCATGGTG
GAGTAGATAGTCTAGTCAACTTGATGAACAAGAGCTTTCCTGAGTTGGGTATTAAAAAAACTGATTGCAAAGA
ATTGAGCTGGTTTGATACTACCATCTTCTACAGTGGTGTTGTAAATTATAACACTACTAATTTTCAAAAGGAA
ATTTTGCTTGATAGATCAGCTGGGCAGAAAGTAGCTTTCTCAGTTAAGTTAGACTACGTTAAGAAACCAATTC
CAGAAACTGCAATTGTCAAAATTTTGGAGAAATTGTATGAAGAAGATGTAGGAGTTGGGGTGTATGTATTGTA
CCCTTACGGTGGTATAATGGACAAGATCTCAGAATCAACAATTCCTTTCCCTCATCGAGCTGGAATCATGTAC
GAAGTTTGATACGCAGCTACCTGGGAGAAGCAAGAAGATAATGAAAAGCATATAAACTGGGTTTGAAGTGTTT
ATAATTTCATGACGCCTTATGTGTCCCAAAATCCAAGAATGGCGTATCTCAATTATAGGGACCTTGATTTAGG
AAAAACTGATCCCAAGAGTCCTAATAATTACACCCAAGCACGTATCTGGGGTGAAAAGTACTTTGGTAAAAAC
TTTGACAAGTTAGTTAAGGTGAAAACCAAAGTTGATCCCAATAATTTTTTTAGAAACGAGCAAAGCATCCCAC
CTCTTCCGCCACGACGTCATTAA
>A3_Jamaican_Lion_JAATIP010000026.1_F8388_024907
ATGAAGTACTACTCAACATTCTCCTTTAGGTTTGTTTACAAAATTATATTTTTCTTTCTCTCATTCAATATCA
AAATTTCAATAGCTAATCCTCAAGAAAATTTCCTAAAATGCTTCTCCCAATATATTCATAACAATCCAGCAAA
TCTAAAACTCGTATACACTCAACACGACCAATTGTATATGTCTGTCCTGAATTTGACAATACAAAATCTTAGA
TTTACCTCTGATACAACCCCAAAACCACTCGTTATTGTCACTCCTTCAAATGTCTCCCATATCCAAGCCACTA
TTCTATGCTCCAAGAAAGTGGGCTTGCAAATTCGAACTCGAAGCGGTGGCCATGATGCTGAGGGTTTGTCCTA
CACATCTCAAGTCCCATTTGTTATAGTAGACTTGAGAAACATGCATTCGGTGAAAATAGATATTCGTAGCCAA
ATTGCGTGGGTTGAAGCCGGAGCTACCCTTGGAGAAGTTTATTATTGGATTAATGAGAATCTTAGTTTTCCTG
GTGGGTATTGCCCTACTGTTGGCGTAGGTGGACACTTTAGTGGAGGAGGCTATAGAGCATTAATGCGAAATTA
TGGCCTCGCAGCTGATAATATCATTGATGCACACTTAGTCAATGTTGATGGAAAAGTTCTAGATCGAAAATCC
ATGGGGGAAGATCTATTTTGGGCTATACGTGGTGGTGGAGGTGAAAACTTTGGAATCATTGCAGCGTGGAAAA
TTAGACTGGTTGCTGTCCCATCAAGGGCTACTATATTCAGTGTTAAAAGGAATATGGAGATACATGGGCTTGT
CAAGTTATTTAATAAATGGCAAAATATTGCTTACAAGTATGACAAAGATTTATTACTCATGACTCACTTCATA
ACCAGGAATATTATAGATAATCAAGGAAAGAATAAGACTACAGTACACGGTTACTTCTCTTGCATTTTCCATG
GTGGAGTAGATAGTCTAGTCAACTTGATGAACAAGAGCTTTCCTGAGTTGGGTATTAAAAAAACTGATTGCAA
AGAATTGAGCTGGATTGATACTACCATCTTCTACAGTGGTGTTGTAAATTATAACACTACTAATTTTCAAAAG
GAAATTTTGCTTGATAGATCAGCTGGGCAGAAAGTAGCTTTCTCAGTTAAGTTAGACTACGTTAAGAAACCAA
TTCCAGAAACTGCAATTGTCAAAATTTTGGAGAAATTGTATGAAGAAGATGTAGGAGTTGGGGTGTATGTATT
GTACCCTTACGGTGGTATAATGGACAAGATCTCAGAATCAACAATTCCTTTCCCTCATCGAGCTGGAATCATG
TACGAAGTTTGATACGCAGCTACCTGGGAGAAGCAAGAAGATAATGAAAAGCATATAAACTGGGTTTGAAGTG
TTTATAATTTCATGACGCCTTATGTGTCCCAAAATCCAAGAATGGCGTATCTCAATTATAGGGACCTTGATTT
AGGAAAAACTGATCCCAAGAGTCCTAATAATTACACCCAAGCACGTATCTGGGGTGAAAAGTACTTTGGTAAA
AACTTTGACAAGTTAGTTAAGGTGAAAACCAAAGTTGATCCCAATAATTTTTTTAGAAACGAGCAAAGCATCC
CACCTCTTCCGCCACGACGTCATTAA
>A3_Purple_Kush__AGQN03001586.1_37412
ATGAAGTACTACTCAACATTCTCCTTTAGGTTTGTTTACAAAATTATATTTTTCTTTCTCTCATTCAATATCA
AAATTTCAATAGCTAATCCTCAAGAAAATTTCCTAAAATGCTTCTCCCAATATATTCATAACAATCCAGCAAA
TCTAAAACTCGTATACACTCAACACGACCAATTGTATATGTCTGTCCTGAATTTGACAATACAAAATCTTAGA
TTTACCTCTGATACAACCCCAAAACCACTCGTTATTGTCACTCCTTCAAATGTCTCCCATATCCAAGCCACTA
TTCTATGCTCCAAGAAAGTGGGCTTGCAAATTCGAACTCGAAGCGGTGGCCATGATGCTGAGGGTTTGTCCTA
CACATCTCAAGTCCCATTTGTTATAGTAGACTTGAGAAACATGCATTCGGTGAAAATAGATATTCGTAGCCAA
ATTGCGTGGGTTGAAGCCGGAGCTACCCTTGGAGAAGTTTATTATTGGATTAATGAGAATCTTAGTTTTCCTG
GTGGGTATTGCCCTACTGTTGGCGTAGGTGGACACTTTAGTGGAGGAGGCTATAGAGCATTAATGCGAAATTA
TGGCCTCGCAGCTGATAATATCATTGATGCACACTTAGTCAATGTTGATGGAAAAGTTCTAGATCGAAAATCC

ATGGGGGAAGATCTATTTTGGGCTATACGTGGTGGTGGAGGTGAAAACTTTGGAATCATTGCAGCGTGGAAAA
TTAGACTGGTTGCTGTCCCATCAAGGGCTACTATATTCAGTGTTAAAAGGAATATGGAGATACATGGGCTTGT
CAAGTTATTTAATAAATGGCAAAATATTGCTTACAAGTATGACAAAGATTTATTACTCATGACTCACTTCATA
ACCAGGAATATTATAGATAATCAAGGAAAGAATAAGACTACAGTACACGGTTACTTCTCTTGCATTTTCCATG
GTGGAGTAGATAGTCTAGTCAACTTGATGAACAAGAGCTTTCCTGAGTTGGGTATTAAAAAAACTGATTGCAA
AGAATTGAGCTGGATTGATACTACCATCTTCTACAGTGGTGTTGTAAATTATAACACTACTAATTTTCAAAAG
GAAATTTTGCTTGATAGATCAGCTGGGCAGAAAGTAGCTTTCTCAGTTAAGTTAGACTACGTTAAGAAACCAA
TTCCAGAAACTGCAATTGTCAAAATTTTGGAGAAATTGTATGAAGAAGATGTAGGAGTTGGGGTGTATGTATT
GTACCCTTACGGTGGTATAATGGACAAGATCTCAGAATCAACAATTCCTTTCCCTCATCGAGCTGGAATCATG
TACGAAGTTTGATACGCAGCTACCTGGGAGAAGCAAGAAGATAATGAAAAGCATATAAACTGGGTTTGAAGTG
TTTATAATTTCATGACGCCTTATGTGTCCCAAAATCCAAGAATGGCGTATCTCAATTATAGGGACCTTGATTT
AGGAAAAACTGATCCCAAGAGTCCTAATAATTACACCCAAGCACGTATCTGGGGTGAAAAGTACTTTGGTAAA
AACTTTGACAAGTTAGTTAAGGTGAAAACCAAAGTTGATCCCAATAATTTTTTAGAAACGAGCAAAGCATCC
CACCTCTTCCGCCACGACGTCATTAA
>A3_Finola_CM011610.1_22244169
ATGAAGTACTACTCAACATTCTCCTTTAGGTTTGTTTACAAAATTATATTTTTCTTTCTCTCATTCAATATCA
AAATTTCAATAGCTAATCCTCAAGAAAATTTCCTAAATTGCTTCTCCCAATATATTCATAACAATCCAGCAAA
TCTAAAACTCGTATACACTCAACACGACCAATTGTATATGTCTGTCCTGAATTTGACAATACAAAATCTTAGA
TTTACCTCTGATACAACCCCAAAACCACTCGTTATTGTCACTCCTTCAAATGTCTCCCATATCCAAGCCACTA
TTCTATGCTCCAAGAAAGTTGGCTTGCAGATTCGAACTCGAAGCGGTGGCCATGATGCTGAGGGTTTGTCCTA
CACATCTCAAGTCCCATTTGTTATAGTAGACTTGAGAAACATGCATTCGGTGAAAATAGATATTCGTAGCCAA
ACTGCGTGGGTTGAAGCCGGAGCTACCCTTGGAGAAGTTTATTATTGGATTAATGAGAAGAATGAGAATCTTA
GTTTTCCTGGTGGGTATTGCCCTACTGTTGGCGTAGGTGGACACTTTAGTGGAGGAGGCTATGGAGCATTAAT
GCGAAATTATGGCCTCGCAGCTGATAATATCATTGATGCACACTTAGTCAATGTTGATGGAAAAGTTCTAGAT
CGAAAATCCATGGGGGAAGATCTATTTTGGGCTATACGTGGTGGTGGAGGTGAAAACTTTGGAATCATTGCAG
CGTGGAAAATTAGACTGGTTGCTGTCCCATCAAGGGCTACTATATTCAGTGTTAAAAGGAATATGGAGATACA
TGGGCTTGTCAAGTTATTTAACAAATGGCAAAATATTGCTTACAAGTATGACAAAGATTTATTACTCATGACT
CACTTCATAACCAGGAATATTATAGATAATCAAGGAAAGAATAAGACTACAGTACACGGTTACTTCTCTTGCA
TTTTCCATGGTGGAGTGGATAGTCTAGTCAACTTGATGAACAAGAGCTTTCCTGAGTTGGGTATTAAAAAAAC
TGATTGCAAAGAATTGAGCTGGATTGATACTACCATCTTCTACAGTGGTGTTGTAAATTATAACACTACTAAT
TTTCAAAAGGAAATTTTGCTTGATAGATCAGCTGGGCAGAAAGTAGCTTTCTCAATTAAGTTAGACTACGTTA
AGAAACCAATTCCAGAAACTGCAATTGTCAAAATTTTGGAGAAATTGTATGAAGAAGATGTAGGAGTTGGAGT
GTATGTATTGTACCCTTACGGTGGTATAATGGACAAGATCTCAGAATCAACAATTCCTTTCCCTCATCGAGCT
GGAATCATGTACGAAGTTTGGTACGCAGCTACCTGGGAGAAGCAAGAAGATAATGAAAAGCATATAAACTGGG
TTCGAAGTGTTTATAATTTCATGACGCCTTATGTGTCCCAAAATCCAAGAATGGCGTATCTCAATTATAGGGA
CCTTGATTTAGGAAAAACTGATCCCAAGAGTCCTAATAATTACACCCAAGCACGTATCTGGGGTGAAAAGTAC
TTTGGTAAAAACTTTGACAAGTTAGTTAAGGTGAAAACCAAAGTTGATCCCAATAATTTTTTAGAAACGAGC
AAAGCATCCCACCTCTTCCGCCACGACGTCATTAA
>A4_Finola_QKVJ02000019.1_876094
CTTAGATTCAACTCTGATGCAACCCCAAAACCACTCGTTATCGTCACTCCTTCAAATGTCTCCCACATCCAAG
CCCCAATTCTATGCTCCAAGAAAGTTGACTTGTAGATTCGAACTTGAAGCGGTAGCCACGATGCTGAGGGTTT
GTCCTACATATCTCAAGTCCCATTCGTTATAGTAGACTTGAGAAACATACATTTGATCAAAATAGATGTTCGT
AGCCAAACTGCATGGGTTGAAGCTGGAGCTACCCTTGGAGAAGTTTACTTCCGGATCAATGAGAAGAATGAGA
ATCTTAATTTTCCTGGTGGGTTTTGCCCTACTGTTGGCGCAGGTGGACACTTTAGTGGAGGAGGCTATGGATC
ATTGATACGAAATTATGGCCTCGCGGCTAATAATATCATTGATGCACACATAGTCAATGTTGATGGAAAGTTT
CTAGATCGAAAATCCATGGGAGAAGATATGTTTTGGGCTATACGTGGTGGTGGAAGAAACTTTGGAATAATTA
CACTGTGGAAAATCAAACTGGTTGATGTCATGTCAAAGTCTACTATATTCAGTGTTAAAAAGAATATGGAGAT
ACATGGGCTTGTCAAGTTATTTGATAAATGGCAAAATATTGCTTACAAGTATGACAAAGATTTATTACTCATG
ACTCACTTCATAACCAAAAATACTACAGATAATCAAGGAAAGAATAAAACAACAGTACACGGTTACTTCCCTT
CCATTTTCCATGGTGGAGCGGATAGTTTGGTTGACTTGATGAACAAGAGCTTTCCTGAGTTGGGTATTAAAAA
AACTGATTGCAAAGAATTGAGCTGAATTGATACCACCATCTTCTACAGTGGAATTGTAAATAACACTGCTAAT
TTTAAAAAGGAAATTTTGCTTGATCGATCAGCTGGGCCGAAGACAGCTTTCTCAATTAAGTTAGACTACGTTA
AGCACCCAATTCCGAAAATCGCAATGGTCAAAATTTTGGAAAAATTATATGAAGAAGATGTAGGAGTTGGGAT
GTATCTTTTGATCCCGTACGGCGGTACAATGGATGAGATTTCAGAATCAGCAATTCCATTCCGTCATCGAGCT
GGAATCATGTATGAACTTTGGTACGCAGCTACCTAGGAAAAGCAAGAAGATAATCAAAAGCATATAAATTGGG
TTCGAAGTGTTTATAATTTCACAACTCCTTATGTGTCCTAAAATCAAAGATTCGCATATCTCAATTATAGGGA
CCTTGATTTAGGAAAAACTAATCCCAGGAGTCCTAATAATTACACACAAGCATGTATTTGGGGTGAAAAGTAT

TTTAGTAAAAATTTTAACAGGTTTGTTAAGGTGAAAACCAAGGTTGATCCCAATAATTTTTTTAGAAACGAAC
AAAGCATCCCACCTCTTCCACCGCGTCGTCCTTAA
>A4_Jamaican_Lion_JAATIP010000103.1
CTTAGATTCAACTCTGATGCAACCCCAAAACCACTCGTTATCGTCACTCCTTCAAATGTCTCCCACATCCAAG
CCCCAATTCTATGCTCCAAGAAAGTTGACTTGTAGATTCGAACTTGAAGCGGTAGCCACGATGCTGAGGGTTT
GTCCTACATATCTCAAGTCCCATTCGTTATAGTAGACTTGAGAAACATACATTTGATCAAAATAGATGTTCGT
AGCCAAACTGCATGGGTTGAAGCTGGAGCTACCCTTGGAGAAGTTTACTTCCGGATCAATGAGAAGAATGAGA
ATCTTAATTTTCCTGGTGGGTTTTGCCCTACTGTTGGCACAGGTGGACACTTTAGTGGAGGAGGCTATGGATC
ATTGATACGAAATTATGGCCTCGCGGCTAATAATATCATTGATGCACACATAGTCAATGTTGATGGAAAGTTT
CTAGATCGAAAATCCATGGGAGAAGATATGTTTTGGGCTATACGTGGTGGTGGAAGAAACTTTGGAATAATTA
CACTGTGGAAAATCAGACTGGTTGATGTCATGTCAAAGTCTACTATATTCAGTGTTAAAAAGAATATGGAGAT
ACATGGGCTTGTCAAGTTATTTGATAAATGGCAAAATATTGCTTACAAGTATGACAAAGATTTATTACTCATG
ACTCACTTCATAACCAAAAATACTACAGATAATCAAGGAAAGAATAAAACAACAGTACACGGTTACTTCCCTT
CCATTTTCCATGGTGGAGCGGATAGTTTGGTTGACTTGATGAACAAGAGCTTTCCTGAGTTGGGTATTAAAAA
AACTGATTGCAAAGAATTGAGCTGAATTGATACCACCATCTTCTACAGTGGAATTGTAAATAACACTGCTAAT
TTTAAAAAGGAAATTTTGCTTGATCGATCAGCTGGGCCGAAGACAGCTTTCTCAATTAAGTTAGACTACGTTA
AGCACCCAATTCCGAAAATCGCAATGGTCAAAATTTTGGAAAAATTATATGAAGAAGATGTAGGAGTTGGGAT
GTATCTTTTGATCCCGTACGGCGGTACAATGGATGAGATTTCAGAATCAGCAATTCCATTCCGTCATCGAGCT
GGAATCATGTATGAACTTTGGTACGCAGCTACCTAGGAAAAGCAAGAAGATAATCAAAAGCATATAAATTGGG
TTCGAAGTGTTTATAATTTCACAACTCCTTATGTGTCCTAAAATCAAAGATTCGCATATCTCAATTATAGGGA
CCTTGATTTAGGAAAAACTAATCCCAGGAGTCCTAATAATTACACACAAGCATGTATTTGGGGTGAAAAGTAT
TTTAGTAAAAATTTTAACAGGTTTGTTAAGGTGAAAACCAAGGTTGATCCCAATAATTTTTTTAGAAACGAAC
AAAGCATCCCACCTCTTCCACCGCGTCGTCCTTAA
>A4_CBDRx_NC_044378.1_25870545
ATGAATTGCTCAACATTCTCCTTTTGGTTTGTTTGCAAAATAATATTTTTCTTTCTCTCATTCAATATCCAAA
TTTCAATAGCTAATCCTCAAGAAAACTTCCTTAAATGCTTCTCGGAATATATTCCTAACAATCCAAGAAATCC
AAAATTCATATACACTCAACACGACCAATTGTATATATGTCTCTTCCGATTGACAATACAAAATCTTAGATTC
AACTCTGATGCAACCCCAAAACCACTCGTTATCGTCACTCCTTCAAATGTCTCCCACATCCAAGCCCCAATTC
TATGCTCCAAGAAAGTTGACTTGTAGATTCGAACTTGAAGCGGTAGCCACGATGCTGAGGGTTTGTCCTACAT
ATCTCAAGTCCCATTCGTTATAGTAGACTTGAGAAACATACATTTGATCAAAATAGATGTTCGTAGCCAAACT
GCATGGGTTGAAGCTGGAGCTACCCTTGGAGAAGTTTACTTCCGGATCAATGAGAAGAATGAGAATCTTAATT
TTCCTGGTGGGTTTTGCCCTACTGTTGGCACAGGTGGACACTTTAGTGGAGGAGGCTATGGATCATTGATACG
AAATTATGGCCTCGCGGCTAATAATATCATTGATGCACACATAGTCAATGTTGATGGAAAGTTTCTAGATCGA
AAATCCATGGGAGAAGATATGTTTTGGGCTATACGTGGTGGTGGAAGAAACTTTGGAATAATTACACTGTGGA
AAATCAGACTGGTTGATGTCATGTCAAAGTCTACTATATTCAGTGTTAAAAAGAATATGGAGATACATGGGCT
TGTCAAGTTATTTGATAAATGGCAAAATATTGCTTACAAGTATGACAAAGATTTATTACTCATGACTCACTTC
ATAACCAAAAATACTACAGATAATCAAGGAAAGAATAAAACAACAGTACACGGTTACTTCCCTTCCATTTTCC
ATGGTGGAGCGGATAGTTTGGTTGACTTGATGAACAAGAGCTTTCCTGAGTTGGGTATTAAAAAAACTGATTG
CAAAGAATTGAGCTGAATTGATACCACCATCTTCTACAGTGGAATTGTAAATAACACTGCTAATTTTAAAAAG
GAAATTTTGCTTGATCGATCAGCTGGGCCGAAGACAGCTTTCTCAATTAAGTTAGACTACGTTAAGCACCCAA
TTCCGAAAATCGCAATGGTCAAAATTTTGGAAAAATTATATGAAGAAGATGTAGGAGTTGGGATGTATCTTTT
GATCCCGTACGGCGGTACAATGGATGAGATTTCAGAATCAGCAATTCCATTCCGTCATCGAGCTGGAATCATG
TATGAACTTTGGTACGCAGCTACCTAGGAAAAGCAAGAAGATAATCAAAAGCATATAAATTGGGTTCGAAGTG
TTTATAATTTCACAACTCCTTATGTGTCCTAAAATCAAAGATTCGCATATCTCAATTATAGGGACCTTGATTT
AGGAAAAACTAATCCCAGGAGTCCTAATAATTACACACAAGCATGTATTTGGGGTGAAAAGTATTTTAGTAAA
AATTTTAACAGGTTTGTTAAGGTGAAAACCAAAGCTGATCCCAATAATTTTTTTAGAAATGAACAAAGTATCC
CACCTCTTCCACCGCGTCATCATTAA
>A4_Jamaican_Lion_JAATIP010000026.1_F8388_024889
ATGAAGTACTCAACATTTTCCTTTTGGTTTGTTTGCAAAATAATATTTTTCCTCTCATTCAATATCCAAATTT
CCATAGCTAATCCTCGAGAAAATTTCCTTAAATGCTTCTCGCAACATATTCCCAACAATGCAGCAAATCCAAA
ACTCGTATACACTCATAATGACCACTTGTATATGTCTGTCCTGAATTCAAAAATACAAAATCTTAGATTCAAC
TCTGATGCAACCCCAAAACCACTCGTTATCGTCACTCCTTCAAATGTCTCCCACATCCAAGCCCCAATTCTAT
GCTCCAAGAAAGTTGACTTGTAGATTCGAACTTGAAGCGGTAGCCACGATGCTGAGGGTTTGTCCTACATATC
TCAAGTCCCATTCATTATAGTAGACTTGAGAAACATACATTTGATCAAAATAGATGTTCGTAGCCAAACTGCA
TGGGTTGAAGCTGGAGCTACCCTTGGAGAAGTTTACTTCCAGATCAATGAGAAGAATGAGAATCTTAGTTTTC
CTGGTGGGTTTTGCCCTACTGTTGGCGCAGGTGGACACTTTAGTGGAGGAGGCTATGGAGCATTGATACGAAA
TTATGGCCTCGCGGCTAATAATATCATTGATGCACACATAGTCAATGTTGATGGAAAGTTTCTAGATCGAAAA
TCCATGAGAGAAGATATGTTTTGGGCTATACGTGGTGGTGGAAGAAACTTTGGAATAATTACAGCGTGGAAAA

TCAGACTGGTTGATGTCCTGTCAAAGTCTACTATATTCAGTGTTAAAAAGAATATGGAGATACATGGGCTTGT
CAAGTTATTTGATAAATGGCAAAATATTGCTTACAAGTATGACAAAGATTTATTACTCATGACTCACTTCATA
ACCAAAAATACTACAGATAATCAAGGAAAGAATAAAACAACAGTACACGATTACTTCCCTTCCATTTTCCATG
GTGGAGCGGATAGTTTGGTTGACTTGATGAACAAGAGCTTTCCTGAGTTGGGTATTAAAAAAACTGATTGCAA
AGAATTGAGCTGAATTGATACCACCATCTTCTATAGTTGAATTGTAAATAACACTGCTAATTTTAAAAAGGAA
ATTTTGCTTGATCGATCAGCTGGGCCGAAGACAGCTTTCTCAATTAAGTTAGACTACGTTAAGAACCCAATTC
TGAAAATCGCAATGGTCAAAATTTTGGAAAAATTATATGAAGAAGATGTAGGAGTTGGGATGTATCTTTTGAT
CCCTTACGGCGGTACAATGGATGAGATTTCAGAATCAGCAATTCCATTCCGTCATCGAGCTGGAATCATGTAT
GAACTTTGGTACGCAGCTACCTAGGAAAAGGAAGAAGATAATCAAAAGCATATAAATTGGGTTCGAAGTGTTT
ATAATTTCACAACTCCTTATGTGTCCCAAAATCAAAGAATCGCATATGTCAATTATAGGGACCTTGATTTAGG
AAAAACTAATCCCAAGAGTCCTAATAATTACACACAAGCATGTATTTGGGGTGAAAAGTATTTTAGTAAAAAT
TTTAACAGGTTTGTTAAGGTGAAAACCAAGGTTGATCCCAATAATTTTTTTAGAAACGAACAAAGCATCCCAC
CTCTTCCACCGCGTCGTCCTTAA
>A4_Purple_Kush__CM010793.2_49189801
ATGAAGTACTCAACATTTTCCTTTTGGTTTGTTTGCAAAATAATATTTTTCCTCTCATTCAATATCCAAATTT
CCATAGCTAATCCTCGAGAAAATTTCCTTAAATGCTTCTCGCAACATATTCCCAACAATGCAGCAAATCCAAA
ACTCGTATACACTCATAATGACCACTTGTATATGTCTGTCCTGAATTCAAAAATACAAAATCTTAGATTCAAC
TCTGATGCAACCCCAAAACCACTCGTTATCGTCACTCCTTCAAATGTCTCCCACATCCAAGCCCCAATTCTAT
GCTCCAAGAAAGTTGACTTGTAGATTCGAACTTGAAGCGGTAGCCACGATGCTGAGGGTTTGTCCTACATATC
TCAAGTCCCATTCATTATAGTAGACTTGAGAAACATACATTTGATCAAAATAGATGTTCGTAGCCAAACTGCA
TGGGTTGAAGCTGGAGCTACCCTTGGAGAAGTTTACTTCCAGATCAATGAGAAGAATGAGAATCTTAGTTTTC
CTGGTGGGTTTTGCCCTACTGTTGGCGCAGGTGGACACTTTAGTGGAGGAGGCTATGGAGCATTGATACGAAA
TTATGGCCTCGCGGCTAATAATATCATTGATGCACACATAGTCAATGTTGATGGAAAGTTTCTAGATCGAAAA
TCCATGAGAGAAGATATGTTTTGGGCTATACGTGGTGGTGGAAGAAACTTTGGAATAATTACAGCGTGGAAAA
TCAGACTGGTTGATGTCCTGTCAAAGTCTACTATATTCAGTGTTAAAAAGAATATGGAGATACATGGGCTTGT
CAAGTTATTTGATAAATGGCAAAATATTGCTTACAAGTATGACAAAGATTTATTACTCATGACTCACTTCATA
ACCAAAAATACTACAGATAATCAAGGAAAGAATAAAACAACAGTACACGATTACTTCCCTTCCATTTTCCATG
GTGGAGCGGATAGTTTGGTTGACTTGATGAACAAGAGCTTTCCTGAGTTGGGTATTAAAAAAACTGATTGCAA
AGAATTGAGCTGAATTGATACCACCATCTTCTATAGTTGAATTGTAAATAACACTGCTAATTTTAAAAAGGAA
ATTTTGCTTGATCGATCAGCTGGGCCGAAGACAGCTTTCTCAATTAAGTTAGACTACGTTAAGAACCCAATTC
TGAAAATCGCAATGGTCAAAATTTTGGAAAAATTATATGAAGAAGATGTAGGAGTTGGGATGTATCTTTTGAT
CCCTTACGGCGGTACAATGGATGAGATTTCAGAATCAGCAATTCCATTCCGTCATCGAGCTGGAATCATGTAT
GAACTTTGGTACGCAGCTACCTAGGAAAAGGAAGAAGATAATCAAAAGCATATAAATTGGGTTCGAAGTGTTT
ATAATTTCACAACTCCTTATGTGTCCCAAAATCAAAGAATCGCATATGTCAATTATAGGGACCTTGATTTAGG
AAAAACTAATCCCAAGAGTCCTAATAATTACACACAAGCATGTATTTGGGGTGAAAAGTATTTTAGTAAAAAT
TTTAACAGGTTTGTTAAGGTGAAAACCAAGGTTGATCCCAATAATTTTTTTAGAAACGAACAAAGCATCCCAC
CTCTTCCACCGCGTCGTCCTTAA
>A4_KJ469381.1
TCTCCCATATCCAAGCAACTATTCTATGCTCCAAGAAAGTTGGCTTGCAGATTCGAACTCGAAGCGGTGGCCA
TGATGCTGAGGGTTTGTCCTACACATCTCAAGTCCCATTTGTTATAGTAGACTTGAGAAACATGCATTCGGTG
AAAATAGATATTCGTAGCCAAACTGCGTGGGTTGAAGCCGGAGCTACCCTTGGAGAAGTTTATTATTGGATTA
ATGAGAAGAATGAGAATCTTAGTTTTCCTGGTGGGTATTGCCCTACTGTTGGCGTAGGTGGACACTTTAGTGG
AGGAGGCTATGGAGCATTAATGCGAAATTATGGCCTCGCAGCTGATAATATCATTGATGCACACTTAGTCAAT
GTTGATGGAAAAGTTCTAGATCGAAAATCCATGGGGGAAGATCTATTTTGGGCTATACGTGGTGGTGGAGGTG
AAAACTTTGGAATCATTGCAGCGTGGAAAATTAGACTGGTTGCTGTCCCATCAAGGGCTACTATATTCAGTGT
TAAAAGGAATATGGAGATACATGGGCTTGTCAAGTTATTTAACAAATGGCAAAATATTGCTTACAAGTATGAC
AAAGATTTATTACTCATGACTCACTTCATAACCAGGAATATTATAGATAATCAAGGAAAGAATAAGACTACAG
TACACGGTTACTTCTCTTGCATTTTCCATGGTGGAGTGGATAGTCTAGTCAACTTGATGAACAAGAGCTTTCC
TGAGTTGGGTATTAAAAAAACTGATTGCAAAGAATTGAGCTGGATTGATACTACCATCTTCTACAGTGGTGTT
GTAAATTATAACACTACTAATTTTCAAAAGGAAATTTTGCTTGATAGATCAGCTGGGCAGAAAGTAGCTTTCT
CAATTAAGTTAGACTACGTTAAGAAACCAATTCCAGAAACTGCAATTGTCAAAATTTTGGAGAAATTGTATGA
AGAAGATGTAGGAGCTGGGATGTATGTG
>A4_JLion_father_JAATIQ010000127.1_G4B88_028756
CTTAGATTCAACTCTGATGCAACCCCAAAACCACTCGTTATCGTCACTCCTTCAAATGTCTCCCACATCCAAG
CCCCAATTCTATGCTCCAAGAAAGTTGACTTGTAGATTCGAACTTGAAGCGGTAGCCACGATGCTGAGGGTTT
GTCCTACATATCTCAAGTCCCATTCGTTATAGTAGACTTGAGAAACATACATTTGATCAAAATAGATGTTCGT
AGCCAAACTGCATGGGTTGAAGCTGGAGCTACCCTTGGAGAAGTTTACTTCCGGATCAATGAGAAGAATGAGA
ATCTTAATTTTCCTGGTGGGTTTTGCCCTACTGTTGGCACAGGTGGACACTTTAGTGGAGGAGGCTATGGATC

ATTGATACGAAATTATGGCCTCGCGGCTAATAATATCATTGATGCACACATAGTCAATGTTGATGGAAAGTTT
CTAGATCGAAAATCCATGGGAGAAGATATGTTTTGGGCTATACGTGGTGGTGGAAGAAACTTTGGAATAATTA
CACTGTGGAAAATCAGACTGGTTGATGTCATGTCAAAGTCTACTATATTCAGTGTTAAAAAGAATATGGAGAT
ACATGGGCTTGTCAAGTTATTTGATAAATGGCAAAATATTGCTTACAAGTATGACAAAGATTTATTACTCATG
ACTCACTTCATAACCAAAAATACTACAGATAATCAAGGAAAGAATAAAACAACAGTACACGGTTACTTCCCTT
CCATTTTCCATGGTGGAGCGGATAGTTTGGTTGACTTGATGAACAAGAGCTTTCCTGAGTTGGGTATTAAAAA
AACTGATTGCAAAGAATTGAGCTGAATTGATACCACCATCTTCTACAGTGGAATTGTAAATAACACTGCTAAT
TTTAAAAAGGAAATTTTGCTTGATCGATCAGCTGGGCCGAAGACAGCTTTCTCAATTAAGTTAGACTACGTTA
AGCACCCAATTCCGAAAATCGCAATGGTCAAAATTTTGGAAAAATTATATGAAGAAGATGTAGGAGTTGGGAT
GTATCTTTTGATCCCGTACGGCGGTACAATGGATGAGATTTCAGAATCAGCAATTCCATTCCGTCATCGAGCT
GGAATCATGTATGAACTTTGGTACGCAGCTACCTAGGAAAAGCAAGAAGATAATCAAAAGCATATAAATTGGG
TTCGAAGTGTTTATAATTTCACAACTCCTTATGTGTCCTAAAATCAAAGATTCGCATATCTCAATTATAGGGA
CCTTGATTTAGGAAAAACTAATCCCAGGAGTCCTAATAATTACACACAAGCATGTATTTGGGGTGAAAAGTAT
TTTAGTAAAAATTTTAACAGGTTTGTTAAGGTGAAAACCAAGGTTGATCCCAATAATTTTTTTAGAAACGAAC
AAAGCATCCCACCTCTTCCACCGCGTCGTCCTTAA
>A4_LAconfidential_LKUA01148100.1_2
GTAGACTTGAGAAACATACATTTGATCAAAATAGATGTTCGTAGCCAAACTGCATGGGTTGAAGCTGGAGCTA
CCCTTGGAGAAGTTTACTTCCGGATCAATGAGAAGAATGAGAATCTTAATTTTCCTGGTGGGTTTTGCCCTAC
TGTTGGCGCAGGTGGACACTTTAGTGGAGGAGGCTATGGAGCATTGATACGAAATTATGGCCTCGCGGCTAAT
AATATCATTGATGCACACATAGTCAATGTTGATGGAAAGTTTCTAGATCGAAAATCCATGGGAGAAGATATGT
TTTGGGCTATACGTGGTGGTGGAGGAAACTTTGGAATAATTACACTGTGGAAAATCAGACTGGTTGATGTCAT
GTCAAAGTCTACTATATTCAGTGTTAAAAAGAATATGGAGATACATGGGCTTGTCAAGTTATTTGATAAATGG
CAAAATATTGCTTACAAGTATGACAAAGATTTATTACTCATGACTCACTTCATAACCAAAAATACTACAGATA
ATCAAGGAAAGAATAAAACAACAGTACACGGTTACTTCCCTTCCATTTTCCATGGTGGAGCGGATAGTTTAGT
TGACTTGATGAACAAGAGCTTTCTTGAGTTGGGTATTAAAAAAACTGATTGCAAAGAATTGAGCTGAATTGAT
ACCACCATCTTTTACAGTGGAATTGTAAATAACACTGCTAATTTTAAAAAGGAAATTTTGCTTGATCGATCAG
CTGGGCCGAAGACAGCTTTCTCAATTAAGTTAGACTACGTTAAGAACCCAATTCCGAAAATCGCAATGGTCAA
AATTTTGGAAAAATTATATGAAGAAGATGTAGGAGTTGGGATGTAACTTTTGATCCTGTACGGCGGTACAATG
GATGAGATTTCAGAATCAGCAATTCCATTCCGTCATCGAGCTGGAATCATGTATGAACTTTGGTACGCAGCTA
CCTAGGAAAAGCAAGAAGATAATCAAAAGCATATAAATTGGGTTCGAAGTGTTTATAATTTCACAACTCCTTA
TGTGTCCTAAAATCAAAGATTCCCATATCTCAATTATAGGGACCTTGATTTAGGAAAAACTAATCCCAAGAGT
CCTAATAATTACACACAAGCATGTATTTGGGGTGAAAAGTATTTTAGTAAAAATTTTAACAGGTTTGTTAAGG
TGAAAACCAAGGTTGATCCCAATAATTTTTTTAGAAACGAACAAAGCATCCCACCTCTTCCACCGCGTCGTCC
TTAA
>B1_JLion_father_JAATIQ010000127.1_G4B88_028766
ATGAAGTGCTCAACATTCTCCTTTTGGTTTGTTTGCAAGATAATATTTTTCTTTTTCTCATTCAATATCCAAA
CTTCCATTGCTAATCCTCGAGAAAACTTCCTTAAATGCTTCTCGCAATATATTCCCAATAATGCAACAAATCT
AAAACTCGTATACACTCAAAACAACCCATTGTATATGTCTGTCCTAAATTCGACAATACACAATCTTAGATTC
ACCTCTGACACAACCCCAAAACCACTTGTTATCGTCACTCCTTCACATGTCTCTCATATCCAAGGCACTATTC
TATGCTCCAAGAAAGTTGGCTTGCAGATTCGAACTCGAAGTGGTGGTCATGATTCTGAGGGCATGTCCTACAT
ATCTCAAGTCCCATTTGTTATAGTAGACTTGAGAAACATGCGTTCAATCAAAATAGATGTTCATAGCCAAACT
GCATGGGTTGAAGCCGGAGCTACCCTTGGAGAAGTTTATTATTGGGTTAATGAGAAAAATGAGAATCTTAGTT
TGGCGGCTGGGTATTGCCCTACTGTTTGCGCAGGTGGACACTTTGGTGGAGGAGGCTATGGACCATTGATGAG
AAACTATGGCCTCGCGGCTGATAATATCATTGATGCACACTTAGTCAACGTTCATGGAAAAGTGCTAGATCGA
AAATCTATGGGGGAAGATCTCTTTTGGGCTTTACGTGGTGGTGGAGCAGAAAGCTTCGGAATCATTGTAGCAT
GGAAAATTAGACTGGTTGCTGTCCCAAAGTCTACTATGTTTAGTGTTAAAAAGATCATGGAGATACATGAGCT
TGTCAAGTTAGTTAACAAATGGCAAAATATTGCTTACAAGTATGACAAAGATTTATTACTCATGACTCACTTC
ATAACTAGGAACATTACAGATAATCAAGGGAAGAATAAGACAGCAATACACACTTACTTCTCTTCAGTTTTCC
TTGGTGGAGTGGATAGTCTAGTCGACTTGATGAACAAGAGTTTTCCTGAGTTGGGTATTAAAAAAACGGATTG
CAGACAATTGAGCTGGATTGATACTATCATCTTCTATAGTGGTGTTGTAAATTACGACACTGATAATTTTAAC
AAGGAAATTTTGCTTGATAGATCCGCTGGGCAGAACGGTGCTTTCAAGATTAAGTTAGACTACGTTAAGAAAC
CAATTCCAGAATCTGTATTTGTCCAAATTTTGGAAAAATTATATGAAGAAGATATAGGAGCTGGGATGTATGC
GTTGTACCCTTACGGTGGTATAATGGATGAGATTTCAGAATCAGCAATTCCATTCCCTCATCGAGCTGGAATC
TTGTATGAGTTATGGTACATATGTAGTTGGGAGAAGCAAGAAGATAACGAAAAGCATCTAAACTGGATTAGAA
ATATTTATAACTTCATGACTCCTTATGTGTCCAAAAATCCAAGATTGGCATATCTCAATTATAGAGACCTTGA
TATAGGAATAAATGATCCCAAGAATCCAAATAATTACACACAAGCACGTATTTGGGGTGAGAAGTATTTTGGT
AAAAATTTTGACAGGCTAGTAAAAGTGAAAACCCTGGTTGATCCCAATAACTTTTTTAGAAACGAACAAAGCA
TCCCACCTCTTCCACGGCATCGTCATTAA

>B1_CBDRx_NC_044378.1_LOC115697762
ATGAAGTGCTCAACATTCTCCTTTTGGTTTGTTTGCAAGATAATATTTTTCTTTTTCTCATTCAATATCCAAA
CTTCCATTGCTAATCCTCGAGAAAACTTCCTTAAATGCTTCTCGCAATATATTCCCAATAATGCAACAAATCT
AAAACTCGTATACACTCAAAACAACCCATTGTATATGTCTGTCCTAAATTCGACAATACACAATCTTAGATTC
ACCTCTGACACAACCCCAAAACCACTTGTTATCGTCACTCCTTCACATGTCTCTCATATCCAAGGCACTATTC
TATGCTCCAAGAAAGTTGGCTTGCAGATTCGAACTCGAAGTGGTGGTCATGATTCTGAGGGCATGTCCTACAT
ATCTCAAGTCCCATTTGTTATAGTAGACTTGAGAAACATGCGTTCAATCAAAATAGATGTTCATAGCCAAACT
GCATGGGTTGAAGCCGGAGCTACCCTTGGAGAAGTTTATTATTGGGTTAATGAGAAAAATGAGAATCTTAGTT
TGGCGGCTGGGTATTGCCCTACTGTTTGCGCAGGTGGACACTTTGGTGGAGGAGGCTATGGACCATTGATGAG
AAACTATGGCCTCGCGGCTGATAATATCATTGATGCACACTTAGTCAACGTTCATGGAAAAGTGCTAGATCGA
AAATCTATGGGGGAAGATCTCTTTTGGGCTTTACGTGGTGGTGGAGCAGAAAGCTTCGGAATCATTGTAGCAT
GGAAAATTAGACTGGTTGCTGTCCCAAAGTCTACTATGTTTAGTGTTAAAAAGATCATGGAGATACATGAGCT
TGTCAAGTTAGTTAACAAATGGCAAAATATTGCTTACAAGTATGACAAAGATTTATTACTCATGACTCACTTC
ATAACTAGGAACATTACAGATAATCAAGGGAAGAATAAGACAGCAATACACACTTACTTCTCTTCAGTTTTCC
TTGGTGGAGTGGATAGTCTAGTCGACTTGATGAACAAGAGTTTTCCTGAGTTGGGTATTAAAAAAACGGATTG
CAGACAATTGAGCTGGATTGATACTATCATCTTCTATAGTGGTGTTGTAAATTACGACACTGATAATTTTAAC
AAGGAAATTTTGCTTGATAGATCCGCTGGGCAGAACGGTGCTTTCAAGATTAAGTTAGACTACGTTAAGAAAC
CAATTCCAGAATCTGTATTTGTCCAAATTTTGGAAAAATTATATGAAGAAGATATAGGAGCTGGGATGTATGC
GTTGTACCCTTACGGTGGTATAATGGATGAGATTTCAGAATCAGCAATTCCATTCCCTCATCGAGCTGGAATC
TTGTATGAGTTATGGTACATATGTAGTTGGGAGAAGCAAGAAGATAACGAAAAGCATCTAAACTGGATTAGAA
ATATTTATAACTTCATGACTCCTTATGTGTCCAAAAATCCAAGATTGGCATATCTCAATTATAGAGACCTTGA
TATAGGAATAAATGATCCCAAGAATCCAAATAATTACACACAAGCACGTATTTGGGGTGAGAAGTATTTTGGT
AAAAATTTTGACAGGCTAGTAAAAGTGAAAACCCTGGTTGATCCCAATAACTTTTTTAGAAACGAACAAAGCA
TCCCACCTCTTCCACGGCATCGTCATTAA
>B1_Jamaican_Lion_JAATIP010000103.1_F8388_004088
ATGAAGTGCTCAACATTCTCCTTTTGGTTTGTTTGCAAGATAATATTTTTCTTTTTCTCATTCAATATCCAAA
CTTCCATTGCTAATCCTCGAGAAAACTTCCTTAAATGCTTCTCGCAATATATTCCCAATAATGCAACAAATCT
AAAACTCGTATACACTCAAAACAACCCATTGTATATGTCTGTCCTAAATTCGACAATACACAATCTTAGATTC
ACCTCTGACACAACCCCAAAACCACTTGTTATCGTCACTCCTTCACATGTCTCTCATATCCAAGGCACTATTC
TATGCTCCAAGAAAGTTGGCTTGCAGATTCGAACTCGAAGTGGTGGTCATGATTCTGAGGGCATGTCCTACAT
ATCTCAAGTCCCATTTGTTATAGTAGACTTGAGAAACATGCGTTCAATCAAAATAGATGTTCATAGCCAAACT
GCATGGGTTGAAGCCGGAGCTACCCTTGGAGAAGTTTATTATTGGGTTAATGAGAAAAATGAGAATCTTAGTT
TGGCGGCTGGGTATTGCCCTACTGTTTGCGCAGGTGGACACTTTGGTGGAGGAGGCTATGGACCATTGATGAG
AAACTATGGCCTCGCGGCTGATAATATCATTGATGCACACTTAGTCAACGTTCATGGAAAAGTGCTAGATCGA
AAATCTATGGGGGAAGATCTCTTTTGGGCTTTACGTGGTGGTGGAGCAGAAAGCTTCGGAATCATTGTAGCAT
GGAAAATTAGACTGGTTGCTGTCCCAAAGTCTACTATGTTTAGTGTTAAAAAGATCATGGAGATACATGAGCT
TGTCAAGTTAGTTAACAAATGGCAAAATATTGCTTACAAGTATGACAAAGATTTATTACTCATGACTCACTTC
ATAACTAGGAACATTACAGATAATCAAGGGAAGAATAAGACAGCAATACACACTTACTTCTCTTCAGTTTTCC
TTGGTGGAGTGGATAGTCTAGTCGACTTGATGAACAAGAGTTTTCCTGAGTTGGGTATTAAAAAAACGGATTG
CAGACAATTGAGCTGGATTGATACTATCATCTTCTATAGTGGTGTTGTAAATTACGACACTGATAATTTTAAC
AAGGAAATTTTGCTTGATAGATCCGCTGGGCAGAACGGTGCTTTCAAGATTAAGTTAGACTACGTTAAGAAAC
CAATTCCAGAATCTGTATTTGTCCAAATTTTGGAAAAATTATATGAAGAAGATATAGGAGCTGGGATGTATGC
GTTGTACCCTTACGGTGGTATAATGGATGAGATTTCAGAATCAGCAATTCCATTCCCTCATCGAGCTGGAATC
TTGTATGAGTTATGGTACATATGTAGTTGGGAGAAGCAAGAAGATAACGAAAAGCATCTAAACTGGATTAGAA
ATATTTATAACTTCATGACTCCTTATGTGTCCAAAAATCCAAGATTGGCATATCTCAATTATAGAGACCTTGA
TATAGGAATAAATGATCCCAAGAATCCAAATAATTACACACAAGCACGTATTTGGGGTGAGAAGTATTTTGGT
AAAAATTTTGACAGGCTAGTAAAAGTGAAAACCCTGGTTGATCCCAATAACTTTTTTAGAAACGAACAAAGCA
TCCCACCTCTTCCACGGCATCGTCATTAA
>B1_Finola_CM011610.1_21837038
ATGAAGTACTCAACATTCTCCTTTTGGTTTGTTTGCAAGATAATATTTTTCTTTTTCTCATTCAATATCCAAA
CTTCCATTGCTAATCCTCGAGAAAACTTCCTTAAATGCTTCTCGCAATATATTCCCAATAATGCAACAAATCT
AAAACTCGTATACACTCAAAACAACCCATTGTATATGTCTGTCCTAAATTCGACAATACACAATCTTAGATTC
AGCTCTGACACAACCCCAAAACCACTTGTTATCGTCACTCCTTCACATGTCTCTCATATCCAAGGCACTATTC
TATGCTCCAAGAAAGTTGGCTTGCAGATTCGAACTCGAAGTGGTGGTCATGATTCTGAGGGCATGTCCTACAT
ATCTCAAGTCCCATTTGTTATAGTAGACTTGAGAAACATGCGTTCAATCAAAATAGATGTTCATAGCCAAACT
GCATGGGTTGAAGCCGGAGCTACCCTTGGAGAAGTTTATTATTGGGTTAATGAGAAAAATGAGAGTCTTAGTT
TGGCTGCTGGGTATTGCCCTACTGTTTGCGCAGGTGGACACTTTGGTGGAGGAGGCTATGGACCATTGATGAG
AAGCTATGGCCTCGCGGCTGATAATATCATTGATGCACACTTAGTCAACGTTCATGGAAAAGTGCTAGATCGA

AAATCTATGGGGGAAGATCTCTTTTGGGCTTTACGTGGTGGTGGAGCAGAAAGCTTCGGAATCATTGTAGCAT
GGAAAATTAGACTGGTTGCTGTCCCAAAGTCTACTATGTTTAGTGTTAAAAAGATCATGGAGATACATGAGCT
TGTCAAGTTAGTTAACAAATGGCAAAATATTGCTTACAAGTATGACAAAGATTTATTACTCATGACTCACTTC
ATAACTAGGAACATTACAGATAATCAAGGGAAGAATAAGACAGCAATACACACTTACTTCTCTTCAGTTTTCC
TTGGTGGAGTGGATAGTCTAGTCGACTTGATGAACAAGAGTTTTCCTGAGTTGGGTATTAAAAAAACGGATTG
CAGACAATTGAGCTGGATTGATACTATCATCTTCTATAGTGGTGTTGTAAATTACGACACTGATAATTTTAAC
AAGGAAATTTTGCTTGATAGATCCGCTGGGCAGAACGGTGCTTTCAAGATTAAGTTAGACTACGTTAAGAAAC
CAATTCCAGAATCTGTATTTGTCCAAATTTTGGAAAAATTATATGAAGAAGATATAGGAGCTGGGATGTATGC
GTTGTACCCTTACGGTGGTATAATGGATGAGATTTCTGAATCAGCAATTCCATTCCCTCATCGAGCTGGAATC
TTGTATGAGTTATGGTACATATGTAGCTGGGAGAAGCAAGAAGATAACGAAAAGCATCTAAACTGGATTAGAA
ATATTTATAACTTCATGACTCCTTATGTGTCCCAAAATCCAAGATTGGCATATCTCAATTATAGAGACCTTGA
TATAGGAATAAATGATCCCAAGAATCCAAATAATTACACACAAGCACGTATTTGGGGTGAGAAGTATTTTGGT
AAAAATTTTGACAGGCTAGTAAAAGTGAAAACCCTGGTTGATCCCAATAATTTTTTTAGAAACGAACAAAGCA
TCCCACCTCTTCCACGGCATCATCATTAA
>B1_AB292682.1
ATGAAGTGCTCAACATTCTCCTTTTGGTTTGTTTGCAAGATAATATTTTTCTTTTTCTCATTCAATATCC
AAACTTCCATTGCTAATCCTCGAGAAAACTTCCTTAAATGCTTCTCGCAATATATTCCCAATAATGCAAC
AAATCTAAAACTCGTATACACTCAAAACAACCCATTGTATATGTCTGTCCTAAATTCGACAATACACAAT
CTTAGATTCACCTCTGACACAACCCCAAAACCACTTGTTATCGTCACTCCTTCACATGTCTCTCATATCC
AAGGCACTATTCTATGCTCCAAGAAAGTTGGCTTGCAGATTCGAACTCGAAGTGGTGGTCATGATTCTGA
GGGCATGTCCTACATATCTCAAGTCCCATTTGTTATAGTAGACTTGAGAAACATGCGTTCAATCAAAATA
GATGTTCATAGCCAAACTGCATGGGTTGAAGCCGGAGCTACCCTTGGAGAAGTTTATTATTGGGTTAATG
AGAAAAATGAGAATCTTAGTTTGGCGGCTGGGTATTGCCCTACTGTTTGCGCAGGTGGACACTTTGGTGG
AGGAGGCTATGGACCATTGATGAGAAACTATGGCCTCGCGGCTGATAATATCATTGATGCACACTTAGTC
AACGTTCATGGAAAAGTGCTAGATCGAAAATCTATGGGGGAAGATCTCTTTTGGGCTTTACGTGGTGGTG
GAGCAGAAAGCTTCGGAATCATTGTAGCATGGAAAATTAGACTGGTTGCTGTCCCAAAGTCTACTATGTT
TAGTGTTAAAAAGATCATGGAGATACATGAGCTTGTCAAGTTAGTTAACAAATGGCAAAATATTGCTTAC
AAGTATGACAAAGATTTATTACTCATGACTCACTTCATAACTAGGAACATTACAGATAATCAAGGGAAGA
ATAAGACAGCAATACACACTTACTTCTCTTCAGTTTTCCTTGGTGGAGTGGATAGTCTAGTCGACTTGAT
GAACAAGAGTTTTCCTGAGTTGGGTATTAAAAAAACGGATTGCAGACAATTGAGCTGGATTGATACTATC
ATCTTCTATAGTGGTGTTGTAAATTACGACACTGATAATTTTAACAAGGAAATTTTGCTTGATAGATCCG
CTGGGCAGAACGGTGCTTTCAAGATTAAGTTAGACTACGTTAAGAAACCAATTCCAGAATCTGTATTTGT
CCAAATTTTGGAAAAATTATATGAAGAAGATATAGGAGCTGGGATGTATGCGTTGTACCCTTACGGTGGT
ATAATGGATGAGATTTCAGAATCAGCAATTCCATTCCCTCATCGAGCTGGAATCTTGTATGAGTTATGGT
ACATATGTAGTTGGGAGAAGCAAGAAGATAACGAAAAGCATCTAAACTGGATTAGAAATATTTATAACTT
CATGACTCCTTATGTGTCCAAAAATCCAAGATTGGCATATCTCAATTATAGAGACCTTGATATAGGAATA
AATGATCCCAAGAATCCAAATAATTACACACAAGCACGTATTTGGGGTGAGAAGTATTTTGGTAAAAATT
TTGACAGGCTAGTAAAAGTGAAAACCCTGGTTGATCCCAATAACTTTTTTAGAAACGAACAAAGCATCCC
ACCTCTTCCACGGCATCGTCATTAA

>B1_KP970862.1
ATGAAGTGCTCAACATTCTCCTTTTGGTTTGTTTGCAAGATAATATTTTTCTTTTTCTCATTCAATATCC
AAACTTCCATTGCTAATCCTCGAGAAAACTTCCTTAAATGCTTCTCGCAATATATTCCCAATAATGCAAC
AAATCTAAAACTCGTATACACTCAAAACAACCCATTGTATATGTCTGTCCTAAATTCGACAATACACAAT
CTTAGATTCACCTCTGACACAACCCCAAAACCACTTGTTATCGTCACTCCTTCACATGTCTCTCATATCC
AAGGCACTATTCTATGCTCCAAGAAAGTTGGCTTGCAGATTCGAACTCGAAGTGGTGGTCATGATTCTGA
GGGCATGTCCTACATATCTCAAGTCCCATTTGTTATAGTAGACTTGAGAAACATGCGTTCAATCAAAATA
GATGTTCATAGCCAAACTGCATGGGTTGAAGCCGGAGCTACCCTTGGAGAAGTTTATTATTGGGTTAATG
AGAAAAATGAGAATCTTAGTTTGGCGGCTGGGTATTGCCCTACTGTTTGCGCAGGTGGACACTTTGGTGG
AGGAGGCTATGGACCATTGATGAGAAACTATGGCCTCGCGGCTGATAATATCATTGATGCACACTTAGTC
AACGTTCATGGAAAAGTGCTAGATCGAAAATCTATGGGGGAAGATCTCTTTTGGGCTTTACGTGGTGGTG
GAGCAGAAAGCTTCGGAATCATTGTAGCATGGAAAATTAGACTGGTTGCTGTCCCAAAGTCTACTATGTT
TAGTGTTAAAAAGATCATGGAGATACATGAGCTTGTCAAGTTAGTTAACAAATGGCAAAATATTGCTTAC
AAGTATGACAAAGATTTATTACTCATGACTCACTTCATAACTAGGAACATTACAGATAATCAAGGGAAGA
ATAAGACAGCAATACACACTTACTTCTCTTCAGTTTTCCTTGGTGGAGTGGATAGTCTAGTCGACTTGAT
GAACAAGAGTTTTCCTGAGTTGGGTATTAAAAAAACGGATTGCAGACAATTGAGCTGGATTGATACTATC
ATCTTCTATAGTGGTGTTGTAAATTACGACACTGATAATTTTAACAAGGAAATTTTGCTTGATAGATCCG
CTGGGCAGAACGGTGCTTTCAAGATTAAGTTAGACTACGTTAAGAAACCAATTCCAGAATCTGTATTTGT

CCAAATTTTGGAAAAATTATATGAAGAAGATATAGGAGCTGGGATGTATGCGTTGTACCCTTACGGTGGT
ATAATGGATGAGATTTCAGAATCAGCAATTCCATTCCCTCATCGAGCTGGAATCTTGTATGAGTTATGGT
ACATATGTAGTTGGGAGAAGCAAGAAGATAACGAAAAGCATCTAAACTGGATTAGAAATATTTATAACTT
CATGACTCCTTATGTGTCCAAAAATCCAAGATTGGCATATCTCAATTATAGAGACCTTGATATAGGAATA
AATGATCCCAAGAATCCAAATAATTACACACAAGCACGTATTTGGGGTGAGAAGTATTTTGGTAAAAATT
TTGACAGGCTAGTAAAAGTGAAAACCCTGGTTGATCCCAATAACTTTTTTAGAAACGAACAAAGCATCCC
ACCTCTTCCACGGCATCGTCATTAA

>B1_MG996433.1
TTCAATATCCAAACTTCCATTGCTAATCCTCGAGAAAACTTCCTTAAATGCTTCTCGCAATATATTCCCAATA
ATGCAACAAATCTAAAACTCGTATACACTCAAAACAACCCATTGTATATGTCTGTCCTAAATTCGACAATACA
CAATCTTAGATTCACCTCTGACACAACCCCAAAACCACTTGTTATCGTCACTCCTTCACATGTCTCTCATATC
CAAGGCACTATTCTATGCTCCAAGAAAGTTGGCTTGCAGATTCGAACTCGAAGTGGTGGTCATGATTCTGAGG
GCATGTCCTACATATCTCAAGTCCCATTTGTTATAGTAGACTTGAGAAACATGCGTTCAATCAAAATAGATGT
TCATAGCCAAACTGCATGGGTTGAAGCCGGAGCTACCCTTGGAGAAGTTTATTATTGGGTTAATGAGAAAAAT
GAGAATCTTAGTTTGGCGGCTGGGTATTGCCCTACTGTTTGCGCAGGTGGACACTTTGGTGGAGGAGGCTATG
GACCATTGATGAGAAACTATGGCCTCGCGGCTGATAATATCATTGATGCACACTTAGTCAACGTTCATGGAAA
AGTGCTAGATCGAAAATCTATGGGGGAAGATCTCTTTTGGGCTTTACGTGGTGGTGGAGCAGAAAGCTTCGGA
ATCATTGTAGCATGGAAAATTAGACTGGTTGCTGTCCCAAAGTCTACTATGTTTAGTGTTAAAAAGATCATGG
AGATACATGAGCTTGTCAAGTTAGTTAACAAATGGCAAAATATTGCTTACAAGTATGACAAAGATTTATTACT
CATGACTCACTTCATAACTAGGAACATTACAGATAATCAAGGGAAGAATAAGACAGCAATACACACTTACTTC
TCTTCAGTTTTCCTTGGTGGAGTGGATAGTCTAGTCGACTTGATGAACAAGAGTTTTCCTGAGTTGGGTATTA
AAAAAACGGATTGCAGACAATTGAGCTGGATTGATACTATCATCTTCTATAGTGGTGTTGTAAATTACGACAC
TGATAATTTTAACAAGGAAATTTTGCTTGATAGATCCGCTGGGCAGAACGGTGCTTTCAAGATTAAGTTAGAC
TACGTTAAGAAACCAATTCCAGAATCTGTATTTGTCCAAATTTTGGAAAAATTATATGAAGAAGATATAGGAG
CTGGGATGTATGCGTTGTACCCTTACGGTGGTATAATGGATGAGATTTCAGAATCAGCAATTCCATTCCCTCA
TCGAGCTGGAATCTTGTATGAGTTATGGTACATATGTAGTTGGGAGAAGCAAGAAGATAACGAAAAGCATCTA
AACTGGATTAGAAATATTTATAACTTCATGACTCCTTATGTGTCCAAAAATCCAAGATTGGCATATCTCAATT
ATAGAGACCTTGATATAGGAATAAATGATCCCAAGAATCCAAATAATTACACACAAGCACGTATTTGGGGTGA
GAAGTATTTTGGTAAAAATTTTGACAGGCTAGTAAAAGTGAAAACCCTGGTTGATCCCAAT
>B1_KP970866.1
ATGAAGTGCTCAACATTCTCCTTTTGGTTTGTTTGCAAGATAATATTTTTCTTTTTCTCATTCAATATCC
AAACTTCCATTGCTAATCCTCGAGAAAACTTCCTTAAATGCTTCTCGCAATATATTCCCAATAATGCAAC
AAATCTAAAACTCGTATACACTCAAAACAACCCATTGTATATGTCTGTCCTAAATTCGACAATACACAAT
CTTAGATTCACCTCTGACACAACCCCAAAACCACTTGTTATCGTCACTCCTTCACATGTCTCTCATATCC
AAGGCACTATTCTATGCTCCAAGAAAGTTGGCTTGCAGATTCGAACTCGAAGTGGTGGTCATGATTCTGA
GGGCATGTCCTACATATCTCAAGTCCCATTTGTTATAGTAGACTTGAGAAACATGCGTTCAATCAAAATA
GATGTTCATAGCCAAACTGCATGGGTTGAAGCCGGAGCTACCCTTGGAGAAGTTTATTATTGGGTTAATG
AGAAAAATGAGAATCTTAGTTTGGCGGCTGGGTATTGCCCTACTGTTTGCGCAGGTGGACACTTTGGTGG
AGGAGGCTATGGACCATTGATGAGAAACTATGGCCTCGCGGCTGATAATATCATTGATGCACACTTAGTC
AACGTTCATGGAAAAGTGCTAGATCGAAAATCTATGGGGGAAGATCTCTTTTGGGCTTTACGTGGTGGTG
GAGCAGAAAGCTTCGGAATCATTGTAGCATGGAAAATTAGACTGGTTGCTGTCCCAAAGTCTACTATGTT
TAGTGTTAAAAAGATCATGGAGATACATGAGCTTGTCAAGTTAGTTAACAAATGGCAAAATATTGCTTAC
AAGTATGACAAAGATTTATTACTCATGACTCACTTCATAACTAGGAACATTACAGATAATCAAGGGAAGA
ATAAGACAGCAATACACACTTACTTCTCTTCAGTTTTCCTTGGTGGAGTGGATAGTCTAGTCGACTTGAT
GAACAAGAGTTTTCCTGAGTTGGGTATTAAAAAAACGGATTGCAGACAATTGAGCTGGATTGATACTATC
ATCTTCTATAGTGGTGTTGTAAATTACGACACTGATAATTTTAACAAGGAAATTTTGCTTGATAGATCCG
CTGGGCAGAACGGTGCTTTCAAGATTAAGTTAGACTACGTTAAGAAACCAATTCCAGAATCTGTATTTGT
CCAAATTTTGGAAAAATTATATGAAGAAGATATAGGAGCTGGGATGTATGCGTTGTACCCTTACGGTGGT
ATAATGGATGAGATTTCAGAATCAGCAATTCCATTCCCTCATCGAGCTGGAATCTTGTATGAGTTATGGT
ACATATGTAGTTGGGAGAAGCAAGAAGATAACGAAAAGCATCTAAACTGGATTAGAAATATTTATAACTT
CATGACTCCTTATGTGTCCAAAAATCCAAGATTGGCATATCTCAATTATAGAGACCTTGATATAGGAATA
AATGATCCCAAGAATCCAAATAATTACACACAAGCACGTATTTGGGGTGAGAAGTATTTTGGTAAAAATT
TTGACAGGCTAGTAAAAGTGAAAACCCTGGTTGATCCCAATAACTTTTTTAGAAACGAACAAAGCATCCC
ACCTCTACCACGGCATCGTCATTAA

>B1_KP970859.1
ATGAAGTGCTCAACATTCTCCTTTTGGTTTGTTTGCAAGATAATATTTTTCTTTTTCTCATTCAATATCC

AAACTTCCATTGCTAATCCTCGAGAAAACTTCCTTAAATGCTTCTCGCAATATATTCCCAATAATGCAAC
AAATCTAAAACTCGTATACACTCAAAACAACCCATTGTATATGTCTGTCCTAAATTCGACAATACACAAT
CTTAGATTCACCTCTGACACAACCCCAAAACCACTTGTTATCGTCACTCCTTCACATGTCTCTCATATCC
AAGGCACTATTCTATGCTCCAAGAAAGTTGGCTTGCAGATTCGAACTCGAAGTGGTGGTCATGATTCTGA
GGGCATGTCCTACATATCTCAAGTCCCATTTGTTATAGTAGACTTGAGAAACATGCGTTCAATCAAAATA
GATGTTCATAGCCAAACTGCATGGGTTGAAGCCGGAGCTACCCTTGGAGAAGTTTATTATTGGGTTAATG
AGAAAAATGAGAATCTTAGTTTGGCGGCTGGGTATTGCCCTACTGTTTGCGCAGGTGGACACTTTGGTGG
AGGAGGCTATGGACCATTGATGAGAAACTATGGCCTCGCGGCTGATAATATCATTGATGCACACTTAGTC
AACGTTCATGGAAAAGTGCTAGATCGAAAATCTATGGGGGAAGATCTCTTTTGGGCTTTACGTGGTGGTG
GAGCAGAAAGCTTCGGAATCATTGTAGCATGGAAAATTAGACTGGTTGCTGTCCCAAAGTCTACTATGTT
TAGTGTTAAAAAGATCATGGAGATACATGAGCTTGTCAAGTTAGTTAACAAATGGCAAAATATTGCTTAC
AAGTATGACAAAGATTTATTACTCATGACTCACTTCATAACTAGGAACATTACAGATAATCAAGGGAAGA
ATAAGACAGCAATACACACTTACTTCTCTTCAGTTTTCCTTGGTGGAGTGGATAGTCTAGTCGACTTGAT
GAACAAGAGTTTTCCTGAGTTGGGTATTAAAAAAACGGATTGCAGACAATTGAGCTGGATTGATACTATC
ATCTTCTATAGTGGTGTTGTAAATTACGACACTGATAATTTTAACAAGGAAATTTTGCTTGATAGATCCG
CTGGGCAGAACGGTGCTTTCAAGATTAAGTTAGACTACGTTAAGAAACCAATTCCAGAATCTGTATTTGT
CCAAATTTTGGAAAAATTATATGAAGAAGATATAGGAGCTGGGATGTATGCGTTGTACCCTTACGGTGGT
ATAATGGATGAGATTTCAGAATCAGCAATTCCATTCCCTCATCGAGCTGGAATCTTGTATGAGTTATGGT
ACATATGTAGTTGGGAGAAGCAAGAAGATAACGAAAAGCATCTAAACTGGATTAGAAATATTTATAACTT
CATGACTCCTTATGTGTCCAAAAATTCAAGATTGGCATATCTCAATTATAGAGACCTTGATATAGGAATA
AATGATCCCAAGAATCCAAATAATTACACACAAGCACGTATTTGGGGTGAGAAGTATTTTGGTAAAAATT
TTGACAGGCTAGTAAAAGTGAAAACCCTGGTTGATCCCAATAACTTTTTTAGAAACGAACAAAGCATCCC
ACCTCTTCCACGGCATCGTCATTAA

>B1_MG996437.1
CAAACTTCCATTGCTAATCCTCGAGAAAACTTCCTTAAATGCTTCTCGCAATATATTCCCAATAATGCAACAA
ATCTAAAACTCGTATACACTCAAAACAACCCATTGTATATGTCTGTCCTAAATTCGACAATACACAATCTTAG
ATTCACCTCTGACACAACCCCAAAACCACTTGTTATCGTCACTCCTTCACATGTCTCTCATATCCAAGGCACT
ATTCTATGCTCCAAGAAAGTTGGCTTGCAGATTCGAACTCGAAGTGGTGGTCATGATTCTGAGGGCATGTCCT
ACATATCTCAAGTCCCATTTGTTATAGTAGACTTGAGAAACATGCGTTCAATCAAAATAGATGTTCATAGCCA
AACTGCATGGGTTGAAGCCGGAGCTACCCTTGGAGAAGTTTATTATTGGGTTAATGAGAAAAATGAGAATCTT
AGTTTGGCGGCTGGGTATTGCCCTACTGTTTGCGCAGGTGGACACTTTGGTGGAGGAGGCTATGGACCATTGA
TGAGAAACTATGGCCTCGCGGCTGATAATATCATTGATGCACACTTAGTCAACGTTCATGGAAAAGTGCTAGA
TCGAAAATCTATGGGGGAAGATCTCTTTTGGGCTTTACGTGGTGGTGGAGCAGAAAGCTTCGGAATCATTGTA
GCATGGAAAATTAGACTGGTTGCTGTCCCAAAGTCTACTATGTTTAGTGTTAAAAAGATCATGGAGATACATG
AGCTTGTCAAGTTAGTTAACAAATGGCAAAATATTGCTTACAAGTATGACAAAGATTTATTACTCATGACTCA
CTTCATAACTAGGAACATTACAGATAATCAAGGGAAGAATAAGACAGCAATACACACTTACTTCTCTTCAGTT
TTCCTTGGTGGAGTGGATAGTCTAGTCGACTTGATGAACAAGAGTTTTCCTGAGTTGGGTATTAAAAAAACGG
ATTGCAGACAATTGAGCTGGATTGATACTATCATCTTCTATAGTGGTGTTGTAAATTACGACACTGATAATTT
TAACAAGGAAATTTTGCTTGATAGATCCGCTGGGCAGAACGGTGCTTTCAAGATTAAGTTAGACTACGTTAAG
AAACCAATTCCAGAATCTGTATTTGTCCAAATTTTGGAAAAATTATATGAAGAAGATATAGGAGCTGGGATGT
ATGCGTTGTACCCTTACGGTGGTATAATGGATGAGATTTCAGAATCAGCAATTCCATTCCCTCATCGAGCTGG
AATCTTGTATGAGTTATGGTACATATGTAGTTGGGAGAAGCAAGAAGATAACGAAAAGCATCTAAACTGGATT
AGAAATATTTATAACTTCATGACTCCTTATGTGTCCAAAAATTCAAGATTGGCATATCTCAATTATAGAGACC
TTGATATAGGAATAAATGATCCCAAGAATCCAAATAATTACACACAAGCACGTATTTGGGGTGAGAAGTATTT
TGGTAAAAATTTTGACAGGCTAGTAAAAGTGAAAACCCTGGTTGA
>B1_KP970868.1
ATGAAGTGCTCAACATTCTCCTTTTGGTTTGTTTGCAAGATAATATTTTTCTTTTTCTCATTCAATATCC
AAACTTCCATTGCTAATCCTCGAGAAAACTTCCTTAAATGCTTCTCGCAATATATTCCCAATAATGCAAC
AAATCTAAAACTCGTATACACTCAAAACAACCCATTGTATATGTCTGTCCTAAATTCGACAATACACAAT
CTTAGATTCACCTCTGACACAACCCCAAAACCACTTGTTATCGTCACTCCTTCACATGTCTCTCATATCC
AAGGCACTATTCTATGCTCCAAGAAAGTTGGCTTGCAGATTCGAACTCGAAGTGGTGGTCATGATTCTGA
GGGCATGTCCTACATATCTCAAGTCCCATTTGTTATAGTAGACTTGAGAAACATGCGTTCAATCAAAATA
GATGTTCATAGCCAAACTGCATGGGTTGAAGCCGGAGCTACCCTTGGAGAAGTTTATTATTGGGTTAATG
AGAAAAATGAGAATCTTAGTTTGGCGGCTGGGTATTGCCCTACTGTTTGCGCAGGTGGACACTTTGGTGG
AGGAGGCTATGGACCATTGATGAGAAACTATGGCCTCGCGGCTGATAATATCATTGATGCACACTTAGTC
AACGTTCATGGAAAAGTGCTAGATCGAAAATCTATGGGGGAAGATCTCTTTTGGGCTTTACGTGGTGGTG
GAGCAGAAAGCTTCGGAATCATTGTAGCATGGAAAATTAGACTGGTTGCTGTCCCAAAGTCTACTATGTT

TAGTGTTAAAAAGATCATGGAGATACATGAGCTTGTCAAGTTAGTTAACAAATGGCAAAATATTGCTTAC
AAGTATGACAAAGATTTATTACTCATGACTCACTTCATAACTAGGAACATTACAGATAATCAAGGGAAGA
ATAAGACAGCAATACACACTTACTTCTCTTCAGTTTTCCTTGGTGGAGTGGATAGTCTAGTCGACTTGAT
GAACAAGAGTTTTCCTGAGTTGGGTATTAAAAAAACGGATTGCAGACAATTGAGCTGGATTGATACTATC
ATCTTCTATAGTGGTGTTGTAAATTACGACACTGATAATTTTAACAAGGAAATTTTGCTTGATAGATCCG
CTGGGCAGAACGGTGCTTTCAAGATTAAGTTAGACTACGTTAAGAAACCAATTCCAGAATCTGTATTTGT
CCAAATTTTGGAAAAATTATATGAAGAAGATATAGGAGCTGGGATGTATGCGTTGTACCCTTACGGTGGT
ATAATGGATGAGATTTCAGAATCAGCAATTCCATTCCCTCATCGAGCTGGAATCTTGTATGAGTTATGGT
ACATATGTAGTTGGGAGAAGCAAGAAGATAACGAAAAGCATCTAAACTGGATTAGAAATATTTATAACTT
CATGACTCCTTATGTGTCCAAAAATTCAAGATTGGCATATCTCAATTATAGAGACCTTGATATAGGAATA
AATGATCCCAAGAATCCAAATAATTACACACAAGCACGTATTTGGGGTGAGAAGTATTTTGGTAAAAATT
TTGACAGGCTAGTAAAAGTGAAAACCCTGGTTGATCCCAATAACTTTTTAGAAACGAACAAAGCATCCC
ACCTCAACCACGGCATCGTCATTAA

>B1_KP970867.1
ATGAAGTGCTCAACATTCTCCTTTTGGTTTGTTTGCAAGATAATATTTTTCTTTTTCTCATTCAATATCC
AAACTTCCATTGCTAATCCTCGAGAAAACTTCCTTAAATGCTTCTCGCAATATATTCCCAATAATGCAAC
AAATCTAAAACTCGTATACACTCAAAACAACCCATTGTATATGTCTGTCCTAAATTCGACAATACACAAT
CTTAGATTCACCTCTGACACAACCCCAAAACCACTTGTTATCGTCACTCCTTCACATGTCTCTCATATCC
AAGGCACTATTCTATGCTCCAAGAAAGTTGGCTTGCAGATTCGAACTCGAAGTGGTGGTCATGATTCTGA
GGGCATGTCCTACATATCTCAAGTCCCATTTGTTATAGTAGACTTGAGAAACATGCGTTCAATCAAAATA
GATGTTCGTAGCCAAACTGCATGGGTTGAAGCCGGAGCTACCCTTGGAGAAGTTTATTATTGGGTTAATG
AGAAAAATGAGAATCTTAGTTTGGCGGCTGGGTATTGCCCTACTGTTTGCGCAGGTGGACACTTTGGTGG
AGGAGGCTATGGACCATTGATGAGAAACTATGGCCTCGCGGCTGATAATATCATTGATGCACACTTAGTC
AACGTTCATGGAAAAGTGCTAGATCGAAAATCTATGGGGGAAGATCTCTTTTGGGCTTTACGTGGTGGTG
GAGCAGAAAGCTTCGGAATCATTGTAGCATGGAAAATTAGACTGGTTGCTGTCCCAAAGTCTACTATGTT
TAGTGTTAAAAAGATCATGGAGATACATGAGCTTGTCAAGTTAGTTAACAAATGGCAAAATATTGCTTAC
AAGTATGACAAAGATTTATTACTCATGACTCACTTCATAACTAGGAACATTACAGATAATCAAGGGAAGA
ATAAGACAGCAATACACACTTACTTCTCTTCAGTTTTCCTTGGTGGAGTGGATAGTCTAGTCGACTTGAT
GAACAAGAGTTTTCCTGAGTTGGGTATTAAAAAAACGGATTGCAGACAATTGAGCTGGATTGATACTATC
ATCTTCTATAGTGGTGTTGTAAATTACGACACTGATAATTTTAACAAGGAAATTTTGCTTGATAGATCCG
CTGGGCAGAACGGTGCTTTCAAGATTAAGTTAGACTACGTTAAGAAACCAATTCCAGAATCTGTATTTGT
CCAAATTTTGGAAAAATTATATGAAGAAGATATAGGAGCTGGGATGTATGCGTTGTACCCTTACGGTGGT
ATAATGGATGAGATTTCTGAATCAGCAATTCCATTCCCTCATCGAGCTGGAATCTTGTATGAGTTATGGT
ACATATGTAGTTGGGAGAAGCAAGAAGATAACGAAAAGCATCTAAACTGGATTAGAAATATTTATAACTT
CATGACTCCTTATGTGTCCAAAAATCCAAGATTGGCATATCTCAATTATAGAGACCTTGATATAGGAATA
AATGATCCCAAGAATCCAAATAATTACACACAAGCACGTATTTGGGGTGAGAAGTATTTTGGTAAAAATT
TTGACAGGCTAGTAAAAGTGAAAACCCTGGTTGATCCCAATAACTTTTTAGAAACGAACAAAGCATCCC
ACCTCTTCCACGGCATCGTCATTAA

>B1_KP970863.1
ATGAAGTGCTCAACATTCTCCTTTTGGTTTGTTTGCAAGATAATATTTTTCTTTTTCTCATTCAATATCC
AAACTTCCATTGCTAATCCTCGAGAAAACTTCCTTAAATGCTTCTCGCAATATATTCCCAATAATGCAAC
AAATCTAAAACTCGTATACACTCAAAACAACCCATTGTATATGTCTGTCCTAAATTCGACAATACACAAT
CTTAGATTCACCTCTGACACAACCCCAAAACCACTTGTTATCGTCACTCCTTCACATGTCTCTCATATCC
AAGGCACTATTCTATGCTCCAAGAAAGTTGGCTTGCAGATTCGAACTCGAAGTGGTGGTCATGATTCTGA
GGGCATGTCCTACATATCTCAAGTCCCATTTGTTATAGTAGACTTGAGAAACATGCGTTCAATCAAAATA
GATGTTCATAGCCAAACTGCATGGGTTGAAGCCGGAGCTACCCTTGGAGAAGTTTATTATTGGGTTAATG
AGAAAAATGAGAATCTTAGTTTGGCGGCTGGGTATTGCCCTACTGTTTGCGCAGGTGGACACTTTGGTGG
AGGAGGCTATGGACCATTGATGAGAAACTATGGCCTCGCGGCTGATAATATCATTGATGCACACTTAGTC
AACGTTCATGGAAAAGTGCTAGATCGAAAATCTATGGGGGAAGATCTCTTTTGGGCTTTACGTGGTGGTG
GAGCAGAAAGCTTCGGAATCATTGTAGCATGGAAAATTAGACTGGTTGCTGTCCCAAAGTCTACTATGTT
TAGTGTTAAAAAGATCATGGAGATACATGAGCTTGTCAAGTTAGTTAACAAATGGCAAAATATTGCTTAC
AAGTATGACAAAGATTTATTACTCATGACTCACTTCATAACTAGGAACATTACAGATAATCAAGGGAAGA
ATAAGACAGCAATACACACTTACTTCTCTTCAGTTTTCCTTGGTGGAGTGGATAGTCTAGTCGACTTGAT
GAACAAGAGTTTTCCTGAGTTGGGTATTAAAAAAACGGATTGCAGACAATTAAGCTGGATTGATACTATC
ATCTTCTATAGTGGTGTTGTAAATTACGACACTGATAATTTTAATAAGGAAATTTTGCTTGATAGATCCG
CTGGGCAGAACGGTGCTTTCAAGATTAAGTTAGACTACGTTAAGAAACCAATTCCAGAATCTGTATTTGT

CCAAATTTTGGAAAAATTATATGAAGAAGATATAGGAGCTGGGATGTATGCGTTGTACCCTTACGGTGGT
ATAATGGATGAGATTTCTGAATCAGCAATTCCATTCCCTCATCGAGCTGGAATCTTGTATGAGTTATGGT
ACATATGTAGTTGGGAGAAGCAAGAAGATAACGAAAAGCATCTAAACTGGATTAGAAATATTTATAACTT
CATGACTCCTTATGTGTCCAAAAATCCAAGATTGGCATATCTCAATTATAGAGACCTTGATATAGGAATA
AATGATCCCAAGAATCCAAATAATTACACACAAGCACGTATTTGGGGTGAGAAGTATTTTGGTAAAAATT
TTGACAGGCTAGTAAAAGTGAAAACCCTGGTTGATCCCAATAACTTTTTAGAAACGAACAAAGCATCCC
ACCTCAACCACGGCATCGTCATTAA

>B1_KP970865.1
ATGAAGTGCTCAACATTCTCCTTTTGGTTTGTTTGCAAGATAATATTTTTCTTTTTCTCATTCAATATCC
AAACTTCCATTGCTAATCCTCGAGAAAACTTCCTTAAATGCTTCTCGCAATATATTCCCAATAATGCAAC
AAATCTAAAACTCGTATACACTCAAAACAACCCATTGTATATGTCTGTCCTAAATTCGACAATACACAAT
CTTAGATTCACCTCTGACACAACCCCAAAACCACTTGTTATCGTCACTCCTTCACATGTCTCTCATATCC
AAGGCACTATTCTATGCTCCAAGAAAGTTGGCTTGCAGATTCGAACTCGAAGTGGTGGTCATGATTCTGA
GGGCATGTCCTACATATCTCAAGTCCCATTTGTTATAGTAGACTTGAGAAACATGCGTTCAATCAAAATA
GATGTTCATAGCCAAACTGCATGGGTTGAAGCCGGAGCTACCCTTGGAGAAGTTTATTATTGGGTTAATG
AGAAAAATGAGAATCTTAGTTTGGCGGCTGGGTATTGCCCTACTGTTTGCGCAGGTGGACACTTTGGTGG
AGGAGGCTATGGACCATTGATGAGAAACTATGGCCTCGCGGCTGATAATATCATTGATGCACACTTAGTC
AACGTTCATGGAAAAGTGCTAGATCGAAAATCTATGGGGGAAGATCTCTTTTGGGCTTTACGTGGTGGTG
GAGCAGAAAGCTTCGGAATCATTGTAGCATGGAAAATTAGACTGGTTGCTGTCCCAAAGTCTACTATGTT
TAGTGTTAAAAAGATCATGGAGATACATGAGCTTGTCAAGTTAGTTAACAAATGGCAAAATATTGCTTAC
AAGTATGACAAAGATTTATTACTCATGACTCACTTCATAACTAGGAACATTACAGATAATCAAGGGAAGA
ATAAGACAGCAATACACACTTACTTCTCTTCAGTTTTCCTTGGTGGAGTGGATAGTCTAGTCGACTTGAT
GAACAAGAGTTTTCCTGAGTTGGGTATTAAAAAAACGGATTGCAGACAATTAAGCTGGATTGATACTATC
ATCTTCTATAGTGGTGTTGTAAATTACGACACTGATAATTTTAATAAGGAAATTTTGCTTGATAGATCCG
CTGGGCAGAACGGTGCTTTCAAGATTAAGTTAGACTACGTTAAGAAACCAATTCCAGAATCTGTATTTGT
CCAAATTTTGGAAAAATTATATGAAGAAGATATAGGAGCTGGGATGTATGCGTTGTACCCTTACGGTGGT
ATAATGGATGAGATTTCTGAATCAGCAATTCCATTCCCTCATCGAGCTGGAATCTTGTATGAGTTATGGT
ACATATGTAGTTGGGAGAAGCAAGAAGATAACGAAAAGCATCTAAACTGGATTAGAAATATTTATAACTT
CATGACTCCTTATGTGTCCAAAAATCCAAGATTGGCATATCTCAATTATAGAGACCTTGATATAGGAATA
AATGATCCCAAGAATCCAAATAATTACACACAAGCACGTATTTGGGGTGAGAAGTATTTTGGTAAAAATT
TTGACAGGCTAGTAAAAGTGAAAACCCTGGTTGATCCCAATAACTTTTTAGAAACGAACAAAGCATCCC
ACCTCTACCACGGCATCGTCATTAA

>B1_KP970864.1
ATGAAGTGCTCAACATTCTCCTTTTGGTTTGTTTGCAAGATAATATTTTTCTTTTTCTCATTCAATATCC
AAACTTCCATTGCTAATCCTCGAGAAAACTTCCTTAAATGCTTCTCGCAATATATTCCCAATAATGCAAC
AAATCTAAAACTCGTATACACTCAAAACAACCCATTGTATATGTCTGTCCTAAATTCGACAATACACAAT
CTTAGATTCACCTCTGACACAACCCCAAAACCACTTGTTATCGTCACTCCTTCACATGTCTCTCATATCC
AAGGCACTATTCTATGCTCCAAGAAAGTTGGCTTGCAGATTCGAACTCGAAGTGGTGGTCATGATTCTGA
GGGCATGTCCTACATATCTCAAGTCCCATTTGTTATAGTAGACTTGAGAAACATGCGTTCAATCAAAATA
GATGTTCATAGCCAAACTGCATGGGTTGAAGCCGGAGCTACCCTTGGAGAAGTTTATTATTGGGTTAATG
AGAAAAATGAGAATCTTAGTTTGGCGGCTGGGTATTGCCCTACTGTTTGCGCAGGTGGACACTTTGGTGG
AGGAGGCTATGGACCATTGATGAGAAACTATGGCCTCGCGGCTGATAATATCATTGATGCACACTTAGTC
AACGTTCATGGAAAAGTGCTAGATCGAAAATCTATGGGGGAAGATCTCTTTTGGGCTTTACGTGGTGGTG
GAGCAGAAAGCTTCGGAATCATTGTAGCATGGAAAATTAGACTGGTTGCTGTCCCAAAGTCTACTATGTT
TAGTGTTAAAAAGATCATGGAGATACATGAGCTTGTCAAGTTAGTTAACAAATGGCAAAATATTGCTTAC
AAGTATGACAAAGATTTATTACTCATGACTCACTTCATAACTAGGAACATTACAGATAATCAAGGGAAGA
ATAAGACAGCAATACACACTTACTTCTCTTCAGTTTTCCTTGGTGGAGTGGATAGTCTAGTCGACTTGAT
GAACAAGAGTTTTCCTGAGTTGGGTATTAAAAAAACGGATTGCAGACAATTAAGCTGGATTGATACTATC
ATCTTCTATAGTGGTGTTGTAAATTACGACACTGATAATTTTAATAAGGAAATTTTGCTTGATAGATCCG
CTGGGCAGAACGGTGCTTTCAAGATTAAGTTAGACTACGTTAAGAAACCAATTCCAGAATCTGTATTTGT
CCAAATTTTGGAAAAATTATATGAAGAAGATATAGGAGCTGGGATGTATGCGTTGTACCCTTACGGTGGT
ATAATGGATGAGATTTCTGAATCAGCAATTCCATTCCCTCATCGAGCTGGAATCTTGTATGAGTTATGGT
ACATATGTAGTTGGGAGAAGCAAGAAGATAACGAAAAGCATCTAAACTGGATTAGAAATATTTATAACTT
CATGACTCCTTATGTGTCCAAAAATCCAAGATTGGCATATCTCAATTATAGAGACCTTGATATAGGAATA
AATGATCCCAAGAATCCAAATAATTACACACAAGCACGTATTTGGGGTGAGAAGTATTTTGGTAAAAATT

TTGACAGGCTAGTAAAAGTGAAAACCCTGGTTGATCCCAATAACTTTTTTAGAAACGAACAAAGCATCCC
ACCTCTTCCACGGCATCGTCATTAA

>B1_KJ469374.1
ATGAAGTGCTCAACATTCTCCTTTTGGTTTGTTTGCAAGATAATATTTTTCTTTTTCTCATTCAATATCCAAA
CTTCCATTGCTAATCCTCGAGAAAACTTCCTTAAATGCTTCTCGCAATATATTCCCAATAATGCAACAAATCT
AAAACTCGTATACACTCAAAACAACCCATTGTATATGTCTGTCCTAAATTCGACAATACACAATCTTAGATTC
AGCTCTGACACAACCCCAAAACCACTTGTTATCGTCACTCCTTCACATGTCTCTCATATCCAAGGCACTATTC
TATGCTCCAAGAAAGTTGGCTTGCAGATTCGAACTCGAAGTGGTGGTCATGATTCTGAGGGCATGTCCTACAT
ATCTCAAGTCCCATTTGTTATAGTAGACTTGAGAAACATGCGTTCAATCAAAATAGATGTTCATAGCCAAACT
GCATGGGTTGAAGCCGGAGCTACCCTTGGAGAAGTTTATTATTGGGTTAATGAGAAAAATGAGAGTCTTAGTT
TGGCTGCTGGGTATTGCCCTACTGTTTGCGCAGGTGGACACTTTGGTGGAGGAGGCTATGGACCATTGATGAG
AAGCTATGGCCTCGCGGCTGATAATATCATTGATGCACACTTAGTCAACGTTCATGGAAAAGTGCTAGATCGA
AAATCTATGGGGGAAGATCTCTTTTGGGCTTTACGTGGTGGTGGAGCAGAAAGCTTCGGAATCATTGTAGCAT
GGAAAATTAGACTGGTTGCTGTCCCAAAGTCTACTATGTTTAGTGTTAAAAAGATCATGGAGATACATGAGCT
TGTCAAGTTAGTTAACAAATGGCAAAATATTGCTTACAAGTATGACAAAGATTTATTACTCATGACTCACTTC
ATAACTAGGAACATTACAGATAATCAAGGGAAGAATAAGACAGCAATACACACTTACTTCTCTTCAGTTTTCC
TTGGTGGAGTGGATAGTCTAGTCGACTTGATGAACAAGAGTTTTCCTGAGTTGGGTATTAAAAAAACGGATTG
CAGACAATTGAGCTGGATTGATACTATCATCTTCTATAGTGGTGTTGTAAATTACGACACTGATAATTTTAAC
AAGGAAATTTTGCTTGATAGATCCGCTGGGCAGAACGGTGCTTTCAAGATTAAGTTAGACTACGTTAAGAAAC
CAATTCCAGAATCTGTATTTGTCCAAATTTTGGAAAAATTATATGAAGAAGATATAGGAGCTGGGATGTATGC
GTTGTACCCTTACGGTGGTATAATGGATGAGATTTCTGAATCAGCAATTCCATTCCCTCATCGAGCTGGAATC
TTGTATGAGTTATGGTACATATGTAGCTGGGAGAAGCAAGAAGATAACGAAAAGCATCTAAACTGGATTAGAA
ATATTTATAACTTCATGACTCCTTATGTGTCCCAAAATCCAAGATTGGCATATCTCAATTATAGAGACCTTGA
TATAGGAATAAATGATCCCAAGAATCCAAATAATTACACACAAGCACGTATTTGGGGTGAGAAGTATTTTGGT
AAAAATTTTGACAGGCTAGTAAAAGTGAAAACCCTGGTTGATCCCAATAATTTTTTTAGAAACGAACAAAGCA
TCCCACCTCTTCCACGGCATCGTCATTAA
>B1_KP970858.1
ATGAAGTGCTCAACATTCTCCTTTTGGTTTGTTTGCAAGATAATATTTTTCTTTTTCTCATTCAATATCC
AAACTTCCATTGCTAATCCTCGAGAAAACTTCCTTAAATGCTTCTCGCAATATATTCCCAATAATGCAAC
AAATCTAAAACTCGTATACACTCAAAACAACCCATTGTATATGTCTGTCCTAAATTCGACAATACACAAT
CTTAGATTCAGCTCTGACACAACCCCAAAACCACTTGTTATCGTCACTCCTTCACATGTCTCTCATATCC
AAGGCACTATTCTATGCTCCAAGAAAGTTGGCTTGCAGATTCGAACTCGAAGTGGTGGTCATGATTCTGA
GGGCATGTCCTACATATCTCAAGTCCCATTTGTTATAGTAGACTTGAGAAACATGCGTTCAATCAAAATA
GATGTTCATAGCCAAACTGCATGGGTTGAAGCCGGAGCTACCCTTGGAGAAGTTTATTATTGGGTTAATG
AGAAAAATGAGAGTCTTAGTTTGGCTGCTGGGTATTGCCCTACTGTTTGCGCAGGTGGACACTTTGGTGG
AGGAGGCTATGGACCATTGATGAGAAGCTATGGCCTCGCGGCTGATAATATCATTGATGCACACTTAGTC
AACGTTCATGGAAAAGTGCTAGATCGAAAATCTATGGGGGAAGATCTCTTTTGGGCTTTACGTGGTGGTG
GAGCAGAAAGCTTCGGAATCATTGTAGCATGGAAAATTAGACTGGTTGCTGTCCCAAAGTCTACTATGTT
TAGTGTTAAAAAGATCATGGAGATACATGAGCTTGTCAAGTTAGTTAACAAATGGCAAAATATTGCTTAC
AAGTATGACAAAGATTTATTACTCATGACTCACTTCATAACTAGGAACATTACAGATAATCAAGGGAAGA
ATAAGACAGCAATACACACTTACTTCTCTTCAGTTTTCCTTGGTGGAGTGGATAGTCTAGTCGACTTGAT
GAACAAGAGTTTTCCTGAGTTGGGTATTAAAAAAACGGATTGCAGACAATTGAGCTGGATTGATACTATC
ATCTTCTATAGTGGTGTTGTAAATTACGACACTGATAATTTTAACAAGGAAATTTTGCTTGATAGATCCG
CTGGGCAGAACGGTGCTTTCAAGATTAAGTTAGACTACGTTAAGAAACCAATTCCAGAATCTGTATTTGT
CCAAATTTTGGAAAAATTATATGAAGAAGATATAGGAGCTGGGATGTATGCGTTGTACCCTTACGGTGGT
ATAATGGATGAGATTTCTGAATCAGCAATTCCATTCCCTCATCGAGCTGGAATCTTGTATGAGTTATGGT
ACATATGTAGCTGGGAGAAGCAAGAAGATAACGAAAAGCATCTAAACTGGATTAGAAATATTTATAACTT
CATGACTCCTTATGTGTCCCAAAATCCAAGATTGGCATATCTCAATTATAGAGACCTTGATATAGGAATA
AATGATCCCAAGAATCCAAATAATTACACACAAGCACGTATTTGGGGTGAGAAGTATTTTGGTAAAAATT
TTGACAGGCTAGTAAAAGTGAAAACCCTGGTTGATCCCAATAATTTTTTTAGAAACGAACAAAGCATCCC
ACCTCTTCCACGGCATCGTCATTAA

>B1_MG996434.1
TTCAATATCCAAACTTCCATTGCTAATCCTCGAGAAAACTTCCTTAAATGCTTCTCGCAATATATTCCCAATA
ATGCAACAAATCTAAAACTCGTATACACTCAAAACAACCCATTGTATATGTCTGTCCTAAATTCGACAATACA
CAATCTTAGATTCAGCTCTGACACAACCCCAAAACCACTTGTTATCGTCACTCCTTCACATGTCTCTCATATC
CAAGGCACTATTCTATGCTCCAAGAAAGTTGGCTTGCAGATTCGAACTCGAAGTGGTGGTCATGATTCTGAGG

GCATGTCCTACATATCTCAAGTCCCATTTGTTATAGTAGACTTGAGAAACATGCGTTCAATCAAAATAGATGT
TCATAGCCAAACTGCATGGGTTGAAGCCGGAGCTACCCTTGGAGAAGTTTATTATTGGGTTAATGAGAAAAAT
GAGAGTCTTAGTTTGGCTGCTGGGTATTGCCCTACTGTTTGCGCAGGTGGACACTTTGGTGGAGGAGGCTATG
GACCATTGATGAGAAGCTATGGCCTCGCGGCTGATAATATCATTGATGCACACTTAGTCAACGTTCATGGAAA
AGTGCTAGATCGAAAATCTATGGGGGAAGATCTCTTTTGGGCTTTACGTGGTGGTGGAGCAGAAAGCTTCGGA
ATCATTGTAGCATGGAAAATTAGACTGGTTGCTGTCCCAAAGTCTACTATGTTTAGTGTTAAAAAGATCATGG
AGATACATGAGCTTGTCAAGTTAGTTAACAAATGGCAAAATATTGCTTACAAGTATGACAAAGATTTATTACT
CATGACTCACTTCATAACTAGGAACATTACAGATAATCAAGGGAAGAATAAGACAGCAATACACACTTACTTC
TCTTCAGTTTTCCTTGGTGGAGTGGATAGTCTAGTCGACTTGATGAACAAGAGTTTTCCTGAGTTGGGTATTA
AAAAAACGGATTGCAGACAATTGAGCTGGATTGATACTATCATCTTCTATAGTGGTGTTGTAAATTACGACAC
TGATAATTTTAACAAGGAAATTTTGCTTGATAGATCCGCTGGGCAGAACGGTGCTTTCAAGATTAAGTTAGAC
TACGTTAAGAAACCAATTCCAGAATCTGTATTTGTCCAAATTTTGGAAAAATTATATGAAGAAGATATAGGAG
CTGGGATGTATGCGTTGTACCCTTACGGTGGTATAATGGATGAGATTTCTGAATCAGCAATTCCATTCCCTCA
TCGAGCTGGAATCTTGTATGAGTTATGGTACATATGTAGCTGGGAGAAGCAAGAAGATAACGAAAAGCATCTA
AACTGGATTAGAAATATTTATAACTTCATGACTCCTTATGTGTCCCAAAATCCAAGATTGGCATATCTCAATT
ATAGAGACCTTGATATAGGAATAAATGATCCCAAGAATCCAAATAATTACACACAAGCACGTATTTGGGGTGA
GAAGTATTTTGGTAAAAATTTTGACAGGCTAGTAAAAGTGAAAACCCTGGTTGATCCCAA
>B1_KP970861.1
ATGAAGTGCTCAACATTCTCCTTTTGGTTTGTTTGCAAGATAATATTTTTCTTTTTCTCATTCAATATCC
AAACTTCCATTGCTAATCCTCGAGAAAACTTCCTTAAATGCTTCTCGCAATATATTCCCAATAATGCAAC
AAATCTAAAACTCGTATACACTCAAAACAACCCATTGTATATGTCTGTCCTAAATTCGACAATACACAAT
CTTAGATTCAGCTCTGACACAACCCCAAAACCACTTGTTATCGTCACTCCTTCACATGTCTCTCATATCC
AAGGCACTATTCTATGCTCCAAGAAAGTTGGCTTGCAGATTCGAACTCGAAGTGGTGGTCATGATTCTGA
GGGCATGTCCTACATATCTCAAGTCCCATTTGTTATAGTAGACTTGAGAAACATGCGTTCAATCAAAATA
GATGTTCATAGCCAAACTGCATGGGTTGAAGCCGGAGCTACCCTTGGAGAAGTTTATTATTGGGTTAATG
AGAAAAATGAGAGTCTTAGTTTGGCTGCTGGGTATTGCCCTACTGTTTGCGCAGGTGGACACTTTGGTGG
AGGAGGCTATGGACCATTGATGAGAAGCTATGGCCTCGCGGCTGATAATATCATTGATGCACACTTAGTC
AACGTTCATGGAAAAGTGCTAGATCGAAAATCTATGGGGGAAGATCTCTTTTGGGCTTTACGTGGTGGTG
GAGCAGAAAGCTTCGGAATCATTGTAGCATGGAAAATTAGACTGGTTGCTGTCCCAAAGTCTACTATGTT
TAGTGTTAAAAAGATCATGGAGATACATGAGCTTGTCAAGTTAGTTAACAAATGGCAAAATATTGCTTAC
AAGTATGACAAAGATTTATTACTCATGACTCACTTCATAACTAGGAACATTACAGATAATCAAGGGAAGA
ATAAGACAGCAATACACACTTACTTCTCTTCAGTTTTCCTTGGTGGAGTGGATAGTCTAGTCGACTTGAT
GAACAAGAGTTTTCCTGAGTTGGGTATTAAAAAAACGGATTGCAGACAATTGAGCTGGATTGATACTATC
ATCTTCTATAGTGGTGTTGTAAATTACGACACTGATAATTTTAACAAGGAAATTTTGCTTGATAGATCCG
CTGGGCAGAACGGTGCTTTCAAGATTAAGTTAGACTACGTTAAGAAACCAATTCCAGAATCTGTATTTGT
CCAAATTTTGGAAAAATTATATGAAGAAGATATAGGAGCTGGGATGTATGCGTTGTACCCTTACGGTGGT
ATAATGGATGAGATTTCTGAATCAGCAATTCCATTCCCTCATCGAGCTGGAATCTTGTATGAGTTATGGT
ACATATGTAGCTGGGAGAAGCAAGAAGATAACGAAAAGCATCTAAACTGGATTAGAAATATTTATAACTT
CATGACTCCTTATGTGTCCCAAAATCCAAGATTGGCATATCTCAATTATAGAGACCTTGATATAGGAATA
AATGATCCCAAGAATCCAAATAATTACACACAAGCACGTATTTGGGGTGAGAAGTATTTTGGTAAAAATT
TTGACAGGCTAGTAAAAGTGAAAACCCTGGTTGATCCCAATAATTTTTTTAGAAACGAACAAAGCATCCC
ACCTCTACCACGGCATCGTCATTAA

>B1_MG996439.1
TTCAATATCCAAACTTCCATTGCTAATCCTCGAGAAAACTTCCTTAAATGCTTCTCGCAATATATTCCCAATA
ATGCAACAAATCTAAAACTCGTATACACTCAAAACAACCCATTGTATATGTCTGTCCTAAATTCGACAATACA
CAATCTTAGATTCAGCTCTGACACAACCCCAAAACCACTTGTTATCGTCACTCCTTCACATGTCTCTCATATC
CAAGGCACTATTCTATGCTCCAAGAAAGTTGGCTTGCAGATTCGAACTCGAAGTGGTGGTCATGATTCTGAGG
GCATGTCCTACATATCTCAAGTCCCATTTGTTATAGTAGACTTGAGAAACATGCGTTCAATCAAAATAGATGT
TCATAGCCAAACTGCATGGGTTGAAGCCGGAGCTACCCTTGGAGAAGTTTATTATTGGGTTAATGAGAAAAAT
GAGAGTCTTAGTTTGGCTGCTGGGTATTGCCCTACTGTTTGCGCAGGTGGACACTTTGGTGGAGGAGGCTATG
GACCATTGATGAGAAGCTATGGCCTCGCGGCTGATAATATCATTGATGCACACTTAGTCAACGTTCATGGAAA
AGTGCTAGATCGAAAATCTATGGGGGAAGATCTCTTTTGGGCTTTACGTGGTGGTGGAGCAGAAAGCTTCGGA
ATCATTGTAGCATGKAAAATTAGACTGGTTGCTGTCCCAAAGTCTACTATGTTTAGTGTTAAAAAGATCATGG
AGATACATGAGCTTGTCAAGTTAGTTAACAAATGGCAAAATATTGCTTACAAGTATGACAAAGATTTATTACT
CATGACTCACTTCATAACTAGGAACATTACAGATAATCAAGGGAAGAATAAGACAGCAATACACACTTACTTC
TCTTCAGTTTTCCTTGGTGGAGTGGATAGTCTAGTCGACTTGATGAACAAGAGTTTTCCTGAGTTGGGTATTA
AAAAAACGGATTGCAGACAATTGAGCTGGATTGATACTATCATCTTCTATAGTGGTGTTGTAAATTACGACAC

TGATAATTTTAACAAGGAAATTTTGCTTGATAGATCCGCTGGGCAGAACGGTGCTTTCAAGATTAAGTTAGAC
TACGTTAAGAAACCAATTCCAGAATCTGTATTTGTCCAAATTTTGGAAAAATTATATGAAGAAGATATAGGAG
CTGGGATGTATGCGTTGTACCCTTACGGTGGTATAATGGATGAGATTTCTGAATCAGCAATTCCATTCCCTCA
TCGAGCTGGAATCTTGTATGAGTTATGGTACATATGTAGCTGGGAGAAGCAAGAAGATAACGAAAAGCATCTA
AACTGGATTAGAAATATTTATAACTTCATGACTCCTTATGTGTCCCAAAATCCAAGATTGGCATATCTCAATT
ATAGAGACCTTGATATAGGAATAAATGATCCCAAGAATCCAAATAATTACACACAAGCACGTATTTGGGGTGA
GAAGTATTTTGGTAAAAATTTTGACAGGCTAGTAAAAGTGAAAACCCTGGTTGAT
>B1_KP970860.1
ATGAAGTGCTCAACATTCTCCTTTTGGTTTGTTTGCAAGATAATATTTTTCTTTTTCTCATTCAATATCC
AAACTTCCATTGCTAATCCTCGAGAAAACTTCCTTAAATGCTTCTCGCAATATATTCCCAATAATGCAAC
AAATCTAAAACTCGTATACACTCAAAACAACCCATTGTATATGTCTGTCCTAAATTCGACAATACACAAT
CTTAGATTCAGCTCTGACACAACCCCAAAACCACTTGTTATCGTCACTCCTTCACATGTCTCTCATATCC
AAGGCACTATTCTATGCTCCAAGAAAGTTGGCTTGCAGATTCGAACTCGAAGTGGTGGTCATGATTCTGA
GGGCATGTCCTACATATCTCAAGTCCCATTTGTTATAGTAGACTTGAGAAACATGCGTTCAATCAAAATA
GATGTTCATAGCCAAACTGCATGGGTTGAAGCCGGAGCTACCCTTGGAGAAGTTTATTATTGGGTTAATG
AGAAAAATGAGAGTCTTAGTTTGGCTGCTGGGTATTGCCCTACTGTTTGCGCAGGTGGACACTTTGGTGG
AGGAGGCTATGGACCATTGATGAGAAGCTATGGCCTCGCGGCTGATAATATCATTGATGCACACTTAGTC
AACGTTCATGGAAAAGTGCTAGATCGAAAATCTATGGGGGAAGATCTCTTTTGGGCTTTACGTGGTGGTG
GAGCAGAAAGCTTCGGAATCATTGTAGCATGGAAAATTAGACTGGTTGCTGTCCCAAAGTCTACTATGTT
TAGTGTTAAAAAGATCATGGAGATACATGAGCTTGTCAAGTTAGTTAACAAATGGCAAAATATTGCTTAC
AAGTATGACAAAGATTTATTACTCATGACTCACTTCATAACTAGGAACATTACAGATAATCAAGGGAAGA
ATAAGACAGCAATACACACTTACTTCTCTTCAGTTTTCCTTGGTGGAGTGGATAGTCTAGTCGACTTGAT
GAACAAGAGTTTTCCTGAGTTGGGTATTAAAAAAACGGATTGCAGACAATTGAGCTGGATTGATACTATC
ATCTTCTATAGTGGTGTTGTAAATTACGACACTGATAATTTTAACAAGGAAATTTTGCTTGATAGATCCG
CTGGGCAGAACGGTGCTTTCAAGATTAAGTTAGACTACGTTAAGAAACCAATTCCAGAATCTGTATTTGT
CCAAATTTTGGAAAAATTATATGAAGAAGATATAGGAGCTGGGATGTATGCGTTGTACCCTTACGGTGGT
ATAATGGATGAGATTTCTGAATCAGCAATTCCATTCCCTCATCGAGCTGGAATCTTGTATGAGTTATGGT
ACATATGTAGCTGGGAGAAGCAAGAAGATAACGAAAAGCATCTAAACTGGATTAGAAATATTTATAACTT
CATGACTCCTTATGTGTCCCAAAATCCAAGATTGGCATATCTCAATTATAGAGACCTTGATATAAGAATA
AATGATCCCAAGAATCCAAATAATTACACACAAGCACGTATTTGGGGTGAGAAGTATTTTGGTAAAAATT
TTGACAGGCTAGTAAAAGTGAAAACCCTGGTTGATCCCAATAATTTTTTTAGAAACGAACAAAGCATCCC
ACCTCTTCCACGGCATCGTCATTAA

>B1_KP970857.1
ATGAAGTGCTCAACATTCTCCTTTTGGTTTGTTTGCAAGATAATATTTTTCTTTTTCTCATTCAATATCC
AAACTTCCATTGCTAATCCTCGAGAAAACTTCCTTAAATGCTTCTCGCAATATATTCCCAATAATGCAAC
AAATCTAAAACTCGTATACACTCAAAACAACCCATTGTATATGTCTGTCCTAAATTCGACAATACACAAT
CTTAGATTCAGCTCTGACACAACCCCAAAACCACTTGTTATCGTCACTCCTTCACATGTCTCTCATATCC
AAGGCACTATTCTATGCTCCAAGAAAGTTGGCTTGCAGATTCGAACTCGAAGTGGTGGTCATGATTCTGA
GGGCATGTCCTACATATCTCAAGTCCCATTTGTTATAGTAGACTTGAGAAACATGCGTTCAATCAAAATA
GATGTTCATAGCCAAACTGCATGGGTTGAAGCCGGAGCTACCCTTGGAGAAGTTTATTATTGGGTTAATG
AGAAAAATGAGAGTCTTAGTTTGGCTGCTGGGTATTGCCCTACTGTTTGCGCAGGTGGACACTTTGGTGG
AGGAGGCTATGGACCATTGATGAGAAGCTATGGCCTCGCGGCTGATAATATCATTGATGCACACTTAGTC
AACGTTCATGGAAAAGTGCTAGATCGAAAATCTATGGGGGAAGATCTCTTTTGGGCTTTACGTGGTGGTG
GAGCAGAAAGCTTCGGAATCATTGTAGCATGGAAAATTAGACTGGTTGCTGTCCCAAAGTCTACTATGTT
TAGTGTTAAAAAGATCATGGAGATACATGAGCTTGTCAAGTTAGTTAACAAATGGCAAAATATTGCTTAC
AAGTATGACAAAGATTTATTACTCATGACTCACTTCATAACTAGGAACATTACAGATAATCAAGGGAAGA
ATAAGACAGCAATACACACTTACTTCTCTTCAGTTTTCCTTGGTGGAGTGGATAGTCTAGTCGACTTGAT
GAACAAGAGTTTTCCTGAGTTGGGTATTAAAAAAACGGATTGCAGACAATTGAGTTGGATTGATACTATC
ATCTTCTATAGTGGTGTTGTAAATTACGACACTGATAATTTTAACAAGGAAATTTTGCTTGATAGATCCG
CTCGGCAGAACGGTGCTTTCAAGATTAAGTTAGACTACGTTAAGAAACCAATTCCAGAATCTGTATTTGT
CCAAATTTTGGAAAAATTATATGAAGAAGATATAGGAGCTGGGATGTATGCGTTGTACCCTTACGGTGGT
ATAATGGATGAGATTTCTGAATCAGCAATTCCATTCCCTCATCGAGCTGGAATCTTGTATGAGTTATGGT
ACATATGTAGCTGGGAGAAGCAAGAAGATAACGAAAAGCATCTAAACTGGATTAGAAATATTTATAACTT
CATGACTCCTTATGTGTCCCAAAATCCAAGATTGGCATATCTCAATTATAGAGACCTTGATATAGGAATA
AATGATCCCAAGAATCCAAATAATTACACACAAGCACGTATTTGGGGTGAGAAGTATTTTGGTAAAAATT
TTGACAGGCTAGTAAAAGTGAAAACCCTGGTTGATCCCAATAATTTTTTTAGAAACGAACAAAGCATCCC
ACCTCTTCCACGGCATCGTCATTAA

>B1_MG996436.1
TTCAATATCCAAACTTCCATTGCTAATCCTCGAGAAAACTTCCTTAAATGCTTCTCGCAATATATTCCCAATA
ATGCAACAAATCTAAAACTCGTATACACTCAAAACAACCCATTGTATATGTCTGTCCTAAATTCGACAATACA
CAATCTTAGATTCAGCTCTGACACAACCCCAAAACCACTTGTTATCGTCACTCCTTCACATGTCTCTCATATC
CAAGGCACTATTCTATGCTCCAAGAAAGTTGGCTTGCAGATTCGAACTCGAAGTGGTGGTCATGATTCTGAGG
GCATGTCCTACATATCTCAAGTCCCATTTGTTATAGTAGACTTGAGAAACATGCGTTCAATCAAAATAGATGT
TCATAGCCAAACTGCATGGGTTGAAGCCGGAGCTACCCTTGGAGAAGTTTATTATTGGGTTAATGAGAAAAAT
GAGAGTCTTAGTTTGGCTGCTGGGTATTGCCCTACTGTTTGCGCAGGTGGACACTTTGGTGGAGGAGGCTATG
GACCATTGATGAGAAGCTATGGCCTCGCGGCTGATAATATCATTGATGCACACTTAGTCAACGTTCATGGAAA
AGTGCTAGATCGAAAATCTATGGGGGAAGATCTCTTTTGGGCTTTACGTGGTGGTGGAGCAGAAAGCTTCGGA
ATCATTGTAGCATGGAAAATTAGACTGGTTGCTGTCCCAAAGTCTACTATGTTTAGTGTTAAAAAGATCATGG
AGATACATGAGCTTGTCAAGTTAGTTAACAAATGGCAAAATATTGCTTACAAGTATGACAAAGATTTATTACT
CATGACTCACTTCATAACTAGGAACATTACAGATAATCAAGGGAAGAATAAGACAGCAATACACACTTACTTC
TCTTCAGTTTTCCTTGGTGGAGTGGATAGTCTAGTCGACTTGATGAACAAGAGTTTTCCTGAGTTGGGTATTA
AAAAAACGGATTGCAGACAATTGAGCTGGATTGATACTATCATCTTCTATAGTGGTGTTGTAAATTACGACAC
TGATAATTTTAACAAGGAAATTTTGCTTGATAGATCCGCTGGGCAGAACGGTGCTTTCAAGATTAAGTTAGAC
TACGTTAAGAAACCAATTCCAGAATCTGTATTTGTCCAAATTTTGGAAAAATTATATGAAGAAGATATAGGAG
CTGGGATGTATGCGTTGTACCCTTACGGTGGTATAATGGATGAGATTTCTGAATCAGCAATTCCATTCCCTCA
TCGAGCTGGAATCTTGTATGAGTTATGGTACATATGTAGCTGGGAGAAGCAAGAAGATAACGAAAAGCATCTA
AACTGGATTAGAAATATTTATAACTTCATGACTCCTTATGTGTCCCAAAATCCAAGATTGGCATATCTCAATT
ATAGAGACCTTGATATAGGAATAAMTGATCCCAAGMATCCAMATMATTWCACMCAMRCACGTATTTGGGSTSA
GAMGTATTTTSGTMAMAATTTTGACAGGCTAGTAAMAGTGMAAACCCTGGTGC
>B1_MG996435.1
CAAACTTCCATTGCTAATCCTCGAGAAAACTTCCTTAAATGCTTCTCGCAATATATTCCCAATAATGCAACAA
ATCTAAAACTCGTATACACTCAAAACAACCCATTGTATATGTCTGTCCTAAATTCGACAATACACAATCTTAG
ATTCAGCTCTGACACAACCCCAAAACCACTTGTTATCGTCACTCCTTCACATGTCTCTCATATCCAAGGCACT
ATTCTATGCTCCAAGAAAGTTGGCTTGCAGATTCGAACTCGAAGTGGTGGTCATGATTCTGAGGGCATGTCCT
ACATATCTCAAGTCCCATTTGTTATAGTAGACTTGAGAAACATGCGTTCAATCAAAATAGATGTTCATAGCCA
AACTGCATGGGTTGAAGCCGGAGCTACCCTTGGAGAAGTTTATTATTGGGTTAATGAGAAAAATGAGAGTCTT
AGTTTGGCTGCTGGGTATTGCCCTACTGTTTGCGCAGGTGGACACTTTGGTGGAGGAGGCTATGGACCATTGA
TGAGAAGCTATGGCCTCGCGGCTGATAATATCATTGATGCACACTTAGTCAACGTTCATGGAAAAGTGCTAGA
TCGAAAATCTATGGGGGAAGATCTCTTTTGGGCTTTACGTGGTGGTGGAGCAGAAAGCTTCGGAATCATTGTA
GCATGGAAAATTAGACTGGTTGCTGTCCCAAAGTCTACTATGTTTAGTGTTAAAAAGATCATGGAGATACATG
AGCTTGTCAAGTTAGTTAACAAATGGCAAAATATTGCTTACAAGTATGACAAAGATTTATTACTCATGACTCA
CTTCATAACTAGGAACATTACAGATAATCAAGGGAAGAATAAGACAGCAATACACACTTACTTCTCTTCAGTT
TTCCTTGGTGGAGTGGATAGTCTAGTCGACTTGATGAACAAGAGTTTTCCTGAGTTGGGTATTAAAAAAACGG
ATTGCAGACAATTGAGCTGGATTGATACTATCATCTTCTATAGTGGTGTTGTAAATTACGACACTGATAATTT
TAACAAGGAAATTTTGCTTGATAGATCCGCTGGGCAGAACGGTGCTTTCAAGATTAAGTTAGACTACGTTAAG
AAACCAATTCCAGAATCTGTATTTGTCCAAATTTTGGAAAAATTATATGAAGAAGATATAGGAGCTGGGATGT
ATGCGTTGTACCCTTACGGTGGTATAATGGATGAGATTTCTGAATCAGCAATTCCATTCCCTCATCGAGCTGG
AATCTTGTATGAGTTATGGTACAYATGYAGCTGGGAGAAGCAAGAAGATMACGAAAAGCATCTAAACTGGATT
AGAAATATTTATAACTTCATGACTCCTTATGTGTCCCAAAATCCAAGATTGGCATATCTCAATTATAGAGACC
TTGATATAGGAATAAATGATCCCAAGCATCCAAATAATCCCACCCACGCACGTATCAGGGCTCAGAAGTATTT
CCGTCAAAATTTTGACAAGCTAGTAA
>B2_Jilong_CM022965.1_91710216
TTACTCATGACTCACTTCATAACTAGGAATATTATAGATAATCAAGGGAAGAATAAGACAACAGTACACACTT
ACTTCTCTTTAGTTTTCATTGGTGGAGTGGATAGTCTAGTCGACTTAATGAACAAGAGTTTTCCTGAGTTTGG
CATTAAGAAAGATTGCAAACAATTGAGCTGGATTGATACTATCATCTTCTATAGCGGTGTTGTAAATTACGGC
ACTGATAATTTTAATAAGGAAATTTTGCTTGATAGATCAGCTGGGCAGAACGGTGCTTTCAAGATTAAGTTAG
ACTACGTTAAGAAACCAATTCCAGAATCTGCGTTTGTCAAAATTTTGGAAAAATTATATGAAGAAGATGAAGG
AGTTGGGATGTATGCGTTGTACACTTACGGTTGTATAATGGATGAGATTTCAGAATCAGCAATTCCATTCCCT
CATCGAGTTGGAATCATGTATGAATTATGGTACACATGTAGCTGGGAGAAGCACGAAGATAAAGAAAAGTATC
TAAACTGGATTCGAAATGTTGATAACTTCATGACTCCTTATGTGTCCCAAAATCCAAGATTGACATATCTCAA
TTATAGACACCTTGATATAGGAATAAATGATCCCAAGAGTCAAAATAATTACACACAAGCATGTATTTGGGGT
GAGAAGTATTTTGGTAAAAATTTTGACAGGCTAGTAAAAGTGAAAACCCTGGTTGATCTCGATAATTTTTTTA
GAAATGAACAAAGCATCCCACCTCTTCCACCGCATCGTCATAAATGA
>B2_Jamaican_Lion_JAATIP010000026.1_F8388_024902

ATGAAGTACTAAACATTCTCCTTTTGGTTTGTTTGCAAGATAATATTTTTTCTCTCATTCAATATCCAAACTT
CAATTACTAATCCTCGAGAAAACTTCCTTAAATGCTTCTCGCAATATATTCCCACCAATGTAACAAATCTAAA
ACTTACTCAAAACAACCAATTGTATATGCCTGCCCAAAATTCGACAATACACAATCTTAGATTCACCTCTGAC
ACAACCCCAAAACCATTTGTTATCGTCACTCCTTCACATGTCTCCCATATCCAAGGCACTATTCTATGCTCTA
AGAAAATTGGTTTGCAAATTCGAACTCGAAGCGGTGGTCATGATTCTGAAGGCATGTCCTACATATCTCAAGT
CCCATTTGTTATAGTAGACTTGAGAAACATGCATTCAATCAAAATAGATGTTCATAGCCAAACTACATGGGTT
GAAGCCGGAGCTACCCTTGGAGAAGTTTATGATTGGGTTAATGAGAAAAATGAGAATCTTAGTTTGGGTGCTG
GGTATTGCCCTACTGTTTGCGCAGCTGGACACTTTGGTGGAGGAGGCTATGGACCATTGATGTGAAATTATGG
CCTTGCGGCTGATAATATCGTTGATGCACACTTAGTCAACATTGATGGAAAAGTGCTAGATCGAAAATATATG
GGAGAAGATCTCTTTTGGGCTATACGTTGTGGTGGAGGAGAAAGCTTCAGAATCATTGTAGCATGGAAAATTA
GATTGGTTGCTGTCCCAACAAAGTCTACTATGTTTAGTGTTAAAAAGATCAAGGAGATACATGAGCTTGTGAA
GTTAGTTAACAAGTGGCAAAACATTTCTTACAAGTATGACAAAGATTTATTACTCATGACTCACTTCATAACT
AGGAATATTATAGATAATCAAGGGAAGAATAAGACAACAGTACACACTTACTTCTCTTTAGTTTTCCTTGGTG
GAGTGGATAGTCTAGTCGACTTAATGAACAAGAGTTTTCCTGAGTTTGGCATTGAAACAGATTGCAAACAATT
GAGCTGGATTGATACTATCATCTTCTATAGCGGTGTTGTAAATTACGGCACTAATTTTAATAAGGAAATTTTG
CTTGATAGATCAGCTGGGCAGAACGGTGCTTTCAAGATTAAGTTAGACTACGTTAAGAAACCAATTCCAGAAT
CTGCGTTTGTCAAAATTTTGGAAAAATTATATGAAGAAGATGAAGGAGTTGGGATGTATGCGTTGTACACTTA
CGGTTGTATAATGGATGAGATTTCAGAATCAGCAATTCCATTCCCTCATCGAGTTGGAATCATGTATGAATTA
TGGTACACATGTAACTGGGAGAAGCACGAAGATAAAGAAAAGTATCTAAACTGGATTCGAAATGTTGATAACT
TCATGACTCCTTATGTGTCCCAAAATCCAAGATTGACATATCTCAATTATAGACACCTTGATATAGGAATAAA
TGATCCCAAGAGTCAAAATAATTACACACAAGCATGTATTTGGGGTGAGAAGTATTTTGGTAAAAATTTTGAC
AGGCTAGTAAAAGTGAAAACCCTGGTTGATCTCGATAATTTTTTTAGAAATGAACAAAGCATCCCACCTCTTC
CACCGCATCGTCATAAATGA
>B2_Purple_Kush__CM010797.2_28773351
ATGAAGTACTAAACATTCTCCTTTTGGTTTGTTTGCAAGATAATATTTTTTCTCTCATTCAATATCCAAACTT
CAATTACTAATCCTCGAGAAAACTTCCTTAAATGCTTCTCGCAATATATTCCCACCAATGTAACAAATCTAAA
ACTTACTCAAAACAACCAATTGTATATGCCTGCCCAAAATTCGACAATACACAATCTTAGATTCACCTCTGAC
ACAACCCCAAAACCATTTGTTATCGTCACTCCTTCACATGTCTCCCATATCCAAGGCACTATTCTATGCTCTA
AGAAAATTGGTTTGCAAATTCGAACTCGAAGCGGTGGTCATGATTCTGAAGGCATGTCCTACATATCTCAAGT
CCCATTTGTTATAGTAGACTTGAGAAACATGCATTCAATCAAAATAGATGTTCATAGCCAAACTACATGGGTT
GAAGCCGGAGCTACCCTTGGAGAAGTTTATGATTGGGTTAATGAGAAAAATGAGAATCTTAGTTTGGGTGCTG
GGTATTGCCCTACTGTTTGCGCAGCTGGACACTTTGGTGGAGGAGGCTATGGACCATTGATGTGAAATTATGG
CCTTGCGGCTGATAATATCGTTGATGCACACTTAGTCAACATTGATGGAAAAGTGCTAGATCGAAAATATATG
GGAGAAGATCTCTTTTGGGCTATACGTTGTGGTGGAGGAGAAAGCTTCAGAATCATTGTAGCATGGAAAATTA
GATTGGTTGCTGTCCCAACAAAGTCTACTATGTTTAGTGTTAAAAAGATCAAGGAGATACATGAGCTTGTGAA
GTTAGTTAACAAGTGGCAAAACATTTCTTACAAGTATGACAAAGATTTATTACTCATGACTCACTTCATAACT
AGGAATATTATAGATAATCAAGGGAAGAATAAGACAACAGTACACACTTACTTCTCTTTAGTTTTCCTTGGTG
GAGTGGATAGTCTAGTCGACTTAATGAACAAGAGTTTTCCTGAGTTTGGCATTGAAACAGATTGCAAACAATT
GAGCTGGATTGATACTATCATCTTCTATAGCGGTGTTGTAAATTACGGCACTGATAATTTTAATAAGGAAATT
TTGCTTGATAGATCAGCTGGGCAGAACGGTGCTTTCAAGATTAAGTTAGACTACGTTAAGAAACCAATTCCAG
AATCTGCGTTTGTCAAAATTTTGGAAAAATTATATGAAGAAGATGAAGGAGTTGGGATGTATGCGTTGTACAC
TTACGGTTGTATAATGGATGAGATTTCAGAATCAGCAATTCCATTCCCTCATCGAGTTGGAATCATGTATGAA
TTATGGTACACATGTAGCTGGGAGAAGCACGAAGATAAAGAAAAGTATCTAAACTGGATTCGAAATGTTGATA
ACTTCATGACTCCTTATGTGTCCCAAAATCCAAGATTGACATATCTCAATTATAGACACCTTGATATAGGAAT
AAATGATCCCAAGAGTCAAAATAATTACACACAAGCATGTATTTGGGGTGAGAAGTATTTTGGTAAAAATTTT
GACAGGCTAGTAAAAGTGAAAACCCTGGTTGATCTCGATAATTTTTTTAGAAATGAACAAAGCATCCCACCTC
TTCCACCGCATCGTCATAAATGA
>B2_Jilong_CM022965.1_91699899
ATGAAGTACTAAACATTCTCCTTTTGGTTTGTTTGCAAGATAATATTTTTTCTCTCATTCAATATCCAAACTT
CAATTACTAATCCTCGAGAAAACTTCCTTAAATGCTTCTCGCAATATATTCCCACCAATGTAACAAATCTAAA
ATACACTCAAAACAACCAATTGTATATGCCTGCCCAAAATTCGACAATACACAATCTTAGATTCACCTCTGAC
ACAACCCCAAAACCATTTGTTATCGTCACTCCTTCACATGTCTCCCATATCCAAGGCACTATTCTATGCTCTA
AGAAAATTGGTTTGCAAATTCGAACTCGAAGCGGTGGTCATGATTCTGAAGGCATGTCCTACATATCTCAAGT
CCCATTTGTTATAGTAGACTTGAGAAACATGCATTCAATCAAAATAGATGTTCATAGCCAAACTACATGGGTT
GAAGCCGGAGCTACCCTTGGAGAAGTTTATGATTGGGTTAATGAGAAAAATGAGAATCTTAGTTTGGGTGCTG
GGTATTGCCCTACTGTTTGCGCAGCTGGACACTTTGGTGGAGGAGGCTATGGACCATTGATGTGAAATTATGG
CCTTGCGGCTGATAATATCGTTGATGCACACTTAGTCAACATTGATGGAAAAGTGCTAGATCGAAAATATATG
GGAGAAGATCTCTTTTGGGCTATACGTTGTGGTGGAGGAGAAAGCTTCAGAATCATTGTAGCATGGAAAATTA

GATTGGTTGCTGTCCCAACAAAGTCTACTATGTTTAGTGTTAAAAAGATCAAGGAGATACATGAGCTTGTGAA
GTTAGTTAACAAGTGGCAAAACATTTCTTACAAGTATGACAAAGATTTATTACTTGTA
>B2_Purple_Kush__CM010792.2_58107644
ATGAAGTACTCAACATTCTCCTTTTGGTTTGTTTGCAAGATAATATTTTTCTTTCTCTCATTCAATATCCAAC
CTTCAATTGCTAATCCTCGAGAAAACTTCCTTAAATGCTTCTCGCAATATATTCCCACCAATGTAACAAATCT
AAAACTTACCCAAAACAACTTGTATATGCCTGTCCAAAATTCGACAATACACAATCTTAGATTCACCTCTAAC
ACAACCCCAAAACTACTTGTTATCGTCACTTCACATGTCTCTCATATCCAAGGCACTATTCTATGTAAGAAAA
TTGGTTTGCAAATTCAAACTCGAAACGGTGGTCATGATTCTGAAGGCATGTCCCACATATCTCAAGTCCCATT
TGTTATAGTAGACTTGAGAAACATGCATTCAATCAAAGATGTTCATAGCCAAATCGCAAGGGTTGAAGCCGGA
GCTACCCTTGGAGAAGTTTATTATTGGGTTAATGAGAAAAATGAGCTTAGTTTGGCTGCTTATTGCCCTACTG
TTAGCGCAGCTGGACACTTTGGTGGAGGAGGCTATGGACCATTGATGTGAAATTATGGCCTCGCGGATGATAA
TATCGTTGATGCACACTTAGTCAACGTTGATGGAAAAGTACTAGATCGAAAATCTATGGGACAAGATCTCTTT
TGGGCTATACGTGGTGGTGGAAGAGAAAGCTTCAGAATCATTGTAGCATGGAAAATTAGACTGGTTGCTGTCC
CAACAAAGTCTACTATGTTTAGTGTTAAAAAGATCAAGGAGATACATGAGCTTGTGAAGTTAGTTAACAAGTG
GCAAAATATTTCTTACAAGTATGACATAGATTTATTACTCATGACTCACTTCATAACTAGGAATATTACAGAT
AATCAAGGGAAGAATAAGACAACAATACACACTTACTTCTCTTTAGTTTTCCTTGGTGGAGTGGATAGTCTAG
TCGACTTAATGAACAAGAGTTTTCCTGAGTTTGGTATTAAAAAAATAGATTGCAAACAATTGAGCTGGATTGA
TACTATCATCTTCTATAGCGGTGTTGTAAATTACGGCACTGATAATTTTAATAACCAAATTTCGCTTGTTAGA
TCAGCTGGGCAGAACGGTGCTTTCAAGATTAAGTTAGACTATGTTAAGAAACCAATTCCAGAATCTGCATTTG
TCAAAATTTTGGAAAAATTATATGAAGAAGATAAAGGAGTTGGGATGTATGCGTTGTACCCTTACGGTTGTCT
AATGGATGAGATTTCAGAATCAGCAATTCCATTCCCTCATCGAGTTGGAATCATGTATGAATTATGGTACATA
TGTAGCTGGGAGAAGCACGAAGATAAAGAAAAGTATCTAAACTGGATTCGAAATGTTGATAACTTCATGACTC
CTTATGTGTCCCAAAATCCAAGATTGACATATCTCAATTATAGACATCTTGATATAGGAATAAATGATCCCAA
GAGTCAAAATAATTACACAGAAGCATGTATTTGGGGTGAGAAATTTGGTAAAAATTTTGACAGGCTAGTAAAA
GTGAAAACCCTGGTTGATTTCAATAATCTTTTTAGAAATGAACAAAGCATCCCACCTCTTCCACCGCATCGTC
ATTAA
>B2_Jamaican_Lion_JAATIP010000026.1_F8388_024903
ATGAAGTACTCAACATTCTCCTTTTGGTTTGTTTGCAAGATAATATTTTTCTTTCTCTCATTCAATATCCAAC
CTTCAATTGCTAATCCTCGAGAAAACTTCCTTAAATGCTTCTCGCAATATATTCCCACCAATGTAACAAATCT
AAAACTTACTCAAAACAACCAATTGTATATGCCTGTCCAAAATTCGACAATACACAATCTTAGATTCACCTCT
AACACAACCCAAAAACCACTTGTTATCGTCACTCCTTCACATGTCTCCCATATCCAAGGCACTATTCTATGTA
AGAAAATTGGTTTGCAAATTCAAACTCGAAACGGTGGTCATGATTCTGAAGGCATGTCCCACATATCTCAAGT
CCCATTTGTTATAGTAGACTTGAGAAACATGCATTCAATCAAAATAGATGTTCATAGCCAAACTACATGGGTT
GAAGCCGGAGCTACCATTGGAGAAGTTTATTATTGGGTTAATGAGAAAAATGAGAATCTTAGTTTGGGTGCTG
GGTATTGCCCTACTATTTGCGCAGCTGGACACTTTGGTGGAGGAGGCTATGGACCATTGATGTGAAATTATGG
CCTCGCGGATGATAATATCGTTGATGCACACTTAGTCAACGTTGATGGAAAAGTACTAGATCGAAAATCTATG
GGACAAGATCTCTTTTGGGCTATACGTGGTGGTGGAAGAGAAAGCTTCAGAATCATTGTAGCATGGAAAATTA
GACTGGTTGCTGTCCCAACAAAGTCTACTATGTTTAGTGTTAAAAAGATCAAGGAGATACATGAGCTTGTGAA
GTTAGTTAACAAGTGGCAAAATATTTCTTACAAGTATGACATAGATTTATTACTCATGACTCACTTCATAACT
AGGAATATTACAGATAATCAAGGGAAGAATAAGACAACAATACACACTTACTTCTCTTTAGTTTTCCTTGGTG
GAGTCGATAGTCTAGTCGACTTAATGAACAAGAGTTTTCCTGAGTTTGGTATTAAAAAAATAGATTGCAAACA
ATTGAGCTGGATTGATACTATCATCTTCTATAGCGGTGTTGTAAATTACGGCACTGATAATTTTAATAACCAA
ATTTCGCTTGTTAGATCAGCTGGGCAGAACGGTGCTTTCAAGATTAAGTTAGACTATGTTAAGAAACCAATTC
CAGAATCTGCATTTGTCAAAATTTTGGAAAAATTATATGAAGAAGATAAAGGAGTTGGGATGTATGCGTTGTA
CCCTTACGGTTGTCTAATGGATGAGATTTCAGAATCAGCAATTCCATTCCCTCATCGAGTTGGAATCATGTAT
GAATTATGGTACATATGTAGCTGGGAGAAGCACGAAGATAAAGAAAAGTATCTAAACTGGATTCGAAATGTTG
ATAACTTCATGACTCCTTATGTGTCCCAAAATCCAAGATTGACATATCTCAATTATAGACATCTTGATATAGG
AATAAATGATCCCAAGAGTCAAAATAATTACACAGAAGCATGTATTTTGGGTGAGAAATTTGGTAAAAATTTT
GACAGGCTAGTAAAAGTGAAAACCCTGGTTGATTTCAATAATCTTTTTAGAAATGAACAAAGCATCCCACCTC
TTCCACCGCATCGTCATTAA
>B2_Jilong_CM022965.1_91789995
ATGAAGTACTCAACATTCTCCTTTTGGTTTGTTTGCAAGATAATATTTTTCTTTCTCTCATTCAATATCCAAC
CTTCAATTGCTAATCCTCGAGAAAACTTCCTTAAATGCTTCTCGCAATATATTCCCACCAATGTAACAAATCT
AAAATACACTCAAAACAACCAATTGTATATGCCTGTCCAAAATTCGACAATACACAATCTTAGATTCACCTCT
AACACAACCCCAAAACCACTTGTTATCGTCACTCCTTCACATGTCTCCCATATCCAAGGCACTATTCTATGTA
AGAAAATTGGTTTGCAAATTCAAACTCGAAACGGTGGTCATGATTCTGAAGGCATGTCCCACATATCTCAAGT
CCCATTTGTTATAGTAGACTTGAGAAACATGCATTCAATCAAAATAGATGTTCATAGCCAAACTACATGGGTT
GAAGCCGGAGCTACCATTGGAGAAGTTTATTATTGGGTTAATGAGAAAAATGAGAATCTTAGTTTGGGTGCTG

GGTATTGCCCTACTATTTGCGCAGCTGGACACTTTGGTGGAGGAGGCTATGGACCATTGATGTGAAATTATGG
CCTCGCGGATGATAATATCGTTGATGCACACTTAGTCAACGTTGATGGAAAAGTACTAGATCGAAAATCTATG
GGACAAGATCTCTTTTGGGCTATACGTGGTGGTGGAAGAGAAAGCTTCAGAATCATTGTAGCATGGAAAATTA
GACTGGTTGCTGTCCCAACAAAGTCTACTATGTTTAGTGTTAAAAAGATCAAGGAGATACATGAGCTTGTGAA
GTTAGTTAACAAGTGGCAAAATATTTCTTACAAGTATGACATAGATTTATTACTCATGACTCACTTCATAACT
AGGAATATTACAGATAATCAAGGGAAGAATAAGACAACAATACACACTTACTTCTCTTTAGTTTTCCTTGGTG
GAGTGGATAGTCTAGTCGACTTAATGAACAAGAGTTTTCCTGAGTTTGGTATTAAAAAAATAGATTGCAAACA
ATTGAGCTGGATTGATACTATCATCTTCTATAGCGGTGTTGTAAATTACGGCACTGATAATTTTAATAACCAA
ATTTCGCTTGTTAGATCAGCTGGGCAGAACGGTGCTTTCAAGATTAAGTTAGACTATGTTAAGAAACCAATTC
CAGAATCTGCATTTGTCAAAATTTTGGAAAAATTATATGAAGAAGATAAAGGAGTTGGGATGTATGCGTTGTA
CCCTTACGGTTGTCTAATGGATGAGATTTCAGAATCAGCAATTCCATTCCCTCATCGAGTTGGAATCATGTAT
GAATTATGGTACATATGTAGCTGGGAGAAGCACGAAGATAAAGAAAAGTATCTAAACTGGATTCGAAATGTTG
ATAACTTCATGACTCCTTATGTGTCCCAAAATCCAAGATTGACATATCTCAATTATAGACATCTTGATATAGG
AATAAATGATCCCAAGAGTCAAAATAATTACACAGAAGCATGTATTTGGGGTGAGAAATTTGGTAAAAATTTT
GACAGGCTAGTAAAAGTGAAAACCCTGGTTGATTTCAATAATCTTTTTAGAAATGAACAAAGCATCCCACCTC
TTCCACCGCATCGTCATTAA
>B2_Jilong_CM022965.1_89962317
ATGAAGTACTCAACATTCTCCTTTTGGTTTGTTTGCAAGATATTTTTCTTTCTCTCAATTAATCAAACTTCAA
TTGCTAATCCTCGAGAAAACTTCCTTAAATGCTTCTCGCAATATATTCCCACCAATGTAACAAATCTAAAATA
CACCCAAAACAACCAATATATGCCTGTCCAAAATTCGACAATACACAATCTTAGATTCACCTCTAACACAACC
CCAAAACTACTTGTTATCGTCACTCCTTCACATGTCTCTCATATCCAAGGCACTATTCTATGTAAGAAAATTG
GTTTGCAAATTCGAACTCGAAGCGGTGGTCATGATTCTGAAGACATGTCCTACATATCTCAAGTCCCATTTGT
TATAGTAGACTTGAGAAACATGCATTCAATCAACATAGATGTTCATAGCCAAATCGCAAGGGTTGAAGCCGGA
GCTACCCTTGGAGAATATTATTGGGTTAATGAGAAAAATGAGAACTTAGTTTGGGCTGCTGGGTATTGCCCTA
CTGTTAGCGCTGGACACTTTGGTGGAGGAGGATATGGACCATTGATGCAAAATTATGGCCTCGCGGCTGATAA
TATCGTTGATGCACACTTAGTCAACGTTGATGCAAAAGTGCTAGATCGAAAATCTATGGGGGAAGATCTCTTT
TGGGCTATACGTGGTGGTGGAGGAGAAAGCTTCGGAATCATTGTAGCATGGAAAATTAGACTGGTTGCTGTCC
CAACAAAGTCTACTATGTTTAGTGTTAAAAAGATCATGGAGATACATGAGCTTGTCAAGTGAGTTAACAAATG
GCAAAATATTGCTTACAAGTATGACAAAGATTTATTACTCATGACTCACTTCATAACTAGGAATATTACAAAT
AATCATGGGAAGACAACAATACACACTTACTTCTCTTCAGTTTTCCTTGGAGTGGATAGTCTAGTCGACTTGA
TGAATAAGAGTTTTCCTGAGTTGGGTATTAAAAAAGATTGCAAACAATTGAGCTAGATTGATATTATCATCTT
TTATAGCGGTGTTGTAAATTACGGCACTGATAATTTTAATAAGGAAATTTTGCTTGATAGATCAGCTGGGCAG
AACGGTTCTTTAAAGATTAAGTTAGACTACGTTAAGAAACCAATTCCATCTGCGTTTGTCAAAATTTTGGAAA
AATATGAAGAAGATGAAGGAGCTGGGATGTTGTACCCTTACGGTGGTATAATGGATGAGATTTCAGAATCAGC
AATTCCATTCCCTCATTGAGCTGGAATCATGTATGAATTATGGTACATATGTAGCTGGGAGAAGCACGAAGAT
AACGAAAAGCATCTAAACTGGATTCGAAATGTTTATAGCTTCACTCCTTATGTGTCCTAAAATCCAAGATTGG
CATATCTCAATTATAGAGACCTTGATACTGGAATAAATGATCCCAAGAGTCCAAATAATTACACACAAGAAAG
TATTTGGGGTGAGAAGTATTTTGGTAAAAATTTTGACAGGGTAGTAAAAGTGAAAACCCTGGTTGATCCCAAT
AATTTTTTTAGAAATGAACAAAGCATCCCACCTCTTCCACCGCATCGTCATTAA
>B2_Jamaican_Lion_JAATIP010000026.1_F8388_024904
ATGAAGTACTCAACATTCTCCTTTTGGTTTGTTTGCAAGATAATATTTTTCTTTCTCTCATTCAATATCCAAA
CTTCAATTGCTAATCCTCGAGAAAACTTCCTTAAATGCTTCTCGCAATATATTCCCACCAATGTAACAAATCT
AAAACTTACCCAAAACAACCAATTGTATATGCCTGTCCAAAATTCGACAATACACAATCTTAGATTCACTCCT
TCACATGTCTCTCATATCCAAGGCACTATTCTATCCAAGAAAATTGGTTTGCAAATTCGAACTCGAAGCGGTG
GTCATGATTCTGAAGACATGTCCTACATATCTCAAGTCCCATTTGTTATAGTAGACTTGAGAAACATGCATTC
AATCAACATAGATGTTCATAGCCAAATCGCAAGGGTTGAAGCCGGAGCTACCCTTGGAGAAGTTTATTATTGG
GTTAATGAGAAAAATGAGAATCTTAGTTTGGCTGCTGGGTATTGCCCTACTGTTAGCGCAGCTGGACACTTTG
GTGGAGGAGGATATGGACCATTGATGCAAAATTATGGCCTCGCGGCTGATAATATCGTTGATGCACACTTAGT
CAACGTTGATGCAAAAGTGCTAGATCGAAAATCTATGGGGGAAGATCTCTTTTGGGCTATACGTGGTGGTGGA
GGAGAAAGCTTCGGAATCATTGTAGCATGGAAAATTAGACTGGTTGCTGTCCCAACAAAGTCTACTATGTTTA
GTGTTAAAAAGATCATGGAGATACATGAGCTTGTCAAGTGAGTTAACAAATGGCAAAATATTGCTTACAAGTA
TGACAAAGATTTATTACTCATGACTCACTTCATAACTAGGAATATTACAAATAATCATGGGAAGAATAAGACA
ACAATACACACTTACTTCTCTTCAGTTTTCCTTGGTGGAGTGGATAGTCTAGTCGACTTGATGAATAAGAGTT
TTCCTGAGTTGGGTATTAAAAAAACAGATTGCAAACAATTGAGCTAGATTGATATTATCATCTTTTATAGCGG
TGTTGTAAATTACGGCACTGATAATTTTAATAAGGAAATTTTGCTTGATAGATCAGCTGGGCAGAACGGTTCT
TTAAAGATTAAGTTAGACTACGTTAAGAAACCAATTCCAGAATCTGCGTTTGTCAAAATTTTGGAAAAATTAT
ATGAAGAAGATGAAGGAGCTGGGATGTATGCGTTGTACCCTTACGGTGGTATAATGGATGAGATTTCAGAATC
AGCAATTCCATTACCTCATTGAGCTGGAATCATGTATGAATTATGGTACATATGTAGCTGGGAGAAGCACGAA

GATAAGAAAAAGCATCTAAACTGGATTCGAAATGTTTATAGCTTCATTACTCCTTATGTGTCCTAAAATCCAA
GATTGGCATATCTCAATTATAGAGACCTTGATACTGGAATAAATGATCCCAAGAGTCCAAATAATTACACACA
AGCAAGTATTTGGGGTGAGAAGTATTTTGGTAAAAATTTTGACAGGGTAGTAAAAGTGAAAACCCTGGTTGAT
CCCAATAATTTTTTTAGAAATGAACAAAGCATCCCACCTCTTCCACCGCATCGTCATTAA
>B2_Jilong_CM022965.1_91891053
ATGAAGTACTCAACATTCTCCTTTTGGTTTGTTTGCAAGATAATATTTTTCTTTCTCTCATTCAATATCCAAA
CTTCAATTGCTAATCCTCGAGAAAACTTCCTTAAATGCTTCTCGCAATATATTCCCACCAATGTAACAAATCT
AAAATACACCCAAAACAACCAATTGTATATGCCTGTCCAAAATTCGACAATACACAATCTTAGATTCACCTCT
AACACAACCCCAAAACTACTTGTTATCGTCACTCCTTCACATGTCTCTCATATCCAAGGCACTATTCTATGTA
AGAAAATTGGTTTGCAAATTCGAACTCGAAGCGGTGGTCATGATTCTGAAGACATGTCCTACATATCTCAAGT
CCCATTTGTTATAGTAGACTTGAGAAACATGCATTCAATCAACATAGATGTTCATAGCCAAATCGCAAGGGTT
GAAGCCGGAGCTACCCTTGGAGAAGTTTATTATTGGGTTAATGAGAAAAATGAGAATCTTAGTTTGGCTGCTG
GGTATTGCCCTACTGTTAGCGCAGCTGGACACTTTGGTGGAGGAGGATATGGACCATTGATGCAAAATTATGG
CCTCGCGGCTGATAATATCGTTGATGCACACTTAGTCAACGTTGATGCAAAAGTGCTAGATCGAAAATCTATG
GGGGAAGATCTCTTTTGGGCTATACGTGGTGGTGGAGGAGAAAGCTTCGGAATCATTGTAGCATGGAAAATTA
GACTGGTTGCTGTCCCAACAAAGTCTACTATGTTTAGTGTTAAAAAGATCATGGAGATACATGAGCTTGTCAA
GTGAGTTAACAAATGGCAAAATATTGCTTACAAGTATGACAAAGATTTATTACTCATGACTCACTTCATAACT
AGGAATATTACAAATAATCATGGGAAGAATAAGACAACAATACACACTTACTTCTCTTCAGTTTTCCTTGGTG
GAGTGGATAGTCTAGTCGACTTGATGAATAAGAGTTTTCCTGAGTTGGGTATTAAAAAAACAGATTGCAAACA
ATTGAGCTAGATTGATATTATCATCTTTTATAGCGGTGTTGTAAATTACGGCACTGATAATTTTAATAAGGAA
ATTTTGCTTGATAGATCAGCTGGGCAGAACGGTTCTTTAAAGATTAAGTTAGACTACGTTAAGAAACCAATTC
CAGAATCTGCGTTTGTCAAAATTTTGGAAAAATTATATGAAGAAGATGAAGGAGCTGGGATGTATGCGTTGTA
CCCTTACGGTGGTATAATGGATGAGATTTCAGAATCAGCAATTCCATTCCCTCATTGAGCTGGAATCATGTAT
GAATTATGGTACATATGTAGCTGGGAGAAGCACGAAGATAACGAAAAGCATCTAAACTGGATTCGAAATGTTT
ATAGCTTCATTACTCCTTATGTGTCCTAAAATCCAAGATTGGCATATCTCAATTATAGAGACCTTGATACTGG
AATAAATGATCCCAAGAGTCCAAATAATTACACACAAGAAAGTATTTGGGGTGAGAAGTATTTTGGTAAAAAT
TTTGACAGGGTAGTAAAAGTGAAAACCCTGGTTGATCCCAATAATTTTTTTAGAAATGAACAAAGCATCCCAC
CTCTTCCACCGCATCGTCATTAA
>B2_Purple_Kush__CM010792.2_58200740
ATGAAGTACTCAACATTCTCCTTTTGGTTTGTTTGCAAGATAATATTTTTCTTTCTCTCATTCAATATCCAAA
CTTCAATTGCTAATCCTCGAGAAAACTTCCTTAAATGCTTCTCGCAATATATTCCCACCAATGTAACAAATCT
AAAACTTACCCAAAACAACCAATTGTATATGCCTGTCCAAAATTCGACAATACACAATCTTAGATTCACCTCT
AACACAACCCCAAAACTACTTGTTATCGTCACTCCTTCACATGTCTCTCATATCCAAGGCACTATTCTATCCA
AGAAAATTGGTTTGCAAATTCGAACTCGAAGCGGTGGTCATGATTCTGAAGACATGTCCTACATATCTCAAGT
CCCATTTGTTATAGTAGACTTGAGAAACATGCATTCAATCAACATAGATGTTCATAGCCAAATCGCAAGGGTT
GAAGCCGGAGCTACCCTTGGAGAAGTTTATTATTGGGTTAATGAGAAAAATGAGAATCTTAGTTTGGCTGCTG
GGTATTGCCCTACTGTTAGCGCAGCTGGACACTTTGGTGGAGGAGGATATGGACCATTGATGCAAAATTATGG
CCTCGCGGCTGATAATATCGTTGATGCACACTTAGTCAACGTTGATGCAAAAGTGCTAGATCGAAAATCTATG
GGGGAAGATCTCTTTTGGGCTATACGTGGTGGTGGAGGAGAAAGCTTCGGAATCATTGTAGCATGGAAAATTA
GACTGGTTGCTGTCCCAACAAAGTCTACTATGTTTAGTGTTAAAAAGATCATGGAGATACATGAGCTTGTCAA
GTGAGTTAACAAATGGCAAAATATTGCTTACAAGTATGACAAAGATTTATTACTCATGACTCACTTCATAACT
AGGAATATTACAAATAATCATGGGAAGAATAAGACAACAATACACACTTACTTCTCTTCAGTTTTCCTTGGTG
GAGTGGATAGTCTAGTCGACTTGATGAATAAGAGTTTTCCTGAGTTGGGTATTAAAAAAACAGATTGCAAACA
ATTGAGCTAGATTGATATTATCATCTTTTATAGCGGTGTTGTAAATTACGGCACTGATAATTTTAATAAGGAA
ATTTTGCTTGATAGATCAGCTGGGCAGAACGGTTCTTTAAAGATTAAGTTAGACTACGTTAAGAAACCAATTC
CAGAATCTGCGTTTGTCAAAATTTTGGAAAAATTATATGAAGAAGATGAAGGAGCTGGGATGTATGCGTTGTA
CCCTTACGGTGGTATAATGGATGAGATTTCAGAATCAGCAATTCCATTCCCTCATTGAGCTGGAATCATGTAT
GAATTATGGTACATATGTAGCTGGGAGAAGCACGAAGATAAGAAAAAGCATCTAAACTGGATTCGAAATGTTT
ATAGCTTCATTACTCCTTATGTGTCCTAAAATCCAAGATTGGCATATCTCAATTATAGAGACCTTGATACTGG
AATAAATGATCCCAAGAGTCCAAATAATTACACACAAGAAAGTATTTGGGGTGAGAAGTATTTTGGTAAAAAT
TTTGACAGGGTAGTAAAAGTGAAAACCCTGGTTGATCCCAATAATTTTTTTAGAAATGAACAAAGCATCCCAC
CTCTTCCACCGCATCGTCATTAA
>B2_AGQN01159678.1
ATGAAGTACTCAACATTCTCCTTTTGGTTTGTTTGCAAGATAATATTTTTCTTTCTCTCATTCAATATCCAAA
CTTCAATTGCTAATCCTCGAGAAAACTTCCTTAAATGCTTCTCGCAATATATTCCCACCAATGTAACAAATCT
AAAATACACCCAAAACAACCAATTGTATATGCCTGTCCAAAATTCGACAATACACAATCTTAGATTCACCTCT
AACACAACCCCAAAACTACTTGTTATCGTCACTCCTTCACATGTCTCTCATATCCAAGGCACTATTCTATGTA
AGAAAATTGGTTTGCAAATTCGAACTCGAAGCGGTGGTCATGATTCTGAAGACATGTCCTACATATCTCAAGT

CCCATTTGTTATAGTAGACTTGAGAAACATGCATTCAATCAACATAGATGTTCATAGCCAAATCGCAAGGGTT
GAAGCCGGAGCTACCCTTGGAGAAGTTTATTATTGGGTTAATGAGAAAAATGAGAATCTTAGTTTGGCTGCTG
GGTATTGCCCTACTGTTAGCGCAGCTGGACACTTTGGTGGAGGAGGATATGGACCATTGATGCAAAATTATGG
CCTCGCGGCTGATAATATCGTTGATGCACACTTAGTCAACGTTGATGCAAAAGTGCTAGATCGAAAATCTATG
GGGGAAGATCTCTTTTGGGCTATACGTGGTGGTGGAGGAGAAAGCTTCGGAATCATTGTAGCATGGAAAATTA
GACTGGTTGCTGTCCCAACAAAGTCTACTATGTTTAGTGTTAAAAAGATCATGGAGATACATGAGCTTGTCAA
GTGAGTTAACAAATGGCAAAATATTGCTTACAAGTATGACAAAGATTTATTACTCATGACTCACTTCATAACT
AGGAATATTACAAATAATCATGGGAAGAATAAGACAACAATACACACTTACTTCTCTTCAGTTTTCCTTGGTG
GAGTGGATAGTCTAGTCGACTTGATGAATAAGAGTTTTCCTGAGTTGGGTATTAAAAAAACAGATTGCAAACA
ATTGAGCTAGATTGATATTATCATCTTTTATAGCGGTGTTGTAAATTACGGCACTGATAATTTTAATAAGGAA
ATTTTGCTTGATAGATCAGCTGGGCAGAACGGTTCTTTAAAGATTAAGTTAGACTACGTTAAGAAACCAATTC
CAGAATCTGCGTTTGTCAAAATTTTGGAAAAATTATATGAAGAAGATGAAGGAGCTGGGATGTATGCGTTGTA
CCCTTACGGTGGTATAATGGATGAGATTTCAGAATCAGCAATTCCATTCCCTCATTGAGCTGGAATCATGTAT
GAATTATGGTACATATGTAGCTGGGAGAAGCACGAAGATAACAAGCATCTAAACTGGATTCGAAATGTTTATA
GCTTCATTACTCCTTATGTGTCCTAAAATCCAAGATTGGCATATCTCAATTATAGAGACCTTGATACTGGAAT
AAATGATCCCAAGAGTCCAAATAATTACACACAAGAAAGTATTTGGGGTGAGAAGTATTTTGGTAAAAATTTT
GACAGGGTAGTAAAAGTGAAAACCCTGGTTGATCCCAATAATTTTTTTAGAAATGAACAAAGCATCCCACCTC
TTCCACCGCATCGTCATTAA
>B2_KJ469376.1
ATGAAGTGCTCAACATTCCCCTTTTGGTTTGTTTGCAAGATAATATTTTTCTTTCTCTCATTCAATATCCAAA
CTTCAATTGCTAATCCTCGAGAAAACTTCCTTAAATGCTTCTCGCAATATATTCCCACCAATGTAACAAATCT
AAAACTTACACCCAAAACAACCAATTGTATATGCCTGTCCAAAATTCAACAATACACAATCTTAGATTCACCT
CTAACACAACCCCAAAACTACTTGTTATCGTCACTCCTTCATATGTCTCTCATATCCAAGGCACTATTCTATG
TCCAAGAAAATTGGTTTGCAAATTCGAACTCGAAGCGGTGGTCATGATTCTGAAGACATGTCCTACATATCTC
AAGTCCCATTTGTTATAGTAGACTTGAGAAACATGCATTCAATCAACATAGATGTTCATAGCCAAATCGCAAG
GGTTGAAGCCGGAGCTACCCTTGGAGAAGTTTATTATTGGGTTAATGAGAAAAATGAGAATCTTAGTTTGGCT
GCTGGGTATTGCCCTACTGTTAGCGCAGCTGGACACTTTGGTGGAGGAGGATATGGACCATTGATGCAAAATT
ATGGCCTCGCGGCTGATAATATCGTTGATGCACACTTAGTCAACGTTGATGCAAAAGTGCTAGATCGAAAATC
TATGGGGGAAGATCTCTTTTGGGCTATACGTGGTGGTGGAGGAGAAAGCTTCGGAATCATTGTAGCATGGAAA
ATTAGACTGGTTGCTGTCCCAACAAAGTCTACTATGTTTAGTGTTAAAAAGATCATGGAGATACATGAGCTTG
TCAAGTGAGTTAACAAATGGCAAAATATTGCTTACAAGTATGACAAAGATTTATTACTCATGACTCACTTCAT
AACTAGGAATATTACAAATAATCATGGGAAGAATAAGACAACAATACACACTTACTTCTCTTCAGTTTTCCTT
GGTGGAGTGGATAGTCTAGTCGACTTGATGAATAAGAGTTTTCCTGAGTTGGGTATTAAAAAAACAGATTGCA
AACAATTGAGCTAGATTGATATTATCATCTTTTATAGCGGTGTTGTAAATTACGGCACTGATAATTTTAATAA
GGAAATTTTGCTTGATAGATCAGCTGGGCAGAACGGTTCTTTAAAGATTAAGTTAGACTACGTTAAGAAACCA
ATTCCAGAATCTGCGTTTGTCAAAATTTTGGAAAAATTATATGAAGAAGATGAAGGAGTTGGGATGTATGCGT
TGTACCCTTACGGTGGTATAATGGATGAGATTTCAGAATCAGCAATTCCATTCCCTCATTGAGCTGGAATCAT
GTATGAATTATGGTACATATGTAGCTGGGAGAAGCACGAAGATAACGAAAAAGCATCTAAACTGGATTCGAAA
TGTTTATAGCTTCATTACTCCTTATGTGTCCTAAAATCCAAGATTGGCATATCTCAATTATAGAGACCTTGAT
ACTGGAATAAATGATCCCAAGAGTCCAAATAATTACACACAAGAAAGTATTTGGGGTGAGAAGTATTTTGGTA
AAAATTTTGACAGGGTAGTAAAAGTGAAAACCCTGGTTGATCCCAATAATTTTTTTAGAAATGAACAAAGCAT
CCCACCTCTTCCACGGCATCGTCATTAA
>B2_MG996422.1
TTCAATATCCAAACTTCAATTGCTAATCCTCGAGAAAACTTCCTTAAATGCTTCTCGCAATATATTCCCACCA
ATGTAACAAATCTAAAACTTACACCCAAAACAACCAATTGTATATGCCTGTCCAAAATTCGACAATACACAAT
CTTAGATTCACCTCTAACACAACCCCAAAACTACTTGTTATCGTCACTCCTTCACATGTCTCTCATATCCAAG
GCACTATTCTATGTCCAAGAAAATTGGTTTGCAAATTCGAACTCGAAGCGGTGGTCATGATTCTGAAGACATG
TCCTACATATCTCAAGTCCCATTTGTTATAGTAGACTTGAGAAACATGCATTCAATCAACATAGATGTTCATA
GCCAAATCGCAAGGGTTGAAGCCGGAGCTACCCTTGGAGAAGTTTATTATTGGGTTAATGAGAAAAATGAGAA
TCTTAGTTTGGCTGCTGGGTATTGCCCTACTGTTAGCGCAGCTGGACACTTTGGTGGAGGAGGATATGGACCA
TTGATGCAAAATTATGGCCTCGCGGCTGATAATATCGTTGATGCACACTTAGTCAACGTTGATGCAAAAGTGC
TAGATCGAAAATCTATGGGGGAAGATCTCTTTTGGGCTATACGTGGTGGTGGAGGAGAAAGCTTCGGAATCAT
TGTAGCATGGAAAATTAGACTGGTTGCTGTCCCAACAAAGTCTACTATGTTTAGTGTTAAAAAGATCATGGAG
ATACATGAGCTTGTCAAGTGAGTTAACAAATGGCAAAATATTGCTTACAAGTATGACAAAGATTTATTACTCA
TGACTCACTTCATAACTAGGAATATTACAAATAATCATGGGAAGAATAAGACAACAATACACACTTACTTCTC
TTCAGTTTTCCTTGGTGGAGTGGATAGTCTAGTCGACTTGATGAATAAGAGTTTTCCTGAGTTGGGTATTAAA
AAAACAGATTGCAAACAATTGAGCTAGATTGATATTATCATCTTTTATAGCGGTGTTGTAAATTACGGCACTG
ATAATTTTAATAAGGAAATTTTGCTTGATAGATCAGCTGGGCAGAACGGTTCTTTAAAGATTAAGTTAGACTA

CGTTAAGAAACCAATTCCAGAATCTGCGTTTGTCAAAATTTTGGAAAAATTATATGAAGAAGATGAAGGAGCT
GGGATGTATGCGTTGTACCCTTACGGTGGTATAATGGATGAGATTTCAGAATCAGCAATTCCATTCCCTCATT
GAGCTGGAATCATGTATGAATTATGGTACATATGTAGCTGGGAGAAGCACGAAGATAACGAAAAAGCATCTAA
ACTGGATTCGAAATGTTTATAGCTTCATTACTCCYTATGTGTCCTAAAATCCAAGATTGGCATATCTCAATTA
TAGAGACCTTGATACTGGAATAAATGATCCCAAGCGTCMAAATAATTACACMCAMGAAAGTATTTGGGGTGAG
AMGTATTTTCGTCAAAATTTTGACAGGGTAGTAAAAGTGAAAACCCTGGT
>B2_MG996420.1
TTCAATATCCAAACTTCAATTGCTAATCCTCGAGAAAACTTCCTTAAATGCTTCTCGCAATATATTCCCACCA
ATGTAACAAATCTAAAACTTACACCCAAAACAACCAATTGTATATGCCTGTCCAAAATTCGACAATACACAAT
CTTAGATTCACCTCTAACACAACCCCAAAACYACTTGTTATCGTCACTCCTTCACATGTCTCYCATATCCAAG
GCACTATTCTATGTCCAAGAAAATTGGTTTGCAAATTCRAACTCGAARCGGTGGTCATGATTCTGAAGRCATG
TCCYACATATCTCAAGTCCCATTTGTTATAGTAGACTTGAGAAACATGCATTCAATCAACATAGATGTTCATA
GCCAAAYYRCAWGGGTTGAAGCCGGAGCTACCCTTGGAGAAGTTTATTATTGGGTTAATGAGAAAAATGAGAA
TCTTAGTTTGGCTGCTGGGTATTGCCCTACTGTTAGCGCAGCTGGACACTTTGGTGGAGGAGGATATGGACCA
TTGATGYRAAATTATGGCCTCGCGGMTGATAATATCGTTGATGCACACTTAGTCAACGTTGATGCAAAAGTGC
TAGATCGAAAATCTATGGGGGAAGATCTCTTTTGGGCTATACGTGGTGGTGGAGGAGAAAGCTTCGGAATCAT
TGTAGCATGGAAAATTAGACTGGTTGCTGTCCCAACAAAGTCTACTATGTTTAGTGTTAAAAAGATCATGGAG
ATACATGAGCTTGTCAAGTGAGTTAACAAATGGCAAAATATTGCTTACAAGTATGACAAAGATTTATTACTCA
TGACTCACTTCATAACTAGGAATATTACAAATAATCATGGGAAGAATAAGACAACAATACACACTTACTTCTC
TTCAGTTTTCCTTGGTGGAGTGGATAGTCTAGTCGACTTGATGAATAAGAGTTTTCCTGAGTTGGGTATTAAA
AAAACAGATTGCAAACAATTGAGCTAGATTGATATTATCATCTTTTATAGCGGTGTTGTAAATTACGGCACTG
ATAATTTTAATAAGGAAATTTTGCTTGATAGATCAGCTGGGCAGAACGGTTCTTTAAAGATTAAGTTAGASTA
CGTTAAGAAACCAATTCCAGAATMTGCGTTTGTCAAAATTTTGGAAAAATTATATGAAGAAGATGAAGGAGMT
GGGATGTATGCGTTGTACCCYTACGGTGGTATAATGGATGAGATTTCAGAATCAGCAATTCCATTCCCTCATT
GAGCTGGAATCATGTATGAATTATGGTACATATGTAGCTGGGAGAAGCACGAAGATAACGAAAAAGCATCTAA
ACTGGATTCGAAATGTTTATAGCTTCATTACTCCTTATGTGTCCTAAAATCCAAGATTGGCATATCTCAATTA
TAGAGACCTTGATACTGGAATAAATGATCCCAAGAGTCCAAATAATTACACACAAGAAAGTATTTGGGGTGAG
AAGTATTTTGGTAAAAATTTTGACAGGGTAGTAAAAGTGAAAACCCTGGTTGAT
>B2_JH231038.1
ATGAAGTACTAAACATTCTCCTTTTGGTTTGTTTGCAAGATAATATTTTTTCTCTCATTCAATATCCAAACTT
CAATTACTAATCCTCGAGAAAACTTCCTTAAATGCTTCTCGCAATATATTCCCACCAATGTAACAAATCTAAA
ATACACTCAAAACAACCAATTGTATATGCCTGTCCAAAATTCGACAATACACAATCTTAGATTCACCTCTAAC
ACAACCCCAAAACCACTTGTTATCGTCACTCCTTCACATGTCTCCCATATCCAAGGCACTATTCTATGTAAGA
AAATTGGTTTGCAAATTCAAACTCGAAACGGTGGTCATGATTCTGAAGGCATGTCCCACATATCTCAAGTCCC
ATTTGTTATAGTAGACTTGAGAAACATGCATTCAATCAAAATAGATGTTCATAGCCAAACTACATGGGTTGAA
GCCGGAGCTACCCTTGGAGAAGTTTATGATTGGGTTAATGAGAAAAATGAGAATCTTAGTTTGGGTGCTGGGT
ATTGCCCTACTGTTTGCGCAGCTGGACACTTTGGTGGAGGAGGCTATGGACCATTGATGTGAAATTATGGCCT
TGCGGCTGATAATATCGTTGATGCACACTTAGTCAACATTGATGGAAAAGTGCTAGATCGAAAATATATGGGA
GAAGATCTCTTTTGGGCTATACGTTGTGGTGGAGGAGAAAGCTTCAGAATCATTGTAGCATGGAAAATTAGAT
TGGTTGCTGTCCCAACAAAGTCTACTATGTTTAGTGTTAAAAAGATCAAGGAGATACATGAGCTTGTGAAGTT
AGTTAACAAGTGGCAAAACATTTCTTACAAGTATGACAAAGATTTATTACTTGTA
>B2_AGQN01254730.1
TTACTCATGACTCACTTCATAACTAGGAATATTATAGATAATCAAGGGAAGAATAAGACAACAGTACACACTT
ACTTCTCTTTAGTTTTCCTTGGTGGAGTGGATAGTCTAGTCGACTTAATGAACAAGAGTTTTCCTGAGTTTGG
CATTGAAACAGATTGCAAACAATTGAGCTGGATTGATACTATCATCTTCTATAGCGGTGTTGTAAATTACGGC
ACTGATAATTTTAATAAGGAAATTTTGCTTGATAGATCAGCTGGGCAGAACGGTGCTTTCAAGATTAAGTTAG
ACTACGTTAAGAAACCAATTCCAGAATCTGCGTTTGTCAAAATTTTGGAAAAATTATATGAAGAAGATGAAGG
AGTTGGGATGTATGCGTTGTACACTTACGGTTGTATAATGGATGAGATTTCAGAATCAGCAATTCCATTCCCT
CATCGAGTTGGAATCATGTATGAATTATGGTACACATGTAGCTGGGAGAAGCACGAAGATAAAGAAAAGTATC
TAAACTGGATTCGAAATGTTGATAACTTCATGACTCCTTATGTGTCCCAAAATCCAAGATTGACATATCTCAA
TTATAGACACCTTGATATAGGAATAAATGATCCCAAGAGTCAAAATAATTACACACAAGCATGTATTTGGGGT
GAGAAGTATTTTGGTAAAAATTTTGACAGGCTAGTAAAAGTGAAAACCCTGGTTGATCTCGATAATTTTTTTA
GAAATGAACAAAGCATCCCACCTCTTCCACCGCATCGTCATTAA
>B2_KJ469375.1
CCAATGTAACAAATCTAAAACTTACACCCAAAACAACCAATTGTATATGCCTGTCCAAAATTCGACAATACAC
AATCTTAGATTCACCTCTAACACAACCCCAAAACCACTTGTTATCGTCACTCCTTCACATGTCTCCCATATCC
AAGGCACTATTCTATGTCCAAGAAAATTGGTTTGCAAATTCAAACTCGAAACGGTGGTCATGATTCTGAAGGC
ATGTCCCACATATCTCAAGTCCCATTTGTTATAGTAGACTTGAGAAACATGCATTCAATCAAAATAGATGTTC

ATAGCCAAACTACATGGGTTGAAGCCGGAGCTACCATTGGAGAAGTTTATTATTGGGTTAATGAGAAAAATGA
GAATCTTAGTTTGGGTGCTGGGTATTGCCCTACTATTTGCGCAGCTGGACACTTTGGTGGAGGAGGCTATGGA
CCATTGATGTGAAATTATGGCCTCGCGGATGATAATATCGTTGATGCACACTTAGTCAACGTTGATGGAAAAG
TACTAGATCGAAAATCTATGGGACAAGATCTCTTTTGGGCTATACGTGGTGGTGGAAGAGAAAGCTTCAGAAT
CATTGTAGCATGGAAAATTAGACTGGTTGCTGTCCCAACAAAGTCTACTATGTTTAGTGTTAAAAAGATCAAG
GAGATACATGAGCTTGTGAAGTTAGTTAACAAGTGGCAAAATATTTCTTACAAGTATGACATAGATTTATTAC
TCATGACTCACTTCATAACTAGGAATATTACAGATAATCAAGGGAAGAATAAGACAACAATACACACTTACTT
CTCTTTAGTTTTCCTTGGTGGAGTGGATAGTCTAGTCGACTTAATGAACAAGAGTTTTCCTGAGTTTGGTATT
AAAAAAATAGATTGCAAACAATTGAGCTGGATTGATACTATCATCTTCTATAGCGGTGTTGTAAATTACGGCA
CTGATAATTTTAATAACCAAATTTCGCTTGTTAGATCAGCTGGGCAGAACGGTGCTTTCAAGATTAAGTTAGA
CTATGTTAAGAAACCAATTCCAGAATCTGCATTTGTCAAAATTTTGGAAAAATTATATGAAGAAGATAAAGGA
GTTGGGATGTATGCGTTGTACCCTTACGGTTGTCTAATGGATGAGATTTCAGAATCAGCAATTCCATTCCCTC
ATCGAGTTGGAATCATGTATGAATTATGGTACATATGTAGCTGGGAGAAGCACGAAGATAAAGAAAAGTATCT
AAACTGGATTCGAAATGTTGATAACTTCATGACTCCTTATGTGTCCCAAAATCCAAGATTGACATATCTCAAT
TATAGACATCTTGATATAGGAATAAATGATCCCAAGAGTCAAAATAATTACACAGAAGCATGTATTTGGGGTG
AGAAATCTTTGGTAAAAATTTTGACAGGCTAGTAAAAGTGAAAACCCTGGTTGATTTCAATAATCTTTTTAGA
AATGAACAAAGCATCCCACCTCTTCCACGGCATCGTCATTAA
>B2_Cannatonic_MNPR01005992_13240
ATGAAGTACTCAACATTCTCCTTTTGGTTTGTTTGCAAGATAATATTTTTCTTTCTCTCATTCAATATCCAAA
CTTCAATTGCTAATCCTCGAGAAAACTTCCTTAAATGCTTCTCGCAATATATTCCCACCAATGTAACAAATCT
AAAATACACCCAAAACAACCAATTGTATATGCCTGTCCAAAATTCGACAATACACAATCTTAGATTCACCTCT
AACACAACCCCAAAACTACTTGTTATCGTCACTCCTTCACATGTCTCTCATATCCAAGGCACTATTCTATGTA
AGAAAATTGGTTTGCAAATTCGAACTCGAAGCGGTGGTCATGATTCTGAAGACATGTCCTACATATCTCAAGT
CCCATTTGTTATAGTAGACTTGAGAAACATGCATTCAATCAACATAGATGTTCATAGCCAAATCGCAAGGGTT
GAAGCCGGAGCTACCCTTGGAGAAGTTTATTATTGGGTTAATGAGAAAAATGAGAATCTTAGTTTGGCTGCTG
GGTATTGCCCTACTGTTAGCGCAGCTGGACACTTTGGTGGAGGAGGATATGGACCATTGATGCAAAATTATGG
CCTCGCGGCTGATAATATCGTTGATGCACACTTAGTCAACGTTGATGCAAAAGTGCTAGATCGAAAATCTATG
GGGGAAGATCTCTTTTGGGCTATACGTGGTGGTGGAGGAGAAAGCTTCGGAATCATTGTAGCATGGAAAATTA
GACTGGTTGCTGTCCCAACAAAGTCTACTATGTTTAGTGTTAAAAAGATCATGGAGATACATGAGCTTGTCAA
GTGAGTTAACAAATGGCAAAATATTGCTTACAAGTATGACAAAGATTTATTACTCATGACTCACTTCATAACT
AGGAATATTACAAATAATCATGGGAAGAATAAGACAACAATACACACTTACTTCTCTTCAGTTTTCCTTGGTG
GAGTGGATAGTCTAGTCGACTTGATGAATAAGAGTTTTCCTGAGTTGGGTATTAAAAAAACAGATTGCAAACA
ATTGAGCTAGATTGATATTATCATCTTTTATAGCGGTGTTGTAAATTACGGCACTGATAATTTTAATAAGGAA
ATTTTGCTTGATAGATCAGCTGGGCAGAACGGTTCTTTAAAGATTAAGTTAGACTACGTTAAGAAACCAATTC
CAGAATCTGCGTTTGTCAAAATTTTGGAAAAATTATATGAAGAAGATGAAGGAGCTGGGATGTATGCGTTGTA
CCCTTACGGTGGTATAATGGATGAGATTTCAGAATCAGCAATTCCATTCCCTCATTGAGCTGGAATCATGTAT
GAATTATGGTACATATGTAGCTGGGAGAAGCACGAAGATAACAAAAAGCATCTAAACTGGATTCGAAATGTTT
ATAGCTTCATTACTCCTTATGTGTCCTAAAATCCAAGATTGGCATATCTCAATTATAGAGACCTTGATACTGG
AATAAATGATCCCAAGAGTCCAAATAATTACACACAAGAAAGTATTTGGGGTGAGAAGTATTTTGGTAAAAAT
TTTGACAGGGTAGTAAAAGTGAAAACCCTGGTTGATCCCAATAATTTTTTTAGAAATGAACAAAGCATCCCAC
CTCTTCCACCGCATCGTCATTAA
>B2_LAconfidential_LKUA01020408.1_3817
ATGAAGTACTCAACATTCTCCTTTTGGTTTGTTTGCAAGATAATATTTTTCTTTCTCTCATTCAATATCCAAA
CTTCAATTGCTAATCCTCGAGAAAACTTCCTTAAATGCTTCTCGCAATATATTCCCACCAATGTAACAAATCT
AAAATACACCCAAAACAACCAATTGTATATGCCTGTCCAAAATTCGACAATACACAATCTTAGATTCACCTCT
AACACAACCCCAAAACTACTTGTTATCGTCACTCCTTCACATGTCTCTCATATCCAAGGCACTATTCTATGTA
AGAAAATTGGTTTGCAAATTCGAACTCGAAGCGGTGGTCATGATTCTGAAGACATGTCCTACATATCTCAAGT
CCCATTTGTTATAGTAGACTTGAGAAACATGCATTCAATCAACATAGATGTTCATAGCCAAATCGCAAGGGTT
GAAGCCGGAGCTACCCTTGGAGAAGTTTATTATTGGGTTAATGAGAAAAATGAGAATCTTAGTTTGGCTGCTG
GGTATTGCCCTACTGTTAGCGCAGCTGGACACTTTGGTGGAGGAGGATATGGACCATTGATGCAAAATTATGG
CCTCGCGGCTGATAATATCGTTGATGCACACTTAGTCAACGTTGATGCAAAAGTGCTAGATCGAAAATCTATG
GGGGAAGATCTCTTTTGGGCTATACGTGGTGGTGGAGGAGAAAGCTTCGGAATCATTGTAGCATGGAAAATTA
GACTGGTTGCTGTCCCAACAAAGTCTACTATGTTTAGTGTTAAAAAGATCATGGAGATACATGAGCTTGTCAA
GTGAGTTAACAAATGGCAAAATATTGCTTACAAGTATGACAAAGATTTATTACTCATGACTCACTTCATAACT
AGGAATATTACAAATAATCATGGGAAGAATAAGACAACAATACACACTTACTTCTCTTCAGTTTTCCTTGGTG
GAGTGGATAGTCTAGTCGACTTGATGAATAAGAGTTTTCCTGAGTTGGGTATTAAAAAAACAGATTGCAAACA
ATTGAGCTAGATTGATATTATCATCTTTTATAGCGGTGTTGTAAATTACGGCACTGATAATTTTAATAAGGAA
ATTTTGCTTGATAGATCAGCTGGGCAGAACGGTTCTTTAAAGATTAAGTTAGACTACGTTAAGAAACCAATTC

CAGAATCTGCGTTTGTCAAAATTTTGGAAAAATTATATGAAGAAGATGAAGGAGCTGGGATGTATGCGTTGTA
CCCTTACGGTGGTATAATGGATGAGATTTCAGAATCAGCAATTCCATTCCCTCATTGAGCTGGAATCATGTAT
GAATTATGGTACATATGTAGCTGGGAGAAGCACGAAGATAACAAAAAGCATCTAAACTGGATTCGAAATGTTT
ATAGCTTCATTACTCCTTATGTGTCCTAAAATCCAAGATTGGCATATCTCAATTATAGAGACCTTGATACTGG
AATAAATGATCCCAAGAGTCCAAATAATTACACACAAGAAAGTATTTGGGGTGAGAAGTATTTTGGTAAAAAT
TTTGACAGGGTAGTAAAAGTGAAAACCCTGGTTGATCCCAATAATTTTTTTAGAAATGAACAAAGCATCCCAC
CTCTTCCACCGCATCGTCATTAA
>B2_Chemdog91_LKUB01066924.1_1
ATTCGAACTCGAAGCGGTGGTCATGATTCTGAAGACATGTCCTACATATCTCAAGTCCCATTTGTTATAGTAG
ACTTGAGAAACATGCATTCAATCAACATAGATGTTCATAGCCAAATCGCAAGGGTTGAAGCCGGAGCTACCCT
TGGAGAAGTTTATTATTGGGTTAATGAGAAAAATGAGAATCTTAGTTTGGCTGCTGGGTATTGCCCTACTGTT
AGCGCAGCTGGACACTTTGGTGGAGGAGGATATGGACCATTGATGCAAAATTATGGCCTCGCGGCTGATAATA
TCGTTGATGCACACTTAGTCAACGTTGATGCAAAAGTGCTAGATCGAAAATCTATGGGGGAAGATCTCTTTTG
GGCTATACGTGGTGGTGGAGGAGAAAGCTTCGGAATCATTGTAGCATGGAAAATTAGACTGGTTGCTGTCCCA
ACAAAGTCTACTATGTTTAGTGTTAAAAAGATCATGGAGATACATGAGCTTGTCAAGTTAGTTAACAAATGGC
AAAATATTGCTTACAAGTATGACAAAGATTTATTACTCATGACTCACTTCATAACTAGGAATATTACAAATAA
TCATGGGAAGAATAAGACAACAATACACACTTACTTCTCTTCAGTTTTCCTTGGTGGAGTGGATAGTCTAGTC
GACTTGATGAATAAGAGTTTTCCTGAGTTGGGTATTAAAAAAACAGATTGCAAACAATTGAGCTAGATTGATA
TTATCATCTTTTATAGCGGTGTTGTAAATTACGGCACTGATAATTTTAATAAGGAAATTTTGCTTGATAGATC
AGCTGGGCAGAACGGTTCTTTAAAGATTAAGTTAGACTACGTTAAGAAACCAATTCCAGAATCTGCGTTTGTC
AAAATTTTGGAAAAATTATATGAAGAAGATGAAGGAGTTGGGATGTATGCGTTGT
>B2_PBBK_MXBD01000395.1_37139
GTTTGCAAGATAATATTTTTCTTTCTCTCATTCAATATCCAAACTTCAATTGCTAATCCTCGAGAAAACTTCC
TTAAATGCTTCTCGCAATATATTCCCACCAATGTAACAAATCTAAAATACACTCAAAACAACCAATTGTATAT
GCCTGTCCAAAATTCGACAATACACAATCTTAGATTCACCTCTAACACAACCCCAAAACCACTTGTTATCGTC
ACTCCTTCACATGTCTCCCATATCCAAGGCACTATTCTATGTAAGAAAATTGGTTTGCAAATTCAAACTCGAA
GCGGTGGTCATGATTCTGAAGGCATGTCCTACATATCTCAAGTCCCATTTGTTATAGTAGACTTGAGAAACAT
GCATTCAATCAAAATAGATGTTCATAGCCAAATCAGGGTTGAAGCCGGAGCTACCCTTGGAGAAGTTTATTAT
TGGGTTAATGAGAAAAATGAGAATCTTAGTTTGGCTGCTGGGTATTGCCCTACTGTTGCAGCTGGACACTTTG
GTGGAGGAGGATATGGACCATTGATGAATTATGGCCTCGCGGCTGATAATATCGTTGATGCACACTTAGTCAA
CGTTGATGCAAAAGTGCTAGATCGAAAATCTATGGGGGAAGATCTCTTTTGGGCTATACGTGGTGGTGGAGGA
GAAAGCTTCGGAATCATTGTAGCATGGAAAATTAGACTGGTTGCTGTCCCAACAAAGTCTACTATGTTTAGTG
TTAAAAAGATCATGGAGATACATGAGCTTGTCAAGTGAGTTAACAAATGGCAAAATATTTACAAGTATGACGA
TTTATTACTCATGACTCACTTCATAACTAGGAATATTACAAATAATCATGGGAAGAATAAGACAACAATACAC
ACTTACTTCTCTTCAGTTTTCCTTGGTGGAGTGGATAGTCTAGTCGACTTAATGAACAAGAGTTTTCCTGAGT
TGGGTATTAAAAAAACAGATTGCAAACAATTGAGCTAGATTGATACTATCATCTATAGCGGTGTTGTAAATTA
CGGCACTGATAATTTTAATGAAATTTTGCTTGATAGATCAGCTGGGCAGAACGGTGCTTTAAAGATTAAGTTA
GACTACGTTAAGAAACCAATTCCAGAATCTGCGTTTGTCAAAATTTTGGAAAAATTATATGAAGAAGATGAAG
GAGCTGGGATGTATGCGTTGTACCCTTACGGTATAATGGATGAGATTTCAGAATCAGCAATTCCATTCCCTCA
TTGAGGAATCATGTATGAATTATGGTACATATGTAGCTGGGAGAAGCACGAAGATAAGAAAGATCTAAACTGG
ATTCGAAATGTTTATAACTTCATTACTCCTTATGTGTCCTAAAATCCAAGATTGGCATATCTCAATTATAGAG
ACCTTGATGGAATAAATGATCCCAAGAGTCCAAATAATTACACACAAGCAAGTATTTGGGGTGAGAAGTATTT
TGGTAAAAATTTTGACAGGGTAGTAAAAGTGAAAACCCTGGTTGATTCCAATAATTTTTTTAGAAATGAACAA
AGCATCCCACCTCTTCCACCGCATCGTCATTAA
>B2_LAconfidential_LKUA01173866.1_3
AAGACAACAGTACACACTTACTTCTCTTTAGTTTTCCTTGGTGGAGTGGATAGTCTAGTCGACTTAATGAACA
AGAGTTTTCCTGAGTTTGGCATTGAAACAGATTGCAAACAATTGAGCTGGATTGATACTATCATCTTCTATAG
CGGTGTTGTAAATTACGGCACTGATAATTTTAATAAGGAAATTTTGCTTGATAGATCAGCTGGGCAGAACGGT
GCTTTCAAGATTAAGTTAGACTACGTTAAGAAACCAATTCCAGAATCTGCGTTTGTCAAAATTTTGGAAAAAT
TATATGAAGAAGATGAAGGAGTTGGGATGTATGCGTTGTACACTTACGGTTGTATAATGGATGAGATTTCAGA
ATCAGCAATTCCATTCCCTCATCGAGTTGGAATCATGTATGAATTATGGTACACATGTAGCTGGGAGAAGCAC
GAAGATAAAGAAAAGTATCTAAACTGGATTCGAAATGTTGATAACTTCATGACTCCTTATGTGTCCCAAAATC
CAAGATTGACATATCTCAATTATAGACACCTTGATATAGGAATAAATGATCCCAAGAGTCAAAATAATTACAC
ACAAGCATGTATTTGGGGTGAGAAGTATTTTGGTAAAAATTTTGACAGGCTAGTAAAAGTGAAAACCCTGGTT
GATCTCGATAATTTTTTTAGAAATGAACAAAGCATCCCACCTCTTCCACCGCATCGTCATAAATGA
>B2_PBBK_MXBD01008229.1_20123
ATGAAGTACTCAACATTCTCCTTTTGGTTTGTTTGCATAATATTTTTCTTTCTCTCATTCAATATCCAAACTT
CAATTACTAATCCTCGAGAAAACTTCCTTAAATGCTTCTCGCAATATATTCCCACCAATGTAACAAATCTAAA

ATACACTCAAAACAACCAATTGTATATGCCTGCCCAAAATTCGACAATACACAATCTTAGATTCACCTCTGAC
ACAACCCCAAAACCACTTGTTATCGTCACTCCTTCACATGTCTCCCATATCCAAGGCACTATTCTATGCTCCA
AGAAAATTGGTTTGCAAATTCGAACTCGAAGCGGTGGTCATGATTCTGAAGGCATGTCCTACATATCTCAAGT
CCCATTTGTTATAGTAGACTTGAGAAACATGCATTCAATCAAAATAGATGTTCATAGCCAAACTACATGGGTT
GAAGCCGGAGCTACCCTTGGAGAAGTTTATTATTGGATTAATGAGAAAAATGAGAATCTTAGTTTGGGTGCTG
GGTATTGCCCTACTGTTTGCGCAGCTGGACACTTTGGTGGAGGAGGCTATGGACCATTGATGTGAAATTATGG
CGCAGCTGATAATATCGTTGATGCACACTTAGTCAACGTTGATAAAGTGCTAGATCGAAAAATGGGAGAAGAT
CTCTTTTGGGCTATACGTGGTGGTGGAGAAAGCTTCAGAATCATTGTAGCATGGAAAATTAGATTGGTTGCTA
TCCCAACAAAGTCTACTATGTTTAGTGTTAAAAAGATCAAGGAGATACATGAGCTTGTGAAGTTAGTTAACAA
GTGGCAAAACATTTCTTACAAGTATGACGATTTATTACTCATGACTCACTTCATAAGGAATATTATAGATAAT
CAAGGGAAGAATAAGACAACACACACTTACTTCTCTTTAGTTTTCCTTGGTGGAGTGGATAGTCTAGTCGACT
TAATGAACAAGAGTTTTCCTGAGTTTGGCATTAAAATAGATTGCAAACAATTGAGCTGGATTGATACTATCAT
CTTCTATAGCGGTGTTGTAAATTACGGCACTGATAATTTTAATAAGGAAATTTTGCTTGATAGATCAGCTGGG
CAGAACGGTGCTTTCAAGATTAAGTTAGACTACGTTAAGAAACCAATTCCAGAATCTGTGTTTGTCAAAATTT
TGGAAAAATTATATGAAGAAGATGAAGGAGTTGGGATGTATGCGTTGTACCCTTACGGTTGTATAATGGATGA
GATTTCAGAATCAGCAATTCCATTCCCTCATCGAGTTGGAATCATGTATGAATTATGGTACATATGTAGCTGG
GAGAAGCACGAAGATAAAGAAAAGTATCTAAACTGGATTCGAAATGTTGATAACTTCATGACTCCTTATGTGT
CCCAAAATCCAAGATTGACATATCTCAATTATAGACACCTTGATATAGGAATAAATGATCCCAAGAGTCAAAA
TAATTACACACAATCATGTATTTGGGGTGAGAAGTTTTGGTAAAATTTTGACAGGCTAGTAAAAGTGAAAACC
CTGGTTGATCTCGATAATTTTTTTAGAAATGAACAAAGCATCCCACCTCTTCCACCGCATCGTCATAAA
>B2_Cannatonic_MNPR01008230_6385
ATGAAGTACTCAACATTCTCCTTTTGGTTTGTTTGCAAGATAATATTTTTCTTTCTCTCATTCAATATCCAAA
CTTCAATTGCTAATCCTCGAGAAAACTTCCTTAAATGCTTCTCGCAATATATTCCCACCAATGTAACAAATCT
AAAATACACTCAAAACAACCAATTGTATATGCCTGTCCAAAATTCGACAATACACAATCTTAGATTCACCTCT
AACAGAACCCCAAAACCACTTGTTATCGTCACTCCTTCACATGTCTCCCATATCCAAGGCACTATTCTATCCA
AGAAAATTGGTTTGCAAATTCAAACTCGAAACGGTGGTCATGATTCTGAAGGCATGTCCCACATATCTCAAGT
CCCATTTGTTATAGTAGACTTGAGAAACATGCATTCAATCAAAATAGATGTTCATAGCCAAACTACATGGGTT
GAAGCCGGAGCTACCATTGGAGAAGTTTATTATTGGGTTAATGAGAAAAATGAGAATCTTAGTTTGGGTGCTG
GGTATTGCCCTACTATTTGCGCAGCTGGACACTTTGGTGGAGGAGGCTATGGACCATTGATGTGAAATTATGG
CCTCGCGGATGATAATATCGTTGATGCACACTTAGTCAACGTTGATGGAAAAGTACTAGATCGAAAATCTATG
GGAGATCTCTTTTGGGCTATACGTGGTGGTGGAAGAGAAAGCTTCAGAATCATTGTAGCATGGAAAATTAGAC
TGGTTGCTGTCCCAACAAAGTCTACTATGTTTAGTGTTAAAAAGATCAAGGAGATACATGAGCTTGTGAAGTT
AGTTAACAAGTGGCAAAATATTTACAAGTATGACATAGATTTATTACTCATGACTCACTTCATAACTAGGAAT
ATTACAGATAATCAAGGGAAGAATAAGACAACAATACACACTTACTTCTCTTTAGTTTTCCTTGGTGGAGTGG
ATAGTCTAGTCGACTTAATGAACAAGAGTTTTCCTGAGTTTGGTATTAAAATAGATTGCAAACAATTGAGCTG
GATTGATACTATCATCTTCTATAGCGGTGTTGTAAATTACGGCACTGATAATTTTAATAACCAAATTTCGCTT
GTTAGATCAGCTGGGCAGAACGGTGCTTTCAAGATTAAGTTAGACTATGTTAAGAAACCAATTCCAGAATCTG
CATTTGTCAAAATTTTGGAAAAATTATATGAAGAAGATAAAGGAGTTGGGATGTATGCGTTGTACCCTTACGG
TTGTCTAATGGATGAGATTTCAGAATCAGCAATTCCATTCCCTCATCGAGGAATCATGTATGAATTATGGTAC
ATATGTAGCTGGGAGAAGCACGAAGATAAAGAAAAGTATCTAAACTGGATTCGAAATGTTGATAACTTCATGA
CTCCTTATGTGCAAAATCCAAGATTGACATATCTCAATTATAGACATCTTGATATAGGAATAAATGATCCCAA
GAGTAATAATTACACAGAAGCATGTATTTGGGGTGAGAAATTTGGTAAAAATTTTGACAGGCTAGTAAAAGTG
AAAACCCTGGTTGATTTCAATAATCTTTTTAGAAATGAACAAAGCATCCCACCTCTTCCACCGCATCGTCATT
AA
>B2_Chemdog91_LKUB01047298.1_2
CCATTGATGTGAAATTATGGCCTCGCGGATGATAATATCGTTGATGCACACTTAGTCAACGTTGATGGAAAAG
TACTAGATCGAAAATCTATGGGACAAGATCTCTTTTGGGCTATACGTGGTGGTGGAAGAGAAAGCTTCAGAAT
CATTGTAGCATGGAAAATTAGACTGGTTGCTGTCCCAACAAAGTCTACTATGTTTAGTGTTAAAAAGATCAAG
GAGATACATGAGCTTGTGAAGTTAGTTAACAAGTGGCAAAATATTTCTTACAAGTATGACATAGATTTATTAC
TCATGACTCACTTCATAACTAGGAATATTACAGATAATCAAGGGAAGAATAAGACAACAATACACACTTACTT
CTCTTTAGTTTTCCTTGGTGGAGTGGATAGTCTAGTCGACTTAATGAACAAGAGTTTTCCTGAGTTTGGTATT
AAAAAAATAGATTGCAAACAGTTGAGCTGGATTGATACTATCATCTTCTATAGCGGTGTTGTAAATTACGGCA
CTGATAATTTTAATAACCAAATTTCGCTTGTTAGATCAGCTGGGCAGAACGGTGCTTTCAAGATTAAGTTAGA
CTATGTTAAGAAACCAATTCCAGAATCTGCATTTGTCAAAATTTTGGAAAAATTATATGAAGAAGATAAAGGA
GTTGGGATGTATGCGTTGTACCCTTACGGTTGTCTAATGGATGAGATTTCAGAATCAGCAATTCCATTCCCTC
ATCGAGTTGGAATCATGTATGAATTATGGTACATATGTAGCTGGGAGAAGCACGAAGATAAAGAAAAGTATCT
AAACTGGATTCGAAATGTTGATAACTTCATGACTCCTTATGTGTCCCAAAATCCAAGATTGACATATCTCAAT
TATAGACATCTTGATATAGGAATAAATGATCCCAAGAGTCAAAATAATTACACAGAAGCATGTATTTGGGGTG

AGAAATTTGGTAAAAATTTTGACAGGCTAGTAAAAGTGAAAACCCTGGTTGATTTCAATAATCTTTTTAGAAA
TGAACAAAGCATCCCACCTCTTCCACCGCATCGTCATTAA
>C_Finola_QKVJ02000019.1_742544
AAATCCATGGGGGAAGATTTGTTTTGGGCTATACGTGGTGGTGGAGGAGAAAACTTTGGAATCATTGCAGCGT
GGAAAATTAGACTTGTTGCTGTCCCATCAATGTCTACTATATTCAGTAATAAAAAGAACATGGAGATACATGA
GCTTGTCAAGTTAGTTAACAAATGGCAAAATATTGCTTACATGTATGAAAAAGAATTATTACTCTTTACTCAC
TTTATAACCAGGAATATTACAGATAATCAAGGAAAGAATAAGACAACAATACATAGTTACTTCTCCTCCATTT
TCCATGGTGGAGTGGATAGTCTAGTCGACTTGATGAACAAGAGCTTTCCTGAATTGGGTATTAAAAAAACAGA
TTGCAAACAGTTGAGCTGGATTGATACTATCATCTTCTACAGTGGTCTTGTAAATTACAACACTACTAATTTT
AAAAAAGAACTTTTGCTTGATAGATCAGGTGGGCGGAAGGCGGCTTTCTCGATTAAGTTAGACTAAGTTAAGA
AACCGATTCCAGAAACCGCAATGGTCACAATTTTGGAAAAATTATATGAAGAAGATGTAGGAGTTGGGATGTT
TGTGTTTTACCCTTATGGTGGAATAATGGATGAGATTTCAGAATCAGCAATTCCATTCCCTCATCGAGCTGGA
ATCATGTATGAAATTTGGTACATAGCTTCATGGGAGAAGCAAGAAGATAATGAAAAGCATATAAACTGGATTC
AGAATGTTTACAATTTCACGACTCCTTATGTGTCCCAAAATCCAAGAATGGCGTATCTCAATTATAGGGACCT
TGATTTAGGAAAAACTAATTTCGAGAGTCCTAATAATTACACACAAGCACGTATTTGGGGTGAAAAGTATTTT
GGTAAAAAATTTAATAGGTTAGTAAAAGTAAAAAAGGTTGATCCCGATAATTTCTTTAGAAACGAACAAAGCA
TCCCACCTCTTCCCCTACGTCATCATTAA
>C_CBDRx_NC_044378.1_LOC115696475
AAATCCATGGGGGAAGATTTGTTTTGGGCTATACGTGGTGGTGGAGGAGAAAACTTTGGAATCATTGCAGCGT
GGAAAATTAGACTTGTTGCTGTCCCATCAATGTCTACTATATTCAGTAATAAAAAGAACATGGAGATACATGA
GCTTGTCAAGTTAGTTAACAAATGGCAAAATATTGCTTACATGTATGAAAAAGAATTATTACTCTTTACTCAC
TTTATAACCAGGAATATTACAGATAATCAAGGAAAGAATAAGACAACAATACATAGTTACTTCTCCTCCATTT
TCCATGGTGGAGTGGATAGTCTAGTCGACTTGATGAACAAGAGCTTTCCTGAATTGGGTATTAAAAAAACAGA
TTGCAAACAGTTGAGCTGGATTGATACTATCATCTTCTACAGTGGTCTTGTAAATTACAACACTACTAATTTT
AAAAAAGAACTTTTGCTTGATAGATCAGGTGGGCGGAAGGCGGCTTTCTCGATTAAGTTAGACTAAGTTAAGA
AACCGATTCCAGAAACCGCAATGGTCACAATTTTGGAAAAATTATATGAAGAAGATGTAGGAGTTGGGATGTT
TGTGTTTTACCCTTATGGTGGAATAATGGATGAGATTTCAGAATCAGCAATTCCATTCCCTCATCGAGCTGGA
ATCATGTATGAAATTTGGTACATAGCTTCATGGGAGAAGCAAGAAGATAATGAAAAGCATATAAACTGGATTC
AGAATGTTTACAATTTCACGACTCCTTATGTGTCCCAAAATCCAAGAATGGCGTATCTCAATTATAGGGACCT
TGATTTAGGAAAAACTAATTTCGAGAGTCCTAATAATTACACACAAGCACGTATTTGGGGTGAAAAGTATTTT
GGTAAAAAATTTAATAGGTTAGTAAAAGTAAAAACCAAGGTTGATCCCGATAATTTCTTTAGAAACGAACAAA
GCATCCCACCTCTTCCCCTACGTCATCATTAA
>C_Jamaican_Lion_JAATIP010000103.1_F8388_004081
AAATCCATGGGGGAAGATTTGTTTTGGGCTATACGTGGTGGTGGAGGAGAAAACTTTGGAATCATTGCAGCGT
GGAAAATTAGACTTGTTGCTGTCCCATCAATGTCTACTATATTCAGTAATAAAAAGAACATGGAGATACATGA
GCTTGTCAAGTTAGTTAACAAATGGCAAAATATTGCTTACATGTATGAAAAAGAATTATTACTCTTTACTCAC
TTTATAACCAGGAATATTACAGATAATCAAGGAAAGAATAAGACAACAATACATAGTTACTTCTCCTCCATTT
TCCATGGTGGAGTGGATAGTCTAGTCGACTTGATGAACAAGAGCTTTCCTGAATTGGGTATTAAAAAAACAGA
TTGCAAACAGTTGAGCTGGATTGATACTATCATCTTCTACAGTGGTCTTGTAAATTACAACACTACTAATTTT
AAAAAAGAACTTTTGCTTGATAGATCAGGTGGGCGGAAGGCGGCTTTCTCGATTAAGTTAGACTAAGTTAAGA
AACCGATTCCAGAAACCGCAATGGTCACAATTTTGGAAAAATTATATGAAGAAGATGTAGGAGTTGGGATGTT
TGTGTTTTACCCTTATGGTGGAATAATGGATGAGATTTCAGAATCAGCAATTCCATTCCCTCATCGAGCTGGA
ATCATGTATGAAATTTGGTACATAGCTTCATGGGAGAAGCAAGAAGATAATGAAAAGCATATAAACTGGATTC
AGAATGTTTACAATTTCACGACTCCTTATGTGTCCCAAAATCCAAGAATGGCGTATCTCAATTATAGGGACCT
TGATTTAGGAAAAACTAATTTCGAGAGTCCTAATAATTACACACAAGCACGTATTTGGGGTGAAAAGTATTTT
GGTAAAAAATTTAATAGGTTAGTAAAAGTAAAAACCAAGGTTGATCCCGATAATTTCTTTAGAAACGAACAAA
GCATCCCACCTCTTCCCCTACGTCATCATTAA
>C_Finola_QKVJ02000019.1_589417
ATGAAGTACTCAACATTCTGTTTTTGGTATGTTTGCAAGATATTTTTCTTTCTCTCATTCAATATCCAAATTT
CAGCTAATCCTCAAGAAAACTTCCTTAACTGCCTCTCACAATATATTCCCACCAATGTAACAAATGCAAAACT
CGTATACACTCAACACGACCAATTTTATATGTCTATCTTAAATTCGACCGTAAATCTTAGATTTACCTCTGAC
ACAACCCCAAACCTTGTTATCACCACTTTAAATGTCTCCCATCAAGGCACTATTTGTTCCAAGAAAGTTGGCT
TGCAGATTCAAACTCGAAGCGGTGGTCATGATGCTGAGGGCATGTCCTACATATCTCAAGTCCCATTTGTTAT
AGTAGACTTGAGAAACATGCATTCGGTCAAAATAGATGTTCATAGCCAAACTGCATGGGTTGAATCCGGAGCT
ACCCTTGGAGAAGTTTATTATTGGATCAATGAGAACAATGAGAATCTTAGTTTTCCTGCTGGGTACTGCCCTA
CTGTTGCGGGTGGACACTTTAGTGGAGGAGGCTATGGAGCATTGATGCGAAATTATGGCCTCGCGGCTGATAA
TATCATTGATGCGCACTTAGTCAATGTTGATGGAAAAGTTTTAGATCGAAAATCCATGGGGGAAGATTTGTTT
TGGGCTATACGTGGTGGTGGAGGAGAAAACTTTGGAATCATTGCAGCGTGGAAAATTAGACTTGTTGCTGTCC

CATCAATGTCTACTATATTCAGTGTTAAAAAGAACATGGAGATACATGAGCTTGTCAAGTTAGTTAACAAATG
GCAAAATATTGCTTACATGTATGAAAAAGAATTATTACTCTTTACTCACTTTATAACCAGGAATATTACAGAT
AATCAAGGGAAGAATAAGACAACAATACACAGTTACTTCTCCTCCATTTTCCATGGTGGAGTGGATAGTCTAG
TCGACTTGATGAACAAGAGCTTTCCTGAATTGGGTATTAAAAAAACAGATTGCAAACAGTTGAGCTGGATTGA
TACTATCATCTTCTACAGTGGTGTTGTAAATTACAACACAACTAATTTTAAAAAAGAAATTTTGCTTGATAGA
TCAGGTGGGCGGAAGGCGGCTTTCTCGATTAAGTTAGACTATGTTAAGAAACCGATTCCAGAAACCGCAATGG
TCACAATTTTGGAAAAATTATATGAAGAAGATGTAGGAGTTGGGATGTTTGTGTTTTACCCTTATGGTGGTAT
AATGGATGAGATTTCAGAATCAGCAATTCCATTCCCTCATCGAGCTGGAATCATGTATGAAATTTGGTACATA
GCTTCATGGGAGAAGCAAGAAGATAATGAAAAGCATATAAACTGGATTCGGAATGTTTATAATTTCACGACTC
CTTATGTGTCCCAAAATCCAAGAATGGCGTATCTCAATTATAGGGACCTTGATTTAGGAAAAACTAATTTCGA
GAGTCCTAATAATTACACACAAGCACGTATTTGGGGTGAAAAGTATTTTGGTAAAAATTTTAATAGGTTAGTA
AAAGTAAAAACCAAGGTTGATCCCGATAATTTCTTTAGAAACGAACAAAGCATCCCACCTCTTCCCCTGCGTC
ATCATTAA
>C_Finola_QKVJ02000019.1_535563
ATGAAGTACTCAACATTCTGTTTTTGGTATGTTTGCAAGATAATATTCTTTCTCTCATTCAATATCCAAATTT
CAATAGCTAATCCTCAAGAAAACAAATGCTTCTCACAATATATTCCCACCAATGTAACAAATGCAAAACTCGT
ATACACTCAACACGACCAATTTTATATGTCTATCCTAAATTCGACCATACAAAATCTTAGATTTACCTCTGAC
ACAACCCCAAAACCACTTGTTATCATCACTCCTTTAAATGTCTCCCATATCCAAGGCACTATTCTATGCTCCA
AGAAAGTTGGCTTGCAGATTCGAACTCGAAGCGGTGGTCATGATGCTGAGGGCATGTCCTACATATCTCAAGT
CCCATTTGTTATAGTAGACTTGAGAAACATGCATTCGGTCAAAATAGATGTTCATAGCCAAACTGCATGGGTT
GAAGCCGGAGCTACCCTTGGAGAAGTTTATTATTGGATCAATGAGAACAATGAGAATCTTAGTTTTCCTGCTG
GGTACTGCCCTACTGTTGGCGCGGGTGGACACTTTAGTGGAGGAGGCTATGGAGCATTGATGCGAAATTATGG
CCTCGCGGCTGATAATATCATTGATGCGCACTTAGTCAATGTTGATGGAAAAGTTTTAGATCGAAAATCCATG
GGGGAAGATTTGTTTTGGGCTATACGTGGTGGTGGAGGAGAAAACTTTGGAATCATTGCAGCGTGGAAAATTA
GACTTGTTGCTGTCCCATCAATGTCTACTATATTCAGTGTTAAAAAGAACATGGAGATACATGAGCTTGTCAA
GTTAGTTAACAAATGGCAAAATATTGCTTACATGTATGAAAAAGAATTATTACTCTTTACTCACTTTATAACC
AGGAATATTACAGATAATCAAGGGAAGAATAAGACAACAATACACAGTTACTTCTCCATTTTCCATGGTGGAG
TGGATAGTCTAGTCGACTTGATGAACAAGAGCTTTCCTGAATTGGGTATTAAAAAAACAGATTGCAAACAGTT
GAGCTGGATTGATACTATCATCTTCTACAGTGGTGTTGTAAATTACAACACAACTAATTTTAAAAAAGAAATT
TTGCTTGATAGATCAGGTGGGCGGAAGGCGGCTTTCTCGATTAAGTTAGACTATGTTAAGAAACCGATTCCAG
AAACCGCAATGGTCACAATTTTGGAAAAATTATATGAAGAAGATGTAGGAGTTGGGATGTTTGTGTTTTACCC
TTATGGTGGTATAATGGATGAGATTTCAGAATCAGCAATTCCATTCCCTCATCGAGCTGGAATCATGTATGAA
ATTTGGTACATAGCTTCATGGGAGAAGCAAGAAGATAATGAAAAGCATATAAACTGGATTCGGAATGTTTATA
ATTTCACGACTCCTTATGTGTCCCAAAATCCAAGAATGGCGTATCTCAATTATAGGGACCTTGATTTAGGAAA
AACTAATTTCGAGAGTCCTAATAATTACACACAAGCACGTATTTGGGGTGAAAAGTATTTTGGTAAAAATTTT
AATAGGTTAGTAAAAGTAAAAACCAAGGTTGATCCCGATAATTTCTTTAGAAACGAACAAAGCATCCCACCTC
TTCCCCTGCGTCATCATTAA
>C_Finola_QKVJ02000019.1_618430
ATGAAGTACTCAACATTCTGTTTTTGGTATGTTTGCAAGATAATATTTTTCTTTCTCTCATTCAATATCCAAA
TTTCAATAGCTAATCCTCAAGAAAACTTCCTTAAATGCTTCTCACAATATATTCCCACCAATGTAACAAATGC
AAAACTCGTATACACTCAACACGACCAATTTTATATGTCTATCCTAAATTCGACCATACAAAATCTTAGATTT
ACCTCTGACACAACCCCAAAACCACTTGTTATCATCACTCCTTTAAATGTCTCCCATATCCAAGGCACTATTC
TATGCTCCAAGAAAGTTGGCTTGCAGATTCGAACTCGAAGCGGTGGTCATGATGCTGAGGGCATGTCCTACAT
ATCTCAAGTCCCATTTGTTATAGTAGACTTGAGAAACATGCATTCGGTCAAAATAGATGTTCATAGCCAAACT
GCATGGGTTGAAGCCGGAGCTACCCTTGGAGAAGTTTATTATTGGATCAATGAGAACAATGAGAATCTTAGTT
TTCCTGCTGGGTACTGCCCTACTGTTGGCGCGGGTGGACACTTTAGTGGAGGAGGCTATGGAGCATTGATGCG
AAATTATGGCCTCGCGGCTGATAATATCATTGATGCGCACTTAGTCAATGTTGATGGAAAAGTTTTAGATCGA
AAATCCATGGGGGAAGATTTGTTTTGGGCTATACGTGGTGGTGGAGGAGAAAACTTTGGAATCATTGCAGCGT
GGAAAATTAGACTTGTTGCTGTCCCATCAATGTCTACTATATTCAGTGTTAAAAAGAACATGGAGATACATGA
GCTTGTCAAGTTAGTTAACAAATGGCAAAATATTGCTTACATGTATGAAAAAGAATTATTACTCTTTACTCAC
TTTATAACCAGGAATATTACAGATAATCAAGGGAAGAATAAGACAACAATACACAGTTACTTCTCCTCCATTT
TCCATGGTGGAGTGGATAGTCTAGTCGACTTGATGAACAAGAGCTTTCCTGAATTGGGTATTAAAAAAACAGA
TTGCAAACAGTTGAGCTGGATTGATACTATCATCTTCTACAGTGGTGTTGTAAATTACAACACAACTAATTTT
AAAAAAGAAATTTTGCTTGATAGATCAGGTGGGCGGAAGGCGGCTTTCTCGATTAAGTTAGACTATGTTAAGA
AACCGATTCCAGAAACCGCAATGGTCACAATTTTGGAAAAATTATATGAAGAAGATGTAGGAGTTGGGATGTT
TTTTTACCCTTATGGTGGTATAATGGATGAGATTTCAGAATCAGCAATTCCATTCCCTCATCGAGCTGGAATC
ATGTATGAAATTTGGTACATAGCTTCATGGGAGAAGCAAGAAGATAATGAAAAGCATATAAACTGGATTCGGA
ATGTTTATAATTTCACGACTCCTTATGTGTCCCAAAATCCAAGAATGGCGTATCTCAATTATAGGGACCTTGA

TTTAGGAAAAACTAATTTCGAGAGTCCTAATAATTACACACAAGCACGTATTTGGGGTGAAAAGTATTTTGGT
AAAAATTTTAATAGGTTAGTAAAAGTAAAAACCAAGGTTGATCCCGATAATTTCTTTAGAAACGAACAAAGCA
TCCCTCTTCCCCTGCGTCATCATTAA
>C_Finola_QKVJ02000019.1_709761
ATGAAGTACTCAACATTCTGTTTTTGGTATGTTTGCAAGATAATATTTTTCTTTCTCTCATTCAATATCCAAA
TTTCAATAGCTAATCCTCAAGAAAACTTCCTTAAATGCTTCTCACAATATATTCCCACCAATGTAACAAATGC
AAAACTCGTATACACTCAACACGACCAATTTTATATGTCTATCCTAAATTCGACCATACAAAATCTTAGATTT
ACCTCTGAAACCCCAAAACCACTTGTTATCATCACTCCTTTAAATGTCTCCCATATCCAAGGCACTATTCTAT
GCTCCAAGAAAGTTGGCTTGCAGATTCGAACTCGAAGCGGTGGTCATGATGCTGAGGGCATGTCCTACATATC
TCAAGTCCCATTTGTTATAGTAGACTTGAGAAACATGCATTCGGTCAAAATAGATGTTCATAGCCAAACTGCA
TGGGTTGAAGCCGGAGCTACCCTTGGAGAAGTTTATTATTGGATCAATGAGAACAATGAGAATCTTAGTTTTC
CTGCTGGGTACTGCCCTACTGTTGGCGCGGGTGGACACTTTAGTGGAGGAGGCTATGGAGCATTGATGCGAAA
TTATGGCCTCGCGGCTGATAATATCATTGATGCGCACTTAGTCAATGTTGATGGAAAAGTTTTAGATCGAAAA
TCCATGGGGGAAGATTTGTTTTGGGCTATACGTGGTGGTGGAGGAGAAAACTTTGGAATCATTGCAGCGTGGA
AAATTAGACTTGATGCTGTCCCATCAATGTCTACTATATTCAGTGTTAAAAAGAACATGGAGATACATGAGCT
TGTCAAGTTAGTTAACAAATGGCAAAATATTGCTTACATGTATGAAAAAGAATTATTACTCTTTACTCACTTT
ATAACCAGGAATATTACAGATAATCAAGGGAAGAATAAGACAACAATACACAGTTACTTCTCCTCCATTTTCC
ATGGTGGAGTGGATAGTCTAGTCGACTTGATGAACAAGAGCTTTCCTGAATTGGGTATTAAAAAAACAGATTG
CAAACAGTTGAGCTGGATTGATACTATCATCTTCTACAGTGGTGTTGTAAATTACAACACAACTAATTTTAAA
AAAGAAATTTTGCTTGATAGATCAGGTGGGCGGAAGGCGGCTTTCTCGATTAAGTTAGACTATGTTAAGAAAC
CGATTCCAGAAACCGCAATGGTCACAATTTTGGAAAAATTATATGAAGAAGATGTAGGAGTTGGGATGTTTGT
GTTTTACCCTTATGGTGGTATAATGGATGAGATTTCAGAATCAGCAATTCCATTCCCTCATCGAGCTGGAATC
ATGTATGAAATTTGGTACATAGCTTCATGGGAGAAGCAAGAAGATAATGAAAAGCATATAAACTGGATTCGGA
ATGTTTATAATTTCACGACTCCTTATGTGTCCCAAAATCCAAGAATGGCGTATCTCAATTATAGGGACCTTGA
TTTAGGAAAAACTAATTTCGAGAGTCCTAATAATTACACACAAGCACGTATTTGGGGTGAAAAGTATTTTGGT
AAAAATTTTAATAGGTTAGTAAAAGTAAAAACCAAGGTTGATCACGATAATTTCTTTAGAAACGAACAAAGCA
TCCCACCTCTTCCCCTGCGTCATCATTAA
>C_CBDRx_NC_044378.1_LOC115696884
ATGAAGTACTCAACATTCTGTTTTTGGTATGTTTGCAAGATAATATTTTTCTTTCTCTCATTCAATATCCAAA
TTTCAATAGCTAATCCTCAAGAAAACTTCCTTAAATGCTTCTCACAATATATTCCCACCAATGTAACAAATGC
AAAACTCGTATACACTCAACACGACCAATTTTATATGTCTATCCTAAATTCGACCATACAAAATCTTAGATTT
ACCTCTGAAACAACCCCAAAACCACTTGTTATCATCACTCCTTTAAATGTCTCCCATATCCAAGGCACTATTC
TATGCTCCAAGAAAGTTGGCTTGCAGATTCGAACTCGAAGCGGTGGTCATGATGCTGAGGGCATGTCCTACAT
ATCTCAAGTCCCATTTGTTATAGTAGACTTGAGAAACATGCATTCGGTCAAAATAGATGTTCATAGCCAAACT
GCATGGGTTGAAGCCGGAGCTACCCTTGGAGAAGTTTATTATTGGATCAATGAGAACAATGAGAATCTTAGTT
TTCCTGCTGGGTACTGCCCTACTGTTGGCGCGGGTGGACACTTTAGTGGAGGAGGCTATGGAGCATTGATGCG
AAATTATGGCCTCGCGGCTGATAATATCATTGATGCGCACTTAGTCAATGTTGATGGAAAAGTTTTAGATCGA
AAATCCATGGGGGAAGATTTGTTTTGGGCTATACGTGGTGGTGGAGGAGAAAACTTTGGAATCATTGCAGCGT
GGAAAATTAGACTTGTTGCTGTCCCATCAATGTCTACTATATTCAGTGTTAAAAAGAACATGGAGATACATGA
GCTTGTCAAGTTAGTTAACAAATGGCAAAATATTGCTTACATGTATGAAAAAGAATTATTACTCTTTACTCAC
TTTATAACCAGGAATATTACAGATAATCAAGGGAAGAATAAGACAACAATACACAGTTACTTCTCCTCCATTT
TCCATGGTGGAGTGGATAGTCTAGTCGACTTGATGAACAAGAGCTTTCCTGAATTGGGTATTAAAAAAACAGA
TTGCAAACAGTTGAGCTGGATTGATACTATCATCTTCTACAGTGGTGTTGTAAATTACAACACAACTAATTTT
AAAAAAGAAATTTTGCTTGATAGATCAGGTGGGCGGAAGGCGGCTTTCTCGATTAAGTTAGACTATGTTAAGA
AACCGATTCCAGAAACCGCAATGGTCACAATTTTGGAAAAATTATATGAAGAAGATGTAGGAGTTGGGATGTT
TGTGTTTTACCCTTATGGTGGTATAATGGATGAGATTTCAGAATCAGCAATTCCATTCCCTCATCGAGCTGGA
ATCATGTATGAAATTTGGTACATAGCTTCATGGGAGAAGCAAGAAGATAATGAAAAGCATATAAACTGGATTC
GGAATGTTTATAATTTCACGACTCCTTATGTGTCCCAAAATCCAAGAATGGCGTATCTCAATTATAGGGACCT
TGATTTAGGAAAAACTAATTTCGAGAGTCCTAATAATTACACACAAGCACGTATTTGGGGTGAAAAGTATTTT
GGTAAAAATTTTAATAGGTTAGTAAAAGTAAAAACCAAGGTTGATCCCGATAATTTCTTTAGAAACGAACAAA
GCATCCCACCTCTTCCCCTACGTCATCATTAA
>C_Finola_QKVJ02000019.1_650928
ATGAAGTACTCAACATTCTGTTTTTGGTATGTTTGCAAGATAATATTTTTCTTTCTCTCATTCAATATCCAAA
TTTCAATAGCTAATCCTCAAGAAAACTTCCTTAAATGCTTCTCACAATATATTCCCACCAATGTAACAAATGC
AAAACTCGTATACACTCAACACGACCAATTTTATATGTCTATCCTAAATTCGACCATACAAAATCTTAGATTT
ACCTCTGAAACAACCCCAAAACCACTTGTTATCATCACTCCTTTAAATGTCTCCCATATCCAAGGCACTATTC
TATGCTCCAAGAAAGTTGGCTTGCAGATTCGAACTCGAAGCGGTGGTCATGATGCTGAGGGCATGTCCTACAT
ATCTCAAGTCCCATTTGTTATAGTAGACTTGAGAAACATGCATTCGGTCAAAATAGATGTTCATAGCCAAACT

GCATGGGTTGAAGCCGGAGCTACCCTTGGAGAAGTTTATTATTGGATCAATGAGAACAATGAGAATCTTAGTT
TTCCTGCTGGGTACTGCCCTACTGTTGGCGCGGGTGGACACTTTAGTGGAGGAGGCTATGGAGCATTGATGCG
AAATTATGGCCTCGCGGCTGATAATATCATTGATGCGCACTTAGTCAATGTTGATGGAAAAGTTTTAGATCGA
AAATCCATGGGGGAAGATTTGTTTTGGGCTATACGTGGTGGTGGAGGAGAAAACTTTGGAATCATTGCAGCGT
GGAAAATTAGACTTGTTGCTGTCCCATCAATGTCTACTATATTCAGTGTTAAAAAGAACATGGAGATACATGA
GCTTGTCAAGTTAGTTAACAAATGGCAAAATATTGCTTACATGTATGAAAAAGAATTATTACTCTTTACTCAC
TTTATAACCAGGAATATTACAGATAATCAAGGGAAGAATAAGACAACAATACACAGTTACTTCTCCTCCATTT
TCCATGGTGGAGTGGATAGTCTAGTCGACTTGATGAACAAGAGCTTTCCTGAATTGGGTATTAAAAAAACAGA
TTGCAAACAGTTGAGCTGGATTGATACTATCATCTTCTACAGTGGTGTTGTAAATTACAACACAACTAATTTT
AAAAAAGAAATTTTGCTTGATAGATCAGGTGGGCGGAAGGCGGCTTTCTCGATTAAGTTAGACTATGTTAAGA
AACCGATTCCAGAAACCGCAATGGTCACAATTTTGGAAAAATTATATGAAGAAGATGTAGGAGTTGGGATGTT
TGTGTTTTACCCTTATGGTGGTATAATGGATGAGATTTCAGAATCAGCAATTCCATTCCCTCATCGAGCTGGA
ATCATGTATGAAATTTGGTACATAGCTTCATGGGAGAAGCAAGAAGATAATGAAAAGCATATAAACTGGATTC
GGAATGTTTATAATTTCACGACTCCTTATGTGTCCCAAAATCCAAGAATGGCGTATCTCAATTATAGGGACCT
TGATTTAGGAAAAACTAATTTCGAGAGTCCTAATAATTACACACAAGCACGTATTTGGGGTGAAAAGTATTTT
GGTAAAAATTTTAATAGGTTAGTAAAAGTAAAAACCAAGGTTGATCCCGATAATTTCTTTAGAAACGAACAAA
GCATCCCACCTCTTCCCCTGCGTCATCATTAA
>C_Jamaican_Lion_JAATIP010000103.1_F8388_004084
ATGAAGTACTCAACATTCTGTTTTTGGTATGTTTGCAAGATAATATTTTTCTTTCTCTCATTCAATATCCAAA
TTTCAATAGCTAATCCTCAAGAAAACTTCCTTAAATGCTTCTCACAATATATTCCCACCAATGTAACAAATGC
AAAACTCGTATACACTCAACACGACCAATTTTATATGTCTATCCTAAATTCGACCATACAAAATCTTAGATTT
ACCTCTGAAACAACCCCAAAACCACTTGTTATCATCACTCCTTTAAATGTCTCCCATATCCAAGGCACTATTC
TATGCTCCAAGAAAGTTGGCTTGCAGATTCGAACTCGAAGCGGTGGTCATGATGCTGAGGGCATGTCCTACAT
ATCTGAAGTCCCATTTGTTATAGTAGACTTGAGAAACATGCATTCGGTCAAAATAGATGTTCATAGCCAAACT
GCATGGGTTGAAGCCGGAGCTACCCTTGGAGAAGTTTATTATTGGATCAATGAGAACAATGAGAATCTTAGTT
TTCCTGCTGGGTACTGCCCTACTGTTGGCGCGGGTGGACACTTTAGTGGAGGAGGCTATGGAGCATTGATGCG
AAATTATGGCCTCGCGGCTGATAATATCATTGATGCGCACTTAGTCAATGTTGATGGAAAAGTTTTAGATCGA
AAATCCATGGGGGAAGATTTGTTTTGGGCTATACGTGGTGGTGGAGGAGAAAACTTTGGAATCATTGCAGCGT
GGAAAATTAGACTTGTTGCTGTCCCATCAATGTCTACTATATTCAGTGTTAAAAAGAACATGGAGATACATGA
GCTTGTCAAGTTAGTTAACAAATGGCAAAATATTGCTTACATGTATGAAAAAGAATTATTACTCTTTACTCAC
TTTATAACCAGGAATATTACAGATAATCAAGGGAAGAATAAGACAACAATACACAGTTACTTCTCCTCCATTT
TCCATGGTGGAGTGGATAGTCTAGTCGACTTGATGAACAAGAGCTTTGCTGAATTGGGTATTAAAAAAACAGA
TTGCAAACAGTTGAGCTGGATTGATACTATCATCTTCTACAGTGGTGTTGTAAATTACAACACAACTAATTTT
AAAAAAGAAATTTTGCTTGATAGATCAGGTGGGCGGAAGGCGGCTTTCTCGATTAAGTTAGACTATGTTAAGA
AACCGATTCCAGAAACCGCAATGGTCACAATTTTGGAAAAATTATATGAAGAAGATGTAGGAGTTGGGATGTT
TGTGTTTTACCCTTATGGTGGTATAATGGATGAGATTTCAGAATCAGCAATTCCATTCCCTCATCGAGCTGGA
ATCATGTATGAAATTTGGTACATAGCTTCATGGGAGAAGCAAGAAGATAATGAAAAGCATATAAACTGGATTC
GGAATGTTTATAATTTCACGACTCCTTATGTGTCCCAAAATCCAAGAATGGCGTATCTCAATTATAGGGACCT
TGATTTAGGAAAAACTAATTTCGAGAGTCCTAATAATTACACACAAGCACGTATTTGGGGTGAAAAGTATTTT
GGTAAAAATTTTAATAGGTTAGTAAAAGTAAAAACCAAGGTTGATCCCGATAATTTCTTTAGAAACGAACAAA
GCATCCCACCTCTTCCCCTACGTCATCATTAA
>C_Jamaican_Lion_JAATIP010000103.1_F8388_004083
ATGAAGTACTCAACATTCTGTTTTTGGTATGTTTGCAAGATAATATTTTTCTTTCTCTCATTCAATATCCAAA
TTTCAATAGCTAATCCTCAAGAAAACTTCCTTAAATGCTTCTCACAATATATTCCCACCAATGTAACAAATGC
AAAACTCGTATACACTCAACACGACCAATTTTATATGTCTATCCTAAATTCGACCATACAAAATCTTAGATTT
ACCTCTGAAACAACCCCAAAACCACTTGTTATCATCACTCCTTTAAATGTCTCCCATATCCAAGGCACTATTC
TATGCTCCAAGAAAGTTGGCTTGCAGATTCGAACTCGAAGCGGTGGTCATGATGCTGAGGGCATGTCCTACAT
ATCTGAAGTCCCATTTGTTATAGTAGACTTGAGAAACATGCATTCGGTCAAAATAGATGTTCATAGCCAAACT
GCATGGGTTGAAGCCGGAGCTACCCTTGGAGAAGTTTATTATTGGATCAATGAGAACAATGAGAATCTTAGTT
TTCCTGCTGGGTACTGCCCTACTGTTGGCGCGGGTGGACACTTTAGTGGAGGAGGCTATGGAGCATTGATGCG
AAATTATGGCCTCGCGGCTGATAATATCATTGATGCGCACTTAGTCAATGTTGATGGAAAAGTTTTAGATCGA
AAATCCATGGGGGAAGATTTGTTTTGGGCTATACGTGGTGGTGGAGGAGAAAACTTTGGAATCATTGCAGCGT
GGAAAATTAGACTTGTTGCTGTCCCATCAATGTCTACTATATTCAGTGTTAAAAAGAACATGGAGATACATGA
GCTTGTCAAGTTAGTTAACAAATGGCAAAATATTGCTTACATGTATGAAAAAGAATTATTACTCTTTACTCAC
TTTATAACCAGGAATATTACAGATAATCAAGGGAAGAATAAGACAACAATACACAGTTACTTCTCCTCCATTT
TCCATGGTGGAGTGGATAGTCTAGTCGACTTGATGAACAAGAGCTTTCCTGAATTGGGTATTAAAAAAACAGA
TTGCAAACAGTTGAGCTGGATTGATACTATCATCTTCTACAGTGGTGTTGTAAATTACAACACAACTAATTTT
AAAAAAGAAATTTTGCTTGATAGATCAGGTGGGCGGAAGGCGGCTTTCTCGATTAAGTTAGACTATGTTAAGA

AACCGATTCCAGAAACCGCAATGGTCACAATTTTGGAAAAATTATATGAAGAAGATGTAGGAGTTGGGATGTT
TGTGTTTTACCCTTATGGTGGTATAATGGATGAGATTTCAGAATCAGCAATTCCATTCCCTCATCGAGCTGGA
ATCATGTATGAAATTTGGTACATAGCTTCATGGGAGAAGCAAGAAGATAATGAAAAGCATATAAACTGGATTC
GGAATGTTTATAATTTCACGACTCCTTATGTGTCCCAAAATCCAAGAATGGCGTATCTCAATTATAGGGACCT
TGATTTAGGAAAAACTAATTTCGAGAGTCCTAATAATTACACACAAGCACGTATTTGGGGTGAAAAGTATTTT
GGTAAAAATTTTAATAGGTTAGTAAAAGTAAAAACCAAGGTTGATCCCGATAATTTCTTTAGAAACGAACAAA
GCATCCCACCTCTTCCCCTACGTCATCATTAA
>C_Jamaican_Lion_JAATIP010000103.1_F8388_004082
ATGAAGTACTCAACATTCTGTTTTTGGTATGTTTGCAAGATAATATTTTTCTTTCTCTCATTCAATATCCAAA
TTTCAATAGCTAATCCTCAAGAAAACTTCCTTAAATGCTTCTCACAATATATTCCCACCAATGTAACAAATGC
AAAACTCGTATACACTCAACACGACCAATTTTATATGTCTATCCTAAATTCGACCATACAAAATCTTAGATTT
ACCTCTGAAACAACCCCAAAACCACTTGTTATCATCACTCCTTTAAATGTCTCCCATATCCAAGGCACTATTC
TATGCTCCAAGAAAGTTGGCTTGCAGATTCGAACTCGAAGCGGTGGTCATGATGCTGAGGGCATGTCCTACAT
ATCTGAAGTCCCATTTGTTATAGTAGACTTGAGAAACATGCATTCGGTCAAAATAGATGTTCATAGCCAAACT
GCATGGGTTGAAGCCGGAGCTACCCTTGGAGAAGTTTATTATTGGATCAATGAGAACAATGAGAATCTTAGTT
TTCCTGCTGGGTACTGCCCTACTGTTGGCGCGGGTGGACACTTTAGTGGAGGAGGCTATGGAGCATTGATGCG
AAATTATGGCCTCGCGGCTGATAATATCATTGATGCGCACTTAGTCAATGTTGATGGAAAAGTTTTAGATCGA
AAATCCATGGGGGAAGATTTGTTTTGGGCTATACGTGGTGGTGGAGGAGAAAACTTTGGAATCATTGCAGCGT
GGAAAATTAGACTTGTTGCTGTCCCATCAATGTCTACTATATTCAGTGTTAAAAAGAACATGGAGATACATGA
GCTTGTCAAGTTAGTTAACAAATGGCAAAATATTGCTTACATGTATGAAAAAGAATTATTACTCTTTACTCAC
TTTATAACCAGGAATATTACAGATAATCAAGGGAAGAATAAGACAACAATACACAGTTACTTCTCCTCCATTT
TCCATGGTGGAGTGGATAGTCTAGTCGACTTGATGAACAAGAGCTTTCCTGAATTGGGTATTAAAAAAACAGA
TTGCAAACAGTTGAGCTGGATTGATACTATCATCTTCTACAGTGGTGTTGTAAATTACAACACAACTAATTTT
AAAAAAGAAATTTTGCTTGATAGATCAGGTGGGCGGAAGGCGGCTTTCTCGATTAAGTTAGACTATGTTAAGA
AACCGATTCCAGAAACCGCAATGGTCACAATTTTGGAAAAATTATATGAAGAAGATGTAGGAGTTGGGATGTT
TGTGTTTTACCCTTATGGTGGTATAATGGATGAGATTTCAGAATCAGCAATTCCATTCCCTCATCGAGCTGGA
ATCATGTATGAAATTTGGTACATAGCTTCATGGGAGAAGCAAGAAGATAATGAAAAGCATATAAACTGGATTC
GGAATGTTTATAATTTCACGACTCCTTATGTGTCCCAAAATCCAAGAATGGCGTATCTCAATTATAGGGACCT
TGATTTAGGAAAAACTAATTTCGAGAGTCCTAATAATTACACACAAGCACGTATTTGGGGTGAAAAGTATTTT
GGTAAAAATTTTAATAGGTTAGTAAAAGTAAAAACCAAGGTTGATCCCGATAATTTCTTTAGAAACGAACAAA
GCATCCCACCTCTTCCCCTACGTCATCATTAA
>C_Jamaican_Lion_JAATIP010000103.1_F8388_004085
ATGAAGTACTCAACATTCTGTTTTTGGTATGTTTGCAAGATAATATTTTTCTTTCTCTCATTCAATATCCAAA
TTTCAATAGCTAATCCTCAAGAAAACTTCCTTAAATGCTTCTCACAATATATTCCCACCAATGTAACAAATGC
AAAACTCGTATACACTCAACACGACCAATTTTATATGTCTATCCTGAATTCGACCATACAAAATCTTAGATTT
ACCTCTGACACAACCCCAAAACCACTTGTTATCATCACTCCTTTAAATGTCTCCCATATCCAAGGCACTATTC
TATGCTCCAAGAAAGTTGGCTTGCAGATTCGAACTCGAAGCGGTGGTCATGATGCTGAGGGCATGTCCTACAT
ATCTCAAGTCCCATTTGTTATAGTAGACTTGAGAAACATGCATTCGGTCAAAATAGATGTTCATAGCCAAACT
GCATGGGTTGAAGCCGGAGCTACCCTTGGAGAAGTTTATTATTGGATCAATGAGAACAATGAGAATCTTAGTT
TTCCTGCTGGGTACTGCCCTACTGTTGGCGCGGGTGGACACTTTAGTGGAGGAGGCTATGGAGCATTGATGCG
AAATTATGGCCTCGCGGCTGATAATATCATTGATGCGCACTTAGTCAATGTTGATGGAAAAGTTTTAGATCGA
AAATCCATGGGGGAAGATTTGTTTTGGGCTATACGTGGTGGTGGAGGAGAAAACTTTGGAATCATTGCAGCGT
GGAAAATTAGACTTGTTGCTGTCCCATCAATGTCTACTATATTCAGTGTTAAAAAGAACATGGAGATACATGA
GCTTGTCAAGTTAGTTAACAAATGGCAAAATATTGCTTACATGTATGAAAAAGAATTATTACTCTTTACTCAC
TTTATAACCAGGAATATTACAGATAATCAAGGGAAGAATAAGACAACAATACACAGTTACTTCTCCTCCATTT
TCCATGGTGGAGTGGATAGTCTAGTCGACTTGATGAACAAGAGCTTTCCTGAATTGGGTATTAAAAAAACAGA
TTGCAAACAGTTGAGCTGGATTGATACTATCATCTTCTACAGTGGTGTTGTAAATTACAACACAACTAATTTT
AAAAAAGAAATTTTGCTTGATAGATCAGGTGGGCGGAAGGCGGCTTTCTCGATTAAGTTAGACTATGTTAAGA
AACCGATTCCAGAAACCGCAATGGTCACAATTTTGGAAAAATTATATGAAGAAGATGTAGGAGTTGGGATGTT
TGTGTTTTACCCTTATGGTGGTATAATGGATGAGATTTCAGAATCAGCAATTCCATTCCCTCATCGAGCTGGA
ATCATGTATGAAATTTGGTACATAGCTTCATGGGAGAAGCAAGAAGATAATGAAAAGCATATAAACTGGATTC
GGAATGTTTATAATTTCACGACTCCTTATGTGTCCCAAAATCCAAGAATGGCGTATCTCAATTATAGGGACCT
TGATTTAGGAAAAACTAATTTCGAGAGTCCTAATAATTACACACAAGCACGTATTTGGGGTGAAAAGTATTTT
GGTAAAAATTTTAATAGGTTAGTAAAAGTAAAAACCAAGGTTGATCCCGATAATTTCTTTAGAAACGAACAAA
GCATTCCACCTCTTCCCCTGCGTCATCATTAA
>C_Purple_Kush__AGQN03001397.1_570
ATGAAGTACTCAACATTCTGTTTTTGGTATGTTTGCAAGATAATATTTTTCTTTCTCTCATTCAATATCCAAA
TTTCAATAGCTAATCCTCAAGAAAACTTCCTTAAATGCCTCTCACAATATATTCCCACCAATGTAACAAATGC

AAAACTCGTATACACTCAACACGACCAATTTTATATGTCTATCTTAAATTCGACCATACAAAATCTTAGATTT
ACCTCTGACACAACCCCAAAACCACTTGTTATCATCACTCCTTTAAATGTCTCCCATATCCAAGGCACTATTC
TATGCTCCAAGAAATTTGGCTTGCAGATTCGAACTCGAAGCGGTGGTCATGATGCTGAGGGCATGTCCTACAT
ATCTCAAGTCCCATTTGTTATAGTAGACTTGAGAAACATGCATTCGGTCAAAATAGATGTTCATAGCCAAAAT
GCATGGGTTGAAGCCGGAGCTACCCTTGGAGAAGTTTATTATTGGATCAATGAGAACAATGAGAATCTTAGTT
TTCCTGCTGGGTACTGCCCTACTGTTGGCGCTTGTGGACACTTTAGTGGAGGAGGCTATGGAGCATTGATGCG
AAATTATGGCCTCGCGGCTGATAATATCATTGATGCACACTTAGTCAATGTTGATGGAAAAGTTTTAGATCGA
AAATCCATGGGGGAAGATTTGTTTTGGGCTATACGTGGTGGTGGAGGAGAAAACTTTGGAATCATTGCAGCGT
GGAAAATTAGACTTGTTGCTGTCCCATCAATGTCTACTATATTCAGTGTTAAAAAGAACATGGAGATACATGA
GCTTGTCAAGTTAGTTAACAAATGGCAAAATATTGCTTACATGTATGAAAAAGAATTATTACTCTTTACTCAC
TTTATAACCAGGAATATTACAGATAATCAAGGGAAGAATAAGACAACAATACACAGTTACTTCTCCTCCATTT
TCCATGGTGGAGTGGATAGTCTAGTCGACTTGATGAACAAGAGCTTTCCTGAATTGGGTATTAAAAAAAGAGA
TTGCAAACAGTTGAGCTGGATTGATACTATCATCTTCTACAGTGGTCTTGTAAATTACAACACAACTAATTTT
AAAAAAGAAATTTTGCTTGATAGATCAGGTGGGCGGAAGGCGGCTTTCTCGATTAAGTTAGACTATGTTAAGA
AACCGATTCCAGAAACCGCAATGGTCACAATTTTGGAAAAATTATATGAAGAAGATGTAGGAGTTGGGATGTT
TGTGTTTTACCCTTATGGTGGTATAATGGATGAGATTTCAGAATCAGCAATTCCATTCCATCGAGCTGGAATC
ATGTATGAAATTTGGTACATAGCTTCATGGGAGAAGCAAGAAGATAATGAAAAGCATATAAACTGGATTCGGA
ATGTTTATAATTTCACGACTCCTTATGTGTCCCAAAATCCAAGAATGGCGTATCTCAATTATAGGGACCTTGA
TTTAGGAAAAACTAATTTCGAGAGTCCTAATAATTACACACAAGCACGTATTTGGGGTGAAAAGTATTTTGGT
AAAAATTTTAATAGGTTAGTAAAAGTAAAAACCAAGGTTGATCCCGATAATTTCTTTAGAAACGAACAAAGCA
TCCCACCTCTTCCCCTGCGTCATCATTAA
>C_Jamaican_Lion_JAATIP010000026.1_F8388_024894
ATGAAGTACTCAACATTCTGTTTTTGGTATGTTTGCAAGATAATATTTTTCTTTCTCTCATTCAATATCCAAA
TTTCAATAGCTAATCCTCAAGAAAACTTCCTTAAATGCCTCTCACAATATATTCCCACCAATGTAACAAATGC
AAAACTCGTATACACTCAACACGACCAATTTTATATGTCTATCTTAAATTCGACCATACAAAATCTTAGATTT
ACCTCTGACACAACCCCAAAACCACTTGTTATCATCACTCCTTTAAATGTCTCCCATATCCAAGGCACTATTC
TATGCTCCAAGAAAGTTGGCTTGCAGATTCGAACTCGAAGCGGTGGTCATGATGCTGAGGGCATGTCCTACAT
ATCTCAAGTCCCATTTGTTATAGTAGACTTGAGAAACATGCATTCGGTCAAAATAGATGTTCATAGCCAAAAT
GCATGGGTTGAAGCCGGAGCTACCCTTGGAGAAGTTTATTATTGGATCAATGAGAACAATGAGAATCTTAGTT
TTCCTGCTGGGTACTGCCCTACTGTTGGCGCTGGTGGACACTTTAGTGGAGGAGGCTATGGAGCATTGATGCG
AAATTATGGCCTCGCGGCTGATAATATCATTGATGCACACTTAGTCAATGTTGATGGAAAAGTTTTAGATCGA
AAATCCATGGGGGAAGATTTGTTTTGGGCTATACGTGGTGGTGGAGGAGAAAACTTTGGAATCATTGCAGCGT
GGAAAATTAGACTTGTTGCTGTCCCATCAATGTCTACTATATTCAGTGTTAAAAAGAACATGGAGATACATGA
GCTTGTCAAGTTAGTTAACAAATGGCAAAATATTGCTTACATGTATGAAAAAGAATTATTACTCTTTACTCAC
TTTATAACCAGGAATATTACAGATAATCAAGGGAAGAATAAGACAACAATACACAGTTACTTCTCCTCCATTT
TCCATGGTGGAGTGGATAGTCTAGTCGACTTGATGAACAAGAGCTTTCCTGAATTGGGTATTAAAAAAAGAGA
TTGCAAACAGTTGAGCTGGATTGATACTATCATCTTCTACAGTGGTCTTGTAAATTACAACACAACTAATTTT
AAAAAAGAAATTTTGCTTGATAGATCAGGTGGGCGGAAGGCGGCTTTCTCGATTAAGTTAGACTATGTTAAGA
AACCGATTCCAGAAACCGCAATGGTCACAATTTTGGAAAAATTATATGAAGAAGATGTAGGAGTTGGGATGTT
TGTGTTTTACCCTTATGGTGGTATAATGGATGAGATTTCAGAATCAGCAATTCCATTCCCTCATCGAGCTGGA
ATCATGTATGAAATTTGGTACATAGCTTCATGGGAGAAGCAAGAAGATAATGAAAAGCATATAAACTGGATTC
GGAATGTTTATAATTTCACGACTCCTTATGTGTCCCAAAATCCAAGAATGGCGTATCTCAATTATAGGGACCT
TGATTTAGGAAAAACTAATTTCGAGAGTCCTAATAATTACACACAAGCACGTATTTGGGGTGAAAAGTATTTT
GGTAAAAATTTTAATAGGTTAGTAAAAGTAAAAACCAAGGTTGATCCCGATAATTTCTTTAGAAACGAACAAA
GCATCCCACCTCTTCCCCTGCGTCATCATTAA
>C_Jamaican_Lion_JAATIP010000103.1_F8388_004086
ATGAAGTACTCAACATTCTGTTTTTGGTATGTTTGCAAGATAATATTTTTCTTTCTCTCATTCAATATCCAAA
TTTCAATAGCTAATCCTCAAGAAAACTTCCTTAAATGCCTCTCACAATATATTCCCACCAATGTAACAAATGC
AAAACTCGTATACACTCAACACGACCAATTTTATATGTCTATCTTAAATTCGACCGTACAAAATCTTAGATTT
ACCTCTGACACAACCCCAAAACCACTTGTTATCACCACTCCTTTAAATGTCTCCCATATCCAAGGCACTATTC
TATGTTCCAAGAAAGTTGGCTTGCAGATTCGAACTCGAAGCGGTGGTCATGATGCTGAGGGCATGTCCTACAT
ATCTCAAGTCCCATTTGTTATAGTAGACTTGAGAAACATGCATTCGGTCAAAATAGATGTTCATAGCCAAACT
GCATGGGTTGAATCCGGAGCTACCCTTGGAGAAGTTTATTATTGGATCAATGAGAACAATGAGAATCTTAGTT
TTCCTGCTGGGTACTGCCCTACTGTTGGCACGGGTGGACACTTTAGTGGAGGAGGCTATGGAGCATTGATGCG
AAATTATGGCCTCGCGGCTGATAATATCATTGATGCGCACTTAGTCAATGTTGATGGAAAAGTTTTAGATCGA
AAATCCATGGGGGAAGATTTGTTTTGGGCTATACGTGGTGGTGGAGGAGAAAACTTTGGAATCATTGCAGCGT
GGAAAATTAGACTTGTTGCTGTCCCATCAATGTCTACTATATTCAGTGTTAAAAAGAACATGGAGATACATGA
GCTTGTCAAGTTAGTTAACAAATGGCAAAATATTGCTTACATGTATGAAAAAGAATTATTACTCTTTACTCAC

TTTATAACCAGGAATATTACAGATAATCAAGGGAAGAATAAGACAACAATACACAGTTACTTCTCCTCCATTT
TCCATGGTGGAGTGGATAGTCTAGTCGACTTGATGAACAAGAGCTTTCCTGAATTGGGTATTAAAAAAACAGA
TTGCAAACAGTTGAGCTGGATTGATACTATCATCTTCTACAGTGGTGTTGTAAATTACAACACAACTAATTTT
AAAAAAGAAATTTTGCTTGATAGATCAGGTGGGCGGAAGGCGGCTTTCTCGATTAAGTTAGACTATGTTAAGA
AACCGATTCCAGAAACCGCAATGGTCACAATTTTGGAAAAATTATATGAAGAAGATGTAGGAGTTGGGATGTT
TGTGTTTTACCCTTATGGTGGTATAATGGATGAGATTTCAGAATCAGCAATTCCATTCCCTCATCGAGCTGGA
ATCACGTATGAAATTTGGTACATAGCTTCATGGGAGAAGCAAGAAGATAATGAAAAGCATATAAACTGGATTC
GGAATGTTTATAATTTCACGACTCCTTATGTGTCCCAAAATCCAAGAATGGCGTATCTCAATTATAGGGACCT
TGATTTAGGAAAAACTAATTTCGAGAGTCCTAATAATTACACACAAGCACGTATTTGGGGTGAAAAGTATTTT
GGTAAAAATTTTAATAGGTTAGTAAAAGTAAAAACCAAGGTTGATCCCGATAATTTCTTTAGAAACGAACAAA
GCATCCCACCTCTTCCCCTGCGTCATCATTAA
>C_Jamaican_Lion_JAATIP010000026.1_F8388_024895
ATGAAGTACTCAACATTCTGTTTTTGGTATGTTTGCAAGATAATATTTTTCTTTCTCTCATTCAATATCCAAA
TTTCAATAGCTAATCCTCAAGAAAACTTCCTTAAATGCTTCTCACAATATATTCCCACCAATGTAACAAATGC
AAAACTCGTATACACTCAACACGACCAATTTTATATGTCTATCCTAAATTCGACCATACAAAATCTTAGATTT
ACCTCTGACACAACCCCAAAACCACTTGTTATCATCGCTCCTTTAAATGTCTCCCATATCCAAGGCACTATTC
TATGCTCCAAGAAAGTTGGCTTGCAGATTCGAGCTCGAAGCAGTGGTCATGATGCTGAGGGCATGTCCTACAT
ATCTCAAGTCCCATTTGTTATAGTAGACTTGAGAAACATGCATTCGGTCAAAATAGATGTTCATAGCCAAACT
GCATGGGTTGAAGCCGGAGCTACCCTTGGAGAAGTTTATTATTGGATCAATGAGAACAATGAGAATCTTAGTT
TTCCTGCTGGGTACTGTCCTACTGTTGGCGCGGGTGGACACTTTAGTGGAGGAGGCTATGGAGCATTGATGCG
AAATTATGGCCTCGCGGCTGATAATATCATTGATGCGCACTTAGTCAATGTTGATGGAAAAGTTTTAGATCGA
AAATCCATGGGGGAAGATTTGTTTTGGGCTATACGTGGTGGTGGAGGAGAAAACTCTGGAATCATTGCAGCGT
GGAAAATTAGACTTGTTGCTGTCCCATCAATGTCTACTATATTCAGTGTTAAAAAGAACATGGAGATACATGA
GCTTGTCAAGTTAGTTAAAAAATGGCAAAATATTGCTTACATGTATGAAAAAGAATTATTACTCTTTACTCAC
TTTATAACCAGGAATATTACAGATAATCAAGGGAAGAATAAGACAACAATACACTGTTACTTCTCCTCCATTT
TCCATGGTGGACTGGATAGTCTAGTCGACTTGATGAACAAGAGCTTTCCTGAATTGGGTATTAAAAAAACAGA
TTGCAAACAGTTGAGCTGGATTGATACTATCATCTTCAACAGTGGTCTTGTAAATTACAACACTACTAATTTT
AAAAAAGAAATTTTGCTTTAAAGATCAGGTGGGCGGAAGGCGGCTTTCTCAATTAAGTTAGACTATGTTAAGA
AACCGATTCCAGAAACCGCAATGGTCACAATTTTGGAAAAATTATATGAAGAAGATGTAGGAGTTGGGATGTT
CGTGTTTTACCCTTATGGTGGTATAATGGATGAGATTTCAGAATCAGCAATTCCATTCCCTCATCGAGCTGGA
ATCATGTATGAAATTTGGTACATAGCTTCATGGGAGAAGCAAGAAGATAATGAAAAGCATATAAACTGGATTC
GGAATGTTTATAATTTCACGACTCCTTATGTGTCCCAAAATCCAAGAATGGCGTATCTCAATTATAGGGACCT
TGATTTAGGAAAAACTAATTTCGAGAGTCCTAATAATTACACACAAGCACGTATTTGGGGTGAAAAGTATTTT
GGTAAAAATTTTAGTAGGTTAGTAAAAGTAAAAACCAAGGTTGATCCCGATAATTTCTTTAGAAACGAACAAA
GCATCCCACCTCTTCCCCTGCGTCATCATTAA
>C_Purple_Kush__CM010796.2_62089454
ATGAAGTACTCAACATTCTGTTTTTGGTATGTTTGCAAGATAATATTTTTCTTTCTCTCATTCAATATCCAAA
TTTCAATAGCTAATCCTCAAGAAAACTTCCTTAAATGCTTCTCACAATATATTCCCACCAATGTAACAAATGC
AAAACTCGTATACACTCAACACGACCAATTTTATATGTCTATCCTAAATTCGACCATACAAAATCTTAGATTT
ACCTCTGACACAACCCCAAAACCACTTGTTATCATCACTCCTTTAAATGTCTCCCATATCCAAGGCACTATTC
TATGCTCCAAGAAAGTTGGCTTGCAGATTCGAACTCGAAGCGGTGGTCATGATGCTGAGGGCATGTCCTACAT
ATCTCAAGTCCCATTTGTTATAGTAGACTTGAGAAACATGCATTCGGTCAAAATAGATGTTCATAGCCAAACT
GCATGGGTTGAAGCCGGAGCTACCCTTGGAGAAGTTTATTATTGGATCAATGAGAACAATGAGAATCTTAGTT
TTCCTGCTGGGTACTGCCCTACTGTTGGCGCGGGTGGACACTTTAGTGGAGGAGGCTATGGAGCATTGATGCG
AAATTATGGCCTCGCGGCTGATAATATCATTGATGCGCACTTAGTCAATGTTGATGGAAAAGTTTTAGATCGA
AAATCCATGGGGGAAGATTTGTTTTGGGCTATACGTGGTGGTGGAGGAGAAAACTTTGGAATCATTGCAGCGT
GGAAAATTAGACTTGTTGCTGTCCCATCAATGTCTACTATATTCAGTGTTAAAAAGAACATGGAGATACATGA
GCTTGTCAAGTTAGTTAACAAATGGCAAAATATTGCTTACATGTATGAAAAAGAATTATTACTCTTTACTCAC
TTTATAACCAGGAATATTACAGATAATCAAGGGAAGAATAAGACAACAATACACTGTTACTTCTCCTCCATTT
TCCATGGTGGACTGGATAGTCTAGTCGACTTGATGAACAAGAGCTTTCCTGAATTGGGTATTAAAAAAACAGA
TTGCAAACAGTTGAGCTGGATTGATACTATCATCTTCAACAGTGGTCTTGTAAATTACAACACTACTAATTTT
AAAAAAGAAATTTTGCTTTAAAGATCAGGTGGGCGGAAGGCGGCTTTCTCAATTAAGTTAGACTATGTTAAGA
AACCGATTCCAGAAACCGCAATGGTCACAATTTTGGAAAAATTATATGAAGAAGATGTAGGAGTTGGGATGTT
TGTGTTTTACCCTTATGGTGGTATAATGGATGAGATTTCAGAATCAGCAATTCCATTCCCTCATCGAGCTGGA
ATCATGTATGAAATTTGGTACATAGCTTCATGGGAGAAGCAAGAAGATAATGAAAAGCATATAAACTGGATTC
GGAATGTTTATAATTTCACGACTCCTTATGTGTCCCAAAATCCAAGAATGGCGTATCTCAATTATAGGGACCT
TGATTTAGGAAAAACTAATTTCGAGAGTCCTAATAATTACACACAAGCACGTATTTGGGGTGAAAAGTATTTT

GGTAAAAATTTTAATAGGTTAGTAAAAGTAAAAACCAAGGTTGATCCCGATAATTTCTTTAGAAACGAACAAA
GCATCCCACCTCTTCCCCTGCGTCATCATTAA
>C_Jamaican_Lion_JAATIP010000026.1_F8388_024892
ATGAAGTACTCAACATTCTGGTATGTTTGCAAGATAATATTTTTTTTTCTCTCATTCAATATCCAAATTTCAA
TAGCTAATCCTCAAGAAAACTTCCTTAAATGCTTCTCACAATATATTCCCACCAATGTAACAAATGCAAAACT
CGTATACACTCAACACGACCAATTTTATATGTCTATCCTAAATTCGACCATACAAAATCTTAGATTTACCTCT
GACACAACCCCAAAACCACTTGTTATCATCACTCCTTTAAATGTCTCCCATATCCAAGGCACTATTCTATGCT
CCAAAAAAGTTGGCTTGCAGATTCGAACTCGAAGCGGTGGTCATGATGCTGAGGGCATGTCCTACATATCTCA
AGTCCCATTTGTTATAGTAGACTTGAGAAACATGCATTCGGTCAAAATAGATGTTCATAGCCAAACTGCATGG
GTTGAAGCCGGAGCTACCCTTGGAGAAGTTTATTATTGGATCAATGAGAACAATGAGAATCTTAGTTTTCCTG
CTGGGTACTGCCCTACTGTTGGCGCGGGTGGACACTTTAGTGGAGGAGGCTATGGAGCATTGATGCGAAATTA
TGGCCTCGCGGCTGATAATATCATTGATGCGCACTTAGTCAATGTTGATGGAAAAGTTTTAGATCGAAAATCC
ATGGGGGAAGATTTGTTTTGGGCTATACGTGGTGGTGGAGGAGAAAACTTTGGAATCATTGTAGCGTGGAAAA
TTAGACTTGTTGCTGTCCCATCAATGTCTACTATATTCAGTGTTAAAAAGAACATGGAGATACATGAGCTTGT
CAAGTTAGTTAACAAATGGCAAAATATTGCTTACATGTATGAAAAAGAATTATTACTCTTTACTCACTTTATA
ACCAGGAATATTACAGATAATCAAGGGAAGAATAAGACAACAATACACAGTTACTTCTCCTCCATTTTCCATG
GTGGAGTGGATAGTCTAGTCGACTTGATGAACAAGAGCTTTCCTGAATTGGGTATTAAAAAAATAGATTGCAA
ACAGTTGAGCTGGATTGATACTATCATCTTCTACAGTGGTCTTGTAAATTACAACACAACTAATTTTAAAAAA
GAAATTTTGCTTGATAGATCAGGTGGGCGGAAGGCGGCTTTCTCGATTAAGTTAGACTATGTTAAGAAACCGA
TTCCAGAAACCGCAATGGTCACAATTTTGGAAAAATTATATGAAGAAGATGTAGGAGTTGGGATGTTTGTGTT
TTACCCTTATGGTGGTATAATGGATGAGATTTCAGAATCAGCAATTCCATTCCCTCATCGAGCTGGAATCATG
TATGAAATTTGGTACATAGCTTCATGGGAGAAGCAAGAAGATAATGAAAAGCATATAAACTGGATTCGGAATG
TTTATAATTTCACGACTCCTTATGTGTCCCAAAATCCAAGAATGGCGTATCTCAATTATAGGGACCTTGATTT
AGGAAAAACTAATTTTGAGAGACCTAATAATTACACACAAGCACGTATTTGGGGTGAAAAGTATTTTGGTAAA
AATTTTAATAGGTTAGTAAAAGTAAAAACCAAGGTTGATCCCGATAATTTCTTTAGAAACGAACAAAGCATCC
CACCTCTTCCCCTGCGTCATCATTAA
>C_Jamaican_Lion_JAATIP010000026.1_F8388_024891
ATGAAGTACTCAACATTCTGTTTTTGGTATGTTTGCAAGATAATATTTTTCTTTCTCTCATTCAATATCCAAA
TTTCAATAGCTAATCCTGAAGGAAACTTCCTTAAATGCTTCTCACAATATATTCCCACCAATGTAACAAATGC
AAAACTCGTATACACTCAACACGACCAATTTTATATGTCTATCCTAAATTCGACCATACAAAATCTTAGATTT
ACCTTTGACACAACCCCAAAACCACTTGTTATCATCACTCCTTTAAATGTCTCCCATATCCAAGGCACTATTC
TATGCTCCAAGAAAGTTGGCTTGTAGATTCGAACTCGAAGCGGTGGTCATGATGCTGAGGGCATGTCCTACAT
ATCTCAAGTCCCATTTGTTATAGTAAACTTGAGAAACATGCATTCGGTCAAAATAGATGTTCATAGCGAAACT
GCATGGGTTGAAGCCGGAGCTACCCTTGGAGAAGTTTATTATTGGATCAATGAGAACAATGAGAATCTTAGTT
TTCTTGCTGGGTACTGCCCTACTGTTGGCGCGGGTGGACACTTTAGTGGAGGAGGCTATGGAGCATTGATGCG
AAATTATGGCCTCGCGGCTAATAACATCATTGATGCGCACTTAGTCAATGTTGATGGAAAAGTTTTAGATCGA
AAATCCATGGGGGAAGATTTGTTTTGGGCTATACGTGGTGGAGGAGAAAACTTTGGAATCATTGCAGCGTGGA
AAATTAGACTTGTTGCTGTCCCATCAATGTCTACTATATTCAGTGTTAAAAAGAACATGGAGATACATGAGCT
TGTCAAGTTAGTTAACAAATGACAAAATATTGCTTACATGTATGAAAAAGAATGATTACTCTTTACTCACTTT
ATAACCAGGAATATTACAGATAATCAAGGGAAGAATAAGACAACAATACACAGTTACTTCTCCTCCATTTTCT
ATGGTGGAGTGGATAGTCTAGTCGACTTGATGAACAAGAGCTTTCCTGAATTGGGTATTAAAAAAACAGATTG
CAAACAGTTGAGCTGGATTGATACTATCATCTTCTACAGTGGTCTTGTAAATTACAACACTACTAATTTTAAA
AAAGAACTTTTGCTTGATAGATCAGGTGGGCGGAAGGCGGCTTTCTCGATTAAGTTAGACTAAGTTAAGAAAC
CGATTCCAGAAACCGCAATGGTCACAATTTTGGAAAAATTATATGAAGAAGATGTAGGAGTTGGGATGTTTGT
GTTTTACCCTTATGGTGGTATAATGGATGAGATTTCAGAATCAGCAATTCCATTCCCTCATCGAGCTGGAATC
ATGTATGAAATTTGGTACATAGCTTCATGGGAGAAGCAAGAAGATAATGAAAAGCATATAAACTGGATTCGGA
ATGTTTACAATTTCACGACTCCTTATGTGTCCCAAAATCCAAGAATGGCGTATCTCAATTATAGGGACCTTGA
TTTAGGAAAAACTAATTTCGAGAGTCCTAATAATTACACACAAGCACGTATTTGGGGTGAAAAGTATTTTGGT
AAAAATTTTAATAGGTTAGTAAAAGTAAAAACCAAGGTTGATCCCGATAATTTCTTTAGAAACGAACAAAGCA
TCCCACCTCTTCCCCTACGTCATCATTAA
>C_Purple_Kush__AGQN03001397.1_88111
ATGAAGTACTCAACATTCTGTTTTTGGTATGTTTGCAAGATAATATTTTTCTTTCTCTCATTCAATATCCAAA
TTTCAATAGCTAATCCTGAAGGAAACTTCCTTAAATGCTTCTCACAATATATTCCCACCAATGTAACAAATGC
AAAACTCGTATACACTCAACACGACCAATTTTATATGTCTATCCTAAATTCGACCATACAAAATCTTAGATTT
ACCTTTGACACAACCCCAAAACCACTTGTTATCATCACTCCTTTAAATGTCTCCCATATCCAAGGCACTATTC
TATGCTCCAAGAAAGTTGGCTTGTAGATTCGAACTCGAAGCGGTGGTCATGATGCTGAGGGCATGTCCTACAT
ATCTCAAGTCCCATTTGTTATAGTAAACTTGAGAAACATGCATTCGGTCAAAATAGATGTTCATAGCGAAACT
GCATGGGTTGAAGCCGGAGCTACCCTTGGAGAAGTTTATTATTGGATCAATGAGAACAATGAGAATCTTAGTT

TTCTTGCTGGGTACTGCCCTACTGTTGGCGCGGGTGGACACTTTAGTGGAGGAGGCTATGGAGCATTGATGCG
AAATTATGGCCTCGCGGCTAATAACATCATTGATGCGCACTTAGTCAATGTTGATGGAAAAGTTTTAGATCGA
AAATCCATGGGGGAAGATTTGTTTTGGGCTATACGTGGTGGAGGAGAAAACTTTGGAATCATTGCAGCGTGGA
AAATTAGATTTGTTGCTGTCCCATCAATGTCTACTATATTCAGTGTTAAAAAGAACATGGAGATACATGAGCT
TGTCAAGTTAGTTAACAAATGGCAAAATATTGCTTACATGTATGAAAAAGAATGATTACTCTTTACTCACTTT
ATAACCAGGAATATTACAGATAATCAAGGGAAGAATAAGACAACAATACACAGTTACTTCTCCTCCATTTTCT
ATGGTGGAGTGGATAGTCTAGTCGACTTGATGAACAAGAGCTTTCCTGAATTGGGTATTAAAAAAACAGATTG
CAAACAGTTGAGCTGGATTGATACTATCATCTTCTACAGTGGTCTTGTAAATTACAACACTACTAATTTTAAA
AAAGAACTTTTGCTTGATAGATCAGGTGGGCGGAAGGCGGCTTTCTCGATTAAGTTAGACTAAGTTAAGAAAC
CGATTCCAGAAACCGCAATGGTCACAATTTTGGAAAAATTATATGAAGAAGATGTAGGAGTTGGGATGTTTGT
GTTTTACCCTTATGGTGGTATAATGGATGAGATTTCAGAATCAGCAATTCCATTCCCTCATCGAGCTGGAATC
ATGTATGAAATTTGGTACATAGCTTCATGGGAGAAGCAAGAAGATAATGAAAAGCATATAAACTGGATTCGGA
ATGTTTACAATTTCACGACTCCTTATGTGTCCCAAAATCCAAGAATGGCGTATCTCAATTATAGGGACCTTGA
TTTAGGAAAAACTAATTTCGAGAGTCCTAATAATTACACACAAGCACGTATTTGGGGTGAAAAGTATTTTGGT
AAAAATTTTAATAGGTTAGTAAAAGTAAAAACCAAGGTTGATCCCGATAATTTCTTTAGAAACGAACAAAGCA
TCCCACCTCTTCCCCTACGTCATCATTAA
>C_CBDRx_NC_044378.1_LOC115696966
ATGAAGTACTCAACATTCTGTTTTTGGTATGTTTGCAAGATAATATTTTTCTTTCTCTCATTCAATATCCAAA
TTTCAATAGCTAATCCTCAAGAAAACTTCCTTAAATGCTTCTCACAATATATTCCCACCAATGTAACAAATGC
AAAACTCGTATACACTCAACACGACCAATTTTATATGTCTATCCTGAATTCGACCATACAAAATCTTAGATTT
ACCTCTGACACAACCCCAAAACCACTTGTTATCATCACTCCTTTAAATGTCTCCCATATCCAAGGCACTATTC
TATGCTCCAAGAAAGTTGGCTTGCAGATTCGAACTCGAAGCGGTGGTCATGATGCTGAGGGCATGTCCTACAT
ATCTCAAGTCCCATTTGTTATAGTAGACTTGAGAAACATGCATTCGGTCAAAATAGATGTTCATAGCCAAACC
GCATGGGTTGAAGCCTGAGCTACCCTTGGAGAAGTTTATTATTGGATCAATGAGAACAATGAGAATCTTAGTT
TTCCATGGTACTGCCTCTTCAAGTTGGCTGGTGGACACTTTAGTGGAGGAGGCTATGGAGCATTGATGCGAAA
TTATGGCCTCGCGGCTGATAATATCATTGATGCGCACTTAGTCAATGTTGATGGAAAAGTTTTAGATCGAAAA
TCCATGGGGGAAGATTTGTTTTGGGCTATACGTGGTGGTGGAGGAGAAAACTTTGGAATCATTGCAGCGTGGA
AAATTAGACTTGTTGCTGTCCCATCAATGTCTACTATATTCAGTGTTAAAAAGAACATGGAGATACATGAGCT
TGTCAAGTTAGTTAACAAATGGCAAAATATTGCTTACATGTATGAAAAAGAATTATTACTCTTTACTCACTTT
ATAACCGTGAATATTACGAATCAAGGGAAGAATAAGACAACAATACACGCTTACTTCTCCTCCATTTTCCATG
GTGGAGTGGATAGTCTAGTCGACTTGATGAACAAGAGCTTTCCTGAATTGGGTATTAAAAAAACAGATTGCAA
ACAGTTGAGCTGGATTGATACTATCATCTTCTACAGTGGTGTTGTAAATTACAACACAACTAATTTTAAAAAA
GAAATTTTGCTTGATAGATCAGGTGGGCGGAAGGCGGCTTTCTCGATTAAGTTAGACTATGTTAAGAAACCGA
TTCCAGAAACCGCAATGGTCACAATTTTGGAAAAATTATATGAAGAAGATGTAGGAGTTGGGATGTTTGTGTT
TTACCCTTATGGTGGTATAATGGATGAGATTTCAGAATCAGCAATTCCATTCCCTCATCGAGCTGGAATCATG
TATGAAATTTGGTACATAGCTTCATGGGAGAAGCAAGAAGATAATGAAAAGCATATAAACTGGATTCGGAATG
TTTATAATTTCACGACTCCTTATGTGTCCCAAAATCCAAGAATGGCGTATCTCAATTATAGGGACCTTGATTT
AGGAAAAACTAATTTCGAGAGTCCTAATAATTACACACAAGCACGTATTTGGGGTGAAAAGTATTTTGGTAAA
AATTTTAATAGGTTAGTAAAAGTAAAAACCAAGGTTGATCCCGATAATTTCTTTAGAAACGAACAAAGCATTC
CACCTCTTCCCCTGCGTCATCATTAA
>C_Purple_Kush__AGQN03001397.1_36865
ATGAAGTACTCAACATTCTGGTATGTTTGCAAGATAATATTTTTTTTTCTCTCATTCAATATCCAAATTTCAA
TAGCTAATCCTCAAGAAAACTTCCTTAAATGCTTCTCACAATATATTCCCACCAATGTAACAAATGCAAAACT
CGTATACACTCAACACGACCAATTTTATATGTCTATCCTAAATTCGACCATACAAAATCTTAGATTTACCTCT
GACACAACCAAACCACTTGTTATCATCACTCCTTTAAATGTCTCCCATATCCAAGGCACTATTCTATGCTCCA
AAAAAGTTGGCTTGCAGATTCGAACTCGAAGCGGTGGTCATGATGCTGAGGGCATGTCCTACATATCTCAAGT
CCCATTTGTTATAGTAGACTTGAGAAACATGCATTCGGTCAAAATAGATGTTCATAGCCAAACTGCATGGGTT
GAAGCCGGAGCTACCCTTGGAGAAGTTTATTATTGGATCAATGAGAACAATGAGAATCTTAGTTTTCCTGCTG
GGTACTGCCCTACTGTTGGCGCGGGACACTTTAGTGGAGGAGGCTATGGAGCATTGATGCGAAATTATGGCCT
CGCGGCTGATAATATCATTGATGCGCACTTAGTCAATGTTGATGGAAAAGTTTTAGATCGAAAATCCATGGGG
GATTTGTTTTGGGCTATACGTGGTGGTGGAGGAGAAAACTTTGGAATCATTGCGTGGAAAATTAGACTTGTTG
CTGTCCCATCAATGTCTACTATATTGTTAAAAAAGAACATGGAGATACATGAGCTTGTCAAGTTAGTTAACAA
ATGGCAAAATATTGCTTACATGTATGAAAAAGAATTATTACTCTTTACTCACTTTATAACCAGGAATATTACA
GATAATCAAGGGAAGAATAAGACAACAATACACAGTTACTTCTCCTCCATTTTCCATGGTGGAGTGGATAGTC
TAGTCGACTTGATGAACAAGAGCTTTCCTGAATTGGGTATTAAAAAAGATTGCAAACAGTTGAGCTGGATTGA
TACTATCATCTTCTACAGTGGTCTTGTAAATTACAACACAACTAATTTTAAAAAAGAAATTTTGCTTGATAGA
TCAGGTGGGCGGAAGGCGGCTTTCTCGATTAAGTTAGACTATGTTAAGAAACCGATTCCAGAAACCGCAATGG
TCACAATTTTGGAAAAATTATATGAAGAAGATGTAGGAGTTGGGATGTTTGTGTTTTACCCTTATGGTGGTAT

AATGGATGAGATTTCAGAATCAGCAATTCCATTCCCTCATCGAGCTGGAATCATGTATGAAATTTGGTACATA
GCTTCATGGGAGAAGCAAGAAGATAATGAAAAGCATATAAACTGGATTCGGAATGTTTATAATTTCACGACTC
CTTATGTGTCCCAAAATCCAAGAATGGCGTATCTCAATTATAGGGACCTTGATTTAGGAAAAACTAATTTCGA
GAGTCCTAATAATTACACACAAGCACGTATTTGGGGTGAAAAGTATTTTGGTAAAAATTTTAATAGGTTAGTA
AAAGTAAAAACCAAGGTTGATCCCGATAATTTCTTTAGAAACGAACAAAGCATCCCACCTCTTCCCCTGCGTC
ATCATTAA
>C_CBDRx_NC_044378.1_LOC115697083
ATGAAGTACTCAACATTCTGTTTTTGGTATGTTTGCAAGATAATATTTTTCTTTCTCTCATTCAATATCCAAA
TTTCAATAGCTAATCCTCAAGAAAACTTCCTTAAATGCCTCTCACAATATATTCCCACCAATGTAACAAATGC
AAAACTCGTATACACTCAACACGACCAATTTTATATGTCTATCTTAAATTCGACCGTACAAAATCTTAGATTT
ACCTCTGACACAACCCCAAAACCACTTGTTATCACCACTCCTTTAAATGTCTCCCATATCCAAGGCACTATTC
TATGTTCCAAGAAAGTTGGCTTGCAGATTCGAACTCGAAGCGGTGGTCATGATGCTGAGGGCATGTCCTACAT
ATCTCAAGTCCCATTTGTTATAGTAGACTTGAGAAACATGCATTCGGTCAAAATAGATGTTCATAGCCAAACT
GCATGGGTTGAATCCGGAGCTACCCTTGGAGAAGTTTATTATTGGATCAATGAGAACAATGAGAATCTTAGTT
TTCCTGCTGGGTACTGCCCTACTGTTGGCACGGGTGGACACTTTAGTGGAGGAGGCTATGGAGCATTGATGCG
AAATTATGGCCTCGCGGCTGATAATATCATTGATGCGCACTTAGTCAATGTTGATGGAAAAGTTTTAGATCGA
AAATCCATGGGGGAAGATTTGTTTTGGGCTATACGTGGTGGTGGAGGAGAAAACTTTGGAATCATTGCAGCGT
GGAAAATTAGACTTGTTGCTGTCCCATCAATGTCTACTATATTCAGTGTTAAAAAGAACATGGAGATACATGA
GCTTGTCAAGTTAGTTAACAAATGGCAAAATATTGCTTACATGTATGAAGAATTATTATTTACTCACTTTATA
ACCAAAAATACTACAGATAATCAAGGAAAGAATAAAACAACAGTACACGGTTACTTCCCTTCCATTTTCCATG
GTGGAGCGGATAGTTTGGTTGACTTGATGAACAAGAGCTTTCCTGAATTGGGTATTAAAAAAGATTGCAAATT
GAGTATTGATACTATCATCTTTTACGCTGGTGTTGTAAATTACAACACAACTAATTTTAAAAAAGAAATTTTG
CTTGATAGATCAGGTGGGCGGAAGGCGGCTTTCTCGATTAAGTTAGACTATGTTAAGAAACCGATTCCAGAAA
CCGCAATGGTCACAATTTTGGAAAAATTATATGAAGAAGATGTAGGAGTTGGGATGTTTGTGTTTTACCCTTA
TGGTGGTATAATGGATGAGATTTCAGAATCAGCAATTCCATTCCCTCATCGAGCTGGAATCACGTATGAAATT
TGGTACATAGCTTCATGGGAGAAGCAAGAAGATAATGAAAAGCATATAAACTGGATTCGGAATGTTTATAATT
TCACGACTCCTTATGTGTCCCAAAATCCAAGAATGGCGTATCTCAATTATAGGGACCTTGATTTAGGAAAAAC
TAATTTCGAGAGTCCTAATAATTACACACAAGCACGTATTTGGGGTGAAAAGTAATTTGGTAAAAATTTTAAT
AGGTTAGTAAAAGTAAAAACCAAGGTTGATCCCGATAATTTCTTTAGAAACGAACAAAGCATCCCACCTCTTC
CCCTGCGTCATCATTAA
>C_CBDRx_NC_044378.1_LOC115696476
ATGAAGTACTCAACATTCTGTTTTTGGTATGTTTGCAAGATAATATTTTTCTTTCTCTCATTCAATATCCAAA
TTTCAATAGCTAATCCTCAAGAAAACTTCCTTAAATGCTTCTCACAATATATTCCCACCAATGTAACAAATGC
AAAACTCGTATACACTCAACACGACCAATTTTATATGTCTATCCTAAATTCGACCATACAAAATCTTAGATTT
ACCTCTGAAACAACCCCAAAACCACTTGTTATCATCACTCCTTTAAATGTCTCCCATATCCAAGGCACTATTC
TATGCTCCAAGAAAGTTGGCCTGATTCGAACTTCAGGCGGTGGTCATGATCTTGAGGGCATGTCCTACATATC
TGAAGTCCCATTTGTTATAGTAGACTTGAGAAACATGCATTAGGTCAAAATAGATGTTCATAGCCAAACCGCA
TGGGTTGAAGCCAGAGCTACCCTTGGAGAAGTTTATTATTGGATCAATGAGAACAATGAGAATCTTAGTTTTG
TTGGATCTTGCCCTCTTGTTGCTGGTGGACACTTTAGTGGAGGAGGCTATGGAGCATTGGAAAATTATGGCCT
CATGGATAATATCATTGATCACTTAGTCAATGTTGATGGAAAAGTTTTAGATCGAAAATCCATGGGGGATTTG
TTTTGGGCTATCCGTGGTGGTGGAGGAGAAAACTTTGGAATCATTGCGGCGTGGAAAATTAGACTTGTTCTTG
TCCCATCAATGTCTACTATATTCAGTGTTAAAAAGAACATGGAGATACATGAGCTTGTCAAGTTAGTTAACAA
ATGGCAAAATATTGCTTACATGTATGAAAAAGAATTATTACTCTTTACTCACTTTATAACCAGGAATATTACA
GATAATCAAGGGAAGAATAAGACAACAATACACAGTTACTTCTCCTCCATTTTCCATGGTGGAGTGGATAGTC
TAGTCGACTTGATGAACAAGAGCTTTCCTGAATTGGGTATTAAAAAAAAAGATTGCAAACAGTTGAGCTGGAT
TGATACTATCATCTTCTACAGTGGTGTTGTAAATTACAACACAACTAATTTTAAAAAAGAAATTTTGCTTGAT
AGATCAGGTGGGCGGAAGGCGGCTTTCTCGATTAAGTTAGACTATGTTAAGAAACCGATTCCAGAAACCGCAA
TGGTCACAATTTTGGAAAAATTATATGAAGAAGATGTAGGAGTTGGGATGTTTGTGTTTTACCCTTATGGTGG
TATAATGGATGAGATTTCAGAATCAGCAATTCCATTCCCTCATCGAGCTGGAATCACGTATGAAATTTGGTAC
ATAGCTTCATGGGAGAAGCAAGAAGATAATGAAAAGCATATAAACTGGATTCGGAATGTTTATAATTTCACGA
CTCCTTATGTGTCCCAAAATCCAAGAATGGCGTATCTCAATTATAGGGACCTTGATTTAGGAAAAACTAATTT
CGAGAGTCCTAATAATTACACACAAGCAAATATTTGGGGTGAAAAGTATTTTGGTAAAAATTTTAATAGGTTA
GTAAAAGTAAAAAAGGTTGATGATAATTTCTTTAGAAGCGAACAAAGCATCCCACCTCTTCCCCGTCATCATT
AA
>C_AB292683.1
ATGAAGTGCTCAACATTCTGTTTTTGGTATGTTTGCAAGATAATATTTTTCTTTCTCTCATTCAATATCC
AAATTTCAATAGCTAATCCTCAAGAAAACTTCCTTAAATGCTTCTCACAATATATTCCCACCAATGTAAC
AAATGCAAAACTCGTATACACTCAACACGACCAATTTTATATGTCTATCCTGAATTCGACCATACAAAAT

```
CTTAGATTTACCTCTGACACAACCCCAAAACCACTTGTTATCATCACTCCTTTAAATGTCTCCCATATCC
AAGGCACTATTCTATGCTCCAAGAAAGTTGGCTTGCAGATTCGAACTCGAAGCGGTGGTCATGATGCTGA
GGGCATGTCCTACATATCTCAAGTCCCATTTGTTATAGTAGACTTGAGAAACATGCATTCGGTCAAAATA
GATGTTCATAGCCAAACTGCATGGGTTGAAGCCGGAGCTACCCTTGGAGAAGTTTATTATTGGATCAATG
AGAACAATGAGAATCTTAGTTTTCCTGCTGGGTACTGCCCTACTGTTGGCGCGGGTGGACACTTTAGTGG
AGGAGGCTATGGAGCATTGATGCGAAATTATGGCCTCGCGGCTGATAATATCATTGATGCGCACTTAGTC
AATGTTGATGGAAAAGTTTTAGATCGAAAATCCATGGGGGAAGATTTGTTTTGGGCTATACGTGGTGGTG
GAGGAGAAAACTTTGGAATCATTGCAGCGTGGAAAATTAGACTTGTTGCTGTCCCATCAATGTCTACTAT
ATTCAGTGTTAAAAAGAACATGGAGATACATGAGCTTGTCAAGTTAGTTAACAAATGGCAAAATATTGCT
TACATGTATGAAAAAGAATTATTACTCTTTACTCACTTTATAACCAGGAATATTACAGATAATCAAGGGA
AGAATAAGACAACAATACACAGTTACTTCTCCTCCATTTTCCATGGTGGAGTGGATAGTCTAGTCGACTT
GATGAACAAGAGCTTTCCTGAATTGGGTATTAAAAAAACAGATTGCAAACAGTTGAGCTGGATTGATACT
ATCATCTTCTACAGTGGTGTTGTAAATTACAACACAACTTATTTTAAAAAAGAAATTTTGCTTGATAGAT
CAGGTGGGCGGAAGGCGGCTTTCTCGATTAAGTTAGACTATGTTAAGAAACCGATTCCAGAAACCGCAAT
GGTCACAATTTTGGAAAAATTATATGAAGAAGATGTAGGAGTTGGGATGTTTGTGTTTTACCCTTATGGT
GGTATAATGGATGAGATTTCAGAATCAGCAATTCCATTCCCTCATCGAGCTGGAATCATGTATGAAATTT
GGTACATAGCTTCATGGGAGAAGCAAGAAGATAATGAAAAGCATATAAACTGGATTCGGAATGTTTATAA
TTTCACGACTCCTTATGTGTCCCAAAATCCAAGAATGGCGTATCTCAATTATAGGGACCTTGATTTAGGA
AAAACTAATTTCGAGAGTCCTAATAATTACACACAAGCACGTATTTGGGGTGAAAAGTATTTTGGTAAAA
ATTTTAATAGGTTAGTAAAAGTAAAAACCAAGGTTGATCCCGATAATTTCTTTAGAAACGAACAAAGCAT
CCCACCTCTTCCCCTGCGTCATCATTAA
>C_KJ469382.1
CATGCATTCGGTCAAAATAGATGTTCATAGCCAAACTGCATGGGTTGAAGCCGGAGCTACCCTTGGAGAAGTT
TATTATTGGATCAATGAGAACAATGAGAATCTTAGTTTTCCTGCTGGGTACTGCCCTACTGTTGGCGCGGGTG
GACACTTTAGTGGAGGAGGCTATGGAGCATTGATGCGAAATTATGGCCTCGCGGCTGATAATATCATTGATGC
GCACTTAGTCAATGTTGATGGAAAAGTTTTAGATCGAAAATCCATGGGGGAAGATTTGTTTTGGGCTATACGT
GGTGGTGGAGGAGAAAACTTTGGAATCATTGCAGCGTGGAAAATTAGACTTGTTGCTGTCCCATCAATGTCTA
CTATATTCAGTGTTAAAAAGAACATGGAGATACATGAGCTTGTCAAGTTAGTTAACAAATGGCAAAATATTGC
TTACATGTATGAAAAAGAATTATTACTCTTTACTCACTTTATAACCAGGAATATTACAGATAATCAAGGGAAG
AATAAGACAACAATACACAGTTACTTCTCCTCCATTTTCCATGGTGGAGTGGATAGTCTAGTCGACTTGATGA
ACAAGAGCTTTCCTGAATTGGGTATTAAAAAAACAGATTGCAAACAGTTGAGCTGGATTGATACTATCATCTT
CTACAGTGGTCTTGTAAATTACAACACAACTAATTTTAAAAAAGAAATTTTGCTTGATAGATCAGGTGGGCGG
AAGGCGGCTTTCTCGATTAAGTTAGACTATGTTAAGAAACCGATTCCAGAAACCGCAATGGTCACAATTTTGG
AAAAATTATATGAAGAAGATGTAGGAGTTGGGATGTTTGTG
>C_KJ469383.1
CATGCATTCGGTCAAAATAGATGTTCATAGCCAAACTGCATGGGTTGAAGCCGGAGCTACCCTTGGAGAAGTT
TATTATTGGATCAATGAGAACAATGAGAATCTTAGTTTTCCTGCTGGGTACTGCCCTACTGTTGGCGCGGGTG
GACACTTTAGTGGAGGAGGCTATGGAGCATTGATGCGAAATTATGGCCTCGCGGCTGATAATATCATTGATGC
GCACTTAGTCAATGTTGATGGAAAAGTTTTAGATCGAAAATCCATGGGGGAAGATTTGTTTTGGGCTATACGT
GGTGGTGGAGGAGAAAACTTTGGAATCATTGCAGCGTGGAAAATTAGACTTGTTGCTGTCCCATCAATGTCTA
CTATATTCAGTGTTAAAAAGAACATGGAGATACATGAGCTTGTCAAGTTAGTTAACAAATGGCAAAATATTGC
TTACATGTATGAAAAAGAATTATTACTCTTTACTCACTTTATAACCAGGAATATTACAGATAATCAAGGGAAG
AATAAGACAACAATACACAGTTACTTCTCCTCCATTTTCCATGGTGGAGTGGATAGTCTAGTCGACTTGATGA
ACAAGAGCTTTCCTGAATTGGGTATTAAAAAAACAGATTGCAAACAGTTGAGCTGGATTGATACTATCATCTT
CTACAGTGGTCTTGTAAATTACAACACAACTAATTTTAAAAAAGAAATTTTGCTTGATAGATCAGGTGGGCGG
AAGGCGGCTTTCTCGATTAAGTTAGACTATGTTAAGAAACCGATTCCAGAAACCGCAATGGTCACAATTTTGG
AAAAATTATATGAAGAAGATGTAGGAGTTGGGATGTTTGTG
>C_AB292684.1
ATGAAGTGCTCAACATTCTGTTTTTGGTATGTTTGCAAGATAATATTTTTCTTTCTCTCATTCAATATCC
AAATTTCAATAGCTAATCCTCAAGAAAACTTCCTTAAATGCCTCTCACAATATATTCCCACCAATGTAAC
AAATGCAAAACTCGTATACACTCAACACGACCAATTTTATATGTCTATCTTAAATTCGACCGTACAAAAT
CTTAGATTTACCTCTGACACAACCCCAAAACCACTTGTTATCACCACTCCTTTAAATGTCTCCCATATCC
AAGGCACTATTCTATGTTCCAAGAAAGTTGGCTTGCAGATTCGAACTCGAAGCGGTGGTCATGATGCTGA
GGGCATGTCCTACATATCTCAAGTCCCATTTGTTATAGTAGACTTGAGAAACATGCATTCGGTCAAAATA
GATGTTCATAGCCAAACTGCATGGGTTGAATCCGGAGCTACCCTTGGAGAAGTTTATTATTGGATCAATG
AGAACAATGAGAATCTTAGTTTTCCTGCTGGGTACTGCCCTACTGTTGGCACGGGTGGACACTTTAGTGG
AGGAGGCTATGGAGCATTGATGCGAAATTATGGCCTCGCGGCTGATAATATCATTGATGCGCACTTAGTC
AATGTTGATGGAAAAGTTTTAGATCGAAAATCCATGGGGGAAGATTTGTTTTGGGCTATACGTGGTGGTG
```

GAGGAGAAAACTTTGGAATCATTGCAGCGTGGAAAATTAGACTTGTTGCTGTCCCATCAATGTCTACTAT
ATTCAGTGTTAAAAAGAACATGGAGATACATGAGCTTGTCAAGTTAGTTAACAAATGGCAAAATATTGCT
TACATGTATGAAAAAGAATTATTACTCTTTACTCACTTTATAACCAGGAATATTACAGATAATCAAGGGA
AGAATAAGACAACAATACACAGTTACTTCTCCTCCATTTTCCATGGTGGAGTGGATAGTCTAGTCGACTT
GATGAACAAGAGCTTTCCTGAATTGGGTATTAAAAAAACAGATTGCAAACAGTTGAGCTGGATTGATACT
ATCATCTTCTACAGTGGTGTTGTAAATTACAACACAACTAATTTTAAAAAAGAAATTTTGCTTGATAGAT
CAGGTGGGCGGAAGGCGGCTTTCTCGATTAAGTTAGACTATGTTAAGAAACCGATTCCAGAAACCGCAAT
GGTCACAATTTTGGAAAAATTATATGAAGAAGATGTAGGAGTTGGGATGTTTGTGTTTTACCCTTATGGT
GGTATAATGGATGAGATTTCAGAATCAGCAATTCCATTCCCTCATCGAGCTGGAATCACGTATGAAATTT
GGTACATAGCTTCATGGGAGAAGCAAGAAGATAATGAAAAGCATATAAACTGGATTCGGAATGTTTATAA
TTTCACGACTCCTTATGTGTCCCAAAATCCAAGAATGGCGTATCTCAATTATAGGGACCTTGATTTAGGA
AAAACTAATTTCGAGAGTCCTAATAATTACACACAAGCACGTATTTGGGGTGAAAAGTATTTTGGTAAAA
ATTTTAATAGGTTAGTAAAAGTAAAAACCAAGGTTGATCCCGATAATTTCTTTAGAAACGAACAAAGCAT
CCCACCTCTTCCCCTGCGTCATCATTAA

>C_Cannatonic_MNPR01004530_417
ATGAAGTACTCAACATTCTTTTGGTATGTTTGCAAGATAATATTTTTCTTTCTCTCATTCAATATCCAAATTT
CAATAGCTAATCCTCAAGAAAACTTCCTTAAATGCTTCTCACAATATATTCCCACCAATGTAACAAATGCAAA
ACTCGTATACACTCAACACGACCAATTTTATATGTCTATCCTGAATTCGACCATACAAAATCTTAGATTTACC
TCTGACACAACCCCAAAACCACTTGTTATCATCACTCCTTTAAATGTCTCCCATATCCAAGGCACTATTCTAT
GCTCCAAGAAAGTTGGCTTGCAGATTCGAACTCGAAGCGGTGGTCATGATGCTGAGGGCATGTCCTACATATC
TCAAGTCCCATTTGTTATAGTAGACTTGAGAAACATGCATTCGGTCAAAATAGATGTTCATAGCCAAACTGCA
TGGGTTGAAGCCGGAGCTACCCTTGGAGAAGTTTATTATTGGATCAATGAGAACAATGAGAATCTTAGTTTTC
CTGCTGGGTACTGCCCTACTGTTGGCGCGGGTGGACACTTTAGTGGAGGAGGCTATGGAGCATTGATGCGAAA
TTATGGCCTCGCGGCTGATAATATCATTGATGCGCACTTAGTCAATGTTGATGGAAAAGTTTTAGATCGAAAA
TCCGGGGAAGATTTGTTTTGGGCTATACGTGGTGGTGGAGGAGAAAACTTTGGAATCATTGCAGCGTGGAAAA
TTAGACTTGTTGCTGTCCCATCAATGTCTACTATATTCAGTGTTAAAAAGAACATGGAGATACATGAGCTTGT
CAAGTTAGTTAACAAATGGCAAAATATTGCTTACATGTATGAAAAAGAATTATTACTCTTTACTCACTTTATA
ACCAGGAATATTACAGATAATCAAGGGAAGAATAAGACAACAATACACAGTTACTTCTCCTCCATTTTCCATG
GTGGAGTGGATAGTCTAGTCGACTTGATGAACAAGAGCTTTCCTGAATTGGGTATTAAAAAAACAGATTGCAA
ACAGTTGAGCTGGATTGATACTATCATCTTCTACAGTGGTGTTGTAAATTACAACACAACTAATTTTAAAAAA
GAAATTTTGCTTGATAGATCAGGTGGGCGGAAGGCGGCTTTCTCGATTAAGTTAGACTATGTTAAGAAACCGA
TTCCAGAAACCGCAATGGTCACAATTTTGGAAAAATTATATGAAGAAGATGTAGGAGTTGGGATGTTTGTGTT
TTACCCTTATGGTGGTATAATGGATGAGATTTCAGAATCAGCAATTCCATTCCCTCATCGAGCTGGAATCATG
TATGAAATTTGGTACATAGCTTCATGGGAGAAGCAAGAAGATAATGAAAAGCATATAAACTGGATTCGGAATG
TTTATAATTTCACGACTCCTTATGTGTCCCAAAATCCAAGAATGGCGTATCTCAATTATAGGGACCTTGATTT
AGGAAAAACTAATTTCGAGAGTCCTAATAATTACACACAAGCACGTATTTGGGGTGAAAAGTATTTTGGTAAA
AATTTTAATAGGTTAGTAAAAGTAAAAACCAAGGTTGATCCCGATAATTTCTTTAGAAACGAACAAAGCATTC
CACCTCTTCCCCTGCGTCATCATTAA
>C_JLion_father_JAATIQ010000127.1_G4B88_028761
ATGAAGTACTCAACATTCTGTTTTTGGTATGTTTGCAAGATAATATTTTTCTTTCTCTCATTCAATATCCAAA
TTTCAATAGCTAATCCTCAAGAAAACTTCCTTAAATGCTTCTCACAATATATTCCCACCAATGTAACAAATGC
AAAACTCGTATACACTCAACACGACCAATTTTATATGTCTATCCTGAATTCGACCATACAAAATCTTAGATTT
ACCTCTGACACAACCCCAAAACCACTTGTTATCATCACTCCTTTAAATGTCTCCCATATCCAAGGCACTATTC
TATGCTCCAAGAAAGTTGGCTTGCAGATTCGAACTCGAAGCGGTGGTCATGATGCTGAGGGCATGTCCTACAT
ATCTCAAGTCCCATTTGTTATAGTAGACTTGAGAAACATGCATTCGGTCAAAATAGATGTTCATAGCCAAACT
GCATGGGTTGAAGCCGGAGCTACCCTTGGAGAAGTTTATTATTGGATCAATGAGAACAATGAGAATCTTAGTT
TTCCTGCTGGGTACTGCCCTACTGTTGGCGCGGGTGGACACTTTAGTGGAGGAGGCTATGGAGCATTGATGCG
AAATTATGGCCTCGCGGCTGATAATATCATTGATGCGCACTTAGTCAATGTTGATGGAAAAGTTTTAGATCGA
AAATCCATGGGGGAAGATTTGTTTTGGGCTATACGTGGTGGTGGAGGAGAAAACTTTGGAATCATTGCAGCGT
GGAAAATTAGACTTGTTGCTGTCCCATCAATGTCTACTATATTCAGTGTTAAAAAGAACATGGAGATACATGA
GCTTGTCAAGTTAGTTAACAAATGGCAAAATATTGCTTACATGTATGAAAAAGAATTATTACTCTTTACTCAC
TTTATAACCAGGAATATTACAGATAATCAAGGGAAGAATAAGACAACAATACACAGTTACTTCTCCTCCATTT
TCCATGGTGGAGTGGATAGTCTAGTCGACTTGATGAACAAGAGCTTTCCTGAATTGGGTATTAAAAAAACAGA
TTGCAAACAGTTGAGCTGGATTGATACTATCATCTTCTACAGTGGTGTTGTAAATTACAACACAACTAATTTT
AAAAAAGAAATTTTGCTTGATAGATCAGGTGGGCGGAAGGCGGCTTTCTCGATTAAGTTAGACTATGTTAAGA
AACCGATTCCAGAAACCGCAATGGTCACAATTTTGGAAAAATTATATGAAGAAGATGTAGGAGTTGGGATGTT
TGTGTTTTACCCTTATGGTGGTATAATGGATGAGATTTCAGAATCAGCAATTCCATTCCCTCATCGAGCTGGA

ATCATGTATGAAATTTGGTACATAGCTTCATGGGAGAAGCAAGAAGATAATGAAAAGCATATAAACTGGATTC
GGAATGTTTATAATTTCACGACTCCTTATGTGTCCCAAAATCCAAGAATGGCGTATCTCAATTATAGGGACCT
TGATTTAGGAAAAACTAATTTCGAGAGTCCTAATAATTACACACAAGCACGTATTTGGGGTGAAAAGTATTTT
GGTAAAAATTTTAATAGGTTAGTAAAAGTAAAAACCAAGGTTGATCCCGATAATTTCTTTAGAAACGAACAAA
GCATTCCACCTCTTCCCCTGCGTCATCATTAA
>C_LAconfidential_LKUA01081076.1_754
ATGAAGTACTCAACATTCTGTTTTTGGTATGTTTGCAAGATAATATTTTTCTTTCTCTCATTCAATATCCAAA
TTTCAATAGCTAATCCTCAAGAAAACTTCCTTAAATGCTTCTCACAATATATTCCCACCAATGTAACAAATGC
AAAACTCGTATACACTCAACACGACCAATTTTATATGTCTATCCTAAATTCGACCATACAAAATCTTAGATTT
ACCTCTGACACAACCCCAAAACCACTTGTTATCATCACTCCTTTAAATGTCTCCCATATCCAAGGCACTATTC
TATGCTCCAAGAAAGTTGGCTTGCAGATTCGAACTCGAAGCGGTGGTCATGATGCTGAGGGCATGTCCTACAT
ATCTCAAGTCCCATTTGTTATAGTAGACTTGAGAAACATGCATTCGGTCAAAATAGATGTTCATAGCCAAACT
GCATGGGTTGAAGCCGGAGCTACCCTTGGAGAAGTTTATTATTGGATCAATGAGAACAATGAGAATCTTAGTT
TTCCTGCTGGGTACTGCCCTACTGTTGGCGCGGGTGGACACTTTAGTGGAGGAGGCTATGGAGCATTGATGCG
AAATTATGGCCTCGCGGCTGATAATATCATTGATGCGCACTTAGTCAATGTTGATGGAAAAGTTTTAGATCGA
AAATCCATGGGGGAAGATTTGTTTTGGGCTATACGTGGTGGTGGAGGAGAAAACTTTGGAATCATTGCAGCGT
GGAAAATTAGACTTGTTGCTGTCCCATCAATGTCTACTATATTCAGTGTTAAAAAGAACATGGAGATACATGA
GCTTGTCAAGTTAGTTAACAAATGGCAAAATATTGCTTACATGTATGAAAAAGAATTATTACTCTTTACTCAC
TTTATAACCAGGAATATTACAGATAATCAAGGGAAGAATAAGACAACAATACACAGTTACTTCTCCTCCATTT
TCCATGGTGGAGTGGATAGTCTAGTCGACTTGATGAACAAGAGCTTTCCTGAATTGGGTATTAAAAAAACAGA
TTGCAAACAGTTGAGCTGGATTGATACTATCATCTTCTACAGTGGTCTTGTAAATTACAACACTACTAATTTT
AAAAAAGAAATTTTGCTTGATAGATCAGGTGGGCGGAAGGCGGCTTTCTCGATTAAGTTAGACTATGTTAAGA
AACCGATTCCAGAAACCGCAATGGTCACAATTTTGGAAAAATTATATGAAGAAGATGTAGGAGTTGGGATGTT
TGTGTTTTACCCTTATGGTGGTATAATGGATGAGATTTCAGAATCAGCAATTCCATTCCCTCATCGAGCTGGA
ATCATGTATGAAATTTGGTACATAGCTTCATGGGAGAAGCAAGAAGATAATGAAAAGCATATAAACTGGATTC
GGAATGTTTATAATTTCACGACTCCTTATGTGTCCCAAAATCCAAGAATGGCGTATCTCAATTATAGGGACCT
TGATTTAGGAAAAACTAATTTCGAGAGTCCTAATAATTACACACAAGCACGTATTTGGGGTGAAAAGTATTTT
GGTAAAAATTTTAATAGGTTAGTAAAAGTAAAAACCAAGGTTGATCCC
>C_PBBK_MXBD01004333.1_20309
ATGAAGTACTCAACATTCTGTTTTTGGTATGTTTGCAAGATAATATTTTTCTTTCTCTCATTCAATATCCAAA
TTTCAATAGCTAATCCTCAAGAAAACTTCCTTAAATGCTTCTCACAATATATTCCCACCAATGTAACAAATGC
AAAACTCGTATACACTCAACACGACCAATTTTATATGTCTATCCTAAATTCGACCATACAAAATCTTAGATTT
ACCTCTGACACAACCCCAAAACCACTTGTTATCATCACTCCTTTAAATGTCTCCCATATCCAAGGCACTATTC
TATGCTCCAAGAAAGTTGGCTTGCAGATTCGAACTCGAAGCGGTGGTCATGATGCTGAGGGCATGTCCTACAT
ATCTCAAGTCCCATTTGTTATAGTAGACTTGAGAAACATGCATTCGGTCAAAATAGATGTTCATAGCCAAACT
GCATGGGTTGAAGCCGGAGCTACCCTTGGAGAAGTTTATTATTGGATCAATGAGAACAATGAGAATCTTAGTT
TTCCTGCTGGGTACTGCCCTACTGTTGGCGCGGGTGGACACTTTAGTGGAGGAGGCTATGGAGCATTGATGCG
AAATTATGGCCTCGCGGCTGATAATATCATTGATGCGCACTTAGTCAATGTTGATGGAAAAGTTTTAGATCGA
AAATCCATGGGGGAAGATTTGTTTTGGGCTATACGTGGTGGTGGAGGAGAAAACTTTGGAATCATTGCAGCGT
GGAAAATTAGACTTGTTGCTGTCCCATCAATGTCTACTATATTCAGTGTTAAAAAGAACATGGAGATACATGA
GCTTGTCAAGTTAGTTAACAAATGGCAAAATATTGCTTACATGTATGAAAAAGAATTATTACTCTTTACTCAC
TTTATAACCAGGAATATTACAGATAATCAAGGGAAGAATAAGACAACAATACACAGTTACTTCTCCTCCATTT
TCCATGGTGGAGTGGATAGTCTAGTCGACTTGATGAACAAGAGCTTTCCTGAATTGGGTATTAAAAAAACAGA
TTGCAAACAGTTGAGCTGGATTGATACTATCATCTTCTACAGTGGTCTTGTAAATTACAACACTACTAATTTT
AAAAAAGAAATTTTGCTTGATAGATCAGGTGGGCGGAAGGCGGCTTTCTCGATTAAGTTAGACTATGTTAAGA
AACCGATTCCAGAAACCGCAATGGTCACAATTTTGGAAAAATTATATGAAGAAGATGTAGGAGTTGGGATGTT
TGTGTTTTACCCTTATGGTGGTATAATGGATGAGATTTCAGAATCAGCAATTCCATTCCCTCATCGAGCTGGA
ATCATGTATGAAATTTGGTACATAGCTTCATGGGAGAAGCAAGAAGATAATGAAAAGCATATAAACTGGATTC
GGAATGTTTATAATTTCACGACTCCTTATGTGTCCCAAAATCCAAGAATGGCGTATCTCAATTATAGGGACCT
TGATTTAGGAAAAACTAATTTCGAGAGTCCTAATAATTACACACAAGCACGTATTTGGGGTGAAAAGTATTTT
GGTAAAAATTTTAATAGGTTAGTAAAAGTAAAAACCAAGGTTGATCCCGATAATTTCTTTAGAAACGAACAAA
GCATCCCACCTCTTCCCCTGCGTCATCATTAA
>C_PBBK_MXBD01006583.1_24636
ATGAAGTACTCAACATTCTGTTTTTGGTATGTTTGCAAGATAATATTTTTCTTTCTCTCATTCAATATCCAAA
TTTCAATAGCTAATCCTCAAGAAAACTTCCTTAAATGCTTCTCACAATATATTCCCACCAATGTAACAAATGC
AAAACTCGTATACACTCAACACGACCAATTTTATATGTCTATCCTAAATTCGACCATACAAAATCTTAGATTT
ACCTCTGACACAACCCCAAAACCACTTGTTATCATCACTCCTTTAAATGTCTCCCATATCCAAGGCACTATTC
TATGCTCCAAGAAAGTTGGCTTGCAGATTCGAACTCGAAGCGGTGGTCATGATGCTGAGGGCATGTCCTACAT

ATCTCAAGTCCCATTTGTTATAGTAGACTTGAGAAACATGCATTCGGTCAAAATAGATGTTCATAGCCAAACT
GCATGGGTTGAAGCCGGAGCTACCCTTGGAGAAGTTTATTATTGGATCAATGAGAACAATGAGAATCTTAGTT
TTCCTGCTGGGTACTGCCCTACTGTTGGCGCGGGTGGACACTTTAGTGGAGGAGGCTATGGAGCATTGATGCG
AAATTATGGCCTCGCGGCTGATAATATCATTGATGCGCACTTAGTCAATGTTGATGGAAAAGTTTTAGATCGA
AAATCCATGGGGGAAGATTTGTTTTGGGCTATACGTGGTGGTGGAGGAGAAAACTTTGGAATCATTGCAGCGT
GGAAAATTAGACTTGTTGCTGTCCCATCAATGTCTACTATATTCAGTGTTAAAAAGAACATGGAGATACATGA
GCTTGTCAAGTTAGTTAACAAATGGCAAAATATTGCTTACATGTATGAAAAAGAATTATTACTCTTTACTCAC
TTTATAACCAGGAATATTACAGATAATCAAGGGAAGAATAAGACAACAATACACAGTTACTTCTCCTCCATTT
TCCATGGTGGAGTGGATAGTCTAGTCGACTTGATGAACAAGAGCTTTCCTGAATTGGGTATTAAAAAAACAGA
TTGCAAACAGTTGAGCTGGATTGATACTATCATCTTCTACAGTGGTCTTGTAAATTACAACACAACTAATTTT
AAAAAAGAAATTTTGCTTGATAGATCAGGTGGGCGGAAGGCGGCTTTCTCGATTAAGTTAGACTATGTTAAGA
AACCGATTCCAGAAACCGCAATGGTCACAATTTTGGAAAAATTATATGAAGAAGATGTAGGAGTTGGGATGTT
TGTGTTTTACCCTTATGGTGGTATAATGGATGAGATTTCAGAATCAGCAATTCCATTCCCTCATCGAGCTGGA
ATCATGTATGAAATTTGGTACATAGCTTCATGGGAGAAGCAAGAAGATAATGAAAAGCATATAAACTGGATTC
GGAATGTTTATAATTTCACGACTCCTTATGTGTCCCAAAATCCAAGAATGGCGTATCTCAATTATAGGGACCT
TGATTTAGGAAAAACTAATTTCGAGAGTCCTAATAATTACACACAAGCACGTATTTGGGGTGAAAAGTATTTT
GGTAAAAATTTTAATAGGTTAGTAAAAGTAAAAACCAAGGTTGATCCCGATAATTTCTTTAGAAACGAACAAA
GCATCCCACCTCTTCCCCTACGTCATCATTAA
>C_JLion_father_JAATIQ010000127.1_G4B88_028760
ATGAAGTACTCAACATTCTGTTTTTGGTATGTTTGCAAGATAATATTTTTCTTTCTCTCATTCAATATCCAAA
TTTCAATAGCTAATCCTCAAGAAAACTTCCTTAAATGCTTCTCACAATATATTCCCACCAATGTAACAAATGC
AAAACTCGTATACACTCAACACGACCAATTTTATATGTCTATCCTAAATTCGACCATACAAAATCTTAGATTT
ACCTCTGAAACAACCCCAAAACCACTTGTTATCATCACTCCTTTAAATGTCTCCCATATCCAAGGCACTATTC
TATGCTCCAAGAAAGTTGGCTTGCAGATTCGAACTCGAAGCGGTGGTCATGATGCTGAGGGCATGTCCTACAT
ATCTGAAGTCCCATTTGTTATAGTAGACTTGAGAAACATGCATTCGGTCAAAATAGATGTTCATAGCCAAACT
GCATGGGTTGAAGCCGGAGCTACCCTTGGAGAAGTTTATTATTGGATCAATGAGAACAATGAGAATCTTAGTT
TTCCTGCTGGGTACTGCCCTACTGTTGGCGCGGGTGGACACTTTAGTGGAGGAGGCTATGGAGCATTGATGCG
AAATTATGGCCTCGCGGCTGATAATATCATTGATGCGCACTTAGTCAATGTTGATGGAAAAGTTTTAGATCGA
AAATCCATGGGGGAAGATTTGTTTTGGGCTATACGTGGTGGTGGAGGAGAAAACTTTGGAATCATTGCAGCGT
GGAAAATTAGACTTGTTGCTGTCCCATCAATGTCTACTATATTCAGTGTTAAAAAGAACATGGAGATACATGA
GCTTGTCAAGTTAGTTAACAAATGGCAAAATATTGCTTACATGTATGAAAAAGAATTATTACTCTTTACTCAC
TTTATAACCAGGAATATTACAGATAATCAAGGGAAGAATAAGACAACAATACACAGTTACTTCTCCTCCATTT
TCCATGGTGGAGTGGATAGTCTAGTCGACTTGATGAACAAGAGCTTTGCTGAATTGGGTATTAAAAAAACAGA
TTGCAAACAGTTGAGCTGGATTGATACTATCATCTTCTACAGTGGTGTTGTAAATTACAACACAACTAATTTT
AAAAAAGAAATTTTGCTTGATAGATCAGGTGGGCGGAAGGCGGCTTTCTCGATTAAGTTAGACTATGTTAAGA
AACCGATTCCAGAAACCGCAATGGTCACAATTTTGGAAAAATTATATGAAGAAGATGTAGGAGTTGGGATGTT
TGTGTTTTACCCTTATGGTGGTATAATGGATGAGATTTCAGAATCAGCAATTCCATTCCCTCATCGAGCTGGA
ATCATGTATGAAATTTGGTACATAGCTTCATGGGAGAAGCAAGAAGATAATGAAAAGCATATAAACTGGATTC
GGAATGTTTATAATTTCACGACTCCTTATGTGTCCCAAAATCCAAGAATGGCGTATCTCAATTATAGGGACCT
TGATTTAGGAAAAACTAATTTCGAGAGTCCTAATAATTACACACAAGCACGTATTTGGGGTGAAAAGTATTTT
GGTAAAAATTTTAATAGGTTAGTAAAAGTAAAAACCAAGGTTGATCCCGATAATTTCTTTAGAAACGAACAAA
GCATCCCACCTCTTCCCCTACGTCATCATTAA
>C_JLion_father_JAATIQ010000127.1_G4B88_028759
ATGAAGTACTCAACATTCTGTTTTTGGTATGTTTGCAAGATAATATTTTTCTTTCTCTCATTCAATATCCAAA
TTTCAATAGCTAATCCTCAAGAAAACTTCCTTAAATGCTTCTCACAATATATTCCCACCAATGTAACAAATGC
AAAACTCGTATACACTCAACACGACCAATTTTATATGTCTATCCTAAATTCGACCATACAAAATCTTAGATTT
ACCTCTGAAACAACCCCAAAACCACTTGTTATCATCACTCCTTTAAATGTCTCCCATATCCAAGGCACTATTC
TATGCTCCAAGAAAGTTGGCTTGCAGATTCGAACTCGAAGCGGTGGTCATGATGCTGAGGGCATGTCCTACAT
ATCTGAAGTCCCATTTGTTATAGTAGACTTGAGAAACATGCATTCGGTCAAAATAGATGTTCATAGCCAAACT
GCATGGGTTGAAGCCGGAGCTACCCTTGGAGAAGTTTATTATTGGATCAATGAGAACAATGAGAATCTTAGTT
TTCCTGCTGGGTACTGCCCTACTGTTGGCGCGGGTGGACACTTTAGTGGAGGAGGCTATGGAGCATTGATGCG
AAATTATGGCCTCGCGGCTGATAATATCATTGATGCGCACTTAGTCAATGTTGATGGAAAAGTTTTAGATCGA
AAATCCATGGGGGAAGATTTGTTTTGGGCTATACGTGGTGGTGGAGGAGAAAACTTTGGAATCATTGCAGCGT
GGAAAATTAGACTTGTTGCTGTCCCATCAATGTCTACTATATTCAGTGTTAAAAAGAACATGGAGATACATGA
GCTTGTCAAGTTAGTTAACAAATGGCAAAATATTGCTTACATGTATGAAAAAGAATTATTACTCTTTACTCAC
TTTATAACCAGGAATATTACAGATAATCAAGGGAAGAATAAGACAACAATACACAGTTACTTCTCCTCCATTT
TCCATGGTGGAGTGGATAGTCTAGTCGACTTGATGAACAAGAGCTTTCCTGAATTGGGTATTAAAAAAACAGA
TTGCAAACAGTTGAGCTGGATTGATACTATCATCTTCTACAGTGGTGTTGTAAATTACAACACAACTAATTTT

AAAAAAGAAATTTTGCTTGATAGATCAGGTGGGCGGAAGGCGGCTTTCTCGATTAAGTTAGACTATGTTAAGA
AACCGATTCCAGAAACCGCAATGGTCACAATTTTGGAAAAATTATATGAAGAAGATGTAGGAGTTGGGATGTT
TGTGTTTTACCCTTATGGTGGTATAATGGATGAGATTTCAGAATCAGCAATTCCATTCCCTCATCGAGCTGGA
ATCATGTATGAAATTTGGTACATAGCTTCATGGGAGAAGCAAGAAGATAATGAAAAGCATATAAACTGGATTC
GGAATGTTTATAATTTCACGACTCCTTATGTGTCCCAAAATCCAAGAATGGCGTATCTCAATTATAGGGACCT
TGATTTAGGAAAAACTAATTTCGAGAGTCCTAATAATTACACACAAGCACGTATTTGGGGTGAAAAGTATTTT
GGTAAAAATTTTAATAGGTTAGTAAAAGTAAAAACCAAGGTTGATCCCGATAATTTCTTTAGAAACGAACAAA
GCATCCCACCTCTTCCCCTACGTCATCATTAA
>C_PBBK_MXBD01005126.1_15361
ATGAAGTACTCAACATTCTGTTTTTGGTATGTTTGCAAGATAATATTTTTCTTTCTCTCATTCAATATCCAAA
TTTCAATAGCTAATCCTCAAGAAAACTTCCTTAAATGCTTCTCACAATATATTCCCACCAATGTAACAAATGC
AAAACTCGTATACAGTCAACACGACCAATTTTATATGTCTATCCTAAATTCGACCATACAAAATCTTAGATTT
ACCTCTGAAACAACCCCAAAACCACTTGTTATCATCACTCCTTTAAATGTCTCCCATATCCAAGGCACTATTC
TATGCTCCAAGAAAGTTGGCTTGCAGATTCGAACTCGAAGCGGTGGTCATGATGCTGAGGGCATGTCCTACAT
ATCTGAAGTCCCATTTGTTATAGTAGACTTGAGAAACATGCATTCGGTCAAAATAGATGTTCATAGCCAAACT
GCATGGGTTGAAGCCGGAGCTACCCTTGGAGAAGTTTATTATTGGATCAATGAGAACAATGAGAATCTTAGTT
TTCCTGCTGGGTACTGCCCTACTGTTGGCGCGGGTGGACACTTTAGTGGAGGAGGCTATGGAGCATTGATGCG
AAATTATGGCCTCGCGGCTGATAATATCATTGATGCGCACTTAGTCAATGTTGATGGAAAAGTTTTAGATCGA
AAATCCATGGGGGAAGATTTGTTTTGGGCTATACGTGGTGGTGGAGGAGAAAACTTTGGAATCATTGCAGCGT
GGAAAATTAGACTTGTTGCTGTCCCATCAATGTCTACTATATTCAGTGTTAAAAAGAACATGGAGATACATGA
GCTTGTCAAGTTAGTTAACAAATGGCAAAATATTGCTTACATGTATGAAAAAGAATTATTACTCTTTACTCAC
TTTATAACCAGGAATATTACAGATAATCAAGGGAAGAATAAGACAACAATACACAGTTACTTCTCCTCCATTT
TCCATGGTGGAGTGGATAGTCTAGTCGACTTGATGAACAAGAGCTTTCCTGAATTGGGTATTAAAAAAACAGA
TTGCAAACAGTTGAGCTGGATTGATACTATCATCTTCTACAGTGGTGTTGTAAATTACAACACAACTAATTTT
AAAAAAGAAATTTTGCTTGATAGATCAGGTGGGCGGAAGGCGGCTTTCTCGATTAAGTTAGACTATGTTAAGA
AACCGATTCCAGAAACCGCAATGGTCACAATTTTGGAAAAATTATATGAAGAAGATGTAGGAGTTGGGATGTT
TGTGTTTTACCCTTATGGTGGTATAATGGATGAGATTTCAGAATCAGCAATTCCATTCCCTCATCGAGCTGGA
ATCATGTATGAAATTTGGTACATAGCTTCATGGGAGAAGCAAGAAGATAATGAAAAGCATATAAACTGGATTC
GGAATGTTTATAATTTCACGACTCCTTATGTGTCCCAAAATCCAAGAATGGCGTATCTCAATTATAGGGACCT
TGATTTAGGAAAAACTAATTTCGAGAGTCCTAATAATTACACACAAGCACGTATTTGGGGTGAAAAGTATTTT
GGTAAAAATTTTAATAGGTTAGTAAAAGTAAAAACCAAGGTTGATCCCGATAATTTCTTTAGAAACGAACAAA
GCATCCCACCTCTTCCCCTACGTCATCATTAA
>C_Cannatonic_MNPR01004356_13281
ATGAAGTACTCAACATTCTGTTTTTGGTATGTTTGCAAGATAATATTTTTCTTTCTCTCATTCAATATCCAAA
TTTCAATAGCTAATCCTCAAGAAAACTTCCTTAAATGCTTCTCACAATATATTCCCACCAATGTAACAAATGC
AAAACTCGTATACACTCAACACGACCAATTTTATATGTCTATCCTAAATTCGACCATACAAAATCTTAGATTT
ACCTCTACAACCCCAAAACCACTTGTTATCATCACTCCTTTAAATGTCTCCCATATCCAAGGCACTATTCTAT
GCTCCAAGAAAGTTGGCTTGCAGATTCGAACTCGAAGCGGTGGTCATGATGCTGAGGGCATGTCCTACATATC
TGAAGTCCCATTTGTTATAGTAGACTTGAGAAACATGCATTCGGTCAAAATAGATGTTCATAGCCAAACTGCA
TGGGTTGAAGCCGGAGCTACCCTTGGAGAAGTTTATTATTGGATCAATGAGAACAATGAGAATCTTAGTTTTC
CTGCTGGGTACTGCCCTACTGTTGGCGCGGGTGGACACTTTAGTGGAGGAGGCTATGGAGCATTGATGCGAAA
TTATGGCCTCGCGGCTGATAATATCATTGATGCGCACTTAGTCAATGTTGATGGAAAAGTTTTAGATCGAAAA
TCCATGGGGGAAGATTTGTTTTGGGCTATACGTGGTGGTGGAGGAGAAAACTTTGGAATCATTGCAGCGTGGA
AAATTAGACTTGTTGCTGTCCCATCAATGTCTACTATATTCAGTGTTAAAAAGAACATGGAGATACATGAGCT
TGTCAAGTTAGTTAACAAATGGCAAAATATTGCTTACATGTATGAAAAAGAATTATTACTCTTTACTCACTTT
ATAACCAGGAATATTACAGATAATCAAGGGAAGAATAAGACAACAATACACAGTTACTTCTCCTCCATTTTCC
ATGGTGGAGTGGATAGTCTAGTCGACTTGATGAACAAGAGCTTTCCTGAATTGGGTATTAAAAAAACAGATTG
CAAACAGTTGAGCTGGATTGATACTATCATCTTCTACAGTGGTGTTGTAAATTACAACACAACTAATTTTAAA
AAAGAAATTTTGCTTGATAGATCAGGTGGGCGGAAGGCGGCTTTCTCGATTAAGTTAGACTATGTTAAGAAAC
CGATTCCAGAAACCGCAATGGTCACAATTTTGGAAAAATTATATGAAGAAGATGTAGGAGTTGGGATGTTTGT
GTTTTACCCTTATGGTGGTATAATGGATGAGATTTCAGAATCAGCAATTCCATTCCCTCATCGAGCTGGAATC
ATGTATGAAATTTGGTACATAGCTTCATGGGAGAAGCAAGAAGATAATGAAAAGCATATAAACTGGATTCGGA
ATGTTTATAATTTCACGACTCCTTATGTGTCCCAAAATCCAAGAATGGCGTATCTCAATTATAGGGACCTTGA
TTTAGGAAAAACTAATTTCGAGAGTCCTAATAATTACACACAAGCACGTATTTGGGGTGAAAAGTATTTTGGT
AAAAATTTTAATAGGTTAGTAAAAGTAAAAACCAAGGTTGATCCCGATAATTTCTTTAGAAACGAACAAAGCA
TCCCACCTCTTCCCCTACGTCATCATTAA
>C_Cannatonic_MNPR01008261_9370

ATGAAGTACTCAACATTCTGTTTTTGGTATGTTTGCAAGATAATATTTTTCTTTCTCTCATTCAATATCCAAA
TTTCAATAGCTAATCCTCAAGAAAACTTCCTTAAATGCTTCTCACAATATATTCCCACCAATGTAACAAATGC
AAAACTCGTATACACTCAACACGACCAATTTTATATGTCTATCCTAAATTCGACCATACAAAATCTTAGATTT
ACCTCTACAACCCCAAAACCACTTGTTATCATCACTCCTTTAAATGTCTCCCATATCCAAGGCACTATTCTAT
GCTCCAAGAAAGTTGGCTTGCAGATTCGAACTCGAAGCGGTGGTCATGATGCTGAGGGCATGTCCTACATATC
TGAAGTCCCATTTGTTATAGTAGACTTGAGAAACATGCATTCGGTCAAAATAGATGTTCATAGCCAAACTGCA
TGGGTTGAAGCCGGAGCTACCCTTGGAGAAGTTTATTATTGGATCAATGAGAACAATGAGAATCTTAGTTTTC
CTGCTGGGTACTGCCCTACTGTTGGCGCGGGTGGACACTTTAGTGGAGGAGGCTATGGAGCATTGATGCGAAA
TTATGGCCTCGCGGCTGATAATATCATTGATGCGCACTTAGTCAATGTTGATGGAAAAGTTTTAGATCGAAAA
TCCATGGGGGAAGATTTGTTTTGGGCTATACGTGGTGGTGGAGGAGAAAACTTTGGAATCATTGCAGCGTGGA
AAATTAGACTTGTTGCTGTCCCATCAATGTCTACTATATTCAGTGTTAAAAAGAACATGGAGATACATGAGCT
TGTCAAGTTAGTTAACAAATGGCAAAATATTGCTTACATGTATGAAAAAGAATTATTACTCTTTACTCACTTT
ATAACCAGGAATATTACAGATAATCAAGGGAAGAATAAGACAACAATACACAGTTACTTCTCCTCCATTTTCC
ATGGTGGAGTGGATAGTCTAGTCGACTTGATGAACAAGAGCTTTCCTGAATTGGGTATTAAAAAAACAGATTG
CAAACAGTTGAGCTGGATTGATACTATCATCTTCTACAGTGGTGTTGTAAATTACAACACAACTAATTTTAAA
AAAGAAATTTTGCTTGATAGATCAGGTGGGCGGAAGGCGGCTTTCTCGATTAAGTTAGACTATGTTAAGAAAC
CGATTCCAGAAACCGCAATGGTCACAATTTTGGAAAAATTATATGAAGAAGATGTAGGAGTTGGGATGTTTGT
GTTTTACCCTTATGGTGGTATAATGGATGAGATTTCAGAATCAGCAATTCCATTCCCTCATCGAGCTGGAATC
ATGTATGAAATTTGGTACATAGCTTCATGGGAGAAGCAAGAAGATAATGAAAAGCATATAAACTGGATTCGGA
ATGTTTATAATTTCACGACTCCTTATGTGTCCCAAAATCCAAGAATGGCGTATCTCAATTATAGGGACCTTGA
TTTAGGAAAAACTAATTTCGAGAGTCCTAATAATTACACACAAGCACGTATTGGTGAAAAGTATTTTGGTAAA
AATTTTAATAGGTTAGTAAAAGTAAAAACCAAGGTTGATCCCGATAATTTCTTTAGAAACGAACAAAGCATCC
CACCTCTTCCCCTACGTCATCATTAA
>C_JLion_father_JAATIQ010000127.1_G4B88_028762
ATGAAGTACTCAACATTCTGTTTTTGGTATGTTTGCAAGATAATATTTTTCTTTCTCTCATTCAATATCCAAA
TTTCAATAGCTAATCCTCAAGAAAACTTCCTTAAATGCCTCTCACAATATATTCCCACCAATGTAACAAATGC
AAAACTCGTATACACTCAACACGACCAATTTTATATGTCTATCTTAAATTCGACCGTACAAAATCTTAGATTT
ACCTCTGACACAACCCCAAAACCACTTGTTATCACCACTCCTTTAAATGTCTCCCATATCCAAGGCACTATTC
TATGTTCCAAGAAAGTTGGCTTGCAGATTCGAACTCGAAGCGGTGGTCATGATGCTGAGGGCATGTCCTACAT
ATCTCAAGTCCCATTTGTTATAGTAGACTTGAGAAACATGCATTCGGTCAAAATAGATGTTCATAGCCAAACT
GCATGGGTTGAATCCGGAGCTACCCTTGGAGAAGTTTATTATTGGATCAATGAGAACAATGAGAATCTTAGTT
TTCCTGCTGGGTACTGCCCTACTGTTGGCACGGGTGGACACTTTAGTGGAGGAGGCTATGGAGCATTGATGCG
AAATTATGGCCTCGCGGCTGATAATATCATTGATGCGCACTTAGTCAATGTTGATGGAAAAGTTTTAGATCGA
AAATCCATGGGGGAAGATTTGTTTTGGGCTATACGTGGTGGTGGAGGAGAAAACTTTGGAATCATTGCAGCGT
GGAAAATTAGACTTGTTGCTGTCCCATCAATGTCTACTATATTCAGTGTTAAAAAGAACATGGAGATACATGA
GCTTGTCAAGTTAGTTAACAAATGGCAAAATATTGCTTACATGTATGAAAAAGAATTATTACTCTTTACTCAC
TTTATAACCAGGAATATTACAGATAATCAAGGGAAGAATAAGACAACAATACACAGTTACTTCTCCTCCATTT
TCCATGGTGGAGTGGATAGTCTAGTCGACTTGATGAACAAGAGCTTTCCTGAATTGGGTATTAAAAAAACAGA
TTGCAAACAGTTGAGCTGGATTGATACTATCATCTTCTACAGTGGTGTTGTAAATTACAACACAACTAATTTT
AAAAAAGAAATTTTGCTTGATAGATCAGGTGGGCGGAAGGCGGCTTTCTCGATTAAGTTAGACTATGTTAAGA
AACCGATTCCAGAAACCGCAATGGTCACAATTTTGGAAAAATTATATGAAGAAGATGTAGGAGTTGGGATGTT
TGTGTTTTACCCTTATGGTGGTATAATGGATGAGATTTCAGAATCAGCAATTCCATTCCCTCATCGAGCTGGA
ATCACGTATGAAATTTGGTACATAGCTTCATGGGAGAAGCAAGAAGATAATGAAAAGCATATAAACTGGATTC
GGAATGTTTATAATTTCACGACTCCTTATGTGTCCCAAAATCCAAGAATGGCGTATCTCAATTATAGGGACCT
TGATTTAGGAAAAACTAATTTCGAGAGTCCTAATAATTACACACAAGCACGTATTTGGGGTGAAAAGTATTTT
GGTAAAAATTTTAATAGGTTAGTAAAAGTAAAAACCAAGGTTGATCCCGATAATTTCTTTAGAAACGAACAAA
GCATCCCACCTCTTCCCCTGCGTCATCATTAA
>C_Cannatonic_MNPR01003194_17359
ATGAAGTACTCAACATTCTGTTTTTGGTATGTTTGCAAGATAATATTTTTCTTTCTCTCATTCAATATCCAAA
TTTCAATAGCTAATCCTCAAGAAAACTTCCTTAAATGCTTCTCACAATATATTCCCACCAATGTAACAAATGC
AAAACTCGTATACACTCAACACGACCAATTTTATATGTCTATCCTAAATTCGACCATACAAAATCTTAGATTT
ACCTCTGACACAACCCCAAAACCACTTGTTATCATCGCTCCTTTAAATGTCTCCCATATCCAAGGCACTATTC
TATGCTCCAAGAAAGTTGGCTTGCAGATTCGAGCTCGAAGCAGTGGTCATGATGCTGAGGGCATGTCCTACAT
ATCTCAAGTCCCATTTGTTATAGTAGACTTGAGAAACATGCATTCGGTCAAAATAGATGTTCATAGCCAAACT
GCATGGGTTGAAGCCGGAGCTACCCTTGGAGAAGTTTATTATTGGATCAATGAGAACAATGAGAATCTTAGTT
TTCCTGCTGGGTACTGTCCTACTGTTGGCGCGGGTGGACACTTTAGTGAAGGAGGCTATGGAGCATTGATGCG
AAATTATGGCCTCGCGGCTGATAATATCATTGATGCGCACTTAGTCAATGTTGATGGAAAAGTTTTAGATCGA
AAATCCATGGGGGAAGATTTGTTTTGGGCTATACGTGGTGGTGGAGGAGAAAACTCTGGAATCATTGCAGCGT

GGAAAATTAGACTTGTTGCTGTCCCATCAATGTCTACTATATTCAGTGTTAAAAAGAACATGGAGATACATGA
GCTTGTCAAGTTAGTTAAAAAATGGCAAAATATTGCTTACATGTATGAAAAAGAATTATTACTCTTTACTCAC
TTTATAACCAGGAATATTACAGATAATCAAGGGAAGAATAAGACAACAATACACTGTTACTTCTCCTCCATTT
TCCATGGTGGACTGGATAGTCTAGTCGACTTGATGAACAAGAGCTTTCCTGAATTGGGTATTAAAAAAACAGA
TTGCAAACAGTTGAGCTGGATTGATACTATCATCTTCAACAGTGGTCTTGTAAATTACAACACTACTAATTTT
AAAAAAGAAATTTTGCTTTAAAGATCAGGTGGGCGGAAGGCGGCTTTCTCAATTAAGTTAGACTATGTTAAGA
AACCGATTCCAGAAACCGCAATGGTCACAATTTTGGAAAAATTATATGAAGAAGATGTAGGAGTTGGGATGTT
CGTGTTTTACCCTTATGGTGGTATAATGGATGAGATTTCAGAATCAGCAATTCCATTCCCTCATCGAGCTGGA
ATCATGTATGAAATTTGGTACATAGCTTCATGGGAGAAGCAAGAAGATAATGAAAAGCATATAAACTGGATTC
GGAATGTTTATAATTTCACGACTCCTTATGTGTCCCAAAATCCAAGAATGGCGTATCTCAATTATAGGGACCT
TGATTTAGGAAAAACTAATTTCGAGAGTCCTAATAATTACACACAAGCACGTATTTGGGGTGAAAAGTATTTT
GGTAAAAATTTTAGTAGGTTAGTAAAAGTAAAAACCAAGGTTGATCCCGATAATTTCTTTAGAAACGAACAAA
GCATCCCACCTCTTCCCCTGCGTCATCATTAA
>C_JLion_father_JAATIQ010000127.1_G4B88_028763
AAAAAATCCATGGGGGAAGATTTGTTTTGGGCTATACGTGGTGGTGGAGGAGAAAACTTTGGAATCATTGCAG
CGTGGAAAATTAGACTTGTTGCTGTCCCATCAATGTCTACTATATTCAGTAATAAAAAGAACATGGAGATACA
TGAGCTTGTCAAGTTAGTTAACAAATGGCAAAATATTGCTTACATGTATGAAAAAGAATTATTACTCTTTACT
CACTTTATAACCAGGAATATTACAGATAATCAAGGAAAGAATAAGACAACAATACATAGTTACTTCTCCTCCA
TTTTCCATGGTGGAGTGGATAGTCTAGTCGACTTGATGAACAAGAGCTTTCCTGAATTGGGTATTAAAAAAAC
AGATTGCAAACAGTTGAGCTGGATTGATACTATCATCTTCTACAGTGGTCTTGTAAATTACAACACTACTAAT
TTTAAAAAAGAACTTTTGCTTGATAGATCAGGTGGGCGGAAGGCGGCTTTCTCGATTAAGTTAGACTAAGTTA
AGAAACCGATTCCAGAAACCGCAATGGTCACAATTTTGGAAAAATTATATGAAGAAGATGTAGGAGTTGGGAT
GTTTGTGTTTTACCCTTATGGTGGAATAATGGATGAGATTTCAGAATCAGCAATTCCATTCCCTCATCGAGCT
GGAATCATGTATGAAATTTGGTACATAGCTTCATGGGAGAAGCAAGAAGATAATGAAAAGCATATAAACTGGA
TTCAGAATGTTTACAATTTCACGACTCCTTATGTGTCCCAAAATCCAAGAATGGCGTATCTCAATTATAGGGA
CCTTGATTTAGGAAAAACTAATTTCGAGAGTCCTAATAATTACACACAAGCACGTATTTGGGGTGAAAAGTAT
TTTGGTAAAAAATTTAATAGGTTAGTAAAAGTAAAAACCAAGGTTGATCCCGATAATTTCTTTAGAAACGAAC
AAAGCATCCCACCTCTTCCCCTACGTCATCATTAA
>C_Cannatonic_MNPR01003544_41587
TGAAAGTACTCAACATTCTTTTGGTATGTTTGCAAGATAATATTTTTCTTTCTCTCATTCAATATCCAAATTT
CAATAGCTAATCCTGAAGGAAACTTCCTTAAATGCTTCTCACAATATATTCCCACCAATGTAACAAATGCAAA
ACTCGTATACACTCAACACGACCAATTTTATATGTCTATCCTAAATTCGACCATACAAAATCTTAGATTTACC
TTTGACACAACCCCAAAACCACTTGTTATCATCACTCCTTTAAATGTCTCCCATATCCAAGGCACTATTCTAT
GCTCCAAGAAAGTTGGCTTGTAGATTCGAACTCGAAGCGGTGGTCATGATGCTGAGGGCATGTCCTACATATC
TCAAGTCCCATTTGTTATAGTAAACTTGAGAAACATGCATTCGGTCAAAATAGATGTTCATAGCGAAACTGCA
TGGGTTGAAGCCGGAGCTACCCTTGGAGAAGTTTATTATTGGATCAATGAGAACAATGAGAATCTTAGTTTTC
TTGCTGGGTACTGCCCTACTGTTGGCGCGGGTGGACACTTTAGTGGAGGAGGCTATGGAGCATTGATGCGAAA
TTATGGCCTCGCGGCTAATAACATCATTGATGCGCACTTAGTCAATGTTGATGGAAAAGTTTTAGATCGAAAA
TCCATGGGGGAAGATTTGTTTTGGGCTATACGTGGTGGAGGAGAAAACTTTGGAATCATTGCAACGTGGAAAA
TTAGACTTGTTGCTGTCCCATCAATGTCTACTATATTCAGTGTTAAAAAGAACATGGAGATACATGAGCTTGT
CAAGTTAGTTAACAAATGACAAAATATTGCTTACATGTATGAAAAAGAATGATTACTCTTTACTCACTTTATA
ACCAGGAATATTACAGATAATCAAGGGAAGAATAAGACAACAATACACAGTTACTTCTCCTCCATTTTCTATG
GTGGAGTGGATAGTCTAGTCGACTTGATGAACAAGAGCTTTCCTGAATTGGGTATTAAAAAAACAGATTGCAA
ACAGTTGAGCTGGATTGATACTATCATCTTCTACAGTGGTCTTGTAAATTACAACACTACTAATTTTAAAAAA
GAACTTTTGCTTGATAGATCAGGTGGGCGGAAGGCGGCTTTCTCGATTAAGTTAGACTAAGTTAAGAAACCGA
TTCCAGAAACCGCAATGGTCACAATTTTGGAAAAATTATATGAAGAAGATGTAGGAGTTGGGATGTTTGTGTT
TTACCCTTATGGTGGTATAATGGATGAGATTTCAGAATCAGCAATTCCATTCCCTCATCGAGCTGGAATCATG
TATGAAATTTGGTACATAGCTTCATGGGAGAAGCAAGAAGATAATGAAAAGCATATAAACTGGATTCGGAATG
TTTACAATTTCACGACTCCTTATGTGTCCCAAAATCCAAGAATGGCGTATCTCAATTATAGGGACCTTGATTT
AGGAAAAACTAATTTCGAGAGTCCTAATAATTACACACAAGCACGTATTTGGGGTGAAAAGTATTTTGGTAAA
AATTTTAATAGGTTAGTAAAAGTAAAAACCAAGGTTGATCCCGATAATTTCTTTAGAAACGAACAAAGCATCC
CACCTCTTCCCCTACGTCATCATTAA
>C_PBBK_MXBD01007873.1_8325
ATGAAGTACTCAACATTCTGTTTTTGGTATGTTTGCAAGATAATATTTTTCTTTCTCTCATTCAATATCCAAA
TTTCAATAGCTAATCCTCAAGAAAACTTCCTTAAATGCTTCTCACAATATATTCCCACCAATGTAACAAATGC
AAAACTCGTATACACTCAACACGACCAATTTTATATGTCTATCCTAAATTCGACCATACAAAATCTTAGATTT
ACCTCTGACACAACCCCAAAACCACTTGTTATCATCACTCCTTTAAATGTCTCCCATATCCAAGGCACTATTC
TATGCTCCAAGAAAGTTGGCTTGCAGATTCGAACTCGAAGCGGTGGTCATGATGCTGAGGGCATGTCCTACAT

ATCTCAAGTCCCATTTGTTATAGTAGACTTGAGAAACATGCATTCGGTCAAAATAGATGTTCATAGCCAAACT
GCATGGGTTGAAGCCGGAGCTACCCTTGGAGAAGTTTATTATTGGATCAATGAGAACAATGAGAATCTTAGTT
TTCCTGCTGGGTACCCTACTGTTGGCGCGGGTGGACACTTTAGTGGAGGAGGCTATGGAGCATTGATGCGAAA
TTATGGCCTCGCGGCTGATAATATCATTGATGCGCACTTAGTCAATGTTGATGGAAAAGTTTTAGATCGAAAA
TCCATGGGGGAAGATTTGTTTTGGGCTATACGTGGTGGTGGAGGAGAAAACTTTGGAATCATTGCAGCGTGGA
AAATTAGACTTGTTGCTGTCCCATCAATGTCTACTATATTCAGTGTTAAAAAGAACATGGAGATACATGAGCT
TGTCAAGTTAGTTAAATGGCAAAATATTGCTTACATGTATGAAAAAGAATTATTACTCTTTACTCACTTTATA
ACCAGGAATATTACAGATAATCAAGGGAAGAATAAGACAACAATACACAGTTACTTCTCCTCCATTTTCCATG
GTGGAGATAGTCTAGTCGACTTGATGAACAAGAGCTTTCCTGAATTGGGTATTAAAAAAACAGATTGCAAACA
GTTGAGCTGGATTGATACTATCATCTTCTACAGTGGTCTTGTAAATTACAACACTAATTTTAAAAAAGAAATT
TTGCTTGATAGATCAGGTGGGCGGAAGGCGGCTTTCTCGATTAAGTTAGACTATGTTAAGAAACCGATTCCAG
AAACCGCAATGGTCACAATTTTGGAAAAATTATATGAAGAAGATGTAGGAGTTGGGATGGTGTTTTACCCTTA
TGGTGGTATAATGGATGAGATTTCAGAATCAGCAATTCCATTCCCTCATCGAGCTGGAATCATGTATGAAATT
TGGTACATAGCTTCATGGGAGAAGCAAGAAGATAATGAAAAGCATATAAACTGGATTCGGAATGTTTATAATT
TCACGACTCCTTATGTGTCCCAAAATCCAAGAATGGCGTATCTCAATTATAGGGACCTTGATTTAGGAAAAAC
TAATTTCGAGAGTCCTAATAATTACACACAAGCACGTATTTGGGGTGAAAAGTATTTTGGTAAAAATTTTAAT
AGGTTAGTAAAAAAAACCAAGGTTGATCCCGATAATTTCTTTAGAAACGAACAAAGCATCCCACCTCTTCCCC
TGCGTCATCATTAA
>C_PBBK_MXBD01002930.1_46853
TTCAATATCCAAATTTCAATAGCTAATCCTCAAGAAAACTTCCTTAAATGCTTCTCACAATATATTCCCACCA
ATGTAACAAATGCAAAACTCGTATACACTCAACACGACCAATTTTATATGTCTATCCTAAATTCGACCATACA
AAATCTTAGATTTACCTCTGACACAACCCCAAAACCACTTGTTATCATCGCTCCTTTAAATGTCTCCCATATC
CAAGGCACTATTCTATGCTCCAAGAAAGTTGGCTTGCAGATTCGAGCTCGAAGCAGTGGTCATGATGCTGAGG
GCATGTCCTACATATCTCAAGTCCCATTTGTTATAGTAGACTTGAGAAACATGCATTCGGTCAAAATAGATGT
TCATAGCCAAACTGCATGGGTTGAAGCCGGAGCTACCCTTGGAGAAGTTTATTATTGGATCAATGAGAACAAT
GAGAATCTTAGTTTTCCTGCTGGGTACTGTCCTACTGTTGGCGCGGGTGGACACTTTAGTGGAGGAGGCTATG
GAGCATTGATGCGAAATTATGGCCTCGCGGCTGATAATATCATTGATGCGCACTTAGTCAATGTTGATGGAAA
AGTTTTAGATAAATCCATGGGGGAAGATTTGTTTTGGGCTATACGTGGTGGTGGAGGAGAAAACTCTGGAATC
ATTGCAGCGTGGAAAATTAGACTTGTTGCTGTCCCATCAATGTCTACTATATTCAGTGTTAAAAAGAACATGG
AGATACATGAGCTTGTCAAGTTAGTTAAATGGCAAAATATTGCTTACATGTATGAAAAAGAATTATTACTCTT
TACTCACTTTATAACCAGGAATATTACAGATAATCAAGGGAAGAATAAGACAACAATACACAGTTACTTCTCC
TCCATTTTCCATGGTGGAGATAGTCTAGTCGACTTGATGAACAAGAGCTTTCCTGAATTGGGTATTAAAACAG
ATTGCAAACAGTTGAGCTGGATTGATACTATCATCTTCAGTGGTCTTGTAAATTACAACACTACTAATTTTAA
AGAAATTTTGCTTTCAGGTGGGCGGAAGGCGGCTTTCTCAATTAAGTTAGACTATGTTAAGAAACCGATTCCA
GAAACCGCAATGGTCACAATTTTGGAAAAATTATATGAAGAAGATGTAGGAGTTGGGATGTTCGTGTTTTACC
CTTATGGTGGTATAATGGATGAGATTTCAGAATCAGCAATTCCATTCCCTCATCGAGCTGGAATCATGTATGA
AATTTGGTACATAGCTTCATGGGAGAAGCAAGAAGATAATGAAAAGCATATAAACTGGATTCGGAATGTTTAT
AATTTCACGACTCCTTATGTGTCCCAAAATCCAAGAATGGCGTATCTCAATTATAGGGACCTTGATTTAGGAA
AAACTAATTTCGAGAGTCCTAATAATTACACACAAGCACGTATTTGGGGTGAAAAGTATTTTGGTAAAAATTT
TAGGTTAGTAAAAGTAAAAACCAAGGTTGATCCCGATAATTTCTTTAGAAACGAACAAAGCATCCCACCTCTT
CCCCTGCGTCATCATTAA
>C_Cannatonic_MNPR01005369_10312
ATGAAGTACTCAACATTCTGGTATGTTTGCAAGATAATATTTTTTTTTCTCTCATTCAATATCCAAATTTCAA
TAGCTAATCCTCAAGAAAACTTCCTTAAATGCTTCTCACAATATATTCCCACCAATGTAACAAATGCAAAACT
CGTATACACTCAACACGACCAATTTTATATGTCTATCCTAAATTCGACCATACAAAATCTTAGATTTACCTCT
GACACAACCCCAAAACCACTTGTTATCATCACTCCTTTAAATGTCTCCCATATCCAAGGCACTATTCTATGCT
CCAAAAAAGTTGGCTTGCAGATTCGAACTCGAAGCGGTGGTCATGATGCTGAGGGCATGTCCTACATATCTCA
AGTCCCATTTGTTATAGTAGACTTGAGAAACATGCATTCGGTCAAAATAGATGTTCATAGCCAAACTGCATGG
GTTGAAGCCGGAGCTACCCTTGGAGAAGTTTATTATTGGATCAATGAGAACAATGAGAATCTTAGTTTTCCTG
CTTACTGCCCTACTGTTGGCGCGGGACACTTTAGTGGAGGAGGCTATGGAGCATTGATGCGAAATTATGGCCT
CGCGGCTGATAATATCATTGATGCGCACTTAGTCAATGTTGATGGAAAAGTTTTAGATCGAAAATCCGGGGAA
GATTTGTTTTGGGCTATACGTGGTGGTGGAGGAGAAAACTTTGGAATCATTGTAGCGTGGAAAATTAGACTTG
TTGCTGTCCCATCAATGTCTACTATATTCAGTGTTAAAAAGAACATGGAGATACATGAGCTTGTCAAGTTAGT
TAACAAATGGCAAAATATTGCTTACATGTATGAAAAAGAATTATTACTCTTTACTCACTTTATAACCAGGAAT
ATTACAGATAATCAAGGGAAGAATAAGACAACAATACACAGTTACTTCTCCTCCATTTTCCATGGTGGAGTGG
ATAGTCTAGTCGACTTGATGAACAAGAGCTTTCCTGAATTGGGTATTAAAATAGATTGCAAACAGTTGAGCTG
GGATACTATCATCTTCTACAGTGGTCTTGTAAATTACAACACAACTAATTTTAAAAAAGAAATTTTGCTTGAT
AGATCAGGTGGGCGGAAGGCGGCTTTCTCGATTAAGTTAGACTATGTTAAGAAACCGATTCCAACCGCAATGG

TCACAATTTTGGAAAAATTATATGAAGAAGATGTAGGAGTTGGGATGTTTGTGTTTTACCCTTATGGTGGTAT
AATGGATGAGTCAGAATCAGCAATTCCATTCCCTCATCGAGCTGGAATCATGTATGAAATTTGGTACATAGCT
TCATGGGAGAAGCAAGAAGATAATGAAAAGCATATAAACTGGATTCGGAATGTTTATAATTTCACGACTCCTT
ATGTGTCCCAAAATCCAAGAATGGCGTATCTCAATTATAGGGACCTTGATTTAGGAAAAACTAATTTTGAGAG
ACCTAATAATTACACACAAGCACGTATTTGGGGTGAAAAGTATTTTGGTAAAAATTTTAATAGGTTAGTAAAA
AAAAACAAGGTTGATCCCGATAATTTCTTTAGAAACGAACAAAGCATCCCACCTCTTCCCCTGCGTCATCATT
AT
>H_Finola_CM011607.1_10525384
ATGAAGTACTCATCAGTATCTTGGTTTCTTTGCAAAATATTATTAGTTTATCATCTTTCATTCTCTATCCAAA
CTTCTCAAGCTAATCCTCACAACAACTTTCTTCAATGCTTCTCCCATATCTCCAACAACAATACATCACTTGC
CAAACTCATACACACTCCAGACTCTTCATTTATCTCCCTTCTAAATTCAACCATACAGAACCTTAGATTCACT
TCTCCAACACCAAAACCACTAGTTATCATCACACCTTCAAATACATCCCATGTCCAAGCCTGTGTTTTATGTT
CCAAGAAATATGGCTTACAGATTCGAACTCGAAGCGGCGGCCATGACTTTGAGGGTGTCTCCTATGTGTCTAA
AGTCCCATTTGTGATATTAGATATGAGAAATCTACGTTCAATCACTGTAGACGTAGATAACAAAACTGCATGG
GTTGAATCTGGAGCTACCCTTGGAGAACTTTATTATAGGATCGCTGAGAAAAATGAGAATCTTAGTTTTCCTG
GTGGCTATTGCCATAGTGTTGGGGTTGGTGGGCATTTCAGTGGAGGAGGCTATGGAGCATTGATGCGAAAATA
TGGCCTTGCAGCTGATAATGTCATTGATGCTCACTTAGTCAACGCTGATGGAGAATTCGTTGACCGAAAATCA
ATGGGAGAAGATTTGTTTTGGGCCATTCGTGGTGGTGGTGGAGCAAGCTTTGGAATTGTTCTTGCTTGGAAAA
TTAGATTGGTTCCTGTGCCATCTAAGGTTACTGTATTATCAGTTAGTAAGAACTTGCCGATAAATGAAACTGT
GAAAATTTATAATAAGTGGCAAAATATTGCTCACAAGTTTGACCAAGATTTGTTAATGGTAGTTAGGTTCTTA
ACTGTGAATTCTACTGATGAGCATGGGAAGAATATGACAACAATACAAGCTACATTCTTTTCTATTTTTCTTG
GTAGAGTGGATAATTTCCTTTCCTTGATGCAAACTAACTTTCCTGAGTTGGGTGTAGTAAGAAAAGATTGTTT
TGAAACGAGTTGGATTGAAATGATCTTTTTCTTCAATGAATTCTCAAGTGAAGATAAATTGGAGGTTTTGCTC
GATCCAACAAATGTAGTAAAGGGTTATTTCAAGGGGAAACTAGACTACGTTAGGAAGCCAATTTCAGAAATTG
TTATGGTCAAACTTTTGGAAAGGTTATATGAAGAAGATGTAGGATTGGCATATATTCAAATGTACCCTTATGG
TGGTAAAATGAGCGAGATTCCTGAATCTGCAATTCCATTCCCACATCGAGCTGGAGTTATGTACAAAATTTTA
TATTGGTCTCAGTGGGAAAAAGAAGAAGAAAGTGAAAGGCATATGAATTGGGTTCGAAGTGTTTATAATTACA
TGACTCCATACGTGTCCAAAAATCCAAGAGCTTCATATATTAATTATAGAGACCTAGATTTGGGAACAAATAA
TGAAAAAGGTCCTATAAGTTATGAACAAGCAAGCATTTGGGGGAAAAAGTACTTCAATAAAAATTTTAAGTTA
GTTCAAGTGAAAACCAAGGTTGACCCGACTAATTTCTTTAGGAACGAACAGAGCATTCCACCTTCATTTCCAC
GACTTCTCTAA
>H_Purple_Kush__CM010793.2_63060305
ATGAAGTACTCATCAGTATCTTGGTTTCTTTGCAAAATATTATTAGTTTATCTTCTTTCATTCTCTATCCAAA
CTTCTCAAGCTAATCCTCACAACAACTTTCTTCAATGCTTCTCCAAACATATCTCCAACAACAATACATCACT
TGCCAAACTCATACACACTCCAAATGACTCATATATCTCCCTTCTAAATTCAACCATACAGAACCTTAGATTC
ACTTCAACAACACCAAAACCACTAGTTATCATCACACCTTCAAATACATCCCATGTCCAAGCCTGTGTTTTAT
GTTCCAAGAAATATGGCTTACAGATTCGAACTCGAAGCGGCGGCCATGACTTTGAGGGTGTCTCCTATGTGTC
TAAAGTCCCATTTGTGATATTAGATATGAGAAATCTACGTTCAATCACTGTAGACGTAGATAACAAAACTGCA
TGGGTTGAATCTGGAGCTACCCTTGGAGAACTTTATTATAGGATTGCTGAGAAAAATGAGAATCTTAGTTTTC
CTGGTGGCTATTGCCATAGTGTTGGGGTTGGTGGGCATTTCAGTGGAGGAGGCTATGGAGCATTGATGCGAAA
ATATGGCCTTGCAGCTGATAATGTCATTGATGCTCACTTAGTCAACGCTGATGGAGAATTCGTTGACCGAAAA
TCTATGGGAGAAGATTTGTTTTGGGCCATTCGTGGTGGTGGTGGAGCAAGCTTTGGAATTGTTCTTGCTTGGA
AAATTAGATTGGTTCCTGTGCCATCTAAGGTTACTGTATTATCAGTTAGTAAGAACTTGCCGATAAATGAAAC
TGTGAAAATTTATAATAAGTGGCAAAATATTGCTCACAAGTTTGACCAAGATTTGTTGATGGTAGTTAGGTTC
TTAACTGTGAATTCTACTGATGAGCATGGGAAGAATATGACAACAATACAAGCTACATTCTTTTCTATTTTTC
TTGGTAGAGTGGATAATTTTCTTTCCTTGATGCAAACTAACTTTCCTGAGTTGGGTGTAGTAAGAAAAGATTG
TTTTGAAACGAGTTGGATTGAAATGATCTTTTTCTTCAATGAATTCTCAAGTGAGGATAAATTGGAGGTTTTG
CTCGATCCAACAAATGTAGTAAAGGGTTATTTCAAGGGGAAACTGGACTACGTTAGGAAGCCAATTTCAGAAA
TTGTTATGGTCAAACTTTTGGAAAAGTTATATGAAGAAGATGTAGGATTGGCATATATTCAAATGTACCCTTA
TGGTGGTAAAATGAGCGAGATTCCTGAATCTGCAATTCCATTCCCACATAGAGCTGGAGTTATGTACAAAATT
TTATATTGGTCTCAGTGGGAAAAAGAAGAAGAAAGTGAAAGGCATATGAATTGGGTTCGAAGTGTTTATAATT
ACATGACTCCATACGTGTCCGAAAATCCAAGAGCTTCATATATTAATTATAGAGACCTAGATTTGGGAACAAA
TAATGAAAAAGGTCCTATAAGTTATGAACAAGCAAGCATTTGGGGGAAAAAGTACTTCAATAAAAATTTTAAG
AAATTAGTTCAAGTGAAAACCAAGGTTGACCCGACTAATTTCTTTAGGAACGAACAGAGCATTCCACCTTTAT
CTCCACGATTTCTCTAA
>H_CBDRx_NC_044375.1_LOC115720716
ATGAAGTACTCATCAGTATCTTGGTTTCTTTGCAAACTATTATTAGTTTATCTTCTTTCATTCTCTATCCAAA
CTTCTCAAGCTAATCCTCACAACAACTTTCTTCAATGCTTCTCCAAACATATCTCCAACAACAATACATCACT

TGCCAAACTCATACACACTCCAAATGACTTTTCATATATCTCCCTTCTAAATTCAACCATACAAAACCTTAGA
TTCACTTCTCCAACACCAAAACCACTAGTTATCATCACACCTTCAAATACATCCCATGTCCAAGCCTGTGTTT
TATGTTCCAAGAAATATGGCTTACAGATTCGAACTCGAAGCGGCGGCCATGACTTTGAGGGTGTCTCCTATGT
GTCTAAAGTCCCATTTGTGATATTAGATATGAGAAATCTACGTTCAATCACTATAGACGTAGATAACAAAACT
GCATGGGTTGAATCTGGAGCTACCCTTGGAGAACTTTATTATAGAATCGCTGAGAAAAATGAGAATCTTAGTT
TTCCCGGTGGCTATTGCCATAGTGTTGGGGTTGGTGGGCATTTCAGTGGAGGAGGCTATGGAGCATTGATGCG
AAAATATGGTCTTGCAGCTGATAATGTCATTGATGCTCACTTAGTCAATGCTGATGGAGAATTCGTTGACCGA
AAATCTATGGGAGAAGATTTGTTTTGGGCCATTCGTGGTGGTGGTGGAGCAAGCTTTGGAATTGTTCTCGCTT
GGAAAATTAGATTGGTTCCTGTGCCATCGAAGGTTACTATATTATCGGTTAGTAAGAACTTGCCGATAAATGA
AACTGTTAAAATATATAATAAGTGGCAAAATATTGCTCACAAGTTTGACCAAGATTTGTTAATGGTAGTTAGG
TTCTTAACTGTGAATTCTACTGATGAGCATGGGAAGAATATGACAACAATACAAGCTACATTCTTTTCTATTT
TTCTTGGTAGAGTGGATAAGTTTCTTTCCTTGATGCAAACTAACTTTCCTGAGTTGGGTGTAGTAAGAAAAGA
TTGTTTTGAAACGAGTTGGATTGAAATGATCTTTTTCTTCAATGAATTCTCAAGTGAGGATAAATTGGAGGTT
TTGCTCGATCCAACAAATGTAGTAAAGGGTTATTTTAAGGGGAAACTAGACTACGTTAGGAAGCCAATTTCAG
AAATTGTTATGGTCAAACTTTTGGAAAAGTTATATGAAGAAGATGTAGGATTGGCATATATTCAAATGTACCC
TTATGGTGGTAAAATGAGCGAGATTCCTGAATCTGCAATTCCATTCCCACATAGAGCTGGAGTTATGTACAAA
ATTTTATATTGGTCTCAGTGGGAAAAAGAAGAAGAAAGTGAAAGGCATATGAATTGGGTTCGAAGTGTTTATA
ATTACATGACTCCATACGTGTCCAAAAAATCCAAGAGCTTCATATATTAATTATAGAGACCTAGATTTGGGAAC
AAATAATGAAAAAGGTCCTATAAGTTATGAACAAGCAAGCATTTGGGGGAAAAAGTACTTCAATAAAAAATTTT
AAGAAATTAGTTCAAGTGAAAACCAAGGTTGACCCGACTAATTTCTTTAGGAACGAACAGAGCATTCCACCTT
CATTTCCACGACTTCTCTAA
>H_Jamaican_Lion_JAATIP010000254.1_F8388_000722
ATGAAGTACTCATCAGTATCTTGGTTTCTTTGCAAAATATTATTAGTTTATCTTCTTTCATTCTCTATCCAAA
CTTCTCAAGCTAATCCTCACAACAACTTTCTTCAATGCTTCTCCAAACATATCTCCAACAACAATACATCACT
TGCCAAACTCATACACACTCCAAATGACTCTTCATTTATCTCCCTTCTAAATTCAACCATACAAAACCTTAGA
TTCACTTCTCCAACACCAAAACCCCTAGTCATCATCACACCTTCAAATACATCCCATGTCCAAGCGTGTGTTT
TATGTTCCAAGAAATATGGCTTACAGATTCGAACTCGAAGCGGCGGCCATGACTTTGAGGGTGTCTCCTTTGT
GTCTAAAGTCCCATTTGTGATATTAGATATGAGAAATCTACGTTCAATCACTATAGACGTAGATAACAAAACT
GCATGGGTTGAATCTGGAGCTACCCTTGGAGAACTTTATTATAGGATTGCTGAGAAAAATGAGAATCTTAGTT
TTCCTGGTGGCTATTGCCATAGTGTTGGGGTTGGTGGGCATTTCAGTGGAGGAGGCTATGGAGCGTTGATGCG
AAAATATGGCCTTGCAGCTGATAATGTCATTGATGCTCACTTAGTCAACGCTGATGGAGAATTCGTTGACCGA
AAATCTATGGGAGAAGATTTGTTTTGGGCCATTCGTGGTGGTGGTGGAGCAAGCTTTGGAATTGTTCTCGCTT
GGAAAATTAGATTGGTTCCTGTGCCATCTAAGGTTACTGTATTATCAGTTAGTAAGAACTTGCCGATAAATGA
AACTGTTAAAATATATAATAAGTGGCAAAATATTGCTCACAAGTTTGACCAAGATTTGTTAATGGTAGTTAGG
TTCTTAACTGTGAATTCTACTGATGAGCATGGGAAGAATATGACAACAATACAAGCTACATTCTTTTCTATTT
TTCTTGGTAGAGTGGATAAGTTTCTTTCCTTGATGCAAACTAACTTTCCTGAGTTGGGTGTAGTAAGAAAAGA
TTGTTTTGAAACGAGTTGGATTGAAATGATCTTTTTCTTCAATGAATTCTCAAGTGAAGATAAATTGGAGGTT
TTGCTCGATCCAACAAATGTAGTAAAGGGTTATTTCAAGGGGAAACTAGACTACGTTAGGAAGCCAATTTCAG
AAATTGTTATGGTCAAACTTTTGGAAAAGTTATATGAAGAAGATGTAGGATTGGCATATATTCAAATGTACCC
TTATGGTGGTAAAATGAGCGAGATTCCTGAATCTGCAATTCCATTCCCACATAGAGCTGGAGTTATGTACAAA
ATTTTATATTGGTCTCAGTGGGAAAAAGAAGAAGAAAGTGAAAGGCATATGAATTGGGTTCGAAGTGTTTATA
ATTACATGACTCCATACGTGTCCGAAAATCCAAGAGCTTCATATATTAATTATAGAGACCTAGATTTGGGAAC
AAATAATGAAAAAGGTCCTATAAGTTATGAACAAGCAAGCATTTGGGGGAAAAAGTACTTCAATAAAAAATTTT
AAGAAATTAGTTCAAGTGAAAACCAAGGTTGACCCGACTAATTTCTTTAGGAACGAACAGAGCATTCCACCTT
TATCTCCACGATTTCTCTAA
>H_H._lupulus_000840F.g23
ATGAAGCACTCAGTATTCTCTTATTGGTTTCTTTGCAAAATAGTCAATATTTCTCTTCTCTCATTCTCAATCC
GATCTACTCGAGCTGATCCTCACGCTGATTTCCTTCAATGCTTCTCCCAATATATCTCAAACAGTACAACCAT
TGCCAAACTCATCTACACTCCAAATGATCCGTTGTATATCTCCATCCTAAACTCGACTATACAAAACAACAGA
TTTTCTTCTCCTTCAACCCCTAAACCACTGATTATCATTACACCATTAAATTCCTTCCATGTACAAGCCTCCA
TTCTATGCTCCAGGAAATATGGCTTGCAAATTCGAACCCGAAGCGGCGGCCATGACTTTGAGGGTGTCTCCTA
TGTGTCTGAAGTCCCATTTGTCATAGTAGACATGAGAAACCTACGTTCCATCACCATTGACGTAGATAACAAA
ACTGCATGGGTTGATGTTGGAGCTACCCTTGGAGAACTGTATTATAGGATTGCTGAGAAAAATGAGAATCTCA
GTTTTCCTGCTGGCTATTGCCATACCGTTGGCGTAGGTGGGCACTTCAGTGGAGGAGGCTATGGAGCATTGAT
GCGAAAATATGGGCTCGCAGCTGATAATGTCATTGATGCTCACTTAGTCAATGTTGATGGAGAAGTTCTTGAC
CGACAATCCATGGGGGAAGATCTGTTTTGGGCCATACGTGGTGGTGGAGGAGCAAGCTTTGGAATCATTCTTG
CTTGGAAAATTAGATTGGTTCCTGTCCCATCAAAGGTTACTATAGTCTCCATTAATAAGAACTTGGAGATAAA
TGAAACCGTGAAGCTTTACAACAAGTGGCAAAATATTGCTCACAAGTTTGACAAAGATTTATTAATCTTTGTA

AGGTTCACAACTATGAATTCTACTGATGGACAAGGGAAGAACAAGACGGCAATACTAACTTCATTCTACTCTA
TTTTCTTTGGTGGAATGGATGGTTTACTTGCTTTGATGGAAAAGAGCTTTCCTGAGTTGGATGTGAAAAGAAA
AGATTGTTTCGAAGCAAGCTGGATCGAAATGATCTTTTACTTCAACGGATTCTCAAGTGGGGATAAATTGGAA
GTCTTGCTTGGTAGAACAAATGAAGAAAAGGGTTTTTTCAAGGCAAAACTAGACTACGTTAGGAAGCCGATAC
CAGAAACAGTAATAGTCAAACTTTTGGAGAAGTTATATAATGAAGATGTAGGATTGGGATTGATTCAAATGTA
CCCGTATGGTGGTAAAATGGACGAGATTCCTGAATCTGCAATTCCATTCCCTCACCGAGTTGGATTCATTTAC
AAAATTTTGTACTTGTCTCAATGGGAGAAAGAAGAAGAAGGTGAAAGACATCTGAACTGGGTTCGGAGTGTTT
ACAATTACATGACTCCCTTTGTGTCTAAAAGTCCAAGAGCTTCATATCTCAATTATAGGGACTTTGATTTGGG
AACAAATAACAAAAATGGTCCTACTAGTTATGGACAAGCAAGCATTTGGGGAAAAAAGTATTTCGATAAAAAC
TTTAAGAGGTTAGTTCATGTGAAAACGAAGGTTGATCCCACTAATTTCTTCAGGAACGAACAAAGCATCCCAC
CTCTTTCAGTGCGAGGTCTCTAA
>Out_CBDRx_NC_044375.1_LOC115718836
ATGAAGTACTCAACATTCTCTTTTTGGTTTCTTTGCAAAATATTAGTTTCACTTCTCTCATTCTCTATCCAAA
CTTCTCAAGCTAATCCACATGACAACTTTCTTCAATGCTTCTCCAAACATATCAACAACAATAACAATAAATC
AATTGTAAAACTCATACACACTCCAAATGATCCATCATATATCTCTGTCCTAAATTCAACTATACAAAACCTT
AGATTCGCTTCTCCTTCAACACCAAAACCACTAGTTATCATCACACCTTCAAATACATCCCATGTCCAAGCCT
GTGTTTTATGCTCCAAGAAATATGGCTTGCAGATTCGAACTCGAAGCGGCGGCCATGACTTTGAGGGTGCCTC
CTATGTGTCTAAAGTCCCATTTGTGATATTAGATATGAGAAATCTACGTTCAATCACTGTAGACGTAGATACC
AAAACTGCATGGGTTGAAGCTGGAGCTACCATTGGTGAACTTTATTATAGGATTGCTGAGAAAAATGGGAATC
TGAGTTTTCCTGCTGGGTATTGTCGTACTGTTGGGGTAGGTGGCATTTCAGTGGAGGAGGCTATGGAGCACT
GTTGCGAAAATATGGCCTTGCAGCTGATAATATCATTGATGCTCACTTAGTCAACGCTGATGGAGAATTTGTT
GACCGAAAATCTATGGGAGAAGATTTGTTTTGGGCCATACGTGGTGGTGGTGGAGCAAGCTTTGGAATCATTC
TCGCTTGGAAAATCAGATTGGTTGCAGTTCCATCTAAAGTTACTATGTTCTCTGTTAGTAAAAACTTGGAGAT
GAATGAGACTGTGAAGATATATAACAAATGGCAAAATATTGCTTACAAGTTTGACAAAGATTTGTTACTCTTT
GTTAGCTTCATGACTATTAATTCTACCGATTCACAAGGGAAATACAAGACAACTATACAAGCTTCATTCTCTT
CTATATTTCTTGGTAGGGTTGAGAGTCTCCTCATATTGATGCAAAAGAAATTTCCTGAGTTGGGAATTGAAAG
AAAAGATTGCCTCGAAAAGAGCTGGATTGAAACTGTCGTTTACTTTGATGGTTTTTCAAGTGGGGATACACCA
GAATCTTTACTTAATACAACATTTCAACAAAATGTATTTTTCAAGGTGAAATTAGACTATGTAAAGAAGCCAG
TTCGAGAAGTTGTGATGGTAAAACTTTTGGAGAAGTTATATGAAGAAGATGTAGGAGTGGGGTTTCTTATGAT
GTACCCTTATGGTGGTAAAATGGATGAGATTTCAGAATCAGCAATTCCATTCCCTCACCGAGCTGGATTTATG
TACAAAATTTTGTACTTGTCTGCATGGGAGAAAGAAGGAGAAAGTGAAAAGCATATGAATTGGGTTCGAAGTG
CATATAATTTCATGTCTCCTTATGTGTCTCAAAATCCAAGAGCTACATATCTCAATTATAGGGACCTTGATTT
GGGAACAAATAACGAGAAGGGTCCTATTAGTTACTCACAAGCAAGTGTTTGGGGTAAAAAGTATTTTGGTATG
AACTTTAAGAGGTTAGTTAATGTGAAAACCAAGGTCGATCCAAGTAATTTCTTTAGAAACGAACAAAGCATCC
CACCACTTATGTCGCGACGCCTCTAA
>Out_Finola_CM011607.1_10150821
ATGAAGTACTCAACATTCTCTTTTTGGTTTCTTTGCAAAATATTAGTTTCACTTCTCTCATTCTCTATCCAAA
CTTCTCGAGCTAATCCACATGAAAAGTTTCTTCAATGCTTCTCCAAACATATCAACAACAATAACAATACATC
AATTGTAAAACTCATACACACTCCAAATGATCCATCATATATCTCTGTCCTAAATTCAACTATACAAAACCTT
AGATTCGCTTCTCCTTCAACACCAAAACCACTAGTTATCATCACACCTTCAAATACATCTCATGTCCAAGCCT
GTGTTTTATGCTCCAAGAAATATGGCTTGCAGATTCGAACTCGAAGCGGCGGCCATGACTTTGAGGGTGCCTC
CTATGTGTCTAAAGTCCCATTTGTGATATTAGATATGAGAAATCTACGTTCAATCACTGTAGACGTAGATACC
AAAACTGCATGGGTTGAAGCTGGAGCTACCATTGGTGAACTTTATTATAGGATTGCTGAGAAAAATGGGAATC
TGAGTTTTCCTGCTGGGTACTGTCGTACTGTTGGGGTAGGTGGCATTTCAGTGGAGGAGGCTATGGAGCACT
GTTGCGAAAATATGGCCTTGCAGCTGATAATATCATTGATGTTCACTTAGTCAACGCAGATGGAGAATTCGTT
GACCGAAAATCCATGGGAGAAGATTTGTTTTGGGCTATACGTGGTGGTGGTGGAGCAAGCTTTGGAATCATTC
TCGCTTGGAAAATCAGATTGGTTGCAGTTCCATCAAAAGTTACTATGTTCTCTGTTAGTAAAAACTTGGAGAT
GAATGAGACTGTGAAGATATATAACAAATGGCAAAATATTGCTTACAAGTTTGACAAAGATTTGTTACTCTTT
GTTAGCTTCATGACTATTAATTCTACCGATTCACAAGGGAAATACAAGACAACTATACAAGCTTCATTCTCTT
CTATATTTCTTGGTAGGGTTGAGAGTCTCCTCACATTGATGCAAAAGAGATTTCCTGAGTTGGGAATTGAAAG
AAAAGATTGCCTCGAAAAGAGCTGGATTGAAACTGTCGTTTACTTTGATGGTTTTTCAAGTGGGGATACACCA
GAATCTTTACTTAATACAACATTTCAACAAAATGTATTTTTCAAGGTGAAATTAGACTATGTAAAGAAGCCAG
TTCCAGAATTTGTGATGGTAAAACTTTTGGAGAAGTTATATGAAGAAGATGTAGGTGTGGGGTTTCTTATGAT
GTACCCTTATGGTGGTAAAATGGATGAGATTTCAGAATCAGCAATTCCATTCCCTCATCGAGCTGGATTTATG
TACAAAATTTTGTACTTGTCTGCATGGGAGAAAGAAGGAGAAAGTGAAAAGCATATGAATTGGGTCCGAAGTG
CATATAATTTCATGTCTCCTTATGTGTCTCAAAATCCAAGAGCTACATATCTCAATTATAGGGACCTTGATTT
GGGAACAAATAACGAGAAGGGTCCTATTAGTTACTCACAAGCAAGTGTTTGGGGTAAAAAGTATTTCGGTATG

AACTTTAAGAGGTTAGTTAATGTGAAAACCAAGGTCGATCCAAGTAATTTCTTTAAAAACGAACAAAGCATCC
CACCACTTCTGTCGCGACGCCTCTAA
>Out_Jamaican_Lion_JAATIP010000254.1_F8388_000720
ATGAAGTACTCAACATTCTCTTTTTGGTTTCTTTGCAAAATATTAGTTTCACTTCTCTCATTCTCTATCCAAA
CTTCTCAAGCTAATCCACATGAAAACTTTCTTCAATGCTTCTCGAAACATATCAACAACAATAACAATACATC
AATTGTAAAACTCATACACACTCCAAATGATCCATCATATATCTCTGTCCTAAATTCAACTATACAAAACCTT
AGATTTGCTTCTCCTTCAACACCAAAACCACTAGTTATCATCACACCTTCAAATACATCCCATGTCCAAGCCT
GTGTTTTATGCTCCAAGAAATATGGCTTGCAGATTCGAACTCGAAGCGGCGGCCATGACTTTGAGGGTGCCTC
CTATGTGTCTAAAGTCCCATTTGTGATATTAGATATGAGAAATCTACGTTCAATCACTGTAGACGTAGATACC
AAAACTGCATGGGTTGAAGCTGGAGCTACCATTGGTGAACTTTATTATAGGATTGCTGAGAAAAATGGGAATC
TGAGTTTTCCTGCTGGATACTGTCGTACTGTTGGGGTAGGTGGGCATTTCAGTGGAGGAGGCTATGGAGCATT
GTTCCGAAAATATGGTCTTGCAGCAGATAATATCATTGATGCTCACTTAGTCAACGCTGATGGAGAATTCCTT
GACCGAAAATCTATGGGAGAAGATTTGTTTTGGGCCATACGTGGTGGTGGTGGAGCAAGCTTTGGAATCATTC
TCGCTTGGAAAATCAGATTGGTTGCAGTTCCATCTAAAGTTACTATGTTCTCTGTTAGTAAAAACTTGGAGAT
GAATGAGACTGTGAAGATATATAACAAATGGCAAAATATTGCTTACAAGTTTGAAAAAGATTTGTTACTCTTT
GTTAGTTTCATGACTATTAATTCTACCGATTCACAAGGGAAATACAAGACAACTATACAAGCTTCATTCTCTT
CTATATTTCTTGGTAGGGTTGAGAGTCTCCTCACATTGATGCAAAAGAAATTTCCTGAGTTGGGAATTGAAAG
AAAAGATTGCCTCGAAAAGAGCTGGATTGAAACTGTCGTTTACTTTGATGGTTTTTCAAGTGGGGATACACCA
GAATCTTTACTTAATACAACATTTCAACAAAATGTATTTTTCAAGGTGAAATTAGACTATGTAAAGAAGCCAG
TTCCAGAAGTTGTGATGGTAAAACTTTTGGAGAAGTTATATGAAGAAGATGTAGGTGTGGGGTTTCTTATGAT
GTACCCTTATGGTGGTAAAATGGATGAGATTTCAGAATCAGCAATTCCATTCCCTCATCGAGCTGGATTTATG
TACAAAATTTTGTACTTGTCTGCATGGGAGAAAGAAGGAGAAAGTGAAAAGCATATGAATTGGGTCCGAAGTG
CATATAATTTCATGTCTCCTTATGTGTCTCAAAATCCAAGAGCTACATATCTCAATTATAGGGACCTTGATTT
GGGAACAAATAACGAGAAGGGTCCTATTAGTTACTCACAAGCAAGTGTTTGGGGTAAAAAGTATTTCAGTATG
AACTTTAAGAGGTTAGTTAATGTGAAAACCAAGGTCGATCCAAGTAATTTCTTTAGAAACGAACAAAGCATCC
CACCACTTCTGTCGCGACGCCTCTAA
