# Supplementary material for: Resurrected Ancestral Cannabis Enzymes Unveil the Origin and Functional Evolution of Cannabinoid Synthases
Source: Plant Biotechnol J. 2025 Dec 26;24(4):2685–97. doi: 10.1111/pbi.70475 (PMC13140220; doi:10.1111/pbi.70475)
Supplement: Supplementary file 1 — Figure S1: Phylogeny of Cannabaceae‐specific Berberine Bridge‐Like genes. Figure S2: Syntenic blocks comprising cannabinoid synthase genes and closely‐related BBLs. Figure S3: Evaluation of enzyme expression by immunodetection. Figure S4: Determination of the optimal pH and reactional temperature for the activity of Ca. Figure S5: Determination of the optimal pH for the activity of HCa → CaSBR and Ca → CBDASSBR_FAD. Figure S6: Design and structure of the THCAS → CBDAS hybrid. Table S1: Analysis of the reconstructed ancestral sequences. Table S2: Design of the HCa → Ca (a), Ca → CBDAS (b) and Ca → A1A2a (c) hybrids, based on sequence and structural comparison. Table S3: Expression level of candidate enzymes (μg mL−1). Table S4: Comparison of the mutations tested in previous studies with mutations included in our hybrids. Table S5: Quality assessment of the three‐dimensional (3D) enzyme homology models. Data S1: Sequence alignment used to generate the gene‐tree and reconstruct the ancestors. Data S2: Ancestral sequences reconstructed with MrBayes and PAML. Data S3: Sequences of A1A1a, Ca and HCa. Data S4: Domesticated sequences used to express and characterise enzymes. Data S5: Berberine Bridge‐Like dataset. Data S6: Ancestral sequence reconstruction with MrBayes. Data S7: Ancestral sequence reconstruction with PAML. [file PBI-24-2685-s001.zip › pbi70475-sup-0010-DataS6.docx]

begin mrbayes;

set autoclose=yes nowarn=yes;

charset codonPos1 = 1-1873\3;

charset codonPos2 = 2-1874\3;

charset codonPos3 = 3-1875\3;

partition currentPartition = 2: codonPos1 codonPos2, codonPos3;

set partition = currentPartition;

lset applyto=(all);

lset nst=mixed rates=gamma;

unlink statefreq=(all) revmat=(all) shape=(all) pinvar=(all);

prset applyto=(all) ratepr=variable;

constraint CladeOfInterest = CBDRx_NC_044378_1_25870545_A2 Jamaican_Lion_JAATIP010000055_1_F8388_016308_A2 Jamaican_Lion_JAATIP010000055_1_F8388_016312_A2 JLion_father_JAATIQ010001028_1_G4B88_000693_A2 CBDRx_NC_044378_1_LOC115697880_A2 KT876030_1_A2 KJ469380_1_A2 KT876023_1_A2 AB212836_1_A2 KT876028_1_A2 KT876014_1_A2 Jilong_CM022965_1_91579693_A1 KT876046_1_A1 Jamaican_Lion_JAATIP010000026_1_F8388_024901_A1 KP970855_1_A1 LC120319_1_A1 KP970851_1_A1 KP970853_1_A1 MN422090_1_A1 MN422091_1_A1 MN422085_1_A1 MN422086_1_A1;

prset topologypr=constraints(CladeOfInterest);

report applyto=(all) ancstates=yes;

mcmcp ngen= 10000000 temp=0.05 relburnin=yes burninfrac=0.25 printfreq=2000 samplefreq=20000 nchains=4;

mcmc;

sumt;

sump;

end;
