# Supplementary material for: Resurrected Ancestral Cannabis Enzymes Unveil the Origin and Functional Evolution of Cannabinoid Synthases
Source: Plant Biotechnol J. 2025 Dec 26;24(4):2685–97. doi: 10.1111/pbi.70475 (PMC13140220; doi:10.1111/pbi.70475)
Supplement: Supplementary file 1 — Figure S1: Phylogeny of Cannabaceae‐specific Berberine Bridge‐Like genes. Figure S2: Syntenic blocks comprising cannabinoid synthase genes and closely‐related BBLs. Figure S3: Evaluation of enzyme expression by immunodetection. Figure S4: Determination of the optimal pH and reactional temperature for the activity of Ca. Figure S5: Determination of the optimal pH for the activity of HCa → CaSBR and Ca → CBDASSBR_FAD. Figure S6: Design and structure of the THCAS → CBDAS hybrid. Table S1: Analysis of the reconstructed ancestral sequences. Table S2: Design of the HCa → Ca (a), Ca → CBDAS (b) and Ca → A1A2a (c) hybrids, based on sequence and structural comparison. Table S3: Expression level of candidate enzymes (μg mL−1). Table S4: Comparison of the mutations tested in previous studies with mutations included in our hybrids. Table S5: Quality assessment of the three‐dimensional (3D) enzyme homology models. Data S1: Sequence alignment used to generate the gene‐tree and reconstruct the ancestors. Data S2: Ancestral sequences reconstructed with MrBayes and PAML. Data S3: Sequences of A1A1a, Ca and HCa. Data S4: Domesticated sequences used to express and characterise enzymes. Data S5: Berberine Bridge‐Like dataset. Data S6: Ancestral sequence reconstruction with MrBayes. Data S7: Ancestral sequence reconstruction with PAML. [file PBI-24-2685-s001.zip › pbi70475-sup-0011-DataS7.docx]

a)

seqfile = Align.phy

treefile = Tree.trees

outfile = mlb

noisy = 3

verbose = 2

runmode = 0

model = 7

Mgene = 4

fix_kappa = 0

kappa = 2.5

fix_alpha = 0

alpha = 1

Malpha = 0

ncatG = 5

nparK = 0

clock = 0

nhomo = 0

getSE = 0

RateAncestor = 1

Small_Diff = 7e-6

cleandata = 0

method = 1

fix_blength = 1

b)

seqfile = Align.phy

treefile = Tree.trees

outfile = mlb

noisy = 3

verbose = 2

runmode = 0

seqtype = 1

model = 0

Mgene = 0

fix_kappa = 0

kappa = 2

fix_omega = 0

omega = 0.4

fix_alpha = 0

alpha = 0.5

Malpha = 1

ncatG = 20

clock = 0

getSE = 0

RateAncestor = 1

CodonFreq = 1

aaDist = 1

NSsites = 0

Small_Diff = 7e-6

cleandata = 0

method = 1

fix_blength = 1

icode = 0

c)

seqfile = Align.phy

treefile = Tree.trees

outfile = mlb

noisy = 3

verbose = 2

runmode = 0

seqtype = 2

model = 3

Mgene = 0

fix_kappa = 0

kappa = 2

fix_omega = 0

omega = 0.4

fix_alpha = 0

alpha = 0.5

Malpha = 1

ncatG = 10

clock = 0

getSE = 0

RateAncestor = 1

aaRatefile = jones.dat

Small_Diff = 7e-6

cleandata = 0

method = 1

fix_blength = 1

icode = 0
